# Global, regional, and national disability-adjusted life-years (DALYs) for 333 diseases and injuries and healthy life expectancy (HALE) for 195 countries and territories, 1990–2016: a systematic analysis for the Global Burden of Disease Study 2016

**DOI:** 10.1016/S0140-6736(17)32130-X

**Published:** 2017-09-16

**Authors:** GBD 2016 DALYs and HALE Collaborators, Amanuel Alemu Abajobir, Kalkidan Hassen Abate, Cristiana Abbafati, Kaja M Abbas, Foad Abd-Allah, Rizwan Suliankatchi Abdulkader, Abdishakur M Abdulle, Teshome Abuka Abebo, Semaw Ferede Abera, Victor Aboyans, Laith J Abu-Raddad, Ilana N Ackerman, Isaac A Adedeji, Olatunji Adetokunboh, Ashkan Afshin, Rakesh Aggarwal, Sutapa Agrawal, Anurag Agrawal, Muktar Beshir Ahmed, Miloud Taki Eddine Aichour, Amani Nidhal Aichour, Ibtihel Aichour, Sneha Aiyar, Tomi F Akinyemiju, Nadia Akseer, Faris Hasan Al Lami, Fares Alahdab, Ziyad Al-Aly, Khurshid Alam, Noore Alam, Tahiya Alam, Deena Alasfoor, Kefyalew Addis Alene, Raghib Ali, Reza Alizadeh-Navaei, Juma M Alkaabi, Ala'a Alkerwi, François Alla, Peter Allebeck, Christine Allen, Fatma Al-Maskari, Mohammad AbdulAziz AlMazroa, Rajaa Al-Raddadi, Ubai Alsharif, Shirina Alsowaidi, Benjamin M Althouse, Khalid A Altirkawi, Nelson Alvis-Guzman, Azmeraw T Amare, Erfan Amini, Walid Ammar, Yaw Ampem Amoako, Mustafa Geleto Ansha, Carl Abelardo T Antonio, Palwasha Anwari, Johan Ärnlöv, Megha Arora, Al Artaman, Krishna Kumar Aryal, Solomon W Asgedom, Tesfay Mehari Atey, Niguse Tadele Atnafu, Leticia Avila-Burgos, Euripide Frinel G Arthur Avokpaho, Ashish Awasthi, Shally Awasthi, Mahmoud Reza Azarpazhooh, Peter Azzopardi, Tesleem Kayode Babalola, Umar Bacha, Alaa Badawi, Kalpana Balakrishnan, Marlena S Bannick, Aleksandra Barac, Suzanne L Barker-Collo, Till Bärnighausen, Simon Barquera, Lope H Barrero, Sanjay Basu, Robert Battista, Katherine E Battle, Bernhard T Baune, Shahrzad Bazargan-Hejazi, Justin Beardsley, Neeraj Bedi, Yannick Béjot, Bayu Begashaw Bekele, Michelle L Bell, Derrick A Bennett, James R Bennett, Isabela M Bensenor, Jennifer Benson, Adugnaw Berhane, Derbew Fikadu Berhe, Eduardo Bernabé, Balem Demtsu Betsu, Mircea Beuran, Addisu Shunu Beyene, Anil Bhansali, Samir Bhatt, Zulfiqar A Bhutta, Sibhatu Biadgilign, Burcu Kucuk Bicer, Kelly Bienhoff, Boris Bikbov, Charles Birungi, Stan Biryukov, Donal Bisanzio, Habtamu Mellie Bizuayehu, Fiona M Blyth, Dube Jara Boneya, Dipan Bose, Ibrahim R Bou-Orm, Rupert R A Bourne, Michael Brainin, Carol Brayne, Alexandra Brazinova, Nicholas J K Breitborde, Paul S Briant, Gabrielle Britton, Traolach S Brugha, Rachelle Buchbinder, Lemma Negesa Bulto Bulto, Blair R Bumgarner, Zahid A Butt, Lucero Cahuana-Hurtado, Ewan Cameron, Ismael Ricardo Campos-Nonato, Hélène Carabin, Rosario Cárdenas, David O Carpenter, Juan Jesus Carrero, Austin Carter, Felix Carvalho, Daniel Casey, Carlos A Castañeda-Orjuela, Chris D Castle, Ferrán Catalá-López, Jung-Chen Chang, Fiona J Charlson, Pankaj Chaturvedi, Honglei Chen, Mirriam Chibalabala, Chioma Ezinne Chibueze, Vesper Hichilombwe Chisumpa, Abdulaal A Chitheer, Rajiv Chowdhury, Devasahayam Jesudas Christopher, Liliana G Ciobanu, Massimo Cirillo, Danny Colombara, Leslie Trumbull Cooper, Cyrus Cooper, Paolo Angelo Cortesi, Monica Cortinovis, Michael H Criqui, Elizabeth A Cromwell, Marita Cross, John A Crump, Abel Fekadu Dadi, Koustuv Dalal, Albertino Damasceno, Lalit Dandona, Rakhi Dandona, José das Neves, Dragos V Davitoiu, Kairat Davletov, Barbora de Courten, Diego De Leo, Hans De Steur, Barthelemy Kuate Defo, Louisa Degenhardt, Selina Deiparine, Robert P Dellavalle, Kebede Deribe, Amare Deribew, Don C Des Jarlais, Subhojit Dey, Samath D Dharmaratne, Preet K Dhillon, Daniel Dicker, Shirin Djalainia, Huyen Phuc Do, Klara Dokova, David Teye Doku, E Ray Dorsey, Kadine Priscila Bender dos Santos, Tim R Driscoll, Manisha Dubey, Bruce Bartholow Duncan, Beth E Ebel, Michelle Echko, Ziad Ziad El-Khatib, Ahmadali Enayati, Aman Yesuf Endries, Sergey Petrovich Ermakov, Holly E Erskine, Setegn Eshetie, Babak Eshrati, Alireza Esteghamati, Kara Estep, Fanuel Belayneh Bekele Fanuel, Tamer Farag, Carla Sofia e Sa Farinha, André Faro, Farshad Farzadfar, Mir Sohail Fazeli, Valery L Feigin, Andrea B Feigl, Seyed-Mohammad Fereshtehnejad, João C Fernandes, Alize J Ferrari, Tesfaye Regassa Feyissa, Irina Filip, Florian Fischer, Christina Fitzmaurice, Abraham D Flaxman, Nataliya Foigt, Kyle J Foreman, Richard C Franklin, Joseph J Frostad, Nancy Fullman, Thomas Fürst, Joao M Furtado, Neal D Futran, Emmanuela Gakidou, Alberto L Garcia-Basteiro, Teshome Gebre, Gebremedhin Berhe Gebregergs, Tsegaye Tewelde Gebrehiwot, Johanna M Geleijnse, Ayele Geleto, Bikila Lencha Gemechu, Hailay Abrha Gesesew, Peter W Gething, Alireza Ghajar, Katherine B Gibney, Richard F Gillum, Ibrahim Abdelmageem Mohamed Ginawi, Melkamu Dedefo Gishu, Giorgia Giussani, William W Godwin, Kashish Goel, Shifalika Goenka, Ellen M Goldberg, Philimon N Gona, Amador Goodridge, Sameer Vali Gopalani, Richard A Gosselin, Carolyn C Gotay, Atsushi Goto, Alessandra Carvalho Goulart, Nicholas Graetz, Harish Chander Gugnani, Prakash C Gupta, Rajeev Gupta, Tanush Gupta, Vipin Gupta, Rahul Gupta, Reyna A Gutiérrez, Vladimir Hachinski, Nima Hafezi-Nejad, Alemayehu Desalegne Hailu, Gessessew Bugssa Hailu, Randah Ribhi Hamadeh, Samer Hamidi, Mouhanad Hammami, Alexis J Handal, Graeme J Hankey, Yuantao Hao, Hilda L Harb, Habtamu Abera Hareri, Josep Maria Haro, Kimani M Harun, James Harvey, Mohammad Sadegh Hassanvand, Rasmus Havmoeller, Simon I Hay, Roderick J Hay, Mohammad T Hedayati, Delia Hendrie, Nathaniel J Henry, Ileana Beatriz Heredia-Pi, Pouria Heydarpour, Hans W Hoek, Howard J Hoffman, Masako Horino, Nobuyuki Horita, H Dean Hosgood, Sorin Hostiuc, Peter J Hotez, Damian G Hoy, Aung Soe Htet, Guoqing Hu, John J Huang, Chantal Huynh, Kim Moesgaard Iburg, Ehimario Uche Igumbor, Chad Ikeda, Caleb Mackay Salpeter Irvine, Sheikh Mohammed Shariful Islam, Kathryn H Jacobsen, Nader Jahanmehr, Mihajlo B Jakovljevic, Peter James, Simerjot K Jassal, Mehdi Javanbakht, Sudha P Jayaraman, Panniyammakal Jeemon, Paul N Jensen, Vivekanand Jha, Guohong Jiang, Denny John, Catherine O Johnson, Sarah Charlotte Johnson, Jost B Jonas, Mikk Jürisson, Zubair Kabir, Rajendra Kadel, Amaha Kahsay, Ritul Kamal, Chittaranjan Kar, Nadim E Karam, André Karch, Corine Kakizi Karema, Seyed M Karimi, Chante Karimkhani, Amir Kasaeian, Getachew Mullu Kassa, Nigussie Assefa Kassaw, Nicholas J Kassebaum, Anshul Kastor, Srinivasa Vittal Katikireddi, Anil Kaul, Norito Kawakami, Peter Njenga Keiyoro, Laura Kemmer, Andre Pascal Kengne, Andre Keren, Chandrasekharan Nair Kesavachandran, Yousef Saleh Khader, Ibrahim A Khalil, Ejaz Ahmad Khan, Young-Ho Khang, Abdullah T Khoja, Ardeshir Khosravi, Jagdish Khubchandani, Aliasghar Ahmad Kiadaliri, Christian Kieling, Yun Jin Kim, Daniel Kim, Ruth W Kimokoti, Yohannes Kinfu, Adnan Kisa, Katarzyna A Kissimova-Skarbek, Niranjan Kissoon, Mika Kivimaki, Ann Kristin Knudsen, Yoshihiro Kokubo, Dhaval Kolte, Jacek A Kopec, Soewarta Kosen, Georgios A Kotsakis, Parvaiz A Koul, Ai Koyanagi, Michael Kravchenko, Kristopher J Krohn, G Anil Kumar, Pushpendra Kumar, Hmwe H Kyu, Anton Carl Jonas Lager, Dharmesh Kumar Lal, Ratilal Lalloo, Tea Lallukka, Nkurunziza Lambert, Qing Lan, Van C Lansingh, Anders Larsson, Janet L Leasher, Paul H Lee, James Leigh, Cheru Tesema Leshargie, Janni Leung, Ricky Leung, Miriam Levi, Yichong Li, Yongmei Li, Xiaofeng Liang, Misgan Legesse Liben, Stephen S Lim, Shai Linn, Patrick Y Liu, Angela Liu, Shiwei Liu, Yang Liu, Rakesh Lodha, Giancarlo Logroscino, Katharine J Looker, Alan D Lopez, Stefan Lorkowski, Paulo A Lotufo, Rafael Lozano, Timothy C D Lucas, Raimundas Lunevicius, Ronan A Lyons, Erlyn Rachelle King Macarayan, Emilie R Maddison, Hassan Magdy Abd Magdy Abd El Razek, Mohammed Magdy Abd El Razek, Carlos Magis-Rodriguez, Mahdi Mahdavi, Marek Majdan, Reza Majdzadeh, Azeem Majeed, Reza Malekzadeh, Rajesh Malhotra, Deborah Carvalho Malta, Abdullah A Mamun, Helena Manguerra, Treh Manhertz, Lorenzo G Mantovani, Chabila C Mapoma, Lyn M March, Laurie B Marczak, Jose Martinez-Raga, Paulo Henrique Viegas Martins, Francisco Rogerlândio Martins-Melo, Ira Martopullo, Winfried März, Manu Raj Mathur, Mohsen Mazidi, Colm McAlinden, Madeline McGaughey, John J McGrath, Martin McKee, Suresh Mehata, Toni Meier, Kidanu Gebremariam Meles, Peter Memiah, Ziad A Memish, Walter Mendoza, Melkamu Merid Mengesha, Mubarek Abera Mengistie, Desalegn Tadese Mengistu, George A Mensah, Tuomo J Meretoja, Atte Meretoja, Haftay Berhane Mezgebe, Renata Micha, Anoushka Millear, Ted R Miller, Shawn Minnig, Mojde Mirarefin, Erkin M Mirrakhimov, Awoke Misganaw, Shiva Raj Mishra, Philip B Mitchell, Karzan Abdulmuhsin Mohammad, Alireza Mohammadi, Muktar Sano Kedir Mohammed, Kedir Endris Mohammed, Shafiu Mohammed, Murali B V Mohan, Ali H Mokdad, Sarah K Mollenkopf, Lorenzo Monasta, Julio Cesar Montañez Hernandez, Marcella Montico, Maziar Moradi-Lakeh, Paula Moraga, Lidia Morawska, Rintaro Mori, Shane D Morrison, Mark Moses, Cliff Mountjoy-Venning, Kalayu Birhane Mruts, Ulrich O Mueller, Kate Muller, Michele E Murdoch, Christopher J L Murray, Gudlavalleti Venkata Satyanarayana Murthy, Srinivas Murthy, Kamarul Imran Musa, Jean B Nachega, Gabriele Nagel, Mohsen Naghavi, Aliya Naheed, Kovin S Naidoo, Vinay Nangia, Jamal T Nasher, Gopalakrishnan Natarajan, Dumessa Edessa Negasa, Ruxandra Irina Negoi, Ionut Negoi, Charles R Newton, Josephine Wanjiku Ngunjiri, Cuong Tat Nguyen, Quyen Le Nguyen, Trang Huyen Nguyen, Grant Nguyen, Minh Nguyen, Emma Nichols, Dina Nur Anggraini Ningrum, Vuong Minh Nong, Ole F Norheim, Bo Norrving, Jean Jacques N Noubiap, Alypio Nyandwi, Carla Makhlouf Obermeyer, Martin J O'Donnell, Felix Akpojene Ogbo, In-Hwan Oh, Anselm Okoro, Olanrewaju Oladimeji, Andrew Toyin Olagunju, Tinuke Oluwasefunmi Olagunju, Helen E Olsen, Bolajoko Olubukunola Olusanya, Jacob Olusegun Olusanya, Kanyin Ong, John Nelson Opio, Eyal Oren, Alberto Ortiz, Richard H Osborne, Aaron Osgood-Zimmerman, Majdi Osman, Erika Ota, Mayowa O Owolabi, Mahesh PA, Rosana E Pacella, Basant Kumar Panda, Jeyaraj Durai Pandian, Christina Papachristou, Eun-Kee Park, Charles D Parry, Mahboubeh Parsaeian, Snehal T Patil, Scott B Patten, George C Patton, Deepak Paudel, Katherine Paulson, Neil Pearce, David M Pereira, Krystle Marie Perez, Norberto Perico, Konrad Pesudovs, Carrie Beth Peterson, William Arthur Petri, Max Petzold, Michael Robert Phillips, Geoffrey Phipps, David M Pigott, Julian David Pillay, Christine Pinho, Michael A Piradov, Dietrich Plass, Martin A Pletcher, Svetlana Popova, Richie G Poulton, Farshad Pourmalek, Dorairaj Prabhakaran, Narayan Prasad, Carrie Purcell, Manorama Purwar, Mostafa Qorbani, Beatriz Paulina Ayala Quintanilla, Rynaz H S Rabiee, Amir Radfar, Anwar Rafay, Kazem Rahimi, Afarin Rahimi-Movaghar, Vafa Rahimi-Movaghar, Mohammad Hifz Ur Rahman, Muhammad Aziz Rahman, Mahfuzar Rahman, Rajesh Kumar Rai, Sasa Rajsic, Usha Ram, Chhabi Lal Ranabhat, Thara Rangaswamy, Zane Rankin, Paturi Vishnupriya Rao, Puja C Rao, Salman Rawaf, Sarah E Ray, Robert C Reiner, Nikolas Reinig, Marissa Reitsma, Giuseppe Remuzzi, Andre M N Renzaho, Serge Resnikoff, Satar Rezaei, Antonio L Ribeiro, Jacqueline Castillo Rivas, Hirbo Shore Roba, Stephen R Robinson, David Rojas-Rueda, Mohammad Bagher Rokni, Luca Ronfani, Gholamreza Roshandel, Gregory A Roth, Dietrich Rothenbacher, Ambuj Roy, Enrico Rubagotti, George Mugambage Ruhago, Soheil Saadat, Mahdi Safdarian, Saeid Safiri, Rajesh Sagar, Ramesh Sahathevan, Mohammad Ali Sahraian, Joseph Salama, Muhammad Muhammad Saleh, Joshua A Salomon, Sundeep Santosh Salvi, Abdallah M Samy, Juan Ramon Sanabria, Maria Dolores Sanchez-Niño, Damian Santomauro, João Vasco Santos, Itamar S Santos, Milena M Santric Milicevic, Benn Sartorius, Maheswar Satpathy, Monika Sawhney, Sonia Saxena, Kathryn Schelonka, Maria Inês Schmidt, Ione J C Schneider, Ben Schöttker, Aletta E Schutte, David C Schwebel, Falk Schwendicke, Soraya Seedat, Sadaf G Sepanlou, Edson E Servan-Mori, Amira Shaheen, Masood Ali Shaikh, Mansour Shamsipour, Rajesh Sharma, Jayendra Sharma, Jun She, Peilin Shi, Kenji Shibuya, Chloe Shields, Girma Temam Shifa, Mekonnen Sisay Shiferaw, Mika Shigematsu, Rahman Shiri, Reza Shirkoohi, Shreya Shirude, Kawkab Shishani, Haitham Shoman, Soraya Siabani, Abla Mehio Sibai, Inga Dora Sigfusdottir, Donald H Silberberg, Diego Augusto Santos Silva, João Pedro Silva, Dayane Gabriele Alves Silveira, Jasvinder A Singh, Om Prakash Singh, Narinder Pal Singh, Virendra Singh, Dhirendra Narain Sinha, Eirini Skiadaresi, Erica Leigh Slepak, David L Smith, Mari Smith, Badr H A Sobaih, Eugene Sobngwi, Michael Soljak, Reed J D Sorensen, Tatiane Cristina Moraes Sousa, Luciano A Sposato, Chandrashekhar T Sreeramareddy, Vinay Srinivasan, Jeffrey D Stanaway, Vasiliki Stathopoulou, Nicholas Steel, Dan J Stein, Caitlyn Steiner, Sabine Steinke, Mark Andrew Stokes, Lars Jacob Stovner, Bryan Strub, Michelle Subart, Muawiyyah Babale Sufiyan, Bruno F Sunguya, Patrick J Sur, Soumya Swaminathan, Bryan L Sykes, Dillon Sylte, Cassandra E I Szoeke, Rafael Tabarés-Seisdedos, Santosh Kumar Tadakamadla, Getachew Redae Taffere, Jukka S Takala, Nikhil Tandon, David Tanne, Yihunie L Tarekegn, Mohammad Tavakkoli, Nuno Taveira, Hugh R Taylor, Teketo Kassaw Tegegne, Arash Tehrani-Banihashemi, Tesfalidet Tekelab, Abdullah Sulieman Terkawi, Dawit Jember Tesfaye, Belay Tesssema, JS Thakur, Ornwipa Thamsuwan, Alice M Theadom, Andrew M Theis, Katie E Thomas, Nihal Thomas, Robert Thompson, Amanda G Thrift, Ruoyan Tobe-Gai, Myriam Tobollik, Marcello Tonelli, Roman Topor-Madry, Miguel Tortajada, Mathilde Touvier, Jefferson Traebert, Bach Xuan Tran, Christopher Troeger, Thomas Truelsen, Derrick Tsoi, Emin Murat Tuzcu, Hayley Tymeson, Stefanos Tyrovolas, Kingsley Nnanna Ukwaja, Eduardo A Undurraga, Chigozie Jesse Uneke, Rachel Updike, Olalekan A Uthman, Benjamin S Chudi Uzochukwu, Job F M van Boven, Santosh Varughese, Tommi Vasankari, Lennert J Veerman, S Venkatesh, Narayanaswamy Venketasubramanian, Ramesh Vidavalur, Lakshmi Vijayakumar, Francesco S Violante, Abhishek Vishnu, Sergey K Vladimirov, Vasiliy Victorovich Vlassov, Stein Emil Vollset, Theo Vos, Fiseha Wadilo, Tolassa Wakayo, Mitchell T Wallin, Yuan-Pang Wang, Scott Weichenthal, Elisabete Weiderpass, Robert G Weintraub, Daniel J Weiss, Andrea Werdecker, Ronny Westerman, Harvey A Whiteford, Tissa Wijeratne, Hywel C Williams, Charles Shey Wiysonge, Belete Getahun Woldeyes, Charles D A Wolfe, Rachel Woodbrook, Anthony D Woolf, Abdulhalik Workicho, Denis Xavier, Gelin Xu, Simon Yadgir, Mohsen Yaghoubi, Bereket Yakob, Lijing L Yan, Yuichiro Yano, Pengpeng Ye, Mahari Gidey Yihdego, Hassen Hamid Yimam, Paul Yip, Naohiro Yonemoto, Seok-Jun Yoon, Marcel Yotebieng, Mustafa Z Younis, Chuanhua Yu, Zoubida Zaidi, Maysaa El Sayed Zaki, Elias Asfaw Zegeye, Zerihun Menlkalew Zenebe, Xueying Zhang, Yingfeng Zheng, Maigeng Zhou, Ben Zipkin, Sanjay Zodpey, Leo Zoeckler, Liesl Joanna Zuhlke

## Abstract

**Background:**

Measurement of changes in health across locations is useful to compare and contrast changing epidemiological patterns against health system performance and identify specific needs for resource allocation in research, policy development, and programme decision making. Using the Global Burden of Diseases, Injuries, and Risk Factors Study 2016, we drew from two widely used summary measures to monitor such changes in population health: disability-adjusted life-years (DALYs) and healthy life expectancy (HALE). We used these measures to track trends and benchmark progress compared with expected trends on the basis of the Socio-demographic Index (SDI).

**Methods:**

We used results from the Global Burden of Diseases, Injuries, and Risk Factors Study 2016 for all-cause mortality, cause-specific mortality, and non-fatal disease burden to derive HALE and DALYs by sex for 195 countries and territories from 1990 to 2016. We calculated DALYs by summing years of life lost and years of life lived with disability for each location, age group, sex, and year. We estimated HALE using age-specific death rates and years of life lived with disability per capita. We explored how DALYs and HALE differed from expected trends when compared with the SDI: the geometric mean of income per person, educational attainment in the population older than age 15 years, and total fertility rate.

**Findings:**

The highest globally observed HALE at birth for both women and men was in Singapore, at 75·2 years (95% uncertainty interval 71·9–78·6) for females and 72·0 years (68·8–75·1) for males. The lowest for females was in the Central African Republic (45·6 years [42·0–49·5]) and for males was in Lesotho (41·5 years [39·0–44·0]). From 1990 to 2016, global HALE increased by an average of 6·24 years (5·97–6·48) for both sexes combined. Global HALE increased by 6·04 years (5·74–6·27) for males and 6·49 years (6·08–6·77) for females, whereas HALE at age 65 years increased by 1·78 years (1·61–1·93) for males and 1·96 years (1·69–2·13) for females. Total global DALYs remained largely unchanged from 1990 to 2016 (–2·3% [–5·9 to 0·9]), with decreases in communicable, maternal, neonatal, and nutritional (CMNN) disease DALYs offset by increased DALYs due to non-communicable diseases (NCDs). The exemplars, calculated as the five lowest ratios of observed to expected age-standardised DALY rates in 2016, were Nicaragua, Costa Rica, the Maldives, Peru, and Israel. The leading three causes of DALYs globally were ischaemic heart disease, cerebrovascular disease, and lower respiratory infections, comprising 16·1% of all DALYs. Total DALYs and age-standardised DALY rates due to most CMNN causes decreased from 1990 to 2016. Conversely, the total DALY burden rose for most NCDs; however, age-standardised DALY rates due to NCDs declined globally.

**Interpretation:**

At a global level, DALYs and HALE continue to show improvements. At the same time, we observe that many populations are facing growing functional health loss. Rising SDI was associated with increases in cumulative years of life lived with disability and decreases in CMNN DALYs offset by increased NCD DALYs. Relative compression of morbidity highlights the importance of continued health interventions, which has changed in most locations in pace with the gross domestic product per person, education, and family planning. The analysis of DALYs and HALE and their relationship to SDI represents a robust framework with which to benchmark location-specific health performance. Country-specific drivers of disease burden, particularly for causes with higher-than-expected DALYs, should inform health policies, health system improvement initiatives, targeted prevention efforts, and development assistance for health, including financial and research investments for all countries, regardless of their level of sociodemographic development. The presence of countries that substantially outperform others suggests the need for increased scrutiny for proven examples of best practices, which can help to extend gains, whereas the presence of underperforming countries suggests the need for devotion of extra attention to health systems that need more robust support.

**Funding:**

Bill & Melinda Gates Foundation.

## Introduction

Objective measurement of population health is a fundamental requirement of good governance that allows international, regional, national, and local actors to frame evidence-based policy informed by past trends and current performance of health systems.^1^, ^2^, ^3^, ^4^ Summary measures of population health include techniques that measure the overall burden of health loss due to fatal and non-fatal diseases, as well as measures of expected fatal and non-fatal disease burden based on Socio-demographic Index (SDI).^5^ The disability-adjusted life-year (DALY) measures health loss due to both fatal and non-fatal disease burden. DALYs are the sum of the years of life lost (YLLs) due to premature mortality and years of life lived with disability (YLDs).^6^ The YLL is based on remaining life expectancy when compared with a reference standard life table at age of death,^7^ and the YLD is calculated by multiplying the prevalence of a disease or injury and its main disabling outcomes by its weighted level of severity.^6^, ^8^ One DALY represents 1 year of healthy life lost. Examination of levels and trends of DALYs facilitates quick comparison between different diseases and injuries. Conversely, healthy life expectancy (HALE), a metric based on methods by Sullivan,^9^ provides a single summary measure of population health across all causes combined by weighting years lived with a measure of functional health loss before death and is the most comprehensive among competing expectancy metrics.^1^, ^2^, ^3^, ^4^ Together, DALYs and HALE enable comparisons of the magnitude of functional health loss across societies due to diseases, injuries, and risk factors, against which provisioning and performance of health systems can be calibrated.^4^

**Research in context**

**Evidence before this study**

The Global Burden of Diseases, Injuries, and Risk Factors Study 2015 (GBD 2015) provided disability-adjusted life-year (DALY) estimates for 315 diseases and injuries for 195 countries and territories, including subnational assessments for 11 countries and, thus, for a total of 519 locations, from 1990 to 2015. GBD 2015 also introduced analyses of DALYs and healthy life expectancy (HALE) in relation to the Socio-demographic Index (SDI). Only the WHO Global Health Estimates has published updated estimates of DALYs, and these estimates were heavily reliant on GBD 2015 results.

**Added value of this study**

This study, the Global Burden of Diseases, Injuries, and Risk Factors Study 2016 (GBD 2016), updates and improves the first of the annual Global Burden of Disease iterations, GBD 2015. GBD 2016 is, to our knowledge, the only peer-reviewed, Guidelines for Accurate and Transparent Health Estimates Reporting-compliant, comprehensive, and annual assessment of DALYs and HALE by age group, sex, cause, and location, analysed consistently from 1990 to 2016. The improved approaches to the analysis and refinements in data (gap fills, updates, and revisions), as well as the widening of scope by cause, location, age, and time are all relevant to this study. The summary population health metrics of DALYs and HALE synthesise the cumulative effect of all of these improvements, the most notable of which are as follows. First, we added substantial location-years of cause-specific mortality data and non-fatal data for GBD 2016. The added data progressively fill gaps in the period of estimation, most substantially for India. Second, many analytical methods have been improved, such as improvement of mortality to incidence ratios for cancers to better reflect lower survival than in GBD 2015 and for non-fatal tuberculosis to better reflect higher incidence in low-income and middle-income countries based on SDI. Third, we included new subnational assessments for Indonesia at the provincial level and further disaggregated subnational estimation in England to the local government area level. Fourth, we refined our estimation of age-specific outcomes for ages 80 years and older into 5 year groups extending to age 95 years and older to better account for disease burden in elderly populations than in GBD 2015. Fifth, we estimated DALYs for several additional causes for the first time. Sixth, we improved our analysis of the epidemiological transition as a function of SDI, which allowed for a more nuanced interpretation of global health trends against the sociodemographic development spectrum than in GBD 2015. Finally, we used these analyses to identify the exemplar countries that exceeded population health summary metric expectations relative to their SDI position alone. The GBD 2016 iteration supersedes all previous GBD studies of DALYs and HALE and re-estimates these measures for the complete time series from 1990 to 2016. We focus on new methods and approaches since GBD 2015 and highlight nations that overperformed or underperformed on the basis of what would be expected on the basis of their SDI.

**Implications of all the available evidence**

The epidemiological transition continues apace globally, with a shift from DALYs attributable to communicable, maternal, neonatal, and nutritional diseases to those attributable to non-communicable diseases. This progression is concomitant with improvements in SDI and thus improvements in education, fertility rates, and economic status. A more detailed analysis than in this study of the epidemiological changes that have occurred in countries that have consistently exceeded expectations could provide improved insights into good practice in public health policy, which might be emulated elsewhere. A similarly detailed appraisal of countries that are lagging in DALYs and HALE relative to expectations on the level of SDI alone will help identify countries in most need of domestic and international attention across the development continuum.

As the second in a series^7^, ^8^, ^10^, ^11^ of now annual updates, the Global Burden of Diseases, Injuries, and Risk Factors Study 2016 (GBD 2016) is the most comprehensive and current source of summary health metrics. The Global Burden of Disease (GBD) is based on development of the largest available database of health outcomes, risk factor exposure, intervention coverage, and sociodemographic factors related to health. We applied analytical techniques to reduce data biases and support comparability, propagated the uncertainty in these estimates, and provided insights at the highest temporal and spatial resolution afforded by the data.

The purpose of this study is to present the results of GBD 2016 for DALYs and HALE, building on updated estimates of mortality, causes of death, and non-fatal health loss^7^, ^10^ to identify nations with notable variation in health performance from that expected on the basis of SDI. Approaches to the analysis have been previously described.^2^, ^3^, ^4^, ^12^ GBD 2016 improvements include addition of newly available retrospective data, refined analytical methods (such as improvement to mortality to incidence ratios [MIRs] for cancers to better reflect lower survival in low-income and middle-income countries based on SDI), new subnational estimation for England and Indonesia, disaggregation of certain cause groupings to capture greater detail, and expansion of older age groups to enhance relevance for a wider range of health policy decisions.^6^

## Methods

### Overview

We used the results of GBD 2016 to evaluate trends in epidemiological patterns and health performance on a global, regional, national, and subnational scale using DALYs and HALE as summary measures of changes in health states. Greater detail than presented in this section for methods used to estimate DALYs and HALE, including analytic approaches for assessment of relative morbidity and mortality from individual diseases and injuries, is provided in related publications in this series^8^, ^10^ and the appendix.

This analysis follows the Guidelines for Accurate and Transparent Health Estimates Reporting,^13^, ^14^ which include recommendations on documentation of data sources, estimation methods, and statistical analysis. We did analyses using Python version 2.7.12 and 2.7.3, Stata version 13.1, and R version 3.2.2. For more information on Guidelines for Accurate and Transparent Health Estimates Reporting compliance, please refer to the appendix (pp 13–15). Additionally, interactive online tools are available to explore GBD 2016 data sources in detail. Cause-specific estimation for GBD 2016 covers the years 1990–2016. For a subset of analyses, we focus on the last decade, from 2006 to 2016, to address current policy priorities. The GBD 2016 results for all years and by location can be explored further with dynamic data visualisations.

### Cause and location hierarchies

In the GBD 2016 study, causes of mortality and morbidity are structured with use of a four-level classification hierarchy to produce levels that are mutually exclusive and collectively exhaustive. GBD 2016 estimates 333 causes of DALYs, 68 of which are a source of disability but not a cause of death (such as trachoma, hookworm, and low back and neck pain) and five of which are causes of death but not sources of morbidity (sudden infant death syndrome, aortic aneurysm, late maternal deaths, indirect maternal deaths, and maternal deaths aggravated by HIV/AIDS). Within each level of the hierarchy, the number of collectively exhaustive and mutually exclusive fatal and non-fatal causes for which the GBD study estimates is three at Level 1, 21 at Level 2, 168 at Level 3, and 276 at Level 4. The full GBD cause hierarchy, including corresponding International Classification of Diseases (ICD)-9 and ICD-10 codes, is detailed in GBD 2016 publications on cause-specific mortality^10^ and non-fatal health outcomes,^8^ with cause-specific methods detailed in the corresponding appendices.

The GBD study is organised by a geographical hierarchy of seven super-regions containing 21 regions, with 195 countries and territories nested within those regions.^12^ GBD 2016 included new subnational assessments for Indonesia by province and for England by local government area. In this study, we present subnational data for the five countries with a population greater than 200 million people in 2016: Brazil, China, India, Indonesia, and the USA.

### Estimation of mortality and non-fatal health loss

To estimate all-cause and cause-specific mortality, the GBD study first systematically addressed known data challenges—such as variation in coding of causes or age group reporting, misclassification of deaths from HIV/AIDS, or methods for incorporation of population-based cancer registry data—using standardised methods described in detail in the GBD 2016 mortality^7^ and causes of death^10^ publications. As noted in other GBD publications, each death is attributed to a single underlying cause in accordance with the ICD. We take steps to standardise cause of death data to address the small fraction of deaths that are not assigned an age or sex, deaths assigned to broad age groups that are not 5 year age groups, and various revisions and national variants of the ICD. Additionally, we identify and redistribute deaths assigned to ICD codes that cannot be underlying causes of death, are intermediate causes of death rather than the underlying causes, or lack specificity in coding.^10^ We estimated cause-specific mortality using standardised modelling processes—most commonly, the Cause of Death Ensemble model, which uses covariate selection and out-of-sample validity analyses and generates estimates for each location-year, age, and sex.^10^ Additional detail, including model specifications and data availability for each cause-specific model, can be found in the appendix of the GBD 2016 mortality^7^ and causes of death^10^ publications. We used the all-cause mortality estimates to establish a reference life table from the lowest death rates for each age group among locations with total populations greater than 5 million.^7^ From this reference life table, we multiplied life expectancy at the age of death by cause-specific deaths to calculate cause-specific YLLs. We then used the GBD world population age standard to calculate age-standardised rates for deaths and YLLs.^7^ The GBD world population age standard and the standard life expectancies are available in the appendix of the GBD 2016 mortality publication.^7^

Changes implemented since the Global Burden of Diseases, Injuries, and Risk Factors Study 2015 (GBD 2015) for cause-specific mortality include incorporation of substantial sources of new mortality data; important model improvements for HIV, malaria, tuberculosis, injuries, diabetes, and cancers; disaggregation of specific causes into subgroupings to provide additional detail (the following were all estimated separately for the first time: alcoholic cardiomyopathy; urogenital, musculoskeletal, and digestive congenital anomalies; Zika virus disease; Guinea worm disease; self-harm by firearm; sexual violence; myocarditis; and the following types of tuberculosis: extensively drug-resistant tuberculosis, multidrug-resistant tuberculosis without extensive drug resistance, drug-susceptible tuberculosis, extensively drug-resistant HIV/AIDS-tuberculosis, multidrug-resistant HIV/AIDS-tuberculosis without extensive drug resistance, and drug-susceptible HIV/AIDS-tuberculosis); modelling of antiretroviral therapy (ART) coverage for each location-year by CD4-positive cell count at initiation; breakdown of terminal age groups from 80 years and older to 80–84 years, 85–89 years, 90–94 years, and 95 years and older; expansion of the GBD location hierarchy; and changes in the calculation of SDI.^10^ The database for GBD 2016 now includes data for the 333 causes estimated for DALYs and new subnational units for Indonesia (n=34) and England (n=150). For GBD 2016, we included substantial amounts of additional data sources from new studies and our network of collaborators; details of the types of data added can be found in the GBD 2016 cause of death^10^ and non-fatal^8^ publications. Additionally, research teams did systematic reviews to incorporate literature data into fatal and non-fatal models. Further details on search strings are available in the GBD 2016 non-fatal^8^ and cause of death^10^ publication appendices. The Registrar General of India provided improved verbal autopsy data collected through their Sample Registration System, enabling a more detailed and thorough analysis of subnational data for India than in GBD 2015. The methods for constructing the SDI, initially developed for GBD 2015,^15^ were revised for GBD 2016 to account for expansion in the number of subnational estimates and the effect of a growing time period of estimation given fixed limits for index components.^10^ The components of SDI—total fertility rate (TFR), educational attainment in the population aged older than 15 years, and lag-distributed income (LDI)—are based on new systematic assessments of educational attainment, LDI, and fertility, and each component is scaled relative to maximum effect on health outcomes.^10^

In most cases, we estimated non-fatal health loss using the Bayesian meta-regression tool DisMod-MR 2.1 to synthesise variable data sources to produce internally consistent estimates of incidence, prevalence, remission, and excess mortality.^16^ Cause-specific data availability and epidemiological characteristics required additional analytical techniques in some cases (details are available in the appendix of the GBD 2016 non-fatal publication^8^); these causes include many neglected tropical diseases (NTDs) such as dengue, as well as injuries, malaria, and HIV/AIDS.^17^, ^18^

We estimated each non-fatal sequela separately and assessed the occurrence of comorbidity in each age group, sex, location, and year separately using a microsimulation framework. We distributed disability estimated for comorbid conditions to each contributing cause during the comorbidity estimation process. Although the distribution of sequelae—and therefore the severity and cumulative disability per case of a condition—can be different by age, sex, location, and year, previous studies have found that disability weights do not substantially vary across locations, income, or levels of educational attainment.^19^, ^20^ In the GBD study, disability weights were based on population surveys with 60 890 respondents and held invariant between locations and over time.^20^ Additional details, including model specifications and data availability for each cause-specific model and development of disability weights by cause and their use in the estimation of non-fatal health loss, are available in the appendix of the GBD 2016 non-fatal publication.^8^

For non-fatal estimation, several methodological changes were made for GBD 2016. New data for the main causes of YLDs were identified through our collaboration with the Indian Council of Medical Research and the Public Health Foundation of India. For particular risk factors and diseases, the volume of available data increased substantially, such as child growth failure (stunting, wasting, or underweight), anaemia, congenital anomalies, schistosomiasis, intestinal helminths, and lymphatic filariasis. We have improved our analysis of total admissions per person by country, year, age, and sex, which facilitated incorporation of additional hospital data sources that were previously excluded because of incomplete knowledge of catchment population size. We extended our analyses of linked USA medical claims data to impute age-specific and sex-specific ratios for multiple admissions per illness episode, ICD code appearance in the non-primary position, and inpatient versus outpatient use.^8^ We applied each of the three ratios sequentially to non-linked hospital inpatient data from elsewhere that only had a single ICD code per visit to adjust prevalence and incidence data. We have incorporated more predictive covariates into our non-fatal disease models to better predict variation in disease levels rather than measurement error as the source of variation, and we improved our analysis of the MIRs for cancers, resulting in considerably higher ratios in lower SDI quintiles and thus substantially lower YLD estimates for cancer.

### Estimation of DALYs, HALE, and corresponding uncertainty

We calculated DALYs as the sum of YLLs and YLDs for each cause, location, age group, sex, and year.^8^, ^10^ The same estimates of YLDs per person for each location, age, sex, and year from 1990 to 2016 are used to establish HALE by age group within abridged multiple-decrement life tables with use of methods developed by Sullivan.^9^

For all results, we report 95% uncertainty intervals (UIs) derived from 1000 draws from the posterior distribution of each step in the estimation process. Unlike confidence intervals, UIs capture uncertainty from multiple modelling steps, as well as from sources such as model estimation and model specification, rather than from sampling error alone. Uncertainty associated with estimation of mortality and YLLs reflects sample sizes of data sources, adjustment and standardisation methods applied to data, parameter uncertainty in model estimation, and uncertainty within all-cause and cause-specific mortality models. For estimation of prevalence, incidence, and YLDs, UIs incorporated variability from sample sizes within data sources, adjustments to data to account for non-reference definitions, parameter uncertainty in model estimation, and uncertainty associated with establishment of disability weights. Because direct information about the correlation between uncertainty in YLLs and YLDs was scarce, we assumed that uncertainty in age-specific YLDs was independent of age-specific YLLs or death rates.

### Epidemiological transition and relationship between DALYs, HALE, and SDI

For GBD 2016, the composite indicator of SDI was again based on the geometric mean of three measures—LDI per person, average years of schooling among populations aged 15 years or older, and TFR—but the analysis was strengthened in three important ways.^10^ First, we substantially revised estimates of education, adding new data and improved methods for subnational locations. Second, instead of using estimates of TFR from the UN Population Division, we systematically reviewed, extracted, and analysed fertility data from all available locations to derive a time series of TFR for each national and subnational GBD location.^7^ Third, rather than rescaling SDI on the basis of the full range of observed values within the time series, we developed a fixed scale for GBD 2016; details on development of this fixed scale are available in the GBD 2016 mortality publication.^7^ We examined the average relationship between DALYs, HALE, and SDI using a Gaussian process regression model; we used these regressions to estimate expected values of these summary measures at each level of SDI. Additional detail on SDI calculation and location-specific SDI values are available in the appendix of the GBD 2016 mortality publication.^7^

### Data sharing

The statistical code used in the entire process is available through an online repository.

### Role of the funding source

The funder of the study had no role in study design, data collection, data analysis, data interpretation, or writing of the report. The corresponding author had full access to all the data in the study and had final responsibility for the decision to submit for publication.

## Results

### Global levels of and trends for DALYs and HALE

The total number of all-age DALYs in 2016 was 2·39 billion (95% UI 2·18 billion to 2·63 billion). Total all-age DALY counts for CMNN causes fell by 40·1% (37·4–42·7) from 1·11 billion (1·07 billion to 1·16 billion) in 1990 to 668 million (632 million to 708 million) in 2016, whereas total all-age DALY counts from NCDs increased by 36·6% from 1·07 billion (958 million to 1·20 billion) in 1990 to 1·47 billion (1·30 billion to 1·66 billion) in 2016 (table 1). Total DALYs from injuries decreased by 1·6% (–3·8 to 6·2) from 260 million (243 million to 277 million) in 1990 to 255 million (236 million to 281 million) in 2016. Age groups older than 80 years had 149 million (139 million to 159 million) all-age DALYs in 2016 compared with 75·1 million (71·1 million to 79·5 million) in 1990, with increases across all SDI quintiles. Of these, 87·8% were due to NCDs in 2016 compared with 86·8% in 1990.

**Table 1:** Global all-age DALYs and age-standardised DALY rates in 1990, 2006, and 2016 with mean percentage changes between 1990 and 2016, 2006 and 2016, and 1990 and 2016 for all causes

				**All-age DALYs (thousands)**					**Age-standardised DALY rate (per 100 000)**				
				1990	2006	2016	Percentage change, 1990–2016	Percentage change, 2006–16	1990	2006	2016	Percentage change, 1990–2016	Percentage change, 2006–16
**All causes**				**2 448 430·5 (2 305 218·2 to 2 608 339·5)**	**2 490 698·9 (2 308 527·1 to 2 689 861·1)**	**2 391 258·0 (2 184 254·1 to 2 631 699·0)**	**–2·3 (−5·9 to 0·9)**	–4·0 (−6·0 to −2·1)*	**48 407·8 (45 385·4 to 51 762·0)**	**40 485·1 (37 556·0 to 43 679·3)**	**33 641·0 (30 808·7 to 36 924·3)**	–30·5 (−32·6 to −28·6)*	–16·9 (−18·6 to −15·3)*
**Communicable, maternal, neonatal, and nutritional diseases**				**1 114 176·6 (1 073 948·8 to 1 156 050·2)**	**918 804·8 (885 242·1 to 959 452·3)**	**667 823·7 (632 212·4 to 708 405·1)**	–40·1 (−42·7 to −37·4)*	–27·3 (−29·8 to −24·9)*	**18 071·6 (17 386·0 to 18 790·5)**	**13 801·1 (13 286·8 to 14 406·5)**	**9396·8 (8894·5 to 9956·2)**	–48·0 (−50·1 to −45·8)*	–31·9 (−34·2 to −29·7)*
	**HIV/AIDS and tuberculosis**			**84 184·5 (79 728·6 to 89 558·0)**	**159 063·9 (152 851·5 to 165 139·0)**	**101 133·3 (97 487·1 to 105 092·9)**	20·1 (13·0 to 26·7)*	–36·4 (−37·9 to −34·7)*	**1788·1 (1690·1 to 1918·3)**	**2439·5 (2345·8 to 2531·5)**	**1355·2 (1306·2 to 1407·5)**	–24·2 (−29·7 to −20·1)*	–44·5 (−45·7 to −43·0)*
		Tuberculosis		68 029·7 (64 153·4 to 73 066·3)	56 881·5 (54 312·6 to 59 442·6)	43 557·9 (41 529·0 to 45 716·5)	–36·0 (−41·3 to −32·4)*	–23·4 (−26·1 to −20·6)*	1480·1 (1390·4 to 1613·6)	916·5 (874·7 to 956·7)	593·1 (565·5 to 621·7)	–59·9 (−63·7 to −57·7)*	–35·3 (−37·6 to −32·9)*
			Drug-susceptible tuberculosis	67 560·5 (63 730·5 to 72 611·6)	51 760·2 (49 194·0 to 54 289·2)	39 869·8 (38 054·8 to 41 916·2)	–41·0 (−45·6 to −37·5)*	–23·0 (−25·7 to −20·0)*	1469·5 (1380·7 to 1603·6)	834·1 (791·6 to 873·6)	543·0 (517·8 to 571·1)	–63·0 (−66·4 to −60·8)*	–34·9 (−37·2 to −32·5)*
			Multidrug-resistant tuberculosis without extensive drug resistance	469·2 (378·3 to 578·8)	4886·9 (4122·2 to 5829·2)	3319·4 (2787·6 to 3910·3)	607·5 (511·8 to 717·0)*	–32·1 (−38·5 to −24·8)*	10·6 (8·5 to 13·1)	78·7 (66·3 to 93·9)	45·1 (37·9 to 53·2)	327·6 (267·7 to 394·1)*	–42·7 (−48·1 to −36·5)*
			Extensively drug-resistant tuberculosis	··	234·5 (194·6 to 279·1)	368·8 (301·1 to 444·5)	··	57·3 (36·1 to 82·1)*	··	3·8 (3·1 to 4·5)	5·0 (4·1 to 6·0)	··	32·5 (14·8 to 53·2)*
			Latent tuberculosis infection	··	··	··	··	··	··	··	··	··	··
			HIV/AIDS	16 154·8 (14 497·1 to 18 106·5)	102 182·3 (96 751·1 to 107 544·2)	57 575·4 (54 618·5 to 60 967·9)	256·4 (220·1 to 293·5)*	–43·6 (−45·4 to −41·6)*	308·1 (276·2 to 345·6)	1522·9 (1443·6 to 1601·3)	762·1 (723·6 to 806·2)	147·4 (121·5 to 173·2)*	–50·0 (−51·5 to −48·2)*
			Drug-susceptible HIV/AIDS-Tuberculosis	4668·5 (3624·4 to 5760·2)	24 070·5 (16 708·0 to 31 379·1)	11 724·0 (8154·4 to 15 522·4)	151·1 (116·8 to 191·3)*	–51·3 (−54·0 to −48·6)*	88·2 (68·0 to 109·1)	359·7 (249·9 to 468·7)	155·5 (108·2 to 205·9)	76·2 (52·6 to 103·8)*	–56·8 (−59·2 to −54·4)*
			Multidrug-resistant HIV/AIDS-Tuberculosis without extensive drug resistance	25·9 (16·0 to 40·6)	2051·8 (1282·8 to 3070·2)	979·2 (597·7 to 1481·6)	3673·4 (2732·2 to 4952·8)*	–52·3 (−61·7 to −41·2)*	0·5 (0·3 to 0·8)	30·7 (19·2 to 45·8)	13·0 (7·9 to 19·7)	2486·4 (1853·0 to 3358·6)*	–57·6 (−66·1 to −47·8)*
			Extensively drug-resistant HIV/AIDS-Tuberculosis	··	39·9 (24·8 to 61·1)	57·3 (34·5 to 89·4)	··	43·5 (25·5 to 65·4)*	··	0·6 (0·4 to 0·9)	0·8 (0·5 to 1·2)	··	26·8 (10·8 to 46·4)*
			HIV/AIDS resulting in other diseases	11 460·3 (9938·9 to 13 435·6)	76 020·0 (67 021·8 to 86 026·2)	44 814·9 (39 932·9 to 50 112·4)	291·1 (245·9 to 337·5)*	–41·0 (−43·6 to −38·2)*	219·3 (189·8 to 257·4)	1131·9 (999·9 to 1280·9)	592·9 (528·6 to 663·0)	170·3 (138·2 to 203·1)*	–47·6 (−49·9 to −45·1)*
**Diarrhoea, lower respiratory, and other common infectious diseases**				**557 388·0 (522 551·7 to 600 325·4)**	**337 062·8 (317 957·5 to 359 176·2)**	**229** **961·4 (213** **682·3 to 247** **975·2)**	–58·7 (−61·9 to −55·2)*	–31·8 (−35·3 to −27·8)*	**8951·2 (8378·0 to 9601·7)**	**5152·7 (4858·8 to 5514·9)**	**3275·6 (3051·7 to 3531·8)**	–63·4 (−66·0 to −60·7)*	–36·4 (−39·7 to −33·0)*
	Diarrhoeal diseases			175 168·6 (150 592·6 to 201 351·3)	113 944·8 (99 183·9 to 135 659·8)	74 414·6 (63 402·0 to 93 414·9)	–57·5 (−62·8 to −50·1)*	–34·7 (−41·0 to −28·1)*	2914·2 (2482·9 to 3469·2)	1768·5 (1526·0 to 2136·7)	1063·1 (907·5 to 1332·3)	–63·5 (−67·6 to −58·3)*	–39·9 (−45·2 to −34·3)*
	Intestinal infectious diseases			15 662·6 (8797·4 to 25 360·4)	12 822·7 (7207·6 to 20 879·4)	10 601·7 (6041·1 to 17 309·3)	–32·3 (−43·5 to −21·8)*	–17·3 (−25·1 to −10·8)*	249·7 (140·6 to 404·8)	184·6 (103·8 to 300·4)	144·3 (82·3 to 235·2)	–42·2 (−51·5 to −33·7)*	–21·9 (−29·4 to −15·6)*
		Typhoid fever		13 362·8 (7235·9 to 22 248·3)	10 793·8 (5876·4 to 17 717·0)	8843·0 (4901·5 to 14 436·1)	–33·8 (−44·0 to −23·6)*	–18·1 (−25·7 to −12·1)*	212·5 (115·0 to 353·9)	155·3 (84·8 to 254·0)	120·4 (66·6 to 196·8)	–43·3 (−51·9 to −35·1)*	–22·4 (−29·7 to −16·6)*
		Paratyphoid fever		1867·3 (850·2 to 3711·1)	1773·6 (826·8 to 3439·6)	1607·0 (759·0 to 3109·8)	–13·9 (−27·1 to −1·5)*	–9·4 (−17·9 to −1·8)*	30·6 (13·9 to 60·4)	25·6 (11·9 to 49·6)	21·7 (10·2 to 42·0)	–29·0 (−39·4 to −19·2)*	–15·1 (−22·9 to −8·0)*
		Other intestinal infectious diseases		432·5 (107·2 to 1290·8)	255·4 (58·5 to 753·4)	151·7 (42·1 to 412·1)	–64·9 (−90·6 to 42·1)	–40·6 (−84·7 to 126·3)	6·7 (1·7 to 19·5)	3·8 (0·9 to 11·2)	2·2 (0·6 to 5·9)	–67·5 (−91·3 to 31·5)	–42·9 (−85·2 to 114·5)
	Lower respiratory infections			202 365·5 (182 794·4 to 220 607·6)	131 015·4 (121 489·8 to 139 228·6)	91 844·6 (84 674·4 to 98 252·6)	–54·6 (−58·7 to −49·4)*	–29·9 (−34·4 to −25·3)*	3237·1 (2942·1 to 3515·4)	2022·6 (1879·0 to 2147·6)	1326·7 (1221·8 to 1419·7)	–59·0 (−62·5 to −54·6)*	–34·4 (−38·6 to −30·2)*
	Upper respiratory infections			4868·6 (3012·8 to 7444·9)	5551·2 (3380·7 to 8488·2)	5991·2 (3621·1 to 9193·8)	23·1 (19·0 to 26·3)*	7·9 (6·2 to 9·4)*	88·2 (54·9 to 134·0)	83·0 (50·8 to 126·9)	81·0 (49·0 to 124·0)	–8·3 (−10·5 to −6·9)*	–2·5 (−3·8 to −1·5)*
	Otitis media			3111·7 (2057·2 to 4485·0)	3171·4 (2005·0 to 4675·3)	3187·5 (1993·2 to 4716·5)	2·4 (−3·8 to 7·0)	0·5 (−2·1 to 3·0)	53·4 (35·3 to 76·9)	46·7 (29·6 to 68·9)	43·3 (27·1 to 64·2)	–18·9 (−23·7 to −15·1)*	–7·3 (−9·8 to −4·9)*
	Meningitis			30 239·3 (23 939·3 to 34 552·6)	24 957·4 (21 655·0 to 28 764·2)	21 865·9 (18 204·6 to 28 280·5)	–27·7 (−41·7 to 3·1)	–12·4 (−23·9 to 7·8)	481·8 (385·6 to 549·0)	369·9 (321·3 to 426·1)	306·1 (254·0 to 398·0)	–36·5 (−48·5 to −9·7)*	–17·2 (−28·1 to 2·1)
		Pneumococcal meningitis		2187·5 (1808·2 to 2576·0)	1940·0 (1649·9 to 2287·4)	1902·8 (1569·5 to 2382·2)	–13·0 (−26·3 to 10·0)	–1·9 (−11·5 to 12·0)	37·2 (31·0 to 43·4)	29·1 (24·8 to 34·2)	26·2 (21·6 to 32·7)	–29·6 (−39·6 to −11·3)*	–9·9 (−18·7 to 3·2)
		*Haemophilus influenzae* type B meningitis		3330·3 (2606·2 to 3982·2)	2725·7 (2276·3 to 3165·4)	2426·0 (1967·2 to 3212·2)	–27·1 (−41·5 to 3·7)	–11·0 (−23·9 to 9·3)	52·4 (41·5 to 62·0)	40·3 (33·7 to 46·8)	34·1 (27·6 to 45·1)	–34·8 (−47·4 to −7·4)*	–15·3 (−27·6 to 3·9)
		Meningococcal meningitis		14 191·0 (11 094·1 to 16 492·1)	11 548·6 (9913·5 to 13 418·2)	8327·1 (6806·4 to 10 911·9)	–41·3 (−53·1 to −16·3)*	–27·9 (−37·7 to −12·2)*	224·2 (177·0 to 259·3)	170·8 (146·7 to 199·1)	116·6 (95·2 to 152·9)	–48·0 (−58·4 to −26·0)*	–31·7 (−41·1 to −16·8)*
		Other meningitis		10 530·5 (8030·6 to 12 434·2)	8743·1 (7350·6 to 10 148·8)	9210·0 (7559·7 to 12 250·5)	–12·5 (−30·3 to 28·9)	5·3 (−9·4 to 33·0)	168·1 (129·1 to 197·5)	129·7 (109·2 to 150·4)	129·2 (105·5 to 173·3)	–23·1 (−38·2 to 12·4)	–0·4 (−14·4 to 26·1)
	Encephalitis			7918·4 (5206·9 to 10 751·2)	7380·9 (6422·5 to 9033·9)	6704·1 (5469·3 to 8574·2)	–15·3 (−44·0 to 40·6)	–9·2 (−24·4 to 10·8)	135·5 (91·8 to 180·2)	111·5 (97·1 to 136·5)	92·7 (75·7 to 118·4)	–31·6 (−53·6 to 10·0)	–16·9 (−30·8 to 1·2)
	Diphtheria			842·7 (611·3 to 1167·2)	263·8 (183·1 to 374·7)	86·9 (62·5 to 123·4)	–89·7 (−93·2 to −84·0)*	–67·0 (−78·6 to −47·7)*	12·9 (9·4 to 17·9)	3·9 (2·7 to 5·6)	1·2 (0·9 to 1·8)	–90·5 (−93·7 to −85·3)*	–68·6 (−79·8 to −49·7)*
	Whooping cough			14 651·2 (6598·0 to 28 290·2)	9778·0 (4727·9 to 17 764·8)	6249·9 (3360·7 to 10 754·7)	–57·3 (−77·1 to −19·2)*	–36·1 (−63·1 to 17·1)	219·5 (98·9 to 424·0)	144·6 (69·9 to 262·6)	89·4 (48·1 to 153·9)	–59·3 (−78·1 to −22·8)*	–38·1 (−64·3 to 13·3)
	Tetanus			24 893·6 (14 235·3 to 33 445·8)	6340·9 (3695·4 to 7940·5)	2366·6 (1446·0 to 3062·9)	–90·5 (−92·7 to −87·7)*	–62·7 (−68·8 to −55·4)*	385·3 (222·8 to 516·6)	93·3 (54·4 to 116·8)	33·6 (20·3 to 43·4)	–91·3 (−93·2 to −88·9)*	–64·0 (−70·0 to −57·1)*
	Measles			76 350·8 (31 267·6 to 147 358·9)	20 794·3 (8237·6 to 43 871·3)	5724·8 (2148·6 to 12 257·6)	–92·5 (−94·4 to −90·5)*	–72·5 (−76·9 to −67·7)*	1150·7 (471·2 to 2220·4)	307·9 (122·0 to 649·2)	81·3 (30·5 to 174·2)	–92·9 (−94·7 to −91·0)*	–73·6 (−77·8 to −69·0)*
	Varicella and herpes zoster			1314·9 (1138·7 to 1509·6)	1042·2 (909·6 to 1205·0)	923·5 (779·5 to 1098·7)	–29·8 (−40·3 to −18·8)*	–11·4 (−20·6 to −2·8)*	22·8 (19·8 to 25·9)	16·2 (14·1 to 18·9)	13·0 (11·0 to 15·4)	–42·8 (−50·3 to −35·4)*	–19·7 (−27·6 to −12·2)*
**Neglected tropical diseases and malaria**				**87 294·8 (71 756·4 to 103 455·7)**	**99 229·2 (85 820·3 to 113 978·1)**	**74 995·1 (63 114·8 to 86 650·7)**	**–14·1 (−31·0 to 6·2)**	–24·4 (−37·6 to −8·6)*	**1423·8 (1183·4 to 1676·5)**	**1478·5 (1280·4 to 1696·3)**	**1050·5 (882·7 to 1217·9)**	–26·2 (−40·4 to −9·3)*	–28·9 (−41·5 to −14·0)*
	Malaria			60 389·3 (46 548·2 to 74 912·5)	77 253·7 (64 810·3 to 91 256·8)	56 201·2 (45 785·6 to 67 880·8)	–6·9 (−30·5 to 26·5)	–27·2 (−43·3 to −6·7)*	931·0 (722·1 to 1150·5)	1147·0 (963·0 to 1354·6)	794·7 (646·5 to 962·2)	–14·6 (−36·1 to 15·2)	–30·7 (−46·2 to −11·1)*
	Chagas disease			309·8 (286·3 to 334·8)	226·1 (204·9 to 251·3)	219·0 (194·6 to 250·7)	–29·3 (−34·4 to −23·7)*	–3·1 (−8·2 to 2·7)	7·7 (7·1 to 8·3)	4·0 (3·7 to 4·5)	3·1 (2·8 to 3·6)	–59·3 (−62·2 to −56·0)*	–22·6 (−26·7 to −17·8)*
	Leishmaniasis			2531·5 (1470·2 to 4203·0)	1897·2 (1151·9 to 3064·4)	981·0 (658·3 to 1480·6)	–61·2 (−67·2 to −52·0)*	–48·3 (−54·0 to −39·6)*	45·7 (27·0 to 75·4)	28·4 (17·2 to 45·7)	13·4 (9·0 to 20·3)	–70·6 (−74·9 to −64·1)*	–52·6 (−57·7 to −44·9)*
		Visceral leishmaniasis		2406·1 (1350·5 to 4080·8)	1684·8 (943·7 to 2878·0)	707·9 (400·1 to 1206·2)	–70·6 (−74·4 to −66·4)*	–58·0 (−61·9 to −53·9)*	43·1 (24·4 to 72·6)	25·1 (14·1 to 42·9)	9·8 (5·5 to 16·6)	–77·4 (−79·9 to −74·6)*	–61·1 (−64·7 to −57·2)*
		Cutaneous and mucocutaneous leishmaniasis		125·3 (67·7 to 217·2)	212·4 (131·6 to 329·4)	273·1 (177·2 to 398·9)	117·9 (67·5 to 215·8)*	28·6 (16·8 to 42·9)*	2·6 (1·4 to 4·4)	3·3 (2·0 to 5·0)	3·7 (2·4 to 5·4)	43·5 (11·8 to 103·5)*	12·5 (1·7 to 26·1)*
	African trypanosomiasis			1046·8 (559·1 to 1711·5)	539·0 (288·0 to 876·8)	128·4 (64·7 to 215·0)	–87·7 (−91·3 to −82·2)*	–76·2 (−83·2 to −65·9)*	19·2 (10·3 to 31·6)	7·8 (4·2 to 12·8)	1·7 (0·9 to 2·9)	–91·1 (−93·6 to −87·2)*	–78·2 (−84·6 to −68·8)*
	Schistosomiasis			2096·8 (1340·2 to 3410·1)	2464·8 (1447·7 to 4194·7)	1863·6 (1122·0 to 3175·2)	–11·1 (−17·5 to −6·7)*	–24·4 (−26·2 to −21·9)*	42·8 (27·5 to 69·2)	37·5 (22·2 to 63·5)	24·9 (15·0 to 42·3)	–41·9 (−46·3 to −38·9)*	–33·7 (−35·4 to −31·6)*
	Cysticercosis			489·0 (363·4 to 621·4)	500·8 (359·2 to 657·1)	468·1 (322·8 to 625·8)	–4·3 (−13·5 to 3·9)	–6·5 (−12·4 to −0·9)*	10·5 (7·8 to 13·4)	8·0 (5·7 to 10·5)	6·3 (4·4 to 8·4)	–40·0 (−45·2 to −35·0)*	–21·0 (−26·0 to −16·5)*
	Cystic echinococcosis			326·8 (237·4 to 449·1)	226·2 (161·3 to 313·8)	136·5 (95·3 to 193·7)	–58·2 (−68·4 to −44·3)*	–39·6 (−53·9 to −16·3)*	6·3 (4·5 to 8·7)	3·5 (2·5 to 4·8)	1·8 (1·3 to 2·6)	–70·6 (−77·7 to −61·1)*	–46·6 (−59·1 to −26·5)*
	Lymphatic filariasis			1595·7 (733·4 to 2983·5)	1897·7 (873·9 to 3542·8)	1189·0 (587·7 to 2114·9)	–25·5 (−41·4 to −9·3)*	–37·4 (−52·4 to −26·0)*	32·5 (14·9 to 60·7)	28·9 (13·3 to 54·0)	15·8 (7·8 to 28·1)	–51·5 (−61·8 to −41·2)*	–45·3 (−58·4 to −35·5)*
	Onchocerciasis			1420·4 (777·0 to 2254·9)	1266·4 (705·0 to 2003·3)	962·5 (452·3 to 1672·1)	–32·2 (−47·6 to −16·8)*	–24·0 (−41·6 to −6·5)*	28·4 (16·0 to 45·4)	19·1 (11·0 to 30·1)	12·9 (6·1 to 22·4)	–54·6 (−65·7 to −43·4)*	–32·6 (−48·5 to −17·5)*
	Trachoma			231·1 (156·9 to 324·3)	246·5 (166·2 to 348·4)	245·2 (162·4 to 353·6)	6·1 (−2·6 to 14·6)	–0·5 (−6·5 to 5·2)	6·7 (4·5 to 9·4)	4·9 (3·3 to 6·9)	3·7 (2·5 to 5·3)	–44·3 (−49·0 to −39·6)*	–23·8 (−28·5 to −19·3)*
	Dengue			822·8 (308·1 to 1364·0)	1798·2 (789·6 to 2494·8)	2956·9 (1359·2 to 4146·9)	259·4 (104·2 to 683·3)*	64·4 (36·2 to 115·9)*	13·9 (5·2 to 23·2)	26·7 (11·7 to 37·1)	40·2 (18·6 to 56·3)	189·0 (65·5 to 523·4)*	50·5 (24·7 to 97·7)*
	Yellow fever			784·5 (170·6 to 2314·6)	424·5 (89·8 to 1247·2)	374·0 (80·8 to 1075·1)	–52·3 (−61·3 to −41·2)*	–11·9 (−26·9 to 7·4)	13·2 (2·9 to 39·0)	6·1 (1·3 to 18·0)	5·0 (1·1 to 14·5)	–61·8 (−68·5 to −52·9)*	–17·1 (−31·3 to 1·3)
	Rabies			2979·4 (1867·4 to 4076·4)	1451·6 (867·1 to 1868·0)	744·2 (383·8 to 1106·3)	–75·0 (−82·7 to −61·4)*	–48·7 (−58·9 to −34·1)*	51·3 (32·6 to 71·3)	21·5 (12·9 to 27·5)	10·1 (5·2 to 15·1)	–80·3 (−86·5 to −69·9)*	–52·9 (−62·4 to −39·5)*
	Intestinal nematode infections			7460·9 (4726·6 to 11 584·0)	4083·3 (2617·1 to 6154·1)	3331·2 (2076·2 to 5158·6)	–55·4 (−57·9 to −52·6)*	–18·4 (−22·9 to −14·3)*	132·8 (83·2 to 206·9)	60·8 (39·0 to 91·6)	45·0 (28·2 to 69·6)	–66·1 (−68·0 to −64·0)*	–25·9 (−30·0 to −22·2)*
		Ascariasis		4634·7 (2996·9 to 7119·9)	1902·0 (1325·4 to 2758·9)	1308·8 (883·2 to 1942·4)	–71·8 (−74·6 to −68·4)*	–31·2 (−37·6 to −24·8)*	80·3 (51·4 to 124·0)	28·3 (19·8 to 41·1)	17·9 (12·1 to 26·4)	–77·8 (−79·9 to −74·9)*	–37·0 (−42·8 to −31·0)*
		Trichuriasis		671·4 (364·9 to 1142·9)	421·5 (233·0 to 717·2)	337·0 (186·2 to 573·6)	–49·8 (−55·2 to −44·3)*	–20·0 (−27·9 to −11·6)*	12·5 (6·8 to 21·2)	6·3 (3·5 to 10·7)	4·5 (2·5 to 7·7)	–63·7 (−67·8 to −59·7)*	–27·8 (−34·9 to −20·2)*
		Hookworm disease		2154·8 (1278·7 to 3371·1)	1759·8 (1058·9 to 2739·1)	1685·4 (1001·5 to 2648·9)	–21·8 (−26·7 to −16·6)*	–4·2 (−9·5 to 1·3)	40·0 (23·7 to 62·8)	26·2 (15·7 to 40·7)	22·6 (13·5 to 35·5)	–43·4 (−47·2 to −39·6)*	–13·5 (−18·2 to −8·5)*
	Food-borne trematodiases			1425·0 (591·7 to 2937·7)	1659·6 (832·5 to 3083·5)	1771·2 (923·9 to 3158·4)	24·3 (−3·9 to 71·7)	6·7 (1·3 to 15·7)*	27·9 (12·0 to 56·7)	25·4 (12·9 to 46·8)	23·7 (12·2 to 42·0)	–15·3 (−34·0 to 13·1)	–7·0 (−11·4 to 0·5)
	Leprosy			23·0 (15·5 to 32·3)	31·3 (21·3 to 44·0)	31·6 (21·4 to 44·0)	37·5 (34·0 to 40·9)*	1·1 (−1·3 to 3·6)	0·6 (0·4 to 0·8)	0·5 (0·4 to 0·8)	0·4 (0·3 to 0·6)	–20·7 (−22·7 to −18·8)*	–18·1 (−20·0 to −16·2)*
	Ebola virus disease			··	··	0·3 (0·2 to 1·1)	··	··	··	··	··	··	··
	Zika virus disease			··	··	5·1 (3·4–8·0)	··	··	··	··	0·1 (0·0–0·1)	··	··
	Guinea worm disease			50·7 (35·3 to 69·2)	0·2 (0·1 to 0·3)	··	–100·0 (−100·0 to −100·0)*	–99·5 (−99·7 to −99·4)*	1·1 (0·7 to 1·4)	··	··	–100·0 (−100·0 to −100·0)*	–99·6 (−99·7 to −99·5)*
	Other neglected tropical diseases			3311·2 (2409·7 to 4421·6)	3262·2 (2470·4 to 4130·1)	3386·0 (2569·6 to 4260·7)	2·3 (−22·0 to 31·6)	3·8 (−14·9 to 23·9)	52·2 (38·2 to 69·0)	48·3 (36·6 to 61·2)	47·4 (35·9 to 59·7)	–9·1 (−29·8 to 15·8)	–1·9 (−19·5 to 17·3)
**Maternal disorders**				**21 597·1 (20 063·6 to 22 834·1)**	**18 093·0 (16 785·8 to 19 171·8)**	**13 763·0 (12 668·6 to 15 064·0)**	–36·3 (−41·3 to −31·0)*	–23·9 (−29·3 to −17·4)*	**388·6 (361·4 to 411·5)**	**257·2 (238·8 to 272·5)**	**179·0 (164·7 to 195·8)**	–53·9 (−57·6 to −50·2)*	–30·4 (−35·4 to −24·4)*
	Maternal haemorrhage			6945·7 (5770·8 to 8257·7)	5416·4 (4627·6 to 6301·7)	4078·3 (3311·7 to 5035·2)	–41·3 (−47·4 to −34·9)*	–24·7 (−32·5 to −16·0)*	124·4 (103·1 to 148·1)	77·0 (65·8 to 89·7)	53·0 (43·1 to 65·4)	–57·4 (−61·7 to −53·0)*	–31·2 (−38·2 to −23·3)*
	Maternal sepsis and other maternal infections			2102·5 (1614·0 to 2721·4)	1562·3 (1223·7 to 1984·3)	1139·4 (833·1 to 1525·2)	–45·8 (−51·9 to −39·7)*	–27·1 (−35·7 to −18·1)*	37·6 (29·0 to 48·2)	22·1 (17·3 to 28·0)	14·8 (10·9 to 19·8)	–60·6 (−64·8 to −56·1)*	–33·0 (−40·9 to −24·8)*
	Maternal hypertensive disorders			2469·0 (1945·4 to 3125·5)	2478·1 (1983·1 to 3052·0)	1996·8 (1569·9 to 2483·1)	–19·1 (−26·0 to −11·2)*	–19·4 (−27·3 to −11·1)*	44·0 (34·8 to 55·2)	35·1 (28·2 to 43·2)	26·0 (20·5 to 32·3)	–40·9 (−45·8 to −35·4)*	–25·8 (−33·1 to −17·9)*
	Maternal obstructed labour and uterine rupture			1440·8 (1086·2 to 1865·8)	1240·4 (944·6 to 1582·8)	969·0 (716·6 to 1270·1)	–32·8 (−37·4 to −27·7)*	–21·9 (−27·4 to −15·9)*	27·0 (20·4 to 35·0)	17·9 (13·6 to 22·9)	12·6 (9·3 to 16·5)	–53·3 (−56·5 to −50·0)*	–29·5 (−34·5 to −24·1)*
	Maternal abortion, miscarriage, and ectopic pregnancy			1743·4 (1338·9 to 2235·5)	1469·0 (1150·0 to 1845·5)	1145·1 (855·9 to 1541·6)	–34·3 (−40·9 to −26·8)*	–22·1 (−30·6 to −12·6)*	31·9 (24·6 to 40·6)	20·9 (16·4 to 26·3)	14·9 (11·1 to 19·9)	–53·3 (−57·8 to −48·0)*	–28·8 (−36·6 to −20·4)*
	Indirect maternal deaths			2611·6 (1943·4 to 3385·8)	2577·2 (1949·1 to 3277·1)	1987·9 (1463·8 to 2619·8)	–23·9 (−30·7 to −16·4)*	–22·9 (−30·1 to −14·8)*	47·0 (35·2 to 60·4)	36·7 (27·7 to 46·6)	25·8 (19·1 to 34·0)	–45·0 (−49·7 to −39·9)*	–29·6 (−36·2 to −22·1)*
	Late maternal deaths			374·2 (244·4 to 552·4)	298·2 (178·6 to 480·8)	228·5 (134·6 to 370·9)	–39·0 (−50·1 to −28·8)*	–23·4 (−29·9 to −16·2)*	6·7 (4·4 to 9·8)	4·2 (2·5 to 6·8)	3·0 (1·8 to 4·8)	–55·8 (−63·7 to −49·2)*	–29·9 (−35·5 to −23·5)*
	Maternal deaths aggravated by HIV/AIDS			36·9 (21·5 to 52·5)	128·5 (81·8 to 169·0)	105·4 (66·7 to 142·9)	185·9 (142·7 to 243·7)*	–17·9 (−27·8 to −6·0)*	0·7 (0·4 to 0·9)	1·9 (1·2 to 2·4)	1·4 (0·9 to 1·9)	104·5 (73·6 to 146·1)*	–26·1 (−35·0 to −15·3)*
	Other maternal disorders			3872·9 (3015·3 to 4777·9)	2923·1 (2365·7 to 3539·5)	2112·5 (1628·9 to 2673·2)	–45·5 (−50·6 to −39·9)*	–27·7 (−34·7 to −19·7)*	69·3 (54·4 to 84·9)	41·5 (33·6 to 50·0)	27·5 (21·2 to 34·8)	–60·4 (−64·0 to −56·5)*	–33·7 (−40·1 to −26·4)*
**Neonatal disorders**				**261 357·2 (248 875·2 to 282 758·2)**	**211 984·8 (203 477·1 to 221 317·2)**	**163 569·7 (154 643·2 to 172 756·7)**	–37·4 (−42·7 to −32·7)*	–22·8 (−26·8 to −18·9)*	**3818·4 (3635·3 to 4130·2)**	**3073·5 (2949·7 to 3208·5)**	**2364·2 (2237·5 to 2493·4)**	–38·1 (−43·2 to −33·5)*	–23·1 (−27·0 to −19·2)*
	Neonatal preterm birth complications			112 767·2 (105 488·9 to 124 122·5)	81 159·7 (76 378·0 to 89 409·0)	62 031·6 (57 062·9 to 67 530·0)	–45·0 (−50·3 to −39·5)*	–23·6 (−29·9 to −17·4)*	1652·8 (1546·2 to 1819·4)	1176·6 (1107·3 to 1296·0)	892·7 (822·1 to 970·7)	–46·0 (−51·2 to −40·7)*	–24·1 (−30·4 to −18·1)*
	Neonatal encephalopathy due to birth asphyxia, and trauma			68 251·9 (61 749·3 to 76 860·5)	60 334·8 (55 822·8 to 65 138·9)	47 031·7 (41 794·2 to 51 919·1)	–31·1 (−41·3 to −20·8)*	–22·1 (−29·3 to −14·6)*	993·0 (897·9 to 1117·7)	874·2 (808·8 to 943·9)	682·2 (606·6 to 751·3)	–31·3 (−41·4 to −21·4)*	–22·0 (−29·1 to −14·6)*
	Neonatal sepsis and other neonatal infections			24 573·0 (18 972·5 to 31 156·6)	25 874·0 (21 266·6 to 32 360·3)	23 675·8 (20 056·0 to 30 684·5)	–3·6 (−22·7 to 20·9)	–8·5 (−19·1 to 4·0)	360·8 (278·4 to 460·0)	375·7 (308·9 to 469·6)	341·7 (291·0 to 444·5)	–5·3 (−23·7 to 18·6)	–9·0 (−19·5 to 3·2)
	Haemolytic disease and other neonatal jaundice			12 277·3 (10 225·3 to 15 113·1)	7996·0 (7149·5 to 9006·9)	4912·8 (4310·5 to 5605·8)	–60·0 (−69·1 to −50·4)*	–38·6 (−46·2 to −30·3)*	179·9 (149·9 to 221·4)	116·1 (103·8 to 130·8)	70·7 (62·0 to 80·7)	–60·7 (−69·6 to −51·4)*	–39·1 (−46·6 to −30·8)*
	Other neonatal disorders			43 487·8 (37 402·4 to 50 951·9)	36 620·3 (33 314·2 to 40 250·5)	25 917·7 (23 440·4 to 28 405·4)	–40·4 (−50·6 to −28·5)*	–29·2 (−36·1 to −21·0)*	631·9 (543·4 to 740·9)	530·9 (483·0 to 583·6)	376·8 (340·8 to 413·1)	–40·4 (−50·6 to −28·4)*	–29·0 (−35·9 to −20·7)*
**Nutritional deficiencies**				**69 823·3 (57 049·4 to 85 126·9)**	**64 648·9 (51 489·2 to 80 980·6)**	**60 936·1 (46 656·8 to 79 062·8)**	–12·7 (−23·8 to −1·2)*	**–5·7 (−12·3 to 0·1)**	**1167·8 (949·2 to 1434·1)**	**971·1 (775·1 to 1213·5)**	**844·3 (649·4 to 1090·3)**	–27·7 (−35·9 to −19·1)*	–13·1 (−18·9 to −7·7)*
	Protein-energy malnutrition			35 843·1 (29 589·9 to 41 569·5)	26 417·6 (23 896·5 to 29 406·0)	20 718·9 (18 009·6 to 24 194·8)	–42·2 (−51·8 to −26·7)*	–21·6 (−32·3 to −8·8)*	561·0 (467·3 to 647·0)	398·8 (361·7 to 443·4)	296·7 (258·1 to 346·4)	–47·1 (−55·8 to −33·4)*	–25·6 (−35·7 to −13·6)*
	Iodine deficiency			4355·1 (2983·7 to 6048·6)	3453·9 (2363·8 to 4790·9)	3240·6 (2213·3 to 4488·7)	–25·6 (−28·2 to −22·9)*	–6·2 (−8·7 to −3·5)*	84·5 (58·0 to 117·3)	52·4 (35·9 to 72·6)	43·5 (29·8 to 60·3)	–48·5 (−50·2 to −46·7)*	–16·9 (−19·2 to −14·5)*
	Vitamin A deficiency			188·5 (116·6 to 294·4)	225·3 (139·7 to 348·3)	252·4 (158·8 to 388·3)	33·9 (27·4 to 41·0)*	12·0 (8·7 to 15·5)*	3·2 (2·0 to 4·9)	3·4 (2·1 to 5·2)	3·4 (2·2 to 5·3)	7·0 (2·2 to 11·4)*	2·6 (−0·3 to 5·6)
	Iron-deficiency anaemia			27 097·5 (18 018·7 to 38 749·1)	32 422·9 (21 535·8 to 46 668·3)	34 841·8 (23 085·2 to 49 693·9)	28·6 (26·5 to 30·4)*	7·5 (6·2 to 8·9)*	475·7 (317·4 to 679·7)	482·7 (320·9 to 693·0)	474·1 (314·1 to 676·0)	–0·3 (−1·5 to 0·7)	–1·8 (−3·0 to −0·5)*
	Other nutritional deficiencies			2339·1 (1908·0 to 2766·9)	2129·2 (1791·2 to 2366·5)	1882·4 (1592·2 to 2158·7)	–19·5 (−32·9 to 5·7)	–11·6 (−20·4 to 0·7)	43·3 (36·7 to 50·2)	33·8 (28·4 to 37·4)	26·4 (22·4 to 30·3)	–38·9 (−48·1 to −24·3)*	–21·7 (−29·1 to −11·6)*
**Other communicable, maternal, neonatal, and nutritional diseases**				**32 531·7 (25 112·3 to 42 021·4)**	**28 722·2 (22 674·6 to 36 833·4)**	**23 465·1 (18 762·9 to 29 098·8)**	–27·9 (−36·2 to −18·6)*	–18·3 (−25·2 to −10·6)*	**533·7 (424·3 to 675·0)**	**428·7 (340·1 to 547·4)**	**328·0 (261·3 to 409·8)**	–38·5 (−44·8 to −31·3)*	–23·5 (−29·8 to −16·4)*
	Sexually transmitted diseases excluding HIV			16 447·6 (9960·1 to 25 590·4)	15 145·5 (9416·5 to 22 818·4)	12 016·0 (7764·9 to 17 118·7)	–26·9 (−37·6 to −13·9)*	–20·7 (−30·2 to −9·4)*	254·0 (157·8 to 389·9)	222·6 (138·7 to 335·0)	169·4 (108·1 to 242·8)	–33·3 (−42·1 to −22·6)*	–23·9 (−32·8 to −13·4)*
		Syphilis		14 613·8 (8275·0 to 23 755·0)	12 825·3 (7321·4 to 20 493·5)	9415·8 (5467·7 to 14 602·8)	–35·6 (−45·5 to −23·1)*	–26·6 (−35·8 to −14·9)*	217·9 (124·4 to 352·0)	188·3 (107·7 to 300·6)	135·5 (78·6 to 210·2)	–37·8 (−47·4 to −25·8)*	–28·1 (−37·1 to −16·8)*
		Chlamydial infection		425·2 (287·5 to 639·0)	519·6 (341·4 to 782·1)	562·4 (370·3 to 851·1)	32·3 (25·6 to 39·6)*	8·2 (5·9 to 10·4)*	8·0 (5·4 to 11·8)	7·5 (5·0 to 11·3)	7·3 (4·8 to 11·0)	–8·6 (−14·2 to −3·4)*	–3·3 (−5·6 to −1·4)*
		Gonococcal infection		465·0 (334·8 to 630·0)	582·3 (412·5 to 824·5)	675·2 (467·7 to 974·9)	45·2 (26·9 to 64·5)*	16·0 (10·3 to 21·8)*	9·2 (6·8 to 12·4)	8·6 (6·1 to 12·1)	8·8 (6·1 to 12·7)	–4·3 (−17·1 to 9·2)	2·7 (−2·7 to 7·8)
		Trichomoniasis		125·7 (48·3 to 265·4)	170·9 (65·2 to 362·0)	198·2 (75·9 to 420·8)	57·7 (55·1 to 60·5)*	15·9 (14·8 to 17·1)*	2·5 (0·9 to 5·2)	2·5 (1·0 to 5·4)	2·6 (1·0 to 5·5)	4·7 (3·6 to 5·8)*	1·8 (0·9 to 2·7)*
		Genital herpes		132·8 (43·0 to 302·2)	187·9 (61·0 to 428·1)	221·4 (71·2 to 507·1)	66·8 (61·5 to 70·0)*	17·8 (15·5 to 19·7)*	2·9 (0·9 to 6·6)	3·0 (1·0 to 6·8)	3·0 (1·0 to 6·8)	2·9 (1·3 to 4·8)*	–0·2 (−1·6 to 1·5)
		Other sexually transmitted diseases		685·1 (474·3 to 964·9)	859·5 (589·4 to 1221·9)	943·0 (643·7 to 1349·4)	37·6 (30·6 to 44·7)*	9·7 (7·4 to 12·2)*	13·5 (9·4 to 18·9)	12·6 (8·7 to 17·9)	12·3 (8·4 to 17·6)	–8·7 (−13·7 to −4·0)*	–2·6 (−4·8 to −0·4)*
		Hepatitis		9017·2 (8255·1 to 9723·9)	7718·6 (7259·2 to 8169·0)	5777·8 (5492·2 to 6078·9)	–35·9 (−41·5 to −30·1)*	–25·1 (−29·2 to −21·0)*	163·7 (151·1 to 175·3)	117·8 (111·0 to 124·6)	78·6 (74·8 to 82·7)	–52·0 (−55·9 to −48·3)*	–33·3 (−36·8 to −29·6)*
		Acute hepatitis A		1271·9 (1017·1 to 1540·7)	849·3 (677·0 to 1036·3)	450·7 (364·0 to 544·8)	–64·6 (−72·1 to −55·0)*	–46·9 (−58·9 to −32·0)*	19·5 (15·7 to 23·5)	12·6 (10·1 to 15·3)	6·4 (5·2 to 7·8)	–67·1 (−74·0 to −58·4)*	–49·0 (−60·6 to −34·6)*
		Hepatitis B		4656·5 (4209·1 to 5096·9)	4373·2 (4013·0 to 4741·4)	3823·8 (3543·7 to 4119·0)	–17·9 (−24·6 to −10·7)*	–12·6 (−17·9 to −7·0)*	94·2 (86·2 to 102·2)	68·7 (63·5 to 74·3)	51·6 (47·9 to 55·6)	–45·2 (−49·2 to −40·9)*	–24·9 (−29·2 to −20·4)*
		Hepatitis C		88·4 (72·0 to 107·8)	90·9 (73·8 to 112·2)	83·7 (66·3 to 104·2)	–5·3 (−17·1 to 7·5)	–7·9 (−17·6 to 3·0)	1·8 (1·5 to 2·3)	1·5 (1·2 to 1·9)	1·1 (0·9 to 1·4)	–37·1 (−44·2 to −29·1)*	–22·3 (−30·4 to −13·0)*
		Acute hepatitis E		3000·4 (2479·7 to 3449·1)	2405·2 (2090·1 to 2702·9)	1419·6 (1230·3 to 1610·1)	–52·7 (−60·4 to −44·5)*	–41·0 (−47·5 to −33·9)*	48·1 (40·0 to 55·0)	35·1 (30·5 to 39·4)	19·4 (16·9 to 22·0)	–59·6 (−65·9 to −52·9)*	–44·6 (−50·6 to −38·0)*
	Other infectious diseases			7066·9 (4328·0 to 9252·2)	5858·1 (4069·7 to 7316·8)	5671·3 (3883·9 to 7280·7)	–19·8 (−39·1 to 6·8)	–3·2 (−20·2 to 20·7)	116·1 (74·8 to 148·6)	88·2 (61·7 to 109·9)	80·0 (54·2 to 103·5)	–31·1 (−46·9 to −10·6)*	–9·4 (−25·1 to 13·1)
**Non-communicable diseases**				**1 074 539·5 (957 634·8 to 1 197 008·3)**	**1 312 102·0 (1 163 063·8 to 1 472 494·4)**	**1 468 000·0 (1 296 535·4 to 1 658 537·7)**	36·6 (32·9 to 39·9)*	11·9 (10·3 to 13·2)*	**25 460·8 (22 961·5 to 28 096·0)**	**22 707·2 (20 288·0 to 25 308·0)**	**20 786·9 (18 438·7 to 23 410·0)**	–18·4 (−20·6 to −16·4)*	–8·5 (−9·9 to −7·3)*
	**Neoplasms**			**151 550·6 (148 429·7 to 155 144·0)**	**189 094·4 (185 939·0 to 192 035·5)**	**213 221·0 (208 458·6 to 217 584·4)**	40·7 (36·8 to 44·9)*	12·8 (10·8 to 15·0)*	**3812·1 (3739·6 to 3895·3)**	**3377·2 (3321·1 to 3427·3)**	**3024·9 (2956·4 to 3086·9)**	–20·6 (−22·7 to −18·4)*	–10·4 (−12·0 to −8·7)*
		Lip and oral cavity cancer		2583·8 (2488·9 to 2719·1)	3668·0 (3546·8 to 3781·2)	4634·7 (4429·7 to 4823·4)	79·4 (64·8 to 88·9)*	26·4 (20·8 to 31·5)*	65·9 (63·5 to 69·2)	64·6 (62·5 to 66·6)	64·4 (61·6 to 67·0)	–2·2 (−10·0 to 2·9)	–0·2 (−4·5 to 3·8)
		Nasopharynx cancer		1590·5 (1506·1 to 1686·4)	1785·6 (1722·8 to 1842·5)	1906·0 (1809·3 to 2011·6)	19·8 (13·1 to 28·0)*	6·7 (0·2 to 13·1)*	37·7 (35·7 to 40·0)	30·1 (29·1 to 31·0)	26·0 (24·7 to 27·4)	–31·1 (−34·9 to −26·4)*	–13·5 (−18·7 to −8·5)*
		Other pharynx cancer		1794·9 (1683·0 to 1987·4)	2530·4 (2412·5 to 2620·7)	3209·6 (2949·9 to 3393·4)	78·8 (51·1 to 97·1)*	26·8 (15·8 to 34·9)*	45·5 (42·7 to 50·3)	44·5 (42·4 to 46·1)	44·4 (40·8 to 46·9)	–2·3 (−17·3 to 7·5)	–0·2 (−8·7 to 6·2)
		Oesophageal cancer		7809·6 (7554·6 to 8065·4)	9206·9 (9018·2 to 9394·6)	9276·1 (9019·5 to 9564·2)	18·8 (13·7 to 24·4)*	0·8 (−2·2 to 4·3)	207·4 (200·7 to 214·2)	170·0 (166·6 to 173·5)	132·7 (129·1 to 136·8)	–36·0 (−38·8 to −33·1)*	–22·0 (−24·2 to −19·3)*
		Stomach cancer		19 027·0 (18463·7 to 19 584·4)	19 048·8 (18 712·9 to 19 405·6)	18 345·5 (17 888·0 to 18 860·9)	–3·6 (−6·8 to 0·4)	–3·7 (−6·2 to −1·2)*	499·4 (485·0 to 513·6)	348·1 (342·0 to 354·6)	262·9 (256·2 to 270·1)	–47·4 (−49·1 to −45·3)*	–24·5 (−26·5 to −22·6)*
		Colon and rectum cancer		11 021·7 (10 741·7 to 11 482·7)	14 637·5 (14 356·4 to 14 918·1)	17 197·2 (16 480·2 to 17 851·9)	56·0 (44·5 to 63·5)*	17·5 (11·6 to 22·3)*	297·6290·6 to 309·1)	272·4 (267·2 to 277·4)	249·0 (238·6 to 258·4)	–16·4 (−22·3 to −12·5)*	–8·6 (−13·0 to −4·8)*
		Liver cancer		13 311·9 (12 450·1 to 14 010·2)	18 332·3 (17 719·9 to 18 813·2)	21 143·8 (20 268·1 to 21 985·9)	58·8 (49·2 to 70·4)*	15·3 (11·5 to 19·9)*	331·7 (311·0 to 348·5)	321·7 (310·9 to 329·8)	295·2 (283·0 to 306·7)	–11·0 (−16·4 to −4·6)*	–8·2 (−11·3 to −4·7)*
			Due to hepatitis B	6537·7 (5718·9 to 7253·4)	8838·7 (7823·1 to 9718·0)	9802·3 (8568·3 to 10 960·1)	49·9 (39·7 to 63·4)*	10·9 (6·3 to 16·3)*	158·5 (138·5 to 176·2)	151·2 (133·7 to 167·0)	134·7 (118·2 to 150·6)	–15·0 (−20·6 to −7·6)*	–10·9 (−14·4 to −6·6)*
			Due to hepatitis C	1850·2 (1639·8 to 2057·7)	2735·7 (2447·6 to 3011·7)	3310·5 (2930·7 to 3673·3)	78·9 (69·0 to 86·6)*	21·0 (16·9 to 24·9)*	49·6 (44·1 to 55·3)	51·2 (45·9 to 56·4)	47·9 (42·5 to 53·0)	–3·6 (−8·7 to 0·3)	–6·5 (−9·8 to −3·7)*
			Due to alcohol use	1686·3 (1384·8 to 2013·9)	2282·2 (1912·1 to 2710·9)	2925·7 (2463·2 to 3400·9)	73·5 (55·4 to 102·0)*	28·2 (20·4 to 38·5)*	45·0 (36·9 to 53·3)	42·1 (35·2 to 49·9)	41·8 (35·4 to 48·5)	–7·0 (−16·5 to 7·8)	–0·6 (−6·7 to 7·2)
			Due to other causes	3237·7 (2880·7 to 3688·9)	4475·8 (4004·8 to 5058·9)	5105·3 (4527·7 to 5757·7)	57·7 (46·9 to 68·8)*	14·1 (8·5 to 19·1)*	78·6 (70·0 to 89·7)	77·2 (69·0 to 87·0)	70·8 (62·9 to 79·7)	–9·9 (−15·9 to −3·8)*	–8·3 (−12·4 to −4·6)*
		Gallbladder and biliary tract cancer		2311·8 (2201·3 to 2555·0)	2885·3 (2655·7 to 3078·8)	3311·1 (3002·9 to 3530·9)	43·2 (26·0 to 53·5)*	14·8 (9·7 to 19·8)*	63·2 (60·3 to 69·5)	53·9 (49·7 to 57·4)	47·8 (43·5 to 50·9)	–24·3 (−33·2 to −19·0)*	–11·2 (−15·0 to −7·5)*
		Pancreatic cancer		4505·6 (4423·5 to 4576·2)	6492·4 (6404·8 to 6602·1)	8230·7 (8008·2 to 8445·8)	82·7 (78·3 to 87·1)*	26·8 (22·7 to 30·4)*	122·8 (120·5 to 124·6)	122·1 (120·5 to 124·1)	119·5 (116·2 to 122·6)	–2·6 (−5·0 to −0·3)*	–2·1 (−5·2 to 0·7)
		Larynx cancer		2290·5 (2207·2 to 2369·6)	2505·6 (2431·0 to 2569·8)	2749·8 (2661·2 to 2845·7)	20·1 (14·9 to 25·6)*	9·8 (6·0 to 13·6)*	59·6 (57·5 to 61·6)	45·3 (44·0 to 46·5)	38·7 (37·4 to 40·0)	–35·1 (−37·9 to −32·1)*	–14·6 (−17·5 to −11·7)*
		Tracheal, bronchus, and lung cancer		24 411·4 (23 862·3 to 25 086·9)	32 059·3 (31 518·1 to 32 631·0)	36 441·0 (35 401·2 to 37 462·8)	49·3 (43·0 to 54·5)*	13·7 (10·1 to 16·9)*	650·6 (636·2 to 667·8)	596·0 (586·5 to 606·2)	526·1 (511·2 to 540·8)	–19·1 (−22·5 to −16·4)*	–11·7 (−14·4 to −9·2)*
		Malignant skin melanoma		947·1 (853·4 to 1081·6)	1296·7 (1166·4 to 1434·0)	1550·5 (1378·4 to 1714·1)	63·7 (46·0 to 74·9)*	19·6 (13·6 to 25·4)*	23·6 (21·3 to 27·2)	22·8 (20·5 to 25·1)	21·8 (19·4 to 24·0)	–7·4 (−17·8 to −1·3)*	–4·3 (−9·0 to 0·3)
		Non-melanoma skin cancer		635·7 (611·9 to 658·6)	864·4 (841·4 to 887·8)	1022·6 (981·1 to 1068·7)	60·9 (54·6 to 70·1)*	18·3 (14·0 to 23·6)*	17·0 (16·4 to 17·7)	16·1 (15·7 to 16·6)	14·9 (14·3 to 15·6)	–12·3 (−15·6 to −7·5)*	–7·6 (−10·8 to −3·5)*
			Non-melanoma skin cancer (squamous-cell carcinoma)	635·2 (611·4 to 658·1)	863·5 (839·9 to 886·8)	1021·5 (980·2 to 1067·6)	60·8 (54·6 to 70·1)*	18·3 (14·0 to 23·6)*	17·0 (16·4 to 17·7)	16·1 (15·7 to 16·6)	14·9 (14·3 to 15·6)	–12·3 (−15·6 to −7·5)*	–7·6 (−10·8 to −3·5)*
			Non-melanoma skin cancer (basal-cell carcinoma)	0·5 (0·2 to 1·0)	0·9 (0·4 to 1·8)	1·1 (0·4 to 2·2)	122·9 (109·6 to 138·3)	23·8 (16·1 to 31·7)	··	··	··	15·8 (10·3 to 21·7)	-5·6 (-11·5 to 0·4)
		Breast cancer		9804·9 (9186·6 to 10 790·8)	13 209·0 (12 668·7 to 13 875·4)	15 107·8 (14 264·6 to 16 175·8)	54·1 (34·8 to 70·0)*	14·4 (6·3 to 22·2)*	248·6 (233·7 to 272·3)	229·4 (220·2 to 240·7)	208·5 (196·9 to 223·1)	–16·2 (−26·2 to −8·2)*	–9·1 (−15·4 to −3·2)*
		Cervical cancer		6047·7 (5226·5 to 7655·0)	7040·2 (5878·2 to 7516·1)	7390·0 (6019·9 to 7868·6)	22·2 (2·0 to 41·2)*	5·0 (−1·3 to 13·2)	149·0 (129·1 to 188·6)	119·3 (99·8 to 127·4)	100·6 (82·0 to 107·1)	–32·5 (−43·5 to −22·0)*	–15·7 (−20·8 to −9·2)*
		Uterine cancer		1573·9 (1480·6 to 1651·3)	1955·1 (1868·8 to 2035·2)	2122·8 (2010·4 to 2229·7)	34·9 (26·3 to 47·1)*	8·6 (2·7 to 16·4)*	41·3 (39·0 to 43·3)	35·6 (34·0 to 37·0)	30·1 (28·5 to 31·7)	–27·1 (−31·6 to −20·6)*	–15·3 (−19·9 to −9·3)*
		Ovarian cancer		2661·5 (2531·2 to 2741·5)	3523·0 (3393·4 to 3660·7)	4258·1 (4035·8 to 4459·3)	60·0 (51·8 to 68·2)*	20·9 (13·9 to 27·0)*	68·3 (65·3 to 70·2)	62·5 (60·2 to 64·8)	59·3 (56·2 to 62·1)	–13·2 (−17·5 to −8·8)*	–5·0 (−10·4 to −0·2)*
		Prostate cancer		3226·7 (2808·5 to 3505·0)	4765·1 (4006·7 to 5107·8)	6073·7 (4992·7 to 6582·3)	88·2 (67·8 to 100·9)*	27·5 (20·3 to 33·4)*	97·5 (85·4 to 105·9)	97·2 (82·2 to 104·2)	93·9 (77·2 to 101·8)	–3·7 (−13·6 to 2·7)	–3·4 (−8·7 to 1·3)
		Testicular cancer		400·0 (380·0 to 423·9)	393·1 (378·2 to 412·0)	391·8 (372·4 to 412·0)	–2·0 (−8·2 to 3·7)	–0·3 (−4·4 to 4·0)	7·9 (7·5 to 8·3)	5·9 (5·7 to 6·2)	5·2 (4·9 to 5·4)	–34·3 (−38·5 to −30·5)*	–12·3 (−15·8 to −8·6)*
		Kidney cancer		1753·1 (1707·2 to 1806·3)	2476·1 (2418·4 to 2528·9)	3023·6 (2911·4 to 3135·9)	72·5 (64·5 to 80·9)*	22·1 (17·6 to 26·5)*	44·2 (43·2 to 45·4)	45·0 (44·0 to 46·0)	43·4 (41·8 to 45·0)	–1·9 (−6·1 to 2·2)	–3·7 (−7·2 to −0·2)*
		Bladder cancer		2235·8 (2157·3 to 2326·8)	2796·3 (2735·8 to 2859·6)	3315·2 (3193·2 to 3425·5)	48·3 (38·9 to 53·9)*	18·6 (13·9 to 22·4)*	63·3 (61·2 to 65·7)	54·3 (53·1 to 55·6)	49·5 (47·7 to 51·1)	–21·8 (−26·6 to −19·0)*	–9·0 (−12·4 to −6·1)*
		Brain and nervous system cancer		5620·2 (4833·4 to 5997·6)	6729·7 (6094·9 to 7049·7)	7660·0 (6922·8 to 8280·4)	36·3 (25·9 to 54·7)*	13·8 (9·4 to 20·8)*	116·8 (101·5 to 123·9)	109·1 (98·5 to 114·1)	105·0 (94·9 to 113·3)	–10·0 (−16·4 to 2·6)	–3·7 (−7·3 to 2·3)
		Thyroid cancer		768·4 (713·7 to 817·8)	960·7 (918·0 to 1004·4)	1122·8 (1071·3 to 1183·5)	46·1 (35·1 to 60·9)*	16·9 (11·0 to 23·8)*	19·1 (17·9 to 20·3)	16·9 (16·2 to 17·6)	15·8 (15·0 to 16·6)	–17·6 (−23·5 to −9·7)*	–6·8 (−11·4 to −1·3)*
		Mesothelioma		398·9 (361·5 to 440·9)	535·4 (509·7 to 569·4)	660·9 (619·4 to 700·6)	65·7 (43·6 to 81·4)*	23·4 (17·8 to 28·2)*	10·5 (9·5 to 11·7)	9·8 (9·3 to 10·3)	9·5 (8·9 to 10·1)	–9·3 (−21·7 to −0·8)*	–2·8 (−7·1 to 0·9)
		Hodgkin's lymphoma		1589·4 (1176·0 to 1775·3)	1269·1 (1048·1 to 1466·8)	1126·8 (943·7 to 1337·1)	–29·1 (−35·9 to −15·9)*	–11·2 (−14·5 to −8·0)*	31·8 (23·8 to 35·5)	19·7 (16·4 to 22·9)	15·2 (12·8 to 18·1)	–52·1 (−56·7 to −43·2)*	–22·7 (−25·4 to −20·1)*
		Non-Hodgkin lymphoma		4095·3 (3844·4 to 4377·0)	5528·6 (5174·2 to 5736·3)	6783·0 (6172·5 to 7083·3)	65·6 (49·2 to 74·7)*	22·7 (16·0 to 27·2)*	92·8 (88·3 to 98·0)	93·6 (88·1 to 96·9)	94·9 (86·6 to 99·1)	2·3 (−7·4 to 7·3)	1·4 (−4·1 to 5·0)
		Multiple myeloma		1141·8 (1032·1 to 1308·8)	1664·3 (1489·5 to 1831·8)	2114·0 (1901·5 to 2339·1)	85·2 (72·6 to 98·4)*	27·0 (22·6 to 32·6)*	30·7 (27·9 to 35·5)	31·0 (27·6 to 34·0)	30·5 (27·4 to 33·9)	–0·7 (−8·6 to 5·8)	–1·5 (−4·9 to 2·7)
		Leukaemia		10 455·3 (9415·6 to 12 242·4)	10 401·6 (9581·8 to 11 171·9)	10 204·0 (9319·4 to 10 809·1)	–2·4 (−14·2 to 7·2)	–1·9 (−6·2 to 2·3)	204·7 (186·8 to 234·4)	166·3 (153·6 to 178·1)	141·6 (129·3 to 149·9)	–30·9 (−38·4 to −25·0)*	–14·9 (−18·4 to −11·3)*
			Acute lymphoid leukaemia	2158·2 (1900·3 to 3001·6)	2339·9 (2178·5 to 2794·3)	2426·6 (2216·0 to 2688·8)	12·4 (−15·5 to 28·2)	3·7 (−7·8 to 9·9)	37·8 (33·5 to 51·5)	35·0 (32·7 to 41·7)	33·1 (30·2 to 36·6)	–12·5 (−33·3 to −1·5)*	–5·7 (−15·9 to −0·2)*
			Chronic lymphoid leukaemia	468·8 (434·3 to 553·7)	617·1 (580·4 to 709·0)	703·1 (654·0 to 805·0)	50·0 (38·9 to 61·4)*	13·9 (9·2 to 19·1)*	12·6 (11·7 to 14·6)	11·6 (10·9 to 13·3)	10·3 (9·7 to 11·8)	–17·6 (−22·8 to −12·2)*	–11·1 (−14·6 to −7·2)*
			Acute myeloid leukaemia	1707·6 (1577·2 to 2023·1)	2327·1 (2152·7 to 2540·6)	2651·6 (2444·2 to 2838·1)	55·3 (34·6 to 68·5)*	13·9 (8·7 to 18·2)*	35·4 (33·1 to 41·0)	38·0 (35·2 to 41·2)	36·9 (34·1 to 39·4)	4·3 (−8·5 to 12·2)	–2·9 (−7·0 to 0·6)
			Chronic myeloid leukaemia	706·2 (639·5 to 775·2)	654·3 (596·6 to 723·6)	609·6 (550·3 to 676·0)	–13·7 (−21·0 to −6·6)*	–6·8 (−11·1 to −2·2)*	16·6 (15·2 to 18·1)	11·0 (10·1 to 12·1)	8·4 (7·6 to 9·3)	–49·5 (−53·5 to −45·7)*	–23·5 (−26·9 to −20·1)*
			Other leukaemia	5414·4 (4487·0 to 6178·9)	4463·2 (3848·9 to 4762·2)	3813·1 (3326·8 to 4039·8)	–29·6 (−38·9 to −18·9)*	–14·6 (−19·6 to −9·0)*	102·3 (86·4 to 114·9)	70·6 (60·9 to 75·1)	52·9 (46·2 to 56·0)	–48·4 (−54·7 to −41·5)*	–25·1 (−29·4 to −20·4)*
		Other neoplasms		7536·4 (7307·3 to 7843·6)	10 533·8 (9864·9 to 10 811·8)	12 847·7 (11 679·5 to 13 268·0)	70·5 (52·9 to 78·0)*	22·0 (17·2 to 25·3)*	163·6 (158·9 to 170·2)	174·2 (163·3 to 178·5)	178·5 (162·3 to 184·3)	9·2 (−2·3 to 13·4)	2·5 (−1·5 to 5·2)
**Cardiovascular diseases**				**266 709·6 (258 611·1 to 274 986·6)**	**321 851·2 (312 966·3 to 330 487·6)**	**353 120·9 (341 794·5 to 364 965·3)**	32·4 (28·8 to 36·2)*	9·7 (7·4 to 12·2)*	**7267·3 (7049·6 to 7496·0)**	**6046·0 (5878·1 to 6208·3)**	**5178·4 (5010·6 to 5352·2)**	–28·7 (−30·7 to −26·8)*	–14·3 (−16·1 to −12·4)*
	Rheumatic heart disease			13 277·2 (11 854·4 to 14 558·1)	11 235·5 (10 229·5 to 12 162·0)	9803·1 (9154·5 to 10 556·6)	–26·2 (−32·8 to −18·5)*	–12·8 (−18·3 to −6·9)*	296·4 (263·4 to 324·9)	185·4 (169·1 to 199·9)	135·9 (127·1 to 145·8)	–54·2 (−58·2 to −49·1)*	–26·7 (−31·3 to −21·7)*
	Ischaemic heart disease			118 996·9 (115 809·8 to 122 723·6)	153 547·6 (149 867·7 to 156 971·0)	174 611·1 (169 736·1 to 179 827·6)	46·7 (42·5 to 51·5)*	13·7 (10·7 to 16·7)*	3344·1 (3255·9 to 3448·7)	2908·3 (2840·4 to 2971·7)	2562·8 (2491·4 to 2640·8)	–23·4 (−25·6 to −21·0)*	–11·9 (−14·2 to −9·6)*
	Cerebrovascular disease			95 306·8 (91 616·6 to 100 606·2)	111 916·0 (108 069·6 to 115 501·2)	116 445·1 (111 385·4 to 121 406·9)	22·2 (16·7 to 27·0)*	4·0 (1·6 to 6·5)*	2600·1 (2496·3 to 2744·0)	2116·6 (2041·9 to 2186·6)	1711·2 (1635·3 to 1784·4)	–34·2 (−37·2 to −31·5)*	–19·1 (−21·0 to −17·2)*
		Ischaemic stroke		37 687·2 (35 505·5 to 39 624·6)	46 909·9 (44 168·6 to 49 455·6)	51 897·4 (47 896·6 to 55 567·7)	37·7 (32·2 to 43·4)*	10·6 (7·1 to 14·0)*	1112·1 (1049·6 to 1169·6)	934·8 (880·6 to 985·5)	787·5 (728·4 to 843·2)	–29·2 (−32·0 to −26·2)*	–15·8 (−18·4 to −13·1)*
		Haemorrhagic stroke		57 619·6 (55 206·4 to 61 574·1)	65 006·1 (63 154·6 to 66 941·1)	64 547·7 (62 622·3 to 66 497·6)	12·0 (6·3 to 17·0)*	–0·7 (−2·8 to 1·5)	1488·0 (1427·4 to 1594·8)	1181·8 (1148·5 to 1216·2)	923·6 (896·2 to 951·9)	–37·9 (−41·3 to −35·2)*	–21·8 (−23·5 to −20·1)*
	Hypertensive heart disease			12 136·6 (9824·4 to 13 763·9)	13 571·4 (11 603·8 to 15 049·4)	16 335·1 (13 456·6 to 17 843·7)	34·6 (24·7 to 52·5)*	20·4 (10·2 to 32·9)*	338·6 (274·9 to 382·5)	259·6 (221·8 to 287·4)	242·5 (199·7 to 265·0)	–28·4 (−33·4 to −19·1)*	–6·6 (−14·6 to 2·8)
	Cardiomyopathy and myocarditis			6713·9 (5820·8 to 7248·0)	8576·8 (7305·3 to 9168·4)	8718·2 (7574·9 to 9628·0)	29·9 (16·7 to 44·8)*	1·6 (−7·1 to 12·9)	159·0 (133·6 to 171·9)	145·7 (123·9 to 155·8)	122·7 (106·4 to 135·1)	–22·9 (−30·0 to −13·5)*	–15·8 (−22·8 to −6·8)*
		Myocarditis		1253·7 (1032·8 to 1618·8)	1422·2 (1132·2 to 1574·2)	1367·5 (1118·2 to 1513·1)	9·1 (−20·2 to 28·8)	–3·9 (−12·7 to 5·6)	24·8 (20·8 to 30·4)	23·0 (18·1 to 25·4)	19·1 (15·6 to 21·1)	–22·8 (−41·0 to −11·7)*	–16·8 (−24·3 to −8·0)*
		Alcoholic cardiomyopathy		1815·2 (1512·9 to 2177·5)	2878·5 (2414·3 to 3221·2)	2591·2 (2055·7 to 3240·6)	42·8 (11·8 to 80·0)*	–10·0 (−27·6 to 14·3)	44·5 (37·1 to 53·3)	47·8 (40·1 to 53·2)	35·2 (28·0 to 43·9)	–21·0 (−37·8 to −0·8)*	–26·3 (−40·4 to −7·6)*
		Other cardiomyopathy		3644·9 (2878·4 to 4234·4)	4276·1 (3615·3 to 4655·1)	4759·4 (4089·9 to 5094·4)	30·6 (14·5 to 52·0)*	11·3 (4·3 to 19·4)*	89·7 (68·9 to 103·1)	75·0 (63·1 to 81·5)	68·3 (58·5 to 73·2)	–23·8 (−32·3 to −9·1)*	–8·8 (−14·3 to −2·7)*
	Atrial fibrillation and flutter			3044·8 (2345·7 to 3888·0)	4460·7 (3484·6 to 5666·6)	5951·3 (4649·6 to 7516·9)	95·5 (91·1 to 100·0)*	33·4 (31·8 to 35·1)*	97·8 (76·0 to 123·5)	93·7 (73·6 to 118·1)	93·4 (73·2 to 118·1)	–4·5 (−6·5 to −2·4)*	–0·2 (−1·3 to 0·8)
	Aortic aneurysm			1942·5 (1855·5 to 2068·9)	2510·2 (2437·9 to 2611·8)	2881·8 (2800·8 to 2975·5)	48·4 (39·9 to 58·1)*	14·8 (10·7 to 20·1)*	54·4 (52·1 to 57·6)	47·8 (46·5 to 49·6)	42·6 (41·4 to 44·0)	–21·7 (−25·8 to −17·0)*	–10·9 (−13·9 to −7·1)*
	Peripheral artery disease			678·4 (486·1 to 948·0)	970·9 (719·7 to 1325·7)	1235·1 (922·2 to 1683·9)	82·1 (68·0 to 103·9)*	27·2 (20·9 to 35·7)*	21·4 (15·4 to 29·7)	20·3 (15·1 to 27·9)	19·3 (14·4 to 26·3)	–9·4 (−17·0 to 0·5)	–4·9 (−9·6 to 1·3)
	Endocarditis			1536·3 (1320·2 to 1751·3)	1998·3 (1748·2 to 2320·0)	2368·7 (2104·5 to 2797·1)	54·2 (39·0 to 69·5)*	18·5 (12·0 to 23·8)*	34·6 (29·7 to 39·2)	34·0 (29·6 to 39·2)	33·6 (29·8 to 39·7)	–3·0 (−11·4 to 5·3)	–1·3 (−6·7 to 2·9)
	Other cardiovascular and circulatory diseases			13 076·2 (10 976·0 to 14 815·8)	13 063·8 (11 561·4 to 15 422·4)	14 771·4 (13 010·9 to 17 397·0)	13·0 (3·2 to 31·9)*	13·1 (9·8 to 17·3)*	320·9 (269·6 to 364·9)	234·7 (208·1 to 276·1)	214·5 (189·4 to 251·8)	–33·2 (−38·7 to −22·3)*	–8·6 (−11·2 to −5·3)*
**Chronic respiratory diseases**				**86 833·7 (79 815·5 to 92 631·5)**	**86 665·1 (81 193·2 to 92 630·9)**	**92 528·7 (86 142·3 to 99 725·6)**	6·6 (2·0 to 15·4)*	6·8 (4·5 to 9·7)*	**2256·8 (2079·1 to 2386·5)**	**1601·5 (1507·8 to 1698·0)**	**1351·7 (1261·3 to 1452·3)**	–40·1 (−43·1 to −34·6)*	–15·6 (−17·6 to −13·2)*
	Chronic obstructive pulmonary disease			59 810·7 (53 271·2 to 64 128·3)	59 572·5 (56 816·4 to 62 571·2)	63 434·3 (60 586·3 to 66 676·3)	6·1 (0·6 to 18·4)*	6·5 (3·8 to 10·1)*	1666·8 (1486·0 to 1780·7)	1151·0 (1098·0 to 1205·8)	945·3 (904·1 to 994·8)	–43·3 (−46·2 to −36·8)*	–17·9 (−19·9 to −15·1)*
	Pneumoconiosis			568·0 (494·8 to 787·9)	560·3 (506·1 to 635·2)	577·1 (517·2 to 647·3)	1·6 (−25·7 to 16·9)	3·0 (−4·5 to 8·4)	15·0 (13·2 to 20·5)	10·4 (9·4 to 11·8)	8·4 (7·6 to 9·4)	–44·0 (−58·3 to −36·2)*	–19·1 (−24·6 to −15·0)*
		Silicosis		303·5 (251·4 to 461·0)	273·2 (247·2 to 310·8)	270·6 (243·6 to 301·5)	–10·8 (−43·4 to 6·3)	–0·9 (−14·2 to 5·7)	8·0 (6·7 to 11·9)	5·0 (4·5 to 5·7)	3·9 (3·5 to 4·4)	–50·9 (−68·2 to −42·0)*	–22·1 (−32·2 to −17·1)*
		Asbestosis		50·9 (40·8 to 70·6)	73·0 (57·2 to 86·9)	83·9 (67·9 to 97·5)	64·8 (29·6 to 83·7)*	14·8 (9·2 to 21·8)*	1·3 (1·1 to 1·9)	1·4 (1·1 to 1·6)	1·2 (1·0 to 1·4)	–5·8 (−26·4 to 4·6)	–9·2 (−13·7 to −3·3)*
		Coal workers’ pneumoconiosis		103·4 (70·5 to 127·8)	87·5 (65·5 to 104·6)	89·1 (70·2 to 108·9)	–13·9 (−30·6 to 15·1)	1·8 (−6·8 to 12·7)	2·9 (1·9 to 3·5)	1·7 (1·2 to 2·0)	1·3 (1·0 to 1·6)	–54·3 (−63·3 to −38·6)*	–21·6 (−28·2 to −13·5)*
		Other pneumoconiosis		110·2 (80·0 to 173·3)	126·6 (104·4 to 161·8)	133·5 (112·1 to 165·9)	21·2 (−8·3 to 52·8)	5·5 (−2·2 to 13·2)	2·9 (2·1 to 4·5)	2·3 (1·9 to 3·0)	1·9 (1·6 to 2·4)	–32·1 (−47·9 to −15·2)*	–16·7 (−22·7 to −10·6)*
	Asthma			23 840·9 (19 339·2 to 28 695·4)	22 827·4 (18 531·6 to 27 887·8)	23 720·5 (18 911·2 to 29 790·8)	–0·5 (−11·3 to 9·8)	3·9 (−0·8 to 8·2)	514·7 (419·4 to 621·4)	375·2 (305·9 to 454·5)	329·2 (263·1 to 413·2)	–36·0 (−44·1 to −28·2)*	–12·2 (−16·9 to −7·7)*
	Interstitial lung disease and pulmonary sarcoidosis			1285·5 (924·8 to 1966·3)	2052·5 (1506·0 to 2609·2)	2721·6 (2075·3 to 3177·4)	111·7 (55·3 to 165·5)*	32·6 (18·7 to 43·0)*	33·8 (24·7 to 50·2)	38·7 (28·4 to 48·6)	40·2 (30·5 to 46·9)	19·0 (−11·4 to 49·3)	3·9 (−6·4 to 11·7)
	Other chronic respiratory diseases			1328·6 (906·1 to 1714·7)	1652·5 (1286·3 to 1932·2)	2075·2 (1697·7 to 2315·1)	56·2 (27·5 to 93·2)*	25·6 (17·5 to 34·0)*	26·6 (18·3 to 33·4)	26·2 (20·3 to 30·5)	28·5 (23·3 to 31·8)	7·4 (−10·0 to 30·2)	9·0 (2·1 to 16·4)*
**Cirrhosis and other chronic liver diseases**				**28 184·1 (26 769·7 to 29 706·9)**	**36 122·1 (34 848·4 to 39 549·1)**	**38 856·7 (36 890·5 to 42 795·8)**	37·9 (28·7 to 49·4)*	7·6 (2·5 to 13·7)*	**659·0 (627·6 to 693·9)**	**603·3 (582·6 to 657·4)**	**531·1 (504·3 to 584·5)**	–19·4 (−24·6 to −12·8)*	–12·0 (−16·1 to −7·2)*
	Due to hepatitis B			7875·2 (7193·4 to 8627·5)	10 373·1 (9525·8 to 11 593·2)	11 240·7 (10 162·6 to 13 138·6)	42·7 (31·8 to 58·9)*	8·4 (2·5 to 15·3)*	189·9 (173·4 to 208·5)	173·9 (160·1 to 195·5)	153·3 (138·5 to 178·9)	–19·3 (−25·4 to −10·3)*	–11·8 (−16·5 to −6·1)*
	Due to hepatitis C			6333·5 (5732·9 to 7049·7)	8709·0 (7906·3 to 9751·8)	9769·1 (8819·4 to 10 985·0)	54·2 (44·4 to 66·7)*	12·2 (6·8 to 18·5)*	155·1 (139·9 to 172·8)	147·4 (133·6 to 164·2)	133·5 (120·7 to 150·0)	–13·9 (−19·3 to −7·3)*	–9·4 (−13·8 to −4·4)*
	Due to alcohol use			6466·4 (5920·0 to 7057·2)	8878·9 (8112·9 to 9688·5)	9754·0 (8873·6 to 10 861·8)	50·8 (42·0 to 61·5)*	9·9 (4·8 to 15·9)*	161·5 (148·2 to 175·7)	151·3 (138·5 to 165·1)	133·5 (121·6 to 148·2)	–17·3 (−22·0 to −11·6)*	–11·8 (−15·7 to −6·9)*
	Due to other causes			7509·0 (6800·3 to 8254·4)	8161·1 (7451·7 to 9036·0)	8092·8 (7310·3 to 9160·6)	7·8 (−1·9 to 20·4)	–0·8 (−5·9 to 5·0)	152·5 (138·5 to 167·9)	130·7 (119·2 to 144·8)	110·8 (100·2 to 125·3)	–27·4 (−32·9 to −19·2)*	–15·3 (−19·4 to −10·9)*
**Digestive diseases**				**32 345·7 (28 585·3 to 34 769·7)**	**33 020·0 (30 716·0 to 35 440·1)**	**34 368·9 (31 754·4 to 37 405·9)**	**6·3 (−0·5 to 17·9)**	4·1 (0·4 to 7·7)*	**720·8 (640·2 to 770·7)**	**561·3 (523·0 to 601·8)**	**484·9 (448·4 to 526·8)**	–32·7 (−36·3 to −26·0)*	–13·6 (−16·4 to −10·8)*
	Peptic ulcer disease			10 011·0 (8963·1 to 10 836·7)	7708·0 (7085·6 to 8454·8)	7106·0 (6459·4 to 8011·1)	–29·0 (−35·4 to −20·6)*	–7·8 (−11·8 to −3·8)*	237·1 (212·3 to 257·4)	134·0 (123·2 to 146·5)	100·2 (91·2 to 112·5)	–57·8 (−61·6 to −52·7)*	–25·2 (−28·4 to −22·0)*
	Gastritis and duodenitis			1952·5 (1571·5 to 2395·4)	2235·1 (1806·0 to 2810·5)	2689·8 (2104·0 to 3454·3)	37·8 (24·2 to 50·6)*	20·4 (13·5 to 26·7)*	45·9 (37·1 to 56·0)	38·4 (31·2 to 48·1)	37·7 (29·6 to 48·3)	–17·8 (−25·0 to −10·7)*	–1·7 (−7·2 to 3·2)
	Appendicitis			2200·3 (1726·6 to 2565·2)	2412·3 (2109·1 to 2744·1)	2150·0 (1928·1 to 2484·8)	–2·3 (−16·9 to 19·1)	–10·9 (−18·1 to −3·0)*	41·3 (32·8 to 47·4)	37·2 (32·3 to 42·2)	29·3 (26·3 to 33·9)	–29·0 (−38·5 to −15·1)*	–21·1 (−27·5 to −14·2)*
	Paralytic ileus and intestinal obstruction			6853·7 (5275·8 to 7778·5)	7379·6 (6085·8 to 7981·1)	7626·5 (6380·0 to 8328·6)	11·3 (−1·7 to 32·7)	3·4 (−2·8 to 10·6)	131·2 (101·5 to 146·5)	120·6 (99·3 to 130·0)	108·0 (90·5 to 117·7)	–17·7 (−25·7 to −3·7)*	–10·5 (−15·4 to −5·0)*
	Inguinal, femoral, and abdominal hernia			2405·0 (1863·4 to 3003·9)	2854·3 (2251·5 to 3551·6)	3106·1 (2401·1 to 3932·1)	29·1 (19·9 to 39·6)*	8·8 (5·5 to 11·4)*	54·2 (42·2 to 67·3)	48·2 (38·2 to 59·9)	43·5 (33·7 to 55·0)	–19·7 (−26·4 to −12·2)*	–9·7 (−12·8 to −7·6)*
	Inflammatory bowel disease			1217·7 (916·1 to 1735·5)	1606·7 (1305·0 to 2000·0)	1844·4 (1541·5 to 2195·5)	51·5 (14·2 to 81·3)*	14·8 (2·7 to 22·6)*	27·4 (21·5 to 36·3)	27·4 (22·3 to 33·5)	25·8 (21·6 to 30·7)	–6·0 (−23·3 to 7·3)	–5·9 (−14·8 to 0·3)
	Vascular intestinal disorders			1012·2 (904·2 to 1107·2)	1457·8 (1317·3 to 1656·0)	1702·6 (1568·9 to 1943·4)	68·2 (55·2 to 86·2)*	16·8 (10·4 to 23·5)*	28·1 (25·5 to 30·5)	28·1 (25·7 to 31·8)	25·5 (23·4 to 29·0)	–9·3 (−15·3 to 0·2)	–9·4 (−14·1 to −4·6)*
	Gallbladder and biliary diseases			1665·1 (1368·2 to 1821·1)	1851·7 (1729·6 to 2093·8)	2098·0 (1953·3 to 2401·6)	26·0 (14·4 to 56·3)*	13·3 (9·8 to 17·6)*	42·8 (35·5 to 47·3)	33·9 (31·8 to 38·2)	30·5 (28·4 to 35·0)	–28·8 (−36·1 to −12·3)*	–10·0 (−12·8 to −6·6)*
	Pancreatitis			2035·5 (1723·4 to 2341·3)	3046·0 (2707·5 to 3380·8)	3347·0 (2910·4 to 3722·1)	64·4 (44·7 to 82·4)*	9·9 (2·9 to 16·8)*	47·5 (40·4 to 54·7)	50·5 (44·9 to 56·2)	45·8 (39·8 to 50·9)	–3·6 (−15·1 to 6·6)	–9·4 (−15·1 to −3·9)*
	Other digestive diseases			2992·8 (2482·9 to 3546·7)	2468·6 (2189·1 to 2834·9)	2698·5 (2428·7 to 3008·7)	–9·8 (−24·4 to 4·2)	9·3 (1·9 to 17·8)*	65·2 (56·4 to 75·0)	43·0 (38·7 to 48·8)	38·6 (34·8 to 43·0)	–40·8 (−49·1 to −33·3)*	–10·2 (−15·5 to −4·2)*
**Neurological disorders**				**64 973·1 (50 343·0 to 80 732·5)**	**87 251·9 (67 830·9 to 107 955·1)**	**103 580·0 (81 171·2 to 128 122·4)**	59·4 (55·2 to 64·6)*	18·7 (17·0 to 20·7)*	**1500·6 (1192·1 to 1832·8)**	**1491·2 (1182·9 to 1824·0)**	**1478·4 (1171·9 to 1813·0)**	**–1·5 (−3·3 to 0·4)**	**–0·9 (−2·1 to 0·3)**
	Alzheimer's disease and other dementias			13 024·5 (11 052·7 to 15 479·9)	20 912·2 (17 919·7 to 24 689·5)	28 764·1 (24 510·8 to 33 952·4)	120·8 (115·3 to 126·5)*	37·5 (35·3 to 39·7)*	460·9 (394·5 to 544·4)	469·6 (403·2 to 552·4)	470·6 (401·2 to 556·3)	2·1 (0·1 to 3·8)*	0·2 (−1·1 to 1·5)
	Parkinson's disease			1304·3 (1024·7 to 1606·9)	2385·3 (1901·1 to 2910·3)	3234·5 (2563·6 to 4012·8)	148·0 (139·8 to 155·8)*	35·6 (32·9 to 38·2)*	42·0 (33·2 to 51·9)	49·9 (39·7 to 61·0)	51·3 (40·6 to 63·4)	22·1 (18·2 to 25·9)*	2·7 (1·0 to 4·5)*
	Epilepsy			12 420·8 (10 285·4 to 14 852·0)	13 435·2 (11 142·0 to 16 031·4)	13 492·2 (11 014·7 to 16 503·1)	8·6 (−2·9 to 23·7)	0·4 (−7·0 to 8·6)	226·5 (187·9 to 269·3)	201·6 (166·8 to 240·4)	182·6 (148·9 to 223·5)	–19·4 (−27·6 to −9·0)*	–9·4 (−16·1 to −2·2)*
	Multiple sclerosis			694·0 (586·0 to 807·1)	974·2 (827·1 to 1123·9)	1151·5 (968·6 to 1345·8)	65·9 (45·2 to 74·8)*	18·2 (12·8 to 21·9)*	16·3 (13·8 to 18·9)	16·2 (13·8 to 18·6)	15·6 (13·2 to 18·3)	–4·2 (−16·4 to 0·8)	–3·2 (−7·3 to −0·2)*
	Motor neuron disease			582·3 (527·8 to 651·4)	774·9 (745·5 to 817·5)	926·1 (881·6 to 961·8)	59·0 (44·5 to 71·6)*	19·5 (14·7 to 22·4)*	13·4 (12·5 to 14·6)	13·7 (13·2 to 14·3)	13·2 (12·5 to 13·7)	–1·5 (−9·3 to 2·9)	–3·6 (−7·3 to −1·3)*
	Migraine			29 843·4 (19 092·9 to 41 793·9)	39 485·3 (25 341·5 to 55 187·3)	45 121·9 (29 045·8 to 62 826·9)	51·2 (49·7 to 52·8)*	14·3 (13·7 to 14·9)*	599·9 (385·7 to 839·1)	597·8 (384·6 to 833·2)	598·6 (385·9 to 833·3)	–0·2 (−0·8 to 0·4)	0·1 (−0·2 to 0·5)
	Tension-type headache			4700·9 (2968·1 to 6989·3)	6236·2 (3972·6 to 9204·2)	7195·1 (4614·6 to 10 499·9)	53·1 (47·5 to 58·4)*	15·4 (13·7 to 17·0)*	96·2 (61·1 to 142·5)	95·5 (61·2 to 139·9)	95·9 (61·5 to 140·0)	–0·2 (−2·5 to 1·9)	0·4 (−0·5 to 1·4)
	Other neurological disorders			2402·9 (2004·0 to 2829·7)	3048·7 (2593·3 to 3555·3)	3694·5 (3114·1 to 4353·4)	53·8 (38·5 to 67·8)*	21·2 (14·0 to 28·5)*	45·538·7 to 52·8)	46·9 (40·2 to 54·4)	50·6 (42·7 to 59·5)	11·2 (2·1 to 20·0)*	7·9 (1·9 to 14·2)*
**Mental and substance use disorders**				**110** **918·3 (83** **056·1 to 141** **228·0)**	**145** **067·1 (108** **650·3 to 183** **887·6)**	**162 509·3 (121 886·4 to 206 517·4)**	46·5 (44·9 to 48·7)*	12·0 (11·2 to 12·9)*	**2240·8 (1681·3 to 2844·2)**	**2226·6 (1669·0 to 2821·8)**	**2172·7 (1629·4 to 2761·5)**	–3·0 (−4·1 to −1·8)*	–2·4 (−3·2 to −1·7)*
	Schizophrenia			8447·6 (6181·9 to 10** **521·3)	11** **492·6 (8420·0 to 14** **326·7)	13** **414·3 (9858·7 to 16** **714·0)	58·8 (55·8 to 62·0)*	16·7 (15·5 to 18·0)*	179·6 (132·2 to 222·3)	178·9 (132·0 to 222·3)	177·2 (130·5 to 220·3)	–1·3 (−2·2 to −0·5)*	–0·9 (−1·7 to −0·2)*
	Alcohol use disorders			11** **264·3 (9057·6 to 14** **013·8)	15** **561·7 (12** **607·6 to 19** **100·6)	16** **244·7 (13** **003·1 to 19** **955·3)	44·2 (37·4 to 51·0)*	4·4 (0·4 to 8·6)*	237·3 (192·3 to 292·7)	240·9 (196·4 to 293·9)	214·4 (172·0 to 262·9)	–9·6 (−14·3 to −5·5)*	–11·0 (−14·6 to −7·5)*
	Drug use disorders			14** **247·5 (11** **250·4 to 17** **370·8)	18** **009·0 (14** **444·2 to 21** **739·6)	20** **394·2 (16** **204·4 to 24** **670·3)	43·1 (36·5 to 58·5)*	13·2 (9·8 to 16·9)*	278·6 (221·2 to 337·7)	270·5 (217·3 to 325·1)	268·4 (213·5 to 324·0)	–3·6 (−8·1 to 6·7)	–0·8 (−3·8 to 2·4)
		Opioid use disorders		10** **261·8 (8052·7 to 12** **572·0)	12** **817·5 (10** **018·1 to 15** **694·7)	14** **788·8 (11** **380·5 to 18** **259·7)	44·1 (37·5 to 56·0)*	15·4 (11·2 to 19·5)*	202·0 (159·3 to 247·4)	193·1 (151·2 to 236·4)	194·3 (149·8 to 239·6)	–3·8 (−8·3 to 3·8)	0·7 (−3·0 to 4·2)
		Cocaine use disorders		790·1 (568·0 to 1051·1)	1060·8 (780·4 to 1375·9)	1154·2 (847·2 to 1512·1)	46·1 (38·4 to 65·1)*	8·8 (5·1 to 12·2)*	15·7 (11·3 to 20·8)	16·1 (11·8 to 20·8)	15·3 (11·2 to 20·0)	–2·6 (−7·8 to 9·6)	–4·9 (−8·3 to −1·7)*
		Amphetamine use disorders		660·3 (430·6 to 985·6)	834·2 (567·1 to 1190·4)	881·7 (599·5 to 1243·1)	33·5 (23·0 to 54·2)*	5·7 (1·8 to 10·2)*	11·8 (7·8 to 17·3)	12·0 (8·2 to 17·0)	11·5 (7·8 to 16·2)	–1·9 (−8·8 to 13·2)	–3·7 (−7·5 to 0·3)
		Cannabis use disorders		514·5 (321·8 to 757·2)	623·9 (389·2 to 905·4)	646·9 (400·9 to 945·5)	25·7 (21·7 to 29·8)*	3·7 (1·2 to 6·0)*	9·1 (5·7 to 13·3)	8·8 (5·5 to 12·9)	8·5 (5·2 to 12·4)	–6·9 (−8·9 to −5·0)*	–4·2 (−5·9 to −2·4)*
		Other drug use disorders		2020·8 (1550·8 to 2453·2)	2672·5 (2235·2 to 3177·1)	2922·5 (2425·1 to 3504·3)	44·6 (31·6 to 89·2)*	9·3 (3·8 to 14·5)*	40·0 (30·8 to 48·3)	40·5 (34·0 to 47·8)	38·8 (32·3 to 46·4)	–3·0 (−12·0 to 26·0)	–4·3 (−9·3 to 0·2)
	Depressive disorders			29** **503·5 (20** **318·2 to 40** **109·0)	39** **052·4 (26** **930·5 to 52** **535·2)	44** **208·4 (30** **573·2 to 59** **878·5)	49·8 (46·9 to 53·1)*	13·2 (12·2 to 14·4)*	630·6 (438·2 to 852·5)	620·1 (430·6 to 831·7)	597·9 (414·5 to 806·2)	–5·2 (−6·2 to −4·1)*	–3·6 (−4·3 to −2·9)*
		Major depressive disorder		23** **423·5 (16** **150·4 to 31** **868·1)	30** **670·3 (21** **102·1 to 41** **462·3)	34** **104·6 (23** **469·5 to 46** **039·4)	45·6 (42·4 to 49·2)*	11·2 (10·1 to 12·3)*	495·7 (341·2 to 671·8)	484·6 (334·7 to 655·2)	461·1 (317·9 to 622·5)	–7·0 (−8·1 to −5·8)*	–4·9 (−5·6 to −4·1)*
		Dysthymia		6080·0 (4134·8 to 8839·6)	8382·0 (5676·3 to 12 123·1)	10** **103·8 (6860·6 to 14** **611·5)	66·2 (62·6 to 69·8)*	20·5 (18·3 to 23·1)*	134·9 (91·8 to 195·5)	135·5 (91·6 to 196·3)	136·8 (93·0 to 197·6)	1·4 (0·5 to 2·3)*	1·0 (−0·5 to 2·7)
		Bipolar disorder		5873·0 (3645·7 to 8618·8)	7795·1 (4869·3 to 11** **432·4)	8954·0 (5588·3 to 13** **186·4)	52·5 (50·0 to 55·0)*	14·9 (13·8 to 16·0)*	118·2 (73·8 to 173·0)	118·3 (74·1 to 173·1)	119·3 (74·7 to 175·1)	0·9 (0·1 to 1·8)*	0·8 (0·2 to 1·4)*
	Anxiety disorders			17** **893·2 (12** **472·9 to 24** **294·6)	23** **364·5 (16** **285·6 to 31** **632·0)	26** **417·4 (18** **440·4 to 35** **634·4)	47·6 (45·4 to 49·8)*	13·1 (11·9 to 14·3)*	357·0 (249·8 to 482·4)	356·6 (248·7 to 481·7)	354·2 (247·1 to 477·8)	–0·8 (−1·8 to 0·2)	–0·7 (−1·7 to 0·2)
	Eating disorders			1400·4 (920·5 to 2016·6)	1861·9 (1222·9 to 2684·6)	2180·1 (1420·8 to 3122·9)	55·7 (52·2 to 59·3)*	17·1 (15·2 to 18·8)*	24·6 (16·2 to 35·3)	26·2 (17·2 to 37·7)	28·6 (18·6 to 40·8)	16·1 (14·3 to 17·7)*	8·9 (7·6 to 10·1)*
		Anorexia nervosa		418·9 (263·0 to 626·4)	523·9 (329·8 to 776·8)	584·7 (367·2 to 859·0)	39·6 (36·1 to 43·4)*	11·6 (9·2 to 14·0)*	7·1 (4·5 to 10·6)	7·3 (4·6 to 10·8)	7·7 (4·8 to 11·3)	8·1 (5·6 to 10·5)*	5·4 (3·2 to 7·4)*
		Bulimia nervosa		981·5 (602·8 to 1471·9)	1338·0 (818·2 to 1997·1)	1595·4 (974·5 to 2394·5)	62·5 (58·5 to 66·9)*	19·2 (16·9 to 21·1)*	17·5 (10·6 to 26·3)	18·9 (11·5 to 28·4)	20·9 (12·7 to 31·2)	19·4 (17·5 to 21·2)*	10·2 (8·7 to 11·7)*
	Autistic spectrum disorders			6525·8 (4418·6 to 9180·8)	8104·7 (5491·2 to 11 393·0)	9025·7 (6119·0 to 12 681·1)	38·3 (37·3 to 39·4)*	11·4 (10·8 to 12·0)*	120·6 (81·9 to 169·6)	121·1 (82·1 to 170·2)	121·4 (82·3 to 170·6)	0·6 (0·0 to 1·2)*	0·3 (−0·2 to 0·8)
		Autism		3371·4 (2170·7 to 4891·2)	4178·3 (2693·8 to 6041·1)	4649·0 (2976·6 to 6699·0)	37·9 (36·3 to 39·6)*	11·3 (10·3 to 12·3)*	62·8 (40·4 to 90·9)	62·6 (40·3 to 90·4)	62·5 (40·0 to 90·2)	–0·4 (−1·3 to 0·5)	–0·1 (−0·9 to 0·8)
		Asperger syndrome and other autistic spectrum disorders		3154·3 (2078·1 to 4617·6)	3926·4 (2574·5 to 5735·4)	4376·7 (2864·1 to 6392·9)	38·8 (37·5 to 40·1)*	11·5 (10·9 to 12·1)*	57·8 (38·0 to 84·2)	58·5 (38·3 to 85·2)	58·9 (38·5 to 85·9)	1·8 (1·2 to 2·3)*	0·6 (0·1 to 1·1)*
	Attention-deficit hyperactivity disorder			599·7 (359·0 to 952·7)	711·9 (426·0 to 1134·5)	755·2 (452·2 to 1196·6)	25·9 (23·5 to 28·3)*	6·1 (5·0 to 7·1)*	10·2 (6·1 to 16·1)	10·1 (6·0 to 16·1)	10·1 (6·0 to 15·9)	–1·3 (−3·0 to 0·5)	–0·5 (−1·4 to 0·4)
	Conduct disorder			5072·5 (3154·4 to 7682·5)	5820·5 (3615·8 to 8803·8)	5947·3 (3701·9 to 8998·5)	17·2 (15·6 to 18·7)*	2·2 (0·9 to 3·4)*	79·5 (49·4 to 120·5)	79·4 (49·4 to 120·6)	81·2 (50·5 to 122·9)	2·1 (0·7 to 3·4)*	2·3 (1·1 to 3·4)*
	Idiopathic developmental intellectual disability			3629·4 (1741·7 to 6242·4)	4500·4 (2160·1 to 7650·1)	4610·9 (2201·5 to 7932·1)	27·1 (21·7 to 30·9)	2·5 (−0·9 to 4·6)	66·2 (31·8 to 113·6)	66·1 (31·8 to 112·4)	61·6 (29·4 to 105·9)	–7·0 (−11·0 to–4·1)	–6·9 (−10·0 to–4·9)
	Other mental and substance use disorders			6461·6 (4417·8 to 9255·4)	8792·3 (5989·8 to 12 580·6)	10** **357·1 (7059·0 to 14** **807·2)	60·3 (59·0 to 61·9)*	17·8 (17·2 to 18·5)*	138·5 (94·5 to 197·7)	138·4 (94·3 to 197·8)	138·5 (94·4 to 197·7)	0·0 (−0·5 to 0·5)	0·1 (−0·4 to 0·5)
**Diabetes, urogenital, blood, and endocrine diseases**				**81 744·6 (71 204·2 to 94 535·9)**	**112 751·7 (98 101·9 to 131 367·5)**	**133 747·8 (115 976·8 to 155 676·1)**	63·6 (59·4 to 68·0)*	18·6 (17·4 to 20·0)*	**1882·2 (1642·5 to 2163·3)**	**1942·7 (1699·4 to 2247·3)**	**1887·6 (1641·9 to 2192·9)**	**0·3 (−1·8 to 2·3)**	–2·8 (−3·9 to −1·7)*
	Diabetes mellitus			27** **475·9 (23** **282·7 to 32** **608·1)	45** **989·7 (38** **695·1 to 54** **713·4)	57** **233·7 (47** **967·9 to 68** **279·3)	108·3 (104·2 to 112·1)*	24·4 (22·7 to 26·2)*	708·6 (604·0 to 837·1)	827·9 (700·5 to 977·9)	814·2 (686·6 to 965·4)	14·9 (12·6 to 16·9)*	–1·6 (−3·0 to −0·2)*
	Acute glomerulonephritis			560·5 (521·8 to 610·3)	337·8 (325·7 to 355·6)	325·6 (310·5 to 343·1)	–41·9 (−46·6 to −37·0)*	–3·6 (−8·4 to 1·5)	11·6 (10·8 to 12·5)	5·5 (5·3 to 5·8)	4·5 (4·3 to 4·8)	–60·9 (−63·9 to −57·6)*	–17·4 (−21·5 to −13·2)*
	Chronic kidney disease			21** **597·2 (20** **094·0 to 23** **354·9)	29** **187·2 (27** **250·7 to 31** **463·0)	35** **032·4 (32** **622·1 to 37** **954·3)	62·2 (56·5 to 68·0)*	20·0 (17·4 to 22·7)*	521·4 (484·6 to 565·3)	515·4 (480·9 to 555·7)	500·1 (465·5 to 541·4)	–4·1 (−7·5 to −1·2)*	–3·0 (−5·0 to −1·0)*
		Due to diabetes mellitus		7860·2 (7048·8 to 8711·8)	11** **731·4 (10** **615·3 to 12** **891·9)	14** **660·6 (13** **206·6 to 16** **203·7)	86·5 (77·5 to 92·6)*	25·0 (21·9 to 27·6)*	202·9 (182·1 to 225·3)	212·1 (191·8 to 233·4)	209·2 (188·8 to 230·8)	3·1 (−1·4 to 6·1)	–1·4 (−3·5 to 0·5)
		Due to hypertension		3481·1 (3030·8 to 3987·5)	5169·1 (4520·8 to 5845·6)	6606·7 (5760·2 to 7493·9)	89·8 (80·4 to 96·3)*	27·8 (24·7 to 30·6)*	97·3 (84·5 to 110·8)	99·2 (87·0 to 112·3)	98·2 (85·5 to 111·1)	0·9 (−3·9 to 4·0)	–1·0 (−3·3 to 0·9)
		Due to glomerulonephritis		4609·2 (4053·1 to 5203·8)	5467·8 (4843·2 to 6157·1)	5932·9 (5226·4 to 6746·0)	28·7 (22·3 to 36·4)*	8·5 (5·5 to 11·8)*	96·8 (85·7 to 109·7)	89·0 (79·1 to 100·4)	82·2 (72·8 to 93·3)	–15·1 (−18·2 to −11·3)*	–7·7 (−9·8 to −5·4)*
		Due to other causes		5646·7 (5024·1 to 6347·1)	6819·0 (6061·0 to 7661·1)	7832·1 (6915·6 to 8848·7)	38·7 (31·1 to 47·3)*	14·9 (11·6 to 18·4)*	124·5 (110·6 to 141·4)	115·1 (102·5 to 129·8)	110·6 (97·5 to 125·5)	–11·2 (−14·7 to −7·3)*	–4·0 (−6·2 to −1·4)*
	Urinary diseases and male infertility			6451·7 (5595·1 to 7476·6)	8383·8 (7248·0 to 9800·2)	9965·5 (8532·5 to 11 725·6)	54·5 (43·5 to 61·3)*	18·9 (14·9 to 21·6)*	152·8 (131·3 to 179·4)	148·8 (128·2 to 175·0)	143·5 (122·7 to 169·2)	–6·1 (−11·5 to −2·8)*	–3·6 (−6·5 to −1·5)*
		Interstitial nephritis and urinary tract infections		2443·8 (2111·8 to 2752·8)	3457·4 (3137·6 to 3688·9)	4269·2 (4005·5 to 4529·9)	74·7 (54·0 to 95·1)*	23·5 (15·5 to 30·3)*	55·6 (48·9 to 61·3)	60·7 (55·4 to 64·5)	61·9 (58·2 to 65·6)	11·3 (−0·3 to 22·0)	1·9 (−4·2 to 7·3)
		Urolithiasis		496·9 (352·4 to 588·4)	566·9 (491·0 to 658·3)	622·5 (523·5 to 778·9)	25·3 (7·1 to 86·6)*	9·8 (2·6 to 25·5)*	11·9 (8·6 to 14·1)	9·7 (8·4 to 11·2)	8·8 (7·4 to 11·0)	–26·5 (−36·9 to 8·6)	–9·8 (−15·6 to 3·0)
		Benign prostatic hyperplasia		1818·5 (1164·2 to 2622·2)	2625·2 (1692·9 to 3763·4)	3383·9 (2178·0 to 4835·1)	86·1 (82·2 to 90·8)*	28·9 (27·6 to 30·3)*	50·4 (32·4 to 72·4)	49·9 (32·2 to 72·1)	49·2 (31·8 to 71·0)	–2·3 (−4·2 to −0·1)*	–1·3 (−2·2 to −0·3)*
		Male infertility		104·7 (42·2 to 207·7)	138·3 (55·3 to 275·1)	165·1 (66·1 to 327·1)	57·7 (52·9 to 62·6)*	19·3 (15·7 to 22·6)*	2·0 (0·8 to 3·9)	2·0 (0·8 to 4·0)	2·1 (0·9 to 4·3)	9·5 (6·8 to 12·1)*	7·9 (4·9 to 10·6)*
		Other urinary diseases		1587·8 (1231·6 to 1910·8)	1596·0 (1346·0 to 1852·1)	1524·9 (1353·8 to 1722·1)	–4·0 (−17·5 to 18·2)	–4·5 (−12·0 to 7·6)	32·9 (25·7 to 39·6)	26·5 (22·4 to 30·7)	21·4 (19·1 to 24·2)	–34·7 (−44·6 to −20·4)*	–19·0 (−25·5 to −9·1)*
	Gynaecological diseases			7068·6 (4854·9 to 10 047·8)	9365·7 (6439·4 to 13 315·0)	10 460·2 (7184·1 to 14 928·1)	48·0 (45·8 to 50·2)*	11·7 (10·4 to 13·1)*	145·2 (99·4 to 206·1)	140·4 (96·1 to 200·0)	136·8 (94·0 to 195·1)	–5·8 (−7·2 to −4·3)*	–2·5 (−3·5 to −1·4)*
		Uterine fibroids		882·6 (552·9 to 1378·7)	1237·0 (765·6 to 1961·3)	1462·5 (897·4 to 2340·2)	65·7 (59·0 to 71·2)*	18·2 (16·3 to 19·8)*	20·1 (12·6 to 31·5)	19·3 (12·0 to 30·7)	19·2 (11·8 to 30·7)	–4·6 (−8·6 to −1·5)*	–0·6 (−2·2 to 0·7)
		Polycystic ovarian syndrome		98·7 (53·5 to 166·2)	108·9 (57·8 to 188·7)	112·4 (59·4 to 198·0)	14·0 (−4·7 to 29·3)	3·3 (−4·0 to 8·3)	1·9 (1·1 to 3·3)	1·6 (0·8 to 2·7)	1·5 (0·8 to 2·6)	–24·7 (−39·1 to −12·8)*	–7·3 (−14·5 to −2·3)*
		Female infertility		103·8 (37·9 to 234·0)	129·8 (47·2 to 288·4)	179·8 (66·2 to 399·0)	73·2 (58·8 to 91·0)*	38·5 (29·4 to 49·5)*	1·9 (0·7 to 4·3)	1·9 (0·7 to 4·2)	2·3 (0·9 to 5·2)	23·2 (13·8 to 35·5)*	25·3 (17·7 to 34·8)*
		Endometriosis		233·8 (156·3 to 325·3)	310·3 (209·4 to 429·1)	334·9 (226·3 to 461·8)	43·2 (36·4 to 50·6)*	7·9 (5·3 to 10·9)*	4·4 (3·0 to 6·2)	4·5 (3·0 to 6·2)	4·3 (2·9 to 6·0)	–2·3 (−5·9 to 1·4)	–3·8 (−6·1 to −1·4)*
		Genital prolapse		586·6 (297·3 to 1051·1)	694·7 (348·9 to 1246·0)	792·6 (394·6 to 1424·8)	35·1 (31·0 to 38·0)*	14·1 (12·5 to 15·4)*	14·9 (7·5 to 26·6)	12·3 (6·2 to 22·0)	11·1 (5·5 to 19·8)	–25·8 (−27·9 to −24·3)*	–10·0 (−11·2 to −9·0)*
		Premenstrual syndrome		2632·6 (1628·3 to 4036·0)	3470·7 (2146·2 to 5295·9)	3791·1 (2344·6 to 5812·9)	44·0 (42·4 to 45·5)*	9·2 (7·7 to 10·6)*	50·7 (31·3 to 77·2)	50·3 (31·1 to 76·7)	49·3 (30·5 to 75·6)	–2·7 (−3·7 to −1·9)*	–2·0 (−3·3 to −0·8)*
		Other gynaecological diseases		2530·6 (1722·7 to 3505·2)	3414·2 (2325·2 to 4737·6)	3786·8 (2576·9 to 5232·9)	49·6 (44·5 to 55·1)*	10·9 (8·6 to 13·8)*	51·2 (34·8 to 71·6)	50·5 (34·4 to 70·1)	49·2 (33·5 to 67·9)	–4·1 (−6·9 to −0·6)*	–2·7 (−4·7 to −0·1)*
	Haemoglobinopathies and haemolytic anaemias			12** **160·7 (9789·5 to 14** **856·4)	12** **033·2 (9847·5 to 14** **691·3)	12** **321·6 (9916·9 to 15** **372·2)	1·3 (−8·3 to 15·8)	2·4 (−2·6 to 8·3)	213·4 (174·0 to 258·9)	181·7 (148·9 to 221·9)	169·7 (136·9 to 211·4)	–20·5 (−27·2 to −10·5)*	–6·6 (−10·9 to −1·3)*
		Thalassaemias		1432·0 (961·6 to 1835·3)	771·2 (639·7 to 918·5)	516·0 (443·5 to 633·5)	–64·0 (−70·0 to −40·5)*	–33·1 (−39·9 to −18·8)*	21·7 (14·6 to 27·8)	11·2 (9·3 to 13·4)	7·2 (6·2 to 8·9)	–66·6 (−72·1 to −45·0)*	–35·5 (−42·1 to −21·6)*
		Thalassaemias trait		2703·8 (1770·0 to 3974·9)	2957·1 (1959·0 to 4290·6)	3280·4 (2156·8 to 4823·8)	21·3 (17·3 to 25·1)*	10·9 (8·0 to 13·9)*	49·7 (32·6 to 73·1)	44·9 (29·7 to 65·2)	44·6 (29·3 to 65·5)	–10·2 (−12·1 to −8·4)*	–0·6 (−3·1 to 2·0)
		Sickle cell disorders		4349·9 (3163·6 to 5209·6)	4224·4 (3414·2 to 4951·4)	4117·9 (3587·8 to 4842·0)	–5·3 (−20·7 to 19·9)	–2·5 (−11·6 to 10·5)	68·7 (50·4 to 81·8)	60·8 (49·0 to 71·5)	56·6 (49·1 to 66·5)	–17·7 (−30·6 to 3·8)	–6·9 (−15·9 to 6·1)
		Sickle cell trait		1119·1 (726·0 to 1651·0)	1331·4 (871·9 to 1959·4)	1555·0 (1014·1 to 2282·9)	39·0 (36·2 to 41·7)*	16·8 (14·8 to 18·8)*	19·5 (12·8 to 28·8)	19·7 (12·9 to 28·9)	21·1 (13·8 to 31·0)	8·1 (6·2 to 9·7)*	7·2 (5·4 to 9·1)*
		G6PD deficiency		577·8 (488·8 to 691·1)	702·7 (606·1 to 834·8)	737·8 (637·7 to 875·0)	27·7 (18·3 to 42·4)*	5·0 (1·2 to 10·1)*	10·9 (9·2 to 13·1)	10·7 (9·3 to 12·7)	9·9 (8·6 to 11·8)	–8·8 (−14·7 to 1·2)	–7·3 (−10·7 to −2·8)*
		G6PD trait		0·4 (0·3 to 0·6)	0·5 (0·3 to 0·7)	0·6 (0·4 to 0·8)	37·0 (30·4 to 43·2)*	17·6 (14·9 to 20·7)*	··	··	··	–3·0 (−6·5 to 0·1)	4·9 (2·6 to 7·5)*
		Other haemoglobinopathies and haemolytic anaemias		1977·7 (1591·8 to 2467·5)	2046·0 (1610·7 to 2612·6)	2113·9 (1640·0 to 2730·4)	6·9 (0·9 to 13·2)*	3·3 (0·6 to 5·8)*	42·9 (35·4 to 52·5)	34·4 (27·5 to 43·3)	30·2 (23·6 to 38·9)	–29·4 (−34·6 to −23·9)*	–12·1 (−15·2 to −9·4)*
	Endocrine, metabolic, blood, and immune disorders			6430·0 (5399·9 to 7454·1)	7454·3 (6326·0 to 8750·0)	8408·8 (7025·4 to 9979·2)	30·8 (20·9 to 40·9)*	12·8 (9·0 to 16·5)*	129·3 (108·6 to 151·1)	123·0 (104·1 to 144·5)	118·7 (99·1 to 140·9)	–8·2 (−13·4 to −3·2)*	–3·5 (−6·4 to −0·8)*
**Musculoskeletal disorders**				**86 655·4 (63 137·9 to 112 703·8)**	**117 031·2 (85 442·0 to 152 359·0)**	**140 030·6 (102 331·5 to 181 687·7)**	61·6 (59·1 to 63·9)*	19·6 (18·5 to 20·8)*	**2014·2 (1474·2 to 2609·4)**	**1944·7 (1422·3 to 2518·0)**	**1918·7 (1404·4 to 2493·3)**	–4·7 (−5·7 to −3·9)*	–1·3 (−2·0 to −0·6)*
Rheumatoid arthritis				3329·7 (2458·2 to 4257·7)	4443·0 (3238·4 to 5693·1)	5563·4 (3985·9 to 7171·2)	67·1 (60·8 to 72·7)*	25·2 (22·4 to 27·6)*	82·8 (61·6 to 104·8)	77·7 (56·9 to 99·3)	78·0 (55·8 to 100·1)	–5·7 (−9·4 to −2·9)*	0·4 (−1·8 to 2·2)
Osteoarthritis				7947·9 (5593·8 to 10 856·2)	12** **385·2 (8735·1 to 16** **806·2)	16** **282·9 (11** **486·0 to 22** **047·2)	104·9 (102·9 to 107·2)*	31·5 (30·8 to 32·2)*	213·4 (150·2 to 291·8)	226·6 (159·7 to 308·0)	232·1 (163·7 to 313·9)	8·8 (7·6 to 10·2)*	2·4 (1·9 to 3·0)*
Low back and neck pain				55** **941·4 (39** **562·5 to 73** **187·3)	72** **599·3 (51** **532·0 to 94** **807·4)	86** **584·5 (61** **335·4 to 113** **628·5)	54·8 (51·8 to 57·4)*	19·3 (17·7 to 20·7)*	1294·3 (914·6 to 1702·0)	1198·9 (855·0 to 1573·7)	1182·7 (837·4 to 1550·6)	–8·6 (−9·6 to −7·7)*	–1·4 (−2·4 to −0·5)*
	Low back pain			39** **129·9 (27** **512·7 to 51** **797·6)	48** **853·4 (34** **564·0 to 64** **073·6)	57** **648·2 (40** **820·5 to 75** **877·0)	47·3 (44·4 to 50·1)*	18·0 (16·0 to 20·1)*	897·3 (637·6 to 1178·5)	805·3 (574·2 to 1053·7)	788·9 (558·7 to 1034·6)	–12·1 (−12·9 to −11·3)*	–2·0 (−3·6 to −0·9)*
	Neck pain			16** **811·5 (11** **404·8 to 23** **792·2)	23** **745·9 (16** **214·4 to 33** **438·2)	28** **936·3 (19** **578·5 to 40** **543·1)	72·1 (68·5 to 76·2)*	21·9 (19·9 to 24·0)*	397·0 (268·6 to 556·8)	393·6 (269·1 to 549·8)	393·8 (267·8 to 550·2)	–0·8 (−2·2 to 0·7)	0·1 (−1·3 to 1·4)
Gout				596·8 (413·3 to 810·6)	848·9 (589·9 to 1155·4)	1071·2 (742·4 to 1455·0)	79·5 (76·2 to 82·9)*	26·2 (24·3 to 27·9)*	15·4 (10·6 to 21·1)	15·2 (10·4 to 20·6)	15·2 (10·4 to 20·6)	–1·8 (−3·3 to −0·2)*	–0·1 (−1·3 to 1·1)
Other musculoskeletal disorders				18** **839·6 (13** **162·2 to 26** **102·7)	26** **754·8 (18 664·4 to 36 839·1)	30** **528·6 (21 196·7 to 42 458·6)	62·0 (57·4 to 67·0)*	14·1 (11·4 to 16·8)*	408·3 (285·4 to 564·2)	426·3 (298·3 to 586·2)	410·6 (285·6 to 568·6)	0·6 (−1·3 to 2·1)	–3·7 (−5·7 to −1·8)*
**Other non-communicable diseases**				**164 624·2 (127 834·6 to 209 136·6)**	**183 247·3 (141 607·2 to 236 700·6)**	**196 036·1 (148 295·9 to 258 150·6)**	19·1 (6·3 to 31·0)*	7·0 (2·4 to 10·8)*	**3107·0 (2367·7 to 4005·6)**	**2912·8 (2241·2 to 3781·4)**	**2758·6 (2096·2 to 3616·8)**	–11·2 (−17·7 to −5·3)*	–5·3 (−8·4 to −2·6)*
	Congenital birth defects			68** **393·0 (53** **592·7 to 84** **283·8)	58** **286·5 (48** **759·3 to 66** **905·7)	50** **429·8 (44** **114·0 to 56** **200·2)	–26·3 (−38·1 to −8·0)*	–13·5 (−20·8 to −5·2)*	1042·0 (821·5 to 1280·7)	853·9 (714·0 to 980·0)	716·0 (626·4 to 798·7)	–31·3 (−42·1 to −15·1)*	–16·1 (−23·1 to −8·3)*
		Neural tube defects		10** **315·0 (7239·9 to 14** **462·5)	6150·2 (4476·4 to 8763·9)	5130·9 (3867·1 to 7016·1)	–50·3 (−58·8 to −39·9)*	–16·6 (−27·1 to −5·4)*	156·9 (111·4 to 218·6)	90·3 (65·8 to 128·5)	72·6 (54·5 to 99·5)	–53·8 (−61·3 to −44·5)*	–19·6 (−29·7 to −9·2)*
		Congenital heart anomalies		27** **581·3 (21** **339·9 to 34** **673·0)	22** **936·6 (19** **833·8 to 26** **150·9)	18** **563·8 (16** **539·0 to 21** **464·8)	–32·7 (−44·5 to −7·0)*	–19·1 (−26·5 to −6·9)*	413·5 (320·8 to 519·5)	334·6 (289·1 to 381·8)	264·7 (235·7 to 305·9)	–36·0 (−47·0 to −11·7)*	–20·9 (−28·1 to −9·0)*
		Orofacial clefts		480·8 (242·6 to 688·3)	320·2 (200·3 to 468·4)	238·7 (148·0 to 369·6)	–50·4 (−67·5 to −18·4)*	–25·5 (−41·3 to −8·5)*	7·2 (3·7 to 10·2)	4·7 (2·9 to 6·8)	3·4 (2·1 to 5·3)	–52·4 (−68·8 to −22·9)*	–27·0 (−42·6 to −10·0)*
		Down's syndrome		1325·4 (767·0 to 3069·7)	1240·1 (945·4 to 2039·8)	1166·0 (1005·7 to 1482·8)	–12·0 (−54·8 to 41·0)	–6·0 (−30·4 to 12·8)	20·7 (12·4 to 46·5)	18·3 (14·0 to 30·1)	16·3 (14·0 to 20·7)	–21·4 (−58·2 to 22·5)	–11·2 (−33·7 to 6·2)
		Turner syndrome		37·8 (18·1 to 60·9)	44·2 (21·1 to 71·5)	47·3 (22·1 to 76·5)	25·2 (20·6 to 30·2)*	6·9 (3·2 to 10·4)*	0·7 (0·3 to 1·1)	0·6 (0·3 to 1·0)	0·6 (0·3 to 1·0)	–3·4 (−6·7 to −0·1)*	–1·0 (−4·3 to 2·1)
		Klinefelter syndrome		13·0 (6·3 to 24·4)	15·7 (7·6 to 29·6)	17·1 (8·2 to 32·3)	32·1 (28·5 to 35·8)*	9·0 (6·2 to 11·7)*	0·2 (0·1 to 0·4)	0·2 (0·1 to 0·4)	0·2 (0·1 to 0·4)	–0·3 (−2·6 to 2·3)	–0·1 (−2·5 to 2·5)
		Other chromosomal abnormalities		1668·6 (1053·4 to 3061·2)	1851·6 (1371·3 to 2805·1)	1952·2 (1542·5 to 2587·9)	17·0 (−18·1 to 54·2)	5·4 (−10·1 to 18·4)	24·9 (15·8 to 45·4)	26·8 (19·9 to 40·8)	27·9 (22·0 to 37·1)	12·2 (−20·7 to 47·3)	4·0 (−11·4 to 16·7)
		Congenital musculoskeletal and limb anomalies		2120·7 (1465·8 to 3497·7)	2234·9 (1621·0 to 3167·4)	2258·4 (1661·2 to 2988·8)	6·5 (−21·7 to 26·8)	1·1 (−12·2 to 9·4)	36·6 (25·7 to 57·3)	33·6 (24·4 to 47·1)	31·2 (23·0 to 41·5)	–14·6 (−32·7 to −2·5)*	–7·0 (−17·7 to −0·0)*
		Urogenital congenital anomalies		1277·1 (845·2 to 1639·7)	1202·0 (877·2 to 1462·4)	1085·9 (858·1 to 1305·3)	–15·0 (−35·6 to 10·8)	–9·7 (−19·8 to 3·1)	19·4 (12·9 to 24·9)	17·6 (12·8 to 21·4)	15·4 (12·2 to 18·6)	–20·4 (−39·5 to 2·8)	–12·1 (−21·9 to 0·3)
		Digestive congenital anomalies		4666·6 (3171·1 to 8658·0)	3973·4 (3024·5 to 6520·3)	3343·0 (2674·6 to 4964·7)	–28·4 (−49·5 to −3·2)*	–15·9 (−27·9 to −1·6)*	69·9 (47·8 to 128·5)	58·2 (44·3 to 95·3)	48·0 (38·3 to 71·5)	–31·3 (−51·3 to −8·1)*	–17·5 (−29·3 to −3·8)*
		Other congenital birth defects		18 906·7 (10 942·1 to 30 995·8)	18 317·5 (12 204·8 to 26 209·6)	16 626·6 (12 154·3 to 21 604·2)	–12·1 (−33·0 to 18·4)	–9·2 (−20·5 to 3·7)	291·9 (171·4 to 473·1)	268·9 (179·1 to 384·4)	235·6 (172·4 to 307·3)	–19·3 (−37·5 to 5·5)	–12·4 (−22·8 to −0·2)*
Skin and subcutaneous diseases				41 366·2 (28 152·7 to 59 346·6)	51 550·5 (35 306·4 to 73 530·7)	57 394·0 (39 334·2 to 81 653·4)	38·8 (37·0 to 41·0)*	11·3 (10·6 to 12·4)*	756·7 (518·9 to 1081·9)	770·3 (529·3 to 1097·1)	781·3 (535·8 to 1110·4)	3·2 (2·5 to 4·4)*	1·4 (0·8 to 2·2)*
		Dermatitis		8427·2 (5021·7 to 13 797·8)	10 043·3 (6010·8 to 16 327·0)	11 210·2 (6714·5 to 18 218·1)	33·0 (31·2 to 35·0)*	11·6 (10·8 to 12·4)*	151·8 (91·0 to 246·4)	151·3 (90·6 to 245·3)	153·0 (91·6 to 248·4)	0·7 (−0·3 to 1·8)	1·1 (0·2 to 1·8)*
		Psoriasis		3321·0 (2384·1 to 4347·4)	4638·9 (3323·7 to 6084·4)	5643·4 (4039·7 to 7377·2)	69·9 (68·4 to 71·5)*	21·7 (20·8 to 22·6)*	69·7 (50·0 to 91·2)	73·5 (52·6 to 96·1)	76·6 (54·9 to 100·0)	9·8 (9·1 to 10·6)*	4·2 (3·5 to 5·0)*
		Cellulitis		254·1 (179·0 to 320·6)	451·3 (288·9 to 558·2)	607·6 (398·4 to 739·6)	139·1 (103·8 to 168·2)*	34·6 (25·1 to 48·9)*	5·5 (3·9 to 6·9)	7·6 (4·8 to 9·4)	8·6 (5·6 to 10·4)	56·3 (32·3 to 74·2)*	13·1 (5·5 to 24·8)*
		Pyoderma		1094·9 (615·2 to 1411·5)	1577·2 (930·0 to 1948·5)	1944·8 (1249·8 to 2603·1)	77·6 (42·6 to 123·0)*	23·3 (7·9 to 40·5)*	22·1 (12·9 to 27·9)	26·0 (15·4 to 32·2)	27·7 (17·8 to 37·0)	25·1 (3·6 to 51·2)*	6·5 (−6·4 to 20·8)
		Scabies		3332·5 (1844·4 to 5364·2)	3670·6 (2034·7 to 5849·4)	3787·8 (2103·6 to 6029·0)	13·7 (10·8 to 16·7)*	3·2 (1·8 to 4·6)*	58·6 (32·4 to 93·0)	53·9 (29·8 to 85·6)	51·0 (28·3 to 81·0)	–13·0 (−13·9 to −12·2)*	–5·4 (−6·2 to −4·8)*
		Fungal skin diseases		2267·4 (900·2 to 4720·9)	2973·4 (1184·9 to 6172·3)	3508·8 (1403·0 to 7271·0)	54·8 (51·8 to 57·7)*	18·0 (17·0 to 19·1)*	46·1 (18·3 to 95·7)	47·7 (18·9 to 98·8)	48·9 (19·5 to 101·4)	6·0 (5·3 to 6·8)*	2·5 (2·2 to 2·9)*
		Viral skin diseases		4543·4 (2823·8 to 6780·1)	5425·8 (3369·5 to 8082·0)	5915·2 (3674·3 to 8828·1)	30·2 (29·2 to 31·2)*	9·0 (8·6 to 9·5)*	80·2 (49·9 to 119·5)	80·0 (49·8 to 119·1)	79·9 (49·6 to 119·3)	–0·4 (−0·8 to 0·0)	–0·1 (−0·5 to 0·2)
		Acne vulgaris		12 086·8 (8150·7 to 17 552·7)	15 067·6 (10 169·9 to 21 752·0)	15 836·0 (10 643·5 to 22 842·6)	31·0 (29·7 to 32·4)*	5·1 (4·3 to 5·8)*	202·3 (136·5 to 292·9)	207·8 (140·1 to 300·1)	212·1 (143·0 to 306·4)	4·9 (4·2 to 5·6)*	2·1 (1·5 to 2·6)*
		Alopecia areata		350·9 (224·8 to 530·5)	447·0 (286·2 to 675·8)	504·2 (322·7 to 760·0)	43·7 (42·1 to 45·3)*	12·8 (11·8 to 13·9)*	6·9 (4·4 to 10·4)	6·8 (4·3 to 10·2)	6·7 (4·3 to 10·1)	–3·5 (−4·4 to −2·6)*	–1·3 (−2·2 to −0·5)*
		Pruritus		452·1 (211·1 to 825·5)	599·8 (278·6 to 1087·3)	709·1 (329·7 to 1298·7)	56·8 (52·5 to 61·0)*	18·2 (16·7 to 19·8)*	9·4 (4·4 to 17·2)	9·6 (4·5 to 17·5)	9·7 (4·5 to 17·8)	2·9 (2·4 to 3·5)*	1·3 (0·8 to 1·8)*
		Urticaria		3155·2 (2020·3 to 4556·7)	3684·4 (2348·7 to 5265·8)	4029·9 (2575·9 to 5745·3)	27·7 (24·9 to 31·0)*	9·4 (8·3 to 10·5)*	55·1 (35·3 to 78·7)	55·0 (35·1 to 78·7)	54·9 (35·1 to 78·6)	–0·5 (−1·1 to 0·1)	–0·3 (−0·8 to 0·3)
		Decubitus ulcer		377·1 (291·5 to 475·3)	553·3 (411·4 to 680·3)	670·4 (513·0 to 836·1)	77·8 (65·5 to 86·8)*	21·2 (17·1 to 27·4)*	10·7 (8·2 to 13·6)	10·8 (8·0 to 13·3)	10·2 (7·8 to 12·6)	–5·1 (−11·6 to 0·4)	–5·7 (−9·0 to −0·4)*
		Other skin and subcutaneous diseases		1703·5 (851·6 to 3091·8)	2417·9 (1203·9 to 4377·1)	3026·6 (1513·0 to 5474·6)	77·7 (76·2 to 79·3)*	25·2 (24·5 to 25·9)*	38·1 (19·1 to 69·0)	40·4 (20·2 to 72·9)	42·2 (21·1 to 76·3)	10·8 (10·1 to 11·7)*	4·5 (4·1 to 5·1)*
	Sense organ diseases			39 443·2 (27 342·1 to 54 552·7)	54 826·6 (38 158·5 to 76 040·8)	66 701·9 (46 534·4 to 92 391·9)	69·1 (67·3 to 71·0)*	21·7 (20·7 to 22·5)*	977·4 (686·1 to 1356·7)	977·9 (685·8 to 1355·9)	959·3 (670·2 to 1331·0)	–1·9 (−2·5 to −1·2)*	–1·9 (−2·6 to −1·3)*
		Glaucoma		211·5 (142·7 to 292·9)	339·7 (230·3 to 471·3)	461·1 (311·4 to 642·1)	118·0 (114·5 to 121·8)*	35·7 (33·7 to 38·0)*	6·3 (4·3 to 8·7)	6·9 (4·7 to 9·5)	7·0 (4·8 to 9·8)	12·1 (10·4 to 14·0)*	2·1 (0·8 to 3·6)*
		Cataract		2698·3 (1924·0 to 3682·2)	4418·1 (3157·8 to 5992·5)	5789·0 (4134·6 to 7915·3)	114·5 (111·4 to 117·9)*	31·0 (29·4 to 32·6)*	80·1 (57·2 to 108·9)	88·3 (63·4 to 119·6)	88·3 (63·3 to 120·5)	10·3 (9·0 to 11·7)*	0·0 (−1·1 to 1·1)
		Macular degeneration		174·5 (118·9 to 238·7)	297·1 (202·1 to 406·9)	408·6 (277·9 to 555·6)	134·1 (129·3 to 139·0)*	37·5 (34·7 to 40·4)*	5·3 (3·6 to 7·3)	6·1 (4·2 to 8·4)	6·3 (4·3 to 8·6)	17·8 (15·3 to 20·4)*	2·7 (0·7 to 4·7)*
		Refraction and accommodation disorders		10 172·5 (6357·4 to 15 852·4)	13 035·5 (8163·5 to 20 302·0)	14 972·5 (9340·6 to 23 361·5)	47·2 (45·2 to 49·0)*	14·9 (13·7 to 15·9)*	230·6 (144·4 to 359·2)	220·0 (137·9 to 342·1)	209·1 (130·4 to 326·0)	–9·3 (−10·0 to −8·7)*	–5·0 (−5·8 to −4·3)*
		Age-related and other hearing loss		21 193·7 (14 943·8 to 29 581·7)	29 673·0 (20 837·2 to 41 494·7)	36 287·5 (25 341·8 to 50 893·6)	71·2 (67·0 to 75·3)*	22·3 (20·4 to 24·0)*	534·7 (379·0 to 745·0)	533·4 (375·3 to 743·4)	524·3 (368·0 to 734·0)	–1·9 (−3·5 to −0·7)*	–1·7 (−2·9 to −0·6)*
		Other vision loss		1205·2 (853·2 to 1628·8)	1776·9 (1256·9 to 2396·0)	2241·2 (1578·3 to 3016·4)	86·0 (80·7 to 91·0)*	26·1 (23·9 to 28·1)*	30·1 (21·3 to 40·6)	31·8 (22·4 to 42·9)	32·0 (22·5 to 43·1)	6·3 (4·6 to 8·0)*	0·7 (−0·8 to 2·0)
		Other sense organ diseases		3787·5 (2395·1 to 5704·0)	5286·3 (3336·8 to 7935·4)	6542·0 (4132·0 to 9828·4)	72·7 (71·6 to 73·9)*	23·8 (23·1 to 24·4)*	90·3 (57·2 to 136·3)	91·4 (57·9 to 137·9)	92·3 (58·4 to 139·0)	2·1 (1·7 to 2·5)*	0·9 (0·6 to 1·3)*
	Oral disorders			11 294·7 (6848·2 to 17 556·0)	15 552·8 (9435·9 to 24 158·8)	19 006·2 (11 586·8 to 29 629·5)	68·3 (65·8 to 70·6)*	22·2 (21·2 to 23·3)*	270·1 (165·4 to 420·1)	266·3 (162·9 to 413·0)	265·7 (162·8 to 413·4)	–1·6 (−2·3 to −0·9)*	–0·2 (−0·7 to 0·2)
		Caries of deciduous teeth		120·6 (53·2 to 233·0)	119·1 (52·5 to 231·1)	127·2 (55·9 to 249·1)	5·5 (2·8 to 7·3)*	6·8 (4·5 to 8·5)*	1·8 (0·8 to 3·5)	1·8 (0·8 to 3·4)	1·8 (0·8 to 3·4)	–3·7 (−5·9 to −2·1)*	0·2 (−1·9 to 1·8)
		Caries of permanent teeth		1295·7 (581·5 to 2521·8)	1571·3 (699·0 to 3047·3)	1707·6 (760·1 to 3324·9)	31·8 (29·7 to 33·9)*	8·7 (8·0 to 9·4)*	25·7 (11·4 to 50·0)	24·0 (10·7 to 46·5)	23·0 (10·2 to 44·6)	–10·6 (−11·9 to −9·3)*	–4·2 (−4·8 to −3·6)*
		Periodontal diseases		2676·0 (1065·8 to 5539·9)	3893·0 (1550·5 to 8078·4)	4898·0 (1946·8 to 10 208·7)	83·0 (80·9 to 84·8)*	25·8 (24·7 to 26·8)*	64·2 (25·6 to 134·6)	65·1 (25·9 to 136·6)	66·6 (26·6 to 139·4)	3·8 (3·0 to 4·4)*	2·3 (1·8 to 2·9)*
		Edentulism and severe tooth loss		4603·5 (3028·6 to 6528·4)	6553·4 (4335·5 to 9234·5)	8338·4 (5467·4 to 11 760·4)	81·1 (79·7 to 82·5)*	27·2 (26·0 to 28·4)*	125·7 (82·1 to 177·4)	122·8 (80·3 to 172·5)	121·7 (79·6 to 171·1)	–3·2 (−3·6 to −2·8)*	–0·9 (−1·6 to −0·3)*
		Other oral disorders		2598·9 (1602·8 to 3894·8)	3416·0 (2109·2 to 5128·9)	3935·0 (2427·2 to 5907·9)	51·4 (50·0 to 52·8)	15·2 (14·6 to 15·8)	52·7 (32·6 to 79·0)	52·7 (32·5 to 79·2)	52·7 (32·5 to 79·2)	–0·0 (−0·4 to 0·3)	0·0 (−0·3 to 0·3)
	Sudden infant death syndrome			4127·1 (3107·6 to 6179·4)	3031·0 (2487·9 to 3993·4)	2504·2 (2015·2 to 3003·1)	–39·3 (−57·8 to −20·1)*	–17·4 (−33·2 to −1·1)*	60·8 (45·8 to 91·0)	44·4 (36·4 to 58·4)	36·4 (29·3 to 43·6)	–40·1 (−58·4 to −21·2)*	–18·0 (−33·7 to −1·9)*
**Injuries**				**259 714·5 (242 647·1 to 276 817·9)**	**259 792·1 (242 234·6 to 280 976·8)**	**255 434·3 (236 089·5 to 280 689·1)**	**–1·6 (−6·2 to 3·8)**	**–1·7 (−4·7 to 1·4)**	**4875·4 (4544·8 to 5239·0)**	**3976·8 (3692·0 to 4319·2)**	**3457·3 (3192·1 to 3803·2)**	–29·1 (−32·0 to −25·9)*	–13·1 (−15·6 to −10·6)*
	**Transport injuries**			**70 430·3 (67 012·4 to 74 545·4)**	**80 802·1 (76 835·3 to 85 315·6)**	**78 051·8 (73 391·8 to 83 700·8)**	10·8 (6·4 to 15·9)*	–3·4 (−5·8 to −0·9)*	**1320·1 (1253·5 to 1399·1)**	**1218·5 (1155·7 to 1292·0)**	**1044·0 (980·2 to 1120·6)**	–20·9 (−23·9 to −17·8)*	–14·3 (−16·4 to −12·3)*
	Road injuries			64 788·4 (61 744·3 to 68 397·9)	74 299·1 (71 021·9 to 78 088·0)	71 395·0 (67 520·8 to 76 128·0)	10·2 (5·5 to 15·2)*	–3·9 (−6·3 to −1·6)*	1210·6 (1151·7 to 1279·9)	1119·1 (1066·5 to 1179·6)	954·5 (901·8 to 1019·0)	–21·1 (−24·1 to −18·0)*	–14·7 (−16·8 to −12·8)*
		Pedestrian road injuries		24 717·6 (22 795·5 to 28 090·8)	26 131·7 (24 588·0 to 28 158·5)	24 001·5 (22 518·2 to 25 750·8)	–2·9 (−15·1 to 4·8)	–8·2 (−13·1 to −5·0)*	459·3 (424·8 to 520·6)	398·7 (375·0 to 429·0)	323·3 (303·6 to 346·5)	–29·6 (−38·3 to −24·6)*	–18·9 (−23·2 to −16·2)*
		Cyclist road injuries		3390·5 (2919·1 to 3889·2)	4747·7 (4149·8 to 5488·4)	4968·2 (4229·6 to 5946·9)	46·5 (30·4 to 67·8)*	4·6 (−0·2 to 10·0)	68·7 (58·8 to 79·8)	74·3 (64·8 to 86·2)	67·2 (57·1 to 80·7)	–2·2 (−12·4 to 11·4)	–9·5 (−13·4 to −4·7)*
		Motorcyclist road injuries		11 467·4 (10 510·3 to 12 944·1)	15 105·0 (13 758·6 to 16 396·3)	14 937·5 (13 499·8 to 16 289·9)	30·3 (18·2 to 40·1)*	–1·1 (−5·4 to 2·6)	211·8 (194·4 to 239·6)	222·6 (202·8 to 242·2)	197·3 (178·6 to 215·4)	–6·8 (−15·5 to 0·2)	–11·3 (−15·1 to −8·2)*
		Motor vehicle road injuries		24 319·9 (20 644·9 to 27 442·2)	27 217·7 (24 983·6 to 30 653·8)	26 211·8 (24 325·0 to 29 404·9)	7·8 (0·7 to 25·4)*	–3·7 (−7·2 to 2·4)	452·8 (386·1 to 509·4)	406·3 (372·9 to 455·6)	349·4 (324·1 to 391·5)	–22·9 (−27·6 to −11·0)*	–14·0 (−17·0 to −8·9)*
		Other road injuries		893·0 (714·6 to 1103·6)	1097·0 (919·3 to 1343·6)	1275·9 (1026·6 to 1626·3)	42·9 (23·2 to 73·5)*	16·3 (9·2 to 23·7)*	17·9 (14·3 to 22·2)	17·2 (14·3 to 21·1)	17·3 (13·9 to 22·1)	–3·3 (−15·0 to 15·3)	0·8 (−4·9 to 6·7)
	Other transport injuries			5641·9 (4761·1 to 6609·6)	6503·0 (5751·7 to 7477·8)	6656·8 (5797·7 to 7707·3)	18·0 (5·2 to 38·4)*	2·4 (−3·0 to 8·9)	109·5 (92·6 to 128·2)	99·4 (87·3 to 114·9)	89·5 (77·8 to 103·9)	–18·3 (−26·4 to −5·1)*	–10·0 (−14·5 to −4·4)*
**Unintentional injuries**				**121 695·5 (109 006·9 to 134 119·5)**	**111 226·3 (98 814·1 to 125 226·5)**	**107 423·5 (94 550·6 to 124 015·0)**	–11·7 (−18·4 to −4·3)*	**–3·4 (−7·1 to 0·1)**	**2282·2 (2056·3 to 2530·5)**	**1750·0 (1549·2 to 1975·9)**	**1486·2 (1306·5 to 1716·5)**	–34·9 (−39·0 to −30·6)*	–15·1 (−18·0 to −12·2)*
Falls				26 607·6 (22 592·9 to 31 276·8)	31 258·7 (26 220·2 to 37 276·0)	35 773·8 (29 777·6 to 43 175·7)	34·5 (22·3 to 44·8)*	14·4 (9·0 to 18·1)*	589·1 (498·2 to 700·1)	530·8 (444·1 to 630·7)	506·5 (422·1 to 610·0)	–14·0 (−20·6 to −9·2)*	–4·6 (−8·8 to −1·8)*
Drowning				34 779·0 (29 934·2 to 37 903·4)	22 886·7 (20 014·2 to 24 143·1)	16 809·0 (15 206·1 to 18 047·4)	–51·7 (−55·6 to −43·2)*	–26·6 (−30·1 to −20·1)*	567·0 (491·4 to 614·9)	337·6 (295·4 to 356·2)	229·2 (207·1 to 246·3)	–59·6 (−62·8 to −53·0)*	–32·1 (−35·4 to −26·1)*
Fire, heat, and hot substances				10 059·5 (7875·0 to 11 492·7)	8822·8 (7294·7 to 10 004·2)	8150·8 (6763·6 to 9476·2)	–19·0 (−28·2 to −5·5)*	–7·6 (−13·1 to −0·1)*	186·1 (150·6 to 212·3)	135·9 (113·3 to 154·4)	111·4 (92·4 to 129·4)	–40·2 (−46·0 to −31·7)*	–18·1 (−22·7 to −11·8)*
Poisonings				5471·1 (3951·1 to 6579·7)	3747·9 (2956·8 to 4213·0)	3149·4 (2379·7 to 3574·1)	–42·4 (−53·8 to −21·9)*	–16·0 (−25·9 to −2·9)*	95·1 (69·4 to 112·2)	56·3 (44·5 to 63·3)	43·0 (32·5 to 48·9)	–54·8 (−63·8 to −39·4)*	–23·6 (−32·8 to −11·8)*
Exposure to mechanical forces				12 931·3 (11 440·5 to 14 960·9)	12 360·8 (10 580·5 to 14 181·8)	11 921·2 (9836·7 to 14 174·8)	–7·8 (−25·2 to 2·4)	–3·6 (−8·0 to 1·0)	239·6 (211·3 to 277·2)	189·7 (162·2 to 219·0)	162·2 (133·8 to 193·1)	–32·3 (−44·1 to −26·6)*	–14·4 (−18·0 to −11·0)*
	Unintentional firearm injuries			1681·2 (1345·7 to 1839·8)	1415·5 (1182·3 to 1575·2)	1351·5 (1104·3 to 1513·3)	–19·6 (−26·2 to −12·4)*	–4·5 (−10·9 to 0·7)	30·9 (24·8 to 34·1)	21·1 (17·6 to 23·5)	18·1 (14·8 to 20·3)	–41·5 (−46·1 to −36·4)*	–14·3 (−19·9 to −9·9)*
	Unintentional suffocation			3239·4 (2677·8 to 4135·3)	2101·3 (1713·6 to 2425·9)	1757·1 (1410·8 to 2041·2)	–45·8 (−59·1 to −34·4)*	–16·4 (−23·6 to −8·8)*	50·6 (42·1 to 63·9)	31·2 (25·4 to 36·0)	24·7 (19·8 to 28·7)	–51·2 (−63·0 to −41·5)*	–20·8 (−27·6 to −13·7)*
	Other exposure to mechanical forces			8010·6 (6937·0 to 9470·1)	8844·1 (7355·1 to 10 402·4)	8812·6 (7062·9 to 10 749·0)	10·0 (−10·2 to 20·3)	–0·4 (−5·0 to 4·2)	158·1 (135·5 to 187·8)	137·4 (113·9 to 162·8)	119·5 (96·0 to 145·9)	–24·4 (−37·5 to −19·1)*	–13·1 (−16·6 to −9·6)*
Adverse effects of medical treatment				5352·0 (3741·7 to 6317·4)	5077·1 (4014·2 to 5746·0)	4991·7 (4229·9 to 5575·1)	–6·7 (−17·7 to 15·4)	–1·7 (−8·0 to 7·5)	99·5 (73·7 to 114·5)	80·2 (64·1 to 90·4)	69·7 (59·2 to 77·9)	–30·0 (−36·6 to −18·4)*	–13·2 (−18·2 to −6·2)*
Animal contact				6346·7 (4715·4 to 7403·2)	6241·9 (4797·7 to 7155·5)	5936·0 (4650·5 to 6820·3)	–6·5 (−17·4 to 13·4)	–4·9 (−10·6 to 3·5)	118·2 (89·0 to 138·0)	95·1 (73·2 to 109·3)	80·7 (63·2 to 92·7)	–31·7 (−38·8 to −18·1)*	–15·1 (−20·1 to −7·7)*
	Venomous animal contact			5129·9 (3648·8 to 6063·6)	5170·5 (3898·3 to 5979·9)	4865·9 (3689·6 to 5640·6)	–5·2 (−16·8 to 17·5)	–5·9 (−12·1 to 3·0)	94·9 (68·3 to 111·9)	78·4 (59·1 to 90·7)	66·0 (50·0 to 76·6)	–30·4 (−37·9 to −14·7)*	–15·7 (−21·1 to −7·9)*
	Non-venomous animal contact			1216·9 (949·1 to 1693·5)	1071·3 (835·4 to 1440·4)	1070·1 (837·8 to 1396·1)	–12·1 (−25·2 to −0·4)*	–0·1 (−7·7 to 7·1)	23·3 (18·2 to 31·6)	16·7 (13·0 to 22·4)	14·7 (11·5 to 19·1)	–37·1 (−44·5 to −30·2)*	–12·3 (−18·3 to −6·4)*
Foreign body				7078·1 (5242·3 to 9108·8)	6599·7 (5467·6 to 7941·9)	6742·8 (5709·7 to 7921·3)	–4·7 (−20·0 to 18·0)	2·2 (−6·6 to 11·3)	127·2 (98·0 to 159·1)	102·2 (84·9 to 122·5)	94·0 (79·8 to 110·3)	–26·1 (−35·1 to −12·1)*	–8·0 (−15·0 to −0·3)*
	Pulmonary aspiration and foreign body in airway			5386·2 (4058·7 to 7061·1)	5309·7 (4303·9 to 6377·5)	5327·3 (4587·3 to 6136·5)	–1·1 (−17·2 to 20·3)	0·3 (−8·8 to 11·6)	96·0 (74·8 to 121·0)	82·1 (66·9 to 98·0)	74·6 (64·3 to 86·0)	–22·3 (−31·9 to −8·3)*	–9·1 (−16·7 to 0·6)
	Foreign body in eyes			124·2 (63·7 to 213·3)	146·6 (78·2 to 243·6)	185·8 (100·2 to 310·1)	49·6 (41·7 to 57·3)*	26·8 (24·1 to 30·1)*	2·6 (1·4 to 4·3)	2·4 (1·3 to 3·9)	2·5 (1·4 to 4·2)	–2·5 (−4·3 to −0·8)*	8·2 (6·3 to 10·3)*
	Foreign body in other body part			1567·7 (948·1 to 2028·4)	1143·5 (869·3 to 1474·9)	1229·7 (929·1 to 1602·4)	–21·6 (−36·7 to 11·6)	7·5 (0·6 to 13·4)*	28·6 (18·2 to 36·8)	17·7 (13·4 to 22·9)	16·8 (12·7 to 21·9)	–41·3 (−50·8 to −21·5)*	–5·2 (−10·6 to −0·8)*
Environmental heat and cold exposure				4613·3 (3492·7 to 5757·7)	4712·3 (3611·7 to 5881·4)	4676·7 (3520·0 to 5993·7)	1·4 (−9·2 to 11·5)	–0·8 (−7·9 to 6·3)	96·6 (74·1 to 121·0)	75·7 (58·1 to 94·5)	64·1 (48·3 to 82·0)	–33·6 (−40·8 to −27·6)*	–15·3 (−21·8 to −9·7)*
Other unintentional injuries				8456·9 (7319·9 to 10 038·8)	9518·6 (8199·6 to 11 247·0)	9272·1 (7666·7 to 11 405·2)	9·6 (−6·2 to 20·4)	–2·6 (−7·2 to 1·8)	163·8 (140·6 to 195·7)	146·4 (124·8 to 174·5)	125·5 (103·7 to 154·8)	–23·4 (−33·6 to −17·0)*	–14·3 (−18·0 to −10·8)*
**Self-harm and interpersonal violence**				**57 011·9 (52 622·2 to 60 351·8)**	**61 630·8 (56 736·9 to 64 509·4)**	**58 717·9 (53 866·4 to 62 809·6)**	**3·0 (−3·5 to 11·0)**	–4·7 (−8·4 to −0·1)*	**1091·2 (1007·5 to 1153·3)**	**919·1 (845·6 to 962·8)**	**776·8 (712·6 to 830·8)**	–28·8 (−33·2 to −23·4)*	–15·5 (−18·7 to −11·4)*
	Self-harm			36 069·6 (33 180·8 to 38 321·5)	37 517·7 (34 901·0 to 39 462·7)	35 149·6 (32 845·1 to 37 938·3)	–2·5 (−8·5 to 6·3)	–6·3 (−10·4 to −0·9)*	705·3 (650·2 to 753·4)	565·6 (526·2 to 594·0)	465·7 (434·7 to 502·5)	–34·0 (−37·9 to −28·2)*	–17·7 (−21·2 to −12·9)*
		Self-harm by firearm		2951·5 (2377·2 to 3829·1)	2875·2 (2348·6 to 3710·6)	2853·2 (2386·0 to 3589·2)	–3·3 (−13·1 to 13·8)	–0·8 (−7·5 to 9·7)	57·9 (47·4 to 75·0)	43·2 (35·6 to 55·7)	37·8 (31·6 to 47·6)	–34·8 (−41·0 to −24·0)*	–12·6 (−18·4 to −3·9)*
		Self-harm by other specified means		33 118·1 (30 211·8 to 35 109·1)	34 642·5 (32 074·6 to 36 526·4)	32 296·4 (30 216·7 to 34 924·8)	–2·5 (−8·5 to 6·3)	–6·8 (−11·0 to −1·3)*	647·4 (590·9 to 686·4)	522·4 (481·9 to 549·9)	427·9 (399·9 to 462·6)	–33·9 (−37·9 to −28·1)*	–18·1 (−21·8 to −13·3)*
	Interpersonal violence			20 942·3 (17 360·5 to 23 608·2)	24 113·2 (19 792·0 to 26 579·9)	23 568·3 (19 646·6 to 26 479·2)	12·5 (3·6 to 22·4)*	–2·3 (−6·9 to 3·5)	385·9 (318·6 to 436·9)	353·5 (290·4 to 391·3)	311·1 (259·7 to 349·8)	–19·4 (−25·8 to −12·5)*	–12·0 (−16·1 to −7·0)*
		Physical violence by firearm		6529·3 (4689·4 to 7918·7)	8381·5 (5616·3 to 9307·9)	8720·1 (5844·7 to 9853·4)	33·5 (15·0 to 48·5)*	4·0 (−0·7 to 9·0)	119·1 (84·7 to 144·7)	120·5 (80·8 to 133·8)	114·3 (76·6 to 129·1)	–4·1 (−17·5 to 6·7)	–5·2 (−9·6 to −0·6)*
		Physical violence by sharp object		5070·8 (3833·9 to 6262·5)	5886·3 (4626·0 to 7279·7)	5288·2 (4290·5 to 6922·3)	4·3 (−8·9 to 25·1)	–10·2 (−16·5 to −0·5)*	94·5 (71·8 to 117·5)	86·4 (67·6 to 106·7)	69·5 (56·4 to 90·9)	–26·5 (−36·0 to −12·1)*	–19·6 (−25·1 to −10·7)*
		Sexual violence		1162·6 (774·9 to 1647·4)	1319·4 (884·1 to 1879·0)	1365·8 (917·1 to 1946·0)	17·5 (14·4 to 20·6)*	3·5 (2·2 to 4·8)*	21·2 (14·2 to 30·0)	19·1 (12·9 to 27·3)	18·1 (12·2 to 25·8)	–14·5 (−15·6 to −13·3)*	–5·5 (−6·2 to −4·9)*
		Physical violence by other means		8179·6 (6388·8 to 9519·2)	8526·0 (7045·4 to 10 132·6)	8194·1 (7011·8 to 10 058·4)	0·2 (−15·5 to 20·3)	–3·9 (−12·3 to 4·9)	151·1 (120·0 to 176·2)	127·5 (105·6 to 151·6)	109·3 (93·5 to 133·9)	–27·7 (−38·2 to −14·0)*	–14·3 (−21·6 to −6·5)*
**Forces of nature, conflict and terrorism, and executions and police conflict**				**10 576·7 (8362·5 to 12 866·6)**	**6132·8 (4005·8 to 8352·3)**	**11 241·1 (8003·1 to 14 753·8)**	**6·3 (−25·0 to 46·5)**	83·3 (24·2 to 183·3)*	**181·9 (143·2 to 221·0)**	**89·3 (58·2 to 121·9)**	**150·3 (107·0 to 197·3)**	**–17·4 (−41·4 to 14·1)**	68·3 (14·3 to 159·6)*
	Exposure to forces of nature			3090·2 (1185·9 to 4943·3)	946·7 (636·1 to 1276·6)	617·1 (369·5 to 992·6)	–80·0 (−90·2 to −49·1)*	–34·8 (−50·5 to −15·8)*	53·5 (20·8 to 85·5)	14·0 (9·4 to 19·0)	8·3 (5·0 to 13·4)	–84·5 (−92·3 to −60·9)*	–40·9 (−54·9 to −24·4)*
	Conflict and terrorism			6889·0 (5852·1 to 7909·2)	4824·1 (2855·5 to 6799·0)	10 326·0 (7176·9 to 13 627·5)	49·9 (5·2 to 100·3)*	114·0 (36·2 to 271·3)*	117·4 (99·2 to 135·3)	69·9 (41·6 to 98·8)	138·1 (95·9 to 182·2)	17·6 (−17·4 to 57·1)	97·4 (25·7 to 240·9)*
	Executions and police conflict			733·5 (292·1 to 958·6)	418·5 (263·0 to 654·0)	355·7 (221·1 to 576·5)	–51·5 (−65·3 to −12·8)*	–15·0 (−22·4 to −7·5)*	13·4 (5·4 to 17·6)	6·2 (3·9 to 9·9)	4·7 (2·9 to 7·7)	–64·8 (−74·6 to −37·4)*	–24·2 (−30·2 to −17·8)*

Data in parentheses are 95% uncertainty intervals. To download the data in this table, please visit the Global Health Data Exchange. ··=not defined (used for asymptomatic causes, epidemics, and outbreaks). DALYs=disability-adjusted life-years. G6PD=glucose-6-phosphate dehydrogenase.

* Percentage changes that are statistically significant.

From 1990 to 2016, global HALE at birth increased from 56·9 years to 63·1 years, with 160 of 195 locations registering significant improvements. Global HALE increased by an average of 6·24 years (95% UI 5·97–6·48) for both sexes combined. Globally, HALE at birth increased from 55·38 years (53·27–57·31) in 1990 to 61·42 years (59·01–63·58) in 2016 for males and from 58·42 years (55·80–60·77) to 64·91 years (61·88–67·54) for females, rising 6·04 years (5·74–6·27) for males and 6·49 years (6·08–6·77) for females (Table 2, Table 3). The total number of years of functional health lost (life expectancy minus HALE) increased from 1990 to 2016, from 8·22 years to 9·34 years. The gap between life expectancy at birth and HALE, which represents years of functional health lost, grew between 1990 and 2016 from 7·32 years (life expectancy 62·70 [62·42–62·99] *vs* HALE 55·38 [53·27–57·31]) to 8·37 years (69·79 [69·29–70·22] *vs* 61·42 [59·01–63·58]) for males and from 9·15 years (67·57 [67·33–67·77] *vs* 58·42 [55·80–60·77]) to 10·42 years (75·33 [74·95–75·64] *vs* 64·91 [61·88–67·54]) for females. Globally, in 2016, life expectancy at age 65 years was 18·57 years (18·37–18·72) for females and 15·72 years (15·61–15·83) for males, whereas HALE was 13·88 years (12·57–15·02) for females and 11·87 years (10·83–12·80) for males. HALE increased by 1·96 years (1·69–2·13) from 11·92 (10·88–12·89) in 1990 for females and by 1·78 years (1·61–1·93) from 10·09 years (9·22–10·87) for males.

**Table 2:** Global, regional, and GBD location-specific life expectancy and HALE at birth, by sex, in 1990, 2006, and 2016

			**1990, at birth**				**2006, at birth**				**2016, at birth**			
			Females		Males		Females		Males		Females		Males	
			Life expectancy	HALE	Life expectancy	HALE	Life expectancy	HALE	Life expectancy	HALE	Life expectancy	HALE	Life expectancy	HALE
**Global**			**67·57 (67·33–67·77)**	**58·42 (55·80–60·77)**	**62·70 (62·42–62·99)**	**55·38 (53·27–57·31)**	**71·44 (71·25–71·65)**	**61·73 (58·91–64·21)**	**66·35 (66·05–66·63)**	**58·51 (56·27–60·55)**	**75·33 (74·95–75·64)**	**64·91 (61·88–67·54)**	**69·79 (69·29–70·22)**	**61·42 (59·01–63·58)**
	High SDI		79·15 (79·05–79·24)	68·23 (65·08–71·02)	72·32 (72·21–72·44)	63·82 (61·37–66·00)	82·13 (82·06–82·21)	70·61 (67·28–73·57)	76·32 (76·23–76·41)	66·98 (64·32–69·32)	83·37 (83·18–83·57)	71·49 (68·03–74·53)	78·06 (77·81–78·29)	68·33 (65·60–70·82)
	High-middle SDI		73·54 (73·16–73·92)	63·64 (60·78–66·23)	66·44 (65·99–66·90)	58·73 (56·43–60·76)	76·76 (76·39–77·11)	66·53 (63·49–69·12)	69·36 (68·83–69·82)	61·45 (59·13–63·51)	79·86 (78·65–80·80)	68·96 (65·69–71·84)	73·12 (72·09–74·05)	64·57 (62·03–67·04)
	Middle SDI		68·75 (68·41–69·09)	60·16 (57·68–62·37)	64·15 (63·79–64·50)	57·36 (55·31–59·11)	73·67 (73·45–73·88)	64·32 (61·62–66·73)	68·32 (68·06–68·58)	60·95 (58·80–62·90)	77·28 (76·99–77·57)	67·19 (64·26–69·70)	71·06 (70·68–71·40)	63·21 (60·91–65·16)
	Low-middle SDI		60·39 (59·94–60·81)	51·69 (49·11–53·94)	58·41 (57·92–58·90)	51·03 (48·92–52·98)	65·55 (65·17–65·94)	56·12 (53·36–58·47)	62·39 (61·93–62·85)	54·51 (52·29–56·57)	70·26 (69·74–70·74)	60·11 (57·16–62·70)	66·25 (65·64–66·79)	57·90 (55·43–60·03)
	Low SDI		53·71 (53·24–54·20)	46·18 (43·95–48·11)	51·14 (50·52–51·75)	44·45 (42·48–46·30)	57·62 (57·12–58·15)	49·75 (47·41–51·86)	55·98 (55·32–56·59)	48·81 (46·70–50·76)	64·10 (63·33–64·82)	55·44 (52·83–57·79)	61·63 (60·68–62·52)	53·86 (51·50–56·11)
	**High income**		**79·34 (79·24–79·43)**	**68·40 (65·23–71·19)**	**72·64 (72·54–72·75)**	**64·15 (61·70–66·33)**	**82·31 (82·23–82·38)**	**70·76 (67·43–73·73)**	**76·62 (76·52–76·70)**	**67·29 (64·63–69·64)**	**83·48 (83·28–83·67)**	**71·61 (68·12–74·66)**	**78·27 (78·04–78·49)**	**68·58 (65·82–71·06)**
High-income North America			79·04 (78·94–79·13)	67·67 (64·44–70·61)	72·15 (72·03–72·26)	63·25 (60·75–65·49)	80·67 (80·58–80·76)	68·83 (65·45–71·90)	75·63 (75·54–75·74)	65·87 (63·11–68·29)	81·50 (81·28–81·72)	69·34 (65·86–72·43)	76·79 (76·54–77·04)	66·71 (63·87–69·21)
	Canada		80·64 (80·33–80·93)	69·83 (66·72–72·67)	74·23 (73·91–74·55)	65·75 (63·26–67·95)	82·75 (82·46–83·01)	71·51 (68·28–74·46)	78·10 (77·84–78·42)	68·86 (66·17–71·23)	83·89 (83·49–84·30)	72·30 (69·00–75·27)	79·76 (79·30–80·22)	70·04 (67·23–72·61)
	Greenland		66·09 (64·72–67·48)	57·28 (54·54–59·83)	57·74 (56·27–59·11)	51·39 (49·21–53·48)	68·78 (67·50–70·02)	59·52 (56·78–62·08)	65·08 (63·83–66·20)	57·69 (55·47–59·79)	72·82 (70·37–75·59)	62·90 (59·41–66·12)	67·80 (64·69–70·64)	60·03 (56·88–63·34)
	USA		78·87 (78·77–78·98)	67·45 (64·20–70·42)	71·93 (71·81–72·05)	62·99 (60·48–65·23)	80·45 (80·35–80·54)	68·54 (65·16–71·62)	75·37 (75·26–75·48)	65·54 (62·79–67·99)	81·23 (80·99–81·46)	69·01 (65·50–72·12)	76·45 (76·19–76·73)	66·34 (63·49–68·86)
	Australasia		79·85 (79·63–80·08)	68·90 (65·74–71·70)	73·76 (73·52–74·00)	64·95 (62·38–67·23)	83·32 (83·13–83·52)	71·69 (68·27–74·72)	78·76 (78·55–78·99)	69·05 (66·22–71·56)	84·39 (83·80–84·99)	72·55 (69·07–75·61)	80·32 (79·63–81·02)	70·28 (67·38–72·83)
	Australia		80·11 (79·87–80·39)	69·11 (65·92–71·94)	73·97 (73·69–74·25)	65·10 (62·51–67·40)	83·58 (83·35–83·80)	71·88 (68·45–74·96)	78·93 (78·68–79·19)	69·17 (66·31–71·70)	84·58 (83·84–85·27)	72·70 (69·18–75·75)	80·48 (79·67–81·21)	70·38 (67·51–72·95)
	New Zealand		78·55 (78·18–78·89)	67·90 (64·78–70·66)	72·73 (72·38–73·08)	64·24 (61·75–66·50)	82·05 (81·73–82·34)	70·75 (67·40–73·65)	77·93 (77·60–78·25)	68·50 (65·75–70·98)	83·40 (82·25–84·62)	71·83 (68·56–74·89)	79·55 (78·27–80·83)	69·78 (66·84–72·44)
High-income Asia Pacific			80·68 (80·38–80·95)	70·30 (67·29–73·05)	74·10 (73·68–74·48)	65·82 (63·48–67·98)	84·95 (84·70–85·18)	73·64 (70·34–76·57)	78·06 (77·75–78·37)	68·88 (66·28–71·30)	86·42 (85·62–87·09)	74·70 (71·14–77·75)	80·07 (79·10–80·94)	70·47 (67·66–73·06)
	Brunei		75·39 (73·88–76·73)	65·61 (62·70–68·40)	71·68 (70·69–72·82)	63·24 (60·71–65·65)	78·90 (77·93–79·65)	68·72 (65·74–71·51)	74·64 (73·89–75·45)	65·84 (63·47–68·18)	79·48 (77·79–81·73)	69·34 (66·13–72·26)	74·56 (72·51–77·34)	65·84 (62·82–68·69)
	Japan		81·81 (81·76–81·85)	71·31 (68·26–74·00)	75·91 (75·86–75·96)	67·45 (65·01–69·60)	85·57 (85·51–85·64)	74·20 (70·86–77·10)	78·76 (78·71–78·81)	69·51 (66·85–71·84)	86·94 (86·73–87·16)	75·10 (71·59–78·08)	80·83 (80·57–81·08)	71·11 (68·36–73·60)
	Singapore		78·10 (76·47–79·73)	68·51 (65·44–71·31)	72·86 (71·33–74·39)	65·11 (62·52–67·52)	83·71 (82·39–84·98)	73·34 (70·20–76·23)	78·55 (77·18–79·92)	69·99 (67·44–72·54)	86·08 (83·92–88·42)	75·16 (71·85–78·57)	81·26 (78·75–83·67)	72·01 (68·76–75·05)
	South Korea		76·33 (74·99–77·68)	66·56 (63·56–69·48)	67·74 (66·18–69·33)	60·30 (57·86–62·70)	82·11 (81·07–83·18)	71·18 (67·93–74·18)	75·48 (74·23–76·96)	66·63 (64·01–69·40)	84·22 (81·22–87·13)	72·97 (68·98–76·74)	77·67 (74·32–81·52)	68·49 (64·84–72·03)
Western Europe			79·50 (79·34–79·65)	68·49 (65·28–71·34)	72·94 (72·77–73·11)	64·52 (62·05–66·70)	82·79 (82·66–82·92)	71·24 (67·89–74·22)	77·24 (77·10–77·39)	68·07 (65·41–70·45)	84·10 (83·84–84·38)	72·27 (68·78–75·35)	79·21 (78·90–79·49)	69·69 (66·89–72·13)
	Andorra		82·80 (80·33–85·33)	70·60 (66·75–74·37)	76·19 (73·96–78·77)	66·81 (63·64–70·01)	85·95 (83·76–87·60)	73·11 (69·15–76·73)	79·16 (77·43–80·82)	69·25 (65·99–72·17)	85·77 (82·96–87·69)	73·03 (69·09–76·68)	79·33 (77·41–81·92)	69·42 (66·07–72·49)
	Austria		78·86 (78·61–79·12)	68·23 (65·16–71·00)	72·15 (71·87–72·41)	63·91 (61·49–66·04)	82·53 (82·30–82·75)	71·30 (68·06–74·20)	77·00 (76·74–77·24)	67·80 (65·05–70·15)	83·85 (83·20–84·52)	72·24 (68·88–75·30)	79·12 (78·50–79·87)	69·55 (66·69–72·18)
	Belgium		79·26 (78·82–79·66)	68·29 (65·15–71·13)	72·65 (72·14–73·14)	64·27 (61·68–66·46)	82·17 (81·80–82·56)	70·29 (66·89–73·26)	76·57 (76·13–77·01)	67·17 (64·45–69·56)	83·37 (82·33–84·52)	71·40 (67·92–74·49)	78·42 (77·24–79·54)	68·80 (65·77–71·49)
	Cyprus		76·97 (76·52–77·41)	66·64 (63·65–69·30)	72·89 (72·36–73·42)	64·67 (62·30–66·83)	81·02 (80·57–81·48)	69·93 (66·72–72·78)	76·30 (75·77–76·81)	67·41 (64·75–69·72)	82·82 (82·27–83·36)	71·45 (68·14–74·43)	78·12 (77·46–78·82)	68·97 (66·28–71·45)
	Denmark		77·96 (77·13–78·75)	67·14 (64·04–70·04)	72·44 (71·66–73·31)	64·02 (61·53–66·33)	80·55 (79·78–81·31)	69·19 (65·95–72·08)	76·12 (75·38–76·89)	66·84 (64·10–69·41)	82·85 (81·51–84·23)	70·83 (67·40–74·11)	78·79 (77·31–80·14)	68·92 (66·08–71·68)
	Finland		79·00 (78·66–79·33)	67·73 (64·43–70·69)	70·97 (70·60–71·34)	62·36 (59·81–64·66)	82·59 (82·27–82·88)	70·51 (66·95–73·66)	75·68 (75·31–76·06)	66·02 (63·18–68·51)	84·58 (83·85–85·41)	72·21 (68·52–75·49)	78·85 (77·95–79·76)	68·81 (65·95–71·54)
	France		81·22 (80·93–81·50)	69·93 (66·71–72·84)	73·09 (72·73–73·46)	64·82 (62·36–66·98)	84·09 (83·78–84·36)	72·51 (69·07–75·52)	77·11 (76·77–77·52)	68·28 (65·62–70·59)	85·39 (84·85–85·99)	73·42 (69·88–76·59)	79·20 (78·58–79·84)	69·93 (67·19–72·37)
	Germany		78·53 (77·95–79·07)	67·63 (64·42–70·49)	72·04 (71·38–72·70)	63·62 (61·19–65·80)	82·13 (81·67–82·62)	70·57 (67·24–73·61)	76·67 (76·12–77·27)	67·43 (64·78–69·89)	83·34 (82·47–84·29)	71·52 (68·00–74·73)	78·47 (77·44–79·52)	68·90 (66·03–71·51)
	Greece		79·54 (79·22–79·86)	68·72 (65·64–71·45)	74·48 (74·11–74·88)	66·03 (63·54–68·20)	82·64 (82·36–82·92)	71·22 (67·96–74·12)	77·11 (76·75–77·46)	68·17 (65·55–70·47)	83·50 (82·77–84·25)	72·02 (68·77–75·06)	78·43 (77·45–79·46)	69·22 (66·58–71·70)
	Iceland		80·41 (79·80–81·00)	69·17 (65·92–72·04)	75·68 (75·14–76·24)	66·75 (64·15–69·07)	83·15 (82·53–83·81)	71·45 (68·04–74·44)	79·55 (79·05–80·05)	69·93 (67·10–72·36)	83·99 (82·92–85·02)	72·17 (68·76–75·31)	80·56 (79·65–81·40)	70·79 (67·97–73·50)
	Ireland		77·64 (77·03–78·27)	67·08 (63·98–69·89)	72·18 (71·58–72·85)	63·93 (61·51–66·08)	81·54 (80·95–82·14)	70·17 (66·93–73·15)	76·97 (76·34–77·55)	67·74 (65·08–70·24)	83·33 (81·96–84·72)	71·59 (68·10–74·73)	78·97 (77·45–80·41)	69·34 (66·29–72·10)
	Israel		77·55 (76·40–78·64)	67·25 (64·05–70·06)	74·11 (73·01–75·34)	65·62 (63·00–68·01)	81·96 (80·91–83·03)	70·70 (67·35–73·75)	77·60 (76·42–78·76)	68·37 (65·55–71·00)	84·14 (82·16–85·93)	72·46 (68·90–75·73)	80·03 (77·74–82·35)	70·37 (67·28–73·45)
	Italy		80·26 (80·02–80·51)	69·20 (66·00–72·03)	73·69 (73·41–73·97)	65·23 (62·77–67·52)	83·75 (83·51–83·96)	72·24 (68·85–75·22)	78·24 (77·97–78·51)	69·07 (66·42–71·48)	84·62 (83·96–85·28)	72·93 (69·56–76·04)	79·92 (79·14–80·69)	70·48 (67·66–72·99)
	Luxembourg		78·49 (77·99–78·99)	67·27 (63·97–70·15)	71·77 (71·28–72·25)	63·07 (60·55–65·33)	82·91 (82·40–83·38)	70·86 (67·36–73·97)	77·78 (77·29–78·27)	67·96 (65·12–70·51)	83·89 (82·76–85·25)	71·72 (68·23–74·88)	80·27 (79·11–81·45)	70·13 (67·02–72·92)
	Malta		77·78 (76·65–78·88)	67·36 (64·26–70·34)	73·69 (72·64–74·75)	65·33 (62·75–67·78)	81·65 (80·63–82·66)	70·51 (67·17–73·48)	77·15 (76·16–78·14)	68·10 (65·44–70·44)	83·83 (82·07–85·79)	72·16 (68·59–75·60)	79·04 (77·13–81·02)	69·70 (66·63–72·57)
	Netherlands		80·09 (79·62–80·59)	68·69 (65·39–71·61)	73·87 (73·37–74·35)	65·23 (62·77–67·50)	81·91 (81·47–82·33)	70·14 (66·76–73·22)	77·65 (77·23–78·12)	68·24 (65·57–70·67)	83·61 (82·62–84·52)	71·53 (67·93–74·73)	79·63 (78·57–80·58)	69·85 (67·02–72·51)
	Norway		80·06 (79·58–80·53)	69·28 (66·16–72·13)	73·63 (73·13–74·20)	65·00 (62·42–67·32)	82·62 (82·16–83·03)	71·46 (68·27–74·40)	78·08 (77·63–78·52)	68·58 (65·80–71·04)	84·10 (82·90–85·35)	72·67 (69·39–75·72)	80·07 (78·99–81·28)	70·29 (67·42–73·13)
	Portugal		77·88 (77·56–78·20)	67·18 (64·16–70·00)	70·74 (70·33–71·13)	62·69 (60·23–64·73)	82·14 (81·79–82·47)	70·72 (67·42–73·65)	75·35 (74·92–75·78)	66·65 (64·07–68·98)	84·03 (83·35–84·77)	72·27 (68·81–75·39)	77·76 (76·94–78·65)	68·65 (65·82–71·07)
	Spain		80·63 (80·44–80·83)	69·85 (66·61–72·64)	73·59 (73·33–73·86)	65·44 (63·01–67·60)	84·03 (83·84–84·19)	72·75 (69·43–75·70)	77·60 (77·37–77·82)	68·86 (66·28–71·16)	85·59 (85·13–86·05)	73·98 (70·65–77·05)	80·28 (79·67–80·83)	71·20 (68·55–73·64)
	Sweden		80·43 (80·06–80·79)	68·95 (65·54–71·97)	74·85 (74·47–75·27)	66·02 (63·36–68·35)	82·83 (82·53–83·14)	70·85 (67·42–73·87)	78·66 (78·30–78·99)	69·03 (66·24–71·48)	83·96 (82·63–85·19)	71·69 (68·22–75·14)	80·13 (78·83–81·40)	70·06 (66·89–72·76)
	Switzerland		80·87 (80·18–81·52)	69·16 (65·79–72·25)	74·01 (73·23–74·76)	65·09 (62·45–67·47)	83·77 (83·16–84·33)	71·23 (67·55–74·56)	78·98 (78·26–79·69)	68·95 (66·07–71·59)	85·23 (82·61–87·65)	72·86 (68·72–76·47)	81·01 (78·06–83·61)	70·96 (67·24–74·23)
	UK		78·47 (78·35–78·61)	67·45 (64·27–70·31)	72·85 (72·72–72·98)	64·24 (61·72–66·47)	81·48 (81·36–81·62)	69·89 (66·55–72·82)	77·17 (77·04–77·29)	67·68 (64·93–70·16)	82·86 (82·65–83·07)	70·97 (67·58–74·07)	78·92 (78·71–79·13)	69·11 (66·28–71·60)
		England	78·69 (78·60–78·78)	67·57 (64·37–70·43)	73·12 (73·03–73·21)	64·43 (61·91–66·67)	81·72 (81·63–81·81)	70·01 (66·66–73·02)	77·47 (77·38–77·57)	67·89 (65·12–70·36)	83·11 (82·96–83·25)	71·12 (67·68–74·16)	79·24 (79·09–79·38)	69·35 (66·49–71·87)
		Northern Ireland	77·86 (76·97–78·86)	67·25 (64·19–70·16)	71·89 (70·92–72·94)	63·72 (61·27–65·96)	81·13 (80·33–82·04)	69·82 (66·50–72·92)	76·24 (75·24–77·20)	67·16 (64·47–69·69)	82·42 (80·83–83·98)	70·89 (67·48–74·17)	77·85 (76·04–79·70)	68·48 (65·52–71·38)
		Scotland	76·82 (75·91–77·71)	66·36 (63·34–69·05)	70·91 (69·99–71·81)	62·73 (60·22–65·05)	79·71 (78·83–80·64)	68·79 (65·75–71·68)	74·88 (73·94–75·77)	65·93 (63·25–68·37)	81·19 (79·67–82·62)	69·85 (66·62–73·15)	76·88 (75·38–78·37)	67·50 (64·51–70·41)
		Wales	78·36 (77·53–79·18)	67·60 (64·53–70·42)	72·57 (71·70–73·35)	64·21 (61·67–66·48)	81·17 (80·50–81·98)	70·03 (66·82–73·00)	76·81 (75·88–77·63)	67·61 (64·93–70·14)	82·21 (80·81–83·50)	70·78 (67·46–73·99)	77·93 (76·40–79·41)	68·50 (65·45–71·32)
Southern Latin America			76·13 (75·77–76·51)	66·19 (63·23–68·81)	68·97 (68·53–69·40)	61·19 (58·93–63·23)	79·48 (79·14–79·86)	68·97 (65·87–71·78)	72·79 (72·37–73·22)	64·40 (62·02–66·62)	80·88 (79·86–81·84)	70·09 (66·75–72·91)	74·36 (73·26–75·41)	65·72 (63·11–68·16)
	Argentina		75·75 (75·29–76·26)	65·95 (63·01–68·56)	68·54 (68·00–69·07)	60·85 (58·59–62·95)	78·76 (78·29–79·23)	68·49 (65·47–71·24)	71·82 (71·27–72·35)	63·67 (61·27–65·87)	79·99 (79·07–80·94)	69·45 (66·22–72·26)	73·31 (72·28–74·38)	64·91 (62·40–67·25)
	Chile		76·84 (76·09–77·67)	66·46 (63·38–69·30)	70·19 (69·28–71·14)	62·03 (59·52–64·35)	81·54 (80·87–82·23)	70·29 (67·00–73·23)	75·82 (75·07–76·66)	66·64 (63·96–69·06)	83·19 (80·15–86·14)	71·75 (67·58–75·74)	77·28 (73·98–80·51)	67·98 (64·16–71·77)
	Uruguay		76·50 (76·12–76·91)	66·84 (63·99–69·27)	69·10 (68·67–69·52)	61·72 (59·57–63·65)	79·58 (79·19–79·94)	69·39 (66·41–71·92)	72·02 (71·61–72·46)	64·20 (61·87–66·23)	81·08 (80·22–81·94)	70·49 (67·40–73·37)	73·45 (72·58–74·37)	65·28 (62·78–67·50)
**Central Europe, eastern Europe, and central Asia**			**73·62 (73·15–74·08)**	**63·68 (60·79–66·31)**	**64·56 (63·90–65·19)**	**56·76 (54·42–58·84)**	**74·26 (73·67–74·80)**	**64·48 (61·53–67·03)**	**63·87 (63·03–64·67)**	**56·45 (54·24–58·54)**	**77·22 (75·05–78·94)**	**66·83 (63·42–69·95)**	**68·17 (66·08–70·23)**	**59·99 (57·07–62·92)**
	Eastern Europe		74·39 (73·62–75·17)	64·24 (61·30–67·02)	64·17 (63·08–65·26)	56·40 (54·03–58·61)	73·71 (72·78–74·57)	64·01 (61·00–66·60)	61·30 (59·98–62·59)	54·31 (52·14–56·50)	76·64 (73·16–79·74)	66·36 (62·34–70·18)	66·07 (62·56–69·75)	58·32 (54·75–62·09)
		Belarus	75·42 (74·82–76·01)	65·14 (62·14–67·87)	65·70 (64·92–66·48)	57·89 (55·55–60·12)	75·38 (74·79–75·96)	65·33 (62·38–67·97)	63·34 (62·55–64·18)	56·18 (53·89–58·22)	78·76 (76·56–81·04)	67·94 (64·17–71·31)	68·18 (65·61–70·84)	60·07 (56·96–63·14)
		Estonia	75·26 (74·76–75·78)	64·68 (61·52–67·39)	64·84 (64·18–65·52)	56·91 (54·53–59·04)	78·54 (78·03–79·08)	67·67 (64·52–70·51)	67·68 (66·99–68·32)	59·59 (57·26–61·84)	81·79 (80·70–83·50)	70·24 (66·74–73·49)	72·96 (71·30–74·75)	63·79 (60·86–66·60)
		Latvia	74·65 (74·03–75·21)	64·20 (61·13–66·92)	64·27 (63·36–65·20)	56·36 (53·96–58·53)	75·93 (75·33–76·57)	65·68 (62·67–68·35)	64·98 (64·20–65·77)	57·45 (55·16–59·55)	79·79 (78·21–81·34)	68·63 (65·36–71·88)	69·96 (68·13–72·10)	61·53 (58·62–64·29)
		Lithuania	76·37 (75·92–76·81)	65·66 (62·50–68·43)	66·40 (65·75–67·05)	58·20 (55·89–60·35)	77·25 (76·78–77·69)	66·55 (63·41–69·39)	65·08 (64·46–65·73)	57·47 (55·20–59·56)	80·35 (79·43–81·33)	69·08 (65·80–72·07)	69·74 (68·57–70·95)	61·15 (58·32–63·59)
		Moldova	70·79 (69·73–71·89)	61·25 (58·46–63·91)	63·58 (62·30–65·03)	56·05 (53·64–58·31)	72·79 (71·81–73·84)	63·39 (60·49–66·05)	65·05 (63·70–66·39)	57·81 (55·42–60·17)	76·14 (74·59–77·77)	66·07 (62·90–69·03)	68·27 (66·56–70·28)	60·43 (57·66–63·22)
		Russia	74·30 (73·15–75·53)	64·17 (61·24–67·09)	63·68 (62·12–65·35)	55·93 (53·49–58·36)	73·46 (72·10–74·72)	63·81 (60·78–66·50)	60·62 (58·76–62·48)	53·67 (51·21–56·11)	76·24 (71·41–80·77)	66·09 (61·24–70·37)	65·39 (60·82–70·72)	57·78 (53·22–62·52)
		Ukraine	74·59 (73·87–75·29)	64·44 (61·53–67·12)	65·17 (64·23–66·02)	57·37 (54·94–59·57)	73·73 (73·02–74·47)	64·05 (61·13–66·66)	62·11 (61·14–63·17)	55·18 (53·00–57·24)	76·98 (73·53–80·45)	66·71 (62·72–70·66)	67·03 (63·32–71·46)	59·28 (55·74–63·20)
	Central Europe		74·78 (74·56–74·99)	64·82 (61·91–67·34)	67·20 (66·93–67·47)	59·01 (56·59–61·15)	78·06 (77·86–78·26)	67·66 (64·65–70·33)	70·72 (70·48–70·96)	62·05 (59·48–64·26)	80·26 (79·81–80·73)	69·37 (66·15–72·15)	73·46 (72·92–74·01)	64·18 (61·51–66·52)
		Albania	76·80 (76·16–77·40)	66·70 (63·79–69·40)	70·99 (70·33–71·57)	62·37 (59·85–64·70)	77·77 (77·25–78·26)	67·82 (64·84–70·51)	72·54 (71·95–73·05)	63·92 (61·30–66·25)	80·73 (79·37–82·15)	70·09 (67·00–73·06)	74·70 (73·12–76·39)	65·64 (62·73–68·36)
		Bosnia and Herzegovina	75·13 (73·76–76·67)	65·36 (62·38–68·11)	69·04 (67·54–70·60)	60·82 (58·20–63·58)	78·49 (77·21–79·85)	68·19 (65·07–71·10)	73·34 (71·84–74·76)	64·16 (61·33–66·77)	80·22 (78·35–82·06)	69·40 (66·06–72·61)	74·92 (73·02–76·90)	65·25 (62·10–68·20)
		Bulgaria	74·75 (74·36–75·12)	65·12 (62·33–67·64)	68·21 (67·75–68·62)	59·90 (57·43–62·08)	76·38 (76·03–76·73)	66·65 (63·83–69·18)	69·21 (68·75–69·66)	61·13 (58·74–63·22)	78·48 (76·47–80·36)	68·23 (64·85–71·41)	71·66 (69·55–73·90)	63·05 (60·16–66·08)
		Croatia	76·05 (75·15–76·92)	66·16 (63·16–68·86)	68·33 (67·25–69·54)	60·18 (57·73–62·48)	78·93 (78·19–79·66)	68·66 (65·57–71·40)	71·76 (70·89–72·70)	63·07 (60·52–65·48)	80·48 (79·01–82·09)	69·84 (66·51–72·81)	74·18 (72·61–76·00)	64·97 (62·11–67·80)
		Czech Republic	75·56 (75·29–75·80)	65·19 (62·16–67·81)	68·09 (67·77–68·40)	59·76 (57·30–61·86)	79·71 (79·42–79·96)	68·54 (65·33–71·43)	73·32 (73·01–73·66)	63·79 (61·01–66·25)	81·89 (81·28–82·51)	70·17 (66·63–73·22)	76·25 (75·52–76·95)	65·99 (62·86–68·76)
		Hungary	73·86 (73·19–74·59)	63·70 (60·67–66·39)	65·29 (64·49–66·12)	57·37 (55·04–59·60)	77·51 (76·87–78·17)	66·99 (64·02–69·86)	69·15 (68·34–69·98)	60·82 (58·29–63·12)	79·08 (77·74–80·34)	68·25 (65·05–71·40)	72·24 (70·72–73·89)	63·29 (60·37–66·06)
		Macedonia	72·90 (72·13–73·66)	63·75 (61·00–66·39)	68·25 (67·47–69·07)	60·33 (57·97–62·57)	75·51 (74·84–76·21)	66·07 (63·33–68·61)	70·72 (70·05–71·46)	62·54 (60·07–64·69)	77·38 (76·62–78·14)	67·46 (64·51–70·11)	72·36 (71·51–73·31)	63·78 (61·12–66·12)
		Montenegro	77·78 (76·60–78·77)	67·53 (64·55–70·39)	72·15 (70·99–73·33)	63·20 (60·35–65·67)	78·22 (77·57–78·80)	68·03 (64·91–70·75)	72·32 (71·56–73·15)	63·53 (60·90–65·94)	79·74 (78·74–81·20)	69·19 (65·84–72·36)	74·35 (73·14–75·51)	65·09 (62·29–67·77)
		Poland	75·45 (75·00–75·89)	65·44 (62·51–68·02)	66·52 (65·99–67·14)	58·50 (56·18–60·68)	79·51 (79·12–79·95)	68·82 (65·68–71·57)	71·08 (70·52–71·66)	62·28 (59·68–64·58)	81·70 (80·62–82·78)	70·53 (67·24–73·68)	74·08 (72·83–75·37)	64·61 (61·82–67·22)
		Romania	73·19 (72·61–73·78)	63·48 (60·52–66·01)	66·61 (65·86–67·30)	58·28 (55·79–60·54)	76·46 (75·89–77·05)	66·33 (63·40–69·05)	69·22 (68·56–69·89)	60·81 (58·29–63·07)	78·88 (77·68–80·08)	68·33 (65·15–71·19)	71·66 (70·14–73·15)	62·82 (60·01–65·52)
		Serbia	75·57 (74·59–76·72)	65·60 (62·61–68·47)	69·62 (68·54–70·89)	61·20 (58·60–63·73)	76·21 (75·52–76·87)	66·32 (63·47–68·92)	70·89 (70·13–71·71)	62·34 (59·78–64·74)	78·78 (78·14–79·39)	68·21 (65·12–70·93)	73·03 (72·20–73·92)	63·93 (61·27–66·40)
		Slovakia	75·40 (74·92–75·91)	65·48 (62·55–68·09)	66·74 (66·12–67·40)	58·67 (56·21–60·75)	78·32 (77·86–78·82)	68·02 (65·01–70·67)	70·47 (69·87–71·01)	61·80 (59·17–64·11)	80·39 (79·07–81·78)	69·59 (66·48–72·65)	73·43 (71·87–75·05)	64·10 (61·10–66·96)
		Slovenia	77·54 (76·57–78·53)	66·71 (63·55–69·70)	69·54 (68·45–70·81)	60·68 (57·92–63·14)	81·53 (80·68–82·48)	69·77 (66·41–72·88)	74·22 (73·07–75·34)	64·26 (61·17–67·11)	83·82 (82·53–85·29)	71·49 (67·82–74·79)	77·82 (76·19–79·51)	67·16 (63·87–70·44)
	Central Asia		70·96 (70·47–71·43)	61·67 (58·93–64·15)	63·41 (62·78–64·01)	56·13 (53·81–58·15)	71·21 (70·56–71·85)	62·21 (59·51–64·62)	63·14 (62·28–63·98)	56·21 (54·16–58·20)	75·11 (74·09–76·00)	65·35 (62·41–68·00)	67·50 (66·36–68·69)	59·80 (57·45–62·23)
		Armenia	73·65 (72·86–74·55)	64·21 (61·39–66·67)	66·84 (65·87–67·83)	58·93 (56·53–61·07)	75·86 (75·06–76·62)	66·28 (63·53–68·81)	69·04 (68·00–70·04)	61·17 (58·69–63·40)	79·32 (77·99–80·71)	69·06 (65·86–71·81)	71·97 (70·46–73·52)	63·59 (60·90–66·11)
		Azerbaijan	70·14 (68·89–71·42)	61·29 (58·55–63·91)	62·31 (60·84–63·68)	55·45 (53·04–57·80)	71·21 (69·75–72·64)	62·51 (59·64–65·19)	64·79 (63·25–66·29)	57·83 (55·48–60·13)	75·80 (73·51–77·94)	66·17 (62·90–69·11)	68·41 (65·71–71·21)	60·78 (57·79–63·89)
		Georgia	73·69 (72·60–74·77)	64·60 (61·82–67·19)	65·44 (64·13–66·83)	58·19 (55·77–60·33)	77·15 (76·33–77·91)	67·50 (64·60–70·01)	67·75 (66·57–68·93)	60·39 (57·98–62·58)	78·83 (76·59–81·37)	68·78 (65·53–71·92)	69·07 (66·20–72·20)	61·32 (58·06–64·46)
		Kazakhstan	72·57 (71·28–73·83)	62·91 (59·93–65·60)	63·02 (61·42–64·78)	55·66 (52·95–58·03)	70·91 (69·54–72·39)	61·82 (58·83–64·44)	59·58 (57·80–61·41)	52·99 (50·58–55·46)	76·18 (73·87–78·50)	66·04 (62·70–69·28)	67·14 (64·13–69·98)	59·19 (56·00–62·47)
		Kyrgyzstan	70·12 (69·47–70·74)	60·78 (58·04–63·25)	61·84 (61·08–62·69)	54·55 (52·38–56·53)	70·78 (70·19–71·40)	61·83 (59·15–64·16)	62·51 (61·72–63·27)	55·63 (53·46–57·56)	74·95 (73·75–76·12)	65·15 (62·22–67·95)	67·51 (66·07–68·99)	59·76 (57·08–62·16)
		Mongolia	65·20 (63·76–66·84)	57·09 (54·37–59·60)	59·84 (58·18–61·22)	53·18 (50·89–55·48)	68·78 (67·56–69·88)	60·40 (57·70–62·78)	60·23 (58·86–61·76)	53·67 (51·52–55·83)	73·04 (71·09–75·10)	63·80 (60·59–66·78)	63·65 (61·39–65·84)	56·38 (53·52–59·08)
		Tajikistan	67·42 (65·76–69·13)	58·79 (56·10–61·55)	62·44 (60·73–64·21)	55·20 (52·77–57·69)	70·35 (68·77–71·80)	61·69 (58·89–64·26)	65·68 (64·11–67·36)	58·31 (55·63–60·71)	74·34 (72·42–76·08)	64·95 (61·89–67·79)	69·41 (67·10–71·52)	61·41 (58·46–64·17)
		Turkmenistan	67·10 (66·05–68·06)	58·73 (55·99–61·13)	60·29 (59·01–61·58)	53·72 (51·34–55·90)	69·90 (68·65–71·06)	61·43 (58·65–63·85)	61·78 (60·32–63·14)	55·19 (52·77–57·37)	74·00 (72·71–75·15)	64·86 (62·01–67·41)	66·48 (65·09–67·80)	59·26 (56·77–61·54)
		Uzbekistan	71·30 (70·34–72·21)	61·69 (58·72–64·34)	65·26 (64·22–66·31)	57·73 (55·40–60·00)	70·16 (68·96–71·38)	61·23 (58·40–63·90)	64·02 (62·34–65·56)	57·06 (54·76–59·46)	73·50 (71·85–75·16)	63·99 (60·85–66·87)	67·08 (65·28–68·84)	59·63 (56·99–62·15)
**Latin America and Caribbean**			**72·68 (72·47–72·88)**	**63·19 (60·37–65·62)**	**66·44 (66·15–66·70)**	**58·85 (56·56–60·86)**	**77·02 (76·79–77·24)**	**66·87 (63·91–69·49)**	**70·87 (70·59–71·12)**	**62·65 (60·25–64·80)**	**78·89 (78·44–79·29)**	**68·50 (65·36–71·17)**	**72·75 (72·23–73·22)**	**64·24 (61·68–66·39)**
	Central Latin America		74·27 (74·01–74·52)	64·94 (62·15–67·38)	68·10 (67·78–68·43)	60·43 (58·05–62·41)	77·98 (77·72–78·24)	68·23 (65·34–70·75)	72·07 (71·74–72·39)	63·97 (61·57–66·13)	79·44 (78·96–79·94)	69·42 (66·40–71·99)	73·58 (72·90–74·21)	65·21 (62·67–67·40)
		Colombia	75·14 (74·65–75·61)	65·94 (63·10–68·39)	67·62 (67·02–68·16)	60·35 (58·03–62·34)	78·34 (77·93–78·75)	68·76 (65·91–71·28)	72·04 (71·54–72·55)	64·29 (61·90–66·41)	81·05 (79·95–82·12)	71·08 (68·06–73·83)	75·42 (74·17–76·72)	67·08 (64·50–69·48)
		Costa Rica	78·90 (78·33–79·44)	69·10 (66·16–71·74)	74·24 (73·67–74·83)	66·15 (63·73–68·37)	82·16 (81·63–82·63)	71·73 (68·60–74·44)	77·09 (76·50–77·68)	68·42 (65·76–70·84)	83·57 (82·65–84·51)	72·85 (69·62–75·81)	78·47 (77·41–79·58)	69·52 (66·66–72·08)
		El Salvador	72·47 (71·78–73·20)	63·57 (60·78–66·00)	63·87 (62·97–64·87)	56·75 (54·59–58·80)	77·36 (76·76–78·00)	67·83 (64·98–70·32)	69·32 (68·50–70·20)	61·47 (59·00–63·74)	79·07 (77·61–80·53)	69·30 (66·19–72·15)	71·46 (69·33–73·45)	63·36 (60·64–66·09)
		Guatemala	66·49 (64·79–68·38)	58·05 (55·25–60·73)	62·75 (60·95–65·05)	55·37 (52·93–58·14)	73·75 (71·64–75·73)	64·16 (60·86–67·31)	66·57 (64·06–69·21)	58·78 (55·71–61·97)	76·03 (72·36–79·77)	66·34 (62·24–70·02)	69·40 (65·37–73·96)	61·41 (57·28–65·80)
		Honduras	67·23 (65·64–68·91)	59·20 (56·47–61·74)	67·35 (63·79–70·22)	59·91 (56·85–63·19)	71·72 (68·57–75·52)	63·09 (59·35–66·67)	69·97 (65·91–74·24)	62·44 (58·43–66·60)	73·71 (70·41–77·70)	64·78 (60·90–68·63)	71·64 (67·32–75·82)	63·86 (59·59–67·72)
		Mexico	74·77 (74·46–75·10)	65·19 (62·44–67·68)	68·33 (67·95–68·72)	60·44 (58·05–62·49)	78·14 (77·79–78·46)	68·29 (65·38–70·81)	72·79 (72·42–73·13)	64·44 (61·96–66·62)	79·11 (78·65–79·56)	69·00 (65·92–71·58)	73·71 (73·20–74·19)	65·17 (62·59–67·43)
		Nicaragua	76·43 (75·87–77·02)	66·58 (63·66–69·19)	70·74 (70·04–71·41)	62·56 (59·98–64·83)	79·65 (79·10–80·27)	69·67 (66·69–72·42)	74·08 (73·42–74·74)	65·79 (63·30–68·12)	81·17 (79·21–83·22)	70·94 (67·63–74·13)	75·25 (72·85–77·72)	66·84 (63·52–69·99)
		Panama	77·54 (76·65–78·49)	67·96 (65·04–70·56)	72·50 (71·40–73·60)	64·56 (62·18–66·83)	80·33 (79·47–81·20)	70·30 (67·20–73·04)	74·78 (73·66–75·91)	66·44 (63·89–68·76)	81·98 (80·38–83·60)	71·57 (68·22–74·62)	76·03 (73·97–78·07)	67·33 (64·33–70·19)
		Venezuela	75·41 (74·60–76·29)	66·21 (63·41–68·75)	69·98 (68·88–70·90)	62·40 (59·97–64·62)	79·05 (78·13–79·89)	69·26 (66·19–71·84)	70·98 (69·93–72·11)	63·25 (60·76–65·44)	79·79 (77·49–81·84)	69·87 (66·40–73·26)	71·30 (68·22–73·83)	63·46 (60·14–66·42)
	Andean Latin America		70·08 (69·45–70·78)	61·05 (58·27–63·48)	66·45 (65·71–67·13)	58·65 (56·38–60·66)	77·68 (77·01–78·35)	67·63 (64·50–70·22)	73·50 (72·83–74·19)	64·81 (62·21–67·09)	79·76 (78·33–81·16)	69·43 (66·22–72·41)	76·02 (74·60–77·53)	66·99 (64·01–69·65)
		Bolivia	62·16 (60·65–63·60)	54·02 (51·26–56·57)	60·53 (59·19–61·86)	53·13 (50·77–55·31)	71·77 (70·20–73·10)	62·29 (59·32–65·00)	69·14 (67·72–70·73)	60·72 (57·94–63·35)	74·27 (71·23–76·97)	64·59 (60·91–67·96)	72·23 (69·63–75·00)	63·45 (60·07–66·93)
		Ecuador	73·91 (73·34–74·50)	64·46 (61·68–67·01)	68·98 (68·32–69·61)	60·96 (58·50–63·15)	78·62 (78·06–79·21)	68·58 (65·52–71·15)	73·05 (72·29–73·76)	64·42 (61·86–66·79)	80·36 (79·36–81·30)	70·02 (66·82–72·77)	75·35 (74·11–76·49)	66·43 (63·73–69·03)
		Peru	71·42 (70·42–72·50)	62·21 (59·36–64·81)	67·44 (66·30–68·55)	59·59 (57·17–61·81)	79·55 (78·55–80·63)	69·25 (66·06–72·00)	75·44 (74·35–76·53)	66·62 (63·82–69·13)	81·60 (79·22–83·98)	71·03 (67·47–74·59)	77·80 (75·33–80·32)	68·64 (65·32–71·83)
	Caribbean		69·50 (68·81–70·13)	60·44 (57·64–62·88)	65·93 (65·29–66·61)	58·28 (55·90–60·50)	72·40 (71·53–73·27)	62·90 (60·07–65·48)	68·67 (67·79–69·54)	60·57 (58·07–62·88)	75·35 (74·02–76·61)	65·39 (62·39–68·26)	71·00 (69·69–72·33)	62·58 (59·79–65·00)
		Antigua and Barbuda	76·68 (75·47–77·85)	66·92 (63·82–69·77)	70·84 (69·63–71·97)	62·64 (60·04–65·02)	78·76 (77·61–79·93)	68·43 (65·23–71·40)	73·34 (72·15–74·65)	64·53 (61·68–67·13)	79·92 (78·32–81·59)	69·37 (65·99–72·65)	74·65 (72·67–76·41)	65·61 (62·64–68·53)
		The Bahamas	74·00 (73·00–74·89)	64·52 (61·51–67·24)	67·09 (66·00–68·09)	59·62 (57·31–61·77)	76·19 (75·39–77·01)	66·38 (63·53–69·02)	70·46 (69·68–71·19)	62·45 (60·10–64·62)	76·30 (74·58–77·97)	66·49 (63·31–69·38)	71·33 (69·70–72·93)	63·22 (60·52–65·91)
		Barbados	76·16 (75·12–77·15)	66·36 (63·33–69·04)	70·92 (69·66–71·99)	62·90 (60·42–65·23)	78·49 (77·53–79·38)	68·02 (64·90–70·80)	73·54 (72·47–74·50)	64·95 (62·20–67·34)	78·68 (77·25–80·06)	68·10 (64·80–71·19)	74·38 (72·78–75·92)	65·64 (62·79–68·37)
		Belize	73·93 (72·68–75·23)	64·26 (61·15–67·05)	69·38 (67·81–70·50)	61·39 (58·70–63·71)	73·29 (72·38–74·17)	63·80 (60·87–66·53)	67·40 (66·22–68·53)	59·76 (57·23–62·05)	74·95 (73·10–76·81)	65·15 (61·97–68·12)	69·11 (67·10–71·40)	61·25 (58·23–64·02)
		Bermuda	72·90 (71·87–73·98)	63·98 (61·26–66·60)	66·78 (65·67–67·86)	59·48 (57·02–61·60)	78·00 (76·91–79·03)	68·18 (65·20–70·92)	72·81 (71·81–73·79)	64·62 (62·21–66·92)	82·39 (80·69–84·46)	71·70 (68·30–74·84)	75·74 (74·10–77·22)	67·03 (64·10–69·63)
		Cuba	76·98 (76·43–77·56)	66·87 (63·85–69·51)	72·70 (72·06–73·27)	64·51 (61·96–66·78)	79·92 (79·40–80·40)	69·58 (66·44–72·32)	75·70 (75·10–76·28)	67·09 (64·47–69·42)	81·30 (80·31–82·36)	70·66 (67·44–73·66)	76·66 (75·59–77·74)	67·80 (65·03–70·35)
		Dominica	73·68 (72·69–74·70)	64·65 (61·88–67·09)	70·44 (69·45–71·54)	62·42 (59·96–64·72)	75·98 (74·80–77·05)	66·47 (63·56–69·05)	70·83 (69·73–71·92)	62·68 (60·23–64·98)	75·84 (73·91–77·37)	66·21 (63·22–69·11)	70·19 (67·89–72·38)	62·08 (59·01–65·02)
		Dominican Republic	72·72 (72·03–73·64)	63·61 (60·92–66·27)	68·92 (67·91–69·97)	61·20 (58·76–63·51)	76·56 (75·18–78·17)	66·72 (63·43–69·68)	70·72 (69·57–72·27)	62·60 (60·06–65·04)	78·57 (76·90–80·63)	68·47 (65·35–71·80)	72·88 (71·17–74·87)	64·51 (61·65–67·18)
		Grenada	73·07 (71·50–74·35)	63·29 (60·23–66·00)	68·65 (66·90–70·37)	60·45 (57·69–63·13)	73·99 (72·55–75·31)	64·33 (61·30–67·01)	68·33 (67·02–69·71)	60·44 (57·82–62·97)	74·43 (72·29–76·68)	64·67 (61·62–67·69)	68·80 (66·68–71·09)	60·80 (57·83–63·61)
		Guyana	67·79 (66·80–68·87)	58·86 (56·01–61·35)	60·94 (59·78–62·10)	53·86 (51·57–56·07)	68·30 (67·14–69·35)	59·16 (56·33–61·63)	61·77 (60·64–62·99)	54·48 (52·07–56·63)	71·17 (69·59–72·89)	61·60 (58·62–64·52)	64·67 (62·99–66·41)	57·01 (54·30–59·41)
		Haiti	54·27 (52·55–56·07)	47·10 (44·54–49·51)	53·86 (52·03–55·82)	47·16 (44·50–49·59)	58·20 (55·79–60·80)	50·59 (47·70–53·48)	58·51 (56·00–61·09)	51·37 (48·35–54·31)	64·42 (61·40–67·53)	55·98 (52·71–59·53)	63·58 (60·17–66·93)	55·92 (52·30–59·54)
		Jamaica	75·36 (74·03–76·62)	65·33 (62·13–68·20)	73·44 (72·09–74·70)	64·70 (61·97–67·25)	76·36 (75·09–77·66)	66·06 (62·79–69·00)	73·28 (71·90–74·66)	64·54 (61·62–67·13)	76·79 (74·50–79·15)	66·43 (63·09–69·99)	73·06 (70·43–75·53)	64·33 (61·11–67·43)
		Puerto Rico	78·45 (77·86–79·07)	68·36 (65·33–71·06)	69·65 (68·82–70·46)	61·58 (59·12–63·83)	81·08 (80·48–81·63)	70·21 (66·94–73·17)	73·52 (72·69–74·23)	64·40 (61·61–66·85)	82·36 (81·27–83·54)	71·33 (68·04–74·29)	75·04 (73·74–76·39)	65·81 (62·94–68·39)
		Saint Lucia	74·26 (73·22–75·19)	64·00 (60·85–66·81)	69·52 (68·34–70·56)	60·90 (58·14–63·36)	77·55 (76·60–78·37)	67·25 (64·22–70·02)	71·39 (70·30–72·29)	62·91 (60·23–65·30)	79·29 (78·14–80·36)	68·59 (65·28–71·52)	73·05 (71·64–74·27)	64·24 (61·19–66·75)
		Saint Vincent and the Grenadines	72·72 (71·69–73·72)	63·32 (60·36–65·87)	68·54 (67·17–69·93)	60·62 (58·00–62·99)	73·96 (73·29–74·76)	64·30 (61·47–67·01)	68·62 (67·69–69·48)	60·56 (58·17–62·78)	74·80 (73·67–76·09)	65·00 (62·00–67·90)	68·80 (67·42–70·06)	60·65 (58·05–63·14)
		Suriname	72·53 (71·89–73·22)	62·93 (59·91–65·65)	67·75 (66·99–68·41)	59·79 (57·40–61·92)	72·46 (71·60–73·26)	62·79 (59·75–65·34)	66·70 (65·95–67·45)	58·89 (56·40–61·02)	74·37 (73·12–75·41)	64·32 (61·24–67·12)	68·41 (67·13–69·69)	60·24 (57·53–62·67)
		Trinidad and Tobago	71·87 (71·25–72·56)	62·39 (59·64–64·91)	66·28 (65·59–66·97)	58·68 (56·31–60·87)	75·17 (74·41–75·89)	65·04 (61·98–67·79)	68·14 (67·33–68·96)	59·89 (57·46–62·16)	76·98 (75·63–78·20)	66·48 (63·13–69·33)	69·31 (67·78–70·76)	60·75 (57·91–63·36)
		Virgin Islands	76·11 (75·19–77·53)	66·70 (63·82–69·42)	69·19 (68·00–70·51)	61·53 (59·07–63·76)	77·74 (76·93–78·51)	67·90 (64·87–70·49)	70·08 (68·90–71·14)	62·18 (59·66–64·42)	78·77 (77·54–80·06)	68·65 (65·55–71·60)	70·63 (68·75–72·91)	62·58 (59·71–65·48)
	Tropical Latin America		72·47 (72·03–72·91)	62·56 (59·56–65·08)	64·85 (64·34–65·35)	57·38 (55·02–59·42)	76·95 (76·51–77·34)	66·18 (62·94–68·96)	69·57 (69·10–70·00)	61·27 (58·86–63·46)	78·92 (78·42–79·40)	67·98 (64·72–70·81)	71·59 (70·97–72·12)	62·99 (60·40–65·22)
		Brazil	72·40 (71·94–72·85)	62·50 (59·50–65·07)	64·64 (64·13–65·16)	57·20 (54·86–59·22)	77·01 (76·56–77·40)	66·21 (62·96–69·01)	69·49 (69·01–69·92)	61·20 (58·75–63·39)	79·00 (78·49–79·49)	68·05 (64·76–70·88)	71·56 (70·94–72·10)	62·96 (60·36–65·16)
		Paraguay	76·30 (75·51–76·96)	65·92 (62·83–68·70)	72·86 (72·16–73·63)	64·25 (61·74–66·64)	76·20 (75·30–77·15)	65·88 (62·68–68·75)	72·04 (71·31–72·71)	63·45 (60·88–65·75)	77·10 (75·69–78·52)	66·50 (63·13–69·55)	72·09 (70·29–73·53)	63·58 (60·67–66·17)
**Southeast Asia, east Asia, and Oceania**			**68·81 (68·39–69·21)**	**60·57 (58·22–62·65)**	**64·47 (64·04–64·88)**	**57·98 (56·05–59·68)**	**74·53 (74·29–74·77)**	**65·49 (62·86–67·79)**	**69·18 (68·89–69·46)**	**62·13 (60·08–64·01)**	**78·37 (78·08–78·64)**	**68·55 (65·74–71·01)**	**72·11 (71·76–72·42)**	**64·57 (62·36–66·48)**
	East Asia		69·41 (68·83–69·96)	61·28 (58·95–63·37)	65·16 (64·58–65·69)	58·89 (57·00–60·58)	75·81 (75·53–76·10)	66·80 (64·19–69·11)	70·34 (70·04–70·64)	63·49 (61·45–65·35)	79·81 (79·51–80·12)	69·96 (67·21–72·47)	73·29 (72·97–73·63)	65·94 (63·80–67·83)
		China	69·20 (68·61–69·76)	61·11 (58·80–63·20)	64·98 (64·41–65·53)	58·75 (56·87–60·44)	75·81 (75·51–76·11)	66·83 (64·23–69·14)	70·34 (70·03–70·65)	63·50 (61·47–65·37)	79·93 (79·63–80·24)	70·06 (67·29–72·55)	73·36 (73·04–73·69)	66·01 (63·88–67·91)
		North Korea	74·06 (72·75–75·74)	65·06 (62·20–67·84)	69·18 (67·60–71·13)	62·46 (60·03–64·70)	72·48 (71·28–73·81)	63·39 (60·56–66·10)	67·13 (66·02–68·33)	60·62 (58·62–62·50)	73·59 (72·47–74·73)	64·70 (61·97–67·31)	67·88 (66·67–69·08)	61·17 (58·94–63·24)
		Taiwan (Province of China)	76·89 (76·37–77·41)	67·05 (64·12–69·73)	71·51 (70·87–72·12)	63·93 (61·56–65·98)	81·18 (80·69–81·66)	70·66 (67·64–73·26)	74·93 (74·33–75·59)	66·97 (64·74–69·04)	82·84 (81·44–84·41)	72·06 (68·74–75·22)	76·68 (74·97–78·54)	68·36 (65·70–70·89)
	Southeast Asia		67·54 (67·07–68·01)	59·01 (56·53–61·29)	63·14 (62·68–63·59)	55·93 (53·88–57·83)	72·13 (71·76–72·56)	63·04 (60·38–65·34)	67·15 (66·61–67·64)	59·50 (57·41–61·49)	75·51 (75·03–75·96)	65·84 (63·04–68·32)	69·96 (69·31–70·56)	61·94 (59·60–63·93)
		Cambodia	59·25 (58·26–60·38)	51·14 (48·49–53·52)	55·50 (54·32–56·71)	48·59 (46·34–50·71)	66·38 (65·50–67·29)	57·46 (54·75–59·88)	61·11 (59·88–62·24)	53·61 (51·44–55·67)	71·60 (70·52–72·72)	62·14 (59·37–64·72)	65·72 (64·50–67·01)	57·97 (55·56–60·13)
		Indonesia	64·90 (64·34–65·40)	56·93 (54·56–59·05)	62·37 (61·83–62·90)	55·36 (53·33–57·28)	70·24 (69·77–70·68)	61·59 (58·97–63·78)	67·39 (66·80–68·02)	59·74 (57·49–61·82)	73·56 (72·96–74·12)	64·15 (61·36–66·57)	69·82 (68·83–70·75)	61·78 (59·34–63·95)
		Laos	54·18 (52·72–55·48)	47·28 (44·92–49·41)	50·74 (49·16–52·24)	44·90 (42·56–47·03)	63·14 (61·87–64·24)	55·13 (52·55–57·41)	58·71 (57·49–59·91)	51·99 (49·78–54·10)	69·70 (68·04–71·13)	60·82 (57·98–63·41)	64·82 (62·91–66·40)	57·37 (54·73–59·81)
		Malaysia	73·70 (73·40–74·04)	64·48 (61·72–66·81)	69·61 (69·27–69·95)	61·94 (59·67–63·97)	75·99 (75·74–76·23)	66·58 (63·75–68·95)	72·12 (71·80–72·45)	63·96 (61·61–66·06)	78·05 (77·50–78·57)	68·14 (65·17–70·73)	73·16 (72·41–73·94)	64·78 (62·37–66·97)
		Maldives	62·00 (60·92–63·23)	53·61 (51·08–56·05)	62·85 (61·73–64·05)	55·11 (52·72–57·26)	77·42 (76·45–78·47)	66·95 (63·89–69·74)	75·23 (74·21–76·20)	66·13 (63·33–68·64)	81·33 (78·62–83·96)	70·26 (66·32–73·72)	77·58 (74·97–80·31)	68·23 (64·84–71·50)
		Mauritius	73·42 (72·89–73·94)	63·63 (60·79–66·22)	65·39 (64·86–65·92)	58·03 (55·89–59·98)	75·89 (75·31–76·40)	65·67 (62·62–68·28)	69·19 (68·61–69·83)	60·98 (58·61–63·08)	77·81 (76·00–79·57)	67·12 (63·74–70·36)	71·42 (69·59–73·36)	62·67 (59·53–65·47)
		Myanmar	60·93 (59·45–62·34)	53·09 (50·61–55·57)	56·26 (54·54–57·98)	49·56 (47·16–51·97)	67·28 (65·95–68·50)	58·84 (56·20–61·27)	61·28 (58·40–63·10)	54·21 (51·28–56·61)	73·35 (72·38–74·34)	64·11 (61·36–66·65)	66·73 (65·16–67·87)	59·04 (56·43–61·32)
		Philippines	70·92 (70·24–71·61)	61·69 (58·96–64·14)	63·45 (62·60–64·36)	55·80 (53·48–57·96)	72·07 (71·24–72·76)	62·73 (59·95–65·22)	64·58 (63·69–65·46)	57·07 (54·88–59·12)	73·87 (72·05–75·85)	64·47 (61·52–67·57)	66·64 (64·58–68·74)	59·07 (56·50–61·71)
		Sri Lanka	75·38 (74·55–76·26)	65·51 (62·68–68·15)	67·77 (66·67–68·89)	59·80 (57·30–62·16)	78·31 (77·17–79·27)	68·15 (65·22–70·82)	70·46 (69·17–71·65)	62·27 (59·85–64·54)	81·09 (78·67–83·77)	70·47 (66·87–74·07)	73·72 (70·53–76·90)	65·07 (61·57–68·32)
		Seychelles	74·15 (73·50–74·82)	65·14 (62·54–67·54)	64·48 (63·41–65·58)	57·71 (55·61–59·61)	76·20 (75·56–77·25)	66·77 (63·92–69·39)	68·64 (67·74–69·55)	61·10 (58·91–63·25)	77·40 (76·02–78·87)	67·72 (64·76–70·58)	70·25 (68·17–72·03)	62·41 (59·83–64·93)
		Thailand	74·08 (73·42–74·68)	64·41 (61·59–66·96)	67·20 (66·41–68·01)	59·48 (57·24–61·59)	77·81 (77·23–78·38)	67·73 (64·85–70·31)	71·48 (70·70–72·29)	63·15 (60·71–65·36)	80·91 (79·63–82·00)	70·24 (67·11–73·26)	74·59 (72·93–76·22)	65·71 (62·91–68·38)
		Timor-Leste	57·61 (54·99–60·60)	50·23 (47·11–53·26)	58·39 (56·06–60·79)	50·62 (47·62–53·51)	69·06 (66·95–71·25)	60·09 (57·11–63·16)	67·57 (65·41–69·81)	58·60 (55·53–61·71)	73·68 (70·57–76·59)	64·27 (60·67–67·86)	71·69 (68·65–74·73)	62·39 (58·56–65·67)
		Vietnam	71·11 (69·45–73·05)	62·16 (59·16–65·03)	65·27 (63·23–67·12)	58·12 (55·61–60·67)	75·49 (73·94–77·73)	66·09 (63·17–69·01)	68·53 (66·58–70·74)	61·21 (58·75–63·77)	78·10 (76·59–79·12)	68·44 (65·57–71·08)	70·87 (69·14–72·67)	63·30 (60·72–65·77)
	Oceania		60·31 (58·44–62·02)	52·36 (49·47–54·96)	56·79 (55·08–58·47)	50·27 (47·85–52·56)	61·11 (58·80–63·18)	53·01 (49·94–55·71)	58·35 (56·00–60·49)	51·58 (48·75–54·22)	63·76 (61·41–66·00)	55·16 (51·88–58·03)	60·72 (58·24–62·92)	53·57 (50·64–56·19)
		American Samoa	75·02 (73·75–76·44)	64·48 (61·29–67·60)	66·93 (65·44–68·49)	58·64 (56·07–61·15)	74·38 (72·61–75·88)	63·93 (60·72–67·22)	69·25 (67·72–70·70)	60·50 (57·64–63·08)	74·43 (71·97–77·03)	63·94 (60·47–67·29)	70·37 (67·82–72·73)	61·39 (58·12–64·51)
		Federated States of Micronesia	65·60 (63·23–67·73)	57·09 (53·99–59·87)	61·03 (58·77–63·16)	54·13 (51·41–56·65)	67·23 (64·39–69·83)	58·56 (55·40–61·67)	64·01 (61·74–66·18)	56·67 (53·87–59·30)	67·58 (64·54–70·55)	58·84 (55·44–62·17)	63·57 (60·73–66·27)	56·28 (53·25–59·28)
		Fiji	68·04 (63·85–72·17)	58·99 (54·86–62·98)	62·73 (59·19–66·20)	55·32 (51·72–58·77)	67·12 (65·31–69·36)	58·07 (54·97–61·03)	62·61 (60·57–64·97)	55·23 (52·53–57·84)	67·67 (63·49–71·89)	58·63 (54·64–62·73)	63·27 (59·17–67·99)	56·00 (52·16–60·22)
		Guam	76·44 (75·25–77·71)	66·30 (63·15–69·05)	70·34 (68·94–71·64)	62·61 (60·08–64·88)	76·69 (75·63–77·75)	66·43 (63·41–69·36)	70·14 (68·50–71·30)	62·27 (59·67–64·55)	76·09 (74·28–78·08)	65·75 (62·62–68·84)	69·05 (66·86–71·35)	61·20 (58·42–63·78)
		Kiribati	60·87 (58·96–62·74)	52·77 (49·98–55·51)	55·53 (53·76–57·42)	49·42 (47·04–51·64)	63·92 (61·83–65·92)	55·29 (52·20–58·18)	56·63 (54·56–58·59)	50·15 (47·47–52·68)	65·59 (62·39–68·39)	56·70 (53·26–60·06)	58·17 (55·44–61·12)	51·49 (48·39–54·51)
		Marshall Islands	67·99 (66·53–69·39)	58·54 (55·60–61·38)	61·47 (59·95–62·84)	54·23 (51·81–56·48)	65·11 (63·20–67·11)	56·14 (53·04–59·02)	61·00 (59·18–62·90)	53·70 (51·07–56·26)	67·26 (64·36–70·12)	57·87 (54·25–61·23)	62·75 (60·08–65·45)	55·07 (52·11–58·20)
		Northern Mariana Islands	74·74 (72·01–77·56)	64·95 (61·34–68·42)	72·67 (70·19–75·43)	64·25 (61·24–67·37)	77·62 (75·38–79·86)	67·24 (63·57–70·35)	74·91 (72·81–76·87)	65·99 (62·86–68·76)	77·37 (74·86–79·95)	66·90 (63·25–70·24)	73·88 (71·52–76·20)	65·02 (62·01–67·86)
		Papua New Guinea	57·55 (55·23–59·80)	49·98 (47·04–52·65)	54·45 (52·17–56·65)	48·22 (45·54–50·61)	58·93 (55·93–61·58)	51·16 (47·90–54·17)	56·57 (53·58–59·29)	50·03 (46·91–52·97)	62·17 (59·37–64·96)	53·81 (50·34–56·94)	59·50 (56·53–62·33)	52·50 (49·24–55·40)
		Samoa	72·32 (70·28–74·30)	62·82 (59·73–65·93)	65·84 (63·47–67·86)	58·20 (55·27–60·85)	73·62 (71·80–75·57)	63·79 (60·62–66·81)	68·98 (67·00–70·99)	60·94 (58·20–63·63)	74·00 (71·99–76·12)	64·09 (60·85–67·24)	69·90 (67·94–72·24)	61·73 (58·95–64·39)
		Solomon Islands	61·76 (59·54–64·33)	53·80 (50·92–56·89)	58·55 (56·42–61·01)	52·15 (49·75–54·77)	62·15 (59·76–64·69)	54·07 (51·07–56·93)	60·06 (57·82–62·35)	53·36 (50·66–55·92)	64·24 (61·49–66·86)	55·79 (52·73–58·68)	61·91 (59·31–64·32)	54·85 (52·12–57·46)
		Tonga	70·12 (67·56–72·67)	60·50 (57·17–63·85)	65·71 (63·10–68·33)	58·15 (55·13–61·11)	72·43 (70·40–74·34)	62·32 (58·91–65·47)	66·85 (64·60–69·05)	59·07 (56·15–61·55)	73·33 (70·85–75·64)	63·03 (59·33–66·31)	67·50 (65·23–70·07)	59·46 (56·61–62·26)
		Vanuatu	63·86 (61·77–66·01)	55·35 (52·25–58·43)	59·86 (57·86–61·86)	53·15 (50·44–55·83)	63·97 (61·41–66·27)	55·50 (52·38–58·48)	60·75 (58·57–62·89)	54·04 (51·41–56·65)	65·68 (62·77–68·20)	56·81 (53·52–60·12)	62·22 (59·66–64·66)	55·23 (52·22–58·05)
**North Africa and Middle East**			**68·93 (68·42–69·38)**	**57·92 (54·77–60·69)**	**65·01 (64·43–65·60)**	**56·14 (53·49–58·51)**	**73·19 (72·67–73·67)**	**61·72 (58·38–64·77)**	**69·13 (68·46–69·80)**	**59·90 (57·22–62·30)**	**75·59 (74·89–76·29)**	**63·66 (60·17–66·71)**	**70·90 (70·05–71·78)**	**61·41 (58·64–63·99)**
	Afghanistan		51·35 (49·83–52·92)	42·97 (40·24–45·55)	51·87 (50·14–53·90)	44·20 (41·28–46·73)	54·51 (53·00–55·97)	46·03 (43·31–48·59)	53·80 (51·93–55·70)	46·34 (43·59–49·01)	59·16 (57·45–60·78)	49·95 (46·99–52·73)	56·83 (54·45–59·11)	49·11 (46·21–51·87)
	Algeria		72·56 (71·34–73·76)	61·07 (57·66–64·19)	69·44 (68·19–70·72)	60·04 (57·20–62·71)	76·85 (75·62–77·68)	64·85 (61·39–68·08)	74·42 (73·29–75·58)	64·42 (61·43–67·15)	78·41 (77·46–79·34)	66·11 (62·60–69·36)	76·43 (75·06–77·66)	66·04 (62·82–68·90)
	Bahrain		70·99 (69·28–72·59)	59·71 (56·49–63·00)	68·54 (66·94–70·18)	59·52 (56·73–62·36)	74·94 (73·50–76·48)	62·73 (59·17–66·14)	72·76 (71·18–74·39)	62·85 (59·79–65·88)	77·81 (75·72–80·15)	65·01 (61·16–68·83)	75·99 (73·45–78·51)	65·52 (61·84–68·99)
	Egypt		67·29 (66·32–68·21)	56·84 (53·88–59·56)	63·32 (62·42–64·29)	55·04 (52·44–57·31)	72·89 (72·08–73·70)	61·63 (58·41–64·61)	67·66 (66·77–68·54)	59·00 (56·40–61·49)	74·98 (73·15–76·99)	63·54 (60·05–66·80)	69·44 (67·60–71·52)	60·64 (57·89–63·40)
	Iran		71·07 (68·91–73·37)	59·86 (56·10–63·19)	65·24 (62·58–68·12)	56·37 (53·08–59·74)	75·20 (72·96–77·42)	63·59 (59·94–66·96)	70·75 (68·00–73·35)	61·37 (57·97–64·66)	78·38 (76·58–80·58)	66·03 (62·27–69·57)	73·78 (71·23–76·22)	63·72 (60·16–66·85)
	Iraq		67·58 (65·61–69·71)	56·63 (53·29–59·79)	64·75 (62·05–67·37)	55·19 (51·77–58·30)	66·88 (64·23–69·42)	56·47 (52·82–59·86)	60·90 (57·38–65·04)	52·54 (48·76–56·43)	70·48 (67·37–73·54)	59·26 (55·41–62·99)	64·83 (61·47–69·19)	55·76 (52·01–59·74)
	Jordan		72·58 (70·91–74·84)	60·94 (57·41–64·21)	71·03 (69·24–72·57)	61·11 (58·15–63·85)	74·23 (71·88–76·82)	62·75 (59·00–66·31)	73·34 (70·65–75·64)	63·51 (60·19–66·80)	77·99 (74·85–81·02)	65·70 (61·49–69·46)	74·71 (71·39–78·25)	64·59 (60·97–68·43)
	Kuwait		76·70 (74·82–78·73)	65·00 (61·29–68·33)	74·54 (72·38–76·73)	64·65 (61·43–67·77)	77·12 (75·72–78·44)	65·38 (61·84–68·56)	76·93 (75·01–78·65)	66·54 (63·23–69·57)	79·47 (76·63–82·70)	67·37 (63·50–71·31)	79·96 (76·38–83·60)	69·01 (65·03–73·34)
	Lebanon		71·92 (70·05–74·03)	60·87 (57·53–64·06)	66·38 (64·58–68·79)	57·36 (54·50–60·39)	78·72 (77·82–79·96)	66·52 (62·98–69·83)	77·22 (75·61–78·85)	66·63 (63·32–69·56)	81·42 (79·91–82·87)	68·66 (64·94–72·23)	78·79 (77·11–80·32)	67·94 (64·59–70·98)
	Libya		74·68 (73·33–75·82)	62·95 (59·53–66·07)	71·59 (70·34–72·98)	61·97 (59·12–64·75)	77·01 (75·27–78·13)	65·03 (61·54–68·16)	73·87 (72·63–75·29)	64·02 (60·99–66·73)	77·59 (76·21–78·74)	65·47 (61·78–68·74)	72·62 (71·01–74·65)	63·00 (59·92–65·82)
	Morocco		68·70 (67·60–69·73)	57·76 (54·46–60·75)	65·69 (64·85–66·57)	56·74 (54·04–59·15)	73·80 (72·47–74·96)	62·22 (58·80–65·42)	71·16 (70·12–72·08)	61·48 (58·51–64·21)	76·44 (75·14–77·71)	64·38 (60·74–67·77)	73·55 (72·48–74·54)	63·58 (60·60–66·20)
	Palestine		71·78 (69·66–73·66)	61·01 (57·61–64·21)	70·13 (68·34–72·03)	60·77 (57·75–63·60)	74·19 (73·77–74·60)	63·12 (59·87–65·98)	69·54 (69·07–69·99)	60·50 (57·83–62·89)	73·48 (72·75–74·22)	62·56 (59·49–65·38)	70·23 (69·47–71·02)	61·05 (58·38–63·44)
	Oman		71·89 (70·46–73·25)	60·59 (57·11–63·71)	69·50 (68·12–70·81)	59·95 (56·92–62·74)	78·21 (77·27–79·33)	66·25 (62·72–69·47)	74·05 (72·92–75·15)	64·33 (61·45–67·07)	79·93 (79·21–80·83)	67·64 (63·95–70·96)	75·28 (74·48–76·27)	65·29 (62·36–68·01)
	Qatar		75·25 (73·06–77·85)	63·35 (59·66–67·15)	74·82 (72·23–77·11)	64·23 (60·77–67·49)	78·98 (77·27–80·91)	66·20 (62·32–69·81)	75·74 (73·25–78·54)	65·28 (61·77–68·76)	81·82 (78·89–85·16)	68·54 (64·22–72·79)	79·00 (75·41–82·65)	67·94 (63·77–72·07)
	Saudi Arabia		73·52 (72·07–74·90)	61·62 (58·14–64·84)	70·62 (69·03–72·20)	61·15 (58·17–63·96)	74·85 (74·23–75·41)	63·49 (60·33–66·49)	73·41 (72·66–74·13)	64·07 (61·36–66·59)	78·67 (77·83–79·62)	66·61 (63·09–69·87)	75·97 (74·95–77·00)	66·21 (63·34–68·81)
	Sudan		59·53 (58·32–60·64)	50·03 (47·25–52·75)	57·60 (56·49–58·74)	49·47 (46·80–51·85)	66·70 (65·69–67·70)	56·23 (53·08–58·98)	63·67 (62·69–64·72)	54·90 (52·20–57·27)	70·29 (68·93–71·52)	59·15 (55·70–62·35)	66·37 (64·87–67·67)	57·23 (54·39–60·00)
	Syria		72·81 (71·69–73·94)	61·54 (58·19–64·52)	69·12 (68·12–70·07)	60·03 (57·31–62·60)	77·06 (76·30–77·70)	65·44 (61·99–68·51)	73·22 (72·43–73·99)	63·91 (61·04–66·43)	73·62 (69·06–78·25)	62·58 (57·77–67·20)	63·29 (56·34–71·29)	55·64 (49·19–62·44)
	Tunisia		74·12 (73·09–75·18)	62·92 (59·62–66·06)	69·36 (68·15–70·56)	60·38 (57·62–62·91)	78·82 (77·62–80·64)	67·00 (63·42–70·58)	73·67 (71·98–75·57)	64·07 (60·96–67·00)	80·46 (78·87–82·70)	68·32 (64·48–72·01)	74·58 (72·07–77·24)	65·02 (61·75–68·47)
	Turkey		73·48 (72·41–74·51)	61·46 (58·05–64·58)	66·84 (65·71–68·03)	57·77 (55·03–60·35)	80·56 (79·58–81·47)	67·67 (63·94–71·19)	73·90 (73·02–74·83)	64·15 (61·44–66·84)	82·33 (80·42–84·35)	69·20 (65·37–72·84)	75·84 (73·66–78·13)	65·94 (62·76–69·10)
	United Arab Emirates		73·80 (71·06–76·69)	62·23 (58·32–65·78)	71·73 (68·48–74·76)	61·87 (57·92–65·75)	78·57 (77·31–79·40)	66·33 (62·78–69·58)	75·06 (73·81–76·25)	64·89 (61·82–67·73)	78·64 (76·18–80·63)	66·53 (62·95–70·18)	74·52 (72·10–76·83)	64·62 (61·09–67·66)
	Yemen		58·84 (57·63–60·16)	48·28 (45·15–51·30)	58·57 (57·40–59·81)	49·39 (46·58–51·99)	65·68 (64·61–66·84)	54·20 (50·83–57·27)	65·37 (64·18–66·55)	55·43 (52·41–58·18)	67·94 (66·60–69·16)	56·24 (52·77–59·33)	65·51 (63·70–67·23)	55·81 (52·76–58·59)
**South Asia**			**59·80 (59·24–60·35)**	**50·89 (48·19–53·20)**	**58·46 (57·94–58·97)**	**50·96 (48·82–52·90)**	**66·30 (65·82–66·79)**	**56·41 (53·59–58·90)**	**63·48 (62·93–64·03)**	**55·24 (52·96–57·29)**	**70·59 (70·08–71·12)**	**60·00 (56·96–62·75)**	**67·11 (66·46–67·67)**	**58·43 (55·97–60·54)**
	Bangladesh		58·61 (57·32–59·87)	49·95 (47·23–52·37)	57·61 (56·57–58·73)	50·10 (47·67–52·26)	70·37 (69·36–71·27)	59·79 (56·76–62·56)	66·64 (65·79–67·55)	58·08 (55·57–60·33)	75·10 (73·49–76·44)	64·04 (60·88–67·11)	70·47 (68·82–72·04)	61·69 (58·94–64·30)
	Bhutan		59·12 (57·08–61·38)	50·11 (47·16–53·12)	60·68 (58·50–62·85)	52·49 (49·55–55·02)	71·21 (69·51–72·79)	60·17 (56·70–63·28)	69·12 (67·36–70·85)	59·86 (57·04–62·76)	75·91 (73·78–77·46)	64·06 (60·43–67·44)	72·23 (70·06–74·38)	62·56 (59·32–65·53)
	India		59·69 (59·02–60·35)	50·75 (48·01–53·17)	58·29 (57·68–58·87)	50·81 (48·69–52·79)	66·01 (65·38–66·59)	56·09 (53·27–58·68)	63·22 (62·59–63·80)	54·96 (52·71–57·10)	70·33 (69·59–71·03)	59·67 (56·62–62·44)	66·93 (66·24–67·56)	58·18 (55·72–60·37)
	Nepal		57·71 (56·74–58·64)	49·63 (47·28–51·88)	57·69 (56·67–58·72)	50·14 (47·90–52·23)	67·17 (66·20–68·21)	57·74 (54·95–60·24)	65·78 (64·76–66·74)	57·35 (54·84–59·72)	71·91 (71·07–72·81)	61·80 (58·81–64·42)	69·74 (68·49–71·04)	60·92 (58·30–63·39)
	Pakistan		62·48 (61·49–63·45)	53·50 (50·73–55·99)	62·41 (61·38–63·42)	54·57 (52·08–56·85)	64·74 (63·33–66·23)	55·57 (52·77–58·33)	63·35 (61·99–64·79)	55·58 (53·21–57·94)	68·89 (66·86–71·19)	59·09 (55·86–62·20)	66·44 (64·27–68·45)	58·23 (55·39–60·97)
**Sub-Saharan Africa**			**55·39 (54·92–55·80)**	**47·87 (45·54–49·89)**	**52·04 (51·48–52·68)**	**45·35 (43·39–47·24)**	**56·38 (55·83–56·89)**	**48·88 (46·67–50·96)**	**54·12 (53·55–54·65)**	**47·33 (45·35–49·21)**	**64·59 (63·91–65·26)**	**56·05 (53·55–58·32)**	**61·18 (60·30–62·02)**	**53·57 (51·23–55·82)**
	Southern sub-Saharan Africa		67·35 (66·60–68·16)	58·18 (55·61–60·63)	60·25 (59·31–61·19)	52·67 (50·49–54·85)	51·29 (50·07–52·53)	44·50 (42·44–46·63)	48·31 (47·41–49·22)	42·47 (40·68–44·15)	64·86 (63·79–65·96)	55·75 (53·05–58·36)	58·36 (57·38–59·33)	50·90 (48·68–53·04)
		Botswana	67·60 (65·60–69·84)	58·83 (55·88–61·79)	61·23 (58·34–64·21)	53·70 (50·51–56·85)	51·04 (45·54–56·94)	44·50 (39·85–49·30)	47·99 (44·54–52·03)	42·16 (38·85–45·79)	69·21 (64·51–78·50)	59·74 (54·95–66·73)	61·69 (58·43–67·01)	53·88 (50·27–58·28)
		Lesotho	64·20 (62·27–66·35)	55·55 (52·72–58·36)	56·59 (54·64–58·76)	49·82 (47·38–52·48)	45·45 (41·73–49·33)	39·46 (36·03–42·72)	41·01 (38·66–43·40)	36·16 (33·65–38·50)	53·67 (49·88–58·25)	46·37 (42·72–50·26)	47·13 (44·61–49·71)	41·46 (38·97–43·98)
		Namibia	64·69 (63·56–65·91)	56·44 (53·69–58·78)	58·32 (57·03–59·62)	51·36 (49·11–53·52)	54·71 (50·75–58·90)	47·83 (44·03–51·74)	50·34 (47·92–52·74)	44·40 (41·90–46·92)	69·31 (65·48–75·19)	60·14 (55·93–65·17)	60·39 (57·83–63·37)	53·00 (50·09–56·01)
		South Africa	68·05 (67·23–68·92)	58·59 (55·83–61·09)	60·72 (59·85–61·59)	52·98 (50·78–55·06)	52·28 (50·85–53·65)	45·25 (42·99–47·42)	49·72 (48·70–50·77)	43·60 (41·64–45·51)	65·51 (64·17–66·75)	56·09 (53·35–58·88)	59·24 (57·90–60·31)	51·47 (49·14–53·72)
		Swaziland	65·42 (63·35–67·49)	56·99 (54·16–59·75)	59·06 (56·88–61·39)	51·93 (49·38–54·50)	44·30 (40·61–48·56)	38·50 (35·00–42·26)	41·59 (38·83–44·65)	36·49 (33·84–39·30)	62·16 (57·33–68·37)	53·38 (48·49–58·49)	53·25 (50·19–57·12)	46·38 (43·19–49·67)
		Zimbabwe	64·36 (62·06–67·28)	56·31 (53·45–59·60)	59·02 (55·45–63·66)	51·98 (48·42–56·28)	47·07 (43·92–50·60)	41·38 (38·39–44·54)	44·34 (42·25–46·58)	39·39 (37·15–41·71)	61·87 (59·24–65·19)	54·11 (51·15–57·35)	56·66 (54·38–59·28)	50·19 (47·52–53·02)
	Western sub-Saharan Africa		55·57 (54·69–56·34)	47·67 (45·11–49·96)	53·32 (52·28–54·31)	46·21 (44·00–48·31)	58·25 (57·26–59·21)	50·20 (47·85–52·54)	56·00 (55·03–56·96)	48·79 (46·59–50·96)	64·77 (63·72–65·90)	55·98 (53·26–58·55)	61·89 (60·59–63·06)	54·12 (51·54–56·44)
		Benin	57·60 (56·50–58·70)	48·94 (46·09–51·36)	53·32 (52·04–54·64)	45·99 (43·44–48·17)	61·95 (60·46–63·47)	53·19 (50·16–55·83)	57·76 (56·51–59·08)	50·39 (48·08–52·79)	66·34 (64·76–68·10)	57·19 (54·17–60·02)	62·53 (60·91–64·06)	54·72 (52·08–57·28)
		Burkina Faso	52·09 (51·11–53·19)	44·26 (41·75–46·61)	49·12 (47·86–50·52)	41·95 (39·58–44·21)	56·81 (55·85–57·76)	48·86 (46·30–51·19)	54·41 (53·23–55·54)	47·20 (44·77–49·52)	62·06 (61·04–63·05)	53·68 (51·08–56·24)	59·51 (57·95–60·84)	52·17 (49·79–54·55)
		Cameroon	59·63 (58·32–61·10)	51·05 (48·24–53·59)	57·28 (55·81–58·81)	49·57 (47·05–51·91)	56·56 (54·54–58·87)	48·79 (46·01–51·46)	54·00 (52·22–55·70)	47·10 (44·57–49·48)	62·02 (59·34–65·18)	53·81 (50·69–57·12)	58·34 (56·01–60·59)	51·26 (48·34–54·11)
		Cape Verde	71·55 (70·49–72·79)	61·66 (58·62–64·50)	64·09 (62·76–65·53)	56·21 (53·78–58·70)	76·48 (74·84–78·29)	65·95 (62·49–69·20)	66·27 (64·07–69·43)	58·27 (55·46–61·31)	78·50 (77·22–80·25)	67·83 (64·48–71·09)	68·79 (66·61–71·11)	60·61 (57·69–63·48)
		Chad	54·43 (53·27–55·67)	46·62 (44·13–48·84)	52·10 (50·71–53·32)	45·17 (42·76–47·24)	55·55 (53·89–57·37)	47·63 (45·00–50·25)	52·59 (50·96–54·22)	45·68 (43·28–48·08)	61·36 (59·50–63·20)	52·71 (49·91–55·50)	58·32 (56·39–60·28)	50·74 (48·15–53·29)
		Côte d'Ivoire	57·68 (56·58–58·79)	49·17 (46·50–51·82)	52·39 (51·01–53·58)	45·37 (42·99–47·51)	56·04 (54·36–57·85)	48·52 (45·85–50·93)	52·09 (50·45–53·67)	45·65 (43·25–47·87)	62·29 (60·54–64·01)	53·92 (51·07–56·57)	57·72 (55·83–59·48)	50·60 (48·00–53·01)
		The Gambia	62·30 (60·68–64·02)	53·36 (50·28–56·14)	60·88 (59·15–62·62)	53·05 (50·22–55·72)	66·26 (64·59–68·06)	56·75 (53·72–59·73)	62·80 (61·35–64·29)	54·78 (51·92–57·28)	69·22 (67·62–71·33)	59·42 (56·30–62·46)	65·46 (63·97–66·94)	57·15 (54·20–59·62)
		Ghana	59·84 (58·36–61·44)	51·82 (49·16–54·38)	57·78 (56·18–59·37)	50·59 (48·06–53·14)	61·92 (60·12–63·95)	54·01 (51·22–56·79)	59·01 (57·79–60·31)	52·03 (49·63–54·16)	67·53 (65·97–69·68)	58·98 (56·19–61·86)	64·49 (62·75–66·08)	56·90 (54·34–59·35)
		Guinea	51·91 (50·53–53·30)	44·55 (42·05–46·92)	52·11 (50·71–53·57)	45·26 (42·89–47·43)	57·25 (55·74–58·97)	49·53 (47·03–51·96)	55·58 (53·83–57·33)	48·76 (46·21–51·09)	61·62 (59·52–64·00)	53·40 (50·48–56·36)	59·76 (57·32–62·13)	52·48 (49·61–55·35)
		Guinea-Bissau	51·89 (50·31–53·78)	44·89 (42·43–47·26)	46·57 (44·71–48·57)	40·83 (38·59–43·17)	56·06 (54·03–58·01)	48·66 (45·95–51·27)	51·56 (50·00–53·30)	45·36 (43·07–47·54)	61·37 (59·44–63·33)	53·32 (50·43–56·03)	56·30 (54·53–58·20)	49·60 (47·09–52·04)
		Liberia	51·76 (50·48–53·07)	43·77 (41·14–46·17)	47·99 (46·63–49·49)	40·93 (38·51–43·17)	58·82 (57·58–60·31)	49·98 (47·20–52·57)	58·52 (57·29–59·75)	49·98 (47·27–52·50)	64·82 (63·59–66·07)	55·40 (52·50–58·00)	64·00 (62·76–65·16)	55·19 (52·43–57·82)
		Mali	49·39 (48·14–50·66)	42·49 (40·11–44·73)	49·41 (48·04–50·70)	42·56 (40·19–44·83)	57·72 (56·20–59·37)	49·75 (47·12–52·25)	56·46 (54·86–58·11)	48·81 (46·21–51·40)	62·74 (60·01–65·49)	54·29 (51·16–57·53)	61·02 (58·09–63·77)	53·18 (49·98–56·44)
		Mauritania	60·41 (59·05–61·76)	52·16 (49·49–54·62)	59·50 (58·11–61·07)	51·87 (49·30–54·28)	65·99 (64·25–68·11)	57·15 (54·23–59·95)	66·83 (64·96–68·67)	58·11 (55·20–60·91)	70·21 (67·56–73·27)	60·85 (57·43–64·22)	70·29 (67·75–73·20)	61·28 (57·96–64·65)
		Niger	48·07 (46·57–49·48)	41·61 (39·47–43·84)	46·19 (44·61–47·75)	40·45 (38·31–42·43)	57·00 (55·24–58·62)	49·47 (46·71–51·86)	55·43 (53·62–57·32)	48·68 (46·27–51·07)	62·83 (60·16–65·50)	54·70 (51·47–57·94)	60·60 (58·25–62·87)	53·42 (50·58–56·30)
		Nigeria	55·80 (53·93–57·43)	47·83 (45·05–50·36)	53·99 (51·98–55·95)	46·72 (44·09–49·35)	58·29 (56·34–60·30)	50·06 (47·39–52·97)	56·69 (54·87–58·62)	49·22 (46·60–51·93)	66·41 (64·30–69·14)	57·17 (53·86–60·50)	63·69 (61·16–66·21)	55·46 (52·16–58·36)
		São Tomé and Príncipe	64·55 (62·95–66·14)	56·13 (53·44–58·78)	61·94 (60·46–63·55)	54·06 (51·51–56·55)	68·04 (66·93–69·14)	59·38 (56·77–61·81)	65·63 (64·35–66·88)	57·37 (54·72–59·76)	72·09 (70·52–73·93)	62·86 (59·84–65·70)	69·04 (66·71–71·46)	60·40 (57·08–63·43)
		Senegal	59·13 (57·93–60·29)	51·16 (48·64–53·57)	55·97 (54·99–56·95)	49·06 (46·73–51·08)	63·73 (62·67–64·93)	55·34 (52·58–57·81)	60·56 (59·49–61·69)	53·17 (50·79–55·42)	67·78 (66·59–68·83)	58·95 (56·18–61·49)	64·64 (63·32–65·97)	56·81 (54·17–59·31)
		Sierra Leone	52·78 (51·41–54·15)	45·44 (42·94–47·75)	48·43 (46·82–50·02)	42·07 (39·83–44·17)	54·14 (52·72–55·61)	46·80 (44·24–49·05)	51·62 (50·37–52·85)	44·94 (42·80–47·02)	60·19 (58·38–62·14)	52·30 (49·54–55·01)	58·11 (56·42–59·82)	50·98 (48·48–53·42)
		Togo	58·64 (57·47–59·92)	50·53 (47·88–52·99)	56·13 (54·90–57·39)	49·04 (46·54–51·27)	59·12 (57·12–61·53)	51·10 (48·41–53·75)	54·86 (53·29–56·46)	48·25 (45·93–50·52)	65·08 (63·58–66·82)	56·63 (53·68–59·26)	60·11 (58·22–61·70)	53·13 (50·60–55·49)
	Eastern sub-Saharan Africa		52·92 (52·39–53·47)	46·24 (44·24–48·02)	49·46 (48·81–50·17)	43·47 (41·58–45·13)	56·64 (55·91–57·36)	49·56 (47·39–51·57)	54·57 (53·86–55·19)	48·01 (46·07–49·81)	65·08 (64·13–66·09)	56·97 (54·43–59·30)	61·72 (60·63–62·80)	54·35 (52·04–56·58)
		Burundi	49·28 (47·21–51·62)	43·59 (41·19–46·03)	46·69 (44·34–49·16)	41·49 (39·09–43·99)	55·18 (53·18–57·01)	48·71 (46·26–51·06)	53·46 (51·54–55·47)	47·33 (44·70–49·70)	61·51 (58·70–64·10)	54·51 (51·44–57·29)	59·20 (56·03–62·28)	52·57 (49·11–55·76)
		Comoros	59·46 (57·78–61·52)	51·92 (49·20–54·61)	57·80 (55·88–59·73)	50·47 (47·88–52·97)	65·30 (63·79–67·10)	57·14 (54·36–59·97)	63·78 (62·30–65·32)	55·98 (53·42–58·63)	68·52 (66·78–70·86)	60·05 (57·27–63·16)	66·61 (64·38–68·77)	58·57 (55·52–61·31)
		Djibouti	64·10 (62·77–65·15)	55·99 (53·27–58·39)	58·43 (57·21–59·67)	51·61 (49·41–53·77)	64·36 (61·78–67·15)	56·34 (53·15–59·62)	59·51 (57·39–61·57)	52·59 (49·86–55·17)	68·80 (66·11–71·85)	60·24 (56·93–63·65)	64·67 (62·50–66·70)	57·03 (54·20–59·80)
		Eritrea	53·74 (52·30–55·22)	47·10 (44·78–49·34)	50·96 (49·44–52·49)	45·01 (42·76–47·10)	61·51 (59·71–63·42)	54·15 (51·29–56·75)	60·05 (58·00–62·07)	52·98 (50·39–55·63)	64·53 (62·75–66·62)	56·84 (54·01–59·55)	62·88 (60·62–65·07)	55·54 (52·73–58·26)
		Ethiopia	48·89 (47·64–50·20)	43·00 (40·98–44·98)	44·37 (43·16–45·76)	39·31 (37·54–41·04)	57·30 (55·68–59·03)	50·56 (48·24–52·87)	56·81 (55·05–58·31)	50·17 (47·86–52·43)	66·53 (64·21–68·96)	58·67 (55·72–61·51)	64·74 (62·10–67·59)	57·20 (54·14–60·45)
		Kenya	62·63 (61·99–63·30)	55·07 (52·71–57·15)	60·25 (59·41–61·07)	53·14 (50·93–55·15)	59·91 (59·18–60·60)	52·60 (50·41–54·67)	57·04 (56·37–57·71)	50·40 (48·34–52·22)	69·03 (68·24–69·88)	60·30 (57·58–62·64)	64·72 (63·89–65·56)	56·94 (54·49–59·05)
		Madagascar	56·76 (55·42–58·23)	49·55 (47·22–51·91)	54·43 (53·13–55·73)	47·74 (45·56–49·87)	61·40 (59·57–63·44)	53·79 (50·81–56·51)	59·41 (57·47–61·41)	52·19 (49·62–54·76)	63·88 (60·78–67·84)	56·22 (52·80–59·92)	61·51 (58·34–64·83)	54·29 (51·18–57·82)
		Malawi	49·89 (48·02–51·91)	43·53 (41·08–46·03)	47·56 (44·19–51·16)	41·45 (38·16–44·72)	49·75 (46·64–53·37)	43·49 (40·30–46·97)	47·18 (44·96–49·62)	41·39 (39·05–44·13)	62·59 (59·81–65·90)	54·63 (51·37–57·95)	57·90 (55·27–60·52)	50·94 (48·22–53·98)
		Mozambique	52·80 (51·72–53·94)	45·66 (43·25–47·89)	48·51 (47·27–49·70)	42·25 (40·17–44·09)	54·89 (51·81–58·00)	47·39 (44·02–50·59)	50·33 (48·12–52·56)	43·91 (41·18–46·35)	62·88 (60·39–65·36)	54·32 (51·22–57·53)	57·05 (54·88–59·18)	49·78 (46·92–52·53)
		Rwanda	50·78 (49·17–52·31)	44·72 (42·38–46·96)	47·51 (45·55–49·50)	42·13 (39·82–44·32)	61·04 (59·22–62·93)	53·38 (50·75–55·88)	57·93 (56·51–59·53)	50·93 (48·55–53·13)	69·32 (67·21–71·54)	60·74 (57·78–63·78)	65·98 (63·86–67·95)	58·02 (55·04–60·77)
		Somalia	51·72 (50·09–53·38)	45·34 (42·86–47·64)	49·60 (47·49–51·75)	43·81 (41·26–46·38)	54·55 (52·80–56·31)	47·93 (45·51–50·47)	53·54 (51·26–55·80)	47·33 (44·72–50·09)	57·74 (55·75–59·45)	50·75 (48·08–53·18)	56·64 (54·11–59·07)	50·10 (47·03–52·87)
		South Sudan	53·28 (51·20–55·51)	44·83 (41·62–47·86)	49·85 (47·40–52·59)	42·34 (39·34–45·54)	57·77 (54·98–60·99)	49·35 (45·93–52·82)	55·76 (53·13–58·60)	47·94 (44·83–51·03)	60·72 (57·90–63·93)	52·40 (49·04–55·82)	58·71 (56·05–61·53)	50·96 (47·77–54·05)
		Tanzania	55·98 (54·76–57·26)	48·69 (46·28–51·04)	53·74 (52·07–55·35)	47·08 (44·67–49·47)	56·72 (54·77–59·54)	49·54 (46·90–52·22)	55·30 (53·82–56·98)	48·68 (46·36–51·06)	66·05 (64·28–68·33)	57·79 (54·94–60·76)	62·59 (60·50–64·45)	55·33 (52·61–57·81)
		Uganda	52·07 (50·38–53·44)	44·88 (42·39–47·10)	46·34 (43·41–48·89)	40·17 (37·23–43·06)	55·59 (53·98–57·23)	48·16 (45·69–50·66)	51·84 (50·37–53·29)	45·29 (42·89–47·31)	64·75 (62·93–67·01)	56·33 (53·55–59·24)	59·77 (57·99–61·40)	52·45 (50·10–54·90)
		Zambia	52·33 (50·38–54·59)	45·94 (43·52–48·33)	52·21 (49·34–54·78)	45·87 (42·88–48·71)	48·21 (45·39–51·35)	42·34 (39·62–45·29)	45·23 (43·18–47·31)	40·12 (37·88–42·23)	61·95 (58·22–66·84)	54·14 (50·45–58·71)	55·58 (52·54–59·34)	49·10 (46·12–52·44)
	Central sub-Saharan Africa		54·41 (53·25–55·53)	46·46 (43·99–48·74)	50·83 (49·71–51·99)	43·71 (41·49–45·86)	56·35 (55·20–57·49)	48·42 (45·79–50·67)	54·80 (53·60–55·95)	47·29 (44·88–49·53)	62·80 (61·19–64·43)	54·20 (51·34–56·76)	60·62 (58·87–62·28)	52·58 (49·77–55·26)
		Angola	52·16 (49·57–55·16)	45·20 (42·37–48·22)	48·27 (45·63–51·05)	42·20 (39·52–45·23)	57·13 (54·31–60·11)	49·77 (46·75–52·71)	56·77 (53·90–59·62)	49·56 (46·60–52·46)	65·37 (61·78–68·70)	56·85 (53·28–60·26)	63·94 (60·34–67·08)	55·76 (52·30–59·13)
		Central African Republic	51·04 (48·84–53·26)	43·80 (40·92–46·39)	45·38 (43·16–47·67)	39·24 (36·70–41·72)	47·44 (44·14–51·09)	41·04 (37·74–44·37)	43·21 (40·31–46·22)	37·65 (34·73–40·63)	52·59 (48·76–56·64)	45·60 (41·97–49·45)	47·91 (44·46–51·58)	41·91 (38·62–45·46)
		Congo (Brazzaville)	55·96 (53·78–58·10)	48·26 (45·37–51·06)	52·19 (50·02–54·61)	45·47 (42·70–48·24)	56·61 (54·64–58·58)	48·95 (46·20–51·56)	58·33 (56·48–60·14)	50·60 (47·97–53·23)	62·35 (59·54–65·61)	53·99 (50·40–57·77)	63·62 (60·10–67·33)	55·47 (51·93–59·12)
		Democratic Republic of the Congo	55·32 (53·74–56·81)	46·96 (44·23–49·57)	52·33 (50·71–53·96)	44·65 (42·09–47·12)	56·75 (55·28–58·22)	48·53 (45·70–51·08)	55·03 (53·48–56·45)	47·24 (44·61–49·74)	62·72 (60·80–64·53)	53·99 (50·96–56·80)	60·44 (58·33–62·42)	52·26 (49·34–55·04)
		Equatorial Guinea	49·82 (47·16–52·85)	43·11 (40·10–46·00)	46·54 (43·66–49·72)	40·62 (37·58–43·52)	59·26 (54·70–64·65)	51·26 (47·01–55·97)	58·66 (54·71–63·17)	50·91 (46·94–55·13)	66·55 (62·28–71·43)	57·44 (53·04–61·96)	64·49 (60·25–69·11)	55·97 (51·95–60·33)
		Gabon	62·90 (61·23–64·49)	54·14 (51·30–56·75)	56·02 (54·28–57·83)	49·01 (46·57–51·43)	61·32 (59·50–63·25)	52·87 (50·05–55·57)	59·71 (57·34–61·77)	52·16 (49·40–54·83)	68·31 (65·83–71·18)	58·73 (55·38–62·22)	64·90 (61·35–68·37)	56·59 (52·95–60·04)

Data in parentheses are 95% uncertainty intervals. To download the data in this table, please visit the Global Health Data Exchange. GBD=Global Burden of Disease. HALE=healthy life expectancy. SDI=Socio-demographic index.

**Table 3:** Global, regional, and GBD location-specific life expectancy and HALE at age 65 years, by sex, in 1990, 2006, and 2016

			**1990, at age 65 years**				**2006, at age 65 years**				**2016, at age 65 years**			
			Females		Males		Females		Males		Females		Males	
			Life expectancy	HALE	Life expectancy	HALE	Life expectancy	HALE	Life expectancy	HALE	Life expectancy	HALE	Life expectancy	HALE
**Global**			**15·90 (15·82–15·98)**	**11·92 (10·88–12·89)**	**13·33 (13·26–13·39)**	**10·09 (9·22–10·87)**	**17·27 (17·19–17·35)**	**12·93 (11·74–13·98)**	**14·69 (14·62–14·75)**	**11·10 (10·13–11·97)**	**18·57 (18·37–18·72)**	**13·88 (12·57–15·02)**	**15·72 (15·61–15·83)**	**11·87 (10·83–12·80)**
	High SDI		18·57 (18·50–18·63)	14·06 (12·85–15·16)	14·79 (14·73–14·84)	11·22 (10·27–12·08)	20·72 (20·67–20·78)	15·69 (14·31–16·93)	17·22 (17·17–17·28)	13·00 (11·86–14·03)	21·67 (21·53–21·81)	16·38 (14·91–17·68)	18·37 (18·22–18·50)	13·86 (12·65–14·98)
	High-middle SDI		16·31 (16·07–16·54)	12·15 (11·02–13·22)	13·35 (13·16–13·54)	10·09 (9·20–10·92)	17·39 (17·15–17·62)	13·00 (11·78–14·08)	14·27 (14·06–14·45)	10·81 (9·86–11·68)	19·19 (18·36–19·89)	14·32 (12·85–15·71)	15·84 (15·39–16·28)	12·01 (10·90–13·06)
	Middle SDI		14·81 (14·63–14·98)	11·25 (10·32–12·13)	12·64 (12·50–12·78)	9·78 (9·00–10·50)	16·55 (16·43–16·67)	12·48 (11·38–13·50)	14·09 (13·98–14·21)	10·84 (9·96–11·66)	18·19 (18·02–18·35)	13·68 (12·48–14·77)	15·01 (14·86–15·17)	11·50 (10·55–12·33)
	Low-middle SDI		13·08 (12·90–13·28)	9·47 (8·51–10·37)	12·48 (12·32–12·64)	9·15 (8·28–9·96)	14·35 (14·16–14·54)	10·40 (9·36–11·34)	13·24 (13·10–13·39)	9·74 (8·83–10·58)	15·50 (15·26–15·73)	11·24 (10·13–12·27)	13·95 (13·75–14·14)	10·27 (9·28–11·13)
	Low SDI		11·65 (11·45–11·86)	8·54 (7·67–9·29)	11·75 (11·52–11·97)	8·59 (7·72–9·42)	12·29 (12·02–12·58)	9·08 (8·19–9·91)	12·43 (12·20–12·67)	9·17 (8·24–9·97)	13·32 (13·00–13·64)	9·90 (8·94–10·78)	13·22 (12·89–13·54)	9·80 (8·82–10·69)
	**High income**		**18·77 (18·71–18·84)**	**14·28 (13·06–15·39)**	**14·96 (14·90–15·01)**	**11·40 (10·45–12·27)**	**20·89 (20·83–20·95)**	**15·88 (14·51–17·12)**	**17·35 (17·30–17·40)**	**13·16 (12·02–14·18)**	**21·80 (21·66–21·94)**	**16·55 (15·09–17·85)**	**18·47 (18·32–18·60)**	**14·00 (12·79–15·11)**
High-income North America			19·09 (19·03–19·16)	14·49 (13·27–15·62)	15·17 (15·11–15·24)	11·46 (10·48–12·36)	20·05 (19·99–20·12)	15·06 (13·71–16·29)	17·33 (17·28–17·40)	12·91 (11·73–13·98)	20·74 (20·59–20·89)	15·46 (14·05–16·73)	18·19 (18·05–18·34)	13·48 (12·23–14·63)
		Canada	19·69 (19·47–19·89)	15·30 (14·07–16·43)	15·50 (15·31–15·70)	12·03 (11·03–12·93)	21·08 (20·86–21·27)	16·28 (15·00–17·49)	17·92 (17·75–18·13)	13·82 (12·68–14·86)	21·96 (21·66–22·27)	16·91 (15·50–18·21)	19·12 (18·82–19·44)	14·71 (13·46–15·88)
		Greenland	11·30 (10·69–11·92)	8·66 (7·82–9·51)	8·95 (8·51–9·40)	6·83 (6·17–7·45)	11·88 (11·27–12·47)	9·10 (8·25–9·92)	10·70 (10·19–11·17)	8·20 (7·47–8·92)	14·20 (12·77–15·95)	10·89 (9·51–12·36)	11·89 (10·54–13·29)	9·14 (7·93–10·46)
		USA	19·04 (18·97–19·11)	14·41 (13·19–15·55)	15·14 (15·07–15·20)	11·41 (10·43–12·30)	19·94 (19·88–20·01)	14·93 (13·57–16·16)	17·27 (17·21–17·33)	12·80 (11·62–13·88)	20·60 (20·44–20·76)	15·30 (13·89–16·59)	18·09 (17·93–18·25)	13·34 (12·08–14·49)
	Australasia		18·95 (18·79–19·12)	14·66 (13·46–15·73)	15·10 (14·95–15·25)	11·65 (10·69–12·53)	21·35 (21·21–21·49)	16·52 (15·14–17·76)	18·31 (18·17–18·46)	14·11 (12·94–15·15)	22·10 (21·65–22·56)	17·07 (15·62–18·38)	19·37 (18·91–19·84)	14·92 (13·66–16·08)
		Australia	19·05 (18·88–19·25)	14·73 (13·54–15·81)	15·18 (15·01–15·35)	11·71 (10·75–12·59)	21·51 (21·34–21·68)	16·63 (15·26–17·91)	18·40 (18·24–18·58)	14·17 (12·98–15·23)	22·22 (21·67–22·74)	17·16 (15·72–18·49)	19·47 (18·93–19·96)	14·98 (13·69–16·17)
		New Zealand	18·43 (18·18–18·66)	14·29 (13·11–15·34)	14·71 (14·50–14·92)	11·36 (10·40–12·22)	20·52 (20·29–20·73)	15·95 (14·61–17·11)	17·83 (17·62–18·03)	13·81 (12·64–14·85)	21·48 (20·65–22·40)	16·64 (15·19–18·03)	18·90 (18·08–19·77)	14·60 (13·30–15·87)
High-income Asia Pacific			19·46 (19·31–19·61)	15·03 (13·80–16·15)	15·65 (15·51–15·78)	11·91 (10·90–12·82)	22·73 (22·57–22·88)	17·47 (15·99–18·77)	17·93 (17·80–18·07)	13·50 (12·30–14·59)	23·79 (23·27–24·24)	18·30 (16·64–19·74)	19·20 (18·75–19·61)	14·51 (13·22–15·73)
	Brunei		16·84 (15·98–17·54)	12·75 (11·48–13·92)	14·52 (14·31–14·78)	10·70 (9·68–11·64)	18·67 (18·25–19·03)	14·26 (13·01–15·41)	15·85 (15·56–16·19)	11·78 (10·69–12·79)	18·88 (18·18–20·19)	14·50 (13·16–15·79)	15·88 (15·05–17·40)	11·88 (10·61–13·13)
	Japan		19·96 (19·92–20·00)	15·43 (14·15–16·55)	16·22 (16·19–16·25)	12·36 (11·32–13·29)	23·23 (23·17–23·28)	17·89 (16·38–19·22)	18·24 (18·21–18·28)	13·75 (12·53–14·83)	24·24 (24·07–24·40)	18·67 (17·07–20·03)	19·53 (19·35–19·70)	14·78 (13·50–15·95)
	Singapore		17·64 (16·49–18·82)	13·74 (12·40–15·01)	14·65 (13·72–15·61)	11·26 (10·13–12·42)	21·50 (20·48–22·49)	16·78 (15·30–18·19)	17·71 (16·77–18·66)	13·67 (12·45–14·94)	23·33 (21·62–25·20)	18·16 (16·31–20·02)	19·70 (17·89–21·49)	15·13 (13·37–16·82)
	South Korea		16·70 (15·82–17·56)	12·82 (11·56–14·06)	12·36 (11·66–13·12)	9·33 (8·35–10·31)	20·05 (19·28–20·86)	15·26 (13·84–16·68)	16·20 (15·45–17·11)	12·11 (10·94–13·41)	21·67 (19·44–23·94)	16·62 (14·54–18·80)	17·52 (15·45–20·08)	13·20 (11·32–15·13)
Western Europe			18·48 (18·37–18·59)	13·98 (12·75–15·11)	14·70 (14·61–14·79)	11·25 (10·32–12·10)	20·81 (20·71–20·91)	15·80 (14·41–17·04)	17·26 (17·17–17·36)	13·23 (12·12–14·24)	21·76 (21·56–21·96)	16·54 (15·08–17·87)	18·49 (18·29–18·69)	14·21 (13·00–15·28)
	Andorra		20·84 (19·08–22·79)	15·59 (13·63–17·52)	16·54 (15·62–17·99)	12·54 (11·23–13·98)	23·19 (21·49–24·44)	17·38 (15·50–19·13)	18·33 (17·43–19·29)	13·94 (12·51–15·26)	23·06 (20·90–24·50)	17·31 (15·33–19·16)	18·46 (17·43–20·09)	14·08 (12·52–15·58)
	Austria		17·88 (17·71–18·06)	13·60 (12·43–14·67)	14·36 (14·20–14·51)	10·97 (9·99–11·81)	20·48 (20·31–20·63)	15·61 (14·25–16·81)	17·16 (17·00–17·32)	13·07 (11·92–14·08)	21·45 (20·98–21·95)	16·35 (14·86–17·69)	18·41 (18·01–18·90)	14·08 (12·82–15·30)
	Belgium		18·47 (18·16–18·74)	13·84 (12·59–14·99)	14·29 (14·00–14·59)	10·86 (9·84–11·73)	20·49 (20·24–20·77)	15·22 (13·79–16·51)	16·87 (16·60–17·15)	12·70 (11·54–13·74)	21·38 (20·63–22·22)	16·00 (14·41–17·50)	17·99 (17·22–18·72)	13·62 (12·27–14·90)
	Cyprus		15·67 (15·34–15·99)	11·88 (10·80–12·88)	14·03 (13·70–14·36)	10·77 (9·81–11·61)	18·99 (18·64–19·34)	14·41 (13·09–15·59)	16·52 (16·19–16·84)	12·64 (11·53–13·62)	20·17 (19·74–20·58)	15·38 (14·04–16·67)	17·44 (17·01–17·92)	13·39 (12·22–14·49)
	Denmark		17·97 (17·41–18·50)	13·58 (12·33–14·73)	14·27 (13·81–14·80)	10·99 (10·00–11·87)	19·09 (18·53–19·65)	14·47 (13·09–15·70)	16·33 (15·86–16·81)	12·50 (11·38–13·56)	20·76 (19·77–21·83)	15·65 (14·08–17·10)	18·01 (17·04–18·92)	13·77 (12·45–15·05)
	Finland		17·74 (17·50–17·98)	13·32 (12·13–14·43)	13·76 (13·56–13·97)	10·37 (9·43–11·23)	20·74 (20·51–20·94)	15·37 (13·87–16·74)	16·73 (16·52–16·95)	12·42 (11·26–13·46)	21·99 (21·44–22·62)	16·49 (14·87–18·00)	18·39 (17·82–18·97)	13·86 (12·61–15·07)
	France		20·15 (19·94–20·34)	15·31 (13·98–16·52)	15·72 (15·52–15·93)	12·08 (11·05–13·01)	22·25 (22·04–22·44)	17·06 (15·60–18·34)	17·91 (17·71–18·15)	13·85 (12·66–14·89)	23·22 (22·83–23·66)	17·74 (16·17–19·19)	19·24 (18·87–19·63)	14·87 (13·56–16·01)
	Germany		17·68 (17·28–18·06)	13·32 (12·06–14·47)	14·03 (13·65–14·41)	10·67 (9·69–11·56)	20·21 (19·87–20·57)	15·31 (13·94–16·58)	16·75 (16·41–17·13)	12·79 (11·69–13·86)	21·10 (20·46–21·82)	15·98 (14·47–17·42)	17·93 (17·27–18·62)	13·69 (12·43–14·90)
	Greece		18·02 (17·79–18·26)	13·71 (12·55–14·78)	15·58 (15·35–15·83)	11·98 (10·98–12·88)	20·24 (20·03–20·46)	15·40 (14·10–16·59)	17·42 (17·19–17·64)	13·37 (12·24–14·38)	20·98 (20·42–21·55)	16·07 (14·70–17·39)	18·34 (17·72–18·99)	14·12 (12·91–15·28)
	Iceland		19·13 (18·70–19·55)	14·48 (13·19–15·67)	16·16 (15·82–16·53)	12·37 (11·30–13·32)	20·76 (20·29–21·27)	15·73 (14·34–16·95)	18·31 (17·99–18·66)	14·02 (12·79–15·10)	21·35 (20·53–22·14)	16·24 (14·78–17·62)	18·98 (18·37–19·56)	14·60 (13·28–15·89)
	Ireland		16·89 (16·46–17·33)	12·86 (11·69–13·94)	13·36 (13·00–13·78)	10·28 (9·37–11·12)	19·70 (19·27–20·16)	14·96 (13·66–16·21)	16·66 (16·26–17·04)	12·79 (11·69–13·85)	21·03 (19·99–22·10)	16·02 (14·42–17·48)	18·13 (17·14–19·09)	13·96 (12·61–15·28)
	Israel		16·81 (15·99–17·61)	12·74 (11·45–13·98)	15·08 (14·39–15·87)	11·53 (10·39–12·59)	20·00 (19·21–20·82)	15·12 (13·63–16·54)	17·63 (16·86–18·41)	13·46 (12·15–14·68)	21·54 (20·02–22·96)	16·31 (14·53–17·97)	18·93 (17·36–20·55)	14·48 (12·89–16·15)
	Italy		18·91 (18·73–19·10)	14·31 (13·04–15·46)	15·08 (14·91–15·25)	11·53 (10·55–12·45)	21·36 (21·18–21·52)	16·26 (14·82–17·57)	17·57 (17·39–17·76)	13·50 (12·38–14·54)	21·94 (21·44–22·46)	16·77 (15·29–18·16)	18·60 (18·05–19·13)	14·34 (13·08–15·50)
	Luxembourg		17·75 (17·43–18·09)	13·25 (12·00–14·38)	14·01 (13·75–14·27)	10·51 (9·54–11·40)	20·82 (20·44–21·15)	15·60 (14·14–16·86)	17·44 (17·14–17·75)	13·13 (11·92–14·24)	21·49 (20·63–22·52)	16·16 (14·62–17·61)	18·95 (18·21–19·74)	14·32 (12·93–15·58)
	Malta		16·55 (15·73–17·38)	12·60 (11·35–13·85)	14·28 (13·61–14·99)	10·99 (9·95–12·06)	19·57 (18·79–20·34)	14·93 (13·49–16·22)	16·55 (15·89–17·23)	12·76 (11·64–13·80)	21·34 (19·94–22·91)	16·28 (14·58–18·01)	18·03 (16·76–19·40)	13·92 (12·45–15·45)
	Netherlands		18·96 (18·64–19·33)	14·26 (12·98–15·45)	14·43 (14·11–14·73)	10·98 (10·03–11·85)	20·17 (19·85–20·47)	15·21 (13·82–16·48)	16·73 (16·45–17·05)	12·72 (11·62–13·74)	21·38 (20·65–22·06)	16·12 (14·61–17·54)	18·11 (17·36–18·78)	13·79 (12·53–15·00)
	Norway		18·81 (18·47–19·16)	14·30 (13·04–15·45)	14·80 (14·50–15·15)	11·24 (10·21–12·17)	20·62 (20·28–20·92)	15·74 (14·38–16·96)	17·42 (17·12–17·70)	13·20 (12·02–14·27)	21·57 (20·67–22·52)	16·49 (14·89–17·88)	18·68 (17·94–19·52)	14·21 (12·90–15·60)
	Portugal		17·57 (17·36–17·79)	13·32 (12·17–14·41)	14·26 (14·04–14·47)	10·96 (9·99–11·76)	20·21 (19·95–20·46)	15·36 (13·97–16·55)	16·50 (16·25–16·75)	12·76 (11·70–13·73)	21·52 (21·01–22·08)	16·43 (14·99–17·78)	17·82 (17·32–18·37)	13·82 (12·60–14·92)
	Spain		19·26 (19·12–19·40)	14·71 (13·40–15·84)	15·59 (15·44–15·74)	12·07 (11·09–12·98)	21·58 (21·44–21·70)	16·53 (15·08–17·79)	17·57 (17·43–17·71)	13·65 (12·54–14·65)	22·82 (22·47–23·17)	17·57 (16·08–18·94)	19·17 (18·76–19·56)	14·99 (13·74–16·09)
	Sweden		19·05 (18·78–19·30)	14·38 (13·12–15·57)	15·34 (15·10–15·60)	11·77 (10·74–12·66)	20·58 (20·36–20·83)	15·53 (14·13–16·77)	17·58 (17·35–17·81)	13·36 (12·20–14·42)	21·37 (20·36–22·32)	16·05 (14·52–17·62)	18·64 (17·74–19·51)	14·12 (12·67–15·40)
	Switzerland		19·57 (19·09–20·04)	14·64 (13·24–15·95)	15·38 (14·93–15·84)	11·70 (10·61–12·72)	21·55 (21·10–21·98)	16·14 (14·60–17·55)	18·22 (17·74–18·69)	13·82 (12·60–15·03)	22·51 (20·50–24·40)	17·02 (14·97–18·88)	19·47 (17·46–21·31)	14·90 (12·94–16·63)
	UK		17·80 (17·71–17·89)	13·43 (12·25–14·50)	13·98 (13·90–14·05)	10·69 (9·78–11·49)	19·88 (19·79–19·97)	15·01 (13·68–16·19)	17·10 (17·02–17·18)	13·08 (11·97–14·10)	20·88 (20·73–21·04)	15·77 (14·37–17·07)	18·27 (18·13–18·40)	14·00 (12·83–15·05)
		England	17·94 (17·87–18·00)	13·51 (12·31–14·61)	14·11 (14·05–14·17)	10·78 (9·86–11·60)	20·03 (19·97–20·10)	15·09 (13·74–16·30)	17·24 (17·18–17·30)	13·17 (12·07–14·18)	21·05 (20·94–21·16)	15·87 (14·44–17·17)	18·44 (18·34–18·54)	14·11 (12·91–15·18)
		Northern Ireland	17·39 (16·77–18·11)	13·25 (12·04–14·43)	13·52 (12·95–14·16)	10·41 (9·47–11·31)	19·69 (19·11–20·35)	15·00 (13·66–16·35)	16·70 (16·11–17·30)	12·86 (11·72–13·93)	20·59 (19·45–21·77)	15·71 (14·13–17·25)	17·75 (16·64–18·93)	13·68 (12·32–15·06)
		Scotland	16·75 (16·13–17·38)	12·77 (11·63–13·89)	12·99 (12·46–13·53)	10·02 (9·10–10·89)	18·69 (18·06–19·34)	14·30 (13·06–15·50)	16·00 (15·44–16·53)	12·38 (11·31–13·40)	19·71 (18·66–20·75)	15·02 (13·60–16·58)	17·16 (16·23–18·09)	13·24 (11·94–14·55)
		Wales	17·67 (17·07–18·28)	13·40 (12·18–14·59)	13·68 (13·14–14·18)	10·51 (9·55–11·40)	19·67 (19·19–20·26)	15·00 (13·70–16·27)	16·92 (16·34–17·45)	13·03 (11·95–14·11)	20·43 (19·42–21·41)	15·58 (14·14–17·10)	17·70 (16·71–18·66)	13·65 (12·32–14·97)
Southern Latin America			17·74 (17·50–17·99)	13·79 (12·63–14·82)	14·03 (13·79–14·27)	10·84 (9·94–11·67)	19·34 (19·11–19·60)	15·03 (13·83–16·15)	15·30 (15·05–15·55)	11·83 (10·86–12·74)	20·24 (19·54–20·92)	15·72 (14·32–16·97)	16·14 (15·49–16·77)	12·50 (11·38–13·58)
	Argentina		17·76 (17·46–18·08)	13·83 (12·70–14·89)	13·90 (13·61–14·19)	10·75 (9·84–11·59)	19·01 (18·70–19·33)	14·82 (13·65–15·91)	14·81 (14·50–15·12)	11·47 (10·52–12·36)	19·73 (19·11–20·39)	15·37 (14·05–16·56)	15·55 (14·94–16·19)	12·06 (11·04–13·05)
	Chile		17·69 (17·18–18·26)	13·64 (12·46–14·77)	14·65 (14·15–15·19)	11·25 (10·19–12·25)	20·40 (19·90–20·91)	15·72 (14·40–16·95)	17·08 (16·61–17·62)	13·14 (11·99–14·23)	21·63 (19·39–23·86)	16·71 (14·58–18·88)	17·91 (15·85–20·06)	13·84 (11·93–15·86)
	Uruguay		17·70 (17·44–17·95)	13·78 (12·64–14·77)	13·69 (13·46–13·92)	10·65 (9·75–11·44)	19·50 (19·24–19·74)	15·17 (13·95–16·22)	14·76 (14·54–15·01)	11·46 (10·49–12·30)	20·44 (19·85–21·02)	15·87 (14·53–17·10)	15·54 (15·03–16·08)	12·09 (11·04–13·07)
**Central Europe, eastern Europe, and central Asia**			**15·81 (15·50–16·12)**	**11·59 (10·43–12·70)**	**12·43 (12·17–12·66)**	**9·08 (8·14–9·92)**	**16·22 (15·89–16·52)**	**11·99 (10·77–13·05)**	**12·41 (12·12–12·70)**	**9·12 (8·21–9·96)**	**17·80 (16·51–18·92)**	**13·16 (11·60–14·68)**	**14·07 (13·21–14·96)**	**10·33 (9·14–11·60)**
	Eastern Europe		15·85 (15·36–16·35)	11·64 (10·50–12·82)	12·20 (11·76–12·66)	8·92 (8·01–9·79)	15·81 (15·30–16·30)	11·74 (10·53–12·79)	11·57 (11·10–12·05)	8·53 (7·66–9·39)	17·37 (15·46–19·28)	12·90 (10·99–14·77)	13·26 (11·85–14·92)	9·79 (8·39–11·34)
		Belarus	16·52 (16·15–16·86)	12·23 (11·04–13·34)	12·94 (12·58–13·28)	9·56 (8·60–10·48)	16·27 (15·89–16·65)	12·10 (10·89–13·16)	11·53 (11·20–11·88)	8·54 (7·67–9·35)	18·15 (16·70–19·71)	13·45 (11·83–15·17)	13·25 (12·01–14·60)	9·79 (8·48–11·19)
		Estonia	16·11 (15·79–16·44)	11·79 (10·58–12·88)	12·21 (11·91–12·52)	8·91 (7·98–9·77)	18·25 (17·93–18·60)	13·55 (12·24–14·75)	13·28 (12·96–13·57)	9·78 (8·80–10·71)	20·14 (19·43–21·38)	14·90 (13·28–16·52)	15·65 (14·85–16·60)	11·50 (10·22–12·74)
		Latvia	15·93 (15·55–16·30)	11·58 (10·34–12·70)	12·23 (11·83–12·64)	8·87 (7·93–9·75)	16·87 (16·49–17·26)	12·49 (11·28–13·60)	12·38 (12·03–12·74)	9·09 (8·16–9·96)	19·04 (18·02–20·10)	14·02 (12·56–15·56)	14·24 (13·32–15·35)	10·44 (9·21–11·68)
		Lithuania	17·10 (16·81–17·39)	12·49 (11·23–13·64)	13·47 (13·18–13·78)	9·82 (8·81–10·74)	17·97 (17·68–18·24)	13·14 (11·83–14·34)	13·26 (12·99–13·56)	9·69 (8·69–10·62)	19·63 (19·02–20·27)	14·42 (12·96–15·79)	14·70 (14·11–15·31)	10·68 (9·43–11·81)
		Moldova	14·43 (13·82–15·08)	10·67 (9·61–11·75)	12·09 (11·51–12·71)	8·94 (7·98–9·83)	14·59 (13·99–15·27)	11·00 (9·86–12·05)	11·89 (11·28–12·52)	8·95 (8·01–9·88)	16·35 (15·32–17·49)	12·29 (10·95–13·68)	13·35 (12·40–14·45)	10·01 (8·85–11·26)
		Russia	15·82 (15·10–16·62)	11·60 (10·43–12·83)	12·01 (11·35–12·78)	8·74 (7·77–9·73)	15·80 (15·04–16·52)	11·70 (10·45–12·88)	11·46 (10·77–12·20)	8·41 (7·47–9·38)	17·27 (14·52–20·14)	12·84 (10·56–15·28)	13·18 (11·27–15·67)	9·72 (7·95–11·73)
		Ukraine	15·79 (15·35–16·24)	11·65 (10·50–12·72)	12·41 (12·02–12·80)	9·15 (8·19–10·04)	15·60 (15·19–16·00)	11·65 (10·49–12·71)	11·64 (11·27–12·04)	8·66 (7·81–9·49)	17·23 (15·13–19·48)	12·93 (11·04–15·00)	13·26 (11·61–15·48)	9·92 (8·44–11·63)
	Central Europe		15·72 (15·58–15·85)	11·42 (10·24–12·46)	12·64 (12·52–12·77)	9·16 (8·18–10·03)	17·49 (17·36–17·64)	12·79 (11·50–13·94)	14·03 (13·90–14·16)	10·19 (9·12–11·12)	18·92 (18·59–19·25)	13·84 (12·43–15·11)	15·38 (15·08–15·69)	11·16 (10·02–12·19)
		Albania	18·27 (18·06–18·51)	13·70 (12·40–14·86)	14·91 (14·71–15·13)	11·14 (10·05–12·10)	17·66 (17·41–17·91)	13·33 (12·10–14·47)	14·96 (14·76–15·17)	11·26 (10·18–12·19)	19·59 (18·61–20·63)	14·70 (13·25–16·22)	16·11 (14·93–17·27)	12·07 (10·68–13·40)
		Bosnia and Herzegovina	15·47 (14·56–16·56)	11·53 (10·22–12·78)	12·83 (12·00–13·72)	9·57 (8·51–10·72)	17·32 (16·38–18·35)	12·84 (11·52–14·23)	14·66 (13·73–15·57)	10·78 (9·57–11·90)	18·56 (17·17–19·95)	13·69 (12·11–15·33)	15·58 (14·44–16·83)	11·39 (10·05–12·76)
		Bulgaria	15·20 (14·94–15·45)	11·24 (10·16–12·23)	12·81 (12·58–13·03)	9·34 (8·39–10·22)	16·22 (15·99–16·46)	12·07 (10·92–13·12)	13·17 (12·93–13·41)	9·71 (8·75–10·59)	17·60 (16·23–18·93)	13·07 (11·52–14·58)	14·28 (13·12–15·58)	10·50 (9·26–11·91)
		Croatia	15·96 (15·35–16·56)	11·68 (10·40–12·87)	12·72 (12·18–13·36)	9·26 (8·25–10·23)	17·50 (16·97–18·04)	12·88 (11·53–14·10)	13·90 (13·41–14·44)	10·18 (9·10–11·21)	18·57 (17·49–19·78)	13·66 (12·08–15·13)	15·15 (14·20–16·27)	11·04 (9·76–12·45)
		Czech Republic	15·33 (15·16–15·49)	10·84 (9·61–11·93)	11·84 (11·68–12·01)	8·44 (7·49–9·26)	18·09 (17·89–18·26)	12·92 (11·51–14·19)	14·69 (14·51–14·89)	10·43 (9·25–11·46)	19·66 (19·21–20·11)	14·10 (12·46–15·53)	16·34 (15·88–16·79)	11·62 (10·26–12·83)
		Hungary	15·43 (15·02–15·87)	10·85 (9·57–12·01)	12·08 (11·72–12·46)	8·58 (7·59–9·51)	17·48 (17·05–17·92)	12·56 (11·22–13·85)	13·54 (13·12–13·98)	9·70 (8·60–10·69)	18·34 (17·45–19·21)	13·25 (11·82–14·70)	14·74 (13·88–15·67)	10·61 (9·36–11·91)
		Macedonia	14·55 (14·03–15·08)	10·86 (9·76–11·88)	12·61 (12·18–13·04)	9·36 (8·42–10·25)	14·93 (14·49–15·43)	11·14 (10·05–12·19)	12·71 (12·32–13·13)	9·44 (8·50–10·33)	16·26 (15·79–16·82)	12·08 (10·86–13·19)	13·67 (13·22–14·15)	10·13 (9·08–11·06)
		Montenegro	18·05 (17·25–18·70)	13·38 (12·05–14·73)	15·39 (14·78–16·01)	11·28 (10·01–12·38)	17·80 (17·45–18·18)	13·23 (11·83–14·45)	14·76 (14·34–15·23)	10·88 (9·78–11·93)	18·50 (17·59–19·69)	13·73 (12·17–15·34)	15·72 (14·87–16·56)	11·56 (10·30–12·82)
		Poland	16·07 (15·76–16·37)	11·70 (10·48–12·78)	12·40 (12·15–12·70)	9·02 (8·08–9·89)	18·64 (18·37–18·94)	13·56 (12·18–14·84)	14·52 (14·22–14·83)	10·51 (9·40–11·53)	20·10 (19·32–20·89)	14·69 (13·06–16·19)	16·03 (15·32–16·80)	11·61 (10·31–12·85)
		Romania	15·37 (15·00–15·76)	11·32 (10·16–12·37)	13·17 (12·78–13·52)	9·57 (8·57–10·52)	16·86 (16·48–17·27)	12·44 (11·23–13·61)	13·89 (13·54–14·25)	10·13 (9·06–11·13)	18·11 (17·30–18·94)	13·40 (12·02–14·73)	14·78 (13·93–15·63)	10·79 (9·53–12·02)
		Serbia	16·12 (15·50–16·90)	11·85 (10·61–13·13)	13·48 (12·93–14·25)	9·88 (8·82–10·96)	15·81 (15·34–16·26)	11·67 (10·49–12·76)	13·42 (13·05–13·85)	9·84 (8·80–10·81)	17·87 (17·46–18·22)	13·12 (11·77–14·30)	14·95 (14·58–15·45)	10·92 (9·77–11·97)
		Slovakia	15·65 (15·33–15·99)	11·39 (10·22–12·44)	12·14 (11·83–12·46)	8·74 (7·76–9·58)	17·30 (16·98–17·65)	12·75 (11·47–13·88)	13·46 (13·14–13·76)	9·76 (8·70–10·71)	18·79 (17·84–19·81)	13·84 (12·34–15·36)	15·07 (14·16–16·02)	10·90 (9·55–12·23)
		Slovenia	16·98 (16·32–17·66)	12·11 (10·74–13·47)	13·24 (12·67–13·90)	9·42 (8·27–10·46)	19·67 (19·06–20·38)	14·01 (12·47–15·47)	15·53 (14·86–16·18)	10·97 (9·67–12·25)	21·28 (20·30–22·41)	15·12 (13·28–16·79)	17·51 (16·48–18·62)	12·44 (10·88–14·05)
	Central Asia		15·86 (15·55–16·14)	11·82 (10·69–12·85)	12·81 (12·53–13·07)	9·64 (8·74–10·45)	15·03 (14·74–15·32)	11·29 (10·24–12·21)	12·04 (11·76–12·30)	9·13 (8·31–9·91)	16·82 (16·31–17·33)	12·58 (11·34–13·68)	13·51 (13·06–14·02)	10·21 (9·17–11·16)
		Armenia	16·36 (15·90–16·84)	12·21 (11·01–13·31)	13·96 (13·48–14·47)	10·43 (9·38–11·39)	16·46 (15·89–16·98)	12·35 (11·12–13·46)	13·65 (13·18–14·16)	10·26 (9·26–11·21)	18·31 (17·42–19·33)	13·70 (12·15–15·07)	14·76 (14·01–15·62)	11·09 (9·93–12·20)
		Azerbaijan	15·87 (15·41–16·47)	11·96 (10·82–13·07)	12·45 (12·04–12·87)	9·48 (8·59–10·34)	14·90 (14·14–15·75)	11·31 (10·09–12·42)	12·22 (11·59–12·93)	9·37 (8·51–10·26)	17·26 (15·65–18·62)	12·98 (11·35–14·41)	13·75 (12·53–15·42)	10·48 (9·13–12·11)
		Georgia	16·28 (15·61–16·95)	12·30 (11·04–13·42)	13·14 (12·46–13·87)	9·94 (8·92–10·88)	17·77 (17·33–18·20)	13·44 (12·20–14·58)	13·26 (12·81–13·78)	10·12 (9·16–11·03)	18·50 (17·13–20·32)	13·91 (12·39–15·46)	13·78 (12·62–15·14)	10·40 (9·08–11·74)
		Kazakhstan	15·94 (15·11–16·76)	11·75 (10·44–12·93)	12·21 (11·48–13·01)	9·09 (8·03–10·08)	14·72 (13·98–15·51)	10·92 (9·69–12·05)	10·91 (10·23–11·64)	8·13 (7·20–9·07)	16·95 (15·49–18·45)	12·54 (11·03–14·06)	13·26 (11·89–14·72)	9·86 (8·52–11·34)
		Kyrgyzstan	15·81 (15·45–16·19)	11·73 (10·58–12·80)	12·46 (12·14–12·82)	9·33 (8·46–10·13)	14·41 (14·08–14·76)	10·85 (9·85–11·76)	11·52 (11·18–11·83)	8·75 (7·93–9·49)	16·64 (15·84–17·41)	12·47 (11·20–13·72)	13·55 (12·87–14·30)	10·27 (9·21–11·28)
		Mongolia	14·23 (13·32–15·22)	10·73 (9·49–11·90)	12·20 (11·48–12·78)	9·26 (8·31–10·19)	13·75 (13·13–14·34)	10·36 (9·33–11·37)	11·11 (10·62–11·75)	8·40 (7·58–9·23)	15·61 (14·55–16·74)	11·69 (10·34–13·01)	12·29 (11·45–13·25)	9·20 (8·10–10·27)
		Tajikistan	15·87 (15·14–16·68)	11·88 (10·66–13·07)	13·64 (12·79–14·49)	10·28 (9·21–11·35)	14·52 (13·56–15·40)	11·01 (9·92–12·18)	13·00 (12·29–13·80)	9·92 (8·89–10·94)	16·92 (15·66–17·89)	12·78 (11·34–14·08)	14·90 (13·58–15·85)	11·34 (10·05–12·50)
		Turkmenistan	15·09 (14·84–15·36)	11·39 (10·31–12·35)	12·42 (12·23–12·61)	9·49 (8·65–10·24)	15·60 (15·30–15·91)	11·86 (10·77–12·87)	12·73 (12·48–12·97)	9·79 (8·93–10·57)	16·95 (16·34–17·52)	12·85 (11·60–14·03)	13·73 (13·31–14·15)	10·54 (9·62–11·42)
		Uzbekistan	15·72 (15·18–16·23)	11·69 (10·51–12·80)	13·19 (12·71–13·72)	9·97 (9·00–10·89)	14·18 (13·65–14·77)	10·68 (9·59–11·72)	11·93 (11·30–12·50)	9·11 (8·24–10·01)	15·76 (14·90–16·65)	11·84 (10·59–13·07)	13·13 (12·43–13·80)	10·02 (8·97–10·98)
**Latin America and Caribbean**			**17·13 (17·03–17·22)**	**12·97 (11·81–13·99)**	**14·69 (14·60–14·78)**	**11·18 (10·21–12·06)**	**18·73 (18·62–18·83)**	**14·17 (12·89–15·31)**	**16·05 (15·95–16·14)**	**12·21 (11·13–13·19)**	**19·49 (19·29–19·68)**	**14·74 (13·39–15·94)**	**16·78 (16·61–16·95)**	**12·78 (11·65–13·77)**
	Central Latin America		17·96 (17·84–18·09)	13·61 (12·38–14·68)	15·76 (15·64–15·89)	11·94 (10·84–12·89)	19·16 (19·01–19·32)	14·60 (13·31–15·75)	16·71 (16·55–16·86)	12·72 (11·58–13·73)	19·75 (19·46–20·07)	15·03 (13·67–16·25)	17·30 (17·00–17·58)	13·17 (12·00–14·22)
		Colombia	17·85 (17·57–18·11)	13·69 (12·44–14·78)	15·41 (15·17–15·66)	11·86 (10·81–12·78)	18·65 (18·41–18·89)	14·40 (13·23–15·45)	16·03 (15·78–16·27)	12·44 (11·41–13·35)	20·24 (19·48–21·04)	15·61 (14·17–16·91)	17·58 (16·85–18·33)	13·60 (12·36–14·77)
		Costa Rica	18·86 (18·53–19·21)	14·42 (13·13–15·58)	16·38 (16·04–16·70)	12·66 (11·59–13·65)	21·26 (20·90–21·58)	16·21 (14·75–17·52)	18·52 (18·19–18·85)	14·24 (13·01–15·37)	22·29 (21·64–22·94)	16·99 (15·40–18·49)	19·50 (18·88–20·18)	15·00 (13·61–16·28)
		El Salvador	17·51 (17·14–17·91)	13·43 (12·21–14·52)	15·26 (14·91–15·61)	11·67 (10·65–12·61)	18·74 (18·39–19·10)	14·36 (13·07–15·50)	17·54 (17·20–17·89)	13·35 (12·13–14·50)	19·56 (18·61–20·48)	14·99 (13·49–16·35)	17·95 (17·06–18·90)	13·68 (12·29–14·98)
		Guatemala	15·45 (14·53–16·45)	11·75 (10·45–13·01)	14·76 (13·91–15·90)	11·23 (10·11–12·51)	18·05 (16·81–19·26)	13·60 (12·08–15·08)	16·33 (15·32–17·47)	12·40 (11·06–13·85)	18·76 (16·58–21·20)	14·24 (12·19–16·30)	16·86 (15·14–18·96)	12·84 (11·08–14·83)
		Honduras	14·00 (13·26–14·84)	10·79 (9·76–11·81)	15·71 (14·10–16·95)	12·11 (10·76–13·48)	15·24 (13·70–17·73)	11·69 (10·10–13·62)	15·29 (13·25–17·63)	11·85 (9·93–13·82)	16·03 (14·34–18·60)	12·29 (10·60–14·36)	15·73 (13·50–17·94)	12·16 (10·19–14·03)
		Mexico	18·45 (18·33–18·59)	13·87 (12·60–15·00)	16·04 (15·91–16·18)	12·00 (10·87–13·00)	19·42 (19·24–19·59)	14·71 (13·38–15·92)	16·91 (16·77–17·05)	12·74 (11·55–13·76)	19·48 (19·23–19·75)	14·72 (13·35–15·93)	17·20 (16·99–17·42)	12·97 (11·74–14·04)
		Nicaragua	20·99 (20·64–21·35)	15·97 (14·58–17·26)	18·21 (17·86–18·57)	13·94 (12·68–15·09)	20·55 (20·18–20·95)	15·73 (14·32–17·00)	18·29 (17·93–18·63)	14·08 (12·86–15·23)	20·98 (19·63–22·42)	16·05 (14·41–17·73)	18·38 (17·12–19·75)	14·16 (12·53–15·71)
		Panama	18·86 (18·27–19·48)	14·45 (13·09–15·71)	16·24 (15·62–16·85)	12·49 (11·36–13·54)	20·69 (20·12–21·25)	15·85 (14·35–17·10)	18·01 (17·40–18·61)	13·82 (12·58–14·95)	21·73 (20·64–22·85)	16·60 (14·95–18·18)	18·76 (17·55–19·96)	14·32 (12·83–15·78)
		Venezuela	17·29 (16·76–17·88)	13·21 (11·94–14·34)	15·08 (14·50–15·59)	11·56 (10·48–12·58)	19·62 (19·01–20·20)	15·00 (13·59–16·26)	16·42 (15·91–16·98)	12·62 (11·48–13·67)	20·19 (18·68–21·62)	15·47 (13·72–17·22)	16·71 (15·27–17·97)	12·85 (11·36–14·26)
	Andean Latin America		17·23 (16·84–17·65)	13·14 (11·88–14·26)	15·71 (15·35–16·06)	11·97 (10·91–12·93)	19·66 (19·22–20·09)	15·03 (13·66–16·22)	17·61 (17·23–18·01)	13·45 (12·21–14·55)	20·12 (19·15–21·07)	15·39 (13·86–16·86)	18·36 (17·53–19·23)	14·04 (12·63–15·41)
		Bolivia	13·86 (12·86–14·74)	10·46 (9·25–11·63)	13·76 (13·09–14·45)	10·31 (9·25–11·31)	16·68 (15·58–17·39)	12·62 (11·19–13·88)	15·79 (15·11–16·68)	11·92 (10·69–13·14)	16·93 (14·96–18·57)	12·88 (10·99–14·55)	16·60 (15·49–18·03)	12·55 (11·04–14·20)
		Ecuador	18·27 (17·92–18·63)	13·99 (12·74–15·13)	16·17 (15·84–16·50)	12·33 (11·20–13·34)	20·21 (19·87–20·58)	15·46 (14·06–16·68)	17·90 (17·50–18·28)	13·57 (12·33–14·73)	20·84 (20·21–21·44)	15·93 (14·44–17·28)	18·67 (18·02–19·29)	14·19 (12·88–15·46)
		Peru	18·20 (17·64–18·79)	13·91 (12·58–15·12)	16·24 (15·61–16·83)	12·45 (11·36–13·53)	20·54 (19·91–21·30)	15·75 (14·26–17·02)	18·13 (17·46–18·81)	13·96 (12·60–15·15)	21·06 (19·44–22·77)	16·17 (14·35–18·06)	18·88 (17·36–20·52)	14·55 (12·85–16·20)
	Caribbean		16·54 (16·30–16·77)	12·69 (11·53–13·70)	15·12 (14·93–15·31)	11·66 (10·65–12·59)	17·99 (17·59–18·41)	13·73 (12·50–14·87)	16·16 (15·91–16·45)	12·40 (11·31–13·40)	18·59 (18·05–19·17)	14·19 (12·92–15·40)	16·50 (16·08–16·90)	12·66 (11·51–13·71)
		Antigua and Barbuda	17·09 (16·33–17·88)	13·17 (11·91–14·43)	14·14 (13·51–14·78)	10·84 (9·86–11·83)	19·02 (18·29–19·74)	14·52 (13·08–15·89)	16·29 (15·63–17·05)	12·44 (11·23–13·63)	19·74 (18·72–20·85)	15·10 (13·53–16·61)	16·79 (15·84–17·78)	12·81 (11·51–14·18)
		The Bahamas	16·94 (16·60–17·27)	13·01 (11·83–14·05)	13·96 (13·64–14·31)	10·79 (9·87–11·67)	17·81 (17·35–18·34)	13·66 (12·44–14·78)	15·53 (15·21–15·85)	11·99 (10·96–12·92)	17·58 (16·57–18·78)	13·50 (12·09–14·92)	15·74 (15·06–16·52)	12·16 (11·02–13·37)
		Barbados	17·20 (16·69–17·72)	13·19 (11·94–14·35)	14·19 (13·68–14·60)	10·95 (9·96–11·88)	19·40 (18·87–19·90)	14·74 (13·35–15·99)	16·36 (15·86–16·88)	12·53 (11·41–13·56)	19·37 (18·61–20·22)	14·70 (13·25–16·03)	16·74 (15·99–17·50)	12·85 (11·63–14·06)
		Belize	17·21 (16·26–18·14)	13·19 (11·80–14·46)	15·55 (14·28–16·17)	12·02 (10·79–13·10)	16·04 (15·65–16·40)	12·21 (11·10–13·28)	13·46 (12·58–14·25)	10·35 (9·26–11·43)	16·61 (15·62–17·69)	12·67 (11·31–14·03)	14·05 (13·07–15·19)	10·82 (9·66–11·98)
		Bermuda	13·93 (13·33–14·58)	10·77 (9·76–11·75)	12·47 (11·92–13·01)	9·61 (8·67–10·44)	16·90 (16·17–17·60)	13·00 (11·83–14·16)	14·73 (14·17–15·26)	11·35 (10·36–12·31)	20·42 (19·08–22·13)	15·70 (14·10–17·46)	16·31 (15·32–17·25)	12·57 (11·28–13·80)
		Cuba	18·18 (17·83–18·54)	13·94 (12·70–15·07)	15·72 (15·35–16·04)	12·19 (11·13–13·19)	19·45 (19·10–19·79)	14·87 (13·50–16·07)	16·88 (16·52–17·24)	13·07 (11·93–14·09)	20·23 (19·53–21·00)	15·44 (13·98–16·78)	17·31 (16·65–18·00)	13·37 (12·10–14·57)
		Dominica	14·93 (14·41–15·55)	11·56 (10·49–12·53)	13·63 (13·14–14·30)	10·54 (9·61–11·48)	16·65 (15·84–17·31)	12·83 (11·63–13·89)	14·38 (13·82–15·01)	11·06 (10·03–12·02)	17·26 (16·02–18·20)	13·30 (11·90–14·66)	14·63 (13·65–15·80)	11·25 (9·98–12·51)
		Dominican Republic	17·36 (16·88–18·08)	13·47 (12·25–14·65)	16·05 (15·48–16·60)	12·50 (11·38–13·60)	19·45 (18·41–20·68)	15·02 (13·38–16·68)	16·81 (16·34–17·89)	12·98 (11·82–14·26)	19·65 (18·48–21·24)	15·19 (13·66–17·00)	17·21 (16·33–18·48)	13·33 (12·00–14·63)
		Grenada	15·64 (14·97–16·19)	11·90 (10·74–12·91)	13·71 (12·95–14·40)	10·50 (9·44–11·58)	16·05 (15·32–16·71)	12·17 (10·94–13·21)	13·79 (13·16–14·36)	10·54 (9·50–11·48)	16·29 (15·19–17·62)	12·37 (11·08–13·68)	13·96 (13·11–14·93)	10·67 (9·53–11·79)
		Guyana	14·71 (14·21–15·34)	11·22 (10·18–12·19)	11·79 (11·24–12·38)	9·03 (8·16–9·91)	14·36 (13·76–14·92)	10·87 (9·76–11·87)	12·34 (11·84–12·84)	9·35 (8·43–10·20)	15·17 (14·32–16·19)	11·49 (10·28–12·70)	12·98 (12·28–13·74)	9·88 (8·86–10·85)
		Haiti	10·84 (10·21–11·40)	8·24 (7·41–9·09)	12·30 (11·78–12·82)	9·34 (8·43–10·24)	12·27 (11·18–13·67)	9·35 (8·20–10·54)	13·25 (12·48–14·47)	10·10 (8·98–11·30)	13·04 (11·71–14·55)	9·95 (8·74–11·38)	13·75 (12·36–15·23)	10·52 (9·14–11·91)
		Jamaica	17·83 (17·01–18·62)	13·63 (12·25–14·93)	16·83 (16·27–17·47)	12·99 (11·83–14·11)	18·14 (17·52–18·87)	13·71 (12·37–15·01)	16·79 (16·09–17·44)	12·85 (11·59–13·95)	17·93 (16·46–19·36)	13·55 (11·91–15·24)	16·28 (14·77–17·58)	12·45 (10·95–13·86)
		Puerto Rico	18·89 (18·50–19·30)	14·53 (13·27–15·68)	15·88 (15·48–16·28)	12·17 (11·08–13·17)	20·52 (20·09–20·90)	15·59 (14·12–16·90)	17·38 (16·94–17·76)	13·14 (11·88–14·26)	21·13 (20·34–21·98)	16·10 (14·54–17·53)	17·57 (16·86–18·31)	13·29 (12·00–14·48)
		Saint Lucia	17·07 (16·72–17·43)	12·89 (11·64–14·01)	14·99 (14·66–15·35)	11·40 (10·43–12·39)	18·95 (18·51–19·41)	14·43 (13·08–15·70)	16·32 (15·93–16·73)	12·49 (11·38–13·53)	20·02 (19·49–20·59)	15·26 (13·84–16·59)	17·16 (16·60–17·66)	13·15 (11·94–14·26)
		Saint Vincent and the Grenadines	15·68 (15·08–16·14)	11·96 (10·81–12·98)	13·84 (13·14–15·13)	10·63 (9·53–11·84)	16·03 (15·63–16·49)	12·14 (11·00–13·20)	14·02 (13·42–14·51)	10·66 (9·60–11·67)	16·39 (15·68–17·33)	12·45 (11·18–13·74)	14·02 (13·13–14·60)	10·64 (9·49–11·70)
		Suriname	16·97 (16·61–17·36)	12·97 (11·75–14·08)	15·17 (14·98–15·38)	11·64 (10·64–12·56)	16·22 (15·66–16·64)	12·32 (11·14–13·38)	14·05 (13·80–14·37)	10·74 (9·75–11·63)	17·14 (16·40–17·75)	12·99 (11·71–14·22)	14·49 (13·84–15·34)	11·05 (9·99–12·13)
		Trinidad and Tobago	15·44 (15·10–15·81)	11·69 (10·61–12·67)	12·43 (12·14–12·73)	9·44 (8·56–10·27)	17·67 (17·25–18·11)	13·29 (12·00–14·44)	13·95 (13·63–14·31)	10·40 (9·36–11·37)	18·72 (18·05–19·41)	14·08 (12·68–15·41)	14·40 (13·83–15·02)	10·71 (9·59–11·83)
		Virgin Islands	17·05 (16·58–18·07)	13·21 (12·03–14·43)	13·96 (13·56–14·49)	10·80 (9·85–11·68)	17·93 (17·52–18·31)	13·79 (12·54–14·86)	14·50 (13·96–14·99)	11·12 (10·09–12·08)	18·47 (17·86–19·07)	14·17 (12·89–15·45)	14·61 (13·76–15·71)	11·18 (9·97–12·37)
	Tropical Latin America		16·41 (16·23–16·57)	12·34 (11·22–13·34)	13·16 (13·01–13·32)	10·04 (9·15–10·87)	18·28 (18·11–18·43)	13·67 (12·37–14·83)	15·00 (14·84–15·14)	11·35 (10·35–12·29)	19·31 (19·02–19·58)	14·45 (13·09–15·67)	15·99 (15·74–16·23)	12·14 (11·05–13·14)
		Brazil	16·36 (16·18–16·52)	12·30 (11·18–13·29)	13·08 (12·93–13·24)	9·97 (9·09–10·80)	18·29 (18·12–18·45)	13·68 (12·37–14·84)	14·96 (14·81–15·11)	11·32 (10·32–12·26)	19·34 (19·05–19·61)	14·48 (13·11–15·69)	16·00 (15·75–16·24)	12·14 (11·06–13·15)
		Paraguay	18·47 (17·94–18·88)	13·98 (12·70–15·17)	16·60 (16·23–17·00)	12·70 (11·60–13·80)	17·79 (17·10–18·51)	13·43 (12·05–14·74)	16·26 (16·01–16·45)	12·38 (11·28–13·37)	17·90 (17·02–18·91)	13·45 (11·97–14·85)	15·75 (14·68–16·54)	12·03 (10·72–13·30)
**Southeast Asia, east Asia, and Oceania**			**14·53 (14·35–14·71)**	**11·16 (10·28–12·02)**	**12·44 (12·30–12·58)**	**9·76 (9·02–10·43)**	**16·47 (16·33–16·61)**	**12·53 (11·46–13·49)**	**14·06 (13·93–14·19)**	**10·95 (10·09–11·75)**	**18·48 (18·30–18·65)**	**13·99 (12·81–15·07)**	**15·09 (14·93–15·25)**	**11·69 (10·79–12·52)**
	East Asia		14·51 (14·29–14·73)	11·23 (10·37–12·07)	12·20 (12·03–12·37)	9·68 (8·98–10·32)	16·76 (16·60–16·93)	12·83 (11·76–13·80)	14·06 (13·92–14·20)	11·05 (10·23–11·84)	18·97 (18·76–19·18)	14·43 (13·22–15·51)	15·17 (15·00–15·35)	11·85 (10·95–12·67)
		China	14·45 (14·22–14·68)	11·19 (10·34–12·02)	12·14 (11·97–12·31)	9·64 (8·94–10·28)	16·73 (16·57–16·90)	12·81 (11·75–13·78)	14·01 (13·86–14·15)	11·02 (10·20–11·81)	19·01 (18·79–19·22)	14·45 (13·24–15·53)	15·14 (14·97–15·32)	11·83 (10·93–12·65)
		North Korea	16·18 (15·40–17·33)	12·48 (11·32–13·76)	14·00 (13·20–15·35)	11·05 (9·93–12·35)	15·59 (14·98–16·45)	11·94 (10·80–13·08)	13·41 (13·04–13·90)	10·56 (9·72–11·32)	15·93 (15·36–16·53)	12·26 (11·20–13·38)	13·49 (13·10–13·97)	10·60 (9·77–11·46)
		Taiwan (Province of China)	16·63 (16·27–16·99)	12·55 (11·40–13·63)	14·61 (14·28–14·95)	11·31 (10·35–12·19)	19·85 (19·50–20·20)	14·95 (13·63–16·14)	17·05 (16·70–17·43)	13·14 (12·09–14·12)	20·99 (19·97–22·16)	15·88 (14·32–17·50)	17·87 (16·87–19·00)	13·79 (12·50–15·10)
	Southeast Asia		14·63 (14·34–14·93)	10·95 (9·93–11·91)	13·39 (13·16–13·58)	10·08 (9·14–10·88)	15·69 (15·50–16·02)	11·73 (10·66–12·70)	14·13 (13·90–14·38)	10·62 (9·71–11·47)	17·13 (16·85–17·41)	12·79 (11·63–13·88)	14·84 (14·52–15·17)	11·12 (10·12–12·02)
		Cambodia	11·93 (11·51–12·45)	8·69 (7·75–9·61)	11·75 (11·15–12·30)	8·65 (7·71–9·57)	13·52 (13·24–13·86)	9·80 (8·78–10·72)	12·67 (12·01–13·24)	9·25 (8·30–10·18)	15·07 (14·62–15·57)	11·06 (9·97–12·11)	13·07 (12·57–13·72)	9·67 (8·71–10·55)
		Indonesia	13·72 (13·37–14·03)	10·32 (9·32–11·21)	13·37 (13·21–13·56)	10·08 (9·19–10·90)	14·55 (14·29–14·79)	10·90 (9·87–11·81)	14·29 (13·98–14·65)	10·73 (9·75–11·62)	15·81 (15·48–16·15)	11·76 (10·69–12·76)	14·60 (14·01–15·27)	10·89 (9·83–11·92)
		Laos	11·73 (11·39–12·17)	8·67 (7·78–9·51)	11·77 (11·57–11·95)	8·73 (7·87–9·50)	13·68 (13·19–14·14)	10·13 (9·09–11·06)	12·67 (12·50–12·86)	9·38 (8·48–10·23)	15·17 (14·61–15·85)	11·23 (10·07–12·33)	13·73 (13·34–14·22)	10·19 (9·23–11·12)
		Malaysia	14·92 (14·72–15·13)	11·19 (10·19–12·12)	13·52 (13·33–13·71)	10·25 (9·33–11·06)	15·32 (15·15–15·48)	11·54 (10·47–12·46)	14·82 (14·64–15·01)	11·18 (10·22–12·06)	16·88 (16·51–17·23)	12·69 (11·48–13·75)	15·29 (14·84–15·80)	11·52 (10·48–12·49)
		Maldives	13·01 (12·41–13·75)	9·59 (8·57–10·60)	13·63 (13·03–14·33)	10·18 (9·17–11·15)	16·96 (16·25–17·74)	12·59 (11·28–13·86)	16·18 (15·50–16·86)	12·14 (10·94–13·28)	19·43 (17·36–21·50)	14·45 (12·44–16·33)	17·22 (15·48–19·16)	12·97 (11·30–14·71)
		Mauritius	15·09 (14·74–15·43)	11·24 (10·20–12·24)	11·01 (10·75–11·27)	8·24 (7·50–8·94)	16·74 (16·36–17·09)	12·41 (11·14–13·51)	13·47 (13·17–13·81)	9·99 (9·08–10·83)	18·00 (16·79–19·13)	13·27 (11·79–14·82)	14·82 (13·88–15·88)	10·92 (9·63–12·24)
		Myanmar	12·18 (11·76–12·67)	9·03 (8·11–9·93)	11·62 (10·81–12·30)	8·65 (7·71–9·53)	13·84 (13·40–14·39)	10·27 (9·21–11·25)	12·70 (11·00–13·42)	9·44 (8·16–10·46)	15·81 (15·37–16·33)	11·77 (10·65–12·85)	13·35 (12·79–13·87)	9·93 (8·97–10·86)
		Philippines	16·15 (15·77–16·56)	12·05 (10·91–13·14)	13·10 (12·72–13·52)	9·72 (8·81–10·60)	15·31 (14·86–15·73)	11·38 (10·31–12·43)	11·96 (11·59–12·37)	8·84 (8·01–9·64)	15·97 (14·91–17·14)	11·92 (10·64–13·33)	12·57 (11·61–13·58)	9·33 (8·33–10·43)
		Sri Lanka	17·52 (17·00–18·08)	13·19 (11·99–14·37)	14·96 (14·42–15·50)	11·28 (10·21–12·32)	18·32 (17·49–19·02)	13·74 (12·46–14·96)	14·95 (14·25–15·60)	11·21 (10·13–12·29)	19·70 (17·93–21·75)	14·78 (12·86–16·74)	16·19 (14·38–18·07)	12·16 (10·51–13·86)
		Seychelles	16·08 (15·70–16·49)	12·20 (11·15–13·23)	12·46 (12·07–12·83)	9·44 (8·59–10·20)	17·04 (16·59–17·71)	12·84 (11·62–14·03)	13·77 (13·52–14·07)	10·34 (9·39–11·21)	17·73 (17·02–18·45)	13·37 (12·11–14·63)	14·42 (13·57–15·40)	10·83 (9·78–11·97)
		Thailand	17·07 (16·70–17·44)	12·73 (11·52–13·82)	14·71 (14·35–15·08)	11·06 (10·03–11·97)	18·61 (18·26–18·95)	13·94 (12·66–15·12)	16·71 (16·32–17·12)	12·60 (11·48–13·67)	20·33 (19·48–21·10)	15·20 (13·72–16·69)	18·29 (17·42–19·15)	13·72 (12·39–15·05)
		Timor-Leste	12·77 (11·35–14·28)	9·51 (8·13–10·90)	14·36 (13·39–15·51)	10·59 (9·34–11·75)	15·27 (13·91–16·46)	11·29 (9·92–12·61)	15·14 (14·16–16·34)	11·09 (9·79–12·44)	16·51 (14·75–18·09)	12·30 (10·69–14·01)	16·13 (14·64–17·71)	11·85 (10·29–13·35)
		Vietnam	15·25 (14·17–16·51)	11·45 (10·09–12·89)	13·48 (12·46–14·35)	10·26 (9·20–11·37)	16·67 (15·90–18·27)	12·50 (11·23–14·01)	13·60 (12·77–14·67)	10·35 (9·39–11·47)	18·08 (17·34–18·54)	13·64 (12·34–14·82)	14·45 (13·89–15·11)	11·02 (10·01–11·98)
	Oceania		11·30 (10·70–11·96)	8·31 (7·36–9·20)	10·56 (10·16–11·10)	7·90 (7·10–8·67)	11·38 (10·70–12·09)	8·32 (7·36–9·27)	10·91 (10·42–11·50)	8·13 (7·29–8·97)	12·01 (11·22–12·77)	8·76 (7·75–9·74)	11·45 (10·87–12·11)	8·51 (7·63–9·39)
		American Samoa	16·43 (15·88–17·15)	11·91 (10·65–13·22)	12·91 (12·44–13·49)	9·45 (8·46–10·44)	16·12 (15·15–16·94)	11·64 (10·29–13·04)	14·02 (13·37–14·63)	10·21 (9·09–11·29)	15·98 (14·58–17·47)	11·53 (9·99–12·99)	14·37 (13·33–15·54)	10·46 (9·16–11·80)
		Federated States of Micronesia	12·64 (11·69–13·56)	9·39 (8·27–10·49)	11·23 (10·67–12·11)	8·45 (7·58–9·41)	12·76 (11·43–14·06)	9·42 (8·17–10·72)	12·21 (11·45–13·00)	9·14 (8·10–10·13)	12·75 (11·38–14·23)	9·42 (8·06–10·85)	12·04 (10·95–13·00)	9·00 (7·87–10·11)
		Fiji	13·45 (11·49–15·47)	9·85 (8·21–11·54)	11·66 (10·65–12·89)	8·62 (7·53–9·83)	13·13 (12·17–14·29)	9·52 (8·37–10·74)	11·20 (10·47–12·23)	8·24 (7·26–9·23)	13·47 (11·50–15·69)	9·82 (8·23–11·61)	11·68 (10·19–13·64)	8·66 (7·25–10·35)
		Guam	17·14 (16·61–17·86)	12·76 (11·46–13·97)	14·25 (13·65–14·71)	10·87 (9·84–11·79)	17·31 (16·78–17·86)	12·80 (11·54–14·04)	14·27 (13·59–14·70)	10·78 (9·75–11·72)	17·05 (16·15–18·10)	12·54 (11·21–13·92)	13·84 (12·96–14·80)	10·41 (9·31–11·48)
		Kiribati	11·64 (11·08–12·19)	8·48 (7·53–9·40)	9·98 (9·56–10·37)	7·43 (6·67–8·17)	12·46 (11·70–13·23)	9·03 (7·98–10·11)	10·19 (9·74–10·71)	7·48 (6·64–8·27)	12·82 (11·82–13·94)	9·30 (8·09–10·54)	10·57 (9·87–11·34)	7·77 (6·83–8·72)
		Marshall Islands	13·63 (13·11–14·14)	9·82 (8·76–10·81)	11·30 (10·95–11·79)	8·31 (7·48–9·12)	12·03 (11·25–12·89)	8·65 (7·56–9·71)	11·28 (10·73–11·95)	8·21 (7·31–9·09)	12·74 (11·41–14·15)	9·15 (7·80–10·49)	11·80 (10·93–12·79)	8·57 (7·50–9·72)
		Northern Mariana Islands	16·02 (14·53–17·64)	11·88 (10·32–13·46)	15·25 (14·18–16·44)	11·44 (10·16–12·73)	17·57 (16·22–18·95)	12·96 (11·34–14·42)	16·23 (15·29–17·11)	12·10 (10·78–13·31)	17·39 (15·85–19·01)	12·82 (11·17–14·47)	15·83 (14·79–16·83)	11·78 (10·52–13·01)
		Papua New Guinea	10·42 (9·66–11·25)	7·67 (6·73–8·53)	9·85 (9·29–10·61)	7·38 (6·59–8·20)	10·53 (9·67–11·44)	7·71 (6·72–8·70)	10·39 (9·69–11·28)	7·75 (6·86–8·70)	11·28 (10·33–12·24)	8·23 (7·15–9·27)	11·06 (10·33–12·01)	8·23 (7·31–9·19)
		Samoa	15·24 (14·27–16·22)	11·28 (10·09–12·57)	12·69 (11·89–13·41)	9·52 (8·44–10·48)	15·81 (14·88–16·91)	11·63 (10·30–13·03)	13·77 (12·99–14·79)	10·30 (9·14–11·42)	15·90 (14·75–17·11)	11·69 (10·38–13·14)	14·12 (13·40–15·41)	10·56 (9·48–11·80)
		Solomon Islands	10·92 (10·08–11·94)	8·07 (7·13–9·17)	10·58 (10·00–11·27)	7·98 (7·24–8·80)	10·86 (9·92–11·88)	7·96 (6·91–9·01)	11·01 (10·37–11·63)	8·25 (7·38–9·11)	11·46 (10·32–12·63)	8·37 (7·28–9·47)	11·54 (10·65–12·35)	8·62 (7·64–9·58)
		Tonga	14·22 (13·03–15·53)	10·35 (9·05–11·70)	12·64 (11·67–13·59)	9·53 (8·42–10·67)	15·32 (14·40–16·39)	11·13 (9·84–12·46)	13·09 (12·29–13·88)	9·79 (8·74–10·85)	15·71 (14·39–16·99)	11·41 (9·94–12·84)	13·34 (12·52–14·34)	9·94 (8·88–11·04)
		Vanuatu	11·74 (10·88–12·67)	8·66 (7·59–9·81)	10·89 (10·39–11·41)	8·21 (7·39–9·03)	11·80 (10·72–12·79)	8·69 (7·56–9·81)	11·19 (10·57–11·88)	8·45 (7·62–9·32)	12·30 (11·03–13·39)	9·02 (7·80–10·23)	11·65 (10·89–12·46)	8·76 (7·81–9·74)
**North Africa and Middle East**			**16·10 (15·87–16·32)**	**11·38 (10·13–12·52)**	**14·38 (14·15–14·64)**	**10·49 (9·38–11·50)**	**16·75 (16·42–17·03)**	**11·92 (10·59–13·15)**	**15·12 (14·79–15·41)**	**11·13 (10·01–12·13)**	**17·73 (17·30–18·17)**	**12·64 (11·24–13·95)**	**15·68 (15·25–16·08)**	**11·55 (10·38–12·65)**
	Afghanistan		10·00 (9·46–10·63)	6·86 (5·96–7·76)	10·76 (10·11–11·63)	7·65 (6·62–8·68)	9·82 (9·30–10·39)	6·79 (5·91–7·66)	10·29 (9·73–11·04)	7·38 (6·51–8·28)	10·74 (10·16–11·32)	7·43 (6·48–8·34)	10·88 (10·37–11·49)	7·79 (6·90–8·67)
	Algeria		16·89 (16·13–17·53)	11·95 (10·57–13·27)	15·33 (14·80–15·99)	11·17 (10·00–12·32)	18·22 (17·40–18·59)	12·98 (11·48–14·36)	17·13 (16·56–17·81)	12·58 (11·24–13·85)	18·60 (18·10–18·98)	13·26 (11·78–14·59)	17·83 (17·03–18·63)	13·10 (11·67–14·42)
	Bahrain		13·09 (12·02–14·15)	9·13 (7·90–10·42)	11·68 (10·77–12·67)	8·43 (7·39–9·58)	14·63 (13·63–15·74)	10·10 (8·72–11·43)	13·74 (12·77–14·84)	9·84 (8·58–11·14)	16·61 (15·16–18·32)	11·50 (9·81–13·22)	15·48 (13·90–17·23)	11·17 (9·46–12·88)
	Egypt		15·50 (15·07–15·93)	11·06 (9·86–12·20)	12·95 (12·55–13·39)	9·54 (8·51–10·44)	16·19 (15·69–16·69)	11·47 (10·14–12·78)	13·56 (13·10–14·01)	9·97 (8·91–10·95)	16·72 (15·54–18·03)	11·92 (10·41–13·43)	13·96 (12·98–15·12)	10·31 (9·14–11·49)
	Iran		17·07 (16·17–18·28)	12·17 (10·65–13·67)	14·78 (13·74–16·12)	10·85 (9·56–12·25)	16·26 (14·70–17·72)	11·68 (10·13–13·19)	15·30 (13·83–16·63)	11·37 (9·85–12·86)	18·06 (16·88–19·65)	12·95 (11·35–14·60)	16·33 (14·88–17·75)	12·06 (10·57–13·45)
	Iraq		13·72 (12·79–14·82)	9·56 (8·35–10·78)	13·49 (12·09–14·54)	9·65 (8·27–10·90)	13·76 (12·77–14·59)	9·65 (8·39–10·83)	12·55 (11·48–13·61)	9·06 (7·88–10·27)	14·88 (13·44–16·39)	10·43 (8·94–11·94)	13·18 (12·10–15·66)	9·47 (8·25–11·13)
	Jordan		15·78 (14·71–17·27)	11·01 (9·60–12·52)	15·55 (14·57–16·18)	11·19 (9·90–12·38)	15·72 (14·19–17·55)	11·12 (9·45–12·80)	15·81 (14·23–17·25)	11·54 (9·99–13·12)	18·37 (16·45–20·42)	13·04 (11·13–14·79)	16·92 (14·71–19·20)	12·39 (10·66–14·42)
	Kuwait		16·73 (15·39–18·20)	12·00 (10·35–13·56)	16·73 (15·43–18·06)	12·32 (10·88–13·78)	15·91 (14·87–16·92)	11·36 (9·91–12·70)	18·03 (16·86–19·13)	13·21 (11·72–14·69)	17·30 (14·97–19·97)	12·47 (10·50–14·68)	19·83 (17·44–22·40)	14·59 (12·52–16·91)
	Lebanon		15·94 (14·78–17·39)	11·49 (9·98–12·96)	13·80 (12·96–15·50)	10·21 (8·93–11·92)	18·57 (18·21–19·43)	13·42 (11·99–14·84)	18·19 (17·02–19·31)	13·50 (11·95–14·99)	20·08 (18·94–21·24)	14·52 (12·82–16·24)	18·59 (17·30–19·72)	13·74 (12·16–15·26)
	Libya		17·46 (16·60–17·98)	12·39 (10·97–13·74)	15·95 (15·37–16·74)	11·67 (10·46–12·91)	17·84 (16·53–18·44)	12·67 (11·04–14·13)	16·49 (15·92–17·19)	12·07 (10·79–13·28)	18·09 (17·32–18·66)	12·86 (11·32–14·24)	15·41 (14·67–16·77)	11·32 (9·98–12·71)
	Morocco		15·46 (14·74–16·09)	11·00 (9·75–12·24)	14·26 (13·88–14·63)	10·45 (9·37–11·45)	16·61 (15·64–17·43)	11·88 (10·50–13·23)	15·77 (15·40–16·10)	11·61 (10·40–12·67)	17·53 (16·58–18·42)	12·54 (10·96–14·05)	16·50 (16·03–16·94)	12·16 (10·91–13·32)
	Palestine		15·00 (13·73–16·28)	10·89 (9·51–12·34)	14·34 (13·52–15·41)	10·64 (9·47–11·82)	14·74 (14·53–14·93)	10·71 (9·56–11·73)	12·89 (12·77–13·01)	9·58 (8·66–10·41)	13·60 (13·20–14·08)	9·83 (8·77–10·82)	12·89 (12·58–13·26)	9·51 (8·54–10·36)
	Oman		16·33 (15·44–17·07)	11·56 (10·15–12·91)	15·16 (14·75–15·63)	10·98 (9·77–12·09)	17·98 (17·43–18·72)	12·83 (11·35–14·25)	15·39 (14·77–16·00)	11·27 (10·08–12·40)	18·96 (18·60–19·48)	13·60 (12·06–14·99)	16·04 (15·69–16·52)	11·78 (10·58–12·93)
	Qatar		16·65 (15·53–18·29)	11·67 (10·08–13·46)	16·98 (15·63–18·37)	12·19 (10·65–13·70)	18·50 (17·66–19·71)	12·87 (11·25–14·40)	16·80 (14·98–18·63)	12·09 (10·20–13·90)	20·26 (18·41–22·92)	14·26 (12·18–16·59)	18·65 (16·07–21·28)	13·53 (11·23–15·85)
	Saudi Arabia		16·00 (15·23–16·82)	11·33 (10·04–12·68)	15·74 (15·05–16·52)	11·54 (10·23–12·80)	14·17 (13·81–14·55)	10·19 (9·07–11·21)	15·17 (14·82–15·53)	11·28 (10·17–12·29)	16·76 (16·12–17·49)	12·11 (10·82–13·42)	16·20 (15·60–16·83)	12·09 (10·88–13·25)
	Sudan		13·16 (12·63–13·67)	9·33 (8·29–10·38)	13·11 (12·83–13·41)	9·53 (8·51–10·50)	14·42 (13·86–15·03)	10·28 (9·12–11·33)	13·82 (13·50–14·28)	10·13 (9·06–11·08)	15·94 (15·13–16·53)	11·37 (10·07–12·58)	14·66 (14·23–15·09)	10·76 (9·61–11·79)
	Syria		16·36 (15·64–17·09)	11·73 (10·38–13·00)	15·01 (14·70–15·35)	11·12 (10·03–12·15)	17·39 (16·84–17·82)	12·62 (11·25–13·85)	15·80 (15·31–16·25)	11·83 (10·64–12·92)	18·11 (17·61–18·87)	13·16 (11·79–14·53)	14·98 (14·34–15·88)	11·26 (10·11–12·48)
	Tunisia		17·09 (16·46–17·78)	12·33 (10·90–13·71)	14·63 (13·97–15·32)	10·82 (9·65–11·91)	18·77 (18·22–20·04)	13·60 (12·08–15·20)	16·29 (15·52–17·38)	12·03 (10·72–13·37)	19·38 (18·34–21·04)	14·09 (12·37–16·01)	16·07 (14·63–17·96)	11·98 (10·39–13·69)
	Turkey		18·63 (18·02–19·22)	13·03 (11·49–14·46)	15·76 (15·30–16·22)	11·42 (10·19–12·57)	20·50 (19·87–21·15)	14·55 (12·80–16·16)	16·83 (16·41–17·34)	12·40 (11·17–13·55)	21·09 (19·63–22·58)	15·03 (13·16–16·95)	17·32 (15·87–18·72)	12·82 (11·25–14·43)
	United Arab Emirates		15·95 (14·69–17·58)	11·24 (9·71–12·78)	15·28 (13·37–17·00)	11·05 (9·25–12·83)	18·38 (17·63–18·78)	13·05 (11·59–14·44)	16·29 (15·71–16·82)	11·84 (10·58–13·03)	18·38 (17·08–19·44)	13·15 (11·56–14·77)	16·00 (14·78–17·04)	11·73 (10·23–13·06)
	Yemen		12·62 (11·92–13·47)	8·60 (7·42–9·84)	13·08 (12·62–13·58)	9·23 (8·14–10·29)	13·36 (12·97–13·99)	9·18 (8·00–10·29)	13·98 (13·56–14·42)	9·96 (8·80–11·02)	14·13 (13·58–14·54)	9·78 (8·54–10·85)	14·54 (13·60–15·37)	10·45 (9·18–11·66)
**South Asia**			**12·72 (12·50–12·93)**	**9·03 (8·05–9·92)**	**12·13 (11·95–12·32)**	**8·79 (7·91–9·59)**	**14·25 (14·04–14·46)**	**10·15 (9·10–11·11)**	**13·15 (12·97–13·33)**	**9·55 (8·65–10·39)**	**15·20 (14·95–15·46)**	**10·84 (9·72–11·89)**	**13·80 (13·58–14·01)**	**10·03 (9·06–10·87)**
	Bangladesh		12·85 (12·14–13·57)	9·29 (8·24–10·32)	12·78 (12·25–13·38)	9·44 (8·44–10·39)	16·29 (15·70–16·71)	11·78 (10·52–12·93)	14·43 (14·00–14·92)	10·73 (9·70–11·65)	17·59 (16·60–18·34)	12·76 (11·42–14·11)	15·35 (14·45–16·11)	11·44 (10·25–12·56)
	Bhutan		13·21 (11·99–14·44)	9·44 (8·18–10·72)	14·26 (13·14–15·32)	10·39 (9·15–11·56)	17·01 (16·06–17·93)	12·20 (10·74–13·53)	16·00 (15·06–16·91)	11·74 (10·46–13·07)	18·12 (16·85–19·04)	13·01 (11·50–14·54)	16·25 (15·21–17·40)	11·92 (10·56–13·23)
	India		12·57 (12·31–12·83)	8·88 (7·89–9·77)	11·82 (11·62–12·02)	8·52 (7·68–9·30)	14·11 (13·88–14·36)	10·02 (8·97–10·99)	12·97 (12·76–13·17)	9·36 (8·48–10·19)	15·03 (14·75–15·31)	10·68 (9·54–11·73)	13·59 (13·37–13·82)	9·82 (8·87–10·67)
	Nepal		12·41 (11·89–12·95)	9·01 (8·08–9·96)	12·65 (12·25–13·08)	9·23 (8·33–10·10)	14·02 (13·54–14·63)	10·20 (9·12–11·26)	13·94 (13·53–14·31)	10·26 (9·27–11·25)	15·17 (14·78–15·68)	11·04 (9·94–12·08)	14·74 (14·00–15·59)	10·89 (9·75–12·07)
	Pakistan		14·20 (13·66–14·72)	10·32 (9·18–11·37)	14·12 (13·60–14·64)	10·42 (9·35–11·46)	13·81 (13·02–14·71)	10·02 (8·89–11·22)	13·36 (12·92–13·88)	9·86 (8·89–10·81)	14·78 (13·59–16·30)	10·73 (9·35–12·19)	14·06 (13·20–14·86)	10·45 (9·31–11·57)
**Sub-Saharan Africa**			**12·74 (12·55–12·91)**	**9·47 (8·54–10·30)**	**12·58 (12·37–12·79)**	**9·28 (8·40–10·14)**	**13·08 (12·84–13·32)**	**9·77 (8·86–10·62)**	**12·68 (12·50–12·86)**	**9·41 (8·49–10·24)**	**14·39 (14·11–14·69)**	**10·82 (9·81–11·76)**	**13·67 (13·39–13·96)**	**10·19 (9·19–11·10)**
	Southern sub-Saharan Africa		17·26 (16·87–17·77)	12·84 (11·61–13·96)	14·86 (14·48–15·50)	11·01 (9·97–12·04)	14·05 (13·62–14·53)	10·38 (9·39–11·41)	11·54 (11·35–11·75)	8·46 (7·63–9·24)	16·73 (16·29–17·22)	12·38 (11·16–13·54)	13·40 (13·08–13·78)	9·82 (8·86–10·75)
		Botswana	14·16 (13·28–15·74)	10·64 (9·49–12·02)	12·46 (11·16–14·04)	9·30 (8·03–10·69)	13·22 (9·32–16·70)	9·97 (6·91–12·50)	10·59 (8·92–12·66)	7·74 (6·34–9·56)	15·80 (12·73–22·97)	11·93 (9·25–16·98)	12·81 (11·28–15·95)	9·42 (7·95–11·65)
		Lesotho	13·46 (12·52–14·91)	10·05 (8·86–11·33)	11·00 (10·18–12·26)	8·22 (7·27–9·43)	11·03 (9·65–12·89)	8·14 (6·84–9·65)	8·55 (8·05–9·14)	6·24 (5·52–6·91)	12·12 (10·11–15·73)	8·99 (7·28–11·33)	9·41 (8·57–10·38)	6·87 (6·00–7·78)
		Namibia	13·16 (12·77–13·76)	9·91 (8·96–10·81)	11·31 (10·78–12·16)	8·46 (7·58–9·42)	13·82 (11·53–16·20)	10·43 (8·36–12·57)	10·66 (9·92–11·95)	7·90 (6·93–8·97)	16·82 (14·39–21·51)	12·83 (10·52–16·49)	12·69 (11·71–14·15)	9·48 (8·32–10·77)
		South Africa	18·40 (18·01–18·79)	13·63 (12·33–14·85)	15·44 (15·09–15·77)	11·40 (10·33–12·40)	14·77 (14·58–14·97)	10·89 (9·86–11·85)	12·30 (12·15–12·47)	8·98 (8·13–9·81)	17·65 (17·26–18·05)	13·01 (11·70–14·24)	14·15 (13·83–14·47)	10·30 (9·29–11·29)
		Swaziland	13·21 (12·25–14·33)	9·88 (8·84–11·02)	11·47 (10·53–12·85)	8·51 (7·48–9·70)	11·34 (8·96–12·71)	8·32 (6·53–9·66)	9·12 (8·24–10·46)	6·51 (5·58–7·59)	14·69 (11·75–19·10)	10·94 (8·33–14·11)	11·06 (9·92–13·44)	8·00 (6·83–9·62)
		Zimbabwe	14·08 (13·05–16·47)	10·73 (9·46–12·72)	14·17 (12·92–18·34)	10·74 (9·24–13·75)	11·44 (9·75–13·85)	8·70 (7·21–10·70)	9·68 (9·09–10·45)	7·25 (6·43–8·12)	13·23 (11·75–15·66)	10·08 (8·79–11·82)	11·58 (10·54–13·13)	8·77 (7·63–10·19)
	Western sub-Saharan Africa		13·26 (12·87–13·57)	9·75 (8·69–10·69)	13·41 (12·98–13·82)	9·81 (8·75–10·80)	13·96 (13·52–14·50)	10·35 (9·33–11·37)	13·66 (13·28–14·12)	10·09 (9·09–11·09)	14·99 (14·49–15·59)	11·19 (10·09–12·24)	14·55 (14·02–15·07)	10·82 (9·73–11·84)
		Benin	13·82 (13·26–14·30)	9·99 (8·81–11·00)	12·69 (12·26–13·18)	9·14 (8·10–10·11)	14·25 (13·38–15·11)	10·46 (9·29–11·58)	12·69 (12·42–13·14)	9·30 (8·36–10·19)	14·29 (13·38–15·41)	10·59 (9·32–11·79)	13·34 (12·96–13·76)	9·84 (8·79–10·78)
		Burkina Faso	12·40 (11·91–13·17)	8·98 (7·91–10·05)	12·23 (11·74–13·15)	8·77 (7·71–9·88)	12·97 (12·44–13·46)	9·62 (8·60–10·58)	12·76 (12·43–13·05)	9·39 (8·42–10·30)	13·30 (12·88–13·77)	9·97 (9·00–10·91)	13·17 (12·89–13·50)	9·83 (8·88–10·73)
		Cameroon	13·39 (12·57–14·32)	9·84 (8·63–10·89)	13·20 (12·62–14·04)	9·66 (8·58–10·75)	13·02 (11·61–14·67)	9·67 (8·30–11·14)	12·33 (11·78–12·90)	9·08 (8·12–9·98)	14·03 (12·41–16·13)	10·55 (8·94–12·39)	12·92 (12·13–13·78)	9·61 (8·58–10·65)
		Cape Verde	16·82 (16·36–17·50)	12·57 (11·34–13·76)	13·64 (13·30–14·20)	10·22 (9·26–11·18)	18·52 (17·54–19·66)	13·89 (12·35–15·40)	13·37 (12·54–15·40)	10·05 (8·97–11·50)	18·81 (18·11–20·06)	14·20 (12·74–15·70)	13·99 (13·16–15·03)	10·55 (9·38–11·70)
		Chad	13·10 (12·56–13·70)	9·58 (8·49–10·56)	12·80 (12·43–13·23)	9·33 (8·30–10·25)	13·59 (12·63–14·50)	9·91 (8·71–11·12)	12·52 (11·98–13·13)	9·12 (8·10–10·07)	14·31 (13·21–15·24)	10·53 (9·28–11·82)	13·14 (12·60–13·96)	9·64 (8·60–10·67)
		Côte d'Ivoire	12·50 (11·87–13·22)	8·95 (7·86–10·06)	11·99 (11·62–12·35)	8·59 (7·60–9·49)	12·16 (11·28–13·45)	9·02 (7·95–10·20)	11·80 (11·06–12·26)	8·66 (7·65–9·57)	12·99 (12·23–14·00)	9·68 (8·59–10·82)	12·58 (11·69–13·06)	9·29 (8·14–10·21)
		The Gambia	13·49 (12·43–14·56)	9·92 (8·68–11·15)	13·98 (13·17–14·71)	10·38 (9·17–11·49)	14·45 (13·37–15·71)	10·63 (9·35–12·04)	13·74 (13·29–14·36)	10·19 (9·10–11·18)	14·90 (14·03–16·33)	11·07 (9·80–12·57)	14·50 (13·75–15·08)	10·81 (9·61–11·81)
		Ghana	12·72 (11·81–13·84)	9·50 (8·43–10·65)	13·03 (12·47–13·83)	9·68 (8·64–10·76)	13·55 (12·53–15·05)	10·28 (9·00–11·72)	12·93 (12·69–13·35)	9·70 (8·75–10·56)	14·31 (13·63–15·75)	10·88 (9·79–12·28)	13·93 (13·19–14·59)	10·44 (9·34–11·45)
		Guinea	12·55 (11·79–13·20)	9·24 (8·22–10·27)	13·69 (13·08–14·38)	10·07 (8·99–11·13)	12·77 (11·85–13·85)	9·53 (8·39–10·57)	12·78 (12·21–13·55)	9·54 (8·47–10·54)	13·00 (12·02–14·44)	9·77 (8·61–11·03)	13·10 (12·52–13·93)	9·80 (8·72–10·84)
		Guinea-Bissau	11·17 (10·40–12·25)	8·28 (7·27–9·32)	10·81 (10·12–11·46)	7·95 (7·04–8·81)	11·67 (10·81–13·00)	8·68 (7·64–9·85)	10·83 (10·08–11·85)	8·02 (7·10–9·02)	12·43 (11·57–13·29)	9·32 (8·21–10·33)	11·07 (10·36–12·17)	8·24 (7·28–9·25)
		Liberia	12·87 (12·19–13·55)	9·19 (8·12–10·24)	13·32 (12·76–13·88)	9·44 (8·35–10·51)	12·85 (11·97–14·00)	9·26 (8·08–10·42)	13·39 (12·82–14·01)	9·62 (8·49–10·70)	13·39 (12·92–14·05)	9·80 (8·69–10·83)	13·87 (13·47–14·35)	10·11 (9·03–11·10)
		Mali	11·41 (10·78–12·08)	8·45 (7·48–9·39)	12·64 (12·18–13·24)	9·22 (8·21–10·21)	13·50 (12·59–14·36)	10·03 (8·88–11·14)	14·23 (13·49–14·98)	10·45 (9·31–11·53)	14·28 (12·68–15·69)	10·70 (9·24–12·11)	14·69 (13·35–16·01)	10·91 (9·54–12·34)
		Mauritania	12·41 (11·70–13·18)	9·15 (8·13–10·19)	13·14 (12·66–13·85)	9·59 (8·50–10·66)	14·11 (13·03–15·46)	10·45 (9·16–11·71)	15·56 (14·62–16·62)	11·41 (10·07–12·76)	15·12 (13·82–17·09)	11·31 (9·75–13·01)	16·12 (14·81–17·86)	11·90 (10·35–13·61)
		Niger	13·13 (12·55–13·58)	9·80 (8·78–10·74)	12·73 (12·10–13·48)	9·45 (8·40–10·48)	13·86 (12·92–14·49)	10·38 (9·23–11·42)	13·34 (12·71–14·10)	9·97 (8·96–10·96)	14·14 (12·56–15·46)	10·68 (9·22–12·03)	13·47 (12·69–14·49)	10·11 (9·00–11·35)
		Nigeria	13·88 (12·99–14·54)	10·20 (9·05–11·27)	14·32 (13·34–15·44)	10·51 (9·27–11·90)	15·00 (14·02–16·49)	11·09 (9·84–12·49)	14·95 (13·99–16·27)	11·05 (9·81–12·48)	16·78 (15·59–18·56)	12·49 (10·97–14·15)	16·32 (14·96–17·83)	12·14 (10·60–13·64)
		São Tomé and Príncipe	14·67 (13·71–15·41)	11·04 (9·88–12·18)	14·05 (13·33–14·80)	10·41 (9·27–11·53)	13·91 (13·52–14·37)	10·51 (9·54–11·41)	13·97 (13·51–14·51)	10·38 (9·32–11·34)	15·21 (14·52–16·23)	11·55 (10·38–12·75)	14·59 (13·22–15·99)	10·89 (9·37–12·43)
		Senegal	13·25 (12·57–13·91)	9·89 (8·85–10·95)	12·63 (12·28–13·06)	9·37 (8·38–10·26)	13·28 (12·72–14·02)	9·96 (8·93–11·01)	13·02 (12·82–13·40)	9·69 (8·68–10·58)	13·86 (13·33–14·26)	10·42 (9·40–11·37)	13·62 (13·00–14·31)	10·17 (9·08–11·22)
		Sierra Leone	13·64 (13·11–14·12)	10·11 (9·03–11·09)	12·33 (11·78–12·96)	9·01 (7·96–9·99)	12·39 (11·59–13·22)	9·17 (8·13–10·23)	12·03 (11·70–12·50)	8·79 (7·85–9·68)	12·93 (11·99–14·20)	9·70 (8·58–10·91)	12·86 (12·42–13·49)	9·52 (8·46–10·54)
		Togo	13·00 (12·26–13·76)	9·63 (8·59–10·69)	12·81 (12·34–13·30)	9·47 (8·41–10·42)	13·60 (12·35–15·28)	10·15 (8·83–11·60)	12·22 (11·58–12·65)	9·10 (8·16–10·03)	13·81 (13·12–15·13)	10·44 (9·27–11·72)	12·82 (11·89–13·45)	9·64 (8·58–10·61)
	Eastern sub-Saharan Africa		11·12 (10·88–11·37)	8·41 (7·63–9·10)	11·42 (11·16–11·72)	8·54 (7·69–9·31)	12·35 (11·95–12·78)	9·37 (8·48–10·18)	12·29 (12·03–12·52)	9·23 (8·34–10·02)	13·67 (13·20–14·20)	10·43 (9·47–11·34)	13·18 (12·71–13·64)	9·94 (8·96–10·81)
		Burundi	9·43 (8·73–10·38)	7·25 (6·51–8·09)	9·51 (8·67–10·67)	7·21 (6·37–8·26)	11·02 (10·25–11·74)	8·49 (7·62–9·38)	11·46 (10·48–12·32)	8·73 (7·60–9·77)	12·09 (11·16–13·02)	9·39 (8·35–10·40)	12·29 (11·00–13·34)	9·42 (8·29–10·56)
		Comoros	12·25 (11·44–13·47)	9·26 (8·20–10·46)	12·63 (12·08–13·35)	9·35 (8·37–10·29)	13·82 (12·96–14·96)	10·53 (9·39–11·83)	13·71 (13·27–14·51)	10·31 (9·29–11·38)	14·31 (13·57–15·90)	10·96 (9·83–12·39)	14·28 (13·44–15·15)	10·79 (9·64–11·90)
		Djibouti	14·87 (14·23–15·29)	11·27 (10·14–12·31)	12·89 (12·58–13·36)	9·75 (8·81–10·66)	14·72 (12·98–16·44)	11·21 (9·45–12·88)	13·05 (12·38–13·73)	9·86 (8·78–10·82)	15·34 (13·92–17·48)	11·73 (10·11–13·71)	13·91 (12·86–15·01)	10·52 (9·34–11·78)
		Eritrea	10·29 (9·80–10·84)	7·80 (7·00–8·61)	10·64 (9·97–11·34)	7·99 (7·13–8·88)	12·03 (11·31–12·92)	9·19 (8·18–10·21)	12·61 (11·58–13·31)	9·50 (8·41–10·50)	12·49 (11·73–13·45)	9·61 (8·63–10·69)	12·75 (11·68–13·73)	9·67 (8·49–10·76)
		Ethiopia	9·54 (9·06–10·09)	7·25 (6·56–7·97)	9·78 (9·24–10·50)	7·35 (6·56–8·14)	11·41 (10·61–12·18)	8·73 (7·79–9·69)	12·47 (11·67–12·84)	9·43 (8·46–10·31)	13·26 (12·20–14·32)	10·18 (9·02–11·38)	13·32 (12·03–14·75)	10·12 (8·80–11·52)
		Kenya	13·41 (13·09–13·74)	10·31 (9·40–11·15)	13·74 (13·38–14·10)	10·44 (9·47–11·32)	14·05 (13·65–14·44)	10·79 (9·84–11·66)	13·14 (12·83–13·42)	9·97 (9·05–10·78)	15·37 (14·93–15·85)	11·79 (10·73–12·79)	14·14 (13·81–14·51)	10·69 (9·71–11·58)
		Madagascar	11·92 (11·31–12·61)	9·05 (8·19–10·01)	12·13 (11·79–12·43)	9·11 (8·22–9·93)	12·57 (11·72–13·83)	9·61 (8·49–10·80)	12·78 (12·19–13·40)	9·66 (8·70–10·60)	13·10 (11·71–15·54)	10·11 (8·76–12·00)	13·05 (11·51–14·31)	9·93 (8·66–11·13)
		Malawi	12·06 (10·88–13·26)	9·11 (7·97–10·40)	13·32 (11·80–16·19)	9·89 (8·35–12·03)	12·63 (10·45–14·93)	9·63 (7·73–11·73)	11·51 (10·27–13·33)	8·57 (7·39–10·06)	13·97 (12·43–16·42)	10·67 (9·21–12·67)	12·90 (11·40–14·06)	9·70 (8·43–10·99)
		Mozambique	12·71 (12·07–13·26)	9·51 (8·52–10·49)	11·86 (11·50–12·31)	8·76 (7·87–9·60)	14·52 (12·48–16·02)	10·81 (8·97–12·42)	11·94 (10·91–12·86)	8·76 (7·56–9·86)	14·76 (13·22–16·48)	11·05 (9·57–12·65)	13·01 (11·80–14·04)	9·57 (8·27–10·72)
		Rwanda	10·17 (9·56–10·82)	7·74 (6·92–8·55)	10·69 (9·78–11·65)	8·06 (7·08–9·05)	14·05 (12·83–15·21)	10·69 (9·41–11·91)	13·03 (12·64–13·77)	9·82 (8·87–10·78)	15·00 (13·91–16·44)	11·48 (10·13–12·98)	14·27 (13·24–15·18)	10·77 (9·57–11·89)
		Somalia	10·21 (9·54–10·86)	7·76 (6·89–8·58)	11·58 (10·94–12·19)	8·72 (7·78–9·63)	10·34 (9·69–11·08)	7·87 (7·03–8·86)	11·59 (10·64–12·32)	8·77 (7·76–9·78)	10·85 (10·08–11·56)	8·29 (7·35–9·17)	11·93 (10·81–12·75)	9·03 (7·87–10·03)
		South Sudan	12·45 (11·38–13·56)	8·77 (7·43–10·01)	12·70 (11·77–13·88)	8·89 (7·68–10·13)	12·72 (11·39–14·54)	9·21 (7·81–10·90)	12·72 (11·88–13·89)	9·13 (7·99–10·42)	12·86 (11·61–14·69)	9·50 (8·22–11·12)	12·94 (12·02–13·95)	9·44 (8·33–10·58)
		Tanzania	12·46 (11·77–13·34)	9·43 (8·40–10·53)	12·65 (12·07–13·54)	9·49 (8·47–10·54)	13·15 (11·96–15·19)	10·01 (8·70–11·77)	12·80 (12·36–13·65)	9·66 (8·66–10·64)	14·10 (13·34–15·98)	10·80 (9·62–12·29)	13·64 (12·43–14·45)	10·38 (9·17–11·42)
		Uganda	10·95 (10·45–11·64)	8·14 (7·30–9·03)	11·31 (10·58–12·11)	8·27 (7·27–9·25)	12·01 (11·33–12·82)	9·01 (8·03–10·01)	11·81 (10·83–12·44)	8·76 (7·74–9·66)	13·61 (12·73–15·05)	10·35 (9·21–11·69)	12·79 (11·72–13·52)	9·58 (8·43–10·61)
		Zambia	11·59 (10·67–13·24)	8·88 (7·83–10·24)	14·29 (13·12–15·19)	10·74 (9·49–11·99)	10·41 (9·17–12·23)	7·93 (6·83–9·42)	9·47 (8·91–10·43)	7·12 (6·34–7·99)	13·01 (11·11–16·03)	9·95 (8·29–12·45)	10·99 (9·92–12·90)	8·27 (7·19–9·81)
	Central sub-Saharan Africa		11·66 (11·23–12·15)	8·47 (7·56–9·35)	11·32 (11·00–11·66)	8·14 (7·24–8·98)	11·65 (11·24–12·11)	8·56 (7·67–9·40)	12·10 (11·81–12·39)	8·80 (7·89–9·69)	12·76 (12·32–13·22)	9·46 (8·51–10·35)	12·88 (12·42–13·25)	9·46 (8·45–10·39)
		Angola	11·07 (9·98–12·74)	8·23 (7·17–9·58)	10·78 (9·56–11·90)	7·98 (6·87–9·08)	11·57 (10·28–13·28)	8·67 (7·46–10·05)	12·53 (11·28–13·71)	9·30 (8·12–10·51)	13·33 (11·70–15·06)	9·99 (8·59–11·47)	13·62 (11·94–15·00)	10·11 (8·69–11·47)
		Central African Republic	10·33 (9·69–11·07)	7·60 (6·71–8·45)	9·17 (8·60–9·84)	6·70 (5·91–7·52)	10·17 (8·73–12·21)	7·56 (6·31–9·11)	8·94 (8·24–10·36)	6·58 (5·71–7·76)	10·65 (9·27–12·61)	7·97 (6·73–9·62)	9·38 (8·61–10·77)	6·95 (6·09–8·16)
		Congo (Brazzaville)	10·23 (9·47–11·12)	7·54 (6·62–8·50)	9·93 (9·32–10·88)	7·27 (6·36–8·22)	11·08 (10·24–12·10)	8·21 (7·19–9·20)	12·97 (12·28–13·52)	9·54 (8·50–10·51)	12·36 (11·03–13·94)	9·22 (7·89–10·70)	13·89 (12·35–15·53)	10·32 (8·97–11·90)
		Democratic Republic of the Congo	12·12 (11·58–12·81)	8·73 (7·67–9·72)	12·06 (11·75–12·46)	8·55 (7·57–9·47)	11·83 (11·37–12·42)	8·65 (7·71–9·57)	12·25 (11·94–12·49)	8·84 (7·89–9·74)	12·78 (12·31–13·29)	9·45 (8·42–10·39)	12·92 (12·42–13·33)	9·46 (8·43–10·38)
		Equatorial Guinea	9·99 (9·12–11·11)	7·42 (6·43–8·39)	9·97 (8·98–11·29)	7·37 (6·34–8·47)	13·95 (11·33–16·90)	10·37 (8·18–12·78)	14·22 (12·47–16·55)	10·46 (8·83–12·43)	15·71 (13·12–19·29)	11·71 (9·45–14·15)	15·35 (13·29–18·00)	11·33 (9·53–13·46)
		Gabon	12·61 (11·83–13·42)	9·26 (8·22–10·27)	10·76 (10·09–11·80)	7·93 (7·01–8·93)	12·21 (11·38–13·06)	8·98 (7·94–10·04)	13·15 (11·99–13·91)	9·72 (8·51–10·79)	14·23 (13·07–16·01)	10·51 (9·24–11·89)	14·30 (12·61–16·05)	10·62 (9·01–12·20)

Data in parentheses are 95% uncertainty intervals. To download the data in this table, please visit the Global Health Data Exchange. GBD=Global Burden of Disease. HALE=healthy life expectancy. SDI=Socio-demographic index.

**Figure 1:**
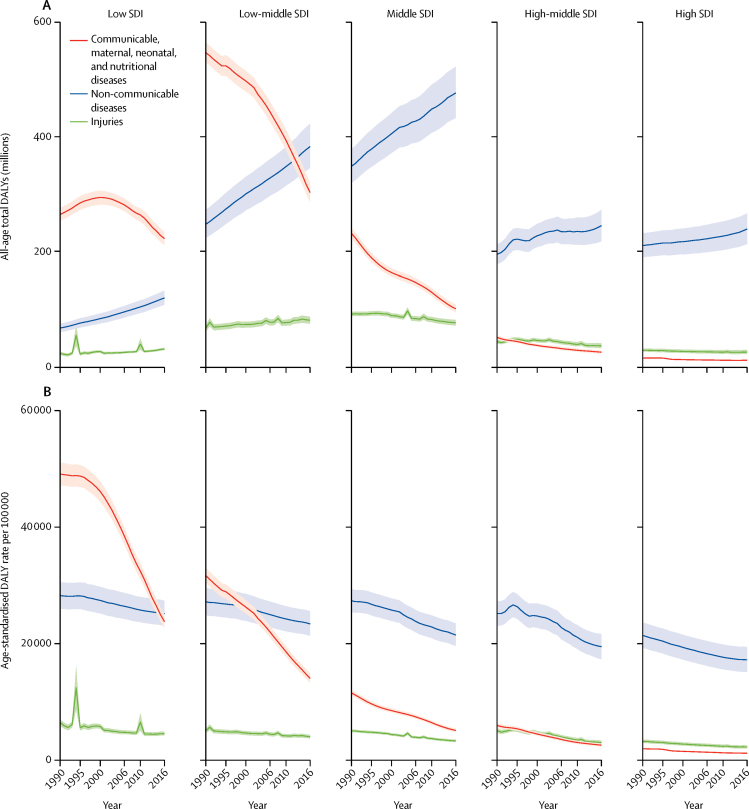
Trends of total DALYs (A) and age-standardised DALY rates (B) from 1990 to 2016 for GBD Level 1 cause groups by SDI quintile Shaded areas show 95% uncertainty intervals. DALYs=disability-adjusted life-years. GBD=Global Burden of Disease. SDI=Socio-demographic Index.

Global trends for all-age DALYs and age-standardised DALY rates for Level 1 causes by SDI quintile are shown in figure 1. Trends in total DALYs, which show the absolute burden at each SDI quintile, are shown in figure 1A. The figure highlights the large burden and subsequent declines in low-middle-SDI (decreased by 44·5% [95% UI 41·3–47·6]) and middle-SDI (decreased by 56·2% [53·6–59·0]) locations for CMNN DALYs from 1990 to 2016, offset by large increases for NCD DALYs over the same time period in low-middle SDI (increased by 54·4% [47·9–60·8]) and middle-SDI (increased by 36·5% [39·9–32·8]) locations. Progress has been made in low-SDI countries for CMNN DALYs, which decreased by 20·7% (16·8–24·7) since 2006. At all levels of SDI, total NCD DALYs have increased since 1990. Trends in age-standardised rates—which account for both population size and age structure—emphasise the reduction in the contribution of CMNN causes to DALYs, both over time and with increasing SDI (figure 1B). These rapid decreases in age-standardised rates were fastest at low SDI, where age-standardised DALY rates from CMNN causes decreased by 51·5% (49·0–54·1) between 1990 and 2016 to be on par with age-standardised DALY rates for NCDs in 2016. At low-middle SDI, age-standardised CMNN DALY rates were more than double those for NCDs in 1990 (31·7 thousand [30·3 thousand to 33·3 thousand] per 100 000), but decreased to 14·1 thousand (13·2 thousand to 15·1 thousand) per 100 000 in 2016. At all other levels of SDI, age-standardised DALY rates for CMNN causes were lower than those for NCDs and lower than those of injuries in the high-SDI quintile. Reductions in age-standardised rates for NCDs occurred across all levels of SDI between 1990 and 2016, a trend that was also evident, albeit less strongly, for age-standardised DALY rates from injuries.

### Global causes of DALYs

Age-standardised DALY rates for all causes decreased by 30·5% (95% UI 28·6–32·6) between 1990 and 2016 (appendix pp 49–62). In 2016, CMNN causes accounted for 28·0% (26·4–29·7) of global DALYs, NCDs contributed 61·4% (59·4–63·2), and injuries contributed 10·7% (10·1–11·3; appendix pp 35–48). From 2006 to 2016, CMNN causes decreased by 31·9% (29·7–34·2), with 48 Level 4 CMNN causes experiencing decreases in age-standardised DALY rates of greater than 20% (table 1). Decreases were greater than 70% for three infectious diseases: Guinea worm disease (decreased by 99·6% [99·5–99·7]), human African trypanosomiasis (decreased by 78·2% [68·8–84·6]), and measles (decreased by 73·6% [68·8–77·8]). By contrast with the overall trend of decreasing DALYs, a subset of Level 4 CMNN causes had increases in age-standardised DALY rates, including dengue (50·5% [24·7–97·7]) and cutaneous and mucocutaneous leishmaniasis (12·5% [1·7–26·1]). Overall, total all-age DALYs attributable to maternal disorders decreased by 23·9% (17·4–29·3) between 2006 and 2016 and by 30·4% (24·4–35·4) in terms of age-standardised DALY rates. As a cause group, neonatal disorders decreased by 22·8% (18·9–26·8) in all-age DALYs and 23·1% (19·2–27·0) in terms of age-standardised DALY rates over the same time period; however, this decrease was not significant for neonatal sepsis. Total DALYs from the London Declaration NTDs was 9·0 million (5·3 million to 14·5 million) in 2016.

In 2016, the leading Level 3 causes of total DALYs among NCDs included ischaemic heart disease (175 million [95% UI 170 million to 180 million] DALYs), cerebrovascular disease (116 million [111 million to 121 million]), and low back and neck pain (87 million [61 million to 114 million]), comprising 16·1% (13·99–17·67) of all DALYs (table 1). Among chronic respiratory diseases, all causes, with the exception of interstitial lung disease, pulmonary sarcoidosis, and other chronic respiratory diseases, decreased in age-standardised DALY rates between 2006 and 2016, whereas total all-age DALY counts increased from 2006 to 2016 for all chronic respiratory diseases, with the exception of silicosis. Cirrhosis and other chronic liver diseases had a mean change of 7·6% (2·5–13·7) from 2006 to 2016 in total all-age DALY counts, but had a mean decrease in age-standardised DALY rates of 12·0% (7·2–16·1) over the same period. Age-standardised DALY rates of digestive diseases decreased from 2006 to 2016, with a mean percentage decrease of 13·6% (10·8–16·4); however, all-age DALY counts for digestive diseases increased by 4·1% (0·4–7·7) over the same period. Total DALYs associated with most neurological disorders increased from 2006 to 2016, with Alzheimer's disease and other dementias (increase of 37·5% [35·3–39·7]) and Parkinson's disease (increase of 35·6% [32·9–38·2]) increasing by more than 30% each. Between 2006 and 2016, various NCDs significantly increased in terms of both total burden and age-standardised DALY rates. Several mental and substance use disorders followed this pattern, including eating disorders (age-standardised DALY rates increased by 8·9% [7·6–10·1]) and bipolar disorder (increased by 0·8% [0·2–1·4]). Diabetes (all-age DALY count increased by 24·4% [22·7–26·2]) and chronic kidney disease (increased by 20·0% [17·4–22·7]) both also increased in all-age DALY counts, as did musculoskeletal disorders (increased by 19·6% [18·5–20·8]).

Percentage change in age-standardised DALY rates of unintentional injuries (decreased by 15·1% [95% UI 12·2–18·0]), road injuries (decreased by 14·7% [12·8–16·8]), and transport injuries (decreased by 14·3% [12·3–16·4]) each decreased substantially between 2006 and 2016 (table 1). Among unintentional injuries, drowning had the largest reduction in both all-age DALY burden (26·6% [20·1–30·1]) and age-standardised DALY rates (32·1% [26·1–35·4]) from 2006 to 2016. Age-standardised DALY rates from self-harm (decreased by 17·7% [12·9–21·2]) and interpersonal violence (12·0% [7·0–16·1]) have both decreased by more than 10% since 2006. Age-standardised DALY rates resulting from conflict and terrorism increased by 97·4% (25·7–240·9) from 2006 to 2016; this rise was primarily driven by ongoing conflicts in north Africa, the Middle East, and sub-Saharan Africa. This result represents an increase in all-age DALYs of 114·0% (36·2–271·3) from 2006 to 2016.

Global DALY counts in 2016 for causes that were estimated separately for the first time are as follows: alcoholic cardiomyopathy 2·59 million (95% UI 2·06 million to 3·24 million), urogenital congenital anomalies 1·09 million (0·86 million to 1·31 million), congenital musculoskeletal and limb anomalies 2·26 million (1·66 million to 2·99 million), digestive congenital anomalies 3·34 million (2·67 million to 4·96 million), Zika virus disease 5100 (3400–8000), Guinea worm disease 0·87 (0·5–1·35), self-harm by firearm 2·85 million (2·39 million to 3·59 million), sexual violence 1·37 million (0·92 million to 1·95 million), myocarditis 1·37 million (1·12 million to 1·51 million), drug-susceptible tuberculosis 39·9 million (38·1 million to 41·9 million), multidrug-resistant tuberculosis without extensive drug resistance 3·32 million (2·79 million to 3·91 million), extensively drug-resistant tuberculosis 369 000 (301 000–445 000), drug-susceptible HIV/AIDS-tuberculosis 11·7 million (8·15 million to 15·5 million), multidrug-resistant HIV/AIDS-tuberculosis without extensive drug resistance 0·98 million (0·60 million to 1·48 million), and extensively drug-resistant HIV/AIDS-tuberculosis 57 300 (34 500–89 400; table 1).

### Changes in leading causes of disease burden over time and SDI quintiles

**Figure 2:**
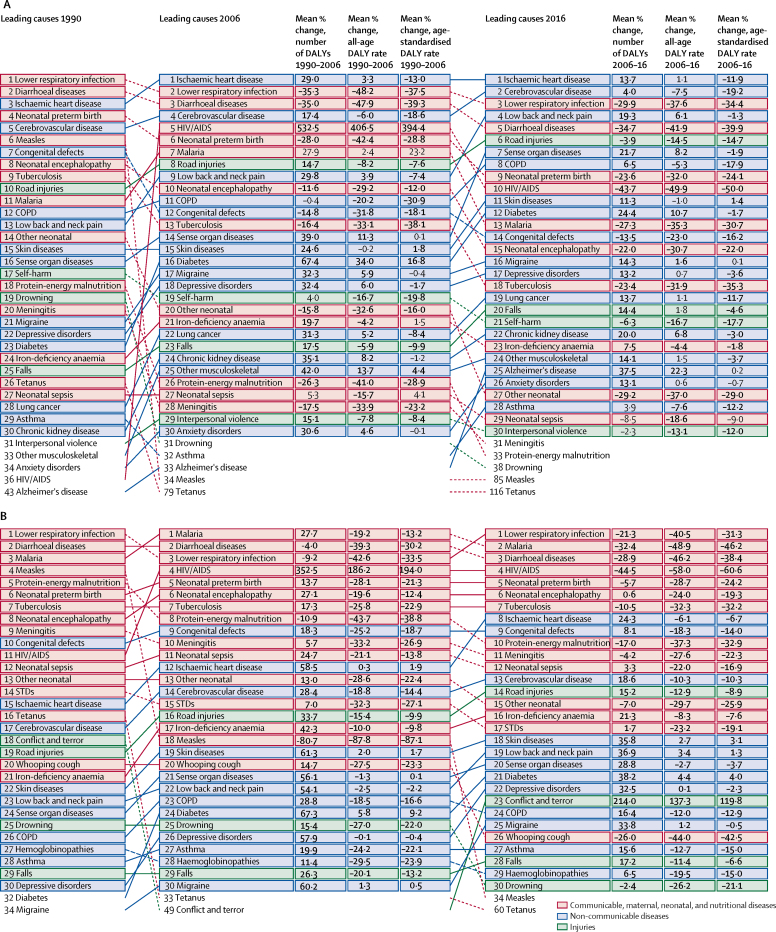

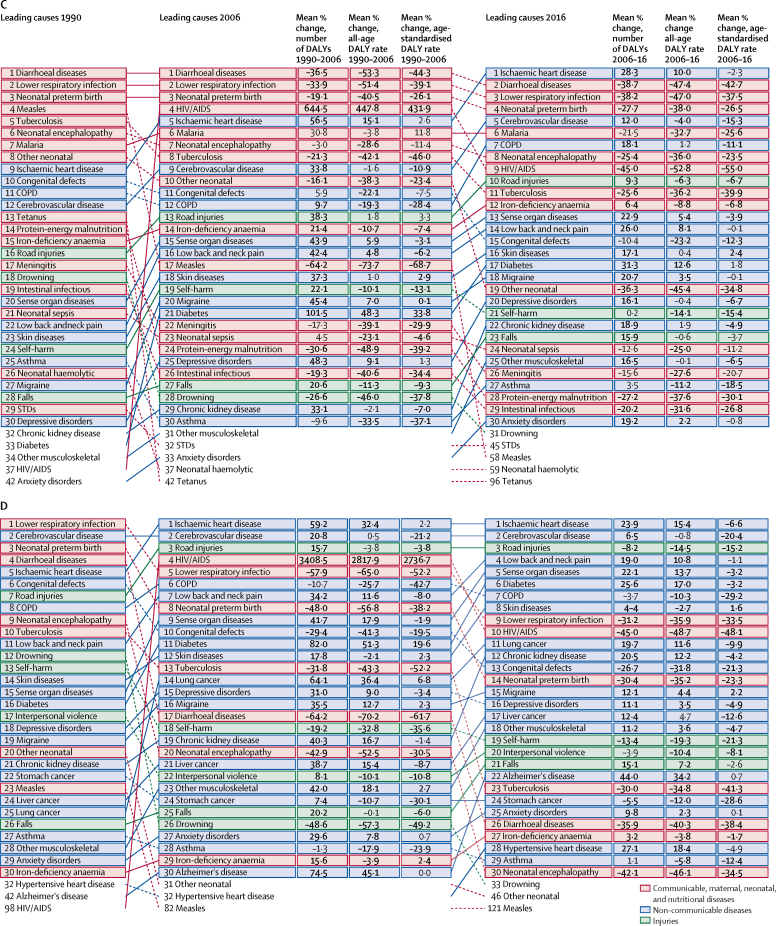

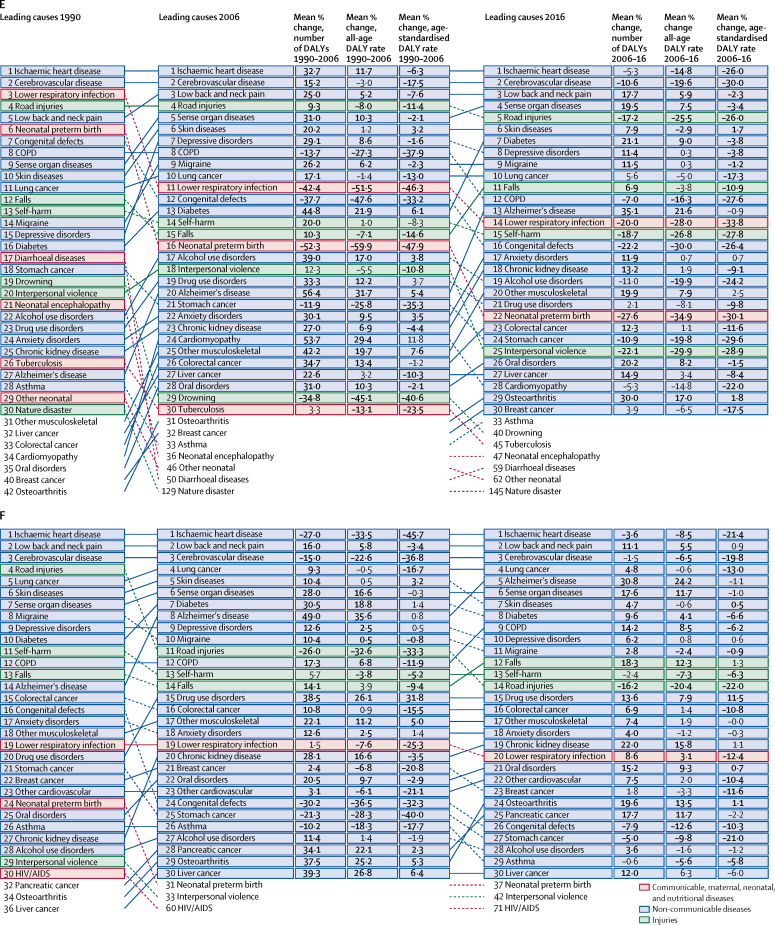
Leading 30 Level 3 causes of total DALYs for 1990, 2006, and 2016, with percentage change in number of DALYs and all-age and age-standardised DALY rates, overall and by SDI quintile Overall (A). Low SDI (B). Low-middle SDI (C). Middle SDI (D). High-middle SDI (E). High SDI (F). Causes are connected by lines between time periods; solid lines are increases and dashed lines are decreases. For the time period of 1990–2006 and 2006–16, three measures of change are shown: percentage change in the number of DALYs, percentage change in the all-age DALY rate, and percentage change in the age-standardised DALY rate. Statistically significant changes are shown in bold. COPD=chronic obstructive pulmonary disease. DALYs=disability-adjusted life-years. STDs=sexually transmitted diseases.

The leading Level 3 causes of global DALYs in 1990 were lower respiratory infections, diarrhoeal diseases, and ischaemic heart disease (figure 2A). By 2016, lower respiratory infections had decreased in relative rank to the third-leading cause, whereas diarrhoeal diseases were fifth and ischaemic heart disease rose to first. The leading 30 causes of DALYs by SDI quintile varied considerably (figure 2B–F). CMNN causes were dominant in the low-SDI quintile, occupying 11 of the top 15 causes in 2016. NCDs were more commonly among the leading causes of DALYs with successively higher levels of SDI, occupying 12 of the top 15 causes in the high-SDI quintile in 2016.

For low SDI, only two NCD causes (congenital birth defects and ischaemic heart disease) ranked in the top ten in 2016, compared with all of the top ten for high SDI. Of the leading 30 causes from 2006 to 2016, HIV/AIDS had the largest decrease in age-standardised DALY rates for low, low-middle, and middle SDI. For high-middle SDI, the largest decrease was for lower respiratory infection, and for high SDI, the largest decrease was for road injuries. At low-middle SDI, 14 of the leading 30 causes in 2016 were NCDs, whereas 13 were CMNN causes and three were injuries, compared with middle SDI, where seven were CMNN causes, 19 were NCDs, and four were injuries. The leading seven causes for low, low-middle, and middle SDI quintiles all decreased in age-standardised DALY rates from 2006 to 2016.

At high-middle and high SDI, the leading three causes of DALYs remained the same from 2006 to 2016 (ischaemic heart disease, low back and neck pain, and cerebrovascular disease). Two of the leading 30 causes for high-middle SDI were CMNN causes in 2016, whereas 24 were NCDs and four were injuries, compared with high SDI, where one was a CMNN cause, 26 were NCDs, and three were injuries. The leading five causes for high-middle SDI decreased in age-standardised DALY rates from both 1990 to 2006 and 2006 to 2016, although this pattern did not hold for high SDI, where just the leading cause decreased over both intervals. Road injuries and falls are the only injuries that appear across all five SDI quintiles; self-harm appears in all quintiles except for low SDI. Ischaemic heart disease and cerebrovascular disease appear in the leading 13 causes across all SDI quintiles. HIV/AIDS appears in the leading ten causes for all quintiles except for high and high-middle SDI, where it did not appear in the leading 30.

### Regional and country-specific HALE and DALYs in 2016

In 2016, HALE at birth was highest in Singapore for both females (75·2 years [95% UI 71·9–78·6]) and males (72·0 years [68·8–75·1]) and lowest for females in the Central African Republic (45·6 years [42·0–49·5]) and for males in Lesotho (41·5 [39·0–44·0]; Table 2, Table 3). In 2016, HALE at birth was greater than 70 years in only 12 locations for males and was greater than this threshold in 49 locations for females. HALE at birth was less than 50 years for both sexes in three locations: the Central African Republic, Lesotho, and Afghanistan. Between 1990 and 2016, HALE at birth increased for males in 160 locations and for females in 167 locations. For males, the largest increase occurred in Ethiopia, rising from 39·3 years (37·5–41·0) in 1990 to 57·2 years (54·1–60·45) in 2016. For females, the largest increase occurred in the Maldives, rising from 53·6 (51·1–56·1) in 1990 to 70·3 (66·3–73·7) in 2016. Over the same time period, HALE at birth decreased in just two countries for females (Lesotho and South Africa) and three for males (Lesotho, South Africa, and Swaziland). In 2016, HALE at age 65 years was highest in Singapore for males (15·1 years [13·4–16·8]) and Japan for females (18·7 years [17·1–20·0]), whereas it was lowest for males in Lesotho (6·9 years [6·0–7·8]) and females in Afghanistan (7·4 years [6·5–8·3]).

**Figure 3:**
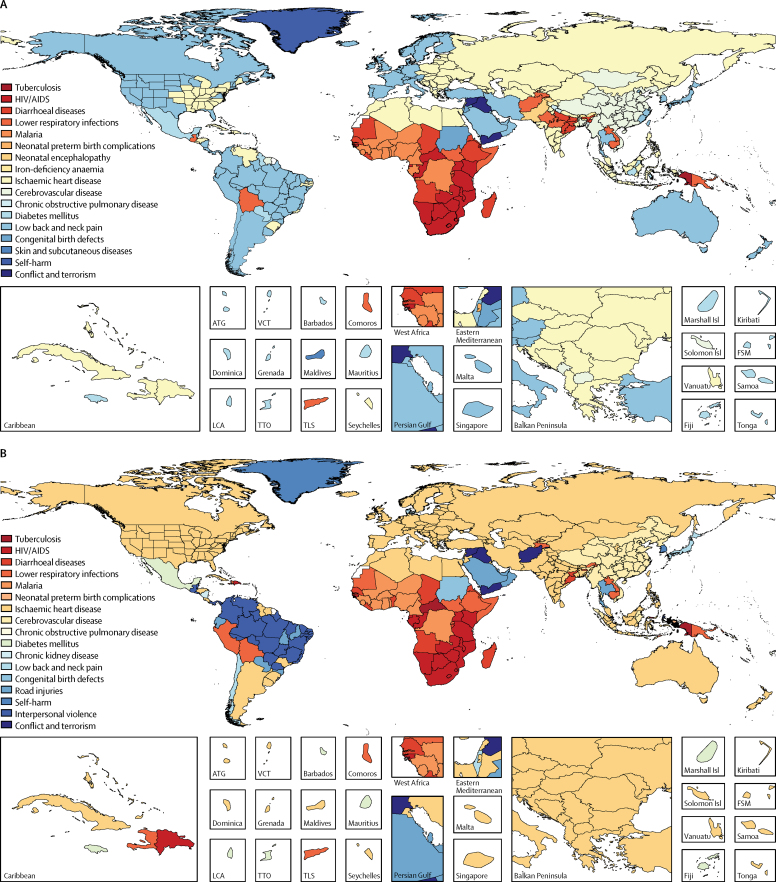
Leading Level 3 causes of all-age DALY rates by location for females (A) and males (B) in 2016 ATG=Antigua and Barbuda. DALY=disability-adjusted life-year. FSM=Federated States of Micronesia. LCA=Saint Lucia. TLS=Timor-Leste. TTO=Trinidad and Tobago. VCT=Saint Vincent and the Grenadines. VUT=Vanuatu. WSM=Samoa.

The leading Level 3 causes of DALYs in 2016 varied by country, but with regional commonalities, described on the basis of GBD regions^12^ in figure 3. Across the 46 countries within sub-Saharan Africa, the leading Level 3 cause of DALYs for females was either HIV/AIDS (17 countries), malaria (13 countries), or diarrhoeal diseases (nine countries). Cerebrovascular disease, ischaemic heart disease, and low back and neck pain were the most common leading Level 3 causes of all-age DALYs for females in all GBD regions except for Oceania, Andean Latin America and central Latin America, and all regions of sub-Saharan Africa. Among the 34 countries in the high-income super-region, low back and neck pain was the leading cause of DALYs for females in all but three countries. Exceptions within the region were Greece and the USA, for which ischaemic heart disease was the leading cause of DALYs, and Greenland, where self-harm was the highest cause of DALYs for females (and for males). For males, these regional commonalities also occurred, but with some notable differences in the leading causes by location compared with females. Leading causes of DALYs for males in sub-Saharan Africa were broadly similar to those for females: HIV/AIDS (16 countries), malaria (11 countries), or lower respiratory infections (nine countries). Cerebrovascular disease or ischaemic heart disease was the leading cause of all-age DALYs for males in almost all GBD regions except for Andean Latin America, central Latin America, tropical Latin America, high-income Asia Pacific, and all regions of sub-Saharan Africa. Exceptions within the regions included Greenland, where self-harm was the leading cause of DALYs for males in 2016, Peru, where lower respiratory infection was the leading cause, and Nicaragua, where chronic kidney disease was the leading cause. The leading cause of DALYs for both males and females in Syria and Iraq was conflict and terrorism, reflecting the ongoing conflict.

### Epidemiological transition

**Figure 4:**
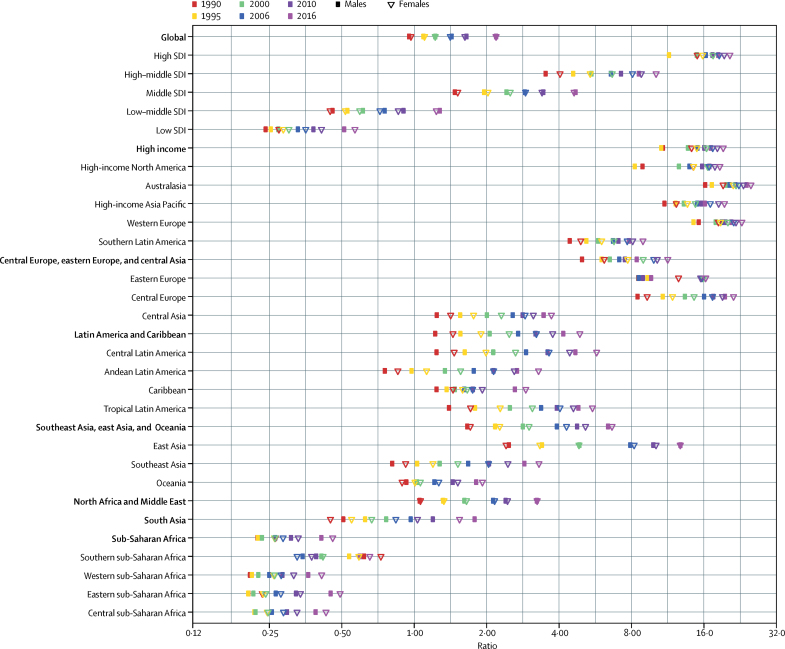
Ratios of all-age DALY counts from NCDs to those from CMNN diseases by SDI quintile, GBD super-region, and region, for males and females separately, for 1990, 1995, 2000, 2006, 2010, and 2016 Each point represents the ratio of DALYs from NCDs to those from CMNN diseases for each GBD region in a given year by sex. Ratios of more than 1·00 represent a greater number of DALYs from NCDs than from CMNN diseases. CMNN=communicable, maternal, neonatal, and nutritional. DALY=disability-adjusted life-year. GBD=Global Burden of Disease. NCDs=non-communicable diseases. SDI=Socio-demographic Index.

The shift in the global burden of DALYs from CMNN to NCDs is a clear indicator of the epidemiological transition.^21^ The ratios of DALYs from NCDs to those from CMNN diseases in 1990, 1995, 2000, 2006, 2010, and 2016 are shown by SDI and at the global, GBD regional, and GBD super-regional scales for both males and females in figure 4. In 2016, 17 (of 21) GBD regions and six (of seven) GBD super-regions had a ratio of more than 1 for either men or women, representing the shift to more DALYs from NCDs than from CMNN diseases, compared with 13 regions and five super-regions in 1990. In 2016, females had more DALYs due to NCDs relative to CMNN causes than did males, with higher ratios in 14 regions. This figure also shows how these ratios shift with increasing SDI, with 18·5 as the 2016 ratio for high SDI for men and 20·5 for women and 0·52 for men in low SDI and 0·57 for women. Several regions showed large shifts toward more NCD DALYs than CMNN DALYs, with 12 regions doubling in ratio from 1990 to 2016 for females and 11 regions doubling for males over the same time period.

**Figure 5:**
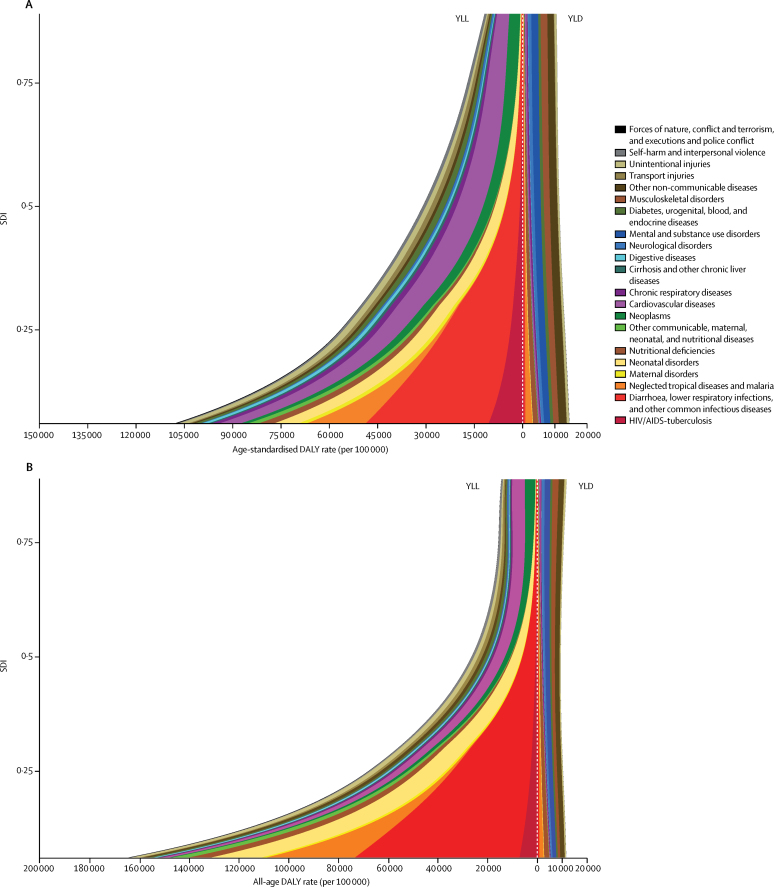
Expected relationship between age-standardised YLL and YLD rates (per 100 000 people) and SDI (A) and all-age YLL and YLD rates (per 100 000 people) and SDI (B) for 21 Level 2 causes These stacked curves represent the average relationship between SDI and YLL and YLD rates for each cause observed across all geographies over the time period 1990–2016. In each figure, the y axis goes from lowest SDI to highest SDI. The left side shows rates for YLLs on a reflected axis and the right side shows rates for YLDs; higher rates are further from the midline in each direction. Differences in patterns between (A) and (B) is the effect of shifts in population age structure in relation to SDI. DALY=disability-adjusted life-year. SDI=Socio-demographic Index. YLDs=years lived with disability. YLLs=years of life lost.

Expected age-standardised YLL rates decreased substantially with increasing SDI, most notably because of decreases in CMNN causes, such as diarrhoea, lower respiratory infections, and other common infectious diseases; NTDs and malaria; neonatal disorders; nutritional deficiencies; and HIV/AIDS and tuberculosis (figure 5A). Several causes had their largest expected age-standardised rates at intermediate levels of SDI, such as diabetes, urogenital, blood, and endocrine diseases; cardiovascular diseases; and chronic respiratory diseases. Causes that increased in expected age-standardised YLL rates with increasing SDI included neoplasms and neurological disorders. Much less variation was estimated for expected age-standardised YLD rates with SDI than for YLL rates. Although the relationship between disability and SDI was generally constant for most Level 2 causes, nutritional deficiencies, NTDs, and malaria resulted in greater than expected levels of disability at lower SDI.

Compared with YLLs and YLDs expressed as all-age rates, the consequences of differences in population age structure become apparent for a number of causes (figure 5B). Cardiovascular diseases, in particular, result in greater YLLs at higher SDI in terms of all-age rates, reflecting the older age of populations at higher levels of SDI. By contrast, the pattern observed when standardising for age structure—YLLs accruing at intermediate SDI—highlights health loss from diseases that typically manifest at older ages undistorted by the lower overall age structure of the populations. The expected all-age YLD rates generally increased with rising SDI, particularly for mental and substance use disorders and musculoskeletal disorders, although variation with SDI remained substantially lower than for YLLs.

**Figure 6:**
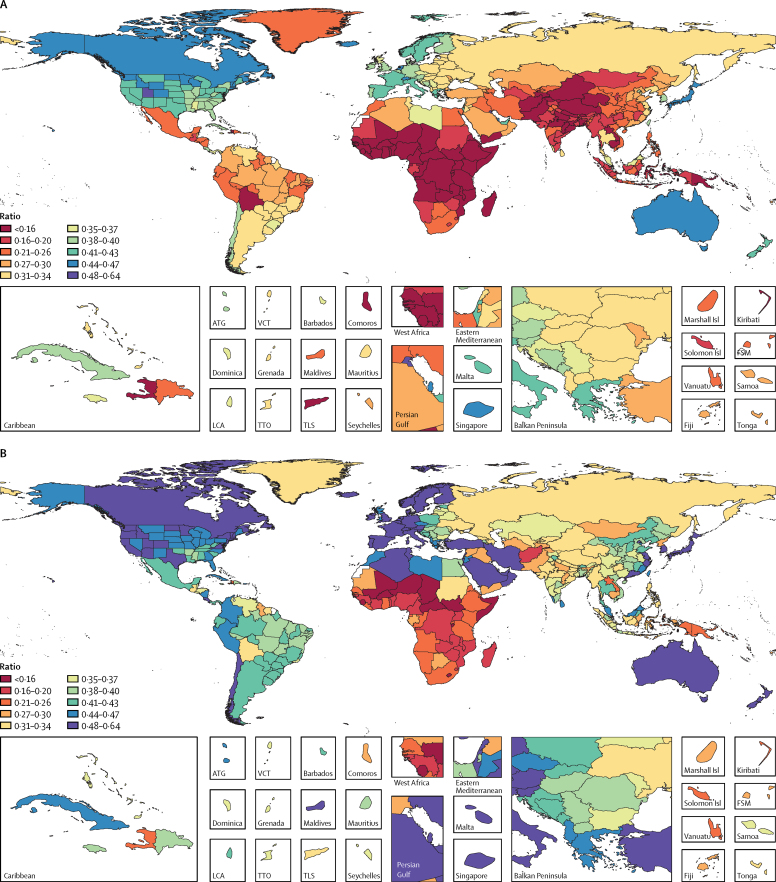
Proportion of all-age DALY counts due to years lived with disability for all ages and both sexes combined for 1990 (A) and 2016 (B) Locations are colour-coded in terms of the magnitude of the ratio. ATG=Antigua and Barbuda. DALY=disability-adjusted life-year. FSM=Federated States of Micronesia. LCA=Saint Lucia. TLS=Timor-Leste. TTO=Trinidad and Tobago. VCT=Saint Vincent and the Grenadines.

The proportion of DALYs due to YLDs versus YLLs has shifted distinctly since 1990, and these changes are shown in figure 6. More all-age DALYs as a result of YLDs rather than YLLs indicates a reduction in premature death, with individuals living longer with disability than previously. Globally, 95·9% of countries increased in the proportion of all-age DALYs due to YLDs from 1990 to 2016. In 2016, Kuwait (0·64), Qatar (0·63), and Bahrain (0·60) had the highest ratios of DALYs due to YLDs, whereas Chad (0·14), Niger (0·14), and the Central African Republic (0·13) had the lowest. This finding is a change from 1990, when the highest three proportions were for Andorra (0·49), Kuwait (0·48), and Iceland (0·47), and the lowest three were for Burundi (0·08), Malawi (0·08), and Niger (0·06).

### Observed versus expected total and cause-specific burden

**Figure 7:**
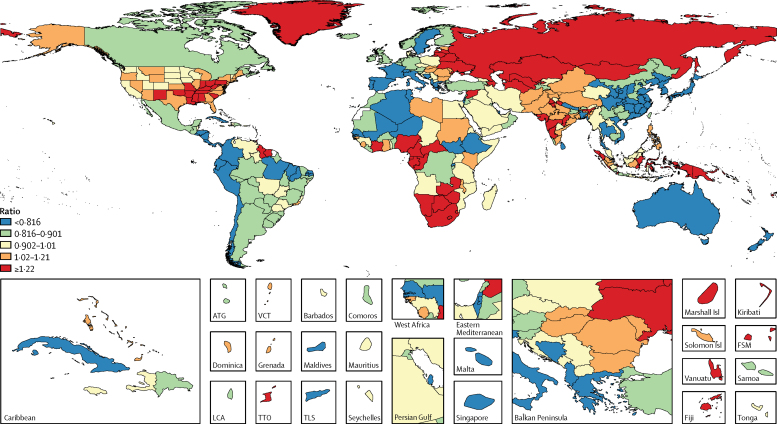
Ratio of observed to expected age-standardised DALY rates (per 100 000) on the basis of SDI alone for both sexes combined in 2016 Ratios are colour-coded in terms of the magnitude of differences between observed and expected age-standardised DALY rates. Blues indicate much lower observed DALY levels than expected on the basis of SDI, whereas reds reflect observed DALYs that far exceed expected levels given SDI. ATG=Antigua and Barbuda. DALY=disability-adjusted life-year. FSM=Federated States of Micronesia. LCA=Saint Lucia. SDI=Socio-demographic Index. TLS=Timor-Leste. TTO=Trinidad and Tobago. VCT=Saint Vincent and the Grenadines.

As shown in figure 7, 83 countries had greater than expected age-standardised DALY rates on the basis of SDI in 2016 (a ratio of observed to expected age-standardised DALY rate of greater than 1), generally within central Europe, eastern Europe, and central Asia (20 of 29 countries) or in the super-region of sub-Saharan Africa (24 of 46 countries). Among the 34 locations in the five regions within the high-income GBD super-region, three had greater than expected DALYs relative to their SDI. The five locations with the lowest level of age-standardised DALYs relative to the level expected on the basis of SDI were Nicaragua, Costa Rica, the Maldives, Peru, and Israel. Countries within western Europe (eg, France, Norway, and Iceland), western sub-Saharan Africa (eg, The Gambia, Senegal, and Liberia), eastern sub-Saharan Africa (eg, Ethiopia, Rwanda, and Burundi), east Asia (eg, China), and a subset of countries in south and southeast Asia (eg, Bhutan and Bangladesh) all had ratios of observed to expected DALY rates of less than 1 in 2016. The five locations with the highest age-standardised DALY rates relative to the rates expected on the basis of SDI were Lesotho, Swaziland, South Africa, Fiji, and Botswana, with Lesotho's ratio of observed to expected measured at 2·43 in 2016.

**Figure 8:**
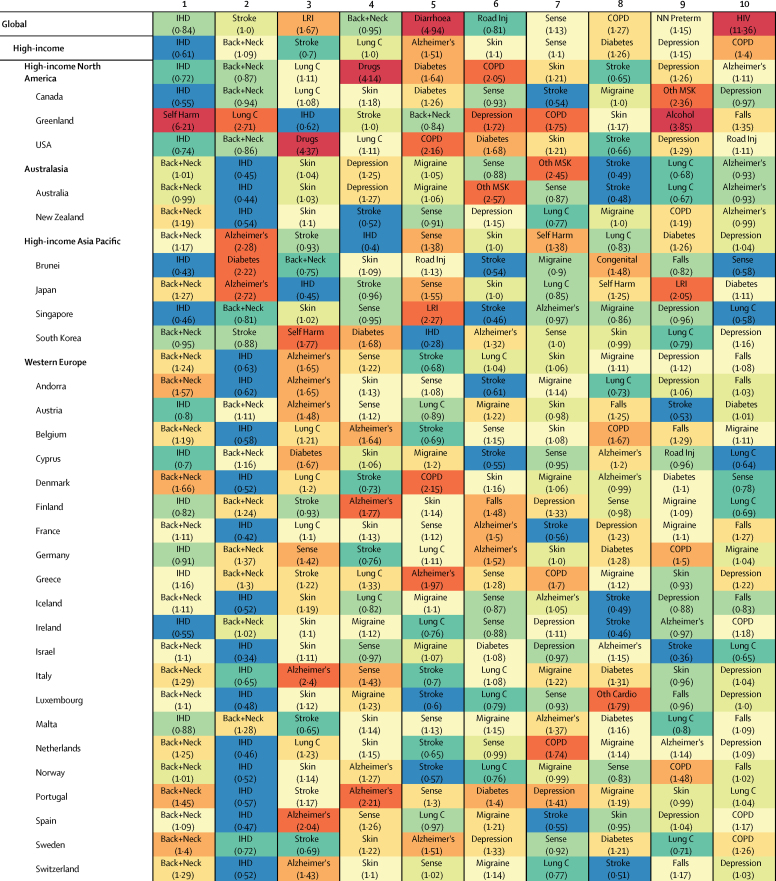

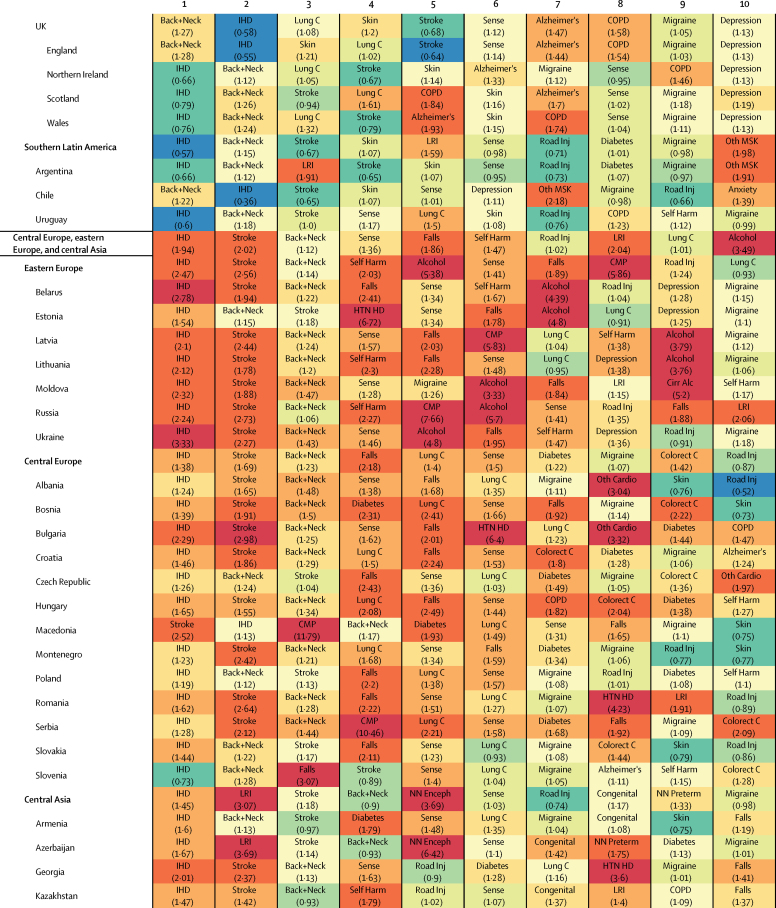

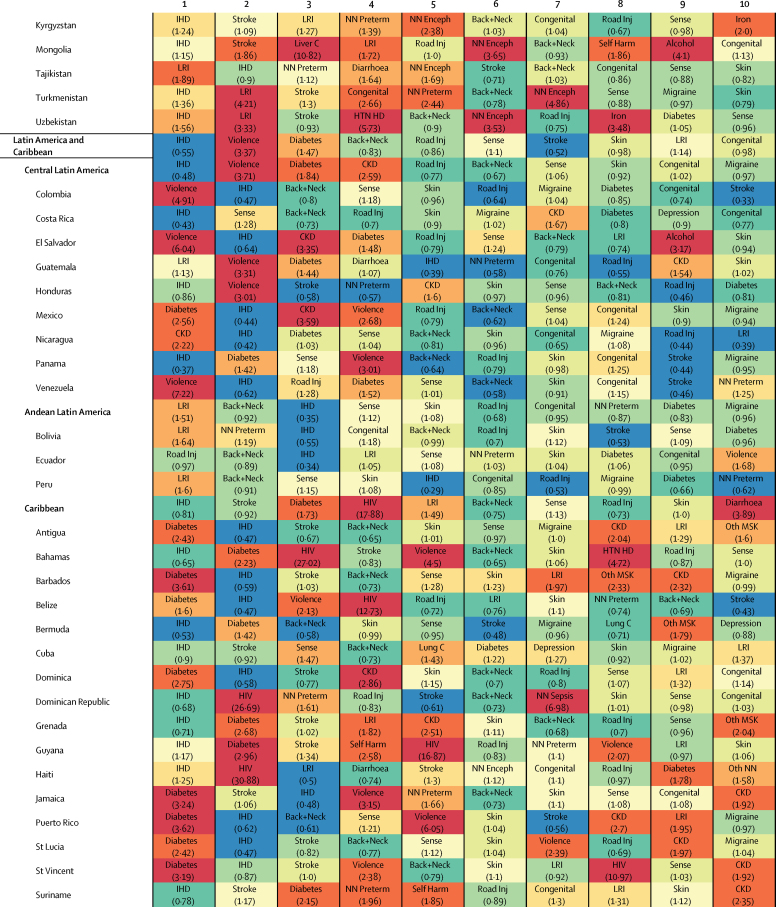

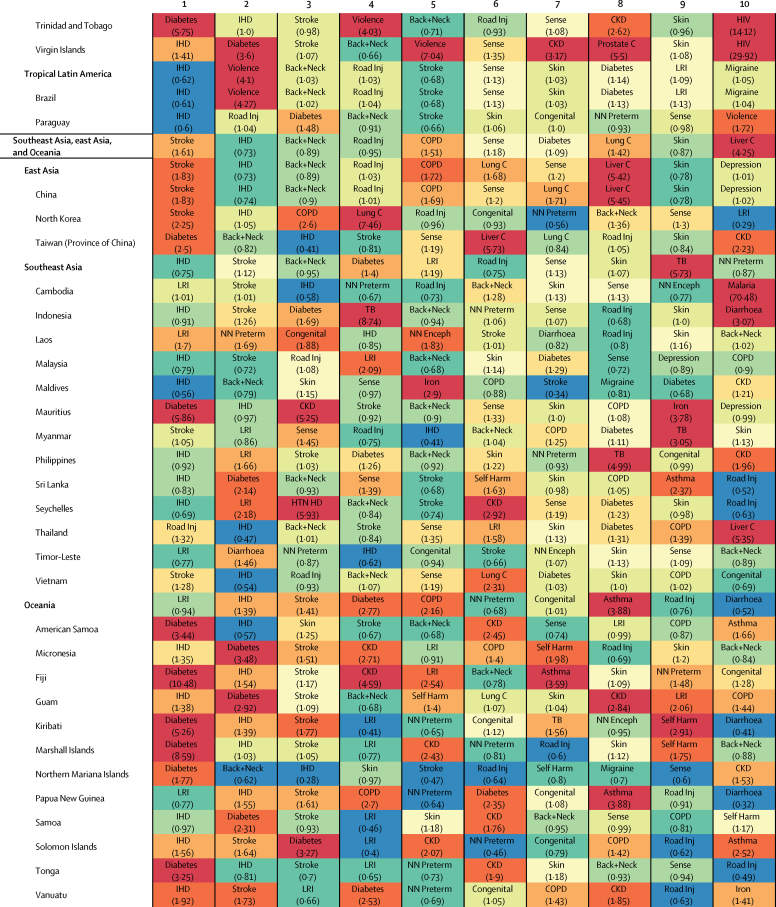

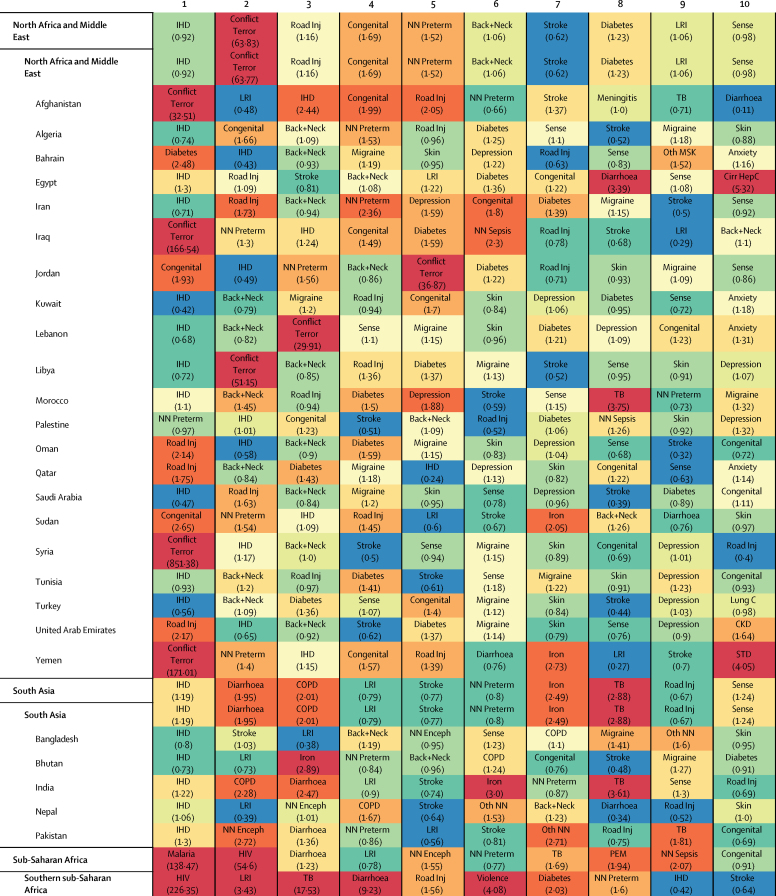

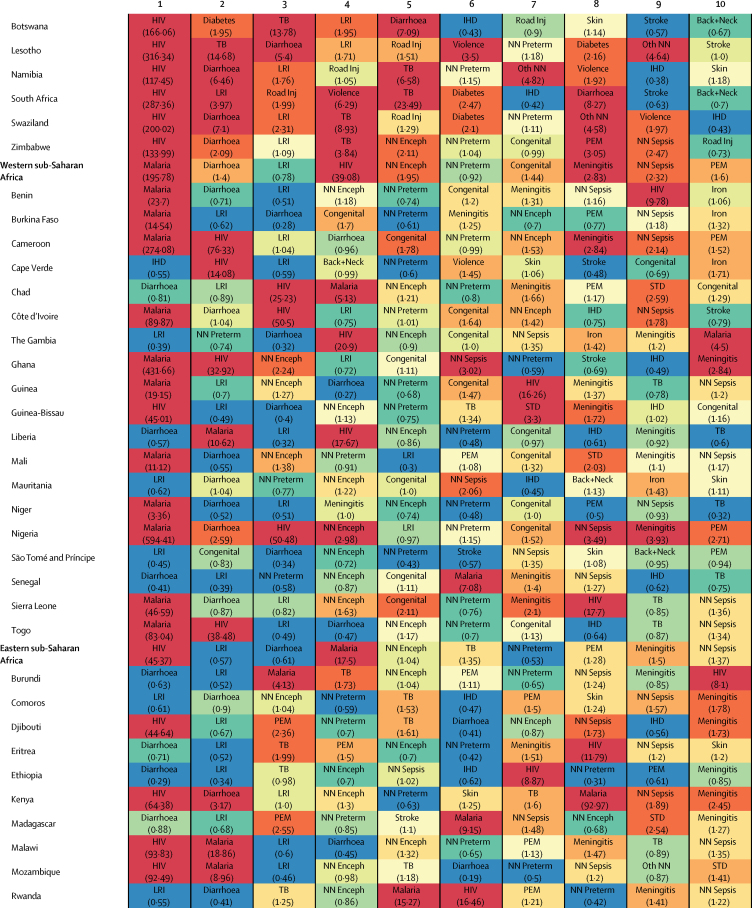

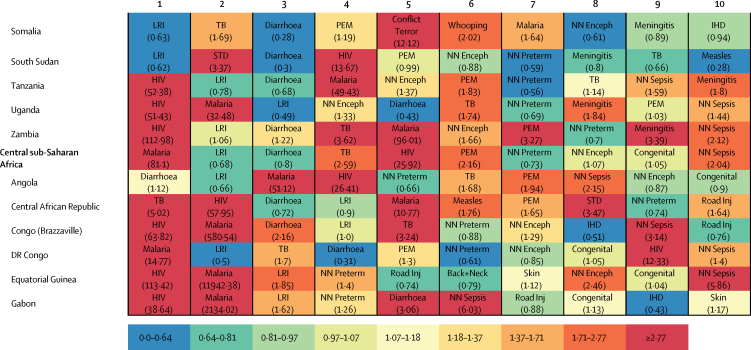
Leading ten causes of all-age DALYs with the ratio of observed to expected DALYs on the basis of Socio-demographic Index in 2016, by location The ratio of observed to expected DALYs on the basis of Socio-demographic Index is provided in brackets for each cause and cells are colour-coded by ratio ranges (calculated to place a roughly equal number of cells into each bin). Shades of blue represent much lower observed DALY levels than expected on the basis of Socio-demographic Index, whereas red shows observed DALYs that exceed expected levels. Alcohol=alcohol use disorders. Alzheimer's=Alzheimer's disease and other dementias. Anxiety=anxiety disorders. Back+Neck=low back and neck pain. Cirr alc=cirrhosis due to alcohol use. Cirr HepC=cirrhosis due to hepatitis C. CKD=chronic kidney disease. CMP=cardiomyopathy and myocarditis. Colorect C=colon and rectum cancer. Conflict Terror=conflict and terrorism. Congenital=congenital anomalies. COPD=chronic obstructive pulmonary disease. Depression=depressive disorders. Diabetes=diabetes mellitus. Diarrhoea=diarrhoeal diseases. DR Congo=Democratic Republic of the Congo. Drugs=drug use disorders. HIV=HIV/AIDS. HTN HD=hypertensive heart disease. IHD=ischaemic heart disease. Iron=iron-deficiency anaemia. Liver C=liver cancer. LRI=lower respiratory infections. Lung C=lung, bronchus, and trachea cancers. NN Enceph=neonatal encephalopathy due to birth asphyxia and trauma. NN Preterm=neonatal preterm birth complications. NN Sepsis=neonatal sepsis and other neonatal infections. Oth Cardio=other cardiovascular and circulatory diseases. Oth MSK=other musculoskeletal disorders. Oth NN=Other neonatal disorders. PEM=protein-energy malnutrition. Prostate C=prostate cancer. Road Inj=road injuries. Sense=sense organ diseases. Skin=skin and subcutaneous diseases. STD=sexually transmitted diseases excluding HIV. Stomach C=stomach cancer. Stroke=Cerebrovascular disease. TB=tuberculosis. Violence=interpersonal violence.

Worldwide, ischaemic heart disease and stroke remained the two leading causes of DALYs, and expected levels were close to those observed at a global scale, with ratios of 0·84 for ischaemic heart disease and 1·0 for stroke (figure 8). The ratio of observed to expected DALYs ranged from 0·24 in Qatar to 3·33 in Ukraine for ischaemic heart disease and from 0·32 in Oman to 2·98 in Bulgaria for stroke. This ratio was less than 1 for 114 of 167 global locations for ischaemic heart disease and 91 of 145 for stroke. Low back and neck pain resulted in fewer than expected DALYs globally, with a ratio of 0·95 globally and 74 locations showing a ratio of less than 1. Of the ten leading causes for each location, observed levels of DALYs were frequently greater than expected on the basis of SDI for causes such as diabetes mellitus (ratio of observed to expected DALYs of greater than 1 in 95 global locations), sense organ diseases (79 locations), and skin and subcutaneous diseases (72 locations).

Causes that resulted in greater than expected DALYs for both sexes combined showed strong regional patterns. In the GBD high-income super-region, of the leading ten causes of DALYs, COPD had greater than expected DALYs in 14 of 34 locations and less than expected in none. Observed DALYs were frequently greater than expected for low back and neck pain (27 locations), although the ratio did not exceed 1·66 for any single location. Although drug use disorders were only within the leading ten causes for one location in the high-income super-region, the ratio of observed to expected DALYs in the USA was 4·37. In addition to being the most common leading causes of DALYs, ischaemic heart disease and stroke were sources of more DALYs than expected for most locations in the central Europe, eastern Europe, and central Asia super-region. Here, the highest ratio of observed to expected DALYs was 3·33 for ischaemic heart disease and 2·98 for stroke. Mental and substance use disorders were also notable in the region for the high levels of observed DALYs relative to those expected, particularly in eastern Europe, where the ratio for the Level 4 cause of alcohol use disorders was 5·38 and ranged from 3·33 in Moldova to 5·7 in Russia. For locations in the super-region of Latin America and Caribbean, the burden from interpersonal violence was greater than expected, with a ratio of 3·37 for the super-region and ranging from 1·68 in Ecuador to 7·22 in Venezuela. Diabetes was also a source of greater than expected burden in this super-region, with DALYs exceeding expected levels in 19 of 37 locations. Additionally, diabetes contributed to a higher burden of DALYs in many locations in the super-region of southeast Asia, east Asia, and Oceania, with ratios of observed to expected levels ranging from 0·68 in the Maldives to 10·48 in Fiji.

In south Asia, neonatal encephalopathy due to birth asphyxia and trauma caused greater than expected DALYs for two countries, with ratios of 1·01 in Nepal and 2·72 in Pakistan. Ongoing conflicts in north Africa and the Middle East were reflected in higher than expected DALYs in seven of the 21 locations in the region. Patterns of observed DALYs compared with those expected in sub-Saharan Africa were dominated by the ongoing effect of HIV/AIDS, particularly in southern sub-Saharan Africa. Observed levels of DALYs exceeded those expected in 37 of 46 locations, ranging from a ratio of 8·10 in Burundi to 316·34 in Lesotho.

The five locations with the largest magnitude of improvement—a decrease in the difference between observed and expected all-cause age-standardised DALY rates from 1990 to 2016—included Greenland, the Maldives, Bermuda, Ethiopia, and Liberia. The five locations that showed the smallest decrease in the difference between observed and expected DALY rates over the period 1990–2016 were Lesotho, Swaziland, Syria, South Africa, and Botswana.

## Discussion

### General findings

Although the world has become healthier since 1990, this progress has been highly heterogeneous. From 1990 to 2016, global HALE at birth increased. The total number of years of functional health lost (life expectancy minus HALE) increased from 1990 to 2016, indicating an absolute expansion of morbidity. YLL rates have fallen more rapidly than YLD rates so that non-fatal health loss as a proportion of DALYs has grown from 1990 to 2016. This progress has been driven by marked reductions in DALYs due to CMNN causes and, to a lesser extent, by declines in age-standardised NCD and injury DALYs. Population growth in the past 27 years (from 5·27 billion to 7·39 billion) has resulted in a significant increase in total DALYs due to NCDs, coupled with a high total DALY burden in 2016, thus leading to an overall increasing toll on health systems.^22^, ^23^ GBD 2016 continues to unequivocally show an absolute expansion of morbidity^4^—that is, as life expectancy increases across a population, the absolute amount of functional health loss increases—but GBD 2016 also shows a relative compression of morbidity—that is, the proportion of total life spent ill per person decreases with increasing SDI.^24^ Substantial opportunities remain for innovation in human resources for health, development assistance for health, and targeted health policies to alleviate causes of old-age disability and transform the experience of later life for billions of people.^25^

From 1990 to 2016, life expectancy, HALE, and years lived with functional health loss all increased as populations moved through the SDI quintiles. These quintiles reflect in a geographical cross section of the world what is seen through time during the epidemiological transition. The rapid decrease in burden from CMNN diseases from low to high SDI quintile was most substantial. NCDs and injuries were also seen to decline, but at much more modest rates. Declines in fertility, increases in education, and increases in income per person (the triad of inputs into the SDI composite), were tightly coupled with population-based HALE outcomes.^26^ This finding suggests that the wide agendas of these three social determinants of health have roles within discussions of overall health system strengthening. Further dissection of the results clearly shows that a substantial impact has been made on the global burden of YLLs. A much more modest effect, however, has been made on the burden of large causes of YLDs at all levels of SDI. The contrast in this ratio is marked and illustrates another striking view of the epidemiological transition when looking at the proportion of DALYs due to YLDs globally. Although continued progress on reduction of YLLs prematurely is important, how to ameliorate the primary causes and underlying risks of YLDs globally should be reflected on (low back and neck pain, sense organ diseases, and skin diseases) to decrease the time that ageing populations live with functional health loss across the SDI spectrum. This persisting burden of morbidity highlights the importance, both in terms of relief of suffering and prevention of health-care costs, of placement of emphasis on prevention of disabling illness and mitigation of the ill effects of the disabling conditions that do occur.

### Cross-cutting themes

The concept of the grand convergence—that levels of under-5 and maternal mortality, as well as selected infectious diseases, would converge across SDI quintiles within a generation—was promoted in the *Lancet* Commission on investing in health^27^ and discussed in the GBD 2015 results.^4^ The conclusion that DALY convergence is likely to be accelerated by escalating increases in SDI remains valid. We have looked a step further in GBD 2016 and highlighted countries that have most improved and showed the largest declines in their DALY performance relative to that expected on the basis of their SDI.

The pace and efficiency of a country's transition through the SDI quintiles and the concurrent improvements in population summary health metrics is by no means inevitable, however, and analysis derived from the ratio of observed to expected DALYs on the basis of SDI has been informative. The exemplars were Nicaragua, Costa Rica, the Maldives, Peru, and Israel, which had the lowest levels of age-standardised DALYs relative to the levels expected on the basis of SDI in 2016. The appendix (pp 63–66) contains a full list of exemplars. These countries considerably outperformed health expectations relative to their development status. Conversely, Lesotho, Swaziland, South Africa, Fiji, and Botswana had the highest levels of age-standardised DALYs relative to those expected, reflecting a need for additional attention and examination of the reasons for these discrepancies. Lesotho had the highest ratio of age-standardised DALYs relative to that expected, which indicates poor performance in terms of health outcomes for mortality and morbidity relative to their SDI status.

The reasons for the success in health outcomes of the five exemplars merit in-depth analyses. Conversely, with the exception of Fiji (which is heavily impacted by the high levels of diabetes seen throughout Oceania), the poor performers in 2016 were clearly those that were most devastated by the continuing HIV/AIDS epidemic and, although progress has been made in South Africa, Swaziland, Zimbabwe, and Lesotho are still disproportionately affected. Many different locations within the top ten exemplars and ten poor performers would make excellent case studies to assess the reasons for their relative rankings, particularly given the different health challenges and successes for each nation.

By highlighting exemplars and poor performers across the SDI quintiles, we have seen that the underlying influences that have contributed to the relative successes and failures of nations are substantially heterogeneous. A clear corollary is that the policy implications for reform will not be the same between quintiles. Nation-by-nation complexity in how countries progress through the SDI continuum and how population health summaries track is clearly too voluminous to document in this study by nation, but we hope that the results of this study will spawn a plethora of derivative and policy-relevant research.

We have highlighted key exemplar nations and those whose progress could be improved, but there are clearly more general lessons that can be learned from the epidemiological transition across all causes and locations. The collection of London Declaration NTDs, along with malaria^28^ and HIV, all show how effective domestic and international collaborations in fighting of specific infectious diseases can be. Whether or not these collaborations provide evidence of health-care improvements in one area, whether or not the benefits cascade, and whether or not non-health-specific interventions, such as poverty alleviation, education,^29^ and family planning, are having synergistic effects are hard to differentiate,^28^, ^30^ but a host of online tools exist that enable national experts to examine performance of and changes in the leading causes of disease burden, compare them with peers, and objectively assess what can be improved.

Since the concept of the DALY was introduced two decades ago,^31^ it has become a key metric for monitoring of population health and prioritisation within health sectors.^32^, ^33^, ^34^, ^35^, ^36^, ^37^, ^38^, ^39^ The following are examples of policy makers, funders, or foundations that use DALYs in decision making: WHO, the World Bank, the National Institutes of Health, the US Centers for Disease Control and Prevention, the Bill & Melinda Gates Foundation, Gavi, the US President's Emergency Plan for AIDS Relief, the Global Fund, and the Wellcome Trust, as well as the Chinese Politburo, the National Institute for Health and Care Excellence, Indonesia Bappenas, and Public Health England at the national level.

### Comparison of GBD 2016 with other global estimates and GBD 2015

The GBD study is the only source of comprehensive quantification of population health summary measures, including YLLs, YLDs, DALYs, and HALE. Specific efforts that are relevant to policy makers are being made to estimate burden within other organisations. Since GBD 2015, most organisations that we assessed have not produced updated estimates for DALYs or HALE. The exception to this is WHO, which has released updated Global Health Estimates for DALYs and HALE for 183 countries from 2000 to 2015.^40^ These estimates draw heavily on the GBD 2015 results, with revisions to the all-cause mortality envelope^40^, ^41^ as detailed in the GBD 2016 cause-specific mortality publication^10^ and to selected cause-specific disability weights and severity distributions for YLDs.^42^ WHO-adjusted disability weights for various causes are further detailed in the WHO Global Health Estimates Technical Paper.^41^

### Disease-specific considerations

The SDI transition was prominent for many CMNN conditions, especially diarrhoea, lower respiratory infection, HIV after 2005 (the year of the global peak), maternal disorders, and vaccine-preventable diseases like measles, tetanus, diphtheria, and pertussis. Focused programmes with community-wide targeting of health interventions—such as access to antenatal care,^43^, ^44^ vaccination, malaria vector control (insecticide-treated bednets and indoor residual spraying),^45^ and artemisinin combination therapies,^46^ water and sanitation efforts,^47^ and ART and prevention of mother-to-child transmission of HIV^30^, ^48^, ^49^—are potentially responsible for DALY reductions in these conditions.^50^ Further scale-up and maintenance of these interventions should continue, including a transition from non-governmental organisations to local ownership and financing. Corresponding improvements in health system functions related to survival of mothers and their children have not occurred,^51^ shown by the comparatively slow improvement in DALYs due to maternal and neonatal disorders compared with other CMNN causes and the growing burden of congenital birth defects, haemoglobinopathies and haemolytic anaemias, and sudden infant death syndrome, especially in the under-5 age group. Early detection of complications of pregnancy like micronutrient deficiencies, hypertensive disorders of pregnancy, pregnancy-transmitted and sexually transmitted infections, and congenital birth defects can help identify the pregnant women and newborns at highest risk, help ensure that appropriate curative treatments are administered, and put the females and newborns in a position to receive timely perinatal care when it is needed. Newborn screening for congenital birth defects and haemoglobinopathies can, at the very least, identify children with these conditions and facilitate maternal education about the importance of presentation for care early when a mother's child gets sick. Delays in seeking care can have deadly consequences, particularly for children with acute conditions. Education of mothers and fathers, especially first-time parents, about how to best care for their babies can help reduce avoidable injuries and deaths. At the same time, stark increases in neonatal sepsis DALYs, in which nosocomial infections of the newborn are included, highlight the crucial importance of being vigilant in infection control within hospitals and clinics.

This iteration of GBD estimated diseases and injuries for 5 year age groups older than 80 years for the first time: 80–84 years, 85–89 years, 90–94 years, and 95 years and older. This addition has provided a clearer picture than from previous GBD publications of the burden of disease in ageing populations, who are disproportionately affected by dementias and other NCDs compared with younger age groups. Ageing populations have increased substantially in size since 1990. As a result of this population shift and the epidemiological transition (which increases the proportion of burden of disease due to NCDs), age groups older than 80 years had increases in all-age DALYs between 1990 and 2016, with increases across all SDI quintiles. The proportion due to NCDs also increased. Therefore, research into these age groups and the diseases that continue to affect them over time should be prioritised.

### Tuberculosis

We made important changes to the modelling strategy for tuberculosis in GBD 2016. For fatal tuberculosis, we first modelled the prevalence of active disease and latent infection, which we then used as covariates for the Cause of Death Ensemble model. For non-fatal tuberculosis, we strengthened our statistical triangulation approach, which enforces consistency between data for different parameters by modelling tuberculosis incidence, prevalence, and mortality among those with latent infection. Application of MIRs estimated on the basis of SDI to better reflect incidence in low-income and middle-income countries has also enhanced consistency between fatal and non-fatal estimates of tuberculosis. All of these changes have resulted in global tuberculosis DALYs that are 15% higher than in GBD 2015 for the year 2010, with prominent increases occurring in several African countries, including Uganda, the Central African Republic, and Zambia.

### HIV/AIDS

A major HIV methods change for GBD 2016 was the distribution of ART coverage by age, sex, and CD4-positive cell count. We used two AIDS Indicator Surveys^52^, ^53^ to predict the age-sex-CD4 cell distribution of ART coverage and applied the distributions to the input counts of people receiving ART in our HIV estimation model. This method shifted the coverage distribution to groups with higher CD4 cell counts, as was seen in the data. All of these changes have resulted in higher global HIV/AIDS DALYs than in GBD 2015 for the year 2010, with the most prominent increases occurring in several African countries, including South Africa, Botswana, Lesotho, and Swaziland. We have also systematically updated other key input parameters to the HIV/AIDS estimation process, such as the on-ART mortality rate and other demographic inputs, including HIV-free mortality.

### Lower respiratory infection and diarrhoea

Total lower respiratory infection (increased by 0·701%) and diarrhoeal disease (increased by 15·5%) DALYs increased compared with GBD 2015 estimates for the year 2010. Like most CMNN causes, the lower respiratory infection and diarrhoeal disease DALY burden is due primarily to YLLs; more than 90% of global DALYs are from these causes. Several changes have been made to the under-5 mortality models that have large impacts on the DALY totals for these causes. We added several new model covariates on the basis of risk factors associated with lower respiratory infections and diarrhoeal diseases, including childhood stunting and suboptimal breastfeeding. Additionally, because of India's large population size and disease burden, its Sample Registration System data changed the magnitude of YLLs. Inclusion of diarrhoeal diseases as a fatal discontinuity in YLL estimation also affected the overall estimation for GBD 2016. Overall, the YLLs due to lower respiratory infections decreased slightly (by 0·626%) for the year 2010 from GBD 2015 to GBD 2016 and those due to diarrhoeal diseases increased (by 14·3%). We believe that these modelling changes improved under-5 mortality estimates for lower respiratory infections and diarrhoea. Although responsible for a smaller contribution to DALYs than cause of death models, the non-fatal models of lower respiratory infections and diarrhoea included new data sources and processing. An example is that we now account for seasonality from the population-representative surveys that provide most prevalence data from sub-Saharan Africa and south Asia in the lower respiratory infection and diarrhoeal disease models. Another major update is a change in the estimated mean duration of illness on the basis of a set of updated systematic reviews. The duration of diarrhoea remained nearly the same as in GBD 2015, but that of lower respiratory infection decreased by about 20% compared with GBD 2015.

### Malaria

Refinements to the methodological approach and addition of substantially more data than in GBD 2015 have led to changes in estimates of both malaria mortality and morbidity estimates in the GBD 2016 iteration, with resulting changes in DALYs. Globally, predicted trends in malaria were similar to GBD 2015, rising to a peak in 2005 before steadily declining. Overall malaria DALYs are lower in GBD 2016 than in GBD 2015, reflecting mainly lower estimates outside of Africa and particularly for India. Outside of Africa, and for lower-burden countries within Africa, estimates were informed for the first time by extensive subnational case-reporting data from routine surveillance systems. These were subsequently adjusted to account for under-reporting, misdiagnosis, and incompleteness, and then entered into a spatiotemporal geostatistical model to infer continuous surfaces of incidence rate before reaggregating them to national and subnational totals. This approach led to notable reductions in estimated cases, YLDs, and DALYs in India, Myanmar, Indonesia, and Pakistan. In high-burden countries in sub-Saharan Africa, where the methods remained similar to GBD 2015, changes were relatively modest and reflected the inclusion of newly available cross-sectional parasite rate surveys or updates to data for malaria intervention coverage in recent years.

### London Declaration diseases

The London Declaration was established in 2012 as a partnership of pharmaceutical companies, private foundations, and global health organisations, committed to providing resources and expertise for controlling, eliminating, or eradicating ten NTDs: human African trypanosomiasis, Chagas disease, Guinea worm disease, leprosy, lymphatic filariasis, onchocerciasis, schistosomiasis, soil-transmitted helminths, blinding trachoma, and visceral leishmaniasis.^54^ With this year's addition of Guinea worm disease to the GBD cause list, we now estimate DALYs for all ten of the London Declaration diseases and GBD estimates can, therefore, offer insight into progress. We estimate that total DALYs from the London Declaration NTDs have declined by 21·1% from 11·4 million (95% UI 6·8 million to 18·5 million) DALYs in 2010 before the London Declaration in 2012 to 9·0 million (5·3 million to 14·5 million) in 2016. Moreover, we find that DALY rates have declined for all ten of the London Declaration NTDs between 1990 and 2016, with the largest declines occurring for Guinea worm disease, reflecting the particular effectiveness of the Guinea worm eradication initiative,^55^ and for human African trypanosomiasis.^56^

### Zika virus disease

The appearance of Zika virus cases in the Americas in 2015, along with the reported associations between Zika virus infection and microcephaly and Guillain-Barré syndrome, led WHO to declare Zika virus a Public Health Emergency of International Concern in February 2016.^57^, ^58^, ^59^ We estimated Zika virus disease for GBD 2016 in response to this heightened global concern and interest. We estimated four non-fatal outcomes of Zika virus infection: asymptomatic infection, symptomatic infection, Guillain-Barré syndrome caused by Zika virus infection, and congenital Zika syndrome. Despite the high incidence observed in the Americas in 2016 and understandable public concern, especially surrounding congenital Zika syndrome, we estimate that less than 0·01% of Zika virus infections result in Guillain-Barré syndrome, congenital Zika syndrome, or death. We estimate that 7·60 million (95% UI 5·70 million to 10·7 million) infections occurred in 2016, resulting in 2·70 million symptomatic Zika virus cases, 1880 cases of Zika-attributable Guillain-Barré syndrome, and 2400 congenital Zika syndrome births.^8^ Our estimate for congenital Zika syndrome is in line with official reports from the Pan American Health Organization of circa 2500 congenital Zika syndrome births.^60^ However, those born with congenital Zika syndrome will have future disability.

### Injuries

Global DALYs due to injuries generally contributed to a lower proportion of total burden than did those due to CMNN diseases and NCDs, and remained relatively stable as a proportion of total DALYs over time. Examination of these trends by SDI quintile, region, and cause of injury reveals much more variable patterns than the global pattern, which are potentially related to a confluence of demographic changes, policy adoption and enforcement, access to high-quality trauma care, and political stability. Among high-SDI and high-middle-SDI countries, all-age DALYs due to road injuries have substantially declined since 1990, while DALYs due to road injuries in low-middle-SDI and low-SDI countries have risen, a trend that is particularly evident in recent years. By contrast, DALYs from drowning markedly declined among middle-SDI and low-middle-SDI countries. Disease burden from falls rose across the development spectrum, a trend primarily driven by population growth and ageing. Finally, the consequences of ongoing conflict, particularly in north Africa and the Middle East, and interpersonal violence, particularly in Latin America, on population health cannot be overlooked.^61^ Fatalities due to such conflict and violence have resulted in stagnated or decreasing life expectancy in many of these countries,^7^, ^62^ and for those who survive, the long-term effects of such injuries could easily result in impaired movement and functioning, heightened risk of other disorders (eg, musculoskeletal conditions), and mental health challenges for an extended period after the end of the conflict.

### Cerebrovascular disease

For GBD 2016, we modelled each stroke subtype independently to produce more reliable ratios of ischaemic to haemorrhagic stroke than for GBD 2015 and better match our independent estimation of subtype-specific stroke mortality. We avoided undercounting of stroke by reclassifying hospital admissions and deaths ascribed to unspecified stroke. Despite this method, haemorrhagic stroke remains a heterogeneous category that includes neonatal intraventricular haemorrhage and all other non-traumatic intracranial bleeding. These deaths in children younger than 5 years result in many more YLLs, and therefore many more DALYs, than from ischaemic stroke. Consistent with population-based and multinational studies of stroke subtype, GBD estimates higher incidence but much lower case fatality and YLLs due to ischaemic stroke among adults than due to haemorrhagic stroke.^63^ This pattern appears true even for locations where this pattern was not previously thought to be the case, such as in China.^64^ Future estimates can be improved by production of separate estimates of non-traumatic subarachnoid haemorrhage and paediatric stroke.

### Mental and substance use disorders

Throughout multiple iterations of GBD, mental and substance use disorders have consistently been shown as the leading causes of YLDs worldwide, with burden present in both sexes across the lifespan. They are also strongly associated with premature mortality, although this association is not reflected in GBD YLL estimates for mental disorders as they are rarely coded as the direct cause of death. Nevertheless, they contribute a substantial number of DALYs and this large contribution to burden has remained constant across time in all countries, including those with high or substantially improving SDI. Treatment rates remain very low^65^, ^66^, ^67^ and, even in high-income countries where treatment coverage has increased, the prevalence of the most common disorders has not changed.^68^ To reduce the burden of these disorders, improved treatment coverage needs to include a focus on the quality of the intervention delivered. Additionally, identification and quantification of modifiable risk factors for mental and substance use disorders are vital for development of effective prevention strategies and are an area noted for expansion in future iterations of GBD.

### Diabetes

We made several important improvements to the process of estimation of diabetes prevalence, including use of more data sources than GBD 2015 and development of a novel approach to standardise the definition of diabetes across different sources. In our assessment of diagnostic criteria for diabetes across different surveys, we identified more than 50 different definitions for diabetes based on various biomarkers (eg, fasting plasma glucose concentration, oral glucose tolerance test result, and glycated haemoglobin A1c concentration) and different levels of each biomarker. To standardise the definition of diabetes, we mostly focused on the surveys that had included fasting plasma glucose concentration as a diagnostic criterion and developed an ensemble model to characterise the distribution of fasting plasma glucose concentration at the population level in each age and sex group. Then, we used the fasting plasma glucose concentration distribution to convert various definitions of diabetes into the standard case definition. Using the ensemble model, we also estimated the prevalence of diabetes on the basis of the mean fasting plasma glucose concentration in places for which we only had data for mean fasting plasma glucose concentration. These changes allowed us to be more consistent than in GBD 2015 in our estimation of the prevalence of diabetes across countries and over time. As a result, our estimates of the prevalence of diabetes globally and in most regions are slightly lower than those reported in GBD 2015. The strong relationship between SDI and diabetes remained, mirroring global rises in overweight and obesity.^69^

### Cancer

DALYs for cancer have changed compared with GBD 2015; this change is predominantly due to lower YLD estimates for GBD 2016 than for GBD 2015. These improvements stem from adjustments made in the modelling of MIRs to better reflect differences in MIR based on SDI in data-sparse locations than in GBD 2015. In addition to stricter inclusion criteria for data used in the MIR modelling than in GBD 2015, we changed the modelling approach and used the most parsimonious model with just SDI as a predictor of MIR.^8^ MIRs are used to estimate cancer incidence and prevalence from GBD cancer mortality estimates and therefore directly determine YLDs. Until cancer registry incidence data and accurate mortality statistics are widely available, validation of MIR is difficult in countries that lack these data sources. The Global Initiative for Cancer Registry Development and expansion of civil registration systems are therefore crucial to further improve estimation of cancer burden.

### Future directions

Challenging data gaps exist in the severity distributions across sequelae for most diseases in the YLD literature. Most data sources for severity are from high-income countries, which probably leads to an underestimation of YLDs in low-income and middle-income countries where the severity of presentation of non-fatal illnesses might be worse than in high-income countries, frequently as a result of late diagnosis and underdiagnosis. Improvements can be made if disease-specific research focuses on routine use of a single established measure of severity in surveys and patient populations and if countries are able to link survey data to a general health assessment instrument, along with improvements in early diagnosis and treatment procedures before illnesses progress to high-severity presentations. Although uncertainty and sample variance will persist at various levels, gaining of greater geographical information on severity of diseases than at present will increase the accuracy of GBD models.

Potential is clearly huge for exploration of this work in relation to development assistance for health,^70^, ^71^ as the connection between health financing and outcomes needs better understanding than at present. Improved understanding will probably help identify the reasons why certain countries have such an impressive record, whereas others are so ineffective with the resources that they have available, at all levels of SDI. More precise measurement of health burden than at present, across countries and at the subnational level, could be tied to health services financing and delivery to identify systematic associations and causal relationships. Additionally, risk factor data, health outcome data, health financing data, and other socioeconomic indicators can be combined to measure and assess health system performance. Furthermore, a core focus moving forward with GBD is to progressively increase the spatial resolution at which we implement estimations to help realise aspirations in precision public health.^72^ A key goal of a geographically refined DALY, at 5 × 5 km spatial resolution—starting with some key CMNN causes and under-5 mortality—is part of a long-term aspiration.^73^

Finally, upcoming GBD work will focus on exploration of future health scenarios, examining the likely burden of disease under different possible trajectories of independent drivers of health. This framework will capture the complex past trends and interdependent relationships in socioeconomic development, risk factors, interventions, morbidity, mortality, and population to both estimate the likely future burden of disease and enable comparison between scenarios based on different sets of assumptions. For instance, it can be used to analyse the likely effect of the introduction and scale-up of a new type of vaccination or different trends in funding for ART for HIV/AIDS. Extension of this work through to DALY and HALE analyses is also part of these future plans.

To look at cause-deleted HALE would also be valuable so that we could understand the remaining DALY burden in the absence of diseases and injuries that might be highly amenable to preventive or curative measures. Compelling examples would include HIV, vaccine-preventable and malnutrition-related diseases, a subset of NCDs that are particularly amenable to health-care or preventive measures (eg, tobacco-related or alcohol-related or some congenital birth defects), and injuries (eg, firearms or transport injuries). This approach would not be fully counterfactual, but would be a first and more rigorous way of showing what conditions contributed to the changes in HALE from 1990 (or 2006) to 2016 than was possible in this study.

### Limitations

Despite our continued methodological advancements and data enrichments, this study has limitations. First, all limitations documented in the elements of the GBD estimation process that allow for DALY and HALE estimation^2^, ^3^, ^4^, ^12^ will contribute to uncertainty in these summary measures. Second, these summary measures will also be influenced by data availability. Time lags in the reporting of health information by national authorities and thus their subsequent incorporation into the GBD estimation mean that some of the most recent changes in health states will not be captured. Relatedly, data deficiencies from populations in conflict zones (eg, Syria, Iraq, Yemen, South Sudan, and Afghanistan), autonomous subnational regions, and certain non-geographically based subpopulations (ie, migrants, refugees, and some indigenous people) limit the precision of some of our estimated levels and trends of disease burden.^74^ Third, the relationship between DALYs, HALE, and SDI, although explanatory, cannot be viewed as causal. Fourth, a non-trivial assumption of the analyses is the independence of the uncertainty calculated for YLLs and YLDs. Because of the link between death and prevalence, a positive correlation probably exists between these uncertainties that we do not capture. As such, we probably underestimate the aggregated uncertainty for DALYs, although the primary source of uncertainty for DALYs comes from uncertainty in disability weights, which are unaffected by this limitation. In future iterations of GBD, this potential correlation will be explored using copula, a statistical method that models the dependence structure among multiple independent marginal probability distributions to estimate correlation between them.^75^

## Conclusion

Many improvements have been made to GBD 2016 to allow for a clearer and more nuanced picture of the changing picture of global health than in GBD 2015. Among these improvements are inclusion of new studies and subnational data (notably in India) and many substantial improvements to modelling and analyses. Prominent results of these changes include higher global DALY estimates for tuberculosis, HIV/AIDS, lower respiratory infection, and diarrhoeal disease than in GBD 2015. We have for the first time discussed our DALY exemplars, countries with the lowest ratio of observed to expected DALYs, as well as our poor performers, those with the largest ratio of observed to expected DALYs. These exemplars and poor performers suggest a need to learn lessons from those with clear health gains and implement system-based strategies for those struggling. This analysis and increasingly more detailed results will allow for more informed public policy and health financing decisions as the world faces an absolute expansion of morbidity.

Globally, individuals could expect to live substantially longer lives in 2016 than they could in 1990. This improvement was due to a rapid decline in YLLs and more modest age-standardised declines in YLDs, leading to lower age-standardised DALY rates across the entire socioeconomic development spectrum in 2016 than in 1990, with a decrease of approximately a third for all causes. At the same time, populations can expect to spend more time with functional health loss due to absolute morbidity expansion than previously. Such improvements have been accompanied by rapid population growth and ageing and, against the backdrop of the epidemiological transition, have resulted in the paradox of a total expansion in DALY burden and thus an ever-increasing demand on health systems, domestic health financing, development assistance for health, and associated global health organisations. This increasing demand on health systems is ubiquitous across time, location, GBD region, and SDI, posing a huge opportunity for health-care innovators in prevention and treatment for morbidity reduction. Our analysis of exemplars and poor performers is also indicative of various practices that can hasten or slow the epidemiological transition and warrant deep investigation.

Correspondence to: Prof Simon Iain Hay, Institute for Health Metrics and Evaluation, Seattle, WA 98121, USA sihay@uw.edu

For the **Global Health Data Exchange** see http://ghdx.healthdata.org/node/311214
