# Department of Error

**DOI:** 10.1016/S0140-6736(17)32648-X

**Published:** 2017-10-28

*GBD 2016 DALYs and HALE Collaborators. Global, regional, and national disability-adjusted life-years (DALYs) for 333 diseases and injuries and healthy life expectancy (HALE) for 195 countries and territories, 1990–2016: a systematic analysis for the Global Burden of Disease Study 2016.* Lancet *2017; **390**: 1260–1344*. The full-text version of this Article has been updated so that the list of authors is displayed in the correct order, in line with the pdf version, rather than in alphabetical order. This correction has been made to the online version as of Oct 12, 2017.

