# Supplementary material for: Global, regional, and national disability-adjusted life-years (DALYs) for 333 diseases and injuries and healthy life expectancy (HALE) for 195 countries and territories, 1990–2016: a systematic analysis for the Global Burden of Disease Study 2016
Source: Lancet. 2017 Sep 16;390(10100):1260–344. doi: 10.1016/S0140-6736(17)32130-X (PMC5605707; doi:10.1016/S0140-6736(17)32130-X)
Supplement: Supplementary appendix [file mmc1.pdf]

THE LANCET

Supplementary appendix

This appendix formed part of the original submission and has been peer reviewed. We post it as supplied by the authors.

Supplement to: GBD 2016 DALYs and HALE Collaborators. Global, regional, and national disability-adjusted life-years (DALYs) for 333 diseases and injuries and healthy life expectancy (HALE) for 195 countries and territories, 1990–2016: a systematic analysis for the Global Burden of Disease Study 2016. *Lancet* 2017; **390:** 1260–344.

Appendix to Global, regional, and national disability-adjusted life-years (DALYs) for 333 diseases and injuries and healthy life expectancy (HALE) for 195 countries and territories, 1990–2016: a systematic analysis for the Global Burden of Disease Study 2016

This appendix provides methodological detail, supplemental figures, and more detailed results for disability-adjusted life years and healthy life expectancy. The appendix is organized into broad sections following the structure of the main paper.

Preamble

This appendix provides methodological detail and detailed results for the estimation of disability-adjusted life years (DALYs) and healthy life expectancy (HALE) for 333 causes of disease, disability, and injuries from the Global Burden of Disease (GBD) 2016. This study complies with the Guidelines for Accurate and Transparent Health Estimates Reporting (GATHER) recommendations. It includes detailed tables and information on data in an effort to maximize transparency in our estimation processes and provide a comprehensive description of analytical steps. We intend this to be a living document, to be updated with each annual iteration of the GBD study.

Authors' Contributions

Managing the estimation process

Ashkan Afshin, Tahiya Alam, Megha Arora, James Bennett, Blair R Bumgarner, Kara Estep, Tatiana Fedosseeva, Christina Fitzmaurice, Emmanuela Gakidou, Jamie Hancock, Caitie Hawley, Simon I Hay, Chantal Huynh, Sarah C Johnson, Nicholas J Kassebaum, Michael Kutz, Darya Li Kappe, Stephen S Lim, Madeline McGaughey, Christopher J L Murray, Mohsen Naghavi, Helen E Olsen, Christine Pinho, Puja Rao, Robert Reiner, Gregory A Roth, Joseph Salama, Katya Shackelford, Chloe Shields, Shreya Shirude, Amber Sligar, Mari Smith, Vinay Srinivasan, Jeffrey Stanaway, Caitlyn Steiner, Theo Vos, Joe Wagner, Haidong Wang, and Leo Zoeckler.

Writing the first draft of the manuscript

Simon I Hay and Sarah E Ray.

Providing data or critical feedback on data sources

Cristiana Abbafati, Foad Abd-Allah, Semaw Ferede Abera, Laith J Abu-Raddad, Ilana N Ackerman, Olatunji Adetokunboh, Rakesh Aggarwal, Aliasghar Ahmad Kiadaliri, Muktar Beshir Ahmed, Amani Nidhal Aichour, Ibtihel Aichour, Miloud Taki Eddine Aichour, Tomi F Akinyemiju, Nadia Akseer, Khurshid Alam, Deena Alasfoor, Kefyalew Addis Alene, Raghib Ali, Juma M Alkaabi, Ala'a Alkerwi, François Alla, Peter Allebeck, Fatma Al-Maskari, Mohammad AbdulAziz AlMazroa, Ubai Alsharif, Benjamin M Althouse, Khalid A. Altirkawi, Azmeraw T. Amare, Erfan Amini, Walid Ammar, Mustafa Geleto Ansha, Carl Abelardo T Antonio, Palwasha Anwari, Johan Ärnlöv, Al Artaman, Krishna Kumar Aryal, Solomon W Asgedom, Ashish Awasthi, Shally Awasthi, Umar Bacha, Kalpana Balakrishnan, Aleksandra Barac, Till Bärnighausen, Simon Barquera, Lope H. Barrero, Sanjay Basu, Bernhard T Baune, Justin Beardsley, Neeraj Bedi, Yannick Béjot, Bayu Begashaw Bekele, Derrick A. Bennett, Isabela M Bensenor, Balem Demtsu Betsu, Mircea Beuran, Addisu Shunu Beyene, Neeraj Bhala, Anil Bhansali, Kelly Bienhoff, Boris Bikbov, Charles Birungi, Donal Bisanzio, Dube Jara Boneya, Ibrahim R. Bou-Orm, Rupert R A Bourne, Michael Brainin, Gabrielle Britton, Traolach Brugha, Lemma Negesa Bulto Bulto, Blair Bumgarner, Hélène Carabin, David O. Carpenter, Juan-Jesus Jesus Carrero, Felix Carvalho, Carlos A. Castañeda-Orjuela, Chris Castle, Ferrán Catalá-López, Fiona J Charlson, Honglei Chen, Mirriam Chibalabala, Abdulaal A Chitheer, Rajiv Chowdhury, Devasahayam Jesudas Christopher, Massimo Cirillo, Cyrus Cooper, Leslie Trumbull Cooper, Monica Cortinovis, Michael H. Criqui, Michael Criqui, Elizabeth Cromwell, Marita Cross, John A Crump, Albertino Damasceno, Lalit Dandona, Rakhi Dandona, José das Neves, Louisa Degenhardt, Kebede Deribe, Amare Deribew, Subhojit Dey, Samath D. Dharmaratne, Preet Kaur Dhillon, Huyen Phuc Do, Klara Dokova, E. Ray Dorsey, Tim R. Driscoll, Manisha Dubey, Bruce Bartholow Duncan, Beth E. Ebel, Sergey Petrovich Ermakov, Holly E Erskine, Alireza Esteghamati, Kara Estep, Tamer H Farag, Carla Sofia e Sa Farinha, Andre Faro, Farshad Farzadfar, Mir Sohail Fazeli, Valery L. Feigin, Andrea B Feigl, Seyed-Mohammad Fereshtehnejad, João C Fernandes, Alize J. Ferrari, Tesfaye Regassa Feyissa, Florian Fischer, Abraham D Flaxman, Richard C. Franklin, Nancy Fullman, Thomas Fürst, João M M Furtado, Neal D. Futran, Emmanuela Gakidou, Teshome Gebre, Johanna M Geleijnse, Ayele Geleto, Hailay Abrha Gesesew, Peter W Gething, Alireza Ghajar, Katherine B Gibney, Ibrahim Abdelmageem Mohamed Ginawi, Melkamu Dedefo Gishu, Shifalika Goenka, Philimon N Gona, Atsushi Goto, Harish Chander Gugnani, Prakash C. Gupta, Tanush Gupta, Nima Hafezi-Nejad, Alemayehu Desalegne Hailu, Gessessew Bugssa Hailu, Hilda L Harb, Habtamu Abera Hareri, Josep Maria Haro, James Harvey, Mohammad Sadegh Hassanvand, Rasmus Havmoeller, Roderick J Hay, Simon I. Hay, Mohammad T. Hedayati, Delia Hendrie,

[i]Nathaniel J. Henry, Ileana Beatriz Heredia-Pi, Pouria Heydarpour, Hans W. Hoek, Howard J. Hoffman, Nobuyuki Horita, Peter J. Hotez, Damian G. Hoy, Aung Soe Htet, Guoqing Hu, Kim Moesgaard Iburg, Kathryn H. Jacobsen, Mihajlo B Jakovljevic, Simerjot K. Jassal, Mehdi Javanbakht, Panniyammakal Jeemon, Paul N Jensen, Vivekanand Jha, Catherine Johnson, Jost B Jonas, Mikk Jürisson, Zubair Kabir, Rajendra Kadel, Ritul Kamal, Chittaranjan Kar, André Karch, Corine Kakizi Karema, Amir Kasaeian, Nigussie Assefa Kassaw, Srinivasa Vittal Katikireddi, Vittal Katikireddi, Anil Kaul, Norito Kawakami, Andre Keren, Chandrasekharan Nair Kesavachandran, Yousef Saleh Khader, Ibrahim A Khalil, Young-Ho Khang, Abdullah T Khoja, Ardeshir Khosravi, Jagdish Khubchandani, Christian Kieling, Yun Jin Kim, Yohannes Kinfu, Mika Kivimaki, Ann Kristin Knudsen, Yoshihiro Kokubo, Soewarta Kosen, Michael Kravchenko, Barthelemy Kuate Defo, Burcu Kucuk Bicer, G Anil Kumar, Pushpendra Kumar, Hmwe H Kyu, Anton Carl Jonas Lager, Dharmesh Kumar Lal, Nkurunziza Lambert, Qing Lan, Janet L Leasher, James Leigh, Cheru Tesema Leshargie, Janni Leung, Ricky Leung, Miriam Levi, Yichong Li, Xiaofeng Liang, Misgan Legesse Liben, Stephen S Lim, Shiwei Liu, Katharine J. Looker, Alan D. Lopez, Stefan Lorkowski, Paulo A. Lotufo, Rafael Lozano, Ronan A Lyons, Hassan Magdy Abd El Razek, Mohammed Magdy Abd El Razek, Carlos Magis-Rodriguez, Marek Majdan, Azeem Majeed, Reza Malekzadeh, Abdullah A Mamun, Lorenzo G Mantovani, Lyn M. March, Ira Martopullo, Winfried März, Manu Raj Mathur, Mohsen Mazidi, Colm McAlinden, John J McGrath, Martin McKee, Suresh Mehata, Toni Meier, Kidanu Gebremariam Meles, Ziad A. Memish, Walter Mendoza, Melkamu Merid Mengesha, Desalegn Tadese Mengistu, Atte Meretoja, Renata Micha, Ted R Miller, Shiva Raj Mishra, Shafiu Mohammed, Murali B V Mohan, Ali H Mokdad, Lorenzo Monasta, Julio Cesar Montañez Hernandez, Marcella Montico, Maziar Moradi-Lakeh, Rintaro Mori, Ulrich O. Mueller, Christopher J L Murray, Gudlavalleti Venkata Satyanarayana Murthy, Kamarul Imran Musa, Jean B Nachega, Gabriele Nagel, Mohsen Naghavi, Kovin S. Naidoo, Ionut Negoi, Ruxandra Irina Negoi, Charles R Newton, Cuong Tat Nguyen, Minh Nguyen, Quyen Le Nguyen, Trang Huyen Nguyen, Vuong Minh Nong, Bo Norrving, Alypio Nyandwi, Felix Akpojene Ogbo, Olanrewaju Oladimeji, Andrew Toyin Olagunju, Bolajoko Olubukunola Olusanya, Jacob Olusegun Olusanya, Kanyin Ong, Majdi Osman, Erika Ota, Mayowa O Owolabi, Mahesh PA, Basant Kumar Panda, Christina Papachristou, Scott B. Patten, Neil Pearce, David M Pereira, Krystle Marie Perez, Norberto Perico, Konrad Pesudovs, William Arthur Petri, Max Petzold, David M Pigott, Dietrich Plass, Martin Pletcher, Farshad Pourmalek, Dorairaj Prabhakaran, Narayan Prasad, Mostafa Qorbani, Rynaz H S Rabiee, Anwar Rafay, Afarin Rahimi-Movaghar, Vafa Rahimi-Movaghar, Rajesh Kumar Rai, Sasa Rajsic, Usha Ram, Chhabi Lal Ranabhat, Salman Rawaf, Giuseppe Remuzzi, Antonio L. Ribeiro, David Rojas-Rueda, Mohammad Bagher Rokni, Luca Ronfani, Gholamreza Roshandel, Gregory A Roth, Dietrich Rothenbacher, Enrico Rubagotti, Soheil Saadat, Saeid Safiri, Rajesh Sagar, Mohammad Ali Sahraian, Joshua A. Salomon, Sundeep Santosh Salvi, Abdallah M. Samy, Juan Ramon Sanabria, Damian Santomauro, Itamar S Santos, Milena M Santric Milicevic, Benn Sartorius, Maheswar Satpathy, Monika Sawhney, Sonia Saxena, Maria Inês Schmidt, Aletta E Schutte, Daivd C Schwebel, Falk Schwendicke, Soraya Seedat, Sadaf G Sepanlou, Edson E. Servan-Mori, Masood Ali Shaikh, Sheikh Mohammed Shariful Islam, Jayendra Sharma, Reza Shirkoohi, Haitham Shoman, Abla Mehio Sibai, Diego Augusto Santos Silva, Dayane Gabriele Alves Silveira, Jasvinder A Singh, Narinder Pal Singh, Virendra Singh, Eirini Skiadaresi, Erica Leigh Slepak, Michael Soljak, Luciano A. Sposato, Chandrashekhar T Sreeramareddy, Vasiliki Stathopoulou, Caitlyn Steiner, Mark Andrew Stokes, Lars Jacob Stovner, Muawiyyah Babale Sufiyan, Rizwan Suliankatchi Abdulkader, Patrick J. Sur, Soumya Swaminathan, Bryan L Sykes, Dillon Sylte, Cassandra E I Szoeke, Rafael Tabarés-Seisdedos, Jukka S Takala, David Tanne, Mohammad Tavakkoli, Nuno Taveira, Hugh R Taylor, Girma Temam Shifa, Abdullah Sulieman Terkawi, Ornwipa Thamsuwan,

Nihal Thomas, Ruoyan Tobe-Gai, Marcello Tonelli, Bach Xuan Tran, Christopher Troeger, Thomas Truelsen, Stefanos Tyrovolas, Kingsley Nnanna Ukwaja, Eduardo A Undurraga, Chigozie Jesse Uneke, Olalekan A Uthman, Job FM F M van Boven, Tommi Vasankari, Lennert J Veerman, Narayanaswamy Venketasubramanian, Ramesh Vidavalur, Francesco S. Violante, Vasiliy Victorovich Vlassov, Stein Emil Vollset, Theo Vos, Tolassa Wakayo, Yuan-Pang Wang, Elisabete Weiderpass, Robert G Weintraub, Andrea Werdecker, Ronny Westerman, Harvey A. Whiteford, Tissa Wijeratne, Hywel C Williams, Charles Shey Shey Wiysonge, Charles D A Wolfe, Rachel Woodbrook, Anthony D Woolf, Abdulhalik Workicho, Denis Xavier, Gelin Xu, Simon Yadgir, Bereket Yakob, Lijing L. Yan, Pengpeng Ye, Hassen Hamid Yimam, Naohiro Yonemoto, Marcel Yotebieng, Mustafa Z Younis, Chuanhua Yu, Zoubida Zaidi, Maysaa El Sayed Zaki, Xueying Zhang, Yingfeng Zheng, Maigeng Zhou, and Liesl Joanna Zuhlke.

Developing methods or computational machinery

Christine Allen, Benjamin M Althouse, Solomon W Asgedom, Marlena Bannick, Katherine E Battle, Samir Bhatt, Charles Birungi, Stan Biryukov, Donal Bisanzio, Lemma Negesa Bulto Bulto, Blair R Bumgarner, Ewan Cameron, Austin Carter, Chris Castle, Fiona J Charlson, Devasahayam Jesudas Christopher, Daniel Dicker, Holly E Erskine, Mir Sohail Fazeli, Alize J Ferrari, Christina Fitzmaurice, Abraham D Flaxman, Kyle J Foreman, Joseph Frostad, Emmanuela Gakidou, Ayele Geleto, Peter W Gething, Ellen Goldberg, Nicholas Graetz, James Harvey, Nathaniel J Henry, Peter J Hotez, Catherine Johnson, Sarah Charlotte Johnson, Jagdish Khubchandani, Janni Leung, Stephen S Lim, Angela Liu, Rafael Lozano, Timothy C D Lucas, Treh Manhertz, Ira Martopullo, Madeline McGaughey, Anoushka Millear, Mark Moses, Christopher J L Murray, Mohsen Naghavi, Grant Nguyen, Kanyin Ong, Richard H Osborne, Aaron Osgood-Zimmerman, Charles D Parry, Katherine Paulson, Geoffrey Phipps, Christine Pinho, Zane Rankin, Robert C Reiner, Gregory A Roth, Joshua A Salomon, Abdallah M Samy, Damian Santomauro, Maheswar Satpathy, David L Smith, Vinay Srinivasan, Jeffrey Stanaway, Dillon Sylte, Ornwipa Thamsuwan, Katie E Thomas, Christopher Troeger, Derrick Tsoi, Hayley Tymeson, Rachel Updike, Theo Vos, Daniel J Weiss, Gelin Xu, Simon Yadgir, Mustafa Z Younis, and Leo Zoeckler.

Applying analytical methods to produce estimates

Muktar Beshir Ahmed, Fares Alahdab, Christine Allen, Marlena Bannick, Katherine E Battle, James Bennett, Adugnaw Berhane, Marissa Bettay, Samir Bhatt, Charles Birungi, Stan Biryukov, Donal Bisanzio, Dube Jara Boneya, Lemma Negesa Bulto Bulto, Blair Bumgarner, Ewan Cameron, Austin Carter, Daniel Casey, Fiona J Charlson, Devasahayam Jesudas Christopher, Danny Colombara, Elizabeth Cromwell, Daniel Dicker, Aman Yesuf Endries, Holly E Erskine, Mir Sohail Fazeli, Alize J Ferrari, Christina Fitzmaurice, Abraham D Flaxman, Joseph Frostad, Emmanuela Gakidou, Peter W Gething, William W Godwin, Ellen Goldberg, Peter J Hotez, Caleb Irvine, Catherine Johnson, Hmwe H Kyu, Janni Leung, Stephen S Lim, Angela Liu, Rafael Lozano, Timothy C D Lucas, Emilie Maddison, Helena Manguerra, Treh Manhertz, Ira Martopullo, Madeline McGaughey, Anoushka Millear, Ali H Mokdad, Sarah K Mollenkopf, Mark Moses, Cliff Mountjoy-Venning, Christopher J L Murray, Mohsen Naghavi, Grant Nguyen, Minh Nguyen, Emma Nichols, Helen E Olsen, Kanyin Ong, Katherine Paulson, David M Pigott, Christine Pinho, Martin Pletcher, Caroline Purcell, Usha Ram, Sarah E Ray, Robert C Reiner, Gregory A Roth, Enrico Rubagotti, Joshua A Salomon, Damian Santomauro, Maheswar Satpathy, Vinay Srinivasan, Jeffrey Stanaway, Bryan Strub, Patrick J Sur, Ornwipa Thamsuwan, Christopher Troeger, Derrick Tsoi, Hayley Tymeson, Rachel Updike, Theo Vos, Tolassa Wakayo, Daniel J Weiss, Ronny Westerman, Tissa Wijeratne, Gelin Xu, Simon Yadgir, Hassen Hamid Yimam, and Leo Zoeckler.

Providing critical feedback on methods or results

Amanuel Alemu Abajobir, Kalkidan Hassen Abate, Cristiana Abbafati, Kaja M Abbas, Teshome Abuka Abebo, Semaw Ferede Abera, Victor Aboyans, Laith J Abu-Raddad, Ilana N Ackerman, Olatunji Adetokunboh, Mohsen Afarideh, Anurag Agrawal, Aliasghar Ahmad Kiadaliri, Muktar Beshir Ahmed, Amani Nidhal Aichour, Ibtihel Aichour, Miloud Taki Eddine Aichour, Tomi F Akinyemiju, Nadia Akseer, Fares Alahdab, Ziyad Al-Aly, Khurshid Alam, Noore Alam, Deena Alasfoor, Kefyalew Addis Alene, Raghib Ali, Reza Alizadeh-Navaei, Juma M Alkaabi, Ala'a Alkerwi, Peter Allebeck, Fatma Al-Maskari, Rajaa Al-Raddadi, Ubai Alsharif, Benjamin M Althouse, Khalid A Altirkawi, Nelson Alvis-Guzman, Azmeraw T Amare, Erfan Amini, Yaw Ampem Amoako, Mustafa Geleto Ansha, Carl Abelardo T Antonio, Palwasha Anwari, Johan Ärnlöv, Al Artaman, Krishna Kumar Aryal, Solomon W Asgedom, Tesfay Mehari Atey, Niguse Tadele Atnafu, Leticia Avila-Burgos, Euripide Frinel G Arthur Avokpaho, Ashish Awasthi, Shally Awasthi, Beatriz Paulina Ayala Quintanilla, Mahmoud Reza Azarpazhooh, Peter Azzopardi, Tesleem Kayode Babalola, Umar Bacha, Alaa Badawi, Aleksandra Barac, Suzanne L Barker-Collo, Till Bärnighausen, Simon Barquera, Lope H Barrero, Sanjay Basu, Katherine E Battle, Bernhard T Baune, Shahrzad Bazargan-Hejazi, Justin Beardsley, Neeraj Bedi, Bayu Begashaw Bekele, Michelle L Bell, Derrick A Bennett, Isabela M Bensenor, Adugnaw Berhane, Derbew Fikadu Berhe, Eduardo Bernabé, Balem Demtsu Betsu, Marissa Bettay, Mircea Beuran, Addisu Shunu Beyene, Neeraj Bhala, Samir Bhatt, Zulfiqar A Bhutta, Sibhatu Biadgilign, Kelly Bienhoff, Boris Bikbov, Charles Birungi, Donal Bisanzio, Habtamu Mellie Bizuayehu, Fiona M Blyth, Dube Jara Boneya, Dipan Bose, Rupert R A Bourne, Alexandra Brazinova, Nicholas J K Breitborde, Traolach Brugha, Rachelle Buchbinder, Lemma Negesa Bulto Bulto, Blair Bumgarner, Zahid A Butt, Lucero Cahuana-Hurtado, Ewan Cameron, Ismael Ricardo Campos-Nonato, Rosario Cárdenas, Austin Carter, Felix Carvalho, Carlos A Castañeda-Orjuela, Jacqueline Castillo Rivas, Ferrán Catalá-López, Jung-Chen Chang, Fiona J Charlson, Pankaj Chaturvedi, Chioma Ezinne Chibueze, Vesper Hichilombwe Chisumpa, Devasahayam Jesudas Christopher, Liliana G Ciobanu, Michael H Criqui, Michael Criqui, Elizabeth Cromwell, John A Crump, Koustuv Dalal, Lalit Dandona, Rakhi Dandona, José das Neves, Kairat Davletov, Barbora de Courten, Diego De Leo, Hans De Steur, Louisa Degenhardt, Kebede Deribe, Amare Deribew, Don C Des Jarlais, Subhojit Dey, Samath D Dharmaratne, Daniel Dicker, Huyen Phuc Do, Klara Dokova, David Teye Doku, Tim R Driscoll, Manisha Dubey, Bruce Bartholow Duncan, Ziad Ziad El-Khatib, Ahmadali Enayati, Aman Yesuf Endries, Holly E Erskine, Setegn Eshetie, Babak Eshrati, Alireza Esteghamati, Kara Estep, Fanuel Belayneh Bekele Fanuel, Tamer H Farag, Carla Sofia e Sa Farinha, Andre Faro, Farshad Farzadfar, Mir Sohail Fazeli, Andrea B Feigl, Seyed-Mohammad Fereshtehnejad, João C Fernandes, Alize J Ferrari, Tesfaye Regassa Feyissa, Irina Filip, Florian Fischer, Christina Fitzmaurice, Abraham D Flaxman, Nataliya Foigt, Kyle J Foreman, Richard C Franklin, Joseph Frostad, Nancy Fullman, Thomas Fürst, João M M Furtado, Neal D Futran, Emmanuela Gakidou, Morsaleh Ganji, Teshome Gebre, Gebremedhin Berhe Gebregergs, Tsegaye Tewelde Gebrehiwot, Ayele Geleto, Bikila Lencha Gemechu, Hailay Abrha Gesesew, Peter W Gething, Katherine B Gibney, Melkamu Dedefo Gishu, Giorgia Giussani, Kashish Goel, Philimon N Gona, Amador Goodridge, Sameer Vali Gopalani, Richard A Gosselin, Carolyn C Gotay, Atsushi Goto, Alessandra Carvalho Goulart, Harish Chander Gugnani, Rahul Gupta, Rajeev Gupta, Tanush Gupta, Vipin Gupta, Reyna A Gutiérrez, Vladimir Hachinski, Nima Hafezi-Nejad, Alemayehu Desalegne Hailu, Gessessew Bugssa Hailu, Randah Ribhi Hamadeh, Samer Hamidi, Mouhanad Hammami, Graeme J Hankey, Yuantao Hao, Habtamu Abera Hareri, Josep Maria Haro, Kimani M Harun, Rasmus Havmoeller, Roderick J Hay, Simon I Hay, Mohammad T Hedayati, Delia Hendrie, Ileana Beatriz Heredia-Pi, Masako Horino, Nobuyuki Horita, H Dean Hosgood, Sorin Hostiuc, Peter J Hotez, Guoqing Hu, John J Huang, Ehimario Uche Igumbor, Kathryn H Jacobsen, Nader Jahanmehr, Mihajlo B Jakovljevic, Peter

James, Mehdi Javanbakht, Sudha P Jayaraman, Panniyammakal Jeemon, Paul N Jensen, Vivekanand Jha, Guohong Jiang, Denny John, Catherine Johnson, Sarah Charlotte Johnson, Jost B Jonas, Mikk Jürisson, Rajendra Kadel, Amaha Kahsay, Ritul Kamal, Nadim E Karam, André Karch, Seyed M Karimi, Chante Karimkhani, Amir Kasaeian, Getachew Mullu Kassa, Nigussie Assefa Kassaw, Anshul Kastor, Norito Kawakami, Peter Njenga Keiyoro, Chandrasekharan Nair Kesavachandran, Yousef Saleh Khader, Young-Ho Khang, Christian Kieling, Daniel Kim, Yun Jin Kim, Yohannes Kinfu, Adnan Kisa, Katarzyna A Kissimova-Skarbek, Niranjan Kissoon, Mika Kivimaki, Ann Kristin Knudsen, Dhaval Kolte, Jacek A Kopec, Georgios A Kotsakis, Parvaiz A Koul, Ai Koyanagi, Michael Kravchenko, Kristopher J Krohn, Barthelemy Kuate Defo, G Anil Kumar, Pushpendra Kumar, Hmwe H Kyu, Dharmesh Kumar Lal, Ratilal Lalloo, Tea Lallukka, Nkurunziza Lambert, Faris Lami, Qing Lan, Van C Lansingh, Anders Larsson, Janet L Leasher, Paul H Lee, James Leigh, Cheru Tesema Leshargie, Janni Leung, Miriam Levi, Yongmei Li, Misgan Legesse Liben, Stephen S Lim, Shai Linn, Yang Liu, Rakesh Lodha, Katharine J Looker, Alan D Lopez, Stefan Lorkowski, Paulo A Lotufo, Rafael Lozano, Ronan A Lyons, Erlyn Rachelle King Macarayan, Hassan Magdy Abd El Razek, Mohammed Magdy Abd El Razek, Carlos Magis-Rodriguez, Mahdi Mahdavi , Marek Majdan, Reza Majdzadeh, Azeem Majeed, Reza Malekzadeh, Rajesh Malhotra, Deborah Carvalho Malta, Abdullah A Mamun, Treh Manhertz, Chabila C Mapoma, Lyn M March, Laurie B Marczak, Jose Martinez-Raga, Paulo Henrique Viegas Martins, Francisco Rogerlândio Martins-Melo, Manu Raj Mathur, Mohsen Mazidi, Colm McAlinden, John J McGrath, Martin McKee, Suresh Mehata, Toni Meier, Kidanu Gebremariam Meles, Peter Memiah, Walter Mendoza, Melkamu Merid Mengesha, Mubarek Abera Mengistie, Desalegn Tadese Mengistu, George A Mensah, Atte Meretoja, Tuomo J Meretoja, Haftay Berhane Mezgebe, Renata Micha, Ted R Miller, Mojde Mirarefin, Erkin M Mirrakhimov, Shiva Raj Mishra, Philip B Mitchell, Alireza Mohammadi, Kedir Endris Mohammed, Muktar Sano Kedir Mohammed, Shafiu Mohammed, Murali B V Mohan, Ali H Mokdad, Sarah K Mollenkopf, Marcella Montico, Lidia Morawska, Shane D Morrison, Kalayu Birhane Mruts, Ulrich O Mueller, Kate Muller, Christopher J L Murray, Gudlavalleti Venkata Satyanarayana Murthy, Kamarul Imran Musa, Jean B Nachega, Gabriele Nagel, Mohsen Naghavi, Aliya Naheed, Vinay Nangia, Jamal T Nasher, Gopalakrishnan Natarajan, Dumessa Edessa Negasa, Ionut Negoi, Ruxandra Irina Negoi, Charles R Newton, Josephine Wanjiku Ngunjiri, Cuong Tat Nguyen, Grant Nguyen, Quyen Le Nguyen, Trang Huyen Nguyen, Dina Nur Anggraini Ningrum, Vuong Minh Nong, Bo Norrving, Jean Jacques N Noubiap, Carla Makhlouf Obermeyer, Martin J O'Donnell, Felix Akpojene Ogbo, In-Hwan Oh, Anselm Okoro, Olanrewaju Oladimeji, Andrew Toyin Olagunju, Helen E Olsen, Bolajoko Olubukunola Olusanya, Jacob Olusegun Olusanya, Kanyin Ong, John Nelson Opio, Eyal Oren, Alberto Ortiz, Richard H Osborne, Erika Ota, Mayowa O Owolabi, Mahesh PA, Rosana E Pacella, Basant Kumar Panda, Eun-Kee Park, Snehal T Patil, George C Patton, Deepak Paudel, Katherine Paulson, David M Pereira, Norberto Perico, Konrad Pesudovs, Michael Robert Phillips, David M Pigott, Julian David Pillay, Dietrich Plass, Svetlana Popova, Richie G Poulton, Farshad Pourmalek, Dorairaj Prabhakaran, Narayan Prasad, Manorama Purwar, Mostafa Qorbani, Amir Radfar, Kazem Rahimi, Afarin Rahimi-Movaghar, Vafa Rahimi-Movaghar, Mahfuzar Rahman, Mohammad Hifz Ur Rahman, Muhammad Aziz Rahman, Rajesh Kumar Rai, Usha Ram, Chhabi Lal Ranabhat, Thara Rangaswamy, Paturi Vishnupriya Rao, Puja C Rao, Salman Rawaf, Sarah E Ray, Robert C Reiner, Giuseppe Remuzzi, Andre M N Renzaho, Serge Resnikoff, Satar Rezaei, Antonio L Ribeiro, Hirbo Shore Roba, Stephen R Robinson, Luca Ronfani, Gholamreza Roshandel, Gregory A Roth, Dietrich Rothenbacher, Ambuj Roy, George Mugambage Ruhago, Mahdi Safdarian, Saeid Safiri, Rajesh Sagar, Mohammad Ali Sahraian, Muhammad Muhammad Saleh, Joshua A Salomon, Sundeep Santosh Salvi, Abdallah M Samy, Juan Ramon Sanabria, Maria Dolores Sanchez-Niño, Damian Santomauro, Itamar S Santos, Milena M Santric Milicevic, Benn Sartorius,

Maheswar Satpathy, Monika Sawhney, Sonia Saxena, Maria Inês Schmidt, Ben Schöttker, Aletta E Schutte, Daivd C Schwebel, Falk Schwendicke, Soraya Seedat, Sadaf G Sepanlou, Amira Shaheen, Masood Ali Shaikh, Mansour Shamsipour, Sheikh Mohammed Shariful Islam, Jayendra Sharma, Rajesh Sharma, Chloe Shields, Mekonnen Sisay Shiferaw, Mika Shigematsu, Rahman Shiri, Reza Shirkoohi, Kawkab Shishani, Haitham Shoman, Soraya Siabani, Inga Dora Sigfusdottir, Diego Augusto Santos Silva, João Pedro Pedro Silva, Dayane Gabriele Alves Silveira, Jasvinder A Singh, Narinder Pal Singh, Om Prakash Singh, Eirini Skiadaresi, Mari Smith, Badr H A Sobaih, Eugene Sobngwi, Michael Soljak, Reed J D Sorensen, Tatiane Cristina Moraes Sousa, Luciano A Sposato, Chandrashekhar T Sreeramareddy, Jeffrey Stanaway, Vasiliki Stathopoulou, Nicholas Steel, Caitlyn Steiner, Sabine Steinke, Mark Andrew Stokes, Muawiyyah Babale Sufiyan, Rizwan Suliankatchi Abdulkader, Bruno F Sunguya, Soumya Swaminathan, Bryan L Sykes, Cassandra E I Szoeke, Rafael Tabarés-Seisdedos, Getachew Redae Taffere, Jukka S Takala, Nikhil Tandon, Yihunie L Tarekegn, Hugh R Taylor, Teketo Kassaw Tegegne, Tesfalidet Tekelab, Girma Temam Shifa, Abdullah Sulieman Terkawi, Dawit Jember Tesfaye, Belay Tesssema, JS Thakur, Alice M Theadom, Nihal Thomas, Robert Thompson, Amanda G Thrift, Ruoyan Tobe-Gai, Marcello Tonelli, Roman Topor-Madry, Miguel Tortajada-Girbés, Mahilde Touvier, Bach Xuan Tran, Thomas Truelsen, Derrick Tsoi, Stefanos Tyrovolas, Kingsley Nnanna Ukwaja, Eduardo A Undurraga, Chigozie Jesse Uneke, Olalekan A Uthman, Benjamin S Chudi Uzochukwu, Job FM F M van Boven, Santosh Varughese, Tommi Vasankari, Srinivasaraghavan Venkatesh, Narayanaswamy Venketasubramanian, Francesco S Violante, Abhishek Vishnu, Sergey K Vladimirov, Vasiliy Victorovich Vlassov, Stein Emil Vollset, Theo Vos, Fiseha Wadilo, Tolassa Wakayo, Mitchell T Wallin, Yuan-Pang Wang, Scott Weichenthal, Elisabete Weiderpass, Robert G Weintraub, Andrea Werdecker, Ronny Westerman, Harvey A Whiteford, Tissa Wijeratne, Hywel C Williams, Charles Shey Shey Wiysonge, Belete Getahun Woldeyes, Charles D A Wolfe, Anthony D Woolf, Abdulhalik Workicho, Denis Xavier, Gelin Xu, Mohsen Yaghoubi, Bereket Yakob, Lijing L Yan, Yuichiro Yano, Mahari Gidey Yihdego, Hassen Hamid Yimam, Paul Yip, Naohiro Yonemoto, Seok-Jun Yoon, Marcel Yotebieng, Mustafa Z Younis, Chuanhua Yu, Zoubida Zaidi, Maysaa El Sayed Zaki, Elias Asfaw Zegeye, Zerihun Menlkalew Zenebe, Sanjay Zodpey, and Liesl Joanna Zuhlke.

Drafting the work or revising is critically for important intellectual content

Amanuel Alemu Abajobir, Kalkidan Hassen Abate, Cristiana Abbafati, Foad Abd-Allah, Semaw Ferede Abera, Ilana N Ackerman, Olatunji Adetokunboh, Mohsen Afarideh, Sutapa Agrawal, Aliasghar Ahmad Kiadaliri, Muktar Beshir Ahmed, Miloud Taki Eddine Aichour, Tomi F Akinyemiju, Fares Alahdab, Khurshid Alam, Raghib Ali, Juma M Alkaabi, Ala'a Alkerwi, Peter Allebeck, Fatma Al-Maskari, Nelson Alvis-Guzman, Azmeraw T Amare, Erfan Amini, Walid Ammar, Yaw Ampem Amoako, Carl Abelardo T Antonio, Palwasha Anwari, Johan Ärnlöv, Tesfay Mehari Atey, Leticia Avila-Burgos, Euripide Frinel G Arthur Avokpaho, Ashish Awasthi, Peter Azzopardi, Alaa Badawi, Aleksandra Barac, Suzanne L Barker-Collo, Till Bärnighausen, Lope H Barrero, Sanjay Basu, Bernhard T Baune, Shahrzad Bazargan-Hejazi, Neeraj Bedi, Michelle L Bell, Isabela M Bensenor, Adugnaw Berhane, Eduardo Bernabé, Balem Demtsu Betsu, Addisu Shunu Beyene, Neeraj Bhala, Sibhatu Biadgilign, Donal Bisanzio, Habtamu Mellie Bizuayehu, Dube Jara Boneya, Traolach Brugha, Lemma Negesa Bulto Bulto, Blair Bumgarner, Zahid A Butt, Lucero Cahuana-Hurtado, Ismael Ricardo Campos-Nonato, Juan-Jesus Jesus Carrero, Carlos A Castañeda-Orjuela, Jacqueline Castillo Rivas, Ferrán Catalá-López, Fiona J Charlson, Chioma Ezinne Chibueze, Devasahayam Jesudas Christopher, Paolo Angelo Cortesi, Michael H Criqui, Michael Criqui, John A Crump, Abel Fekadu Dadi, Koustuv Dalal, Lalit Dandona, Rakhi Dandona, José das Neves, Dragos V Davitoiu, Barbora de Courten, Louisa Degenhardt, Selina Deiparine, Robert P Dellavalle, Kebede Deribe, Amare Deribew, Don C Des Jarlais, Subhojit Dey, Samath D Dharmaratne, Huyen Phuc Do, E Ray Dorsey,

Tim R Driscoll, Manisha Dubey, Bruce Bartholow Duncan, Aman Yesuf Endries, Holly E Erskine, Setegn Eshetie, Babak Eshrati, Alireza Esteghamati, Kara Estep, Andre Faro, Mir Sohail Fazeli, Seyed-Mohammad Fereshtehnejad, João C Fernandes, Alize J Ferrari, Tesfaye Regassa Feyissa, Irina Filip, Florian Fischer, Abraham D Flaxman, Nataliya Foigt, Kyle J Foreman, Nancy Fullman, Thomas Fürst, João M M Furtado, Emmanuela Gakidou, Morsaleh Ganji, Alberto L Garcia-Basteiro, Teshome Gebre, Gebremedhin Berhe Gebregergs, Tsegaye Tewelde Gebrehiwot, Johanna M Geleijnse, Ayele Geleto, Hailay Abrha Gesesew, Peter W Gething, Alireza Ghajar, Katherine B Gibney, Richard F Gillum, Melkamu Dedefo Gishu, Philimon N Gona, Amador Goodridge, Sameer Vali Gopalani, Alessandra Carvalho Goulart, Harish Chander Gugnani, Rajeev Gupta, Tanush Gupta, Reyna A Gutiérrez, Nima Hafezi-Nejad, Gessessew Bugssa Hailu, Randah Ribhi Hamadeh, Alexis J Handal, Hilda L Harb, Josep Maria Haro, Rasmus Havmoeller, Simon I Hay, Ileana Beatriz Heredia-Pi, Nobuyuki Horita, H Dean Hosgood, Sorin Hostiuc, Peter J Hotez, Damian G Hoy, Guoqing Hu, Ehimario Uche Igumbor, Chad Ikeda, Kathryn H Jacobsen, Mihajlo B Jakovljevic, Sudha P Jayaraman, Panniyammakal Jeemon, Paul N Jensen, Vivekanand Jha, Denny John, Jost B Jonas, Mikk Jürisson, Rajendra Kadel, Ritul Kamal, André Karch, Seyed M Karimi, Chante Karimkhani, Amir Kasaeian, Norito Kawakami, Peter Njenga Keiyoro, Andre Pascal Pascal Kengne, Chandrasekharan Nair Kesavachandran, Yousef Saleh Khader, Ejaz Ahmad Khan, Young-Ho Khang, Abdullah T Khoja, Jagdish Khubchandani, Christian Kieling, Daniel Kim, Yun Jin Kim, Katarzyna A Kissimova-Skarbek, Niranjan Kissoon, Mika Kivimaki, Ann Kristin Knudsen, Yoshihiro Kokubo, Dhaval Kolte, Jacek A Kopec, Georgios A Kotsakis, Parvaiz A Koul, Ai Koyanagi, Kristopher J Krohn, Barthelemy Kuate Defo, G Anil Kumar, Hmwe H Kyu, Anton Carl Jonas Lager, Dharmesh Kumar Lal, Tea Lallukka, Van C Lansingh, Anders Larsson, Janet L Leasher, James Leigh, Janni Leung, Misgan Legesse Liben, Stephen S Lim, Giancarlo Logroscino, Alan D Lopez, Paulo A Lotufo, Rafael Lozano, Raimundas Lunevicius, Ronan A Lyons, Hassan Magdy Abd El Razek, Mohammed Magdy Abd El Razek, Marek Majdan, Reza Majdzadeh, Azeem Majeed, Abdullah A Mamun, Laurie B Marczak, Jose Martinez-Raga, Francisco Rogerlândio Martins-Melo, Mohsen Mazidi, Colm McAlinden, John J McGrath, Toni Meier, Kidanu Gebremariam Meles, Ziad A Memish, Walter Mendoza, Melkamu Merid Mengesha, Mubarek Abera Mengistie, Desalegn Tadese Mengistu, George A Mensah, Atte Meretoja, Tuomo J Meretoja, Haftay Berhane Mezgebe, Ted R Miller, Shiva Raj Mishra, Philip B Mitchell, Karzan Abdulmuhsin Mohammad, Alireza Mohammadi, Muktar Sano Kedir Mohammed, Shafiu Mohammed, Ali H Mokdad, Sarah K Mollenkopf, Lorenzo Monasta, Maziar Moradi-Lakeh, Paula Moraga, Shane D Morrison, Ulrich O Mueller, Kate Muller, Christopher J L Murray, Srinivas Murthy, Kamarul Imran Musa, Jean B Nachega, Mohsen Naghavi, Kovin S Naidoo, Dumessa Edessa Negasa, Ionut Negoi, Josephine Wanjiku Ngunjiri, Cuong Tat Nguyen, Quyen Le Nguyen, Trang Huyen Nguyen, Vuong Minh Nong, Ole F Norheim, Bo Norrving, Jean Jacques N Noubiap, Felix Akpojene Ogbo, Anselm Okoro, Olanrewaju Oladimeji, Andrew Toyin Olagunju, Tinuke Oluwasefunmi Olagunju, Helen E Olsen, Bolajoko Olubukunola Olusanya, Jacob Olusegun Olusanya, Eyal Oren, Alberto Ortiz, Richard H Osborne, Erika Ota, Mayowa O Owolabi, Mahesh PA, Rosana E Pacella, Basant Kumar Panda, Christina Papachristou, Mahboubeh Parsaeian, Scott B Patten, George C Patton, Neil Pearce, David M Pereira, Konrad Pesudovs, Carrie Beth Peterson, Michael Robert Phillips, MIchael A Piradov, Dietrich Plass, Richie G Poulton, Mostafa Qorbani, Amir Radfar, Anwar Rafay, Vafa Rahimi-Movaghar, Mahfuzar Rahman, Rajesh Kumar Rai, Salman Rawaf, Sarah E Ray, Robert C Reiner, Andre M N Renzaho, Serge Resnikoff, Antonio L Ribeiro, Hirbo Shore Roba, Stephen R Robinson, David Rojas-Rueda, Gholamreza Roshandel, Gregory A Roth, Dietrich Rothenbacher, Ambuj Roy, George Mugambage Ruhago, Mahdi Safdarian, Saeid Safiri, Rajesh Sagar, Ramesh Sahathevan, Joshua A Salomon, Abdallah M Samy, Juan Ramon Sanabria, Damian Santomauro, Itamar S Santos, João Vasco Vasco Santos, Milena M Santric Milicevic,

Maheswar Satpathy, Sonia Saxena, Maria Inês Schmidt, Ione J C Schneider, Aletta E Schutte, Daivd C Schwebel, Falk Schwendicke, Sadaf G Sepanlou, Masood Ali Shaikh, Sheikh Mohammed Shariful Islam, Jun She, Kenji Shibuya, Mika Shigematsu, Rahman Shiri, Inga Dora Sigfusdottir, Diego Augusto Santos Silva, Jasvinder A Singh, Dhirendra Narain Sinha, Eirini Skiadaresi, Vegard Skirbekk, Eugene Sobngwi, Chandrashekhar T Sreeramareddy, Jeffrey Stanaway, Dan J Stein, Caitlyn Steiner, Sabine Steinke, Mark Andrew Stokes, Muawiyyah Babale Sufiyan, Bryan L Sykes, Cassandra E I Szoeke, Santosh Kumar Tadakamadla, Arash Tehrani-Banihashemi, Belay Tesssema, Ornwipa Thamsuwan, Alice M Theadom, Amanda G Thrift, Marcello Tonelli, Roman Topor-Madry, Miguel Tortajada-Girbés, Jefferson Traebert, Bach Xuan Tran, Thomas Truelsen, Stefanos Tyrovolas, Kingsley Nnanna Ukwaja, Eduardo A Undurraga, Olalekan A Uthman, Job FM F M van Boven, Tommi Vasankari, Lennert J Veerman, Narayanaswamy Venketasubramanian, Vasiliy Victorovich Vlassov, Stein Emil Vollset, Theo Vos, Tolassa Wakayo, Yuan-Pang Wang, Elisabete Weiderpass, Robert G Weintraub, Ronny Westerman, Harvey A Whiteford, Tissa Wijeratne, Charles Shey Shey Wiysonge, Denis Xavier, Gelin Xu, Bereket Yakob, Yuichiro Yano, Hassen Hamid Yimam, Naohiro Yonemoto, Mustafa Z Younis, Zoubida Zaidi, Maysaa El Sayed Zaki, Zerihun Menlkalew Zenebe, Ben Zipkin, and Liesl Joanna Zuhlke.

Extracting, cleaning, or cataloging data; designing or coding figures and tables

Muktar Beshir Ahmed, Sneha Aiyar, Christine Allen, Megha Arora, Robert Battista, James Bennett, Jennifer Benson, Adugnaw Berhane, Donal Bisanzio, Dube Jara Boneya, Lemma Negesa Bulto Bulto, Daniel Casey, Chris Castle, Fiona J Charlson, Devasahayam Jesudas Christopher, Danny Colombara, Kadine Priscila Bender dos Santos, Michelle Echko, Sergey Petrovich Ermakov, Holly E Erskine, Alize J Ferrari, Joseph Frostad, Nancy Fullman, Ayele Geleto, Ellen Goldberg, Nathaniel J Henry, Howard J Hoffman, Damian G Hoy, Chad Ikeda, Catherine Johnson, Sarah Charlotte Johnson, Amir Kasaeian, Laura Kemmer, Soewarta Kosen, Kristopher J Krohn, Barthelemy Kuate Defo, Qing Lan, Janni Leung, Xiaofeng Liang, Angela Liu, Patrick Y Liu, Emilie Maddison, Reza Malekzadeh, Treh Manhertz, Mohsen Mazidi, Melkamu Merid Mengesha, Anoushka Millear, Shawn Minnig, Alireza Mohammadi, Sarah K Mollenkopf, Mark Moses, Cliff Mountjoy-Venning, Helen E Olsen, Kanyin Ong, Mahesh PA, Katherine Paulson, David M Pigott, Mostafa Qorbani, Zane Rankin, Sarah E Ray, Nikolas Reinig, Enrico Rubagotti, Abdallah M Samy, Damian Santomauro, Maheswar Satpathy, Peilin Shi, Diego Augusto Santos Silva, Erica Leigh Slepak, Jeffrey Stanaway, Bryan Strub, Michelle Subart, Patrick J Sur, Dillon Sylte, Mohammad Tavakkoli, Ornwipa Thamsuwan, Andrew M Theis, Katie E Thomas, Derrick Tsoi, Rachel Updike, Rachel Woodbrook, Maysaa El Sayed Zaki, Ben Zipkin, and Leo Zoeckler.

Managing the overall research enterprise

Tahiya Alam, Kelly Bienhoff, Blair Bumgarner, Kara Estep, Abraham D Flaxman, Emmanuela Gakidou, Peter W Gething, Simon I Hay, Peter J Hotez, Chantal Huynh, Laura Kemmer, Xiaofeng Liang, Stephen S Lim, Rafael Lozano, Laurie B Marczak, Kate Muller, Christopher J L Murray, Mohsen Naghavi, Carla Makhlouf Obermeyer, Helen E Olsen, Puja C Rao, Sarah E Ray, Gregory A Roth, Joseph Salama, Chloe Shields, Shreya Shirude, Caitlyn Steiner, and Theo Vos.

Did not provide authorship contribution information

Abdishakur M Abdulle, Isaac A Adedeji, Ruth W Kimokoti, and Myriam Tobollik.

Table of Contents

Appendix Tables & Figures 4

Section 1. GBD Overview 5

Section 1.1. Locations of the Analysis 5

Section 1.2. Time Period of the Analysis 5

Section 1.3. Statement of GATHER Compliance 5

Section 1.4. List of abbreviations 6

Section 1.5. GBD results overview 8

Section 1.6. Data input sources overview 8

Section 1.7. Funding Sources 8

Section 2. Estimation Process for DALYs/HALE 9

Section 2.1 Estimation for DALYs/HALE 9

Section 2.2 Socio-Demographic Index (SDI) Definitions & Method 10

Case definition 10

Modeling strategy 10

Section 2.3 Socio-Demographic Index (SDI) analysis & Epidemiological Transition 11

Section 3. Comparison of GBD 2016 to other global estimates ....

Section 4. GATHER Compliance Table 13

Section 5. Tables & Figures 16

Appendix Tables & Figures

Appendix Table 1. GATHER checklist of information that should be included in reports of global health estimates, with description of compliance and location of information

Appendix Table 2. Life expectancy at birth and HALE at birth and at age 65 by location for GBD years 1990, 2000, 2006 & 2016, both sexes, combined

Appendix Table 3. All-age DALYs counts for all causes and level 1 cause groups by location for 1990 , 2000, 2006 & 2016, both sexes combined

Appendix Table 4. All-age DALYs rates per 100,000 for all causes and level 1 cause groups by location for 1990, 2000, 2006 & 2016, both sexes combined

Appendix Table 5. GBD 2016 DALYs exemplars for 1990, 2006 & 2016 on the basis of SDI

Appendix Table 6. SDI groupings by location, based on 2016 values

Appendix Table 7. SDI values for all estimated GBD 2016 locations, 1980-2016

Appendix Table 8. GBD 2016 location hierarchy with levels

Appendix Table 9. Causes included in the 2016 Global Burden of Disease study

Appendix Figure 1. Estimation Process for DALYs and HALE

Appendix Figure 2a-f. Leading ten level 3 causes of age-specific DALYs for all estimated GBD 2016 age groups, for a.) global and b.) SDI quintiles

Appendix Figure 3a-f. Composition of age-specific DALYs by GBD level 2 causes for 23 GBD age groups, 1990 vs. 2016, both sexes combined, for a) global and b-f) SDI quintiles

Appendix Figure 4. Leading level 3 causes of all-age DALY counts by location for i) females and ii) males, separately, in 1990

Appendix Figure 5. Annualized rate of change in age-standardised DALY rates from 1990 to 2016 vs. global age-standardised DALY rates per 100,000 in 2016 due for GBD level 3 causes

Appendix Figure 6. Ratio of global all–age DALY counts from NCDs to CMNN diseases by GBD locations and grouped by SDI quintile, for males and females separately, in 1990, 1995, 2000, 2006, 2010, and 2016.

Section 1. GBD Overview

Section 1.1. Locations of the Analysis

The GBD 2016 locations are arranged into a set of hierarchical categories composed of seven super-regions and 21 regions containing 195 countries and territories. The locations for which GBD estimated global, regional, and national cause-specific mortality and years of life lost (YLLs) have not expanded following GBD 2015. New sub-national locations estimated for GBD 2016 are the local government authorities of England and provinces of Indonesia. Subnational assessments for GBD 2016 include: 26 states and one federal district for Brazil, 33 provinces and municipalities for China, nine regions and 150 local government authorities (LGAs) for England, 31 states and union territories by urbanicity for India, 34 provinces for Indonesia, 47 prefectures for Japan, 47 counties for Kenya, 31 states and one federal district for Mexico, two areas for Sweden, nine provinces for South Africa, 13 states for the Kingdom of Saudi Arabia, and 50 states and one federal district for the United States. Combined, there are a total of 335 locations at the first subnational unit level. Included in subnational Level 1 locations are countries that have are subdivided into the first subnational level, such as states or provinces, for the GBD analysis; subnational Level 2 only applies to India and England. For this paper, we present data at the national and territory level. For more detailed information on the cause list with levels, please refer to the Cause of Death and Non-fatal Capstone Appendices.

Section 1.2. Time Period of the Analysis

A complete set of cause-specific mortality and years of life lost (YLL) numbers and rates were computed for the years 1980-2016. A set of non-fatal burden and years lived with disability (YLDs) numbers and rates were computed for 1990-2016.

We calculated DALYs as the sum of YLLs and YLDs for each cause, location, age group, sex, and year from 1990 to 2016. The same estimates of YLDs per capita for each location-age-sex-year from 1990 to 2016 are used to determine HALE by age group within abridged multiple-decrement life tables.

Section 1.3. Statement of GATHER Compliance

This study complies with the Guidelines for Accurate and Transparent Health Estimates Reporting (GATHER) recommendations. We have documented the steps involved in our analytical procedures and detailed the data sources used in compliance with the Guidelines for Accurate and Transparent Health Estimates Reporting (GATHER). See Appendix Table 1 for GATHER checklist.

The GATHER recommendations may be found here: http://gather-statement.org/

Section 1.4. List of abbreviations

Phrase	Acronym
Annualised Rate Of Change	ARC
Antenatal Care	ANC
Antiretroviral Therapy	ART
Basic Tabulation List	BTL
Body-mass index	BMI
Cancer Registry	CR
Case-Detection Rates	CDRs
Cause-Specific Mortality Rate	CSMR
Causes Of Death	COD
Causes-Of-Death Ensemble Modelling	CODEm
Centers for Disease Control & Prevention	CDC
Civil Registration System	CRS
Cluster Of Differentiation 4	CD4
Comparative Risk Assessment	CRA
Complete Birth Histories	CBH
Crude Death Rate	CDR
Data Representativeness Index	DRI
Demographic and Health Survey	DHS
Disability-Adjusted Life Year	DALY
Disease Model-Bayesian Meta-regression	DisMod-MR
Disease Surveillance Points	DSP
Emergency Obstetric Care	EmOC
Enzyme-Linked Immunosorbent Assay	ELISA
Epidemiology	Epi
Estimation and Projection Package	EPP
European Commission	EC
Fasting Plasma Glucose	FPG
Food Frequency Questionnaires	FFQs
Global Burden of Disease	GBD
Global Enteric Multicentre Study	GEMS
Guidelines for Accurate and Transparent Health Estimates Reporting	GATHER
Healthy Life Expectancy	HALE
Heart Outcomes Prevention Evaluation–3	HOPE-3
Human Development Index	HDI
Institute for Health Metrics and Evaluation	IHME
Integrated Exposure Response	IER
Inter-agency Group for Child Mortality Estimation	IGME
International Agency For Research On Cancer	IARC
International Classification of Diseases	ICD

International Diabetes Federation	IDF
Lagged Distributed Income	LDI
Low-Density Lipoprotein Cholesterol	LDL cholesterol
Low-Income And Middle-Income Countries	LMICs
Maternal Mortality	MM
Maternal Mortality Estimation Inter-Agency Group	MMEIG
Maternal Mortality Ratio	MMR
Maternal Mortality Surveillance	MMS
Medical Certification of Causes of Death	MCCD
Medical Expenditure Panel Surveys	MEPS
Millennium Development Goals	MDGs
Mortality/Incidence Ratio	MIR
Non-communicable Diseases	NCDs
Organisation for Economic Co-operation and Development	OECD
Polyunsaturated Fatty Acids	PUFAs
Pooled Resource Open-<br>Access ALS Clinical Trials	PROACT
Population-attributable Fraction	PAF
Prevention Of Mother-To-Child Transmission	PMTCT
Probability Of Death From Age 15 To 60 Years	45q15
Probability Of Death From Birth To Age 5 Years	5q0
Prospective Urban Rural Epidemiology	PURE
Quantitative Polymerase Chain Reaction Diagnostic	qPCR
Relative Risk	RR
Root Mean Square Error	RMSE
Sample Registration System	SRS
Short Form 12 questions	SF-12
Socio-demographic Index	SDI
Spatiotemporal Gaussian Process Regression	ST-GPR
Standardised Mortality Ratio	SMR
Stillbirth Epidemiology Investigator Group	SEIG
Summary Birth History	SBH
Summary Exposure Value	SEV
Super-Region Median Average Deviation	SR MAD
Surveillance, Epidemiology, and End Results Program	SEER
Survey of Causes of Death	SCD
Sustainable Development Goals	SDGs
Systolic Blood Pressure Intervention Trial	SPRINT
TaqMan Array Card	TAC
The Joint United Nations Programme on HIV and AIDS	UNAIDS
The United Nations Children's Fund	UNICEF
Theoretical Minimum Risk Level	TMREL
Uncertainty Intervals	UIs

Verbal Autopsy	VA
Vital Registration	VR
World Malaria Report	WMR
World Population Prospects	WPP
Years Lived with Disability	YLDs
Years of Life Lost	YLLs

Section 1.5. GBD results overview

Results from GBD 2016 are now measured in terabytes. Results are available in an interactive data downloading tool available in the Global Health Data exchange (GHDx). The tool contains the complete set of results from all summary papers; however, specialized tables from the papers are available as separate entries in the GHDx, as were made available for GBD 2015.

In the GBD 2016 version of the GHDx, the tool also contains measures such as prevalence and incidence as well as rate of change data. Data above a certain size cannot be viewed online but can be downloaded. Depending on the size of the download, users need to enter an email address and a download location will be sent to them when the files are prepared.

Section 1.6. Data input sources overview

GBD 2016 incorporated a large number and wide variety of input sources to estimate mortality, causes of death and illness, and risk factors for 195 countries and territories from 1990-2016. These input sources are accessible through an interactive citation tool available in IHME's GHDx.

Users can retrieve citations for a specific GBD component, cause or risk, and location by choosing from the available selection boxes. They can then view and access GHDx records for input sources and export a CSV file that includes the GHDx metadata, citations, and information about where the data were used in GBD. Additional metadata for each input source are available through the citation tool, as required by the GATHER statement.

The citation tool is accessible through the GHDx at http://ghdx.healthdata.org/gbd-2016/data-input-sources-restricted. Password: worldwideburdenestimates

Section 1.7. Funding Sources

Funding for this research was provided by the Bill & Melinda Gates Foundation.

Section 2. Estimation Process for DALYs/HALE

Section 2.1 Computing DALYs

In order to estimate DALYs for GBD 2016, we first estimated cause-specific mortality and non-fatal health loss. For each year for which YLDs have been estimated (1990, 1995, 2000, 2006, 2010 and 2016), we compute DALYs by adding YLLs and YLDs for each age-sex-country group. We assume uncertainty in YLLs is independent of uncertainty in YLDs. This approach is operationalised by summing the first draw of the 1000 draws for YLLs and YLDs and then repeating for each subsequent draw. 95% uncertainty intervals (UI) are computed using the 25th and 975th ordered draw of the DALY uncertainty distribution. Please refer to the appendices of the GBD 2016 non-fatal capstone and cause of death capstone publications. We calculate DALYs as the sum of YLLs and YLDs for each cause, location, age group, sex, and year. For more information, please refer to Appendix Figure 1 below.

Appendix Figure 1. Estimation for DALYs & HALE for GBD 2016

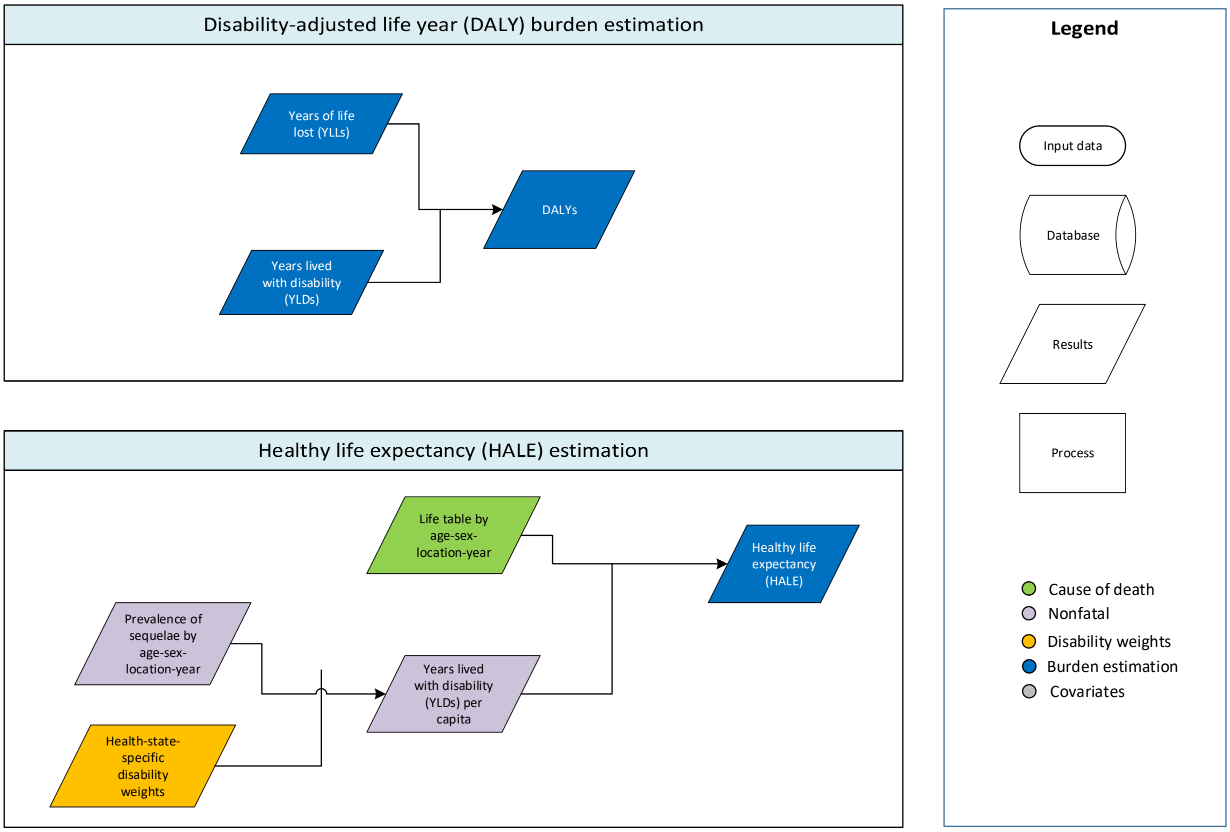

Section 2.2 Computing HALE

We compute HALE following the methods outlined in the main text of the paper, which are also outlined here. In brief, Sullivan's method is used to incorporate information about average levels of health experienced at different ages into an abridged life table to produce estimates of life expectancy that are adjusted for reductions in functional health status relating to prevalent health conditions. Effectively the cumulative years lived in an age-group in the abridged life table (the life expectancy column) for each country-age-sex group is multiplied by the YLDs per capita for that country-age-sex group. Calculation of HALE relies on three inputs from GBD 2016: life tables by sex, country and year; estimates of the prevalence of sequelae by age, sex, country and year; and disability weights for all unique health states that collectively cover the range of functional health losses and symptoms associated with each sequelae. Data sources and methods for estimating mortality and life tables are described in the mortality capstone of this issue; data sources and approaches to measuring prevalence of sequelae and disability weights are described in the nonfatal capstone appendix of this issue. Information on prevalence and disability weights are combined into measures of the overall rate of YLDs per capita in each age-sex-country group. We make the strong assumption that uncertainty in YLDs per capita is independent of uncertainty in age-specific death rates to compute uncertainty distributions in HALE.

Section 2.3 Socio-Demographic Index (SDI) Definitions & Method

Case definition

The Socio-demographic Index (SDI) is a composite indicator of development status used in the GBD whose components are correlated with health outcomes. It is the geometric mean of 0 to 1 indices of total fertility rate, mean education for those aged 15 and older, and lag distributed income per capita.

Modeling strategy

SDI was calculated using the Human Development Index (HDI) methodology, wherein an index value was determined for each of the covariate inputs (log LDI, mean educational attainment over age 15, and TFR). For GBD 2016 these indices were computed on the basis of a relative scale, in which the upper and lower bounds were established by the maximum and minimum observed values, respectively, for each input over the entire estimation period of 1980-2016.

Prompted by the observations that the scales (and by extension SDI) were sensitive to the addition of new subnational locations as GBD becomes more granular and to the length of the time period over which SDI is computed, for GBD 2016 we implemented fixed scales in determining individual indices. Thus, an index score of 0 now represents the minimum level of each covariate input past which health outcomes can get no worse. An index score of 1 represents the maximum level of each covariate input past which health outcomes cease to improve. As a composite, a location with an SDI of 0 would have a theoretical minimum level of development relevant to health, while a location with an SDI of 1 would have a theoretical maximum level of development relevant to health.

We selected the minima and maxima of the scales by examining the relationships each of the inputs had with life expectancy at birth and under-5 mortality and identifying points of limiting returns at both high and low values, if they occurred prior to theoretical limits (e.g., a TFR of 0). The final scales are summarized in the table below.

Input	Lower Bound	Upper Bound
TFR	1.5[a]	8
LDI per capita	250 USD (5.52 log USD)[b]	60,000 USD (11.00 log USD)
Mean educational attainment for ages 15 and older	0 years	17 years

[a] The low point of limiting returns for TFR was identified at 1 during GBD 2015; however, incorporating feedback with regard to accounting for a pattern of TFR rebound in highly developed countries, we instead set the lower limit of TFR at 1.5.
[b] The minimum for the LDI scale was originally set at the theoretical limit of 0 USD, as we did not observe an asymptotic relationship between log(LDI) and E_0 or 5q0 at lower values of log(LDI). Empirically, however, we also did not observe an LDI below 350 USD (5.86 log USD) for the estimation period 1970-2016. In log-space, this meant that approximately half of our scale was not being utilized, compressing the observed variation in LDI and diminishing its meaningful contribution to SDI. Accordingly, we set the lower limit on LDI to 250 USD (5.52 log USD) to ensure we were fully utilizing the range of the scale to capture its variation across space and time, as is the case with the other two inputs.

Using the limits on the scales described above, we computed the index scores underlying SDI analogously to GBD 2016 as follows:

$$I_{Cly} = {(C_{ly} - C_{low})}\Big/{(C_{high} - C_{low})}$$

Where I_{cly} – the index for covariate *C*, location *l*, and year *y* – is equal to the difference between the value of that covariate in that location-year and the lower bound of the covariate divided by the difference between the upper and lower bounds for that covariate. If the values of input covariates fell outside the upper or lower bounds (e.g. LDI per capita greater than 60,000 USD), they were mapped to the respective upper or lower bounds. We also note that the index value for TFR was computed as $1 - I_{TFRly}$, as lower TFRs correspond to higher levels of development, and thus higher index scores. For GBD 2016 we expanded the computation of SDI to 755 national and subnational locations spanning the time period 1970-2016.

The composite Socio-Demographic Index is the geometric mean of these three indices for a given location-year. The cutoff values used to determine quintiles for analysis were then computed using country-level estimates of SDI for the year 2016, excluding countries with populations less than 1 million. More information on SDI values can be found in the Cause of Death and Non-fatal Appendices.

Section 2.3 Socio-Demographic Index (SDI) analysis & Epidemiological Transition

We derived expected DALYs on the basis of SDI using the separate YLL and YLD models described in the GBD 2016 cause of death and non-fatal papers.[1] Additionally, HALE was calculated using the same methods described in both the main text of the paper and earlier in the appendix, with expected YLD rates and expected life tables as inputs. Please see Appendix Table 5 for the ratio of observed to expected DALYs for GBD 2016.

Section 3. Comparison of GBD 2016 to other global estimates

The GBD study is the only source of comprehensive quantification of population health summary measures, including YLLs, YLDs, DALYs and HALE. There are specific efforts to estimate burden within other organisations which are relevant to policy makers. In the following sections we explore how some of the major sources of DALYs and HALE compare with other global estimates. Since GBD 2015, most organisations we evaluated have not produced updated estimates for DALYs or HALE. The exception to this is WHO, which has released updated Global Health Estimates for DALYs and HALE for 183 countries from 2000 to 2015. These estimates draw heavily on the GBD 2015 results with revisions to the all-cause mortality envelope and revisions to selected cause-specific disability weights and severity distributions for YLDs. Please see the Non-fatal Capstone Appendix for more information.

Section 4. GATHER Compliance Table

Table 1. GATHER checklist of information that should be included in reports of global health estimates, with description of compliance and location of information for GBD 2016 DALYs and HALE capstone

#	GATHER checklist item	Description of compliance	Reference
**Objectives and funding**			
1	Define the indicators, populations, and time periods for which estimates were made.	Narrative provided in paper describing indicators, definitions, time periods, and populations.	Main text (Introduction, Methods)
2	List the funding sources for the work.	Funding sources listed in paper.	Main text (Summary, Funding)
**Data Inputs**			
For all data inputs from multiple sources that are synthesized as part of the study:			
3	Describe how the data were identified and how the data were accessed.	All data seeking methodology referenced in main text.	1. Main text (Methods)<br>2. Nonfatal & Cause of Death Appendices
4	Specify the inclusion and exclusion criteria. Identify all ad-hoc exclusions.	All inclusion and exclusion criteria by data type referenced in main text.	1. Main text (Methods)<br>2. Appendix (Methods)
5	Provide information on all included data sources and their main characteristics. For each data source used, report reference information or contact name/institution, population represented, data collection method, year(s) of data collection, sex and age range, diagnostic criteria or measurement method, and sample size, as relevant.	Interactive, online data source tool that provides metadata for data sources by component, geography, cause, or impairment has been developed.	Online data tools (live with GBD 2016 at publication) http://ghdx.healthdata.org/gbd-2016-data-citations
6	Identify and describe any categories of input data that have potentially important biases (e.g., based on characteristics listed in item 5).	Summary of known biases by cause included in methodological appendices of referenced papers.	1. Main text (Methods)<br>2. Appendix<br>3. Cause of Death & Nonfatal Appendices

For data inputs that contribute to the analysis but were not synthesized as part of the study:			
7	Describe and give sources for any other data inputs.	Included in list of all data sources provided in submission materials for referenced papers as well as online data source tool.	Online data tools (to go live with GBD 2016 at publication) http://ghdx.healthdata.org/gbd-2016-data-citations
For all data inputs:			
8	Provide all data inputs in a file format from which data can be efficiently extracted (e.g., a spreadsheet as opposed to a PDF), including all relevant meta-data listed in item 5. For any data inputs that cannot be shared due to ethical or legal reasons, such as third-party ownership, provide a contact name or the name of the institution that retains the right to the data.	Downloads of input data will be available through online tools, including data visualization tools and data query tools. Input data not available in tools will be made available upon request.	Online data tools (to go live with GBD 2016 at publication) http://www.healthdata.org/results/data-visualizations; http://ghdx.healthdata.org/; http://ghdx.healthdata.org/gbd-data-tool
**Data analysis**			
9	Provide a conceptual overview of the data analysis method. A diagram may be helpful.	Flow diagrams of the overall methodological processes as well as cause-specific modelling processes have been provided.	Main text (Methods)
10	Provide a detailed description of all steps of the analysis, including mathematical formulae. This description should cover, as relevant, data cleaning, data pre-processing, data adjustments and weighting of data sources, and mathematical or statistical model(s).	Methodological write-up for the calculation of DALYs and HALE have been provided.	Main text (Methods)
11	Describe how candidate models were evaluated and how the final model(s) were selected.	Provided in the methodological write-ups within referenced appendix material.	1. Main text (Methods)<br>2. Cause of Death and Nonfatal Appendices (see citations in main text)
12	Provide the results of an evaluation of model performance, if done, as well as the results of any relevant sensitivity analysis.	Provided in the methodological write-ups within referenced appendix material.	1. Main text (Methods)<br>2. Cause of Death and Nonfatal Appendices
13	Describe methods for calculating uncertainty of the estimates. State which sources of uncertainty were, and were not, accounted for in the uncertainty analysis.	Provided in the methodological write-ups within referenced appendix material.	1. Main text (Methods)<br>2. Cause of Death and Nonfatal Appendices
14	State how analytic or statistical source code used to generate estimates can be accessed.	Access statement provided.	Git hub link for final submission, forthcoming
**Results and Discussion**			

15	Provide published estimates in a file format from which data can be efficiently extracted.	GBD 2015 results will be made available through online data visualization tools, the Global Health Data Exchange, and the online data query tool (these tools are already available for GBD 2013 results).	Main text, appendix supplementary results, and online data tools (to go live with GBD 2016 at publication) http://www.healthdata.org/results/data-visualizations; http://ghdx.healthdata.org/; http://ghdx.healthdata.org/gbd-data-tool
16	Report a quantitative measure of the uncertainty of the estimates (e.g. uncertainty intervals).	Uncertainty intervals are provided with all results.	Main text, appendix supplementary results, and online data tools (to go live with GBD 2016 at publication) http://www.healthdata.org/results/data-visualizations; http://ghdx.healthdata.org/; http://ghdx.healthdata.org/gbd-data-tool
17	Interpret results in light of existing evidence. If updating a previous set of estimates, describe the reasons for changes in estimates.	Discussion of methodological changes between GBD rounds provided in the narrative of the papers and appendices of referenced papers.	1. Main text (Methods)<br>2. Appendix (Methods)
18	Discuss limitations of the estimates. Include a discussion of any modelling assumptions or data limitations that affect interpretation of the estimates.	Discussion of limitations provided in the narrative of the main paper as well as in the methodological write-ups in the appendices of referenced papers.	Main text (Methods, Limitations)

Section 5. Tables & Figures

To download the data in the tables and figures, please visit the Global Health Data Exchange (GHDx) at: http://ghdx.healthdata.org/node/311214

Appendix Figure 2a: Leading ten level 3 causes of global age-specific DALYs colored by percentage change from 1990 to 2016 for GBD 2016

	1	2	3	4	5	6	7	8	9	10
**Early Neonatal**	NN Preterm	NN Enceph	Oth NN	Congenital	NN Sepsis	LRI	NN Hemol	STD	Diarrhea	Meningitis
**Late Neonatal**	NN Sepsis	NN Preterm	LRI	Congenital	Oth NN	NN Enceph	Diarrhea	Malaria	NN Hemol	STD
**Post Neonatal**	LRI	Diarrhea	Malaria	Congenital	PEM	Meningitis	HIV	STD	Whooping	NN Preterm
**1 to 4**	Malaria	Diarrhea	LRI	PEM	Congenital	Iron	Meningitis	Skin	Drown	Measles
**5 to 9**	Iron	Skin	Malaria	Diarrhea	Intest Inf	LRI	Road Inj	Congenital	Asthma	Drown
**10 to 14**	Skin	Iron	Conduct	Migraine	HIV	Road Inj	Anxiety	Malaria	Intest Inf	Diarrhea
**15 to 19**	Skin	Road Inj	Migraine	Self Harm	HIV	Depression	Violence	Iron	Anxiety	Back+Neck
**20 to 24**	Road Inj	Skin	Self Harm	Migraine	Violence	Back+Neck	Depression	HIV	Drugs	TB
**25 to 29**	Road Inj	HIV	Back+Neck	Self Harm	Migraine	Skin	Drugs	Depression	Violence	TB
**30 to 34**	HIV	Road Inj	Back+Neck	Migraine	Self Harm	IHD	TB	Depression	Skin	Drugs
**35 to 39**	HIV	Back+Neck	Road Inj	IHD	Migraine	TB	Depression	Self Harm	Stroke	Skin
**40 to 44**	Back+Neck	IHD	HIV	Road Inj	Migraine	Stroke	Depression	TB	Diabetes	Sense
**45 to 49**	IHD	Back+Neck	Stroke	Road Inj	Diabetes	HIV	Sense	Migraine	Depression	TB
**50 to 54**	IHD	Stroke	Back+Neck	Diabetes	Sense	Road Inj	COPD	Lung C	TB	Depression
**55 to 59**	IHD	Stroke	Back+Neck	Diabetes	COPD	Sense	Lung C	TB	Road Inj	CKD
**60 to 64**	IHD	Stroke	COPD	Diabetes	Back+Neck	Sense	Lung C	CKD	Liver C	Depression
**65 to 69**	IHD	Stroke	COPD	Sense	Diabetes	Lung C	Back+Neck	CKD	LRI	Stomach C
**70 to 74**	IHD	Stroke	COPD	Sense	Diabetes	Lung C	Back+Neck	Alzheimer	LRI	CKD
**75 to 79**	IHD	Stroke	COPD	Sense	Alzheimer	Diabetes	Back+Neck	Lung C	LRI	CKD
**80 to 84**	IHD	Stroke	Alzheimer	COPD	Sense	Diabetes	LRI	Back+Neck	CKD	Diarrhea
**85 to 89**	IHD	Alzheimer	Stroke	COPD	Sense	LRI	Diabetes	Falls	CKD	Back+Neck
**90 to 94**	IHD	Alzheimer	Stroke	COPD	Sense	LRI	Falls	CKD	Diabetes	HTN HD
**95 plus**	IHD	Alzheimer	Stroke	LRI	COPD	Sense	Falls	CKD	HTN HD	Diabetes

Percent change in age-specific DALYs

-0.92 to -0.44	-0.44 to -0.27	-0.27 to 0.05	0.05 to 0.21	0.21 to 0.37	0.37 to 0.55	0.55 to 0.85	0.85 to 1.09	1.09 to 1.82	1.82 to 69.45

IHD=ischaemic heart disease. Back & neck=low back and neck pain. Diabetes=diabetes mellitus. Stroke=Cerebrovascular disease. Lung C=lung, bronchus, and trachea cancers. Sense=sense organ diseases. Depression=depressive disorders. Alzheimer's=Alzheimer's disease and other dementias. Oth MSK=other musculoskeletal disorders. COPD=chronic obstructive pulmonary disease. NN Preterm=neonatal preterm birth complications. Diarrhoea=diarrhoeal diseases. Skin=skin and subcutaneous diseases. NN Enceph=neonatal encephalopathy due to birth asphyxia and trauma. Drugs=drug use disorders. Congenital=congenital anomalies. Liver C=liver cancer. Stomach C=stomach cancer. CKD=chronic kidney disease. Anxiety=anxiety disorders. Iron=iron-deficiency anaemia. HIV=HIV/AIDS. Colorect C=colon and rectum cancer. LRI=lower respiratory infections. Cirr HepC=cirrhosis due to hepatitis C. CMP=cardiomyopathy and myocarditis. Cirr alc=cirrhosis due to alcohol use. Other Cardio=other cardiovascular and circulatory diseases. Alcohol=alcohol use disorders. Violence=interpersonal violence. HTN HD=hypertensive heart disease. Haemog=haemoglobinopathies and haemolytic anaemias. TB=tuberculosis. Prostate C=prostate cancer. War=collective violence and legal intervention . Other Unint= other unintentional injuries. Oth trans=other transport injuries. Other NN=Other neonatal disorders. NN Sepsis=neonatal sepsis and other neonatal infections. PEM=protein-energy malnutrition. STD=sexually transmitted diseases excluding HIV. Ebola=Ebola virus disease. Other NTD=other neglected tropical diseases. GBD=Global Burden of Disease. SDI=Socio-demographic Index. DALYs=disability-adjusted life-years.

Appendix Figure 2b: Leading ten level 3 causes of age-specific DALYs colored by percentage change from 1990 to 2016 in Low SDI for GBD 2016

	1	2	3	4	5	6	7	8	9	10
**Early Neonatal**	NN Enceph	NN Preterm	Oth NN	NN Sepsis	Congenital	LRI	STD	NN Hemol	Diarrhea	Meningitis
**Late Neonatal**	NN Sepsis	LRI	NN Preterm	Oth NN	Congenital	NN Enceph	Diarrhea	Malaria	STD	Meningitis
**Post Neonatal**	LRI	Diarrhea	Malaria	PEM	Congenital	Meningitis	STD	Whooping	HIV	NN Sepsis
**1 to 4**	Malaria	Diarrhea	LRI	PEM	Meningitis	Whooping	Congenital	Measles	Iron	STD
**5 to 9**	Malaria	Iron	Diarrhea	HIV	Skin	LRI	Meningitis	Road Inj	Asthma	Congenital
**10 to 14**	HIV	Skin	Malaria	Iron	Diarrhea	Meningitis	LRI	Road Inj	Conduct	Migraine
**15 to 19**	HIV	Skin	Road Inj	Diarrhea	TB	Depression	Iron	Malaria	Migraine	Meningitis
**20 to 24**	HIV	Road Inj	TB	Diarrhea	Skin	Violence	Depression	Malaria	Migraine	Iron
**25 to 29**	HIV	TB	Road Inj	Diarrhea	Back+Neck	Violence	Migraine	Depression	LRI	Skin
**30 to 34**	HIV	TB	Road Inj	Diarrhea	Back+Neck	Migraine	Depression	LRI	IHD	Violence
**35 to 39**	HIV	TB	Back+Neck	Diarrhea	Road Inj	IHD	LRI	Migraine	Depression	Stroke
**40 to 44**	HIV	TB	IHD	Back+Neck	Diarrhea	Stroke	Road Inj	LRI	Depression	Sense
**45 to 49**	HIV	TB	IHD	Stroke	Back+Neck	Diarrhea	LRI	Diabetes	Sense	Road Inj
**50 to 54**	IHD	TB	Stroke	HIV	Back+Neck	Diarrhea	Diabetes	LRI	Sense	COPD
**55 to 59**	IHD	TB	Stroke	Diarrhea	Diabetes	LRI	Back+Neck	COPD	Sense	HIV
**60 to 64**	IHD	Stroke	TB	Diarrhea	COPD	LRI	Diabetes	Sense	Back+Neck	CKD
**65 to 69**	IHD	Stroke	Diarrhea	TB	COPD	LRI	Diabetes	Sense	Back+Neck	CKD
**70 to 74**	IHD	Stroke	Diarrhea	LRI	COPD	TB	Diabetes	Sense	Back+Neck	Oth Cardio
**75 to 79**	IHD	Stroke	Diarrhea	LRI	COPD	TB	Sense	Diabetes	Alzheimer	Oth Cardio
**80 to 84**	IHD	Stroke	Diarrhea	LRI	COPD	Alzheimer	Sense	TB	Diabetes	Oth Cardio
**85 to 89**	IHD	Diarrhea	Stroke	Alzheimer	LRI	COPD	Sense	TB	Diabetes	Oth Cardio
**90 to 94**	IHD	Diarrhea	Alzheimer	Stroke	LRI	Sense	COPD	Falls	Diabetes	TB
**95 plus**	IHD	Diarrhea	Alzheimer	LRI	Stroke	Sense	COPD	Falls	TB	Oth Cardio

Percent change in age-specific DALYs

-0.91 to -0.05	-0.05 to 0.19	0.19 to 0.42	0.42 to 0.61	0.61 to 0.81	0.81 to 1	1 to 1.15	1.15 to 1.27	1.27 to 1.74	1.74 to 136.71

IHD=ischaemic heart disease. Back & neck=low back and neck pain. Diabetes=diabetes mellitus. Stroke=Cerebrovascular disease. Lung C=lung, bronchus, and trachea cancers. Sense=sense organ diseases. Depression=depressive disorders. Alzheimer's=Alzheimer's disease and other dementias. Oth MSK=other musculoskeletal disorders. COPD=chronic obstructive pulmonary disease. NN Preterm=neonatal preterm birth complications. Diarrhoea=diarrhoeal diseases. Skin=skin and subcutaneous diseases. NN Enceph=neonatal encephalopathy due to birth asphyxia and trauma. Drugs=drug use disorders. Congenital=congenital anomalies. Liver C=liver cancer. Stomach C=stomach cancer. CKD=chronic kidney disease. Anxiety=anxiety disorders. Iron=iron-deficiency anaemia. HIV=HIV/AIDS. Colorect C=colon and rectum cancer. LRI=lower respiratory infections. Cirr HepC=cirrhosis due to hepatitis C. CMP=cardiomyopathy and myocarditis. Cirr alc=cirrhosis due to alcohol use. Other Cardio=other cardiovascular and circulatory diseases. Alcohol=alcohol use disorders. Violence=interpersonal violence. HTN HD=hypertensive heart disease. Haemog=haemoglobinopathies and haemolytic anaemias. TB=tuberculosis. Prostate C=prostate cancer. War=collective violence and legal intervention . Other Unint= other unintentional injuries. Oth trans=other transport injuries. Other NN=Other neonatal disorders. NN Sepsis=neonatal sepsis and other neonatal infections. PEM=protein-energy malnutrition. STD=sexually transmitted diseases excluding HIV. Ebola=Ebola virus disease. Other NTD=other neglected tropical diseases. GBD=Global Burden of Disease. SDI=Socio-demographic Index. DALYs=disability-adjusted life-years.

Appendix Figure 2c: Leading ten level 3 causes of age-specific DALYs colored by percentage change from 1990 to 2016 in Low-Middle SDI for GBD 2016

	1	2	3	4	5	6	7	8	9	10
**Early Neonatal**	NN Enceph	NN Preterm	Oth NN	Congenital	NN Sepsis	LRI	NN Hemol	STD	Diarrhea	Tetanus
**Late Neonatal**	NN Sepsis	NN Preterm	LRI	Oth NN	NN Enceph	Congenital	Diarrhea	NN Hemol	Malaria	Meningitis
**Post Neonatal**	LRI	Diarrhea	Malaria	Congenital	PEM	Meningitis	Whooping	HIV	STD	NN Preterm
**1 to 4**	Malaria	Diarrhea	LRI	PEM	Iron	Congenital	Meningitis	Measles	Drown	Skin
**5 to 9**	Iron	Intest Inf	Malaria	Skin	Diarrhea	LRI	Congenital	Road Inj	Drown	HIV
**10 to 14**	Skin	Iron	Intest Inf	Migraine	HIV	Malaria	Conduct	Diarrhea	Road Inj	Anxiety
**15 to 19**	Skin	Road Inj	Self Harm	Iron	Migraine	HIV	Depression	Diarrhea	Anxiety	Intest Inf
**20 to 24**	Road Inj	Self Harm	Skin	Migraine	Iron	HIV	Depression	TB	Back+Neck	Violence
**25 to 29**	HIV	Road Inj	Self Harm	Migraine	TB	Back+Neck	Depression	Skin	Iron	Oth MSK
**30 to 34**	HIV	Road Inj	TB	Migraine	Back+Neck	IHD	Self Harm	Depression	Iron	Oth MSK
**35 to 39**	HIV	IHD	Road Inj	TB	Back+Neck	Migraine	Depression	Self Harm	Sense	Stroke
**40 to 44**	IHD	HIV	Back+Neck	TB	Road Inj	Stroke	Migraine	Depression	Sense	Diabetes
**45 to 49**	IHD	Back+Neck	Stroke	TB	HIV	Road Inj	Sense	Diabetes	COPD	Migraine
**50 to 54**	IHD	Stroke	Back+Neck	COPD	TB	Diabetes	Sense	Road Inj	Diarrhea	CKD
**55 to 59**	IHD	Stroke	COPD	TB	Diabetes	Sense	Back+Neck	Diarrhea	CKD	Road Inj
**60 to 64**	IHD	Stroke	COPD	Diabetes	Sense	TB	Diarrhea	Back+Neck	LRI	CKD
**65 to 69**	IHD	Stroke	COPD	Sense	Diabetes	Diarrhea	TB	LRI	Back+Neck	CKD
**70 to 74**	IHD	COPD	Stroke	Diarrhea	Sense	Diabetes	LRI	TB	CKD	Back+Neck
**75 to 79**	IHD	COPD	Stroke	Diarrhea	Sense	Diabetes	LRI	Alzheimer	TB	Falls
**80 to 84**	IHD	COPD	Stroke	Diarrhea	Sense	Diabetes	Alzheimer	LRI	TB	Falls
**85 to 89**	IHD	COPD	Diarrhea	Stroke	Alzheimer	Sense	LRI	Diabetes	Falls	CKD
**90 to 94**	IHD	Diarrhea	Alzheimer	COPD	Stroke	Sense	LRI	Diabetes	Falls	CKD
**95 plus**	IHD	Diarrhea	Alzheimer	Stroke	COPD	LRI	Sense	Falls	Diabetes	CKD

Percent change in age-specific DALYs

-0.93 to -0.48	-0.48 to -0.27	-0.27 to 0.06	0.06 to 0.44	0.44 to 0.58	0.58 to 0.83	0.83 to 0.97	0.97 to 1.6	1.6 to 2.59	2.59 to 251.78

IHD=ischaemic heart disease. Back & neck=low back and neck pain. Diabetes=diabetes mellitus. Stroke=Cerebrovascular disease. Lung C=lung, bronchus, and trachea cancers. Sense=sense organ diseases. Depression=depressive disorders. Alzheimer's=Alzheimer's disease and other dementias. Oth MSK=other musculoskeletal disorders. COPD=chronic obstructive pulmonary disease. NN Preterm=neonatal preterm birth complications. Diarrhoea=diarrhoeal diseases. Skin=skin and subcutaneous diseases. NN Enceph=neonatal encephalopathy due to birth asphyxia and trauma. Drugs=drug use disorders. Congenital=congenital anomalies. Liver C=liver cancer. Stomach C=stomach cancer. CKD=chronic kidney disease. Anxiety=anxiety disorders. Iron=iron-deficiency anaemia. HIV=HIV/AIDS. Colorect C=colon and rectum cancer. LRI=lower respiratory infections. Cirr HepC=cirrhosis due to hepatitis C. CMP=cardiomyopathy and myocarditis. Cirr alc=cirrhosis due to alcohol use. Other Cardio=other cardiovascular and circulatory diseases. Alcohol=alcohol use disorders. Violence=interpersonal violence. HTN HD=hypertensive heart disease. Haemog=haemoglobinopathies and haemolytic anaemias. TB=tuberculosis. Prostate C=prostate cancer. War=collective violence and legal intervention . Other Unint= other unintentional injuries. Oth trans=other transport injuries. Other NN=Other neonatal disorders. NN Sepsis=neonatal sepsis and other neonatal infections. PEM=protein-energy malnutrition. STD=sexually transmitted diseases excluding HIV. Ebola=Ebola virus disease. Other NTD=other neglected tropical diseases. GBD=Global Burden of Disease. SDI=Socio-demographic Index. DALYs=disability-adjusted life-years.

Appendix Figure 2d Leading ten level 3 causes of age-specific DALYs colored by percent change from 1990 to 2016 in Middle SDI for GBD 2016

	1	2	3	4	5	6	7	8	9	10
**Early Neonatal**	NN Preterm	NN Enceph	Congenital	Oth NN	NN Sepsis	LRI	NN Hemol	STD	Diarrhea	Oth Inf
**Late Neonatal**	NN Preterm	Congenital	NN Sepsis	LRI	Oth NN	NN Enceph	Diarrhea	NN Hemol	Meningitis	SIDS
**Post Neonatal**	LRI	Congenital	Diarrhea	HIV	NN Preterm	PEM	Meningitis	F Body	NN Enceph	Oth NN
**1 to 4**	LRI	Congenital	Diarrhea	Skin	Drown	PEM	Iron	Road Inj	HIV	Meningitis
**5 to 9**	Skin	Road Inj	Iron	Drown	Congenital	Asthma	LRI	Intest Inf	Diarrhea	Sense
**10 to 14**	Skin	Road Inj	Conduct	Drown	Migraine	Anxiety	Iron	Congenital	Asthma	Sense
**15 to 19**	Skin	Road Inj	Violence	Migraine	Self Harm	Depression	Anxiety	Back+Neck	Conduct	Drown
**20 to 24**	Road Inj	Violence	Skin	Self Harm	Migraine	Back+Neck	Depression	Drugs	Oth MSK	HIV
**25 to 29**	Road Inj	HIV	Violence	Back+Neck	Self Harm	Migraine	Skin	Depression	Drugs	Oth MSK
**30 to 34**	Road Inj	HIV	Back+Neck	Migraine	IHD	Violence	Self Harm	Skin	Oth MSK	Depression
**35 to 39**	Road Inj	HIV	Back+Neck	IHD	Migraine	Stroke	Depression	Diabetes	Violence	Oth MSK
**40 to 44**	Back+Neck	IHD	Road Inj	Stroke	HIV	Diabetes	Migraine	Sense	Depression	Oth MSK
**45 to 49**	IHD	Back+Neck	Stroke	Road Inj	Diabetes	Sense	HIV	Liver C	Depression	Migraine
**50 to 54**	IHD	Stroke	Back+Neck	Diabetes	Road Inj	Sense	Liver C	Lung C	COPD	CKD
**55 to 59**	IHD	Stroke	Diabetes	Back+Neck	Sense	Lung C	COPD	Liver C	Road Inj	CKD
**60 to 64**	IHD	Stroke	Diabetes	COPD	Lung C	Sense	Back+Neck	Liver C	CKD	Stomach C
**65 to 69**	IHD	Stroke	COPD	Diabetes	Sense	Lung C	Back+Neck	CKD	Liver C	Stomach C
**70 to 74**	IHD	Stroke	COPD	Sense	Diabetes	Lung C	Alzheimer	CKD	Back+Neck	Stomach C
**75 to 79**	IHD	Stroke	COPD	Sense	Alzheimer	Diabetes	Lung C	CKD	Back+Neck	HTN HD
**80 to 84**	IHD	Stroke	COPD	Alzheimer	Sense	Diabetes	HTN HD	LRI	CKD	Lung C
**85 to 89**	IHD	Stroke	Alzheimer	COPD	Sense	Diabetes	LRI	HTN HD	CKD	Falls
**90 to 94**	IHD	Alzheimer	Stroke	COPD	Sense	LRI	HTN HD	Diabetes	CKD	Falls
**95 plus**	IHD	Alzheimer	Stroke	COPD	Sense	LRI	HTN HD	CKD	Falls	Diabetes

Percent change in age-specific DALYs

-0.91 to -0.66	-0.66 to -0.34	-0.34 to -0.09	-0.09 to 0.15	0.15 to 0.4	0.4 to 0.91	0.91 to 1.14	1.14 to 1.43	1.43 to 2.7	2.7 to 38.16

IHD=ischaemic heart disease. Back & neck=low back and neck pain. Diabetes=diabetes mellitus. Stroke=Cerebrovascular disease. Lung C=lung, bronchus, and trachea cancers. Sense=sense organ diseases. Depression=depressive disorders. Alzheimer's=Alzheimer's disease and other dementias. Oth MSK=other musculoskeletal disorders. COPD=chronic obstructive pulmonary disease. NN Preterm=neonatal preterm birth complications. Diarrhoea=diarrhoeal diseases. Skin=skin and subcutaneous diseases. NN Enceph=neonatal encephalopathy due to birth asphyxia and trauma. Drugs=drug use disorders. Congenital=congenital anomalies. Liver C=liver cancer. Stomach C=stomach cancer. CKD=chronic kidney disease. Anxiety=anxiety disorders. Iron=iron-deficiency anaemia. HIV=HIV/AIDS. Colorect C=colon and rectum cancer. LRI=lower respiratory infections. Cirr HepC=cirrhosis due to hepatitis C. CMP=cardiomyopathy and myocarditis. Cirr alc=cirrhosis due to alcohol use. Other Cardio=other cardiovascular and circulatory diseases. Alcohol=alcohol use disorders. Violence=interpersonal violence. HTN HD=hypertensive heart disease. Haemog=haemoglobinopathies and haemolytic anaemias. TB=tuberculosis. Prostate C=prostate cancer. War=collective violence and legal intervention . Other Unint= other unintentional injuries. Oth trans=other transport injuries. Other NN=Other neonatal disorders. NN Sepsis=neonatal sepsis and other neonatal infections. PEM=protein-energy malnutrition. STD=sexually transmitted diseases excluding HIV. Ebola=Ebola virus disease. Other NTD=other neglected tropical diseases. GBD=Global Burden of Disease. SDI=Socio-demographic Index. DALYs=disability-adjusted life-years.

Appendix Figure 2e: Leading ten level 3 causes of age-specific DALYs colored by percent change from 1990 to 2016 in High-Middle for GBD 2016

	1	2	3	4	5	6	7	8	9	10
**Early Neonatal**	NN Preterm	Congenital	NN Enceph	Oth NN	NN Sepsis	LRI	NN Hemol	Oth Inf	Endocrine	STD
**Late Neonatal**	Congenital	NN Preterm	NN Sepsis	Oth NN	NN Enceph	LRI	Oth Inf	NN Hemol	Diarrhea	SIDS
**Post Neonatal**	Congenital	LRI	NN Preterm	Diarrhea	F Body	SIDS	Oth NN	NN Enceph	Meningitis	Iron
**1 to 4**	Congenital	Skin	LRI	Road Inj	Iron	Drown	Diarrhea	PEM	Leukemia	Epilepsy
**5 to 9**	Skin	Road Inj	Congenital	Asthma	Iron	Drown	Sense	Conduct	Anxiety	Epilepsy
**10 to 14**	Skin	Conduct	Migraine	Anxiety	Road Inj	Depression	Congenital	Asthma	Sense	Back+Neck
**15 to 19**	Skin	Road Inj	Migraine	Depression	Anxiety	Back+Neck	Self Harm	Violence	Conduct	Sense
**20 to 24**	Road Inj	Skin	Migraine	Back+Neck	Self Harm	Depression	Drugs	Violence	Anxiety	Oth MSK
**25 to 29**	Road Inj	Back+Neck	Migraine	Drugs	Self Harm	Skin	Depression	Violence	Alcohol	Anxiety
**30 to 34**	Back+Neck	Road Inj	Migraine	Self Harm	Drugs	Depression	Skin	HIV	Alcohol	IHD
**35 to 39**	Back+Neck	Road Inj	Migraine	IHD	Depression	Self Harm	Alcohol	HIV	Drugs	Skin
**40 to 44**	Back+Neck	IHD	Road Inj	Migraine	Depression	Stroke	Self Harm	Alcohol	Diabetes	Sense
**45 to 49**	Back+Neck	IHD	Stroke	Road Inj	Migraine	Depression	Diabetes	Sense	Falls	Lung C
**50 to 54**	IHD	Back+Neck	Stroke	Diabetes	Lung C	Sense	Road Inj	Depression	Migraine	Falls
**55 to 59**	IHD	Stroke	Back+Neck	Lung C	Diabetes	Sense	Depression	Road Inj	Falls	COPD
**60 to 64**	IHD	Stroke	Back+Neck	Lung C	Sense	Diabetes	COPD	Stomach C	Falls	Depression
**65 to 69**	IHD	Stroke	Lung C	Back+Neck	Sense	Diabetes	COPD	Stomach C	Colorect C	Falls
**70 to 74**	IHD	Stroke	Sense	Lung C	COPD	Back+Neck	Diabetes	Alzheimer	Colorect C	CKD
**75 to 79**	IHD	Stroke	Sense	Alzheimer	COPD	Back+Neck	Diabetes	Lung C	Colorect C	Falls
**80 to 84**	IHD	Stroke	Alzheimer	Sense	COPD	Back+Neck	Diabetes	CKD	HTN HD	Falls
**85 to 89**	IHD	Stroke	Alzheimer	Sense	COPD	Back+Neck	LRI	Diabetes	Falls	HTN HD
**90 to 94**	IHD	Alzheimer	Stroke	Sense	COPD	LRI	Falls	HTN HD	CKD	Diabetes
**95 plus**	IHD	Alzheimer	Stroke	COPD	Sense	LRI	Falls	HTN HD	CKD	Diabetes

Percent change in age-specific DALYs

-0.97 to -0.79	-0.79 to -0.56	-0.56 to -0.42	-0.42 to -0.28	-0.28 to -0.17	-0.17 to -0.06	-0.06 to 0.09	0.09 to 0.34	0.34 to 0.7	0.7 to 2.14

IHD=ischaemic heart disease. Back & neck=low back and neck pain. Diabetes=diabetes mellitus. Stroke=Cerebrovascular disease. Lung C=lung, bronchus, and trachea cancers. Sense=sense organ diseases. Depression=depressive disorders. Alzheimer's=Alzheimer's disease and other dementias. Oth MSK=other musculoskeletal disorders. COPD=chronic obstructive pulmonary disease. NN Preterm=neonatal preterm birth complications. Diarrhoea=diarrhoeal diseases. Skin=skin and subcutaneous diseases. NN Enceph=neonatal encephalopathy due to birth asphyxia and trauma. Drugs=drug use disorders. Congenital=congenital anomalies. Liver C=liver cancer. Stomach C=stomach cancer. CKD=chronic kidney disease. Anxiety=anxiety disorders. Iron=iron-deficiency anaemia. HIV=HIV/AIDS. Colorect C=colon and rectum cancer. LRI=lower respiratory infections. Cirr HepC=cirrhosis due to hepatitis C. CMP=cardiomyopathy and myocarditis. Cirr alc=cirrhosis due to alcohol use. Other Cardio=other cardiovascular and circulatory diseases. Alcohol=alcohol use disorders. Violence=interpersonal violence. HTN HD=hypertensive heart disease. Haemog=haemoglobinopathies and haemolytic anaemias. TB=tuberculosis. Prostate C=prostate cancer. War=collective violence and legal intervention . Other Unint= other unintentional injuries. Oth trans=other transport injuries. Other NN=Other neonatal disorders. NN Sepsis=neonatal sepsis and other neonatal infections. PEM=protein-energy malnutrition. STD=sexually transmitted diseases excluding HIV. Ebola=Ebola virus disease. Other NTD=other neglected tropical diseases. GBD=Global Burden of Disease. SDI=Socio-demographic Index. DALYs=disability-adjusted life-years.

Appendix Figure 2f: Leading ten level 3 causes of age-specific DALYs colored by percent change from 1990 to 2016 in High SDI for GBD 2016

	1	2	3	4	5	6	7	8	9	10
**Early Neonatal**	NN Preterm	Congenital	Oth NN	NN Enceph	NN Sepsis	Endocrine	LRI	Violence	Oth Inf	NN Hemol
**Late Neonatal**	Congenital	NN Preterm	Oth NN	NN Sepsis	NN Enceph	SIDS	LRI	Endocrine	Oth Inf	Mech
**Post Neonatal**	Congenital	SIDS	NN Preterm	LRI	Mech	Diarrhea	F Body	Endocrine	Violence	Oth NN
**1 to 4**	Skin	Congenital	Asthma	URI	Road Inj	Diarrhea	Drown	Iron	ASD	Endocrine
**5 to 9**	Skin	Asthma	Congenital	Anxiety	Conduct	URI	ASD	Road Inj	Sense	Migraine
**10 to 14**	Skin	Anxiety	Conduct	Migraine	Depression	Asthma	Back+Neck	Congenital	Road Inj	Sense
**15 to 19**	Skin	Depression	Road Inj	Migraine	Anxiety	Back+Neck	Drugs	Self Harm	Oth MSK	Conduct
**20 to 24**	Drugs	Skin	Road Inj	Depression	Back+Neck	Self Harm	Migraine	Anxiety	Oth MSK	Violence
**25 to 29**	Drugs	Back+Neck	Migraine	Self Harm	Skin	Depression	Road Inj	Anxiety	Oth MSK	Alcohol
**30 to 34**	Back+Neck	Drugs	Migraine	Self Harm	Depression	Skin	Road Inj	Anxiety	Oth MSK	Alcohol
**35 to 39**	Back+Neck	Migraine	Drugs	Depression	Self Harm	Skin	Road Inj	Anxiety	Oth MSK	Falls
**40 to 44**	Back+Neck	Migraine	Depression	Self Harm	Drugs	Skin	IHD	Oth MSK	Anxiety	Road Inj
**45 to 49**	Back+Neck	IHD	Migraine	Depression	Self Harm	Diabetes	Skin	Oth MSK	Drugs	Anxiety
**50 to 54**	Back+Neck	IHD	Lung C	Diabetes	Migraine	Depression	Self Harm	Stroke	Sense	Oth MSK
**55 to 59**	Back+Neck	IHD	Lung C	Diabetes	Stroke	Sense	COPD	Depression	Colorect C	Falls
**60 to 64**	IHD	Back+Neck	Lung C	Diabetes	Stroke	Sense	COPD	Colorect C	Falls	Depression
**65 to 69**	IHD	Lung C	Back+Neck	Stroke	Diabetes	Sense	COPD	Colorect C	Falls	CKD
**70 to 74**	IHD	Lung C	Back+Neck	Stroke	Sense	COPD	Diabetes	Alzheimer	Colorect C	Falls
**75 to 79**	IHD	Stroke	Alzheimer	Lung C	Sense	Back+Neck	COPD	Diabetes	Colorect C	Falls
**80 to 84**	IHD	Alzheimer	Stroke	Sense	COPD	Back+Neck	Lung C	Diabetes	Falls	LRI
**85 to 89**	IHD	Alzheimer	Stroke	Sense	COPD	LRI	Falls	Back+Neck	CKD	Diabetes
**90 to 94**	Alzheimer	IHD	Stroke	Sense	LRI	Falls	COPD	CKD	Diabetes	Oth Cardio
**95 plus**	Alzheimer	IHD	Stroke	LRI	Falls	Sense	CKD	COPD	HTN HD	Oth Cardio

Percent change in age-specific DALYs

-0.89 to -0.67	-0.67 to -0.55	-0.55 to -0.42	-0.42 to -0.27	-0.27 to -0.2	-0.2 to -0.07	-0.07 to 0.02	0.02 to 0.24	0.24 to 0.56	0.56 to 3.16

IHD=ischaemic heart disease. Back & neck=low back and neck pain. Diabetes=diabetes mellitus. Stroke=Cerebrovascular disease. Lung C=lung, bronchus, and trachea cancers. Sense=sense organ diseases. Depression=depressive disorders. Alzheimer's=Alzheimer's disease and other dementias. Oth MSK=other musculoskeletal disorders. COPD=chronic obstructive pulmonary disease. NN Preterm=neonatal preterm birth complications. Diarrhoea=diarrhoeal diseases. Skin=skin and subcutaneous diseases. NN Enceph=neonatal encephalopathy due to birth asphyxia and trauma. Drugs=drug use disorders. Congenital=congenital anomalies. Liver C=liver cancer. Stomach C=stomach cancer. CKD=chronic kidney disease. Anxiety=anxiety disorders. Iron=iron-deficiency anaemia. HIV=HIV/AIDS. Colorect C=colon and rectum cancer. LRI=lower respiratory infections. Cirr HepC=cirrhosis due to hepatitis C. CMP=cardiomyopathy and myocarditis. Cirr alc=cirrhosis due to alcohol use. Other Cardio=other cardiovascular and circulatory diseases. Alcohol=alcohol use disorders. Violence=interpersonal violence. HTN HD=hypertensive heart disease. Haemog=haemoglobinopathies and haemolytic anaemias. TB=tuberculosis. Prostate C=prostate cancer. War=collective violence and legal intervention . Other Unint= other unintentional injuries. Oth trans=other transport injuries. Other NN=Other neonatal disorders. NN Sepsis=neonatal sepsis and other neonatal infections. PEM=protein-energy malnutrition. STD=sexually transmitted diseases excluding HIV. Ebola=Ebola virus disease. Other NTD=other neglected tropical diseases. GBD=Global Burden of Disease. SDI=Socio-demographic Index. DALYs=disability-adjusted life-years.

Appendix Figure 3a−f. Composition of number of DALYs by level 2 causes for 23 GBD age groups, both sexes, combined, 1990 vs. 2016

a) Global

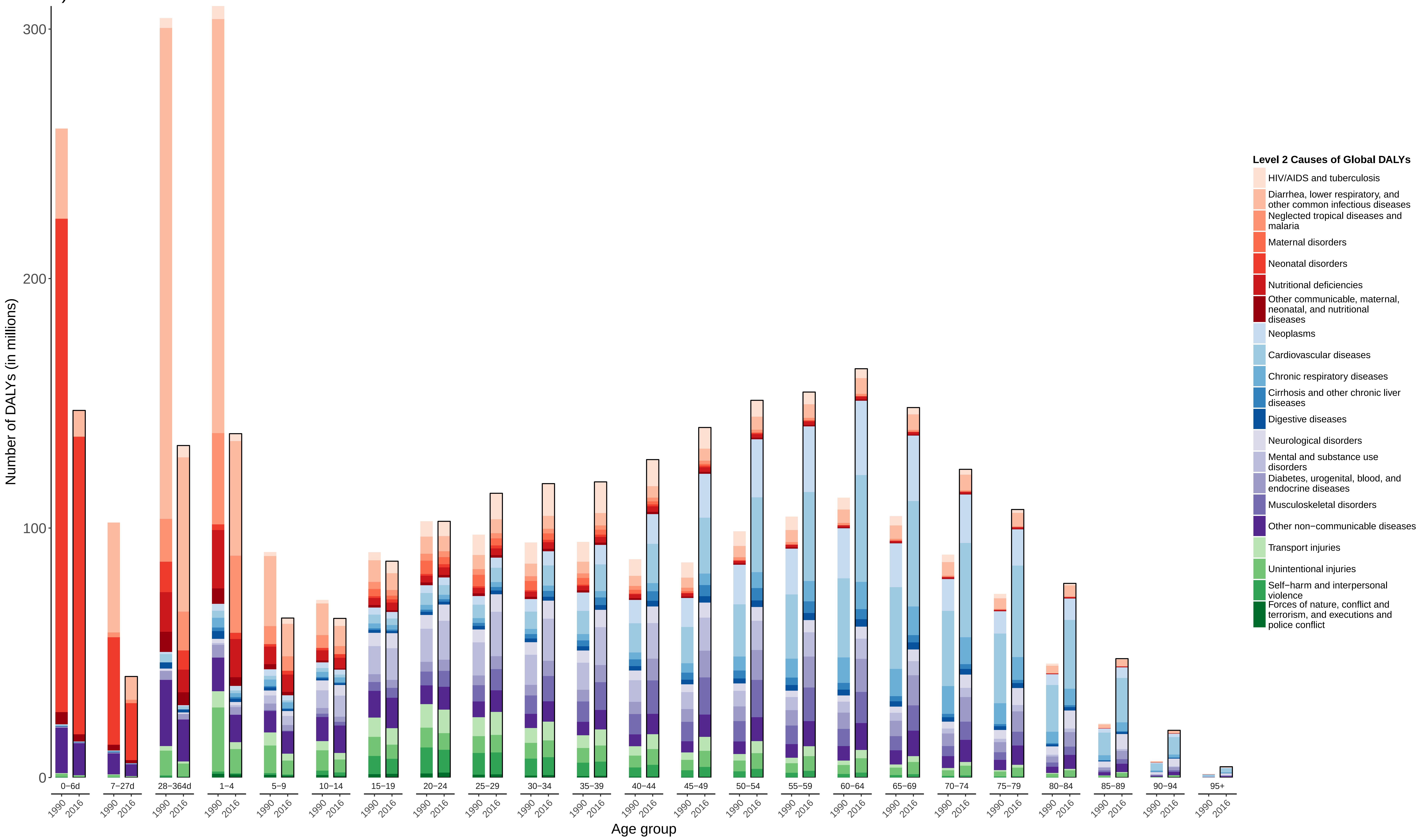

Appendix Figure 3a−f. Composition of number of DALYs by level 2 causes for 23 GBD age groups, both sexes, combined, 1990 vs. 2016

b) Low SDI

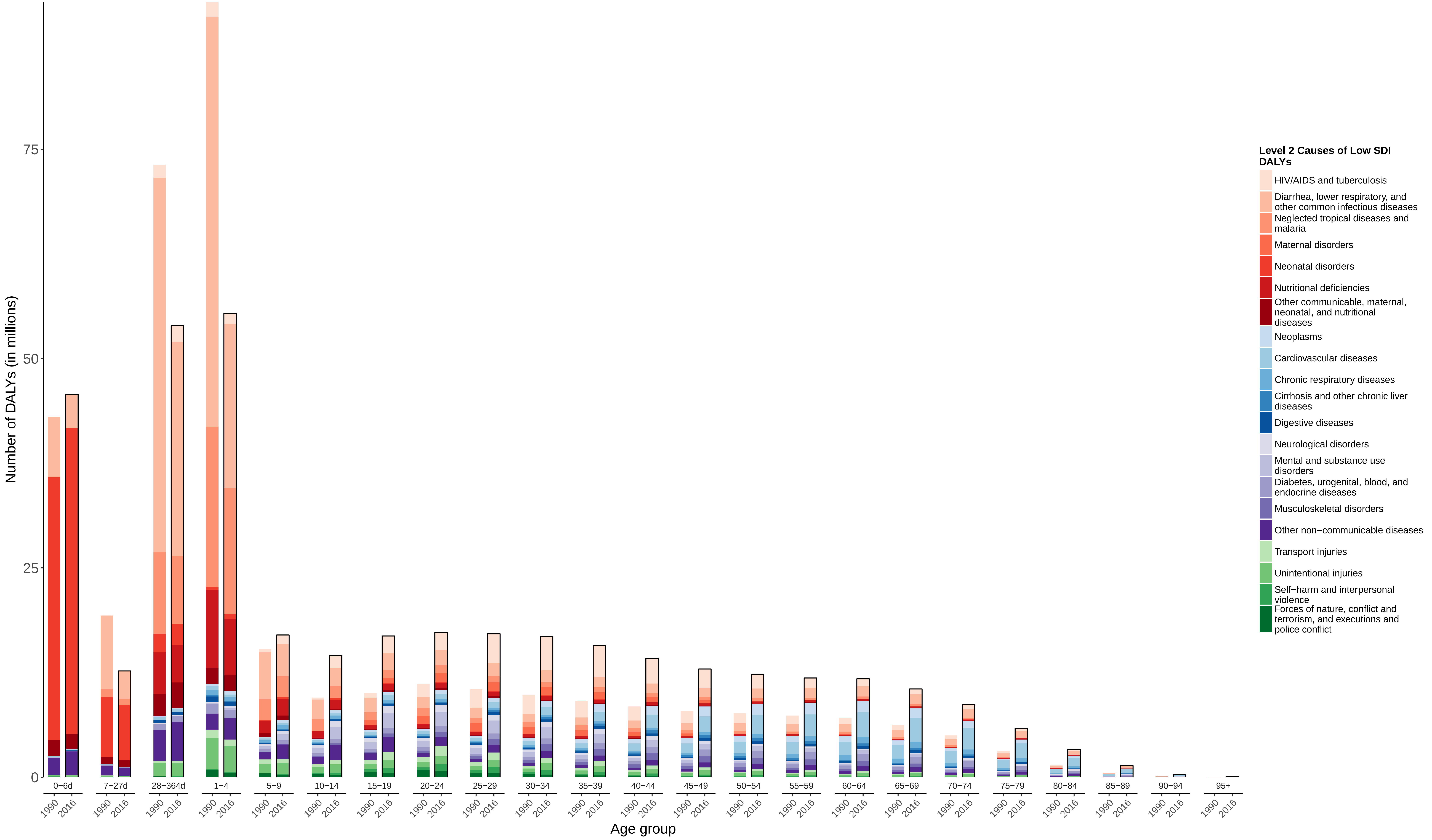

Appendix Figure 3a−f. Composition of number of DALYs by level 2 causes for 23 GBD age groups, both sexes, combined, 1990 vs. 2016

c) Low−middle SDI

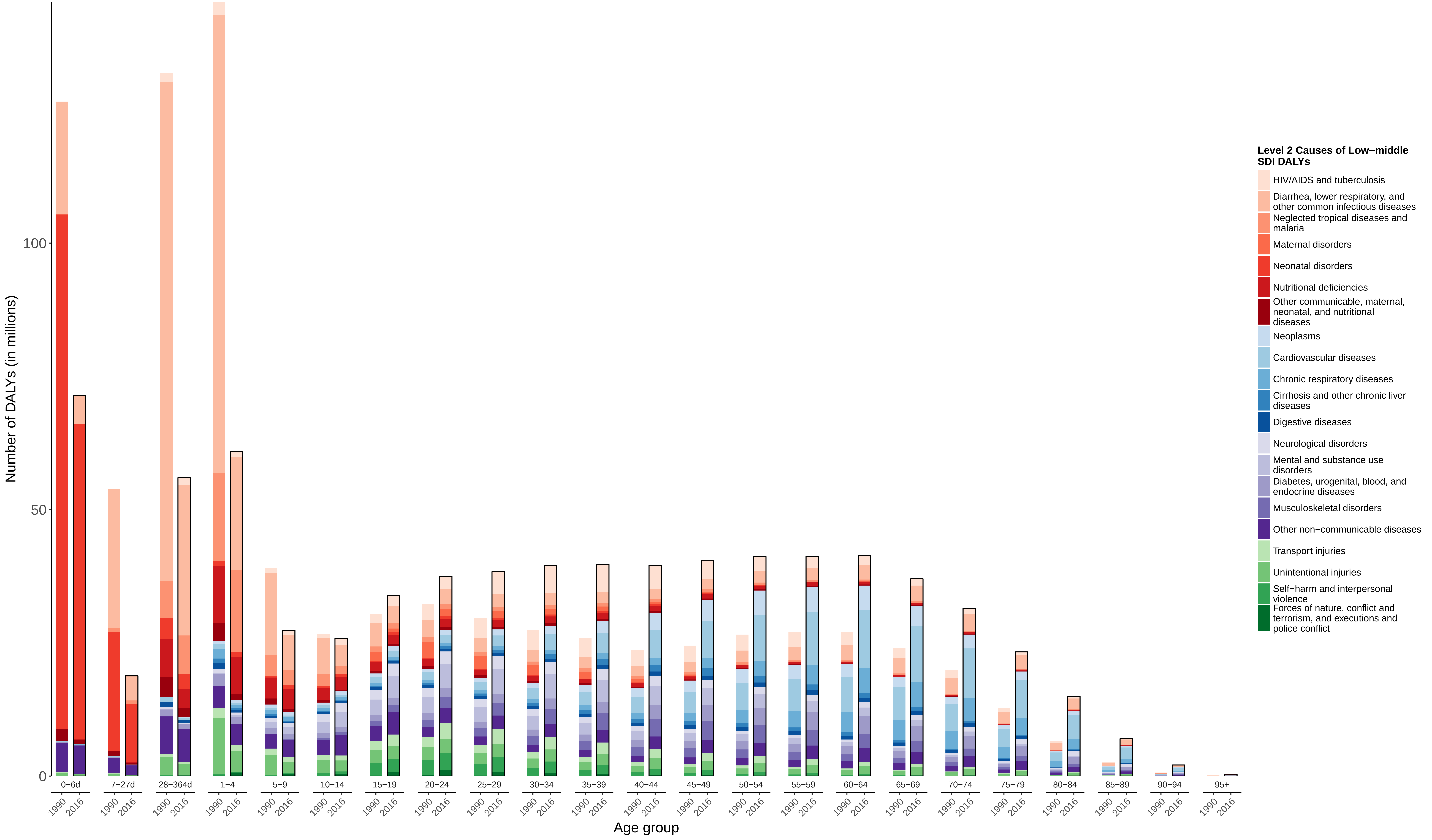

Appendix Figure 3a−f. Composition of number of DALYs by level 2 causes for 23 GBD age groups, both sexes, combined, 1990 vs. 2016

d) Middle SDI

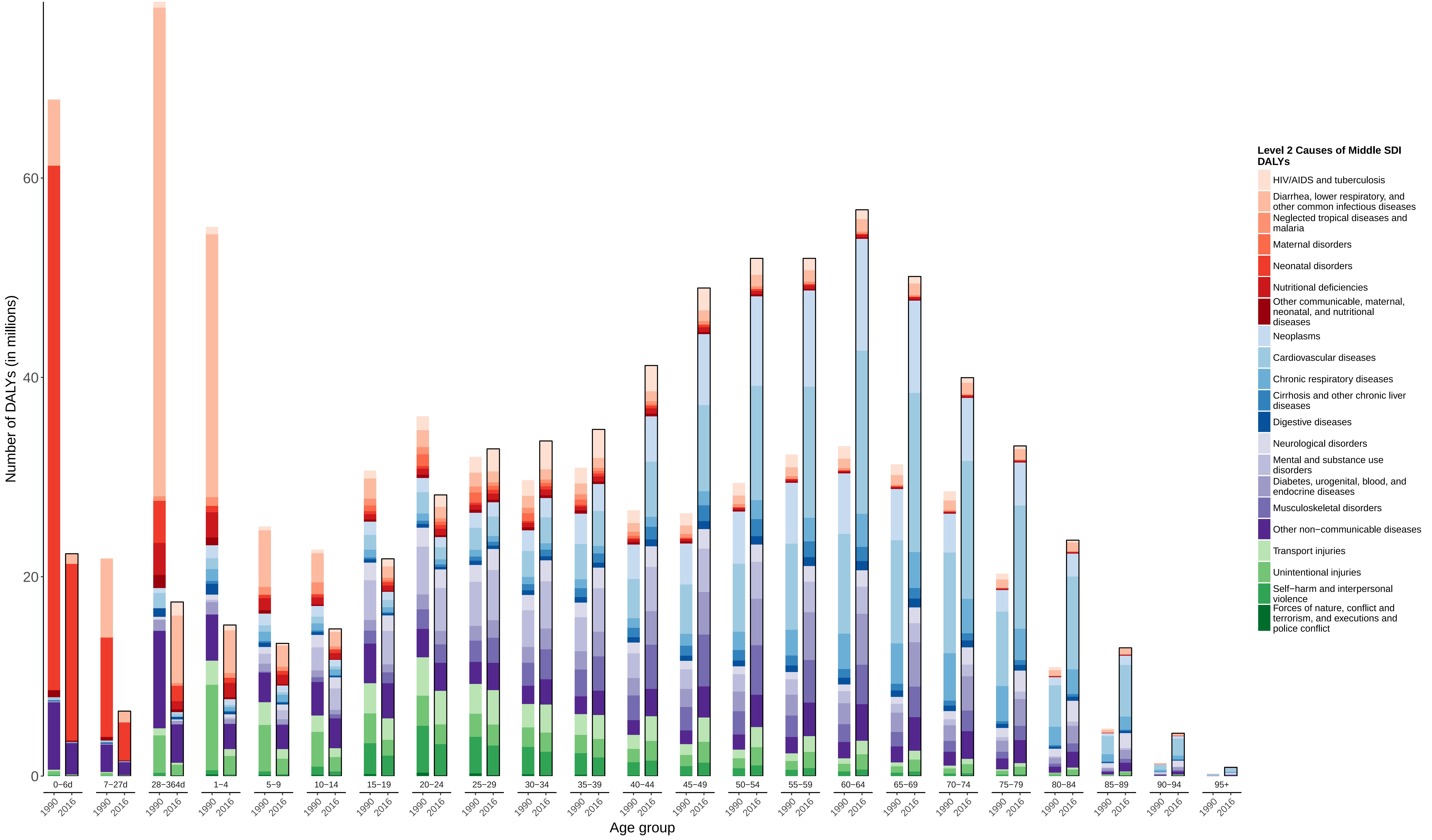

Appendix Figure 3a−f. Composition of number of DALYs by level 2 causes for 23 GBD age groups, both sexes, combined, 1990 vs. 2016

e) High−middle SDI

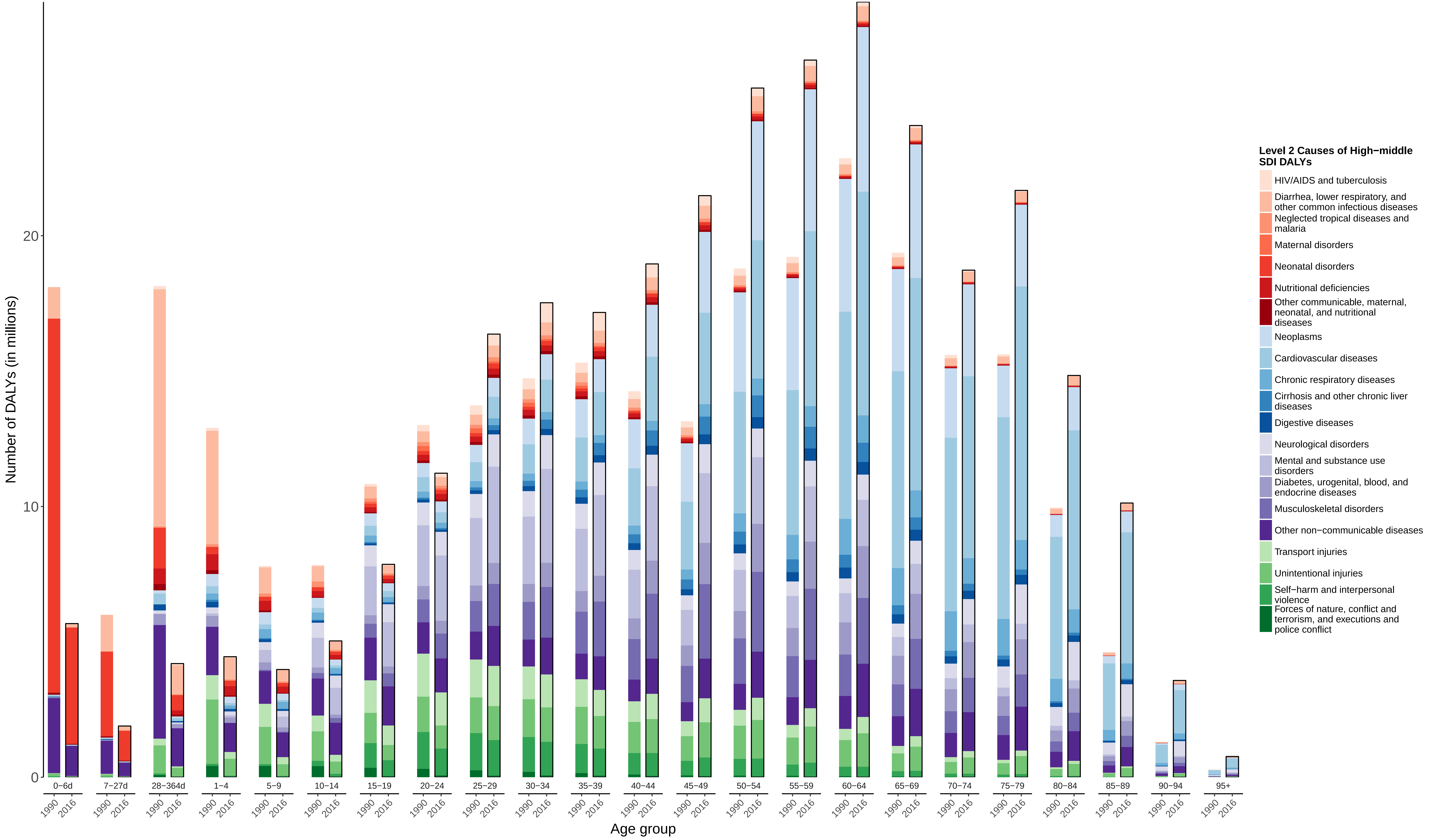

Appendix Figure 3a−f. Composition of number of DALYs by level 2 causes for 23 GBD age groups, both sexes, combined, 1990 vs. 2016

f) High SDI

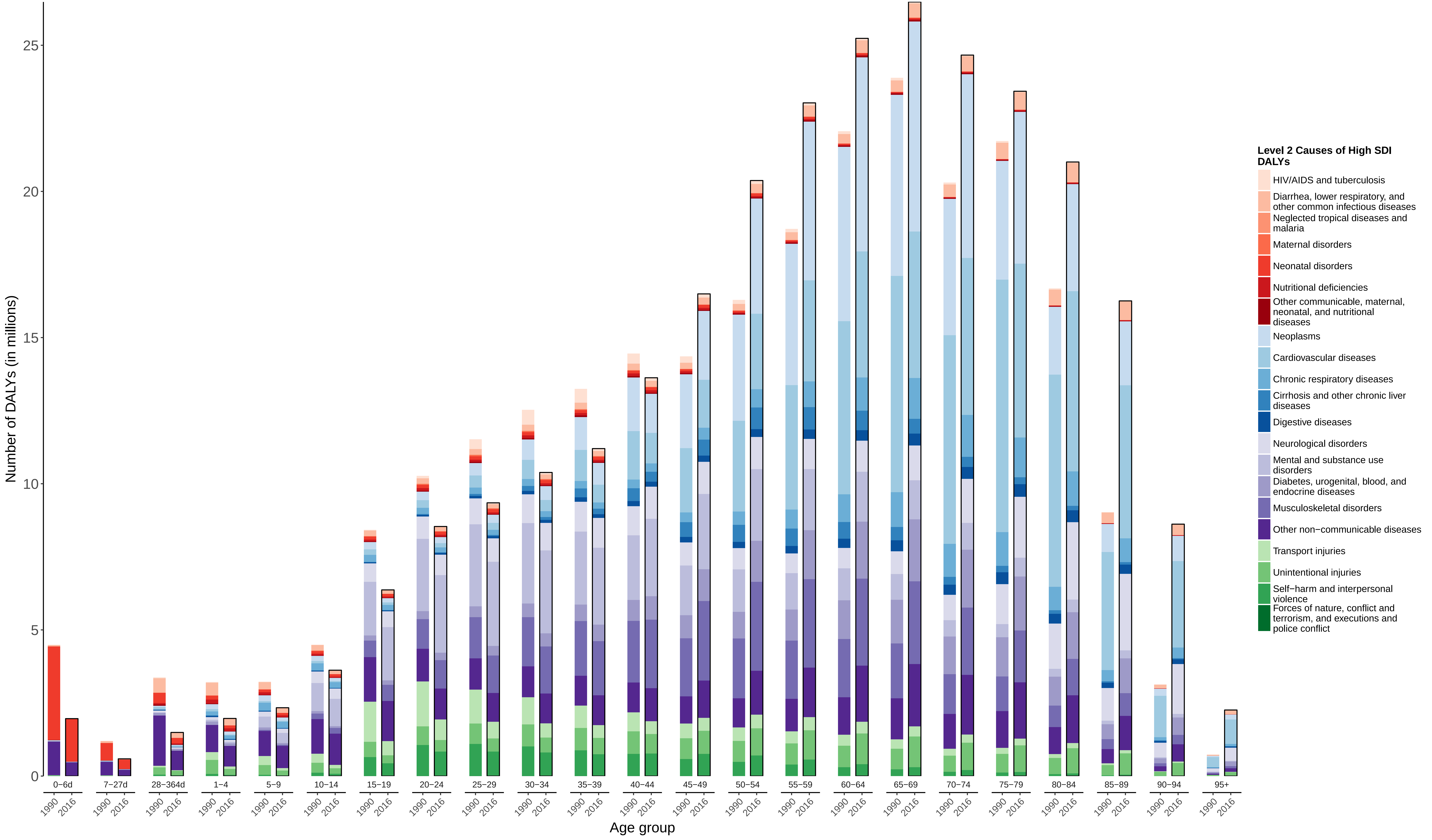

Appendix Figure 4. Leading level 3 causes of all-age DALY counts by location for i) females and ii) males, separately, in 1990

i)

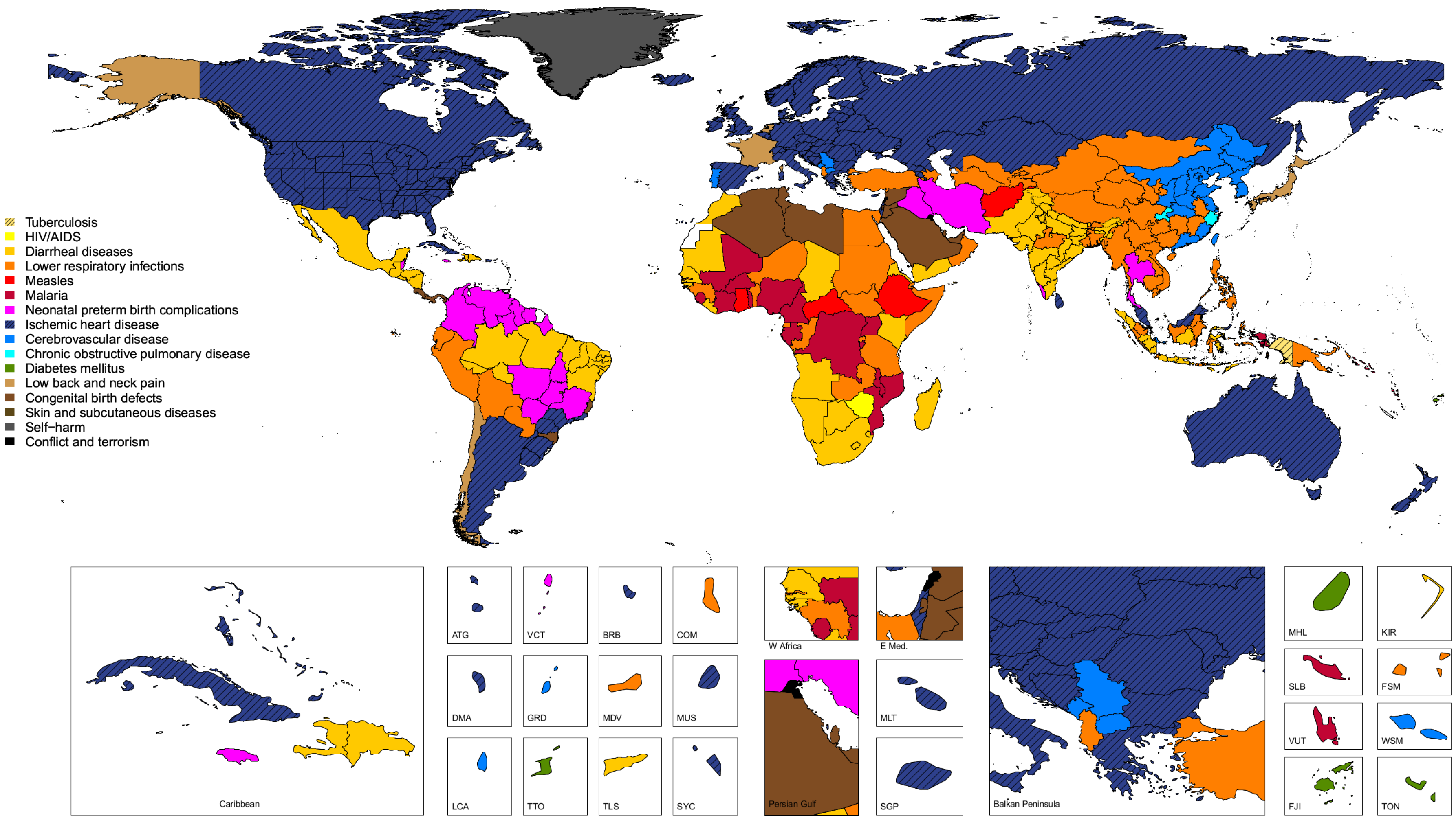

ii)

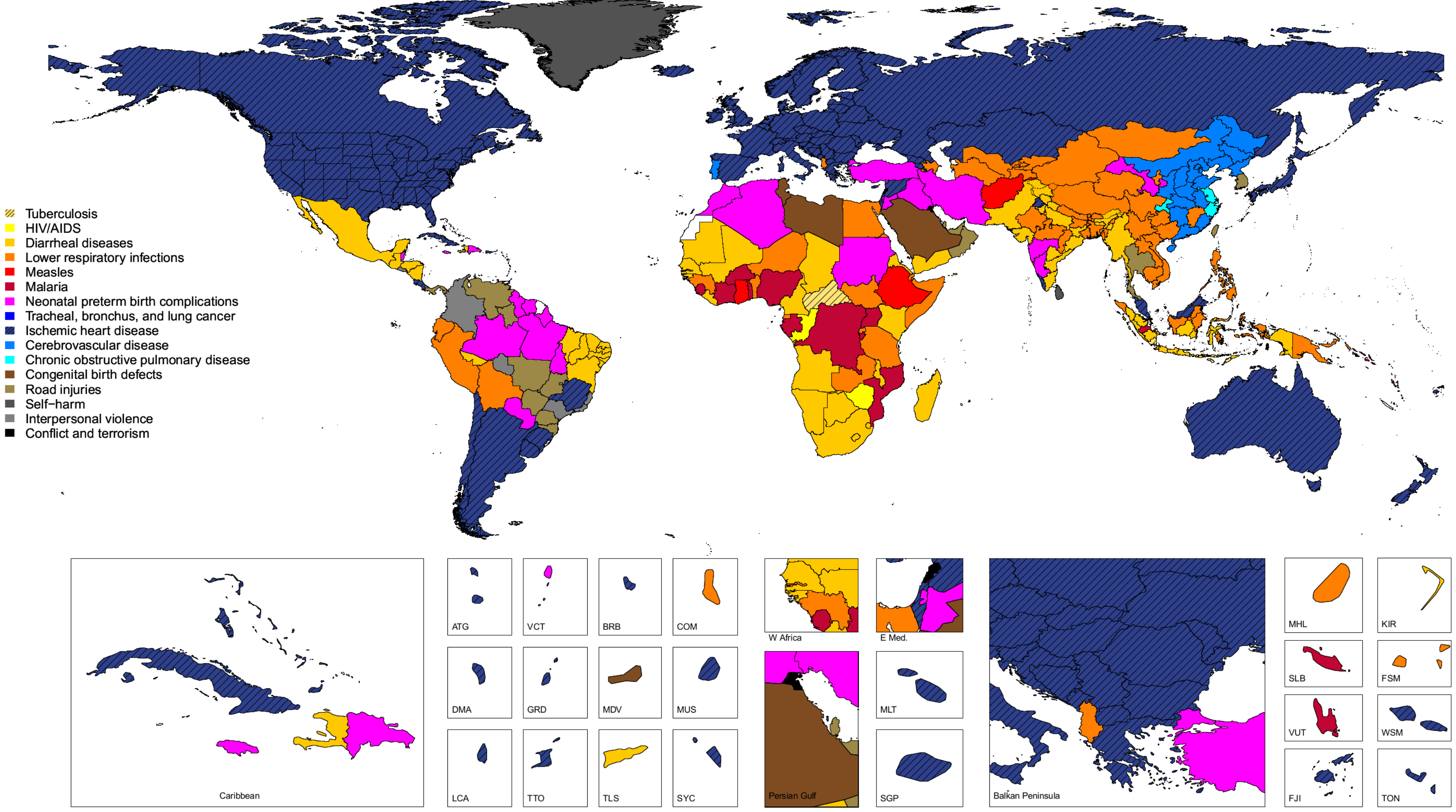

ATG=Antigua and Barbuda. BRB=Barbados. COM=Comoros. DMA=Dominica. E Med=Eastern Mediterranean. FJI=Fiji. FSM=Federated States of Micronesia. GRD=Grenada. KIR=Kiribati. LCA=Saint Lucia. MDV=Maldives. MHL=Marshall Islands. MLT=Malta. MUS=Mauritius. SGP=Singapore. SLB=Solomon Islands. SYC=Seychelles. TLS=Timor-Leste. TON=Tonga. TTO=Trinidad and Tobago. VCT=Saint Vincent and the Grenadines. VUT=Vanuatu. W Africa=West Africa. WSM=Samoa. DALYs=disability adjusted life years.

Appendix Figure 5. Annualized rate of change in age-standardised DALYs from 1990 to 2016 vs. global age-standardised DALYs per 100,000 due to each GBD 2016 level 3 cause

Not shown in figure: Zika: DALY = 0.07 ARC = 0.6, Guinea worm disease: DALY = 0 ARC = -1.04, Ebola: DALY = 0.004 ARC = 0.35

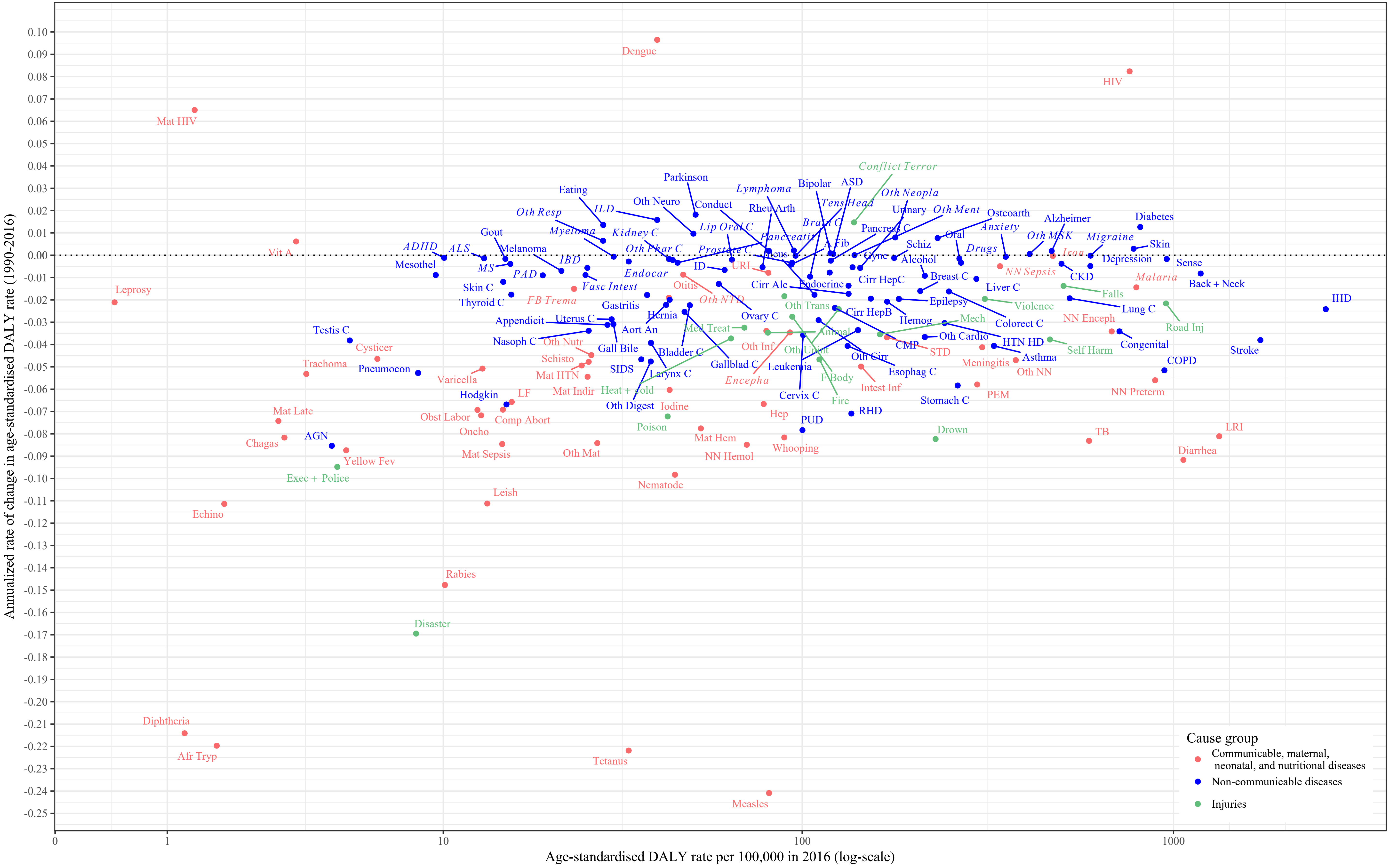

Figure 4. Ratio of global all−age DALY counts from non−communicable diseases (NCDs) to communicable, maternal, neonatal, and nutritional (CMNN) diseases by GBD region, for males and females separately, for 1990, 1995, 2000, 2006, 2010, and 2016.

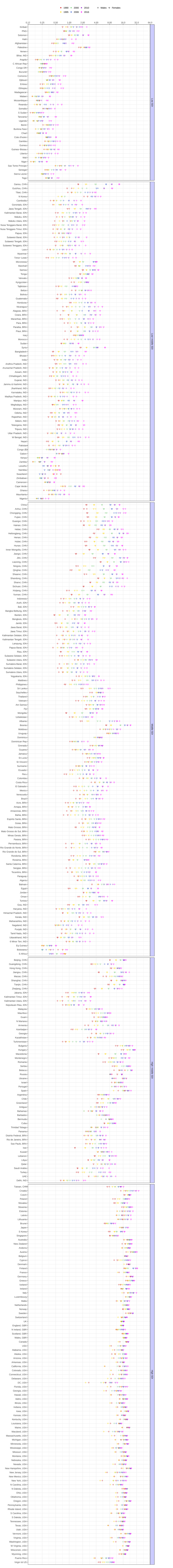

Each point represents the ratio of DALYs from non−communicable diseases (NCDs) to communicable, maternal, neonatal, and nutritional (CMNN) diseases for each GBD region in the given year, by sex. Points are colour−coded by year with circles for males and triangles for females. Ratios above 1.0 represent a greater number of DALYs from non−communicable diseases than from communicable, maternal, neonatal, and nutritional diseases. DALYs=disability−adjusted life−years. Taiwan, CHN = Taiwan, Province of China. Congo (B) = Congo (Brazzaville).

Appendix Table 2. GBD 2016 Life expectancy at birth and HALE at birth and at age 65 by location for GBD years 1990, 2000, 2006 & 2016, both sexes combined.

Location	1990			2000			2006			2016		
	Life Expectancy	HALE		Life Expectancy	HALE		Life Expectancy	HALE		Life Expectancy	HALE	
	At birth	At birth	At age 65	At birth	At birth	At age 65	At birth	At birth	At age 65	At birth	At birth	At age 65
Global	65.10 (64.87-65.32)	56.88 (54.53-59.02)	11.08 (10.11-11.96)	66.82 (66.62-67.01)	58.38 (55.93-60.55)	11.50 (10.50-12.41)	68.84 (68.60-69.06)	60.09 (57.57-62.33)	12.06 (10.98-13.04)	72.48 (72.10-72.83)	63.12 (60.42-65.48)	12.91 (11.75-13.96)
High SDI	75.78 (75.70-75.85)	66.06 (63.31-68.54)	12.81 (11.71-13.81)	77.93 (77.86-78.00)	67.81 (64.91-70.38)	13.73 (12.54-14.79)	79.28 (79.22-79.34)	68.84 (65.85-71.48)	14.47 (13.19-15.61)	80.74 (80.59-80.91)	69.94 (66.85-72.70)	15.20 (13.85-16.41)
High-middle SDI	69.98 (69.63-70.31)	61.18 (58.55-63.47)	11.28 (10.26-12.22)	70.92 (70.58-71.25)	62.18 (59.62-64.44)	11.41 (10.40-12.33)	72.97 (72.64-73.32)	63.93 (61.28-66.28)	12.01 (10.89-12.99)	76.44 (75.59-77.16)	66.73 (63.87-69.32)	13.26 (11.98-14.42)
Middle SDI	66.34 (66.05-66.62)	58.69 (56.44-60.68)	10.52 (9.67-11.32)	68.94 (68.74-69.14)	60.94 (58.60-63.01)	11.04 (10.11-11.90)	70.87 (70.67-71.07)	62.55 (60.13-64.71)	11.66 (10.67-12.57)	74.00 (73.71-74.28)	65.09 (62.49-67.30)	12.58 (11.51-13.54)
Low-middle SDI	59.37 (59.00-59.75)	51.35 (49.08-53.46)	9.31 (8.41-10.14)	61.71 (61.34-62.06)	53.37 (50.97-55.49)	9.65 (8.72-10.51)	63.92 (63.54-64.29)	55.29 (52.85-57.48)	10.08 (9.10-10.96)	68.18 (67.68-68.61)	58.96 (56.29-61.32)	10.77 (9.72-11.69)
Low SDI	52.41 (51.97-52.88)	45.30 (43.22-47.21)	8.57 (7.70-9.33)	53.69 (53.26-54.16)	46.49 (44.36-48.39)	8.71 (7.83-9.49)	56.78 (56.27-57.25)	49.27 (47.05-51.29)	9.12 (8.22-9.92)	62.84 (62.10-63.55)	54.64 (52.21-56.84)	9.85 (8.90-10.71)
High-income	76.04 (75.96-76.12)	66.32 (63.53-68.80)	13.00 (11.91-14.01)	78.17 (78.10-78.24)	68.03 (65.11-70.59)	13.92 (12.73-14.98)	79.52 (79.46-79.58)	69.07 (66.08-71.73)	14.65 (13.37-15.78)	80.91 (80.76-81.06)	70.12 (67.03-72.88)	15.36 (14.02-16.55)
High-income North America	75.64 (75.56-75.71)	65.50 (62.64-68.08)	13.13 (12.03-14.17)	77.18 (77.11-77.25)	66.66 (63.67-69.29)	13.53 (12.34-14.58)	78.20 (78.13-78.27)	67.39 (64.31-70.13)	14.09 (12.81-15.23)	79.16 (78.99-79.32)	68.05 (64.88-70.81)	14.54 (13.20-15.75)
Canada	77.43 (77.21-77.67)	67.80 (65.03-70.32)	13.78 (12.66-14.78)	79.26 (79.03-79.47)	69.19 (66.29-71.79)	14.40 (13.21-15.48)	80.48 (80.29-80.69)	70.23 (67.32-72.85)	15.15 (13.96-16.27)	81.85 (81.54-82.21)	71.20 (68.14-73.96)	15.87 (14.54-17.09)
Greenland	61.40 (60.31-62.39)	54.01 (51.73-55.95)	7.75 (7.02-8.40)	65.05 (64.18-65.93)	57.11 (54.77-59.32)	8.23 (7.51-8.92)	66.76 (65.91-67.62)	58.54 (56.25-60.70)	8.61 (7.86-9.32)	70.00 (67.91-72.02)	61.26 (58.34-64.20)	9.88 (8.78-11.08)
United States	75.45 (75.37-75.53)	65.26 (62.38-67.85)	13.07 (11.97-14.10)	76.96 (76.89-77.03)	66.39 (63.40-69.04)	13.43 (12.24-14.49)	77.95 (77.88-78.02)	67.08 (63.99-69.84)	13.97 (12.69-15.12)	78.86 (78.67-79.03)	67.69 (64.51-70.47)	14.38 (13.05-15.60)
Australasia	76.79 (76.61-76.95)	66.93 (64.05-69.48)	13.25 (12.17-14.23)	79.52 (79.36-79.69)	69.16 (66.16-71.81)	14.59 (13.40-15.64)	81.07 (80.91-81.22)	70.39 (67.24-73.16)	15.38 (14.10-16.52)	82.35 (81.88-82.79)	71.41 (68.28-74.15)	16.02 (14.69-17.22)
Australia	77.02 (76.81-77.21)	67.10 (64.21-69.65)	13.32 (12.23-14.31)	79.71 (79.53-79.91)	69.30 (66.28-71.98)	14.67 (13.47-15.72)	81.27 (81.10-81.45)	70.54 (67.38-73.32)	15.46 (14.18-16.61)	82.52 (81.97-83.03)	71.53 (68.38-74.28)	16.09 (14.74-17.30)
New Zealand	75.64 (75.39-75.89)	66.08 (63.26-68.55)	12.92 (11.85-13.89)	78.55 (78.32-78.78)	68.41 (65.50-71.09)	14.18 (13.04-15.20)	80.04 (79.82-80.26)	69.66 (66.58-72.35)	14.95 (13.70-16.03)	81.48 (80.56-82.30)	70.80 (67.75-73.60)	15.64 (14.30-16.88)
High-income Asia Pacific	77.45 (77.17-77.71)	68.12 (65.47-70.60)	13.65 (12.52-14.68)	80.02 (79.82-80.24)	70.16 (67.31-72.72)	14.91 (13.64-16.07)	81.58 (81.37-81.78)	71.34 (68.39-74.01)	15.67 (14.33-16.90)	83.30 (82.63-83.93)	72.64 (69.55-75.42)	16.54 (15.10-17.83)
Brunei	73.34 (72.50-74.19)	64.29 (61.71-66.80)	11.61 (10.54-12.64)	75.83 (75.10-76.51)	66.42 (63.81-68.85)	12.67 (11.56-13.71)	76.63 (76.01-77.25)	67.17 (64.55-69.62)	12.94 (11.81-14.01)	76.88 (75.51-78.62)	67.48 (64.62-70.19)	13.16 (11.91-14.35)
Japan	78.94 (78.91-78.98)	69.46 (66.71-71.89)	14.06 (12.90-15.10)	81.03 (80.99-81.07)	71.04 (68.15-73.60)	15.29 (13.99-16.45)	82.24 (82.20-82.28)	71.93 (68.92-74.54)	15.98 (14.61-17.20)	83.94 (83.77-84.12)	73.16 (70.00-75.89)	16.84 (15.40-18.10)
Singapore	75.41 (74.28-76.50)	66.75 (64.08-69.20)	12.55 (11.39-13.68)	79.38 (78.28-80.44)	70.08 (67.25-72.78)	14.32 (13.04-15.60)	81.16 (80.15-82.12)	71.68 (68.83-74.28)	15.30 (14.00-16.55)	83.73 (81.95-85.38)	73.62 (70.48-76.71)	16.74 (15.15-18.33)
South Korea	72.03 (70.91-73.14)	63.43 (60.84-65.94)	11.33 (10.26-12.34)	76.05 (75.19-76.96)	66.81 (64.01-69.38)	12.71 (11.57-13.80)	78.90 (77.99-79.79)	68.99 (66.13-71.76)	13.91 (12.61-15.18)	81.00 (78.51-83.58)	70.76 (67.36-74.06)	15.05 (13.30-16.89)
Western Europe	76.29 (76.16-76.40)	66.56 (63.75-69.06)	12.79 (11.68-13.77)	78.56 (78.45-78.67)	68.47 (65.55-71.02)	13.84 (12.64-14.89)	80.08 (79.98-80.18)	69.71 (66.71-72.35)	14.63 (13.38-15.77)	81.69 (81.48-81.90)	71.01 (67.90-73.77)	15.44 (14.13-16.66)
Andorra	79.64 (78.09-81.46)	68.80 (65.57-72.04)	14.26 (12.74-15.79)	81.94 (80.35-83.38)	70.67 (67.39-73.82)	15.44 (13.79-16.91)	82.63 (81.29-83.78)	71.23 (67.71-74.34)	15.78 (14.21-17.17)	82.52 (80.87-84.13)	71.20 (67.62-74.27)	15.73 (14.07-17.20)
Austria	75.69 (75.49-75.89)	66.21 (63.48-68.69)	12.57 (11.49-13.55)	78.26 (78.08-78.46)	68.33 (65.47-70.88)	13.69 (12.49-14.78)	79.87 (79.69-80.05)	69.63 (66.64-72.25)	14.50 (13.22-15.60)	81.54 (81.05-82.01)	70.94 (67.84-73.68)	15.30 (13.91-16.54)
Belgium	75.99 (75.66-76.34)	66.31 (63.47-68.77)	12.49 (11.37-13.49)	77.83 (77.48-78.17)	67.64 (64.72-70.30)	13.35 (12.15-14.46)	79.42 (79.11-79.73)	68.77 (65.70-71.47)	14.07 (12.76-15.23)	80.91 (80.07-81.77)	70.11 (66.95-72.92)	14.87 (13.46-16.17)
Cyprus	74.92 (74.57-75.26)	65.64 (63.01-68.06)	11.34 (10.37-12.26)	76.82 (76.49-77.15)	67.15 (64.36-69.64)	12.65 (11.53-13.64)	78.66 (78.29-78.98)	68.66 (65.69-71.19)	13.56 (12.37-14.63)	80.48 (80.00-80.95)	70.20 (67.25-72.93)	14.42 (13.19-15.55)
Denmark	75.20 (74.64-75.80)	65.59 (62.83-68.11)	12.37 (11.28-13.36)	76.95 (76.39-77.53)	67.02 (64.10-69.62)	12.85 (11.71-13.90)	78.36 (77.81-78.91)	68.04 (65.10-70.74)	13.56 (12.35-14.65)	80.82 (79.81-81.83)	69.88 (66.85-72.73)	14.74 (13.41-16.00)
Finland	75.06 (74.79-75.33)	65.11 (62.23-67.73)	12.11 (11.03-13.13)	77.81 (77.55-78.05)	67.28 (64.18-69.97)	13.23 (11.97-14.33)	79.18 (78.92-79.43)	68.30 (65.15-71.13)	14.06 (12.72-15.29)	81.74 (81.14-82.41)	70.53 (67.27-73.47)	15.27 (13.85-16.60)
France	77.16 (76.92-77.42)	67.39 (64.54-69.90)	13.87 (12.67-14.95)	79.12 (78.89-79.38)	69.16 (66.26-71.74)	14.76 (13.52-15.83)	80.67 (80.43-80.92)	70.45 (67.47-73.06)	15.61 (14.30-16.76)	82.34 (81.91-82.79)	71.71 (68.59-74.49)	16.40 (14.96-17.69)
Germany	75.47 (75.00-75.89)	65.77 (62.99-68.23)	12.29 (11.20-13.29)	78.02 (77.63-78.43)	67.88 (64.92-70.51)	13.46 (12.25-14.54)	79.49 (79.10-79.91)	69.06 (66.10-71.79)	14.19 (12.95-15.32)	80.93 (80.24-81.62)	70.23 (67.04-73.11)	14.90 (13.56-16.21)
Greece	77.00 (76.74-77.24)	67.37 (64.58-69.82)	12.90 (11.81-13.87)	78.62 (78.39-78.86)	68.77 (65.93-71.32)	13.76 (12.63-14.80)	79.85 (79.63-80.09)	69.68 (66.73-72.24)	14.44 (13.22-15.51)	80.97 (80.34-81.61)	70.63 (67.74-73.30)	15.14 (13.93-16.35)
Iceland	78.02 (77.60-78.46)	67.96 (65.01-70.58)	13.46 (12.32-14.51)	79.89 (79.48-80.30)	69.54 (66.53-72.14)	14.25 (13.01-15.33)	81.35 (80.98-81.78)	70.71 (67.56-73.37)	14.92 (13.61-16.05)	82.27 (81.56-82.96)	71.48 (68.27-74.32)	15.44 (14.08-16.65)
Ireland	74.84 (74.41-75.31)	65.47 (62.69-67.93)	11.60 (10.58-12.53)	76.62 (76.13-77.10)	66.90 (64.12-69.46)	12.52 (11.46-13.53)	79.27 (78.85-79.72)	68.97 (66.02-71.61)	13.94 (12.73-15.06)	81.14 (80.04-82.19)	70.45 (67.14-73.23)	15.00 (13.59-16.27)
Israel	75.84 (75.01-76.64)	66.44 (63.60-68.99)	12.16 (11.00-13.22)	78.52 (77.59-79.47)	68.52 (65.60-71.24)	13.60 (12.33-14.84)	79.85 (79.11-80.67)	69.58 (66.53-72.29)	14.36 (13.03-15.59)	82.14 (80.58-83.63)	71.44 (68.21-74.48)	15.44 (13.92-16.96)
Italy	77.02 (76.83-77.21)	67.25 (64.43-69.78)	13.06 (11.91-14.09)	79.60 (79.43-79.79)	69.48 (66.56-72.08)	14.23 (12.99-15.34)	81.09 (80.90-81.25)	70.73 (67.71-73.41)	15.00 (13.71-16.17)	82.34 (81.82-82.87)	71.75 (68.71-74.54)	15.63 (14.28-16.88)
Luxembourg	75.19 (74.83-75.53)	65.23 (62.31-67.82)	12.08 (10.95-13.10)	78.51 (78.16-78.88)	67.88 (64.80-70.63)	13.55 (12.26-14.67)	80.43 (80.09-80.76)	69.48 (66.33-72.24)	14.50 (13.18-15.69)	82.12 (81.26-82.98)	70.96 (67.77-73.94)	15.30 (13.87-16.63)
Malta	75.77 (75.02-76.54)	66.36 (63.53-68.92)	11.85 (10.76-12.96)	77.51 (76.78-78.25)	67.84 (64.97-70.50)	12.72 (11.59-13.82)	79.51 (78.80-80.22)	69.40 (66.55-71.98)	13.96 (12.69-15.07)	81.43 (79.97-82.85)	70.93 (67.76-73.98)	15.14 (13.64-16.59)
Netherlands	77.03 (76.70-77.42)	67.01 (64.16-69.59)	12.76 (11.64-13.75)	78.14 (77.78-78.48)	67.82 (64.77-70.48)	13.19 (12.00-14.26)	79.86 (79.55-80.19)	69.26 (66.18-72.01)	14.07 (12.85-15.21)	81.64 (80.91-82.29)	70.70 (67.51-73.58)	14.99 (13.63-16.27)
Norway	76.81 (76.43-77.17)	67.12 (64.25-69.64)	12.86 (11.74-13.87)	78.74 (78.36-79.07)	68.68 (65.71-71.22)	13.70 (12.47-14.78)	80.40 (80.05-80.72)	70.07 (67.07-72.75)	14.56 (13.29-15.72)	82.10 (81.29-82.94)	71.49 (68.55-74.34)	15.39 (14.05-16.69)
Portugal	74.32 (74.03-74.59)	64.94 (62.18-67.32)	12.24 (11.18-13.19)	76.70 (76.45-76.96)	67.00 (64.16-69.55)	13.23 (12.11-14.27)	78.79 (78.52-79.08)	68.72 (65.75-71.30)	14.17 (12.99-15.25)	80.99 (80.43-81.56)	70.51 (67.45-73.27)	15.24 (13.91-16.42)
Spain	77.12 (76.94-77.30)	67.66 (64.83-70.13)	13.52 (12.37-14.55)	79.40 (79.24-79.56)	69.68 (66.81-72.18)	14.48 (13.27-15.55)	80.83 (80.68-80.98)	70.83 (67.87-73.44)	15.19 (13.90-16.33)	82.97 (82.59-83.35)	72.62 (69.67-75.32)	16.35 (15.03-17.55)
Sweden	77.63 (77.35-77.90)	67.49 (64.52-70.14)	13.14 (11.98-14.18)	79.70 (79.46-79.96)	69.11 (66.00-71.83)	13.93 (12.67-15.03)	80.77 (80.55-81.00)	69.98 (66.80-72.77)	14.51 (13.25-15.67)	82.05 (81.09-82.97)	70.88 (67.62-73.82)	15.11 (13.66-16.43)
Switzerland	77.49 (76.94-78.02)	67.17 (64.16-69.92)	13.31 (12.08-14.48)	79.97 (79.42-80.50)	68.85 (65.65-71.79)	14.31 (13.04-15.57)	81.48 (80.95-81.94)	70.16 (66.86-73.08)	15.10 (13.72-16.36)	83.18 (81.23-84.93)	71.93 (68.39-75.17)	16.02 (14.31-17.66)
United Kingdom	75.72 (75.63-75.82)	65.89 (63.04-68.44)	12.19 (11.13-13.13)	77.84 (77.75-77.94)	67.64 (64.69-70.26)	13.23 (12.09-14.25)	79.38 (79.29-79.47)	68.82 (65.77-71.51)	14.12 (12.90-15.23)	80.90 (80.73-81.05)	70.05 (66.93-72.84)	14.91 (13.60-16.09)
England	75.96 (75.89-76.04)	66.05 (63.18-68.59)	12.27 (11.19-13.24)	78.09 (78.02-78.16)	67.80 (64.83-70.45)	13.30 (12.16-14.34)	79.65 (79.58-79.71)	68.99 (65.92-71.70)	14.21 (12.97-15.32)	81.18 (81.07-81.28)	70.24 (67.10-73.04)	15.02 (13.70-16.20)
Northern Ireland	74.94 (74.23-75.69)	65.53 (62.80-68.05)	11.96 (10.89-12.98)	77.45 (76.72-78.13)	67.52 (64.60-70.09)	13.08 (11.92-14.11)	78.76 (78.08-79.44)	68.54 (65.62-71.36)	14.04 (12.81-15.21)	80.15 (79.08-81.39)	69.68 (66.60-72.69)	14.73 (13.42-16.07)
Scotland	73.94 (73.29-74.62)	64.60 (61.83-67.04)	11.55 (10.54-12.46)	75.97 (75.30-76.66)	66.28 (63.44-68.76)	12.64 (11.54-13.63)	77.36 (76.73-78.05)	67.40 (64.53-70.00)	13.43 (12.30-14.47)	79.06 (78.03-80.00)	68.68 (65.63-71.55)	14.17 (12.90-15.42)
Wales	75.52 (74.90-76.12)	65.94 (63.19-68.45)	12.08 (11.00-13.08)	77.44 (76.85-78.08)	67.57 (64.73-70.20)	13.12 (11.98-14.19)	79.04 (78.45-79.62)	68.85 (65.91-71.55)	14.08 (12.87-15.19)	80.06 (78.96-81.12)	69.63 (66.52-72.44)	14.63 (13.29-15.90)
Southern Latin America	72.50 (72.20-72.79)	63.65 (61.04-65.96)	12.41 (11.36-13.32)	74.98 (74.68-75.25)	65.69 (63.00-68.07)	13.17 (12.06-14.13)	76.17 (75.90-76.47)	66.72 (63.96-69.18)	13.57 (12.49-14.57)	77.69 (76.92-78.43)	67.96 (65.09-70.55)	14.26 (13.05-15.40)
Argentina	72.08 (71.72-72.49)	63.35 (60.73-65.68)	12.39 (11.34-13.33)	74.19 (73.79-74.53)	65.12 (62.50-67.44)	12.98 (11.90-13.93)	75.31 (74.96-75.71)	66.10 (63.38-68.52)	13.29 (12.22-14.29)	76.71 (75.99-77.46)	67.22 (64.39-69.77)	13.86 (12.71-14.95)
Chile	73.50 (72.88-74.08)	64.24 (61.57-66.72)	12.53 (11.46-13.55)	77.27 (76.72-77.84)	67.26 (64.37-69.90)	13.82 (12.68-14.88)	78.74 (78.16-79.28)	68.51 (65.48-71.14)	14.55 (13.32-15.64)	80.32 (78.05-82.55)	69.90 (66.35-73.25)	15.39 (13.58-17.18)
Uruguay	72.75 (72.42-73.06)	64.24 (61.71-66.39)	12.31 (11.29-13.19)	74.82 (74.54-75.12)	65.99 (63.41-68.25)	13.10 (12.07-14.03)	75.85 (75.57-76.16)	66.83 (64.23-69.12)	13.49 (12.39-14.44)	77.33 (76.69-78.01)	67.93 (65.23-70.44)	14.16 (13.02-15.26)
Central Europe, Eastern Europe, and Central Asia	69.18 (68.75-69.62)	60.28 (57.63-62.62)	10.66 (9.59-11.65)	67.57 (67.00-68.09)	59.15 (56.69-61.36)	10.38 (9.35-11.28)	68.97 (68.40-69.55)	60.39 (57.82-62.64)	10.80 (9.72-11.76)	72.73 (71.06-74.23)	63.43 (60.41-66.30)	11.98 (10.67-13.23)
Eastern Europe	69.46 (68.73-70.23)	60.45 (57.72-62.94)	10.74 (9.69-11.78)	66.03 (65.12-66.88)	57.80 (55.42-60.01)	10.01 (9.01-10.90)	67.34 (66.46-68.29)	59.03 (56.57-61.31)	10.47 (9.40-11.42)	71.42 (68.66-74.01)	62.36 (58.98-65.68)	11.68 (10.18-13.10)
Belarus	70.74 (70.15-71.29)	61.65 (58.97-64.07)	11.29 (10.17-12.30)	68.34 (67.72-68.94)	59.91 (57.53-62.21)	10.44 (9.44-11.37)	69.23 (68.65-69.81)	60.65 (58.12-62.87)	10.67 (9.63-11.61)	73.60 (71.85-75.53)	64.09 (61.04-67.06)	12.00 (10.61-13.41)
Estonia	70.17 (69.71-70.62)	60.88 (58.13-63.30)	10.75 (9.64-11.75)	70.84 (70.36-71.32)	61.69 (58.98-64.15)	11.29 (10.19-12.30)	73.20 (72.73-73.63)	63.69 (60.94-66.28)	12.05 (10.89-13.14)	77.58 (76.48-78.76)	67.16 (63.89-70.05)	13.55 (12.10-14.86)
Latvia	69.60 (69.07-70.21)	60.39 (57.70-62.77)	10.59 (9.48-11.59)	70.50 (69.93-71.11)	61.37 (58.69-63.79)	11.24 (10.10-12.30)	70.49 (69.97-71.06)	61.59 (58.95-63.88)	11.15 (10.02-12.14)	75.06 (73.84-76.42)	65.21 (62.20-68.00)	12.61 (11.31-13.86)
Lithuania	71.48 (71.08-71.93)	62.01 (59.28-64.44)	11.44 (10.28-12.49)	72.10 (71.66-72.54)	62.64 (59.90-65.14)	11.84 (10.66-12.94)	71.07 (70.61-71.52)	61.94 (59.25-64.34)	11.73 (10.55-12.80)	75.16 (74.34-75.97)	65.20 (62.16-67.90)	12.91 (11.58-14.11)
Moldova	67.27 (66.32-68.20)	58.70 (56.17-61.06)	9.96 (8.97-10.89)	67.62 (66.75-68.48)	59.35 (56.73-61.80)	10.04 (9.03-10.98)	68.94 (68.09-69.88)	60.62 (58.14-63.01)	10.14 (9.12-11.05)	72.25 (71.00-73.54)	63.28 (60.53-65.98)	11.31 (10.15-12.46)
Russia	69.17 (68.10-70.29)	60.18 (57.48-62.78)	10.68 (9.61-11.81)	65.17 (63.95-66.37)	57.03 (54.59-59.38)	9.82 (8.80-10.74)	66.85 (65.58-68.18)	58.58 (56.06-60.96)	10.41 (9.31-11.42)	70.87 (67.07-74.55)	61.91 (58.09-65.70)	11.60 (9.83-13.39)
Ukraine	70.08 (69.44-70.68)	61.06 (58.43-63.48)	10.79 (9.74-11.78)	67.35 (66.61-68.10)	59.05 (56.63-61.36)	10.24 (9.28-11.16)	67.82 (67.14-68.52)	59.53 (57.06-61.82)	10.44 (9.46-11.38)	72.10 (69.37-74.71)	63.03 (59.40-66.33)	11.71 (10.14-13.19)
Central Europe	70.93 (70.75-71.12)	61.87 (59.21-64.18)	10.42 (9.33-11.36)	72.87 (72.70-73.04)	63.65 (60.95-66.02)	11.09 (9.96-12.10)	74.37 (74.21-74.53)	64.84 (62.01-67.26)	11.64 (10.45-12.68)	76.87 (76.51-77.22)	66.78 (63.83-69.35)	12.64 (11.36-13.80)
Albania	73.73 (73.17-74.29)	64.41 (61.69-66.90)	12.43 (11.23-13.47)	74.26 (73.76-74.72)	65.02 (62.29-67.47)	12.32 (11.18-13.31)	75.02 (74.51-75.46)	65.76 (63.01-68.24)	12.27 (11.14-13.28)	77.59 (76.50-78.82)	67.77 (64.90-70.55)	13.35 (11.96-14.70)
Bosnia and Herzegovina	72.14 (71.01-73.34)	63.13 (60.36-65.77)	10.72 (9.64-11.81)	74.87 (73.74-75.96)	65.38 (62.50-68.04)	11.69 (10.49-12.87)	75.95 (74.87-76.96)	66.19 (63.36-68.83)	11.93 (10.73-13.11)	77.55 (76.17-79.00)	67.30 (64.08-70.16)	12.62 (11.21-13.95)
Bulgaria	71.37 (71.07-71.67)	62.42 (59.79-64.73)	10.33 (9.33-11.26)	71.67 (71.35-71.99)	62.96 (60.40-65.25)	10.57 (9.56-11.48)	72.70 (72.39-73.03)	63.82 (61.22-66.12)	10.98 (9.92-11.93)	75.03 (73.50-76.58)	65.60 (62.61-68.43)	11.90 (10.56-13.23)
Croatia	72.22 (71.45-72.97)	63.18 (60.53-65.63)	10.70 (9.56-11.74)	73.91 (73.33-74.59)	64.77 (62.06-67.19)	11.26 (10.12-12.33)	75.36 (74.78-75.99)	65.87 (63.09-68.40)	11.69 (10.47-12.79)	77.36 (76.24-78.65)	67.42 (64.47-70.16)	12.48 (11.10-13.73)
Czech Republic	71.80 (71.58-72.01)	62.46 (59.68-64.79)	9.79 (8.68-10.75)	75.03 (74.82-75.25)	65.22 (62.30-67.73)	11.21 (9.99-12.28)	76.55 (76.32-76.77)	66.19 (63.21-68.86)	11.83 (10.54-12.98)	79.10 (78.65-79.58)	68.10 (64.81-70.94)	12.98 (11.50-14.25)
Hungary	69.49 (68.96-70.06)	60.47 (57.79-62.80)	9.85 (8.70-10.88)	71.80 (71.23-72.33)	62.61 (59.95-65.01)	10.76 (9.59-11.84)	73.37 (72.80-73.94)	63.93 (61.19-66.47)	11.36 (10.14-12.50)	75.75 (74.70-76.84)	65.82 (62.90-68.68)	12.12 (10.78-13.47)
Macedonia	70.50 (69.91-71.06)	61.98 (59.49-64.30)	10.11 (9.12-11.05)	72.18 (71.71-72.66)	63.57 (61.04-65.89)	10.08 (9.07-10.98)	73.07 (72.59-73.58)	64.28 (61.71-66.64)	10.34 (9.36-11.26)	74.82 (74.16-75.48)	65.59 (62.79-68.04)	11.15 (10.04-12.13)
Montenegro	75.04 (74.18-75.86)	65.42 (62.46-68.03)	12.50 (11.19-13.66)	73.82 (73.27-74.38)	64.61 (61.88-67.00)	11.67 (10.51-12.72)	75.27 (74.74-75.79)	65.78 (62.91-68.25)	12.14 (10.90-13.24)	77.04 (76.15-77.97)	67.13 (64.10-69.95)	12.72 (11.36-14.03)
Poland	70.90 (70.56-71.29)	61.91 (59.23-64.23)	10.54 (9.43-11.50)	73.77 (73.40-74.12)	64.38 (61.65-66.85)	11.50 (10.33-12.57)	75.30 (74.95-75.66)	65.55 (62.61-68.03)	12.24 (10.93-13.39)	77.93 (77.05-78.78)	67.60 (64.59-70.41)	13.34 (11.96-14.68)
Romania	69.84 (69.33-70.34)	60.83 (58.09-63.24)	10.55 (9.47-11.53)	71.12 (70.61-71.65)	62.12 (59.48-64.53)	10.98 (9.87-11.97)	72.79 (72.28-73.24)	63.54 (60.80-65.95)	11.41 (10.25-12.45)	75.23 (74.24-76.18)	65.54 (62.68-68.19)	12.22 (10.98-13.44)
Serbia	72.58 (71.74-73.49)	63.38 (60.63-65.90)	10.96 (9.79-12.09)	72.13 (71.53-72.76)	63.18 (60.58-65.52)	10.44 (9.42-11.40)	73.54 (73.02-74.12)	64.32 (61.61-66.77)	10.83 (9.77-11.82)	75.90 (75.35-76.47)	66.07 (63.14-68.61)	12.11 (10.85-13.20)
Slovakia	70.95 (70.52-71.38)	61.99 (59.28-64.27)	10.18 (9.10-11.11)	73.25 (72.82-73.66)	63.94 (61.21-66.40)	10.95 (9.84-11.98)	74.42 (74.03-74.86)	64.93 (62.09-67.42)	11.46 (10.28-12.52)	76.97 (75.87-78.03)	66.88 (63.94-69.84)	12.57 (11.22-13.89)
Slovenia	73.64 (72.87-74.38)	63.76 (60.86-66.41)	11.03 (9.80-12.20)	76.03 (75.31-76.78)	65.75 (62.75-68.46)	12.01 (10.72-13.30)	77.93 (77.21-78.76)	67.05 (63.80-70.03)	12.71 (11.24-14.09)	80.84 (79.77-82.10)	69.32 (65.90-72.55)	13.89 (12.19-15.49)
Central Asia	67.27 (66.82-67.71)	58.96 (56.46-61.19)	10.99 (9.97-11.92)	66.13 (65.61-66.64)	58.26 (55.95-60.39)	10.35 (9.43-11.22)	67.10 (66.45-67.74)	59.15 (56.79-61.35)	10.32 (9.38-11.18)	71.31 (70.44-72.18)	62.58 (59.94-65.12)	11.52 (10.40-12.55)
Armenia	70.33 (69.61-71.03)	61.64 (59.05-63.87)	11.51 (10.37-12.48)	71.96 (71.27-72.65)	63.23 (60.67-65.54)	11.57 (10.48-12.57)	72.43 (71.64-73.13)	63.71 (61.08-66.03)	11.39 (10.32-12.38)	75.85 (74.83-76.91)	66.47 (63.68-68.96)	12.57 (11.31-13.71)
Azerbaijan	66.32 (65.13-67.51)	58.43 (56.00-60.84)	11.00 (9.98-11.95)	66.54 (65.39-67.55)	58.88 (56.55-61.14)	10.37 (9.41-11.27)	67.96 (66.78-69.14)	60.13 (57.62-62.55)	10.41 (9.45-11.37)	72.03 (70.14-73.97)	63.40 (60.58-66.04)	11.79 (10.58-13.07)
Georgia	69.70 (68.77-70.57)	61.49 (58.81-63.75)	11.41 (10.26-12.41)	71.81 (70.93-72.74)	63.46 (60.99-65.71)	11.85 (10.82-12.88)	72.45 (71.63-73.24)	63.95 (61.40-66.24)	11.94 (10.88-12.93)	73.92 (71.97-75.72)	65.01 (61.94-67.73)	12.31 (11.05-13.58)
Kazakhstan	67.92 (66.88-69.06)	59.36 (56.64-61.75)	10.81 (9.66-11.85)	64.42 (63.16-65.62)	56.70 (54.18-59.01)	9.82 (8.83-10.82)	65.05 (63.79-66.32)	57.25 (54.76-59.61)	9.74 (8.69-10.68)	71.75 (69.69-73.67)	62.67 (59.52-65.68)	11.43 (10.06-12.75)
Kyrgyzstan	66.05 (65.51-66.63)	57.71 (55.27-59.87)	10.83 (9.79-11.77)	66.32 (65.73-66.88)	58.22 (55.81-60.38)	10.50 (9.55-11.40)	66.50 (65.98-67.05)	58.62 (56.26-60.72)	9.89 (8.99-10.72)	71.24 (70.23-72.29)	62.45 (59.72-64.92)	11.51 (10.38-12.59)
Mongolia	62.44 (61.26-63.56)	55.07 (52.70-57.24)	10.01 (9.00-10.97)	62.70 (61.81-63.67)	55.53 (53.26-57.48)	9.18 (8.30-9.95)	64.24 (63.29-65.27)	56.83 (54.51-59.01)	9.41 (8.50-10.28)	68.12 (66.52-69.76)	59.91 (57.09-62.58)	10.51 (9.39-11.58)
Tajikistan	64.96 (63.54-66.31)	57.01 (54.58-59.44)	11.21 (10.13-12.26)	65.16 (63.84-66.44)	57.45 (54.92-59.73)	10.33 (9.28-11.29)	67.93 (66.69-69.12)	59.93 (57.32-62.27)	10.46 (9.45-11.40)	71.76 (70.11-73.35)	63.09 (60.25-65.71)	12.02 (10.79-13.15)
Turkmenistan	63.73 (62.59-64.86)	56.25 (53.75-58.45)	10.66 (9.68-11.51)	63.62 (62.23-64.80)	56.38 (53.93-58.63)	10.58 (9.66-11.43)	65.76 (64.44-66.98)	58.25 (55.74-60.54)	10.95 (9.96-11.85)	70.24 (69.02-71.31)	62.06 (59.43-64.45)	11.81 (10.71-12.84)
Uzbekistan	68.35 (67.58-69.18)	59.76 (57.15-62.07)	11.03 (9.97-11.96)	66.33 (65.50-67.15)	58.40 (55.92-60.68)	9.89 (8.96-10.73)	67.06 (65.80-68.18)	59.11 (56.69-61.51)	9.96 (9.05-10.85)	70.29 (68.99-71.65)	61.80 (59.09-64.37)	11.00 (9.90-12.04)
Latin America and Caribbean	69.50 (69.26-69.70)	60.98 (58.40-63.19)	12.12 (11.05-13.06)	72.41 (72.20-72.63)	63.45 (60.80-65.78)	12.80 (11.66-13.82)	73.92 (73.69-74.12)	64.74 (62.06-67.15)	13.25 (12.06-14.30)	75.80 (75.36-76.18)	66.36 (63.47-68.76)	13.81 (12.57-14.90)
Central Latin America	71.14 (70.89-71.40)	62.65 (60.04-64.85)	12.82 (11.65-13.82)	73.85 (73.61-74.09)	65.03 (62.40-67.34)	13.49 (12.27-14.55)	75.01 (74.76-75.26)	66.09 (63.46-68.43)	13.71 (12.51-14.77)	76.49 (76.02-76.95)	67.29 (64.52-69.67)	14.14 (12.89-15.27)
Colombia	71.32 (70.91-71.71)	63.10 (60.52-65.30)	12.83 (11.69-13.80)	73.19 (72.83-73.58)	64.76 (62.26-66.98)	13.23 (12.11-14.22)	75.18 (74.80-75.55)	66.52 (63.92-68.84)	13.48 (12.36-14.44)	78.26 (77.33-79.17)	69.09 (66.28-71.60)	14.67 (13.36-15.88)
Costa Rica	76.52 (76.09-76.98)	67.59 (64.92-69.95)	13.55 (12.40-14.60)	77.96 (77.54-78.35)	68.77 (66.11-71.20)	14.31 (13.10-15.43)	79.59 (79.15-80.01)	70.05 (67.17-72.60)	15.25 (13.89-16.46)	80.98 (80.24-81.73)	71.16 (68.19-73.83)	16.02 (14.55-17.39)
El Salvador	68.13 (67.49-68.82)	60.12 (57.73-62.36)	12.60 (11.47-13.58)	72.25 (71.61-72.88)	63.71 (61.13-65.90)	13.54 (12.35-14.55)	73.48 (72.92-74.11)	64.76 (62.15-67.14)	13.90 (12.67-15.01)	75.47 (74.17-76.77)	66.48 (63.53-69.05)	14.40 (13.04-15.66)
Guatemala	64.61 (63.27-66.13)	56.69 (54.09-59.13)	11.49 (10.35-12.58)	68.64 (66.96-70.32)	60.10 (57.05-62.75)	12.50 (11.10-13.74)	70.19 (68.41-71.84)	61.48 (58.63-64.29)	13.01 (11.68-14.32)	72.75 (69.97-75.66)	63.86 (60.52-67.30)	13.54 (12.06-15.20)
Honduras	67.20 (65.34-68.79)	59.47 (57.08-61.94)	11.35 (10.34-12.41)	69.55 (67.13-72.15)	61.59 (58.65-64.55)	11.51 (10.22-12.88)	70.83 (68.12-73.96)	62.71 (59.54-66.02)	11.73 (10.37-13.33)	72.67 (69.75-75.42)	64.27 (61.01-67.39)	12.20 (10.75-13.65)
Mexico	71.51 (71.19-71.84)	62.78 (60.21-65.06)	12.99 (11.80-14.05)	74.61 (74.29-74.89)	65.53 (62.82-67.89)	13.71 (12.45-14.82)	75.46 (75.15-75.74)	66.36 (63.67-68.69)	13.78 (12.54-14.89)	76.38 (75.96-76.77)	67.06 (64.25-69.43)	13.87 (12.58-14.97)
Nicaragua	73.64 (73.13-74.10)	64.61 (62.00-67.00)	15.05 (13.73-16.26)	75.67 (75.22-76.15)	66.61 (63.97-69.07)	14.91 (13.60-16.09)	76.92 (76.46-77.39)	67.77 (65.06-70.26)	14.96 (13.66-16.14)	78.27 (76.72-79.73)	68.92 (65.82-71.70)	15.16 (13.67-16.60)
Panama	74.91 (74.10-75.71)	66.18 (63.59-68.58)	13.44 (12.25-14.56)	76.76 (76.05-77.44)	67.72 (65.00-70.24)	14.47 (13.14-15.68)	77.45 (76.74-78.19)	68.29 (65.52-70.69)	14.82 (13.50-15.97)	78.92 (77.52-80.17)	69.38 (66.27-72.22)	15.46 (13.92-16.86)
Venezuela	72.62 (71.95-73.30)	64.25 (61.66-66.51)	12.41 (11.30-13.42)	74.19 (73.52-74.91)	65.56 (62.95-67.88)	13.31 (12.09-14.38)	74.89 (74.15-75.67)	66.16 (63.47-68.49)	13.86 (12.61-14.96)	75.43 (73.29-77.11)	66.56 (63.66-69.31)	14.22 (12.82-15.60)
Andean Latin America	68.25 (67.74-68.78)	59.84 (57.38-62.02)	12.59 (11.45-13.58)	73.17 (72.73-73.61)	64.15 (61.48-66.58)	13.54 (12.34-14.66)	75.59 (75.09-76.08)	66.22 (63.42-68.62)	14.28 (13.00-15.39)	77.90 (76.78-78.94)	68.21 (65.15-70.89)	14.75 (13.29-16.03)
Bolivia	61.33 (60.29-62.38)	53.56 (51.08-55.84)	10.39 (9.33-11.41)	67.65 (66.85-68.38)	59.09 (56.44-61.50)	11.66 (10.54-12.75)	70.45 (69.31-71.49)	61.49 (58.66-63.92)	12.28 (11.07-13.37)	73.24 (71.14-75.30)	64.00 (60.97-66.91)	12.71 (11.29-14.09)
Ecuador	71.38 (70.91-71.83)	62.66 (60.03-65.00)	13.17 (11.99-14.24)	74.21 (73.73-74.72)	65.10 (62.32-67.56)	14.05 (12.80-15.20)	75.78 (75.25-76.27)	66.45 (63.68-68.89)	14.54 (13.19-15.70)	77.83 (77.07-78.63)	68.20 (65.40-70.74)	15.09 (13.74-16.32)
Peru	69.42 (68.61-70.19)	60.89 (58.35-63.16)	13.21 (12.07-14.28)	74.81 (74.08-75.57)	65.64 (62.82-68.18)	14.02 (12.78-15.17)	77.52 (76.72-78.25)	67.95 (65.05-70.50)	14.91 (13.58-16.08)	79.74 (77.91-81.39)	69.84 (66.51-72.86)	15.40 (13.82-16.97)
Caribbean	67.69 (67.10-68.25)	59.34 (56.81-61.68)	12.19 (11.13-13.15)	69.66 (69.06-70.29)	61.01 (58.34-63.38)	12.76 (11.64-13.77)	70.53 (69.78-71.25)	61.73 (59.11-64.18)	13.10 (11.94-14.13)	73.16 (71.98-74.27)	63.97 (61.11-66.66)	13.46 (12.30-14.56)
Antigua and Barbuda	73.84 (72.92-74.70)	64.83 (62.13-67.30)	12.13 (11.04-13.13)	74.83 (73.95-75.68)	65.51 (62.66-68.02)	13.13 (11.91-14.27)	76.07 (75.23-76.95)	66.48 (63.52-69.15)	13.53 (12.25-14.76)	77.36 (76.12-78.52)	67.53 (64.44-70.29)	14.04 (12.67-15.36)

Appendix Table 2. GBD 2016 Life expectancy at birth and HALE at birth and at age 65 by location for GBD years 1990, 2000, 2006 & 2016, both sexes combined.

Location	1990			2000			2006			2016		
	Life Expectancy	HALE		Life Expectancy	HALE		Life Expectancy	HALE		Life Expectancy	HALE	
	At birth	At birth	At age 65	At birth	At birth	At age 65	At birth	At birth	At age 65	At birth	At birth	At age 65
The Bahamas	70.53 (69.50-71.34)	62.06 (59.45-64.43)	11.99 (10.97-12.93)	71.30 (70.69-71.89)	62.76 (60.22-65.07)	12.15 (11.10-13.13)	73.39 (72.75-74.02)	64.46 (61.86-66.87)	12.94 (11.79-13.97)	73.86 (72.52-75.14)	64.88 (62.01-67.47)	12.91 (11.68-14.09)
Barbados	73.70 (72.69-74.54)	64.75 (62.07-67.21)	12.21 (11.12-13.24)	74.96 (73.97-75.89)	65.65 (62.82-68.20)	13.22 (12.00-14.35)	76.12 (75.28-76.85)	66.56 (63.64-69.22)	13.75 (12.49-14.86)	76.62 (75.41-77.68)	66.92 (63.78-69.63)	13.86 (12.55-15.04)
Belize	71.57 (70.48-72.48)	62.77 (59.87-65.22)	12.61 (11.42-13.67)	68.63 (67.65-69.52)	60.40 (57.84-62.71)	10.98 (10.01-11.89)	70.16 (69.33-71.00)	61.64 (59.01-63.95)	11.24 (10.18-12.23)	71.84 (70.33-73.40)	63.05 (60.13-65.84)	11.68 (10.54-12.86)
Bermuda	69.69 (68.87-70.46)	61.61 (59.07-63.89)	10.18 (9.23-11.05)	72.51 (71.82-73.23)	64.07 (61.56-66.46)	10.59 (9.67-11.50)	75.33 (74.59-76.03)	66.35 (63.71-68.83)	12.18 (11.15-13.19)	78.93 (77.73-80.15)	69.25 (66.26-71.96)	14.10 (12.83-15.38)
Cuba	74.77 (74.37-75.18)	65.64 (62.92-68.09)	13.06 (11.93-14.09)	76.52 (76.16-76.95)	67.26 (64.50-69.75)	13.64 (12.43-14.72)	77.76 (77.38-78.17)	68.31 (65.46-70.76)	13.98 (12.75-15.05)	78.91 (78.16-79.70)	69.19 (66.32-71.87)	14.40 (13.09-15.63)
Dominica	72.04 (71.23-72.85)	63.52 (60.92-65.82)	11.06 (10.07-11.99)	72.86 (72.08-73.66)	64.12 (61.50-66.56)	11.61 (10.56-12.60)	73.34 (72.45-74.17)	64.52 (61.83-66.84)	11.95 (10.91-12.90)	72.92 (71.33-74.46)	64.07 (61.09-66.71)	12.28 (11.06-13.42)
Dominican Republic	70.74 (70.04-71.51)	62.34 (59.72-64.75)	12.96 (11.83-14.04)	73.05 (72.21-74.15)	64.22 (61.44-66.77)	13.67 (12.45-14.85)	73.49 (72.50-74.79)	64.54 (61.79-67.08)	13.95 (12.65-15.29)	75.64 (74.26-77.28)	66.41 (63.44-69.27)	14.24 (12.92-15.62)
Grenada	70.93 (69.54-72.12)	61.91 (59.06-64.49)	11.31 (10.24-12.29)	71.25 (70.23-72.29)	62.46 (59.80-64.79)	11.52 (10.46-12.48)	71.14 (70.02-72.20)	62.37 (59.66-64.91)	11.44 (10.36-12.41)	71.56 (69.97-73.30)	62.69 (59.74-65.40)	11.59 (10.44-12.70)
Guyana	64.18 (63.37-64.96)	56.21 (53.82-58.49)	10.10 (9.16-10.96)	65.43 (64.66-66.28)	57.07 (54.52-59.34)	10.86 (9.82-11.79)	64.90 (64.04-65.74)	56.72 (54.22-58.98)	10.12 (9.16-11.00)	67.74 (66.37-69.16)	59.17 (56.37-61.70)	10.69 (9.61-11.72)
Haiti	53.99 (52.45-55.53)	47.07 (44.61-49.43)	8.71 (7.90-9.54)	56.75 (55.03-58.60)	49.47 (46.91-52.06)	9.33 (8.40-10.19)	58.29 (56.38-60.50)	50.91 (48.21-53.66)	9.66 (8.69-10.68)	63.97 (61.34-66.72)	55.90 (52.79-59.00)	10.18 (9.06-11.44)
Jamaica	74.43 (73.29-75.44)	65.03 (62.17-67.67)	13.34 (12.13-14.46)	73.82 (72.79-74.82)	64.48 (61.67-67.13)	12.99 (11.81-14.10)	74.83 (73.68-75.83)	65.29 (62.25-67.97)	13.29 (12.05-14.41)	74.90 (73.09-76.71)	65.34 (62.11-68.27)	12.99 (11.61-14.36)
Puerto Rico	73.96 (73.39-74.54)	64.90 (62.19-67.28)	13.36 (12.22-14.40)	76.06 (75.55-76.64)	66.32 (63.32-68.91)	14.07 (12.75-15.21)	77.33 (76.87-77.83)	67.33 (64.29-69.98)	14.45 (13.09-15.68)	78.77 (77.80-79.68)	68.62 (65.55-71.33)	14.82 (13.39-16.06)
Saint Lucia	71.87 (70.85-72.68)	62.44 (59.60-65.04)	12.18 (11.08-13.20)	74.17 (73.40-74.82)	64.71 (61.91-67.33)	13.49 (12.29-14.63)	74.42 (73.56-75.14)	65.05 (62.18-67.52)	13.49 (12.30-14.60)	76.13 (74.91-77.08)	66.38 (63.17-69.00)	14.25 (12.95-15.44)
Saint Vincent and the Grenadines	70.65 (69.64-71.58)	61.99 (59.25-64.31)	11.35 (10.28-12.32)	70.15 (69.34-70.90)	61.49 (58.74-63.83)	11.21 (10.13-12.19)	71.18 (70.53-71.84)	62.35 (59.77-64.74)	11.43 (10.39-12.44)	71.64 (70.58-72.64)	62.70 (59.98-65.28)	11.54 (10.42-12.63)
Suriname	70.08 (69.46-70.62)	61.31 (58.59-63.70)	12.33 (11.24-13.32)	69.64 (68.99-70.27)	60.92 (58.26-63.33)	11.83 (10.71-12.83)	69.51 (68.80-70.24)	60.80 (58.05-63.14)	11.59 (10.51-12.58)	71.34 (70.30-72.36)	62.24 (59.44-64.73)	12.09 (10.96-13.22)
Trinidad and Tobago	69.00 (68.44-69.54)	60.48 (57.96-62.77)	10.59 (9.62-11.48)	69.41 (68.81-69.98)	60.76 (58.20-63.12)	11.00 (9.94-11.89)	71.56 (70.93-72.25)	62.40 (59.62-64.82)	11.91 (10.77-12.95)	73.02 (71.73-74.18)	63.51 (60.50-66.13)	12.44 (11.22-13.59)
Virgin Islands, U.S.	72.60 (71.75-73.70)	64.07 (61.44-66.50)	12.05 (10.99-13.08)	73.35 (72.55-74.00)	64.61 (61.92-66.95)	12.34 (11.24-13.32)	73.83 (73.01-74.49)	64.97 (62.26-67.32)	12.46 (11.34-13.44)	74.62 (73.38-76.19)	65.54 (62.60-68.36)	12.68 (11.46-13.85)
Tropical Latin America	68.55 (68.11-69.00)	59.90 (57.22-62.18)	11.25 (10.23-12.15)	71.44 (71.00-71.85)	62.24 (59.50-64.69)	11.95 (10.87-12.92)	73.22 (72.79-73.59)	63.69 (60.86-66.20)	12.59 (11.44-13.64)	75.22 (74.73-75.66)	65.46 (62.51-67.97)	13.38 (12.15-14.49)
Brazil	68.41 (67.96-68.87)	59.78 (57.10-62.06)	11.20 (10.18-12.10)	71.36 (70.91-71.79)	62.17 (59.42-64.62)	11.91 (10.84-12.89)	73.20 (72.76-73.58)	63.67 (60.83-66.17)	12.58 (11.43-13.63)	75.24 (74.75-75.69)	65.48 (62.53-67.99)	13.39 (12.16-14.50)
Paraguay	74.59 (74.07-75.11)	65.09 (62.30-67.60)	13.38 (12.19-14.53)	74.37 (73.85-74.89)	64.92 (62.23-67.39)	13.21 (12.00-14.25)	74.06 (73.39-74.66)	64.63 (61.80-67.15)	12.92 (11.66-14.04)	74.48 (73.28-75.58)	64.96 (61.94-67.70)	12.73 (11.45-13.99)
Southeast Asia, East Asia, and Oceania	66.54 (66.19-66.89)	59.21 (57.07-61.12)	10.47 (9.67-11.24)	69.14 (68.88-69.38)	61.53 (59.35-63.49)	11.00 (10.12-11.79)	71.72 (71.51-71.93)	63.73 (61.39-65.80)	11.74 (10.78-12.61)	75.05 (74.79-75.30)	66.44 (63.95-68.59)	12.82 (11.78-13.77)
East Asia	67.16 (66.64-67.62)	60.01 (57.91-61.88)	10.46 (9.69-11.20)	69.82 (69.45-70.17)	62.42 (60.28-64.34)	11.05 (10.20-11.82)	72.89 (72.65-73.14)	65.03 (62.71-67.08)	11.91 (10.97-12.77)	76.29 (76.03-76.55)	67.78 (65.36-69.95)	13.07 (12.04-14.02)
China	66.96 (66.44-67.44)	59.85 (57.76-61.70)	10.42 (9.66-11.15)	69.94 (69.63-70.25)	62.55 (60.41-64.48)	11.02 (10.18-11.79)	72.88 (72.63-73.13)	65.05 (62.73-67.09)	11.88 (10.94-12.73)	76.36 (76.12-76.62)	67.85 (65.44-70.01)	13.06 (12.03-14.00)
North Korea	71.95 (70.79-73.26)	63.96 (61.35-66.32)	12.02 (10.91-13.14)	63.83 (58.15-69.35)	56.76 (51.80-61.69)	10.47 (9.26-11.68)	69.99 (69.07-71.03)	62.12 (59.69-64.38)	11.48 (10.47-12.49)	70.85 (69.94-71.75)	63.01 (60.62-65.28)	11.64 (10.69-12.58)
Taiwan, Province of China	73.94 (73.52-74.37)	65.33 (62.76-67.66)	11.87 (10.85-12.86)	76.10 (75.68-76.51)	67.30 (64.75-69.67)	13.07 (11.95-14.13)	77.85 (77.45-78.26)	68.69 (66.09-70.96)	13.99 (12.84-15.05)	79.66 (78.45-81.03)	70.14 (67.38-72.72)	14.84 (13.51-16.12)
Southeast Asia	65.32 (64.95-65.69)	57.45 (55.22-59.49)	10.55 (9.60-11.43)	67.90 (67.54-68.23)	59.72 (57.34-61.79)	10.87 (9.88-11.74)	69.62 (69.24-69.98)	61.25 (58.88-63.38)	11.23 (10.23-12.13)	72.70 (72.24-73.11)	63.87 (61.32-66.08)	12.02 (10.95-12.99)
Cambodia	57.39 (56.53-58.33)	49.86 (47.47-51.93)	8.66 (7.77-9.49)	59.53 (58.60-60.43)	51.69 (49.32-53.77)	8.93 (8.01-9.78)	63.83 (63.00-64.63)	55.59 (53.21-57.83)	9.56 (8.62-10.46)	68.75 (67.82-69.68)	60.12 (57.55-62.38)	10.46 (9.44-11.40)
Indonesia	63.62 (63.22-64.00)	56.14 (53.92-58.11)	10.21 (9.27-11.04)	67.24 (66.89-67.59)	59.31 (56.98-61.35)	10.56 (9.61-11.43)	68.79 (68.38-69.18)	60.65 (58.25-62.71)	10.82 (9.83-11.71)	71.66 (71.04-72.25)	62.95 (60.34-65.11)	11.36 (10.28-12.31)
Laos	52.42 (50.98-53.81)	46.06 (43.72-48.18)	8.69 (7.83-9.50)	57.03 (55.78-58.16)	50.14 (47.81-52.20)	9.25 (8.34-10.09)	60.92 (59.75-61.98)	53.55 (51.16-55.70)	9.79 (8.83-10.64)	67.25 (65.60-68.58)	59.08 (56.36-61.53)	10.75 (9.70-11.69)
Malaysia	71.57 (71.33-71.80)	63.15 (60.69-65.30)	10.72 (9.77-11.57)	73.01 (72.81-73.25)	64.45 (62.03-66.64)	11.00 (10.02-11.87)	73.98 (73.77-74.19)	65.22 (62.66-67.41)	11.35 (10.35-12.23)	75.56 (75.08-76.05)	66.42 (63.81-68.80)	12.11 (11.08-13.08)
Maldives	62.46 (61.66-63.36)	54.40 (52.02-56.66)	9.94 (8.97-10.87)	72.12 (71.34-72.85)	62.90 (60.18-65.25)	11.04 (10.00-11.99)	76.26 (75.55-77.00)	66.50 (63.60-69.11)	12.33 (11.15-13.42)	79.32 (77.48-81.07)	69.13 (65.79-72.12)	13.61 (12.04-15.10)
Mauritius	69.25 (68.87-69.66)	60.72 (58.23-62.88)	9.85 (8.95-10.70)	71.39 (70.97-71.78)	62.52 (59.98-64.82)	10.85 (9.85-11.77)	72.47 (72.04-72.90)	63.28 (60.59-65.64)	11.28 (10.22-12.24)	74.58 (73.19-75.92)	64.86 (61.68-67.66)	12.18 (10.85-13.47)
Myanmar	58.57 (56.96-60.14)	51.31 (48.87-53.75)	8.86 (7.95-9.75)	61.10 (59.30-62.71)	53.71 (51.08-56.11)	9.29 (8.31-10.20)	64.26 (62.22-65.69)	56.51 (53.80-58.89)	9.89 (8.83-10.86)	70.02 (68.84-71.07)	61.56 (59.01-63.99)	10.91 (9.88-11.89)
Philippines	67.03 (66.41-67.69)	58.63 (56.19-60.87)	10.95 (9.92-11.88)	68.05 (67.49-68.67)	59.52 (57.03-61.78)	10.29 (9.30-11.19)	68.16 (67.50-68.81)	59.77 (57.33-61.93)	10.17 (9.25-11.08)	70.10 (68.69-71.50)	61.64 (59.12-64.15)	10.68 (9.66-11.76)
Sri Lanka	71.29 (70.54-72.05)	62.43 (59.82-64.84)	12.16 (11.02-13.21)	72.51 (71.77-73.36)	63.60 (60.95-66.05)	12.26 (11.11-13.36)	74.25 (73.36-75.09)	65.11 (62.46-67.47)	12.49 (11.30-13.57)	77.41 (75.24-79.70)	67.75 (64.58-70.84)	13.53 (11.99-15.13)
Seychelles	69.03 (68.31-69.76)	61.21 (58.94-63.19)	10.92 (9.96-11.76)	70.91 (70.25-71.52)	62.71 (60.31-64.84)	11.40 (10.39-12.33)	72.31 (71.65-73.00)	63.85 (61.33-66.02)	11.77 (10.68-12.73)	73.68 (72.25-74.87)	64.95 (62.37-67.41)	12.18 (11.07-13.25)
Thailand	70.56 (69.97-71.12)	61.89 (59.41-64.16)	11.93 (10.80-12.92)	71.89 (71.17-72.53)	62.98 (60.44-65.38)	12.76 (11.57-13.87)	74.60 (74.09-75.11)	65.41 (62.79-67.78)	13.32 (12.12-14.40)	77.70 (76.71-78.82)	67.94 (65.07-70.60)	14.51 (13.15-15.86)
Timor-Leste	57.90 (55.82-60.00)	50.34 (47.55-52.97)	9.95 (8.79-11.10)	63.64 (62.04-65.33)	55.34 (52.64-57.95)	10.59 (9.55-11.63)	68.30 (66.65-70.13)	59.33 (56.42-62.20)	11.19 (10.00-12.38)	72.67 (70.35-75.01)	63.29 (60.10-66.61)	12.07 (10.72-13.47)
Vietnam	68.23 (66.76-69.72)	60.15 (57.55-62.64)	10.94 (9.82-12.10)	70.75 (69.54-72.19)	62.44 (59.92-64.81)	11.20 (10.18-12.23)	72.06 (70.71-73.48)	63.68 (61.25-66.17)	11.57 (10.52-12.75)	74.55 (73.40-75.64)	65.91 (63.25-68.33)	12.51 (11.36-13.56)
Oceania	58.46 (56.65-60.13)	51.26 (48.64-53.69)	8.11 (7.24-8.92)	59.59 (57.51-61.55)	52.19 (49.40-54.65)	8.24 (7.35-9.08)	59.69 (57.38-61.81)	52.27 (49.31-54.94)	8.23 (7.36-9.12)	62.19 (59.79-64.41)	54.34 (51.28-57.09)	8.64 (7.69-9.53)
American Samoa	70.48 (69.37-71.59)	61.20 (58.40-63.80)	10.57 (9.46-11.59)	71.53 (70.37-72.68)	61.94 (58.98-64.63)	10.87 (9.67-11.96)	71.73 (70.49-72.78)	62.15 (59.22-64.79)	10.99 (9.82-12.14)	72.39 (70.52-74.25)	62.64 (59.54-65.50)	11.08 (9.76-12.32)
Federated States of Micronesia	63.20 (60.87-65.33)	55.54 (52.67-58.16)	8.92 (7.93-9.94)	64.71 (62.43-67.04)	56.79 (53.96-59.59)	9.27 (8.27-10.31)	65.58 (63.13-67.95)	57.59 (54.57-60.39)	9.29 (8.15-10.38)	65.50 (62.53-68.30)	57.51 (54.22-60.64)	9.21 (7.96-10.46)
Fiji	65.20 (62.46-67.88)	56.99 (53.82-60.20)	9.19 (8.05-10.37)	64.18 (62.50-65.67)	56.02 (53.24-58.60)	8.83 (7.79-9.82)	64.74 (63.26-66.31)	56.55 (53.78-59.05)	8.87 (7.84-9.88)	65.38 (62.35-68.63)	57.21 (53.90-60.34)	9.23 (8.03-10.51)
Guam	73.09 (72.13-73.96)	64.27 (61.61-66.62)	11.80 (10.69-12.79)	73.89 (72.89-74.73)	64.86 (62.12-67.33)	12.00 (10.86-13.06)	73.20 (72.10-73.98)	64.22 (61.48-66.67)	11.78 (10.63-12.83)	72.34 (70.71-73.88)	63.31 (60.39-65.94)	11.47 (10.28-12.60)
Kiribati	58.14 (56.41-59.79)	51.05 (48.51-53.41)	8.00 (7.18-8.81)	59.37 (58.11-60.60)	52.01 (49.52-54.18)	8.17 (7.30-8.98)	60.18 (58.33-61.86)	52.65 (49.90-55.19)	8.36 (7.43-9.24)	61.79 (59.09-64.15)	54.02 (50.86-56.94)	8.61 (7.58-9.65)
Marshall Islands	64.50 (63.19-65.65)	56.23 (53.65-58.65)	9.08 (8.15-9.97)	63.21 (61.64-64.75)	55.04 (52.22-57.57)	8.64 (7.65-9.62)	62.97 (61.40-64.44)	54.86 (52.20-57.42)	8.43 (7.46-9.34)	64.92 (62.93-67.01)	56.40 (53.40-59.27)	8.87 (7.75-9.95)
Northern Mariana Islands	73.64 (71.56-75.88)	64.60 (61.44-67.62)	11.66 (10.36-12.95)	76.14 (74.59-77.77)	66.58 (63.40-69.30)	12.57 (11.20-13.85)	76.18 (74.61-77.77)	66.58 (63.39-69.20)	12.55 (11.16-13.74)	75.47 (73.77-77.13)	65.86 (62.67-68.60)	12.31 (10.89-13.59)
Papua New Guinea	55.93 (53.61-58.17)	49.06 (46.28-51.55)	7.54 (6.66-8.39)	57.60 (54.84-60.19)	50.47 (47.30-53.23)	7.73 (6.79-8.61)	57.71 (54.75-60.40)	50.57 (47.48-53.51)	7.72 (6.80-8.68)	60.79 (57.85-63.60)	53.13 (49.77-56.15)	8.23 (7.20-9.21)
Samoa	68.89 (67.15-70.52)	60.37 (57.51-63.01)	10.37 (9.29-11.39)	70.30 (68.64-71.97)	61.55 (58.73-64.29)	10.75 (9.58-11.87)	71.20 (69.77-72.85)	62.30 (59.50-65.00)	10.99 (9.78-12.20)	71.89 (70.33-73.60)	62.87 (59.92-65.64)	11.16 (9.97-12.34)
Solomon Islands	60.07 (57.92-62.57)	52.93 (50.32-55.76)	8.03 (7.21-8.98)	61.40 (59.30-63.69)	54.02 (51.41-56.71)	8.22 (7.33-9.17)	61.07 (58.80-63.44)	53.70 (50.88-56.42)	8.10 (7.15-9.03)	63.04 (60.41-65.53)	55.30 (52.43-58.00)	8.49 (7.49-9.47)
Tonga	67.87 (65.99-69.72)	59.28 (56.30-62.15)	9.94 (8.87-11.10)	68.72 (66.93-70.34)	59.99 (56.97-62.75)	10.22 (9.12-11.29)	69.61 (67.92-71.12)	60.67 (57.62-63.32)	10.50 (9.37-11.61)	70.39 (68.52-72.31)	61.22 (58.18-64.00)	10.73 (9.52-11.92)
Vanuatu	61.68 (59.67-63.71)	54.15 (51.24-57.00)	8.40 (7.49-9.33)	62.28 (60.28-64.22)	54.70 (51.94-57.41)	8.54 (7.58-9.50)	62.29 (59.97-64.49)	54.73 (51.92-57.48)	8.56 (7.61-9.51)	63.90 (61.20-66.34)	55.99 (52.79-59.05)	8.89 (7.82-9.95)
North Africa and Middle East	66.92 (66.48-67.34)	57.01 (54.17-59.60)	10.95 (9.79-12.04)	69.72 (69.37-70.05)	59.56 (56.63-62.20)	11.28 (10.12-12.36)	71.10 (70.63-71.55)	60.78 (57.72-63.44)	11.53 (10.29-12.65)	73.16 (72.48-73.80)	62.50 (59.45-65.21)	12.10 (10.84-13.26)
Afghanistan	51.60 (50.34-52.94)	43.57 (40.88-45.98)	7.22 (6.34-8.09)	51.61 (50.43-52.86)	43.87 (41.39-46.10)	6.90 (6.07-7.70)	54.12 (52.84-55.38)	46.17 (43.71-48.54)	7.06 (6.22-7.86)	57.93 (56.15-59.58)	49.49 (46.70-52.07)	7.59 (6.72-8.47)
Algeria	70.99 (70.17-71.88)	60.55 (57.66-63.37)	11.57 (10.37-12.78)	73.85 (72.86-74.75)	63.15 (60.06-65.99)	12.27 (10.99-13.53)	75.62 (74.81-76.46)	64.63 (61.47-67.48)	12.79 (11.45-14.05)	77.41 (76.54-78.26)	66.07 (62.80-69.00)	13.18 (11.77-14.47)
Bahrain	69.59 (68.41-70.69)	59.65 (56.78-62.54)	8.75 (7.70-9.86)	71.41 (70.24-72.55)	60.97 (57.92-63.73)	9.23 (8.08-10.31)	73.66 (72.55-74.84)	62.84 (59.66-65.81)	9.96 (8.76-11.18)	76.77 (75.00-78.49)	65.36 (61.84-68.75)	11.32 (9.77-12.83)
Egypt	65.31 (64.53-66.13)	55.95 (53.20-58.40)	10.36 (9.24-11.34)	69.85 (69.15-70.53)	59.91 (56.96-62.62)	11.05 (9.88-12.15)	70.22 (69.51-70.88)	60.29 (57.39-63.00)	10.77 (9.59-11.92)	72.13 (70.67-73.53)	62.04 (59.13-64.85)	11.16 (9.88-12.35)
Iran	67.92 (65.72-70.26)	57.97 (54.75-61.13)	11.43 (10.13-12.75)	71.44 (70.10-72.82)	61.10 (57.93-64.20)	11.53 (10.25-12.76)	72.85 (71.03-74.61)	62.41 (59.09-65.47)	11.50 (10.22-12.77)	75.94 (74.23-77.64)	64.79 (61.31-67.91)	12.46 (11.00-13.87)
Iraq	66.14 (64.37-67.65)	55.88 (52.78-58.76)	9.59 (8.41-10.73)	65.96 (64.60-67.32)	56.06 (53.00-58.86)	9.33 (8.22-10.41)	63.77 (61.03-66.70)	54.41 (50.99-57.74)	9.37 (8.24-10.44)	67.58 (65.11-70.40)	57.43 (54.06-60.82)	9.96 (8.71-11.25)
Jordan	71.77 (70.57-72.92)	61.02 (57.89-64.00)	11.10 (9.80-12.37)	73.05 (71.34-74.87)	62.41 (59.17-65.45)	11.36 (9.88-12.75)	73.77 (71.91-75.60)	63.12 (59.86-66.33)	11.32 (9.88-12.74)	76.29 (74.11-78.53)	65.10 (61.65-68.36)	12.70 (11.09-14.30)
Kuwait	75.56 (74.05-77.20)	64.89 (61.56-67.95)	12.17 (10.73-13.45)	76.44 (75.36-77.60)	65.68 (62.50-68.55)	12.18 (10.77-13.44)	77.09 (75.85-78.31)	66.15 (62.87-69.21)	12.51 (11.14-13.90)	79.70 (77.41-82.13)	68.24 (64.58-71.92)	13.58 (11.81-15.43)
Lebanon	69.08 (67.63-70.90)	59.07 (56.02-61.93)	10.81 (9.57-12.20)	76.47 (75.22-77.47)	65.26 (62.01-68.15)	12.87 (11.48-14.14)	77.95 (76.95-78.95)	66.58 (63.29-69.41)	13.46 (12.05-14.78)	80.07 (78.87-81.12)	68.28 (64.81-71.45)	14.12 (12.57-15.64)
Libya	73.03 (72.14-74.01)	62.44 (59.44-65.28)	12.03 (10.74-13.26)	74.34 (73.29-75.31)	63.63 (60.55-66.47)	12.18 (10.80-13.45)	75.38 (74.30-76.43)	64.51 (61.23-67.41)	12.37 (10.94-13.66)	75.05 (73.80-76.44)	64.21 (60.90-67.19)	12.10 (10.70-13.39)
Morocco	67.21 (66.47-67.86)	57.25 (54.29-59.91)	10.74 (9.57-11.81)	70.90 (70.10-71.63)	60.46 (57.46-63.35)	11.42 (10.19-12.59)	72.50 (71.67-73.35)	61.85 (58.65-64.72)	11.76 (10.50-12.93)	75.00 (74.10-75.90)	63.97 (60.67-66.87)	12.35 (11.00-13.60)
Palestine	70.95 (69.60-72.34)	60.88 (57.88-63.66)	10.76 (9.50-11.90)	71.40 (70.76-71.98)	61.50 (58.63-63.97)	10.02 (9.03-10.93)	71.78 (71.39-72.15)	61.76 (58.76-64.37)	10.15 (9.11-11.06)	71.82 (71.21-72.44)	61.79 (58.90-64.38)	9.68 (8.67-10.57)
Oman	70.65 (69.56-71.69)	60.32 (57.17-63.10)	11.30 (10.04-12.47)	74.40 (73.59-75.24)	63.91 (60.80-66.72)	11.71 (10.47-12.91)	75.89 (75.07-76.75)	65.23 (62.09-68.13)	12.05 (10.75-13.28)	76.97 (76.31-77.67)	66.20 (63.04-69.10)	12.55 (11.20-13.78)
Qatar	74.96 (72.94-76.78)	63.98 (60.61-67.22)	11.96 (10.54-13.40)	75.01 (73.12-76.86)	64.10 (60.77-67.24)	11.80 (10.36-13.25)	76.78 (74.93-78.69)	65.62 (62.10-68.80)	12.38 (10.82-13.93)	79.87 (77.08-82.50)	68.15 (64.14-71.93)	13.76 (11.81-15.75)
Saudi Arabia	71.87 (70.66-73.08)	61.38 (58.20-64.28)	11.42 (10.15-12.61)	72.85 (72.06-73.56)	62.68 (59.82-65.35)	10.40 (9.34-11.42)	73.98 (73.42-74.52)	63.79 (60.89-66.49)	10.69 (9.57-11.67)	77.10 (76.41-77.82)	66.38 (63.26-69.22)	12.10 (10.90-13.25)
Sudan	58.55 (57.57-59.49)	49.74 (47.07-52.25)	9.42 (8.42-10.39)	62.57 (61.71-63.35)	53.29 (50.59-55.77)	9.75 (8.72-10.70)	65.17 (64.32-65.98)	55.55 (52.63-58.13)	10.21 (9.14-11.19)	68.30 (66.88-69.44)	58.17 (55.08-61.12)	11.07 (9.89-12.15)
Syria	70.89 (70.16-71.62)	60.75 (57.75-63.49)	11.42 (10.18-12.53)	73.24 (72.63-73.95)	63.12 (60.12-65.89)	11.50 (10.29-12.70)	75.02 (74.45-75.52)	64.62 (61.53-67.42)	12.20 (10.92-13.38)	68.05 (61.97-74.64)	58.84 (53.20-64.46)	12.21 (10.93-13.45)
Tunisia	71.62 (70.76-72.37)	61.57 (58.61-64.31)	11.54 (10.29-12.68)	74.87 (73.83-75.88)	64.37 (61.23-67.23)	12.39 (11.09-13.64)	76.17 (75.03-77.42)	65.48 (62.29-68.38)	12.80 (11.46-14.07)	77.46 (75.92-79.14)	66.61 (63.28-69.77)	13.04 (11.53-14.61)
Turkey	70.08 (69.22-70.95)	59.56 (56.53-62.35)	12.28 (10.93-13.53)	74.05 (73.35-74.76)	63.25 (60.23-66.11)	12.66 (11.31-13.91)	77.25 (76.50-78.00)	65.91 (62.72-68.94)	13.53 (12.05-14.90)	79.16 (77.60-80.78)	67.60 (64.09-70.75)	14.01 (12.36-15.53)
United Arab Emirates	72.51 (70.08-74.61)	62.07 (58.38-65.59)	11.13 (9.58-12.62)	74.33 (72.92-75.65)	63.84 (60.60-66.82)	11.60 (10.24-12.94)	76.13 (75.13-77.04)	65.37 (62.19-68.25)	12.30 (11.00-13.55)	75.51 (73.45-77.45)	65.08 (61.51-68.19)	12.11 (10.64-13.42)
Yemen	58.69 (57.48-59.95)	48.77 (45.85-51.59)	8.86 (7.71-10.02)	62.61 (61.49-63.84)	52.26 (49.21-55.10)	9.15 (8.02-10.19)	65.51 (64.39-66.70)	54.80 (51.61-57.75)	9.56 (8.40-10.64)	66.68 (65.08-68.07)	56.00 (52.70-58.87)	10.09 (8.86-11.20)
South Asia	59.10 (58.67-59.53)	50.92 (48.58-53.07)	8.91 (7.99-9.72)	62.54 (62.13-62.93)	53.82 (51.38-56.02)	9.42 (8.47-10.29)	64.82 (64.35-65.27)	55.79 (53.25-58.07)	9.85 (8.87-10.74)	68.77 (68.23-69.23)	59.17 (56.46-61.58)	10.43 (9.40-11.36)
Bangladesh	58.09 (57.29-58.96)	50.03 (47.60-52.31)	9.37 (8.37-10.30)	65.41 (64.66-66.17)	56.27 (53.53-58.75)	10.73 (9.62-11.76)	68.37 (67.74-69.03)	58.87 (56.14-61.36)	11.21 (10.12-12.23)	72.62 (71.44-73.73)	62.77 (59.96-65.40)	12.04 (10.83-13.21)
Bhutan	59.92 (58.30-61.55)	51.34 (48.69-53.89)	9.91 (8.73-11.00)	65.61 (64.17-66.94)	56.22 (53.39-58.94)	11.08 (9.89-12.22)	70.04 (68.79-71.25)	59.99 (57.03-62.79)	11.94 (10.68-13.15)	73.79 (72.25-75.26)	63.18 (60.00-66.21)	12.36 (11.05-13.62)
India	58.96 (58.49-59.44)	50.77 (48.39-52.90)	8.70 (7.80-9.50)	62.29 (61.81-62.72)	53.54 (51.11-55.75)	9.25 (8.30-10.13)	64.55 (64.00-65.05)	55.50 (52.95-57.81)	9.69 (8.73-10.59)	68.55 (67.92-69.11)	58.89 (56.14-61.33)	10.25 (9.23-11.19)
Nepal	57.70 (56.93-58.50)	49.87 (47.68-51.98)	9.10 (8.21-9.98)	63.72 (63.00-64.41)	55.10 (52.62-57.38)	9.83 (8.86-10.75)	66.47 (65.69-67.20)	57.53 (54.90-59.85)	10.22 (9.23-11.16)	70.86 (70.07-71.66)	61.37 (58.60-63.77)	10.97 (9.87-12.00)
Pakistan	62.44 (61.69-63.20)	54.05 (51.41-56.35)	10.38 (9.30-11.38)	62.61 (61.81-63.49)	54.48 (52.14-56.64)	9.72 (8.77-10.63)	64.01 (62.87-65.07)	55.56 (53.03-57.95)	9.93 (8.92-10.87)	67.61 (66.07-69.13)	58.63 (55.82-61.19)	10.57 (9.41-11.67)
Sub-Saharan Africa	53.69 (53.26-54.12)	46.59 (44.52-48.56)	9.38 (8.48-10.20)	52.84 (52.38-53.32)	45.93 (43.90-47.82)	9.21 (8.32-10.02)	55.25 (54.76-55.70)	48.10 (46.06-50.04)	9.60 (8.69-10.41)	62.87 (62.14-63.54)	54.80 (52.36-57.02)	10.53 (9.53-11.44)
Southern Sub-Saharan Africa	63.76 (63.00-64.51)	55.39 (53.06-57.65)	12.03 (10.93-13.13)	53.18 (51.96-54.38)	46.32 (44.18-48.40)	9.80 (8.86-10.76)	49.82 (48.83-50.84)	43.49 (41.60-45.27)	9.54 (8.64-10.41)	61.62 (60.70-62.56)	53.33 (50.95-55.66)	11.27 (10.18-12.28)
Botswana	64.43 (61.94-67.07)	56.28 (53.34-59.29)	10.07 (8.88-11.41)	46.56 (41.13-53.70)	40.92 (36.03-46.55)	8.46 (5.88-11.05)	49.46 (45.18-54.07)	43.26 (39.54-47.29)	8.93 (6.69-10.97)	65.23 (61.71-71.46)	56.60 (52.77-61.71)	10.70 (8.81-13.79)
Lesotho	60.42 (58.95-61.98)	52.69 (50.17-55.10)	9.20 (8.17-10.25)	48.82 (45.82-51.91)	42.60 (39.53-45.50)	7.89 (6.89-9.30)	43.24 (40.33-46.10)	37.81 (35.00-40.50)	7.26 (6.32-8.26)	50.31 (47.56-52.76)	43.82 (41.11-46.44)	8.02 (6.86-9.37)
Namibia	61.48 (60.46-62.53)	53.87 (51.53-56.05)	9.25 (8.37-10.13)	53.58 (50.52-57.08)	47.05 (43.87-50.32)	8.62 (7.29-10.44)	52.50 (49.55-55.47)	46.08 (43.00-49.08)	9.20 (7.83-10.66)	64.77 (62.09-68.16)	56.47 (53.20-59.89)	11.23 (9.67-13.29)
South Africa	64.35 (63.70-65.05)	55.76 (53.30-58.01)	12.64 (11.45-13.68)	55.40 (54.14-56.39)	48.13 (45.90-50.24)	10.32 (9.35-11.23)	51.05 (49.94-52.20)	44.46 (42.38-46.43)	10.08 (9.13-10.97)	62.44 (61.24-63.47)	53.83 (51.37-56.32)	11.87 (10.70-12.97)
Swaziland	62.36 (60.46-64.23)	54.54 (51.99-57.06)	9.26 (8.29-10.34)	47.40 (43.19-52.03)	41.36 (37.58-45.27)	8.28 (6.56-9.96)	42.93 (39.90-46.42)	37.47 (34.57-40.44)	7.46 (6.13-8.54)	57.44 (53.93-61.59)	49.62 (45.86-53.17)	9.47 (7.73-11.46)
Zimbabwe	61.61 (58.66-65.18)	54.07 (50.98-57.58)	10.72 (9.43-12.99)	46.59 (43.30-50.52)	41.03 (37.90-44.52)	8.47 (7.12-10.07)	45.65 (43.26-48.28)	40.32 (37.97-42.91)	7.96 (6.97-9.17)	59.20 (57.12-61.37)	52.08 (49.59-54.69)	9.43 (8.33-10.72)
Western Sub-Saharan Africa	54.42 (53.63-55.13)	46.92 (44.63-49.08)	9.78 (8.76-10.74)	54.76 (53.84-55.67)	47.35 (45.08-49.50)	9.83 (8.84-10.79)	57.11 (56.27-57.96)	49.48 (47.27-51.67)	10.23 (9.23-11.21)	63.31 (62.29-64.26)	55.03 (52.45-57.33)	11.01 (9.96-12.02)
Benin	55.52 (54.60-56.50)	47.49 (44.86-49.77)	9.63 (8.51-10.58)	57.60 (56.52-58.66)	49.67 (47.23-51.93)	9.72 (8.67-10.63)	59.88 (58.74-60.99)	51.80 (49.16-54.17)	9.96 (8.86-10.90)	64.42 (63.05-65.66)	55.95 (53.15-58.38)	10.26 (9.18-11.26)
Burkina Faso	50.65 (49.63-51.82)	43.14 (40.73-45.45)	8.89 (7.82-9.98)	52.22 (51.09-53.34)	44.79 (42.38-47.08)	9.14 (8.11-10.16)	55.64 (54.66-56.58)	48.05 (45.65-50.35)	9.53 (8.57-10.45)	60.79 (59.53-61.85)	52.93 (50.52-55.37)	9.91 (8.98-10.84)
Cameroon	58.45 (57.32-59.64)	50.30 (47.73-52.58)	9.76 (8.65-10.74)	54.45 (52.89-56.12)	47.10 (44.55-49.51)	9.16 (8.05-10.26)	55.27 (53.60-57.03)	47.93 (45.46-50.31)	9.38 (8.25-10.44)	60.14 (57.83-62.44)	52.49 (49.62-55.16)	10.09 (8.83-11.38)
Cape Verde	67.97 (67.08-68.96)	59.03 (56.42-61.66)	11.43 (10.39-12.47)	69.81 (67.96-71.83)	60.66 (57.72-63.62)	11.82 (10.53-13.13)	71.26 (69.78-73.04)	62.02 (59.12-64.97)	11.96 (10.74-13.24)	73.59 (72.27-75.09)	64.18 (61.25-66.90)	12.46 (11.18-13.64)
Chad	53.26 (52.23-54.27)	45.89 (43.42-48.03)	9.47 (8.43-10.36)	52.15 (50.93-53.49)	44.98 (42.62-47.20)	9.17 (8.12-10.15)	54.06 (52.61-55.52)	46.64 (44.21-49.05)	9.53 (8.44-10.52)	59.83 (58.14-61.41)	51.72 (49.18-54.35)	10.10 (9.01-11.18)
Cote d'Ivoire	54.77 (53.70-55.70)	47.09 (44.64-49.41)	8.76 (7.74-9.69)	52.42 (51.07-53.75)	45.52 (43.02-47.72)	8.56 (7.51-9.54)	53.92 (52.50-55.32)	46.98 (44.54-49.21)	8.82 (7.81-9.73)	59.87 (58.43-61.26)	52.15 (49.52-54.57)	9.46 (8.39-10.38)
The Gambia	61.58 (60.34-62.95)	53.21 (50.41-55.72)	10.15 (9.00-11.21)	63.30 (62.18-64.46)	54.70 (51.77-57.31)	10.18 (9.06-11.33)	64.48 (63.33-65.71)	55.72 (52.79-58.27)	10.40 (9.30-11.49)	67.34 (66.13-68.67)	58.27 (55.40-60.85)	10.92 (9.76-12.01)
Ghana	58.77 (57.59-60.00)	51.18 (48.72-53.56)	9.58 (8.57-10.65)	59.31 (57.96-60.74)	51.92 (49.49-54.16)	9.78 (8.71-10.84)	60.46 (59.23-61.74)	53.00 (50.54-55.26)	10.01 (8.95-11.06)	66.01 (64.71-67.52)	57.92 (55.33-60.37)	10.67 (9.62-11.74)
Guinea	51.94 (50.72-53.13)	44.85 (42.58-46.99)	9.59 (8.56-10.59)	54.66 (53.47-55.82)	47.49 (45.13-49.53)	9.43 (8.46-10.34)	56.41 (55.01-57.80)	49.14 (46.65-51.47)	9.53 (8.51-10.45)	60.68 (58.78-62.49)	52.92 (50.13-55.61)	9.77 (8.71-10.80)
Guinea-Bissau	49.16 (47.70-50.65)	42.80 (40.68-44.92)	8.13 (7.26-8.96)	52.21 (50.91-53.60)	45.55 (43.19-47.64)	8.21 (7.35-9.05)	53.75 (52.23-55.34)	46.96 (44.65-49.19)	8.36 (7.47-9.30)	58.75 (57.25-60.16)	51.39 (48.80-53.74)	8.79 (7.85-9.69)
Liberia	49.83 (48.74-50.96)	42.31 (39.81-44.50)	9.31 (8.25-10.34)	54.44 (53.34-55.64)	46.28 (43.85-48.67)	9.25 (8.16-10.32)	58.65 (57.60-59.74)	49.96 (47.34-52.45)	9.42 (8.36-10.52)	64.38 (63.37-65.31)	55.27 (52.46-57.85)	9.94 (8.88-10.86)

Appendix Table 2. GBD 2016 Life expectancy at birth and HALE at birth and at age 65 by location for GBD years 1990, 2000, 2006 & 2016, both sexes combined.

Location	1990			2000			2006			2016		
	Life Expectancy	HALE		Life Expectancy	HALE		Life Expectancy	HALE		Life Expectancy	HALE	
	At birth	At birth	At age 65	At birth	At birth	At age 65	At birth	At birth	At age 65	At birth	At birth	At age 65
Mali	49.33 (48.27-50.43)	42.47 (40.29-44.61)	8.79 (7.84-9.71)	53.26 (52.07-54.51)	45.91 (43.43-48.22)	9.57 (8.54-10.57)	57.05 (55.78-58.38)	49.25 (46.83-51.67)	10.21 (9.15-11.18)	61.84 (59.55-64.16)	53.69 (50.71-56.66)	10.78 (9.54-11.98)
Mauritania	59.91 (58.85-61.06)	51.97 (49.48-54.21)	9.34 (8.33-10.29)	63.57 (62.52-64.71)	55.19 (52.54-57.56)	10.12 (9.04-11.18)	66.27 (64.88-67.84)	57.53 (54.83-60.15)	10.83 (9.67-12.08)	70.16 (68.28-72.41)	60.99 (57.95-63.92)	11.53 (10.22-12.94)
Niger	47.14 (45.81-48.30)	41.03 (38.97-42.95)	9.64 (8.67-10.57)	52.17 (50.97-53.42)	45.44 (43.11-47.63)	10.00 (9.01-10.97)	56.19 (54.68-57.72)	49.06 (46.59-51.34)	10.19 (9.16-11.17)	61.68 (59.68-63.72)	54.02 (51.25-56.79)	10.40 (9.22-11.54)
Nigeria	54.85 (53.30-56.28)	47.24 (44.71-49.67)	10.33 (9.21-11.46)	54.89 (53.06-56.88)	47.32 (44.73-50.06)	10.45 (9.24-11.72)	57.47 (55.84-59.24)	49.62 (47.04-52.30)	11.07 (9.86-12.44)	65.02 (63.18-67.05)	56.28 (53.18-59.23)	12.31 (10.91-13.65)
Sao Tome and Principe	63.25 (62.11-64.48)	55.09 (52.64-57.61)	10.74 (9.64-11.81)	64.54 (63.51-65.54)	56.36 (53.84-58.69)	10.07 (9.08-11.05)	66.84 (65.97-67.76)	58.38 (55.84-60.63)	10.44 (9.46-11.34)	70.60 (69.05-72.13)	61.65 (58.67-64.31)	11.26 (10.03-12.43)
Senegal	57.54 (56.78-58.33)	50.10 (47.75-52.30)	9.65 (8.65-10.60)	59.66 (58.86-60.51)	52.08 (49.51-54.29)	9.67 (8.66-10.59)	62.17 (61.33-62.98)	54.26 (51.63-56.55)	9.83 (8.85-10.77)	66.24 (65.34-67.11)	57.89 (55.30-60.24)	10.31 (9.32-11.23)
Sierra Leone	50.52 (49.26-51.75)	43.68 (41.45-45.66)	9.53 (8.49-10.42)	50.89 (49.78-51.98)	43.98 (41.70-45.99)	9.09 (8.11-9.97)	52.87 (51.77-54.02)	45.86 (43.55-47.91)	8.98 (8.03-9.92)	59.15 (57.74-60.62)	51.63 (49.11-54.05)	9.60 (8.60-10.60)
Togo	57.39 (56.36-58.41)	49.78 (47.22-52.01)	9.56 (8.56-10.50)	56.33 (54.75-58.02)	48.92 (46.49-51.20)	9.44 (8.36-10.54)	56.99 (55.49-58.58)	49.66 (47.22-51.95)	9.65 (8.58-10.72)	62.59 (61.28-63.87)	54.87 (52.27-57.20)	10.07 (9.01-11.06)
Eastern Sub-Saharan Africa	51.17 (50.67-51.70)	44.83 (42.88-46.54)	8.47 (7.67-9.18)	51.47 (50.91-52.09)	45.12 (43.21-46.85)	8.65 (7.86-9.40)	55.61 (55.01-56.20)	48.79 (46.74-50.63)	9.30 (8.43-10.06)	63.39 (62.51-64.22)	55.65 (53.16-57.84)	10.20 (9.22-11.08)
Burundi	47.99 (46.11-49.80)	42.53 (40.39-44.64)	7.22 (6.47-7.95)	47.89 (46.21-49.54)	42.36 (40.17-44.44)	7.49 (6.73-8.25)	54.30 (52.75-55.81)	47.99 (45.55-50.09)	8.58 (7.71-9.42)	60.34 (57.89-62.72)	53.51 (50.62-56.28)	9.39 (8.46-10.31)
Comoros	58.60 (57.36-60.17)	51.17 (48.65-53.66)	9.29 (8.32-10.28)	61.46 (60.29-62.76)	53.73 (51.24-56.21)	9.72 (8.76-10.63)	64.53 (63.38-65.83)	56.55 (53.99-58.99)	10.42 (9.40-11.47)	67.54 (65.93-69.18)	59.29 (56.51-62.00)	10.87 (9.81-11.96)
Djibouti	61.18 (60.16-62.13)	53.73 (51.45-55.88)	10.54 (9.57-11.45)	61.30 (59.66-63.29)	53.84 (51.25-56.29)	10.50 (9.38-11.65)	61.85 (59.55-64.01)	54.39 (51.45-57.04)	10.54 (9.26-11.74)	66.68 (64.66-68.95)	58.58 (55.64-61.49)	11.13 (9.81-12.66)
Eritrea	52.30 (51.12-53.56)	46.01 (43.93-48.08)	7.85 (7.06-8.61)	58.08 (56.78-59.44)	51.17 (48.84-53.41)	9.04 (8.16-9.96)	60.71 (59.19-62.21)	53.51 (51.01-55.93)	9.29 (8.35-10.25)	63.66 (62.13-65.27)	56.16 (53.60-58.64)	9.61 (8.67-10.59)
Ethiopia	46.57 (45.65-47.58)	41.10 (39.28-42.73)	7.30 (6.59-7.98)	51.10 (50.06-52.22)	45.13 (43.24-47.00)	8.21 (7.42-8.95)	57.02 (55.75-58.28)	50.33 (48.20-52.43)	9.04 (8.15-9.88)	65.63 (63.88-67.48)	57.91 (55.18-60.49)	10.14 (9.06-11.24)
Kenya	61.43 (60.76-62.06)	54.09 (51.84-56.08)	10.37 (9.44-11.22)	55.20 (54.51-55.89)	48.55 (46.54-50.37)	9.90 (8.98-10.68)	58.47 (57.85-59.06)	51.50 (49.31-53.37)	10.40 (9.45-11.24)	66.84 (66.12-67.57)	58.59 (55.98-60.73)	11.27 (10.25-12.19)
Madagascar	55.59 (54.47-56.70)	48.63 (46.42-50.76)	9.07 (8.21-9.87)	58.45 (57.22-59.64)	51.22 (48.89-53.48)	9.32 (8.44-10.16)	60.40 (58.97-61.89)	52.98 (50.25-55.41)	9.63 (8.65-10.60)	62.68 (60.36-65.21)	55.21 (52.34-58.40)	10.01 (8.90-11.28)
Malawi	48.68 (46.23-51.02)	42.45 (39.78-45.24)	9.42 (8.19-10.89)	44.72 (42.13-47.82)	39.11 (36.41-42.16)	8.44 (7.13-10.29)	48.45 (46.15-51.25)	42.41 (39.89-45.34)	9.11 (7.72-10.77)	60.18 (57.98-62.57)	52.71 (50.03-55.57)	10.22 (9.03-11.68)
Mozambique	50.73 (49.78-51.73)	44.00 (41.77-45.95)	9.18 (8.26-10.09)	51.36 (49.17-53.63)	44.56 (41.81-47.16)	9.39 (8.10-10.75)	52.63 (50.10-55.21)	45.66 (42.69-48.44)	9.86 (8.40-11.19)	60.02 (57.87-61.89)	52.08 (49.32-54.87)	10.38 (9.19-11.64)
Rwanda	49.12 (47.65-50.61)	43.40 (41.25-45.48)	7.86 (7.08-8.68)	49.44 (47.94-51.02)	43.33 (41.22-45.29)	8.22 (7.38-9.03)	59.60 (58.31-61.04)	52.23 (49.82-54.50)	10.30 (9.23-11.31)	67.77 (66.09-69.39)	59.47 (56.70-62.13)	11.18 (9.98-12.33)
Somalia	50.59 (48.67-52.33)	44.53 (42.12-46.83)	8.16 (7.26-9.02)	51.99 (50.22-53.82)	45.75 (43.48-48.04)	8.06 (7.16-8.98)	53.98 (52.05-55.84)	47.59 (45.16-50.09)	8.25 (7.35-9.25)	57.13 (54.93-59.19)	50.39 (47.63-52.86)	8.60 (7.58-9.51)
South Sudan	51.53 (49.34-53.92)	43.56 (40.53-46.52)	8.83 (7.55-10.04)	54.50 (52.10-57.16)	46.36 (43.20-49.39)	8.78 (7.52-10.27)	56.76 (53.99-59.70)	48.63 (45.43-51.93)	9.17 (7.89-10.67)	59.70 (56.96-62.59)	51.67 (48.40-54.87)	9.47 (8.27-10.82)
Tanzania	54.85 (53.58-56.09)	47.88 (45.60-50.07)	9.46 (8.49-10.44)	52.85 (51.20-55.03)	46.23 (43.85-48.53)	9.27 (8.21-10.54)	56.00 (54.49-57.89)	49.10 (46.75-51.44)	9.83 (8.73-11.04)	64.29 (62.80-65.90)	56.53 (53.88-59.11)	10.60 (9.51-11.75)
Uganda	49.10 (46.95-50.96)	42.44 (39.78-44.93)	8.19 (7.31-9.08)	48.66 (47.18-50.27)	42.22 (40.02-44.39)	8.31 (7.43-9.19)	53.69 (52.34-54.93)	46.70 (44.42-48.85)	8.89 (7.98-9.75)	62.20 (60.77-63.84)	54.35 (51.80-56.76)	9.99 (8.93-11.10)
Zambia	52.12 (49.96-54.12)	45.79 (43.38-48.23)	9.61 (8.57-10.74)	44.15 (41.69-46.73)	38.87 (36.48-41.39)	7.35 (6.46-8.34)	46.71 (44.53-48.88)	41.21 (38.88-43.55)	7.54 (6.69-8.59)	58.62 (56.01-61.39)	51.46 (48.77-54.47)	9.13 (7.99-10.51)
Central Sub-Saharan Africa	52.61 (51.58-53.68)	45.08 (42.74-47.23)	8.32 (7.45-9.15)	52.77 (51.61-53.87)	45.32 (42.94-47.51)	8.33 (7.44-9.17)	55.55 (54.43-56.67)	47.84 (45.34-50.04)	8.66 (7.76-9.49)	61.70 (60.08-63.24)	53.38 (50.56-55.95)	9.45 (8.49-10.32)
Angola	50.19 (47.57-53.04)	43.68 (40.91-46.74)	8.12 (7.03-9.35)	53.32 (50.58-56.48)	46.45 (43.44-49.69)	8.39 (7.15-9.60)	56.89 (54.13-59.84)	49.63 (46.69-52.54)	8.93 (7.72-10.23)	64.64 (61.04-67.84)	56.29 (52.85-59.67)	10.03 (8.66-11.43)
Central African Republic	48.09 (46.20-49.88)	41.42 (38.81-43.72)	7.17 (6.38-7.94)	44.37 (41.75-47.00)	38.38 (35.55-41.18)	6.91 (6.02-7.92)	45.25 (42.79-47.89)	39.26 (36.42-42.02)	7.08 (6.11-8.14)	50.16 (47.19-53.35)	43.65 (40.42-46.76)	7.48 (6.53-8.55)
Congo (Brazzaville)	54.03 (52.46-55.65)	46.82 (44.19-49.20)	7.41 (6.53-8.20)	52.75 (51.28-54.26)	45.72 (43.41-48.04)	7.82 (6.90-8.72)	57.34 (55.86-58.87)	49.68 (47.10-52.28)	8.76 (7.79-9.67)	62.89 (60.52-65.47)	54.63 (51.46-57.92)	9.67 (8.53-10.96)
Democratic Republic of the Congo	53.82 (52.29-55.27)	45.81 (43.28-48.24)	8.65 (7.64-9.56)	53.35 (51.78-54.83)	45.55 (42.78-48.08)	8.52 (7.58-9.37)	55.87 (54.45-57.11)	47.87 (45.24-50.30)	8.72 (7.77-9.61)	61.56 (59.63-63.33)	53.11 (50.12-55.81)	9.45 (8.45-10.31)
Equatorial Guinea	48.09 (45.38-51.18)	41.80 (38.76-44.71)	7.39 (6.39-8.42)	54.31 (50.83-58.16)	47.12 (43.76-50.84)	9.02 (7.65-10.70)	58.96 (54.79-63.68)	51.08 (47.08-55.56)	10.40 (8.53-12.53)	65.47 (61.15-70.04)	56.66 (52.48-60.94)	11.50 (9.50-13.67)
Gabon	59.39 (58.13-60.67)	51.52 (49.10-53.80)	8.64 (7.75-9.52)	59.11 (57.61-60.70)	51.32 (48.80-53.81)	8.80 (7.84-9.79)	60.44 (58.70-62.06)	52.46 (49.90-54.93)	9.27 (8.28-10.20)	66.53 (64.38-68.73)	57.58 (54.45-60.54)	10.54 (9.29-11.78)

Appendix Table 3. GBD 2016 DALY counts for all causes and level 1 cause groups by location for 1990, 2000, 2006 & 2016, both sexes combined.

	Cause	1990	2000	2006	2016
Global	All causes	244,843,050,675,000<br>(230,521,824,022,000–260,833,953,140,000)	251,519,925,984,000<br>(234,507,299,842,000–269,886,235,928,000)	249,069,886,884,000<br>(230,852,711,979,000–268,986,112,971,000)	239,125,803,263,000<br>(218,425,413,363,000–263,169,901,686,000)
Global	Group I	111,417,656,953,000<br>(107,394,882,631,000–115,605,018,432,000)	100,921,524,965,000<br>(97,129,628,182,000–105,047,078,124,000)	91,880,479,818,800<br>(88,524,212,748,200–95,945,232,235,100)	66,782,372,793,600<br>(63,221,237,922,100–70,840,514,487,900)
Global	NCD	107,453,948,360,000<br>(95,763,476,055,700–119,700,832,754,000)	123,939,923,889,000<br>(110,362,612,481,000–138,278,233,841,000)	131,210,199,438,000<br>(116,306,376,838,000–147,249,442,210,000)	146,799,996,380,000<br>(129,653,544,270,000–165,853,771,564,000)
Global	Injuries	25,971,445,361,900<br>(24,264,713,787,600–27,681,790,013,600)	26,658,477,129,400<br>(24,922,317,357,400–28,588,685,743,100)	25,979,207,627,000<br>(24,223,462,094,800–28,097,675,164,000)	25,543,434,088,900<br>(23,608,955,431,100–28,068,907,654,400)
High SDI	All causes	25,734,884,512,200<br>(23,061,492,666,000–28,711,649,444,700)	25,944,281,058,600<br>(23,034,061,737,800–29,269,353,054,900)	26,306,701,129,900<br>(23,213,594,883,100–29,797,300,699,400)	27,928,285,612,700<br>(24,497,215,072,900–31,750,833,377,100)
High SDI	Group I	1,622,970,031,360<br>(1,493,984,316,970–1,775,655,186,060)	1,349,725,834,770<br>(1,229,857,652,940–1,498,413,261,920)	1,282,723,556,860<br>(1,162,279,376,970–1,432,277,159,580)	1,235,162,040,630<br>(1,111,080,941,270–1,401,092,946,530)
High SDI	NCD	21,142,312,526,900<br>(18,788,991,598,000–23,731,548,552,100)	21,820,513,331,100<br>(19,226,985,749,500–24,752,415,910,300)	22,321,095,640,000<br>(19,547,766,621,500–25,409,207,139,000)	24,025,171,733,000<br>(20,961,183,338,000–27,420,219,702,200)
High SDI	Injuries	2,969,601,953,960<br>(2,674,548,214,420–3,331,438,908,640)	2,774,041,892,710<br>(2,468,511,746,680–3,166,551,472,770)	2,702,881,933,040<br>(2,382,448,306,610–3,115,530,469,150)	2,667,951,839,020<br>(2,293,663,805,420–3,131,705,925,380)
High-middle SDI	All causes	29,341,168,681,900<br>(26,827,216,355,300–32,178,323,491,900)	31,348,822,272,400<br>(28,580,755,951,300–34,422,664,223,600)	31,170,726,404,400<br>(28,148,942,113,200–34,681,636,144,300)	31,018,424,051,300<br>(27,184,236,630,400–35,061,937,044,900)
High-middle SDI	Group I	5,212,421,411,820<br>(4,848,626,874,880–5,633,490,565,850)	3,816,289,413,660<br>(3,539,498,655,100–4,145,910,134,430)	3,256,664,198,280<br>(2,967,713,901,180–3,619,537,888,690)	2,649,461,609,740<br>(2,338,399,877,550–3,002,400,229,920)
High-middle SDI	NCD	19,638,582,152,100<br>(17,564,185,818,800–21,878,145,747,200)	22,855,951,225,800<br>(20,519,706,345,300–25,332,947,039,700)	23,557,778,287,900<br>(21,006,708,074,200–26,411,455,896,800)	24,613,895,865,200<br>(21,476,361,666,400–27,955,705,662,700)
High-middle SDI	Injuries	4,490,165,117,960<br>(4,109,822,517,570–4,900,509,794,990)	4,676,581,632,950<br>(4,308,693,461,880–5,097,907,055,730)	4,356,283,918,260<br>(3,982,554,522,870–4,783,462,268,900)	3,755,066,576,360<br>(3,266,392,705,690–4,312,446,086,570)
Middle SDI	All causes	67,482,434,087,600<br>(63,201,553,325,200–72,366,502,286,500)	66,061,560,572,800<br>(61,125,919,945,300–71,643,334,282,600)	66,009,615,427,200<br>(60,656,692,034,900–72,109,116,729,500)	65,656,933,311,500<br>(59,642,059,462,600–72,497,230,217,800)
Middle SDI	Group I	23,278,069,353,500<br>(22,292,101,461,700–24,440,372,931,600)	16,455,517,510,200<br>(15,695,126,218,600–17,431,047,443,200)	14,740,408,714,600<br>(14,002,739,486,600–15,618,666,341,700)	10,202,384,835,300<br>(9,459,328,533,060–11,058,811,327,300)
Middle SDI	NCD	34,945,379,341,000<br>(31,343,638,865,100–38,676,024,547,700)	40,611,237,848,000<br>(36,426,145,612,100–45,191,292,735,300)	42,778,163,925,800<br>(38,290,236,371,100–47,780,987,045,200)	47,697,619,454,000<br>(42,720,377,219,800–53,454,174,809,100)
Middle SDI	Injuries	9,258,985,393,080<br>(8,799,073,222,270–9,693,869,703,470)	8,994,805,214,530<br>(8,530,947,518,200–9,476,020,909,620)	8,491,042,786,740<br>(8,000,523,094,700–9,011,086,714,020)	7,756,929,022,250<br>(7,205,526,352,950–8,404,741,203,100)
Low-middle SDI	All causes	86,383,898,222,000<br>(82,512,671,834,200–90,862,451,382,100)	87,380,398,458,500<br>(82,677,647,742,700–92,523,078,460,100)	85,117,565,457,700<br>(79,888,162,374,100–90,971,991,570,000)	76,925,631,883,200<br>(70,816,533,192,200–83,635,957,451,000)
Low-middle SDI	Group I	54,715,692,821,300<br>(52,706,025,103,000–56,804,677,527,700)	49,767,740,047,400<br>(47,673,718,005,100–51,843,700,539,400)	44,400,399,072,200<br>(42,402,806,237,500–46,445,421,152,900)	30,372,859,830,200<br>(28,441,688,944,400–32,458,077,630,000)
Low-middle SDI	NCD	24,853,616,284,800<br>(21,992,476,952,300–27,825,650,085,600)	30,144,427,928,300<br>(26,709,117,666,100–33,872,753,023,200)	32,881,131,574,400<br>(29,026,209,500,200–36,946,136,120,500)	38,361,114,678,600<br>(33,911,910,159,900–43,240,329,348,600)
Low-middle SDI	Injuries	6,814,589,115,930<br>(6,125,261,329,540–7,412,967,224,870)	7,468,230,482,770<br>(6,803,175,473,490–8,052,976,711,180)	7,836,034,811,110<br>(7,185,452,719,590–8,445,587,342,870)	8,191,657,374,420<br>(7,490,164,571,860–8,983,296,071,210)
Low SDI	All causes	35,847,917,711,400<br>(34,247,608,716,200–37,434,168,162,100)	40,772,048,114,400<br>(38,851,718,331,600–42,650,132,860,700)	40,462,788,331,200<br>(38,598,851,835,400–42,508,508,165,400)	37,628,921,958,700<br>(35,207,448,951,100–40,339,236,818,100)
Low SDI	Group I	26,553,843,165,200<br>(25,279,472,745,000–27,906,181,688,900)	29,524,858,313,000<br>(28,114,958,705,100–30,924,866,662,000)	28,200,551,930,300<br>(26,961,219,740,900–29,505,227,952,700)	22,357,268,792,800<br>(21,035,942,226,900–23,793,200,850,900)
Low SDI	NCD	6,845,877,040,280<br>(5,992,657,787,070–7,729,764,811,940)	8,488,271,398,530<br>(7,480,709,905,020–9,566,913,158,180)	9,651,832,606,430<br>(8,546,557,032,980–10,876,246,325,500)	12,079,062,719,300<br>(10,629,835,609,500–13,613,526,639,500)
Low SDI	Injuries	2,448,197,505,960<br>(2,098,355,462,130–2,760,099,009,010)	2,758,918,402,850<br>(2,475,400,594,820–3,035,866,540,190)	2,610,403,794,480<br>(2,390,274,471,770–2,840,639,057,780)	3,192,590,446,600<br>(2,955,896,880,430–3,475,217,780,870)
High-income	All causes	25,695,703,604,200<br>(23,004,208,930,100–28,735,583,703,300)	26,113,978,312,500<br>(23,175,116,242,800–29,505,216,300,100)	26,537,673,006,500<br>(23,358,860,957,300–30,097,773,593,300)	28,356,322,011,300<br>(24,828,931,253,300–32,293,239,654,000)
High-income	Group I	1,713,913,861,080<br>(1,590,064,280,240–1,862,493,193,360)	1,461,884,414,860<br>(1,340,784,446,280–1,612,281,003,450)	1,388,451,022,700<br>(1,264,244,499,770–1,543,174,789,380)	1,340,761,900,820<br>(1,210,759,177,210–1,507,812,558,840)
High-income	NCD	21,105,279,285,000<br>(18,700,064,512,700–23,756,417,927,200)	21,941,121,559,800<br>(19,302,037,272,000–24,923,842,238,500)	22,497,824,311,800<br>(19,663,782,568,100–25,662,632,531,900)	24,371,097,348,600<br>(21,182,262,522,500–27,906,783,964,400)
High-income	Injuries	2,876,510,458,120<br>(2,593,380,196,970–3,223,103,200,840)	2,710,972,337,880<br>(2,411,261,211,750–3,092,152,390,330)	2,651,397,672,040<br>(2,334,124,239,130–3,050,419,452,780)	2,644,462,761,880<br>(2,279,010,350,880–3,097,857,449,050)
High-income North America	All causes	8,209,953,962,990<br>(7,339,195,287,860–9,165,228,114,870)	8,732,865,305,600<br>(7,753,574,222,470–9,858,604,334,840)	9,127,951,511,760<br>(8,060,555,214,100–10,330,904,173,400)	10,143,317,960,100<br>(8,955,145,142,090–11,500,795,111,100)
High-income North America	Group I	598,944,923,398<br>(566,194,556,276–638,893,123,622)	504,535,408,165<br>(470,350,405,890–546,723,177,812)	498,677,721,999<br>(461,522,362,871–544,711,094,367)	485,101,141,127<br>(443,078,321,809–537,226,581,337)
High-income North America	NCD	6,625,276,790,580<br>(5,843,094,894,770–7,479,970,270,030)	7,273,404,770,600<br>(6,394,832,142,060–8,261,341,504,330)	7,641,767,108,730<br>(6,671,372,865,740–8,717,078,092,440)	8,621,877,331,680<br>(7,533,583,799,260–9,843,154,196,050)
High-income North America	Injuries	985,732,249,016<br>(900,477,378,931–1,094,515,326,590)	954,925,126,827<br>(855,191,020,395–1,081,399,356,380)	987,506,681,023<br>(885,322,372,936–1,118,684,096,590)	1,036,339,487,310<br>(912,324,702,967–1,190,698,464,150)
Canada	All causes	691,303,211,393<br>(611,425,690,875–780,336,742,224)	739,318,917,570<br>(646,520,852,994–842,548,213,673)	777,122,439,908<br>(678,526,085,969–887,022,704,581)	882,428,757,019<br>(763,809,623,808–1,012,126,723,910)
Canada	Group I	36,526,068,740<br>(33,502,338,670–40,164,041,866)	32,471,947,247<br>(29,292,198,726–36,466,754,960)	33,588,843,039<br>(30,237,797,003–37,679,117,942)	37,157,404,248<br>(32,828,405,707–42,124,738,664)
Canada	NCD	573,230,840,802<br>(501,693,588,298–652,849,314,753)	631,076,752,652<br>(548,486,542,378–721,023,563,297)	665,448,467,906<br>(576,716,530,131–763,385,549,262)	761,489,723,910<br>(656,456,103,709–876,472,243,373)
Canada	Injuries	81,546,301,852<br>(73,241,835,145–92,694,473,071)	75,770,217,671<br>(66,565,918,813–87,489,728,386)	78,085,128,962<br>(67,484,358,148–91,717,835,672)	83,781,628,862<br>(71,407,478,559–100,425,036,643)
Greenland	All causes	2,352,555,946<br>(2,178,385,642–2,559,211,866)	1,922,224,442<br>(1,749,805,473–2,109,077,738)	1,849,909,933<br>(1,681,720,247–2,029,931,918)	1,725,909,776<br>(1,474,859,902–1,991,840,170)
Greenland	Group I	303,120,379<br>(270,449,656–339,820,339)	161,059,188<br>(145,188,535–178,163,163)	140,073,971<br>(123,590,413–154,955,530)	112,928,979<br>(95,112,635–130,011,203)
Greenland	NCD	1,275,903,991<br>(1,140,578,944–1,425,248,503)	1,234,382,803<br>(1,096,286,145–1,383,684,557)	1,237,759,788<br>(1,097,179,981–1,383,099,185)	1,239,422,737<br>(1,042,991,027–1,436,990,447)
Greenland	Injuries	773,531,576<br>(697,230,546–847,158,277)	526,782,451<br>(482,953,101–572,479,630)	472,076,175<br>(433,956,329–514,546,138)	373,558,060<br>(307,273,355–452,231,773)
United States	All causes	7,514,913,691,110<br>(6,721,059,808,500–8,380,598,432,870)	7,990,136,229,480<br>(7,092,795,926,710–9,004,540,733,650)	8,347,417,764,070<br>(7,377,268,410,000–9,441,001,213,800)	9,257,535,753,190<br>(8,172,411,846,610–10,473,613,849,400)
United States	Group I	562,061,161,397<br>(531,512,679,790–598,332,838,934)	471,850,696,671<br>(441,270,991,213–509,967,107,965)	464,897,346,085<br>(431,289,616,181–506,686,193,076)	447,780,571,383<br>(409,434,007,759–495,416,234,801)
United States	NCD	6,049,540,678,690<br>(5,338,197,277,730–6,823,461,206,500)	6,639,766,481,750<br>(5,842,630,867,420–7,532,765,922,680)	6,973,681,066,890<br>(6,092,406,870,640–7,949,120,865,700)	7,857,693,259,810<br>(6,876,181,496,270–8,955,204,591,960)
United States	Injuries	903,311,851,027<br>(826,436,539,382–1,002,311,530,550)	878,519,051,055<br>(788,766,802,540–995,176,962,568)	908,839,351,087<br>(816,790,848,412–1,027,695,630,410)	952,061,922,002<br>(839,685,452,884–1,091,385,885,020)
Australasia	All causes	529,736,524,430<br>(466,929,605,039–598,389,412,233)	546,334,542,588<br>(477,306,935,511–624,117,875,532)	565,716,977,257<br>(488,184,341,503–654,041,622,153)	649,094,994,585<br>(560,653,390,493–748,783,159,792)
Australasia	Group I	25,274,963,171<br>(23,049,774,735–28,049,306,152)	22,531,973,435<br>(20,113,310,614–25,402,585,024)	22,804,156,343<br>(20,331,228,276–25,864,611,855)	23,048,400,424<br>(19,930,192,588–26,800,919,015)
Australasia	NCD	445,113,377,849<br>(389,002,726,051–505,433,027,102)	469,314,306,658<br>(405,099,685,074–539,689,968,387)	489,656,008,893<br>(419,133,355,199–568,312,207,029)	568,412,112,525<br>(486,766,826,486–659,814,061,913)
Australasia	Injuries	59,348,183,410<br>(53,766,694,247–66,240,126,812)	54,488,262,495<br>(48,297,970,875–62,218,812,116)	53,256,812,021<br>(46,635,509,615–62,224,967,388)	57,634,481,636<br>(49,174,993,580–68,319,152,724)
Australia	All causes	436,745,011,024<br>(384,984,552,002–493,498,888,187)	451,904,491,487<br>(393,775,315,056–517,600,808,911)	467,705,594,738<br>(403,206,678,638–541,162,525,466)	542,405,359,358<br>(468,000,807,480–626,174,334,019)
Australia	Group I	20,608,834,498<br>(18,695,960,226–22,944,003,036)	18,648,957,541<br>(16,679,014,137–21,039,209,893)	19,028,713,637<br>(16,962,651,078–21,602,279,449)	19,280,317,716<br>(16,618,229,162–22,456,870,857)
Australia	NCD	368,361,704,696<br>(321,943,200,532–418,710,170,510)	388,621,153,655<br>(334,996,828,083–446,853,009,629)	405,262,154,896<br>(346,274,951,738–470,902,412,711)	475,319,940,353<br>(405,419,099,552–552,719,026,415)
Australia	Injuries	47,774,471,831<br>(43,065,666,096–53,561,388,818)	44,634,380,291<br>(39,454,902,030–51,025,003,767)	43,414,726,205<br>(37,848,464,646–50,880,152,092)	47,805,101,288<br>(40,624,574,697–56,848,967,784)
New Zealand	All causes	92,991,513,405<br>(83,136,936,130–104,288,506,309)	94,430,051,101<br>(82,551,634,659–107,356,290,751)	98,011,382,519<br>(85,278,169,515–112,737,856,493)	106,689,635,227<br>(91,869,094,586–123,125,700,502)
New Zealand	Group I	4,666,128,673<br>(4,269,445,665–5,147,144,579)	3,883,015,894<br>(3,441,262,458–4,424,834,410)	3,775,442,706<br>(3,310,183,807–4,354,871,232)	3,768,082,707<br>(3,247,357,669–4,425,547,211)
New Zealand	NCD	76,751,673,153<br>(67,527,602,990–86,941,212,774)	80,693,153,003<br>(70,042,116,781–92,250,127,763)	84,393,853,997<br>(72,836,133,020–97,472,176,362)	93,092,172,172<br>(79,636,934,752–107,862,650,607)
New Zealand	Injuries	11,573,711,580<br>(10,625,525,346–12,796,695,698)	9,853,882,204<br>(8,780,475,784–11,201,375,084)	9,842,085,816<br>(8,699,563,394–11,393,959,376)	9,829,380,348<br>(8,395,862,018–11,700,626,527)
High-income Asia Pacific	All causes	4,045,086,962,920<br>(3,568,861,855,260–4,558,524,907,360)	4,260,024,508,100<br>(3,737,575,421,030–4,842,376,288,990)	4,372,103,817,170<br>(3,795,538,797,690–4,998,557,863,330)	4,627,238,177,350<br>(4,000,725,868,560–5,325,399,891,160)
High-income Asia Pacific	Group I	279,541,079,712<br>(251,483,786,487–313,151,037,037)	250,289,177,554<br>(224,918,402,676–282,919,762,522)	228,341,679,382<br>(203,772,477,936–260,769,614,400)	222,263,880,333<br>(197,135,419,288–254,417,021,345)
High-income Asia Pacific	NCD	3,246,466,323,090<br>(2,836,430,452,020–3,695,815,972,120)	3,493,817,632,630<br>(3,036,224,636,330–3,996,522,150,130)	3,647,527,103,230<br>(3,146,576,189,580–4,178,660,668,090)	3,930,816,712,160<br>(3,382,677,225,560–4,518,588,189,810)
High-income Asia Pacific	Injuries	519,079,560,118<br>(463,364,428,088–586,211,381,162)	515,917,697,914<br>(456,082,186,136–592,415,228,314)	496,235,034,562<br>(433,749,103,419–575,501,200,097)	474,157,584,858<br>(399,923,615,387–568,818,092,465)
Brunei	All causes	5,243,798,240<br>(4,614,150,655–5,921,987,437)	6,076,700,587<br>(5,276,737,420–6,905,724,087)	6,814,933,282<br>(5,904,502,053–7,761,650,423)	8,838,361,474<br>(7,467,685,331–10,270,735,686)
Brunei	Group I	603,109,446<br>(542,168,555–673,699,161)	627,437,325<br>(560,401,411–708,934,503)	616,399,540<br>(548,439,464–701,797,246)	693,083,417<br>(599,544,845–799,892,242)
Brunei	NCD	3,761,266,041<br>(3,250,841,439–4,315,477,426)	4,526,364,691<br>(3,862,477,705–5,215,134,335)	5,263,246,507<br>(4,507,977,400–6,036,220,568)	7,063,176,226<br>(5,951,981,106–8,271,699,459)
Brunei	Injuries	879,422,752<br>(760,929,710–1,004,939,551)	922,898,571<br>(804,866,352–1,060,133,774)	935,287,235<br>(803,307,452–1,085,391,813)	1,082,101,831<br>(863,013,523–1,337,697,318)
Japan	All causes	2,875,761,879,910<br>(2,531,542,013,220–3,266,605,898,450)	3,118,799,706,770<br>(2,736,124,824,360–3,547,545,038,440)	3,232,667,100,640<br>(2,832,607,538,310–3,693,816,176,950)	3,350,749,973,390<br>(2,918,088,791,980–3,842,086,441,270)
Japan	Group I	169,807,599,597<br>(154,751,229,828–189,371,400,947)	171,707,727,182<br>(156,085,846,292–191,382,099,964)	162,616,051,763<br>(146,878,778,544–182,441,857,175)	159,075,860,901<br>(142,745,956,911–180,353,181,659)
Japan	NCD	2,388,611,170,520<br>(2,080,948,029,610–2,729,662,925,930)	2,605,434,216,380<br>(2,267,174,026,060–2,979,109,455,150)	2,733,359,801,960<br>(2,373,184,922,020–3,130,719,030,190)	2,880,175,664,810<br>(2,484,746,741,780–3,305,116,674,070)
Japan	Injuries	317,343,109,801<br>(279,103,310,219–364,654,195,449)	341,657,763,200<br>(299,183,339,752–397,412,843,945)	336,691,246,919<br>(292,199,297,572–395,162,684,383)	311,498,447,677<br>(263,582,085,732–374,172,404,450)
Singapore	All causes	59,258,880,570<br>(51,606,411,546–67,538,879,860)	62,852,410,036<br>(53,462,573,668–72,749,623,268)	68,042,058,196<br>(58,056,128,518–78,837,752,182)	75,030,439,116<br>(62,138,143,912–88,646,723,001)
Singapore	Group I	5,780,520,406<br>(5,175,771,504–6,510,357,872)	5,219,484,273<br>(4,594,632,399–5,990,297,703)	5,468,721,550<br>(4,780,014,914–6,288,284,401)	5,593,245,464<br>(4,742,349,236–6,584,127,764)
Singapore	NCD	46,900,055,877<br>(40,638,656,565–53,888,686,159)	51,681,594,992<br>(43,569,466,244–59,776,888,766)	56,428,844,327<br>(47,806,167,315–65,434,643,758)	63,001,217,066<br>(52,120,042,086–74,779,924,531)

Appendix Table 3. GBD 2016 DALY counts for all causes and level 1 cause groups by location for 1990, 2000, 2006 & 2016, both sexes combined.

	Cause	1990	2000	2006	2016
Singapore	Injuries	6,578,304,287 (5,679,100,287–7,664,584,716)	5,951,330,771 (4,979,012,491–7,195,295,612)	6,144,492,319 (4,999,972,889–7,625,884,330)	6,435,976,586 (4,993,090,223–8,213,530,127)
South Korea	All causes	1,104,822,404,200 (978,483,153,131–1,248,929,352,780)	1,072,295,690,710 (936,433,651,101–1,220,838,823,890)	1,064,579,725,050 (914,771,198,978–1,223,284,173,980)	1,192,619,403,370 (970,200,080,204–1,438,974,837,160)
South Korea	Group I	103,349,850,263 (88,741,235,301–120,186,785,373)	72,734,528,774 (62,960,459,906–84,878,451,772)	59,640,506,529 (49,830,247,024–71,326,023,770)	56,901,690,551 (45,706,448,020–69,984,246,795)
South Korea	NCD	807,193,830,656 (704,872,461,834–916,904,970,429)	832,175,456,567 (721,162,404,353–950,307,641,760)	852,475,210,434 (724,545,704,391–980,700,973,541)	980,576,654,053 (800,428,553,215–1,179,384,183,880)
South Korea	Injuries	194,278,723,278 (172,305,601,062–219,870,239,880)	167,385,705,372 (147,864,000,540–189,536,152,877)	152,464,008,090 (131,625,071,491–173,769,085,048)	155,141,058,764 (120,090,100,414–195,648,872,476)
Western Europe	All causes	11,449,306,056,100 (10,254,377,209,400–12,797,289,276,700)	11,081,010,706,400 (9,828,249,020,720–12,522,759,803,700)	10,944,630,316,300 (9,619,074,527,250–12,438,717,593,700)	11,283,672,419,900 (9,836,611,016,390–12,900,746,554,000)
Western Europe	Group I	581,040,684,738 (528,315,863,075–644,225,645,088)	502,318,564,653 (450,146,101,101–566,859,466,722)	472,218,596,117 (420,205,663,184–538,835,659,013)	452,689,105,098 (399,144,056,129–522,922,767,056)
Western Europe	NCD	9,718,065,869,420 (8,650,955,471,940–10,904,488,105,500)	9,562,300,081,480 (8,440,134,228,340–10,829,343,781,600)	9,524,156,165,840 (8,347,237,352,050–10,836,357,123,400)	9,932,181,863,410 (8,637,889,985,100–11,344,818,571,700)
Western Europe	Injuries	1,150,199,501,960 (1,025,973,841,210–1,306,867,170,530)	1,016,392,060,270 (887,285,813,192–1,177,994,697,140)	948,255,554,337 (810,993,456,534–1,120,144,877,690)	898,801,451,369 (745,240,019,218–1,086,921,563,010)
Andorra	All causes	1,284,364,412 (1,070,244,762–1,495,301,669)	1,492,363,180 (1,250,253,626–1,738,627,512)	1,780,326,752 (1,503,640,714–2,091,664,112)	2,049,738,327 (1,728,584,683–2,430,254,296)
Andorra	Group I	65,772,940 (48,632,985–95,600,048)	70,231,480 (50,376,465–95,829,906)	75,050,045 (55,073,925–97,082,854)	82,534,305 (62,331,830–104,152,125)
Andorra	NCD	1,108,434,779 (919,225,404–1,292,290,146)	1,301,981,361 (1,089,587,231–1,522,332,797)	1,563,938,975 (1,319,884,603–1,839,791,825)	1,809,962,223 (1,528,827,403–2,151,172,194)
Andorra	Injuries	110,156,693 (86,121,104–138,218,522)	120,150,338 (95,049,256–150,857,186)	141,337,732 (112,215,831–176,501,197)	157,241,799 (123,799,770–198,131,959)
Austria	All causes	238,765,749,089 (214,262,880,405–265,650,731,791)	222,661,790,653 (197,026,648,229–251,186,459,736)	218,799,838,417 (192,476,664,408–249,092,038,204)	227,651,499,026 (197,620,619,750–261,169,235,328)
Austria	Group I	9,584,679,305 (8,554,150,951–10,831,094,557)	7,345,103,438 (6,224,374,966–8,605,371,036)	6,912,266,229 (5,819,109,920–8,213,538,190)	6,353,400,774 (5,272,730,745–7,779,109,133)
Austria	NCD	200,657,596,751 (179,595,950,158–223,633,106,332)	191,828,550,905 (169,595,506,429–216,627,451,941)	190,201,866,737 (166,497,223,984–217,012,557,070)	200,766,901,119 (173,817,588,456–230,187,459,857)
Austria	Injuries	28,523,473,032 (25,465,710,530–32,226,070,888)	23,488,136,311 (20,390,568,895–27,296,917,100)	21,685,705,450 (18,533,318,216–25,611,477,253)	20,531,197,132 (17,005,114,376–24,749,723,038)
Belgium	All causes	307,320,953,711 (275,845,381,077–343,676,069,119)	302,533,203,720 (267,813,828,092–340,434,043,496)	297,045,246,118 (261,850,528,659–336,579,157,834)	307,281,531,480 (267,502,187,133–350,999,698,311)
Belgium	Group I	13,907,602,879 (12,591,730,574–15,402,303,221)	13,758,531,431 (12,414,896,765–15,458,314,431)	13,816,972,097 (12,431,775,547–15,567,188,484)	13,655,824,607 (12,150,689,549–15,646,746,952)
Belgium	NCD	258,418,862,593 (230,072,120,854–290,742,908,650)	254,590,819,220 (224,009,058,017–287,735,388,673)	250,621,497,948 (219,484,279,695–284,894,123,549)	262,502,863,576 (227,278,220,692–300,404,638,322)
Belgium	Injuries	34,994,488,239 (31,370,688,260–39,231,049,444)	34,183,853,069 (30,372,703,216–38,764,055,992)	32,606,776,074 (28,559,707,620–37,989,529,751)	31,122,843,297 (26,470,567,310–36,862,036,649)
Cyprus	All causes	18,010,413,650 (16,078,614,964–20,058,500,440)	18,507,096,590 (16,409,808,429–20,819,579,276)	19,027,414,749 (16,742,387,581–21,645,741,644)	21,649,807,617 (18,733,165,997–24,726,865,736)
Cyprus	Group I	1,341,227,908 (1,183,219,056–1,522,935,932)	937,491,016 (823,017,761–1,072,861,869)	849,645,750 (737,939,772–977,318,813)	839,582,693 (704,821,169–988,482,731)
Cyprus	NCD	14,625,473,857 (12,916,675,972–16,421,882,393)	15,352,982,241 (13,498,947,303–17,434,162,230)	16,065,300,093 (14,081,056,033–18,371,159,842)	18,719,844,574 (16,088,420,900–21,423,304,642)
Cyprus	Injuries	2,043,711,886 (1,836,526,142–2,299,964,176)	2,216,623,333 (1,990,787,266–2,510,534,662)	2,112,468,906 (1,860,919,252–2,434,720,808)	2,090,380,350 (1,773,456,411–2,485,891,480)
Denmark	All causes	168,913,242,566 (151,336,633,233–187,928,271,182)	161,809,116,358 (143,659,638,996–181,282,097,267)	157,193,734,783 (138,386,829,748–177,786,520,837)	154,647,568,205 (133,915,380,043–177,063,656,653)
Denmark	Group I	7,150,827,256 (6,490,830,091–7,952,736,993)	6,336,880,575 (5,675,042,834–7,176,576,745)	6,507,880,056 (5,839,841,747–7,309,876,666)	6,338,477,077 (5,541,343,508–7,276,834,248)
Denmark	NCD	144,128,958,413 (128,654,499,317–160,702,645,466)	141,460,408,777 (125,742,758,864–158,765,679,340)	138,311,727,120 (121,560,612,741–156,318,088,364)	137,072,431,600 (118,634,804,733–156,898,255,948)
Denmark	Injuries	17,633,456,896 (15,507,839,979–20,241,760,998)	14,011,827,006 (12,111,819,912–16,421,206,177)	12,374,127,607 (10,514,759,191–14,699,361,162)	11,236,659,528 (9,111,357,403–13,885,768,425)
Finland	All causes	158,552,060,208 (141,761,557,145–177,111,858,078)	151,198,294,760 (133,617,745,451–171,185,704,708)	151,764,354,645 (133,426,933,209–171,979,194,277)	148,216,120,941 (128,186,926,036–169,687,038,668)
Finland	Group I	7,548,573,304 (6,831,164,280–8,422,563,330)	6,261,536,662 (5,576,356,054–7,102,880,263)	4,780,369,723 (4,111,880,792–5,557,159,392)	3,921,081,825 (3,246,631,556–4,741,949,235)
Finland	NCD	129,716,188,085 (115,311,991,287–145,783,440,238)	126,228,135,614 (110,623,030,019–142,909,379,830)	128,779,195,283 (113,072,826,355–146,275,476,651)	129,659,736,622 (111,731,595,565–148,585,906,559)
Finland	Injuries	21,287,298,818 (18,992,414,420–23,758,465,911)	18,708,622,483 (16,416,304,558–21,615,596,466)	18,204,789,639 (15,809,504,911–21,373,828,132)	14,635,302,495 (12,147,656,539–17,589,124,191)
France	All causes	1,595,705,329,110 (1,422,996,888,080–1,791,255,907,510)	1,574,987,806,690 (1,393,699,815,800–1,780,168,648,610)	1,568,955,163,700 (1,380,149,393,750–1,786,593,352,720)	1,624,013,265,860 (1,407,143,935,030–1,864,587,424,350)
France	Group I	87,857,786,435 (80,951,770,735–96,302,351,060)	74,054,928,787 (66,981,893,711–82,210,094,923)	68,883,681,562 (61,791,003,733–77,791,007,529)	67,065,166,285 (59,417,564,143–76,807,902,580)
France	NCD	1,282,953,900,940 (1,131,412,882,440–1,449,458,092,270)	1,302,174,351,930 (1,141,165,286,780–1,486,234,010,650)	1,320,440,540,300 (1,148,963,791,310–1,511,557,582,960)	1,388,806,067,480 (1,195,681,192,490–1,595,784,894,910)
France	Injuries	224,893,641,738 (204,561,389,577–250,875,059,461)	198,758,525,971 (177,243,562,743–224,866,369,561)	179,630,941,840 (157,641,729,676–207,112,474,822)	168,142,032,093 (142,322,341,664–198,070,286,496)
Germany	All causes	2,594,303,001,910 (2,330,145,456,010–2,885,866,055,690)	2,427,715,340,280 (2,145,833,211,840–2,742,153,147,470)	2,362,013,541,170 (2,075,914,649,900–2,667,149,569,850)	2,416,997,149,430 (2,098,432,363,610–2,773,497,309,560)
Germany	Group I	107,737,648,211 (96,240,369,156–121,210,815,864)	87,406,845,273 (76,377,755,738–101,019,966,016)	85,979,720,428 (75,363,239,893–98,766,255,073)	85,353,390,118 (74,057,990,488–99,122,692,773)
Germany	NCD	2,253,720,880,680 (2,021,108,201,380–2,512,531,336,370)	2,141,097,499,210 (1,894,865,020,800–2,423,488,714,020)	2,096,749,320,470 (1,843,981,191,780–2,367,641,489,020)	2,157,608,668,120 (1,875,031,445,080–2,465,699,860,600)
Germany	Injuries	232,844,473,020 (205,180,625,993–268,974,559,410)	199,210,995,804 (172,169,338,923–232,586,593,780)	179,284,500,271 (151,092,022,510–212,574,693,158)	174,035,091,194 (143,140,768,086–211,584,240,097)
Greece	All causes	283,968,606,860 (253,215,171,386–319,556,724,585)	301,207,550,661 (266,652,500,414–340,390,410,623)	303,246,034,251 (267,685,365,250–344,081,876,902)	310,417,902,751 (272,066,088,443–350,851,460,221)
Greece	Group I	12,661,139,436 (11,162,128,102–14,658,898,142)	11,499,619,037 (10,096,473,457–13,258,090,230)	10,550,291,518 (9,147,273,368–12,330,772,883)	9,875,100,450 (8,392,944,358–11,555,926,703)
Greece	NCD	243,143,187,261 (215,996,310,694–273,855,657,996)	261,620,795,723 (230,744,892,371–296,000,198,440)	266,928,479,918 (234,896,732,146–302,120,993,469)	277,582,130,150 (243,925,384,961–313,403,330,796)
Greece	Injuries	28,164,280,164 (24,759,749,050–32,529,435,367)	28,087,135,902 (24,404,096,742–32,659,589,261)	25,767,262,815 (21,875,341,826–30,435,391,938)	22,960,672,151 (18,966,974,256–28,120,329,573)
Iceland	All causes	5,966,607,947 (5,230,446,199–6,795,104,601)	6,159,101,263 (5,351,675,234–7,080,844,442)	6,408,341,044 (5,513,735,157–7,440,702,383)	7,151,865,603 (6,094,620,984–8,304,814,880)
Iceland	Group I	366,335,930 (331,899,736–411,571,418)	300,619,417 (265,276,086–347,427,128)	284,384,671 (247,405,007–331,269,869)	281,814,877 (238,762,993–331,710,598)
Iceland	NCD	4,938,171,706 (4,285,603,605–5,654,117,283)	5,252,322,981 (4,528,312,174–6,068,783,573)	5,528,961,594 (4,735,991,994–6,458,317,432)	6,239,622,446 (5,300,736,521–7,280,493,591)
Iceland	Injuries	662,100,311 (593,489,527–750,812,478)	606,158,865 (526,390,440–702,701,821)	594,994,779 (507,355,794–705,680,910)	630,428,280 (523,799,141–771,588,634)
Ireland	All causes	97,361,760,590 (86,930,897,781–109,194,748,656)	99,378,409,264 (87,514,832,654–111,831,483,429)	98,537,486,910 (85,619,955,729–112,909,394,500)	104,610,002,005 (89,845,453,770–121,849,775,294)
Ireland	Group I	5,648,721,470 (5,139,250,182–6,311,002,993)	5,478,219,591 (4,959,693,281–6,108,745,846)	4,531,295,438 (3,974,693,146–5,222,154,614)	4,284,432,497 (3,651,603,690–5,067,037,155)
Ireland	NCD	82,862,793,671 (73,551,028,179–92,941,450,111)	84,253,242,203 (73,691,440,472–95,561,529,648)	84,603,975,426 (73,125,859,264–97,196,319,667)	91,433,828,515 (78,361,544,835–106,513,997,927)
Ireland	Injuries	8,850,245,448 (7,838,096,619–10,122,483,596)	9,646,947,470 (8,423,651,973–11,139,413,297)	9,402,216,046 (8,083,105,966–11,084,298,307)	8,891,740,993 (7,293,870,620–10,989,042,227)
Israel	All causes	107,160,364,914 (94,454,745,531–121,102,694,306)	132,043,280,294 (114,523,508,605–151,471,694,894)	142,356,581,147 (123,333,631,815–163,750,684,558)	157,629,823,308 (132,400,248,119–184,716,869,298)
Israel	Group I	9,081,561,056 (8,266,966,939–10,231,274,952)	8,917,522,087 (7,940,509,995–10,059,068,941)	8,787,115,552 (7,765,740,258–10,065,004,458)	9,012,127,083 (7,711,145,534–10,529,440,476)
Israel	NCD	87,162,847,871 (76,160,073,230–99,425,037,835)	109,165,992,334 (93,678,670,143–125,485,826,390)	116,282,546,404 (99,440,741,995–134,573,227,782)	133,744,742,147 (112,070,988,810–157,116,916,220)
Israel	Injuries	10,915,955,987 (9,586,984,263–12,574,674,317)	13,959,765,872 (12,085,226,217–16,370,459,551)	17,286,919,191 (15,042,589,540–19,964,183,158)	14,872,954,079 (11,856,111,656–18,456,604,074)
Italy	All causes	1,656,682,858,590 (1,474,028,496,480–1,860,124,492,180)	1,583,846,741,080 (1,396,177,230,970–1,793,856,326,490)	1,559,657,417,110 (1,365,883,785,840–1,779,160,375,290)	1,629,251,868,240 (1,414,034,316,250–1,861,377,645,740)
Italy	Group I	77,809,344,842 (69,904,585,998–86,895,056,060)	58,571,709,649 (51,199,222,621–67,442,456,497)	55,256,531,235 (47,630,927,073–64,778,015,070)	49,916,883,573 (41,525,511,143–59,904,175,184)
Italy	NCD	1,423,258,200,620 (1,261,453,248,560–1,601,619,891,180)	1,386,667,868,610 (1,222,164,890,500–1,571,392,883,680)	1,377,845,396,140 (1,206,243,883,160–1,567,469,577,050)	1,454,934,424,870 (1,267,226,500,100–1,657,999,424,800)
Italy	Injuries	155,615,313,127 (136,011,795,373–179,481,748,890)	138,607,162,819 (118,025,841,119–163,608,408,067)	126,555,489,731 (105,542,031,294–152,373,443,016)	124,400,559,802 (101,099,662,770–153,301,736,999)
Luxembourg	All causes	12,165,733,838 (10,898,287,272–13,597,956,634)	11,654,547,141 (10,215,825,639–13,277,264,899)	11,600,439,808 (10,095,825,558–13,334,801,662)	13,463,844,306 (11,494,866,379–15,571,272,675)
Luxembourg	Group I	515,841,714 (464,317,067–577,467,911)	477,538,783 (421,009,426–545,517,155)	449,872,562 (392,307,028–523,241,949)	497,790,895 (425,039,220–583,527,113)
Luxembourg	NCD	10,247,634,715 (9,126,925,029–11,525,096,170)	9,974,800,032 (8,730,809,705–11,397,321,695)	10,068,908,886 (8,665,724,267–11,618,229,515)	11,794,790,977 (10,020,139,411–13,695,798,928)
Luxembourg	Injuries	1,402,257,409 (1,246,944,271–1,590,221,776)	1,202,208,326 (1,042,245,959–1,403,505,755)	1,081,658,360 (920,327,382–1,286,538,393)	1,171,262,433 (968,816,406–1,430,209,278)
Malta	All causes	9,078,164,238 (7,945,588,950–10,272,637,267)	9,692,811,879 (8,419,025,558–11,028,870,916)	9,956,954,234 (8,720,695,184–11,326,411,560)	10,620,795,941 (8,954,590,427–12,392,864,420)
Malta	Group I	613,537,101 (554,908,287–689,804,798)	538,093,878 (480,361,166–605,958,704)	467,845,250 (414,248,807–532,864,288)	449,827,679 (384,421,551–526,377,151)
Malta	NCD	7,748,604,952 (6,746,814,260–8,790,407,870)	8,410,003,782 (7,297,496,302–9,617,864,999)	8,736,019,394 (7,630,664,976–9,899,521,616)	9,413,676,636 (7,936,301,692–10,973,061,016)
Malta	Injuries	716,022,186 (598,067,469–853,591,257)	744,714,218 (622,521,389–906,556,815)	753,089,590 (618,283,861–917,954,926)	757,291,627 (596,230,239–966,337,348)
Netherlands	All causes	407,243,780,293 (361,856,295,197–459,018,614,466)	431,043,486,948 (379,747,841,063–490,773,562,404)	418,284,559,558 (364,080,314,674–479,423,362,187)	435,778,449,108 (373,600,015,343–501,580,985,168)
Netherlands	Group I	19,841,564,095 (17,695,501,110–22,233,094,938)	21,899,978,122 (19,723,417,841–24,582,305,494)	19,927,750,539 (17,737,840,711–22,590,030,645)	18,872,190,767 (16,398,062,147–21,609,581,762)

Appendix Table 3. GBD 2016 DALY counts for all causes and level 1 cause groups by location for 1990, 2000, 2006 & 2016, both sexes combined.

	Cause	1990	2000	2006	2016
Netherlands	NCD	354,546,227,370 (313,438,049,003–401,013,497,922)	378,899,023,496 (332,835,610,151–432,861,222,647)	370,254,995,526 (321,178,188,615–424,306,588,613)	386,609,244,540 (330,861,842,833–445,632,011,420)
Netherlands	Injuries	32,855,988,828 (28,760,421,392–37,924,819,046)	30,244,485,331 (26,380,115,034–35,002,794,279)	28,101,813,493 (23,897,334,565–33,380,879,213)	30,297,013,801 (24,802,905,506–37,120,763,239)
Norway	All causes	125,801,078,653 (112,225,238,374–141,168,049,887)	120,613,644,182 (106,916,586,219–136,017,473,390)	115,626,484,972 (100,958,651,647–131,833,142,127)	121,237,268,091 (104,097,207,497–139,121,206,425)
Norway	Group I	6,700,239,477 (6,139,248,667–7,375,015,599)	5,294,623,731 (4,692,249,318–6,044,545,684)	4,935,761,492 (4,364,458,553–5,634,671,703)	4,840,950,172 (4,150,601,616–5,697,974,458)
Norway	NCD	106,176,620,762 (93,841,025,938–119,769,892,959)	104,166,642,323 (91,633,123,110–118,053,026,173)	100,354,964,412 (87,534,375,598–114,456,525,793)	105,745,105,044 (90,155,509,414–122,201,988,627)
Norway	Injuries	12,924,218,413 (11,471,595,145–14,662,736,633)	11,152,378,129 (9,722,839,202–12,945,288,634)	10,335,759,068 (8,836,964,213–12,258,873,534)	10,651,212,875 (8,814,121,848–12,898,726,862)
Portugal	All causes	320,482,312,822 (290,720,636,074–356,372,325,653)	315,589,917,451 (281,518,186,811–353,407,677,800)	302,189,104,563 (268,262,705,179–341,278,691,938)	295,588,634,363 (258,152,234,620–337,672,250,765)
Portugal	Group I	21,266,344,484 (19,530,428,769–23,481,476,396)	23,151,819,347 (21,171,639,794–25,738,107,076)	20,034,231,046 (18,040,976,138–22,597,794,520)	17,064,447,627 (14,833,642,283–19,904,524,608)
Portugal	NCD	257,602,404,937 (231,653,129,145–288,596,828,964)	260,148,514,881 (230,519,303,523–293,765,055,424)	255,026,383,633 (225,199,444,541–289,086,344,528)	255,666,061,804 (222,080,245,300–293,304,818,724)
Portugal	Injuries	41,613,563,400 (37,967,912,982–46,250,609,933)	32,289,583,224 (28,633,334,957–36,796,654,026)	27,128,489,884 (23,565,218,560–31,416,979,514)	22,858,124,932 (19,321,926,478–27,185,170,685)
Spain	All causes	1,050,244,634,480 (933,623,106,541–1,183,337,670,230)	1,060,591,994,900 (937,655,206,327–1,204,874,649,930)	1,099,945,800,810 (961,931,920,712–1,258,389,196,860)	1,113,759,511,680 (963,724,810,708–1,273,384,744,630)
Spain	Group I	60,718,936,160 (55,040,993,400–67,673,651,022)	52,210,094,064 (46,389,875,631–59,956,451,796)	51,344,981,202 (45,016,328,798–59,716,876,481)	45,002,973,115 (38,487,523,484–53,416,446,315)
Spain	NCD	876,224,608,553 (774,482,844,423–990,196,640,943)	908,637,641,394 (797,418,876,051–1,031,836,837,040)	948,880,653,920 (826,129,427,978–1,084,633,192,460)	985,087,497,770 (854,044,879,555–1,128,877,060,980)
Spain	Injuries	113,301,089,763 (100,811,804,022–128,468,118,985)	99,744,259,444 (85,958,388,815–116,791,738,981)	99,720,165,688 (83,067,473,309–119,572,731,269)	83,669,040,790 (67,816,895,476–103,754,277,486)
Sweden	All causes	260,231,271,729 (231,854,595,782–292,370,498,296)	246,413,833,132 (216,564,739,223–280,246,277,145)	242,930,653,472 (212,148,384,978–277,648,598,647)	253,427,313,979 (218,043,901,723–293,369,900,371)
Sweden	Group I	11,358,336,267 (10,339,523,106–12,670,193,736)	8,739,426,666 (7,685,127,243–10,022,920,319)	8,481,694,590 (7,411,572,910–9,849,707,327)	9,005,835,483 (7,712,484,698–10,565,325,391)
Sweden	NCD	224,724,129,910 (198,837,179,604–253,487,186,825)	217,174,249,979 (190,572,259,304–247,379,531,400)	214,032,282,524 (186,074,255,341–244,229,611,395)	223,274,047,112 (191,229,449,565–258,789,432,200)
Sweden	Injuries	24,148,805,552 (21,218,722,527–27,803,416,338)	20,500,156,488 (17,617,108,244–24,178,718,235)	20,416,676,358 (17,359,162,434–24,477,650,014)	21,147,431,384 (17,367,864,257–25,904,276,184)
Switzerland	All causes	194,294,947,149 (170,814,284,723–218,652,300,142)	190,450,673,381 (164,949,912,871–217,678,361,481)	188,768,039,609 (162,583,608,077–218,032,139,917)	199,290,167,176 (165,572,038,633–236,966,178,869)
Switzerland	Group I	10,477,750,001 (9,190,665,173–12,064,456,170)	9,460,747,739 (8,121,347,006–11,105,997,559)	8,814,091,004 (7,371,948,585–10,472,826,416)	9,072,520,231 (7,510,833,645–10,997,175,535)
Switzerland	NCD	157,663,384,662 (137,677,271,989–178,043,254,448)	159,851,461,465 (138,055,804,981–183,145,252,778)	161,016,932,132 (138,019,758,634–185,966,578,715)	172,733,618,711 (143,322,370,553–204,924,882,451)
Switzerland	Injuries	26,153,812,486 (23,105,270,197–30,007,743,206)	21,138,464,177 (18,116,801,726–24,737,494,613)	18,937,016,472 (15,950,740,320–22,562,447,212)	17,484,028,234 (13,756,136,831–22,159,173,630)
United Kingdom	All causes	1,831,233,500,480 (1,644,976,601,400–2,040,657,389,850)	1,706,596,779,390 (1,514,102,971,720–1,924,433,292,890)	1,663,360,082,270 (1,463,788,082,870–1,886,677,113,180)	1,723,473,647,690 (1,504,758,199,160–1,967,135,920,010)
United Kingdom	Group I	108,609,423,537 (100,068,984,598–119,396,895,588)	99,422,874,663 (91,348,824,042–109,987,096,024)	90,354,682,364 (82,493,514,202–100,630,411,302)	90,709,715,614 (81,554,924,874–102,411,187,131)
United Kingdom	NCD	1,592,441,072,600 (1,423,309,953,630–1,781,097,456,700)	1,489,783,675,180 (1,317,549,903,320–1,680,911,936,410)	1,457,285,461,040 (1,278,244,389,820–1,656,040,168,900)	1,516,139,115,260 (1,317,373,549,670–1,733,410,631,330)
United Kingdom	Injuries	130,183,004,340 (113,589,419,810–152,588,394,912)	117,390,229,544 (100,602,496,790–139,724,807,172)	115,719,938,875 (97,640,979,045–139,514,582,684)	116,624,816,822 (96,020,753,305–143,267,539,626)
England	All causes	1,513,112,130,440 (1,359,382,988,670–1,687,635,962,220)	1,410,664,534,830 (1,249,869,647,470–1,590,989,632,460)	1,374,543,088,000 (1,207,307,284,910–1,563,727,862,980)	1,426,264,327,690 (1,244,017,795,080–1,632,078,952,510)
England	Group I	90,508,217,227 (83,260,508,033–99,572,594,969)	83,872,476,542 (76,803,272,175–92,736,469,510)	76,231,459,412 (69,709,774,357–85,014,421,047)	77,186,340,915 (69,336,586,450–87,417,126,321)
England	NCD	1,316,942,857,340 (1,176,222,168,270–1,474,250,975,490)	1,231,629,820,950 (1,085,701,079,490–1,392,153,011,510)	1,204,464,900,880 (1,053,352,433,250–1,373,152,976,220)	1,254,179,126,510 (1,086,837,225,570–1,436,114,559,940)
England	Injuries	105,661,055,873 (91,551,545,006–124,302,492,019)	95,162,237,337 (80,859,152,116–113,626,797,829)	93,846,727,707 (78,462,222,673–113,494,517,016)	94,898,860,269 (77,713,136,776–117,127,588,061)
Northern Ireland	All causes	46,996,034,214 (41,959,254,142–52,624,299,130)	44,777,940,234 (39,516,645,600–50,862,546,512)	45,247,731,640 (39,345,674,623–51,366,207,841)	47,974,867,581 (40,963,319,580–55,133,570,977)
Northern Ireland	Group I	3,451,125,444 (3,158,406,930–3,785,225,011)	2,886,183,231 (2,622,840,723–3,191,515,410)	2,564,762,014 (2,312,138,635–2,856,653,904)	2,439,366,093 (2,103,332,177–2,805,986,665)
Northern Ireland	NCD	39,440,016,113 (34,934,763,913–44,384,724,803)	38,145,166,268 (33,399,391,892–43,446,024,927)	38,674,792,471 (33,422,794,606–44,041,560,227)	41,479,982,547 (35,207,262,670–47,759,474,282)
Northern Ireland	Injuries	4,104,892,657 (3,582,937,512–4,748,147,014)	3,746,590,735 (3,199,042,091–4,390,308,613)	4,008,177,155 (3,341,426,926–4,750,598,155)	4,055,518,941 (3,308,894,327–4,995,263,180)
Scotland	All causes	176,131,129,556 (159,230,456,103–195,668,454,521)	162,396,986,727 (145,732,139,410–182,221,776,456)	157,767,464,292 (140,269,350,373–176,553,789,542)	160,567,991,135 (139,604,442,760–182,778,338,309)
Scotland	Group I	9,772,658,643 (8,959,450,332–10,751,768,212)	7,948,651,961 (7,235,824,187–8,838,375,003)	7,385,846,742 (6,674,826,741–8,235,398,423)	6,973,725,539 (6,095,712,469–7,942,094,322)
Scotland	NCD	152,227,211,817 (137,028,958,256–169,315,091,196)	142,009,464,540 (127,060,275,668–159,054,321,905)	138,419,634,630 (123,089,172,087–155,328,109,144)	141,959,306,695 (123,070,403,582–161,353,828,422)
Scotland	Injuries	14,131,259,096 (12,099,104,421–16,294,598,082)	12,438,870,226 (10,339,277,041–14,651,664,487)	11,961,982,920 (9,804,894,009–14,175,934,801)	11,634,958,901 (9,496,375,643–14,303,969,566)
Wales	All causes	94,994,200,191 (85,196,278,549–105,887,077,809)	88,757,308,097 (78,719,871,270–99,525,563,688)	85,801,794,740 (75,749,381,048–96,711,633,154)	88,666,455,725 (76,901,812,794–101,269,208,885)
Wales	Group I	4,877,422,058 (4,423,350,824–5,396,922,281)	4,715,562,637 (4,268,896,413–5,244,745,816)	4,172,614,088 (3,718,033,046–4,685,150,695)	4,110,282,878 (3,613,206,481–4,696,714,012)
Wales	NCD	83,830,981,806 (75,062,168,090–93,418,152,628)	77,999,214,881 (68,909,815,424–87,767,665,483)	75,726,129,807 (66,558,015,090–85,285,755,819)	78,520,694,608 (68,075,423,042–89,897,379,859)
Wales	Injuries	6,285,796,327 (5,431,651,929–7,304,575,321)	6,042,530,579 (5,124,499,960–7,151,946,639)	5,903,050,846 (4,947,864,012–7,103,005,257)	6,035,478,240 (4,935,398,891–7,436,477,357)
Southern Latin America	All causes	1,461,620,097,770 (1,328,882,571,890–1,611,642,200,920)	1,493,743,249,850 (1,341,126,488,760–1,664,080,206,660)	1,527,270,384,050 (1,365,361,123,570–1,712,414,164,460)	1,652,998,459,360 (1,453,703,270,510–1,872,711,445,040)
Southern Latin America	Group I	229,112,210,058 (217,881,264,470–243,247,064,855)	182,209,291,054 (171,484,672,491–195,589,214,761)	166,408,868,861 (155,445,663,301–179,050,164,006)	157,659,373,843 (144,401,659,121–172,905,210,316)
Southern Latin America	NCD	1,070,356,924,090 (951,494,361,647–1,200,533,033,640)	1,142,284,768,420 (1,005,639,176,630–1,292,044,773,580)	1,194,717,925,090 (1,050,283,013,570–1,360,035,631,410)	1,317,809,328,820 (1,140,312,095,810–1,509,459,523,820)
Southern Latin America	Injuries	162,150,963,613 (150,833,775,152–175,794,800,728)	169,249,190,378 (157,330,328,580–184,218,544,219)	166,143,590,099 (153,607,481,968–182,054,282,021)	177,529,756,699 (158,516,475,711–200,794,056,703)
Argentina	All causes	1,011,053,823,440 (922,161,529,994–1,111,417,178,490)	1,046,881,553,720 (947,197,026,810–1,160,338,708,260)	1,064,365,793,740 (954,311,274,790–1,184,141,490,790)	1,137,385,720,670 (1,006,880,603,060–1,276,866,101,760)
Argentina	Group I	165,748,626,928 (157,543,518,357–175,854,117,587)	141,059,496,534 (132,569,580,499–151,498,914,095)	131,293,383,098 (122,877,708,364–141,260,353,533)	123,635,698,372 (112,780,896,125–135,633,202,407)
Argentina	NCD	742,357,102,135 (663,171,395,234–831,615,008,805)	788,773,758,197 (700,266,911,221–890,149,968,435)	817,830,045,001 (722,570,878,956–924,623,053,623)	890,638,038,414 (776,499,356,717–1,015,754,023,410)
Argentina	Injuries	102,948,094,380 (95,170,071,314–111,968,655,391)	117,048,298,994 (108,128,358,737–127,890,412,458)	115,242,365,642 (106,210,290,879–127,098,602,527)	123,111,983,883 (110,126,961,594–138,515,477,870)
Chile	All causes	351,085,504,452 (314,255,203,198–390,477,462,000)	348,713,505,935 (305,614,817,167–396,798,747,701)	367,508,579,020 (320,611,432,710–421,599,754,044)	419,046,761,910 (348,150,139,978–500,335,503,675)
Chile	Group I	52,473,802,784 (48,469,082,805–56,837,714,575)	32,251,780,988 (29,668,651,227–35,447,961,460)	27,371,508,091 (24,749,242,342–30,558,516,886)	27,257,270,452 (23,563,882,008–31,500,488,003)
Chile	NCD	249,602,400,711 (216,985,394,266–283,263,102,638)	275,253,368,907 (236,104,022,175–319,212,611,716)	299,704,569,497 (257,495,022,853–347,519,160,040)	348,502,390,865 (287,578,899,323–417,630,195,950)
Chile	Injuries	49,009,300,957 (44,327,290,144–53,647,646,368)	41,208,356,040 (37,885,366,882–45,308,045,388)	40,432,501,432 (36,815,983,284–44,769,944,536)	43,287,100,593 (34,512,692,905–54,155,157,981)
Uruguay	All causes	99,458,779,385 (90,928,567,880–109,094,891,934)	98,114,518,249 (88,859,394,933–108,254,998,933)	95,360,725,550 (86,055,861,542–105,852,050,183)	96,528,907,415 (85,858,017,313–108,610,983,472)
Uruguay	Group I	10,888,536,494 (10,021,356,346–11,695,281,049)	8,896,460,052 (8,223,372,222–9,634,541,828)	7,742,443,345 (7,099,733,134–8,452,487,331)	6,764,994,426 (5,947,767,209–7,783,826,893)
Uruguay	NCD	78,377,938,149 (71,023,735,463–87,188,328,409)	78,227,448,889 (69,922,725,424–87,288,097,003)	77,151,598,106 (68,737,829,358–86,447,309,206)	78,635,638,380 (69,223,852,881–88,940,240,895)
Uruguay	Injuries	10,192,304,741 (9,363,794,811–11,134,355,013)	10,990,609,308 (10,166,041,330–12,081,379,257)	10,466,684,098 (9,574,680,284–11,480,026,402)	11,128,274,609 (9,930,855,272–12,425,489,197)
Central Europe, Eastern Europe, and Central Asia	All causes	15,924,897,480,000 (14,579,985,925,300–17,398,497,745,900)	17,538,216,540,400 (16,182,752,394,000–19,001,602,148,100)	17,098,909,964,100 (15,724,359,650,300–18,623,621,980,400)	15,455,985,226,300 (13,587,818,476,500–17,516,957,328,500)
Central Europe, Eastern Europe, and Central Asia	Group I	2,067,202,837,250 (1,953,799,966,550–2,210,532,157,830)	1,733,298,231,210 (1,615,356,713,770–1,863,650,933,150)	1,579,947,956,090 (1,469,359,765,040–1,721,755,918,740)	1,264,295,055,400 (1,124,502,877,800–1,417,426,098,180)
Central Europe, Eastern Europe, and Central Asia	NCD	11,420,914,168,800 (10,384,524,769,900–12,566,239,940,000)	13,002,550,239,500 (11,916,209,911,700–14,174,976,270,800)	13,004,898,933,000 (11,875,559,683,300–14,216,279,908,400)	12,187,392,117,000 (10,673,628,643,500–13,828,292,470,000)
Central Europe, Eastern Europe, and Central Asia	Injuries	2,436,780,474,000 (2,205,833,521,500–2,689,481,821,640)	2,802,368,069,670 (2,538,914,541,980–3,074,607,511,760)	2,514,063,074,920 (2,277,708,763,240–2,785,641,546,910)	2,004,298,053,850 (1,677,250,741,430–2,387,437,386,850)
Eastern Europe	All causes	8,815,043,182,400 (8,019,363,021,070–9,622,586,456,260)	10,690,551,762,200 (9,801,165,900,970–11,640,937,914,300)	10,285,942,503,000 (9,448,833,333,350–11,181,037,153,000)	8,952,733,918,260 (7,588,995,034,970–10,516,202,204,400)
Eastern Europe	Group I	648,503,670,688 (600,582,999,913–704,850,727,454)	725,764,180,131 (670,501,544,358–785,434,957,944)	717,167,858,412 (663,909,156,936–772,635,689,025)	586,722,903,354 (511,723,328,869–665,717,747,104)
Eastern Europe	NCD	6,628,452,510,920 (6,006,516,362,560–7,268,616,982,240)	7,984,098,945,630 (7,314,314,389,080–8,730,058,123,930)	7,857,086,696,800 (7,183,145,534,340–8,573,266,651,500)	7,089,749,381,320 (5,984,654,189,820–8,329,647,114,460)
Eastern Europe	Injuries	1,538,087,000,800 (1,381,244,033,550–1,708,724,196,030)	1,980,688,636,400 (1,779,739,155,630–2,188,156,468,920)	1,711,687,947,780 (1,538,150,086,750–1,904,526,247,420)	1,276,261,633,590 (1,009,727,657,100–1,599,256,174,020)
Belarus	All causes	377,557,733,501 (343,457,030,769–414,065,762,833)	440,057,491,504 (402,370,386,840–477,805,421,500)	429,757,575,442 (395,090,795,781–469,046,967,013)	363,591,403,265 (311,861,721,557–412,814,796,164)
Belarus	Group I	25,677,857,570 (22,389,685,648–29,451,386,588)	21,304,937,562 (19,077,225,793–23,993,949,484)	19,193,798,975 (17,326,924,364–21,539,040,488)	14,536,646,323 (12,642,991,338–16,741,573,393)
Belarus	NCD	290,836,282,051 (263,141,710,191–320,577,895,832)	342,573,373,220 (311,969,018,956–372,310,838,953)	340,219,877,969 (312,454,712,702–370,427,663,933)	297,112,180,958 (256,020,683,883–337,346,375,913)
Belarus	Injuries	61,043,593,880 (54,951,510,265–67,812,631,206)	76,179,180,723 (69,258,820,285–83,699,676,663)	70,343,898,498 (63,840,837,228–77,890,872,596)	51,942,575,985 (42,713,922,578–62,068,603,660)
Estonia	All causes	62,439,352,906 (57,018,187,685–68,382,522,026)	58,075,267,008 (53,041,338,214–63,531,704,917)	52,486,249,510 (47,282,917,711–57,705,517,436)	44,360,083,897 (38,935,894,328–50,338,717,790)

Appendix Table 3. GBD 2016 DALY counts for all causes and level 1 cause groups by location for 1990, 2000, 2006 & 2016, both sexes combined.

	Cause	1990	2000	2006	2016
Estonia	Group I	3,579,801,302 (3,248,522,971–3,959,917,149)	2,848,767,789 (2,575,376,292–3,174,453,362)	2,329,695,854 (2,067,491,286–2,621,754,127)	1,637,102,321 (1,396,784,983–1,928,297,844)
Estonia	NCD	47,386,454,865 (43,053,981,744–52,226,103,896)	45,449,416,309 (41,315,547,108–49,920,240,684)	42,642,246,449 (38,332,452,561–47,021,494,058)	37,607,646,147 (33,081,987,947–42,661,092,872)
Estonia	Injuries	11,473,096,738 (10,481,648,028–12,632,989,823)	9,777,082,910 (8,840,813,950–10,851,146,048)	7,514,307,208 (6,716,887,628–8,454,482,447)	5,115,335,429 (4,223,836,994–6,192,044,868)
Latvia	All causes	110,387,264,534 (101,109,385,887–120,529,247,941)	99,390,864,368 (90,386,001,313–108,928,150,516)	98,825,770,360 (90,500,996,413–107,504,338,069)	77,341,777,951 (68,231,249,856–86,864,354,374)
Latvia	Group I	6,392,531,214 (5,866,181,301–7,018,635,267)	5,023,622,859 (4,555,666,388–5,607,762,427)	4,594,881,855 (4,152,043,935–5,112,863,219)	3,222,405,256 (2,796,653,845–3,673,897,944)
Latvia	NCD	83,262,435,914 (75,871,159,164–91,152,445,303)	77,043,962,204 (69,808,770,386–84,659,402,219)	79,584,994,876 (72,715,436,166–86,609,064,803)	64,789,321,370 (57,262,641,185–72,393,937,104)
Latvia	Injuries	20,732,297,405 (18,929,038,170–22,749,191,328)	17,323,279,306 (15,576,951,455–19,250,669,089)	14,645,893,629 (13,289,131,344–16,220,645,817)	9,330,051,325 (7,941,591,133–10,980,918,900)
Lithuania	All causes	133,776,984,792 (121,772,533,677–146,864,367,450)	129,285,548,989 (117,048,021,869–141,769,959,940)	138,401,037,525 (126,440,933,927–151,374,978,507)	111,620,655,586 (100,170,486,918–124,668,991,716)
Lithuania	Group I	7,353,629,439 (6,625,160,236–8,251,977,755)	6,507,207,361 (5,831,571,779–7,315,141,418)	6,567,146,678 (5,921,808,159–7,300,831,875)	4,459,199,924 (3,931,733,047–5,068,217,008)
Lithuania	NCD	100,863,913,432 (90,951,020,479–111,306,828,392)	98,020,217,554 (88,052,907,291–108,201,249,690)	107,515,074,792 (97,884,345,501–118,135,197,597)	90,925,739,515 (81,306,360,986–101,152,359,942)
Lithuania	Injuries	25,559,441,921 (23,250,788,691–28,212,962,159)	24,758,124,075 (22,504,063,847–27,291,616,454)	24,318,816,054 (21,995,457,175–26,867,412,468)	16,235,716,147 (14,265,275,737–18,737,351,553)
Moldova	All causes	175,523,977,674 (159,676,736,071–191,835,241,736)	163,572,088,813 (147,981,358,616–180,047,467,682)	161,137,142,739 (145,980,437,569–176,781,734,715)	145,215,646,096 (127,902,326,722–162,548,193,416)
Moldova	Group I	22,299,670,832 (19,443,847,993–25,762,371,098)	17,503,344,241 (15,386,206,166–19,878,958,949)	14,436,018,220 (12,716,341,247–16,212,114,418)	10,433,192,360 (8,939,215,966–12,219,550,391)
Moldova	NCD	124,626,301,410 (112,458,791,154–136,742,123,481)	123,700,092,664 (111,656,432,647–136,866,687,919)	126,822,994,590 (114,553,726,867–140,029,062,141)	118,716,209,046 (104,883,857,645–133,380,103,327)
Moldova	Injuries	28,598,005,431 (25,988,420,701–31,522,349,690)	22,368,651,908 (20,114,215,714–24,889,213,050)	19,878,129,928 (17,727,396,319–22,260,224,092)	16,066,244,690 (13,608,689,097–18,669,322,875)
Russia	All causes	5,883,494,353,980 (5,282,096,460,580–6,445,413,828,630)	7,463,338,324,130 (6,795,808,528,880–8,172,594,461,680)	7,107,716,045,550 (6,424,948,747,940–7,814,513,528,060)	6,288,548,215,540 (5,035,431,353,960–7,778,686,973,800)
Russia	Group I	451,013,803,547 (416,980,163,841–490,979,968,538)	525,315,344,930 (482,140,840,249–571,364,589,075)	512,206,913,176 (467,816,240,754–556,525,297,481)	438,010,078,344 (372,838,000,320–510,052,937,241)
Russia	NCD	4,344,728,951,930 (3,883,185,301,050–4,790,595,491,330)	5,456,839,215,350 (4,946,548,676,910–6,000,848,627,690)	5,328,124,926,630 (4,802,138,190,910–5,854,531,649,880)	4,893,852,085,080 (3,921,897,833,580–6,045,868,421,730)
Russia	Injuries	1,087,751,598,510 (963,450,015,168–1,217,698,478,480)	1,481,183,763,850 (1,314,712,328,870–1,658,026,832,570)	1,267,384,205,740 (1,120,671,582,070–1,425,274,695,680)	956,686,052,119 (719,946,903,574–1,256,316,852,000)
Ukraine	All causes	2,071,863,515,010 (1,893,577,103,480–2,269,014,851,860)	2,336,832,177,350 (2,145,568,488,120–2,540,660,933,170)	2,297,618,681,880 (2,115,127,785,720–2,488,467,658,550)	1,922,056,135,920 (1,615,617,369,150–2,290,086,603,370)
Ukraine	Group I	132,186,376,783 (115,944,373,619–151,290,533,405)	147,260,955,390 (134,949,951,206–163,348,820,114)	157,839,403,653 (145,886,032,195–171,397,387,198)	114,424,278,825 (98,282,739,032–132,438,801,109)
Ukraine	NCD	1,636,748,171,320 (1,487,812,854,790–1,806,584,321,630)	1,840,472,668,330 (1,683,988,898,180–2,010,616,374,430)	1,832,176,581,500 (1,683,529,068,230–1,996,179,801,610)	1,586,746,199,200 (1,332,338,414,920–1,894,623,901,230)
Ukraine	Injuries	302,928,966,912 (271,417,999,959–338,402,714,185)	349,098,553,623 (314,174,156,479–385,670,355,376)	307,602,696,725 (277,417,865,888–341,534,222,963)	220,885,657,899 (171,623,773,309–274,907,365,082)
Central Europe	All causes	4,366,647,663,270 (3,990,254,156,960–4,799,528,186,040)	4,104,981,876,010 (3,725,214,140,380–4,526,279,227,290)	3,999,530,270,640 (3,619,081,232,540–4,438,575,142,360)	3,805,030,758,430 (3,410,919,497,190–4,247,427,282,540)
Central Europe	Group I	385,368,360,332 (357,706,242,994–420,340,758,571)	242,177,146,559 (218,093,885,941–270,151,736,576)	200,379,767,284 (178,155,398,385–227,583,625,992)	158,522,243,133 (139,224,829,948–182,158,813,064)
Central Europe	NCD	3,421,137,304,110 (3,114,864,429,200–3,756,965,545,170)	3,376,767,849,790 (3,061,368,737,270–3,714,855,705,860)	3,345,922,874,520 (3,030,344,108,330–3,700,533,230,840)	3,236,996,562,830 (2,911,203,133,180–3,600,864,834,930)
Central Europe	Injuries	560,141,998,827 (498,710,531,410–634,146,341,553)	486,036,879,659 (428,078,933,840–559,901,999,538)	453,227,628,837 (393,529,220,513–527,827,556,204)	409,511,952,475 (343,159,798,676–492,707,863,565)
Albania	All causes	91,724,955,089 (82,079,881,825–101,735,667,011)	79,778,686,469 (71,374,837,172–89,125,426,131)	77,700,879,916 (69,013,256,790–87,376,199,368)	76,884,739,246 (66,640,827,119–87,437,390,161)
Albania	Group I	28,364,001,917 (24,200,393,036–33,175,229,244)	12,292,985,977 (10,475,250,339–14,216,198,619)	7,276,779,221 (6,130,372,538–8,767,587,867)	5,176,094,567 (3,981,474,813–6,707,888,576)
Albania	NCD	53,017,291,095 (45,998,284,412–60,034,066,480)	56,859,649,986 (50,155,137,990–64,262,732,088)	62,499,236,477 (55,454,870,902–70,353,870,232)	64,375,239,591 (56,049,277,346–73,107,565,125)
Albania	Injuries	10,343,662,077 (9,216,666,962–11,711,452,050)	10,626,050,506 (8,980,978,773–12,205,081,788)	7,924,864,218 (6,765,683,298–9,412,155,508)	7,333,405,088 (5,976,014,504–9,042,058,149)
Bosnia and Herzegovina	All causes	129,083,904,624 (113,611,743,031–144,708,088,890)	109,355,840,096 (95,932,552,317–123,812,463,529)	115,231,417,589 (101,383,033,774–130,252,123,162)	119,519,189,214 (103,668,290,470–137,238,289,904)
Bosnia and Herzegovina	Group I	14,946,780,044 (13,293,360,327–16,865,194,626)	6,644,427,617 (5,712,669,816–7,766,809,361)	5,694,449,353 (4,831,362,683–6,812,007,705)	4,485,714,983 (3,692,424,301–5,427,573,934)
Bosnia and Herzegovina	NCD	99,957,576,206 (87,392,374,743–112,961,870,939)	92,538,124,540 (81,665,231,850–104,207,498,960)	99,514,438,059 (87,980,427,179–111,690,438,213)	104,527,789,506 (91,105,326,490–119,143,606,117)
Bosnia and Herzegovina	Injuries	14,179,548,374 (11,897,535,322–16,579,459,865)	10,173,287,938 (8,298,912,268–12,517,953,481)	10,022,530,176 (8,026,198,564–12,479,484,372)	10,505,684,725 (8,214,643,252–13,492,027,438)
Bulgaria	All causes	334,075,996,141 (305,608,455,075–365,760,816,508)	323,334,720,628 (296,150,005,252–353,961,887,216)	310,316,411,168 (283,431,983,811–340,147,250,462)	283,031,571,701 (246,365,284,586–321,175,126,601)
Bulgaria	Group I	23,878,366,014 (21,936,108,038–26,218,377,296)	16,766,035,045 (15,203,044,425–18,656,531,682)	13,625,485,731 (12,225,511,807–15,289,290,335)	10,309,534,892 (8,693,632,608–12,302,680,382)
Bulgaria	NCD	272,251,659,808 (249,196,031,554–298,886,222,122)	274,761,797,569 (252,617,210,782–299,532,359,766)	268,391,071,687 (245,914,634,152–292,646,909,074)	248,520,717,368 (216,323,755,867–281,020,451,567)
Bulgaria	Injuries	37,945,970,319 (33,166,398,311–43,473,845,961)	31,806,888,014 (27,697,263,569–36,718,824,792)	28,299,853,751 (24,135,313,199–33,260,536,427)	24,201,319,440 (19,313,149,121–29,904,763,765)
Croatia	All causes	168,386,674,362 (151,624,192,620–186,561,272,988)	156,099,728,025 (140,510,829,511–172,800,070,606)	150,857,208,424 (134,809,866,936–167,843,219,665)	143,217,218,217 (125,851,096,913–161,599,879,806)
Croatia	Group I	8,680,511,137 (7,965,797,493–9,502,224,273)	6,508,710,705 (5,839,296,807–7,244,090,115)	5,279,386,101 (4,678,329,668–5,985,947,304)	3,747,109,697 (3,241,558,806–4,363,776,677)
Croatia	NCD	136,898,103,016 (122,889,668,776–151,479,304,863)	132,942,909,677 (119,447,107,264–147,178,814,706)	129,906,830,757 (116,104,296,633–144,378,531,012)	125,013,525,072 (110,101,649,573–140,636,889,730)
Croatia	Injuries	22,808,060,209 (20,179,862,770–25,991,204,258)	16,648,107,643 (14,514,558,511–19,077,840,770)	15,670,991,566 (13,529,813,734–18,347,051,679)	14,456,583,449 (11,981,224,256–17,620,736,504)
Czech Republic	All causes	375,995,059,939 (343,846,855,881–414,628,852,220)	329,401,267,439 (295,679,971,359–368,255,919,661)	325,619,628,910 (289,441,125,185–365,376,805,107)	319,281,854,644 (279,881,367,195–364,590,043,135)
Czech Republic	Group I	18,167,182,563 (16,364,123,999–20,301,370,630)	12,507,734,030 (10,916,755,059–14,563,743,274)	12,518,097,674 (10,945,559,111–14,615,175,818)	11,762,001,631 (10,053,419,139–13,851,128,512)
Czech Republic	NCD	309,800,639,435 (283,334,559,179–340,437,559,559)	275,634,429,321 (247,980,357,861–306,343,679,932)	271,784,242,005 (242,757,158,864–303,179,905,398)	269,046,197,982 (236,654,496,047–304,403,602,063)
Czech Republic	Injuries	48,027,237,940 (41,865,195,071–55,358,440,751)	41,259,104,087 (35,483,614,055–48,491,222,626)	41,317,289,230 (34,676,951,524–49,444,858,983)	38,473,655,030 (31,173,345,593–47,662,842,622)
Hungary	All causes	446,061,338,665 (407,769,779,234–487,927,248,456)	406,997,088,955 (370,777,467,340–448,060,736,574)	386,810,260,160 (349,588,742,702–427,612,663,479)	361,345,139,725 (316,606,166,374–407,112,980,505)
Hungary	Group I	23,546,259,451 (21,390,894,584–26,218,579,243)	15,191,661,749 (13,275,614,222–17,563,437,789)	12,352,648,874 (10,541,088,729–14,543,523,533)	10,754,000,175 (8,832,566,480–12,912,174,150)
Hungary	NCD	362,040,061,792 (330,242,898,819–394,368,109,292)	344,530,348,599 (314,471,841,836–378,593,008,966)	332,554,256,270 (301,880,470,677–366,816,460,033)	314,164,215,463 (277,711,914,210–351,337,205,080)
Hungary	Injuries	60,475,017,422 (53,118,376,935–68,657,945,976)	47,275,078,608 (41,296,779,813–54,706,106,445)	41,903,355,016 (36,214,945,808–48,731,086,691)	36,426,924,086 (30,012,188,487–44,212,325,248)
Macedonia	All causes	62,626,618,307 (56,835,517,588–68,907,614,698)	60,765,952,339 (54,854,391,817–67,442,685,050)	62,599,652,156 (56,214,113,532–69,268,021,806)	65,106,202,122 (58,405,048,228–72,667,171,232)
Macedonia	Group I	10,262,121,932 (9,402,401,929–11,226,699,437)	4,730,769,126 (4,181,790,500–5,340,183,856)	3,721,559,917 (3,269,303,549–4,277,406,086)	3,070,406,326 (2,429,339,346–3,851,436,810)
Macedonia	NCD	46,362,639,950 (41,609,611,969–51,377,287,445)	50,549,924,760 (45,453,351,918–55,993,250,497)	53,674,959,935 (48,382,547,304–59,364,475,178)	56,641,002,622 (51,174,278,507–63,006,760,778)
Macedonia	Injuries	6,001,856,424 (5,206,290,291–6,941,763,942)	5,485,258,454 (4,754,148,673–6,437,329,182)	5,203,132,304 (4,404,902,806–6,260,690,821)	5,394,793,174 (4,409,738,359–6,613,115,685)
Montenegro	All causes	16,248,290,463 (14,380,007,461–18,370,019,690)	18,868,779,962 (17,014,650,991–20,984,492,842)	18,410,485,362 (16,435,966,177–20,577,444,016)	18,382,866,507 (16,120,308,219–20,774,167,781)
Montenegro	Group I	1,537,916,303 (1,242,914,762–1,873,133,467)	1,453,872,652 (1,310,248,213–1,633,214,761)	1,015,496,336 (895,208,279–1,157,619,633)	720,756,055 (592,318,878–879,343,221)
Montenegro	NCD	12,664,084,894 (11,194,789,997–14,305,915,548)	15,272,674,843 (13,733,400,001–17,021,489,945)	15,367,855,089 (13,719,357,747–17,163,618,545)	15,651,777,955 (13,833,665,786–17,681,820,729)
Montenegro	Injuries	2,046,289,267 (1,770,199,357–2,384,918,991)	2,142,232,467 (1,871,740,087–2,474,892,209)	2,027,133,937 (1,744,631,932–2,390,725,222)	2,010,332,497 (1,674,659,680–2,424,274,366)
Poland	All causes	1,318,095,503,200 (1,200,848,744,750–1,447,901,830,740)	1,211,440,050,360 (1,091,648,274,730–1,345,597,429,150)	1,214,898,094,830 (1,090,216,407,820–1,355,497,450,320)	1,184,762,208,230 (1,036,303,056,260–1,339,798,269,440)
Poland	Group I	104,621,157,358 (95,922,754,411–115,281,513,256)	59,668,493,345 (53,132,506,859–67,731,462,547)	54,883,945,108 (48,498,748,446–62,801,239,783)	47,301,721,925 (40,445,402,750–55,827,980,536)
Poland	NCD	1,039,311,444,870 (946,405,510,418–1,143,331,930,340)	990,718,324,827 (892,925,472,303–1,096,992,054,490)	1,001,853,621,020 (900,278,009,637–1,116,532,428,620)	993,511,426,170 (871,407,898,422–1,119,787,712,340)
Poland	Injuries	174,162,900,981 (155,380,097,025–196,382,485,367)	161,053,232,185 (141,885,991,809–186,201,412,917)	158,160,528,708 (137,819,458,232–184,678,088,008)	143,949,060,137 (119,796,728,280–173,136,388,200)
Romania	All causes	879,868,292,738 (803,229,356,552–966,400,908,500)	836,885,616,605 (759,794,331,942–917,714,811,586)	783,387,303,374 (709,800,318,610–865,187,059,734)	707,059,466,370 (625,825,416,081–790,496,851,525)
Romania	Group I	109,291,636,429 (101,603,235,936–118,920,797,831)	76,269,710,845 (69,843,514,219–84,451,917,595)	58,636,772,260 (52,749,608,672–65,288,244,257)	40,165,541,984 (35,043,589,041–46,600,208,348)
Romania	NCD	655,611,617,271 (594,762,322,555–720,815,160,412)	666,643,461,501 (604,060,305,308–733,105,599,043)	641,134,949,417 (582,071,111,272–708,061,111,813)	596,760,257,315 (530,424,277,741–662,850,211,760)
Romania	Injuries	114,965,039,037 (101,316,291,153–130,649,899,993)	93,972,444,260 (81,646,879,177–108,047,788,559)	83,615,581,698 (72,267,003,336–97,715,719,364)	70,133,667,071 (58,240,439,871–84,269,486,849)
Serbia	All causes	301,836,803,111 (270,193,340,075–336,840,400,584)	340,778,729,013 (309,174,870,349–375,869,739,147)	323,469,819,985 (291,655,423,368–357,011,674,028)	300,379,250,330 (268,737,486,328–335,427,862,086)
Serbia	Group I	25,082,603,510 (20,996,704,195–30,158,051,893)	18,246,899,129 (15,799,381,125–21,132,032,252)	14,446,151,268 (12,474,733,583–16,768,355,961)	11,455,744,444 (9,831,561,143–13,576,010,335)
Serbia	NCD	242,554,096,488 (215,779,742,251–270,557,123,711)	287,214,826,557 (260,356,125,360–315,090,197,976)	278,740,885,107 (252,522,501,642–307,399,266,019)	259,605,715,050 (232,946,996,095–288,134,857,300)
Serbia	Injuries	34,200,103,112 (30,071,306,809–39,294,623,292)	35,317,003,328 (30,277,005,103–40,819,595,510)	30,282,783,610 (25,817,679,333–36,226,712,025)	29,317,790,835 (24,631,901,975–35,486,586,460)

Appendix Table 3. GBD 2016 DALY counts for all causes and level 1 cause groups by location for 1990, 2000, 2006 & 2016, both sexes combined.

	Cause	1990	2000	2006	2016
Slovakia	All causes	178,943,833,666 (163,174,739,973–197,315,966,712)	169,071,580,064 (151,315,591,309–187,818,305,231)	169,455,252,599 (152,084,096,262–189,393,297,722)	166,631,348,131 (144,707,455,340–190,215,136,938)
Slovakia	Group I	13,802,047,800 (12,330,181,976–15,218,398,429)	9,320,883,303 (8,253,657,870–10,606,582,856)	8,670,224,190 (7,629,980,773–9,947,242,712)	7,601,808,565 (6,400,753,944–8,910,142,531)
Slovakia	NCD	140,991,060,541 (128,501,124,509–155,697,658,117)	138,859,739,822 (124,405,495,930–154,186,637,563)	141,194,406,310 (126,830,067,344–157,265,210,572)	140,030,465,211 (121,993,865,130–158,885,218,333)
Slovakia	Injuries	24,150,725,325 (21,340,545,956–27,522,580,770)	20,890,956,939 (18,204,055,967–24,143,736,331)	19,590,622,099 (16,878,929,528–22,860,501,946)	18,999,074,355 (15,673,905,097–23,052,119,503)
Slovenia	All causes	63,700,392,958 (56,603,438,812–71,414,070,369)	62,203,836,055 (54,833,813,423–70,104,531,041)	60,773,856,166 (52,963,370,026–69,261,739,878)	59,429,703,994 (50,415,614,694–69,198,252,631)
Slovenia	Group I	3,187,775,872 (2,824,326,710–3,643,870,522)	2,574,963,036 (2,230,570,962–2,990,688,525)	2,258,771,250 (1,933,810,038–2,668,789,271)	1,971,807,887 (1,655,736,678–2,365,955,017)
Slovenia	NCD	49,677,028,747 (44,062,348,733–55,541,918,235)	50,241,637,788 (44,149,507,097–56,432,033,748)	49,306,122,391 (43,131,276,134–55,678,687,930)	49,148,233,519 (42,039,796,961–56,533,976,341)
Slovenia	Injuries	10,835,588,340 (9,501,920,589–12,491,266,916)	9,387,235,231 (8,054,276,581–11,035,061,431)	9,208,962,524 (7,724,526,377–11,011,492,577)	8,309,662,587 (6,719,436,596–10,292,379,060)
Central Asia	All causes	2,743,206,634,350 (2,547,637,726,220–2,954,532,958,110)	2,742,682,902,210 (2,533,067,305,700–2,964,041,038,780)	2,813,437,190,410 (2,600,112,424,060–3,068,732,444,550)	2,698,220,549,550 (2,425,625,541,760–2,977,256,320,570)
Central Asia	Group I	1,033,330,806,230 (972,251,284,244–1,102,465,484,340)	765,356,904,523 (711,283,110,945–828,919,730,384)	662,400,330,391 (598,774,226,104–743,103,039,614)	519,049,908,913 (438,876,383,145–616,444,309,075)
Central Asia	NCD	1,371,324,353,750 (1,241,001,867,990–1,518,376,201,990)	1,641,683,444,070 (1,493,043,281,370–1,801,813,463,470)	1,801,889,361,720 (1,637,759,235,580–1,975,534,580,560)	1,860,646,172,860 (1,654,547,503,260–2,070,422,865,150)
Central Asia	Injuries	338,551,474,380 (314,817,742,551–365,856,683,151)	335,642,553,615 (310,597,043,078–363,559,003,554)	349,147,498,295 (320,588,839,672–379,260,795,296)	318,524,467,784 (284,780,614,002–356,876,555,165)
Armenia	All causes	118,283,423,290 (107,291,377,712–129,817,218,873)	94,100,696,156 (85,231,116,065–103,873,949,069)	93,839,262,781 (84,708,593,560–103,833,811,189)	88,024,772,274 (77,923,870,804–99,064,132,010)
Armenia	Group I	29,685,774,644 (26,206,697,132–33,988,681,204)	11,512,844,961 (10,004,913,422–13,117,578,402)	9,083,941,024 (7,657,027,585–10,698,195,449)	6,298,432,289 (5,202,194,035–7,598,675,495)
Armenia	NCD	72,359,748,872 (64,947,473,178–80,552,342,320)	72,804,354,423 (65,353,029,637–80,681,192,302)	75,881,321,118 (68,606,586,762–84,065,917,016)	73,731,284,321 (65,167,351,271–83,020,693,004)
Armenia	Injuries	16,237,899,773 (14,417,035,723–18,506,136,866)	9,783,496,772 (8,452,420,003–11,553,315,903)	8,874,000,640 (7,628,047,974–10,551,330,793)	7,995,055,664 (6,636,820,702–9,899,906,270)
Azerbaijan	All causes	299,064,645,216 (269,841,673,762–328,293,293,085)	279,723,236,788 (254,964,868,540–307,327,390,207)	299,092,386,390 (268,451,862,685–332,490,825,652)	302,291,855,079 (259,448,379,295–347,100,398,494)
Azerbaijan	Group I	125,221,476,427 (106,931,809,638–145,206,939,560)	83,288,997,435 (73,445,122,794–94,830,406,375)	77,890,948,907 (65,246,696,730–93,118,986,414)	54,966,878,780 (40,407,207,437–72,397,532,841)
Azerbaijan	NCD	144,145,949,086 (128,610,026,945–159,946,617,817)	171,546,535,656 (153,851,912,001–190,590,255,565)	196,690,574,582 (175,184,524,151–218,854,421,205)	220,485,080,886 (189,722,060,533–253,826,315,703)
Azerbaijan	Injuries	29,697,219,703 (26,452,874,027–33,261,542,508)	24,887,703,697 (22,273,446,586–28,076,840,466)	24,510,862,901 (21,397,186,904–28,092,792,437)	26,839,895,413 (22,214,438,547–32,194,360,805)
Georgia	All causes	200,944,448,664 (183,798,022,578–220,781,982,775)	157,653,214,031 (141,817,734,428–174,096,255,463)	156,004,061,218 (140,940,581,415–172,317,827,208)	139,222,248,545 (120,724,286,104–159,080,840,775)
Georgia	Group I	39,412,646,467 (35,335,224,883–43,981,237,478)	22,850,988,280 (20,187,150,574–25,887,797,232)	18,125,522,381 (15,469,500,729–21,300,390,451)	12,432,172,405 (10,240,281,032–15,166,098,208)
Georgia	NCD	138,938,535,758 (124,829,941,190–154,190,404,633)	121,994,749,498 (109,198,756,297–134,740,535,624)	124,511,627,942 (112,431,075,343–137,697,442,884)	113,010,844,477 (98,129,195,131–129,734,372,163)
Georgia	Injuries	22,593,266,439 (20,276,759,732–25,375,485,384)	12,807,476,253 (11,165,234,531–14,733,906,420)	13,366,910,895 (11,659,239,822–15,404,141,535)	13,779,231,663 (11,368,181,677–16,571,424,880)
Kazakhstan	All causes	608,832,793,370 (551,181,650,154–671,250,386,746)	647,573,144,408 (586,960,353,913–710,973,114,185)	681,534,439,996 (616,056,823,273–750,936,137,511)	557,083,610,448 (482,353,739,687–642,721,584,601)
Kazakhstan	Group I	134,964,425,082 (120,029,176,288–150,201,277,211)	108,247,885,126 (95,770,848,275–122,243,063,873)	99,971,549,004 (86,496,813,492–114,657,617,810)	65,302,663,605 (53,885,793,574–78,996,110,601)
Kazakhstan	NCD	373,803,156,199 (334,558,046,828–418,184,194,819)	435,329,632,144 (392,245,088,522–479,245,387,671)	461,010,436,636 (415,887,583,861–507,605,480,930)	405,156,932,513 (349,020,980,947–468,879,251,539)
Kazakhstan	Injuries	100,065,212,090 (90,173,422,594–111,188,273,571)	103,995,627,138 (93,110,517,522–116,145,871,613)	120,552,454,356 (108,233,870,657–133,850,913,160)	86,624,014,330 (72,936,261,906–102,897,893,196)
Kyrgyzstan	All causes	189,045,730,985 (175,800,689,205–202,738,861,366)	177,807,162,281 (164,401,304,513–192,234,995,919)	182,684,607,018 (168,797,409,722–198,425,405,256)	175,308,951,249 (155,828,232,655–194,236,883,740)
Kyrgyzstan	Group I	74,553,748,401 (69,008,655,836–80,600,667,243)	51,364,181,040 (47,513,755,020–55,483,011,739)	45,830,291,180 (41,898,954,634–50,238,668,638)	43,259,557,886 (36,847,701,677–50,699,310,961)
Kyrgyzstan	NCD	89,320,970,876 (80,772,486,793–98,462,937,878)	104,104,477,055 (94,060,195,893–114,490,869,893)	115,123,923,947 (104,512,976,178–126,267,214,892)	111,418,686,962 (97,673,682,938–125,943,718,658)
Kyrgyzstan	Injuries	25,171,011,708 (23,089,078,405–27,251,899,386)	22,338,504,186 (20,462,520,781–24,166,385,407)	21,730,391,891 (20,006,704,762–23,691,857,550)	20,630,706,401 (18,273,852,037–23,291,111,202)
Mongolia	All causes	111,441,956,130 (103,033,620,803–120,797,752,565)	93,261,105,695 (86,225,055,828–101,491,727,012)	95,283,335,790 (87,170,066,142–103,729,617,966)	102,253,396,373 (90,621,924,952–116,405,338,845)
Mongolia	Group I	53,251,971,577 (47,884,665,370–59,568,331,875)	27,110,710,468 (24,338,813,414–30,107,936,341)	20,163,929,150 (17,888,217,348–22,623,056,364)	16,725,327,231 (13,769,270,666–20,105,205,496)
Mongolia	NCD	45,657,207,736 (40,677,389,370–51,109,185,721)	52,569,038,581 (47,440,817,629–58,168,133,200)	59,669,246,912 (53,688,730,628–66,065,085,529)	69,127,744,274 (60,605,629,334–78,881,082,554)
Mongolia	Injuries	12,532,776,817 (11,134,315,877–14,082,069,387)	13,581,356,646 (11,985,820,815–14,961,331,558)	15,450,159,728 (13,453,955,524–17,217,045,883)	16,400,324,868 (13,872,406,368–19,151,360,150)
Tajikistan	All causes	280,367,207,184 (253,232,246,079–308,346,448,076)	243,613,262,030 (219,480,335,957–271,510,852,193)	221,270,286,163 (199,425,054,780–247,141,248,865)	246,159,404,634 (206,507,482,421–292,971,823,457)
Tajikistan	Group I	165,695,832,088 (145,226,089,889–187,837,273,293)	117,989,631,009 (101,437,247,175–137,837,854,823)	88,151,195,786 (76,446,501,961–102,586,799,356)	90,145,022,672 (63,570,970,750–124,187,110,378)
Tajikistan	NCD	89,541,583,613 (79,058,520,757–100,177,457,116)	99,875,953,569 (88,615,990,068–112,191,342,183)	110,059,817,267 (96,635,090,495–124,559,596,065)	129,629,447,584 (111,427,647,332–149,243,689,084)
Tajikistan	Injuries	25,129,791,483 (22,248,426,951–29,134,463,478)	25,747,677,451 (22,941,309,804–28,760,419,869)	23,059,273,110 (20,566,698,796–26,153,283,981)	26,384,934,378 (22,320,200,982–30,866,186,876)
Turkmenistan	All causes	183,978,369,032 (165,626,452,925–202,628,548,187)	190,134,437,313 (168,034,258,043–216,368,633,062)	180,606,613,344 (160,390,873,026–203,917,814,000)	168,757,857,046 (148,704,517,460–190,292,650,497)
Turkmenistan	Group I	92,971,499,894 (79,879,525,240–107,367,844,442)	73,246,075,236 (58,701,011,060–92,375,621,628)	55,113,507,382 (42,228,819,178–70,235,803,173)	37,341,504,313 (27,973,046,776–50,198,088,664)
Turkmenistan	NCD	71,194,938,143 (64,716,959,723–78,537,523,948)	95,011,356,476 (86,635,715,501–104,123,675,407)	104,419,074,908 (94,908,324,892–114,980,363,517)	114,764,004,390 (102,096,824,609–128,179,727,300)
Turkmenistan	Injuries	19,811,930,995 (17,952,427,585–21,901,094,059)	21,877,005,602 (19,647,441,891–24,360,819,514)	21,074,031,053 (18,844,955,851–23,575,287,460)	16,652,348,343 (14,427,318,624–19,209,985,641)
Uzbekistan	All causes	751,248,060,481 (678,918,542,587–828,203,367,084)	858,816,643,504 (781,615,821,465–944,417,064,153)	903,122,197,711 (802,991,329,181–1,015,370,214,180)	919,118,453,905 (801,064,967,858–1,055,991,911,880)
Uzbekistan	Group I	317,573,431,648 (274,917,726,538–362,751,880,938)	269,745,590,969 (235,354,342,603–309,973,531,053)	248,069,445,576 (190,537,104,066–313,637,158,287)	192,578,349,731 (135,526,119,135–277,537,481,759)
Uzbekistan	NCD	346,362,263,462 (308,968,416,216–388,663,006,853)	488,447,346,666 (438,614,509,912–538,532,828,370)	554,523,338,413 (495,969,983,409–616,801,638,133)	623,322,147,451 (551,261,916,460–701,193,477,746)
Uzbekistan	Injuries	87,312,365,371 (79,537,391,176–96,780,575,077)	100,623,705,869 (91,227,470,380–110,422,990,022)	100,529,413,721 (89,294,718,701–112,705,825,457)	103,217,956,724 (89,545,589,440–117,624,801,665)
Latin America and Caribbean	All causes	13,700,202,574,300 (12,739,643,525,400–14,786,829,987,000)	13,372,347,612,700 (12,194,327,388,600–14,699,122,543,500)	13,570,885,613,900 (12,280,019,345,000–15,036,789,171,700)	14,588,104,390,100 (13,068,632,741,100–16,313,820,388,200)
Latin America and Caribbean	Group I	4,991,363,686,600 (4,760,319,481,050–5,259,117,481,580)	3,458,878,477,050 (3,282,798,457,260–3,681,149,315,410)	2,905,468,146,160 (2,737,162,638,220–3,124,120,847,540)	2,249,097,513,780 (2,078,647,552,360–2,460,731,826,330)
Latin America and Caribbean	NCD	6,641,107,028,630 (5,832,066,347,870–7,471,051,731,810)	7,811,901,716,840 (6,823,522,966,800–8,886,636,777,580)	8,573,500,013,530 (7,492,643,668,540–9,784,497,801,660)	10,141,981,276,500 (8,853,902,774,100–11,599,023,294,600)
Latin America and Caribbean	Injuries	2,067,731,859,060 (1,959,696,904,210–2,178,648,848,610)	2,101,567,418,780 (1,975,930,477,040–2,222,790,096,900)	2,091,917,454,220 (1,970,345,890,900–2,218,666,421,610)	2,197,025,599,780 (2,052,401,930,630–2,363,301,603,000)
Central Latin America	All causes	5,285,301,343,430 (4,918,825,966,170–5,712,610,358,340)	5,251,914,637,620 (4,799,553,955,200–5,777,262,076,080)	5,341,442,384,860 (4,828,684,533,690–5,929,308,401,880)	5,904,288,117,700 (5,264,290,137,600–6,608,560,522,960)
Central Latin America	Group I	1,870,641,261,730 (1,800,358,621,340–1,952,498,176,740)	1,290,226,889,680 (1,231,368,441,160–1,370,337,739,410)	1,047,975,469,880 (989,352,258,378–1,118,642,412,410)	806,726,506,555 (743,380,606,463–876,384,427,952)
Central Latin America	NCD	2,521,836,356,670 (2,216,384,978,540–2,863,740,595,990)	3,052,194,613,590 (2,669,401,755,710–3,491,430,368,240)	3,403,586,757,810 (2,967,838,108,590–3,906,924,778,500)	4,170,315,666,610 (3,625,420,612,490–4,784,247,223,530)
Central Latin America	Injuries	892,823,725,023 (841,791,079,471–938,944,778,305)	909,493,134,352 (853,035,355,524–960,931,026,142)	889,880,157,176 (833,262,999,330–946,205,074,402)	927,245,944,536 (853,565,669,753–1,009,656,758,890)
Colombia	All causes	1,000,871,884,210 (915,195,137,883–1,098,531,958,240)	1,064,652,713,960 (968,402,570,500–1,170,443,851,810)	1,032,386,248,640 (926,890,668,755–1,149,113,977,190)	1,028,139,803,870 (896,468,280,268–1,174,464,028,670)
Colombia	Group I	261,817,408,272 (231,827,756,879–293,807,568,212)	208,324,013,304 (189,127,229,022–230,545,396,919)	179,713,249,635 (161,153,904,264–199,997,871,121)	124,828,159,312 (109,164,036,770–143,161,529,756)
Colombia	NCD	490,043,448,807 (426,601,160,582–560,843,669,488)	582,451,549,295 (503,406,490,307–669,819,840,696)	640,333,710,362 (554,446,295,119–739,789,766,830)	720,850,173,728 (610,589,856,454–847,393,350,916)
Colombia	Injuries	249,011,027,133 (227,074,134,100–264,762,656,799)	273,877,151,356 (249,887,519,086–291,072,559,593)	212,339,288,647 (196,876,583,659–226,617,014,623)	182,461,470,828 (162,602,670,846–203,692,780,918)
Costa Rica	All causes	63,618,032,523 (56,468,015,649–71,540,861,400)	76,392,543,739 (67,216,301,952–86,793,658,485)	82,136,801,254 (71,552,925,029–94,426,215,244)	95,060,133,454 (81,635,325,320–110,262,687,823)
Costa Rica	Group I	12,628,257,888 (11,033,836,829–14,581,129,030)	10,628,425,446 (9,099,601,188–12,541,167,638)	9,577,121,736 (8,177,512,180–11,314,630,627)	8,083,600,855 (6,557,990,742–9,951,429,941)
Costa Rica	NCD	43,080,742,950 (37,053,101,383–49,883,751,414)	55,428,090,175 (47,530,294,410–64,309,118,080)	61,778,239,029 (52,743,004,271–72,359,511,188)	75,238,283,928 (63,828,901,340–87,908,088,069)
Costa Rica	Injuries	7,909,031,685 (7,364,286,404–8,533,208,330)	10,336,028,117 (9,639,677,971–11,201,025,820)	10,781,440,489 (9,935,151,218–11,729,161,477)	11,738,248,672 (10,388,990,307–13,227,217,007)
El Salvador	All causes	201,985,407,998 (186,405,902,654–218,387,614,260)	170,987,724,008 (155,987,439,230–188,034,935,028)	164,233,467,665 (148,843,855,497–182,033,176,098)	159,610,230,086 (141,294,368,585–181,190,244,467)
El Salvador	Group I	67,497,786,973 (60,103,454,548–76,089,288,037)	39,064,414,983 (34,563,261,629–44,207,985,407)	26,327,567,610 (22,857,096,779–30,702,403,608)	16,814,211,052 (14,003,153,466–20,199,835,425)
El Salvador	NCD	90,660,047,909 (79,091,155,647–102,404,576,211)	93,966,572,856 (82,621,399,896–107,519,776,034)	99,086,636,383 (86,520,255,595–113,237,937,004)	107,997,479,980 (92,994,437,541–124,804,638,360)
El Salvador	Injuries	43,827,573,116 (40,944,835,559–47,035,801,208)	37,956,736,170 (35,205,598,166–40,618,265,547)	38,819,263,672 (34,955,067,338–41,525,689,417)	34,798,539,054 (30,371,675,931–39,569,198,072)
Guatemala	All causes	466,752,360,124 (433,129,769,371–502,008,735,949)	442,621,003,926 (401,805,017,951–487,519,854,457)	447,573,758,579 (402,092,302,230–497,034,124,127)	452,818,730,409 (385,351,424,278–525,865,006,613)
Guatemala	Group I	270,822,148,018 (249,561,573,871–291,657,133,069)	210,205,628,380 (192,418,283,904–228,254,594,559)	172,816,531,146 (158,127,719,953–189,285,168,461)	119,557,419,927 (102,798,586,263–137,403,274,983)
Guatemala	NCD	145,632,207,990 (127,803,367,518–163,949,469,180)	174,798,174,349 (150,410,425,953–203,095,903,672)	203,027,100,911 (172,797,022,931–233,580,409,472)	254,944,911,920 (212,647,542,903–299,473,610,167)

Appendix Table 3. GBD 2016 DALY counts for all causes and level 1 cause groups by location for 1990, 2000, 2006 & 2016, both sexes combined.

	Cause	1990	2000	2006	2016
Guatemala	Injuries	50,298,004,116 (43,385,635,910–59,974,660,175)	57,617,201,196 (49,873,399,162–67,485,025,862)	71,730,126,522 (60,905,350,871–83,309,357,681)	78,316,398,561 (60,724,365,230–98,597,425,909)
Honduras	All causes	190,500,702,635 (172,967,276,727–207,980,313,844)	182,438,887,959 (159,308,151,262–206,664,844,410)	187,456,303,753 (160,995,512,516–215,289,303,848)	209,289,602,998 (177,663,596,404–246,235,293,329)
Honduras	Group I	80,753,628,100 (72,244,308,031–89,432,392,808)	56,337,907,072 (49,301,135,236–63,451,021,997)	46,730,658,980 (41,192,856,030–53,087,346,893)	39,571,517,574 (33,540,008,270–46,293,121,838)
Honduras	NCD	85,521,310,180 (74,940,653,158–95,690,212,539)	100,254,195,561 (84,834,994,883–116,031,715,284)	112,949,024,153 (94,368,129,740–132,309,362,884)	138,114,538,402 (114,467,853,201–164,078,102,134)
Honduras	Injuries	24,225,764,356 (19,534,493,639–28,799,299,877)	25,846,785,325 (19,881,541,543–32,420,069,113)	27,776,620,619 (21,217,303,844–35,532,120,215)	31,603,547,023 (23,385,982,441–42,295,727,041)
Mexico	All causes	2,668,090,773,440 (2,484,610,765,730–2,874,189,054,220)	2,540,630,355,140 (2,309,718,477,860–2,804,383,359,220)	2,581,723,638,800 (2,330,114,875,640–2,871,229,062,460)	2,966,833,311,070 (2,656,658,085,860–3,340,871,125,710)
Mexico	Group I	954,137,603,160 (907,094,160,263–992,401,735,914)	586,996,717,932 (559,049,194,744–623,524,197,689)	457,865,004,102 (429,364,306,866–491,635,990,750)	356,182,439,976 (328,416,424,692–390,611,446,174)
Mexico	NCD	1,303,753,318,220 (1,146,914,066,540–1,475,336,174,260)	1,591,700,838,670 (1,396,487,575,570–1,815,982,622,540)	1,769,452,667,020 (1,548,772,888,000–2,023,941,729,190)	2,220,483,821,470 (1,947,890,817,960–2,548,772,939,490)
Mexico	Injuries	410,199,852,050 (387,553,597,097–433,081,981,311)	361,932,798,532 (340,875,833,026–387,732,406,850)	354,405,967,674 (334,452,223,682–382,268,704,453)	390,167,049,623 (363,731,911,316–421,487,325,286)
Nicaragua	All causes	133,329,873,698 (123,519,839,465–143,473,808,246)	125,442,983,124 (113,903,271,005–137,298,900,415)	118,215,542,475 (105,732,891,663–131,217,614,434)	121,971,645,464 (105,406,942,559–141,601,178,604)
Nicaragua	Group I	65,092,819,476 (60,947,883,123–69,121,528,501)	42,788,043,064 (39,558,981,374–46,039,910,050)	29,951,398,049 (27,269,226,298–33,008,150,298)	17,920,067,415 (15,636,904,197–20,575,329,897)
Nicaragua	NCD	51,634,595,912 (44,274,564,217–59,499,272,605)	65,905,452,335 (56,575,483,654–75,876,540,714)	72,638,581,182 (61,943,049,268–84,300,280,472)	88,255,614,674 (74,560,125,619–103,936,741,619)
Nicaragua	Injuries	16,602,458,309 (15,164,797,810–18,220,943,639)	16,749,487,724 (15,487,562,388–18,170,089,394)	15,625,563,244 (14,453,862,676–17,029,521,844)	15,795,963,376 (13,387,041,246–18,700,498,880)
Panama	All causes	57,875,967,727 (52,040,620,524–64,453,722,538)	67,228,282,820 (59,831,535,590–75,297,616,628)	73,970,040,914 (66,159,485,337–83,122,684,860)	85,970,078,474 (73,822,997,862–99,140,373,040)
Panama	Group I	14,063,780,799 (12,295,482,187–15,907,583,205)	15,487,092,582 (13,807,350,789–17,771,963,258)	15,078,668,569 (13,302,103,869–17,484,219,143)	13,813,206,175 (11,572,391,276–16,625,038,224)
Panama	NCD	35,023,907,996 (30,400,169,795–40,363,162,999)	42,374,897,430 (36,225,212,045–49,270,221,111)	48,514,919,352 (41,860,507,472–56,047,251,456)	60,250,407,097 (51,121,906,559–70,535,972,693)
Panama	Injuries	8,788,278,931 (8,105,884,419–9,559,791,485)	9,366,292,809 (8,519,882,351–10,259,741,893)	10,376,452,993 (9,426,384,669–11,483,401,970)	11,906,465,203 (9,948,943,729–13,906,811,224)
Venezuela	All causes	502,276,341,077 (457,968,637,045–552,845,736,466)	581,520,142,951 (524,188,644,564–646,574,555,633)	653,746,582,782 (589,860,439,496–727,200,117,828)	784,594,581,878 (683,199,034,531–894,884,176,416)
Venezuela	Group I	143,827,829,044 (137,686,952,690–151,117,911,512)	120,394,646,915 (109,998,523,422–131,213,405,789)	109,915,270,054 (102,462,223,970–118,507,400,902)	109,955,884,271 (97,587,650,026–124,062,921,178)
Venezuela	NCD	276,486,776,706 (240,844,140,835–318,558,700,772)	345,314,842,914 (299,037,967,919–397,971,604,748)	395,805,879,413 (343,089,547,571–455,128,986,282)	504,180,435,411 (427,711,890,363–584,281,709,885)
Venezuela	Injuries	81,961,735,328 (75,902,122,025–89,333,982,746)	115,810,653,122 (105,761,736,271–126,052,807,248)	148,025,433,315 (131,877,315,721–163,226,461,809)	170,458,262,197 (140,596,001,258–211,452,242,730)
Andean Latin America	All causes	1,638,757,302,800 (1,532,522,158,090–1,748,588,696,610)	1,372,977,741,250 (1,255,467,168,430–1,499,301,378,260)	1,310,417,122,190 (1,183,098,509,460–1,454,991,353,650)	1,355,274,658,420 (1,195,836,153,700–1,536,012,581,720)
Andean Latin America	Group I	788,677,156,079 (738,432,333,058–840,899,444,143)	484,089,228,241 (455,582,230,673–517,171,173,188)	385,149,779,636 (356,276,963,148–416,828,300,512)	295,382,778,866 (264,239,174,097–327,796,197,724)
Andean Latin America	NCD	635,411,773,360 (557,272,001,796–720,806,126,801)	701,263,283,570 (607,082,829,970–804,028,121,374)	749,152,143,627 (646,285,125,253–866,903,844,561)	876,460,394,656 (746,245,575,911–1,021,693,944,560)
Andean Latin America	Injuries	214,668,373,364 (192,597,382,600–241,296,572,563)	187,625,229,445 (173,271,402,281–202,156,880,110)	176,115,198,928 (163,751,872,032–188,452,443,967)	183,431,484,894 (164,811,523,292–202,348,959,529)
Bolivia	All causes	429,906,409,871 (403,804,729,658–457,624,329,124)	359,157,033,762 (331,425,988,151–390,322,585,786)	332,733,985,337 (299,261,501,075–367,037,126,709)	321,610,109,180 (279,486,027,277–367,910,067,345)
Bolivia	Group I	238,349,124,288 (218,314,233,259–260,685,866,961)	163,068,675,417 (145,715,725,452–183,629,048,998)	131,165,009,351 (115,587,936,393–150,171,489,408)	93,564,515,873 (77,548,715,510–111,958,651,845)
Bolivia	NCD	142,096,113,327 (123,786,635,887–162,075,837,106)	152,919,391,646 (134,408,423,464–173,684,828,854)	162,617,180,092 (141,776,127,105–184,862,386,127)	189,774,185,608 (160,580,793,752–221,574,770,989)
Bolivia	Injuries	49,461,172,256 (41,146,408,704–57,519,390,361)	43,168,966,700 (38,133,001,105–48,863,389,295)	38,951,795,894 (34,361,137,495–43,825,113,273)	38,271,407,699 (31,802,957,946–45,158,610,956)
Ecuador	All causes	344,323,190,068 (315,862,935,973–376,047,472,673)	348,584,462,591 (316,339,289,827–385,458,960,172)	354,134,521,483 (317,492,035,016–395,243,389,669)	379,020,123,411 (336,263,538,705–428,364,543,329)
Ecuador	Group I	139,978,411,976 (127,208,886,228–153,245,931,872)	104,107,820,832 (93,704,838,282–114,891,940,164)	85,020,591,178 (76,057,117,859–94,981,724,487)	64,261,033,809 (56,515,341,714–72,936,359,437)
Ecuador	NCD	156,688,559,478 (136,524,365,668–177,446,480,317)	188,432,151,042 (162,073,598,326–216,837,305,601)	208,565,843,506 (179,942,044,067–240,331,880,057)	247,327,582,152 (211,401,546,454–286,327,722,459)
Ecuador	Injuries	47,656,218,614 (44,525,555,340–52,008,160,839)	56,044,490,717 (51,964,396,018–59,814,463,947)	60,548,086,800 (55,490,170,926–64,913,300,763)	67,431,507,451 (58,847,564,015–76,381,476,038)
Peru	All causes	864,527,702,864 (800,878,247,551–932,541,869,850)	665,236,244,902 (598,235,565,220–738,745,543,323)	623,548,615,370 (552,541,204,041–703,886,562,540)	654,644,425,825 (556,933,203,290–763,475,880,446)
Peru	Group I	410,349,619,815 (375,058,706,017–446,830,565,665)	216,912,731,992 (199,737,684,643–236,259,221,078)	168,964,179,106 (153,854,053,638–186,648,304,391)	137,557,229,184 (118,940,835,694–160,367,873,834)
Peru	NCD	336,627,100,555 (292,748,945,921–385,390,825,555)	359,911,740,882 (307,567,093,516–415,979,695,019)	377,969,120,029 (321,828,198,162–443,914,542,893)	439,358,626,896 (362,893,432,191–524,791,324,294)
Peru	Injuries	117,550,982,494 (99,627,232,115–137,416,188,291)	88,411,772,028 (78,780,058,530–97,662,324,037)	76,615,316,235 (69,303,328,427–84,294,963,637)	77,728,569,745 (66,192,018,494–90,963,911,876)
Caribbean	All causes	1,426,431,883,420 (1,302,979,199,230–1,553,388,136,870)	1,420,969,903,710 (1,285,715,318,590–1,574,157,512,370)	1,455,303,842,070 (1,321,336,115,970–1,610,351,740,770)	1,415,116,192,410 (1,261,203,620,350–1,590,816,391,910)
Caribbean	Group I	538,519,329,636 (476,001,811,334–603,148,601,266)	494,335,150,058 (429,958,616,008–568,654,195,787)	485,748,738,951 (432,779,810,208–545,568,467,670)	333,347,888,557 (280,147,366,882–405,147,951,296)
Caribbean	NCD	723,394,821,400 (644,191,365,664–806,031,722,467)	776,291,033,330 (685,760,552,412–874,529,350,898)	819,560,614,263 (722,480,140,190–926,413,945,560)	923,466,354,452 (806,944,395,486–1,047,338,505,360)
Caribbean	Injuries	164,517,732,386 (147,316,722,656–180,193,271,336)	150,343,720,323 (137,550,169,553–163,530,789,127)	149,994,488,858 (136,788,846,974–164,864,009,002)	158,301,949,403 (141,694,880,924–176,850,531,971)
Antigua and Barbuda	All causes	1,548,055,469 (1,367,621,073–1,737,490,845)	1,887,471,989 (1,650,622,664–2,144,825,482)	1,953,680,162 (1,701,264,643–2,237,181,902)	2,163,693,029 (1,866,916,822–2,492,447,994)
Antigua and Barbuda	Group I	238,646,804 (199,200,063–291,638,392)	365,197,699 (307,408,193–443,859,337)	312,352,571 (269,124,724–368,758,249)	276,732,277 (231,037,445–338,152,951)
Antigua and Barbuda	NCD	1,173,270,598 (1,030,053,662–1,323,448,887)	1,359,033,576 (1,179,058,097–1,556,712,448)	1,448,736,662 (1,248,002,246–1,671,726,481)	1,684,208,928 (1,443,769,749–1,942,277,612)
Antigua and Barbuda	Injuries	136,138,067 (121,520,673–153,359,617)	163,240,714 (143,812,768–186,167,894)	192,590,928 (169,954,931–217,912,204)	202,751,824 (172,689,465–234,057,929)
The Bahamas	All causes	7,352,293,288 (6,471,548,284–8,343,495,039)	8,620,895,614 (7,806,219,932–9,597,497,863)	9,376,639,347 (8,424,826,084–10,494,003,678)	11,736,645,134 (10,317,087,852–13,256,575,070)
The Bahamas	Group I	1,591,912,413 (1,227,168,842–2,118,251,471)	2,159,589,540 (1,968,553,182–2,384,722,918)	1,836,860,355 (1,651,120,866–2,044,733,723)	1,633,148,985 (1,370,632,820–2,084,532,440)
The Bahamas	NCD	4,708,320,355 (4,151,200,738–5,302,092,527)	5,466,419,936 (4,825,021,443–6,222,200,153)	6,385,589,781 (5,608,639,429–7,268,484,314)	8,684,353,496 (7,546,433,476–9,837,714,878)
The Bahamas	Injuries	1,052,060,520 (955,504,562–1,163,890,768)	994,886,138 (902,657,206–1,101,027,014)	1,154,189,210 (1,027,943,169–1,270,059,263)	1,419,142,653 (1,209,541,377–1,637,200,392)
Barbados	All causes	7,318,635,077 (6,528,620,023–8,184,536,407)	7,742,289,877 (6,843,762,315–8,737,343,650)	7,771,517,372 (6,910,418,254–8,748,496,749)	8,514,031,886 (7,486,188,137–9,639,426,055)
Barbados	Group I	1,045,035,408 (837,718,577–1,310,894,677)	1,213,365,719 (1,022,429,369–1,482,094,991)	1,026,809,434 (884,478,179–1,201,488,037)	957,865,606 (777,858,444–1,212,855,727)
Barbados	NCD	5,667,854,223 (5,003,035,735–6,373,126,524)	5,918,518,887 (5,183,046,052–6,721,462,066)	6,085,884,178 (5,358,839,238–6,896,612,655)	6,899,832,107 (6,039,080,832–7,825,700,575)
Barbados	Injuries	605,745,446 (538,804,878–685,992,293)	610,405,271 (541,668,380–694,657,142)	658,823,760 (577,342,312–741,838,180)	656,334,174 (562,997,645–762,039,420)
Belize	All causes	6,183,401,793 (5,537,077,521–6,924,840,400)	7,502,878,682 (6,701,708,062–8,390,824,815)	7,888,588,110 (6,919,904,412–8,958,770,526)	9,628,388,898 (8,190,646,711–11,409,373,584)
Belize	Group I	2,375,375,221 (2,026,442,419–2,777,114,371)	2,267,319,339 (1,909,025,996–2,661,148,144)	2,132,291,380 (1,762,794,179–2,598,647,376)	2,071,663,372 (1,549,165,633–2,863,405,852)
Belize	NCD	2,977,650,983 (2,615,615,867–3,378,242,757)	3,995,481,910 (3,500,817,579–4,527,539,515)	4,463,349,905 (3,864,739,994–5,101,863,883)	6,040,626,493 (5,124,443,261–7,001,820,649)
Belize	Injuries	830,375,588 (735,166,560–927,652,236)	1,240,077,433 (1,084,551,915–1,388,687,244)	1,292,946,825 (1,142,157,366–1,447,535,114)	1,516,099,033 (1,204,409,959–1,868,579,120)
Bermuda	All causes	1,550,679,059 (1,397,620,441–1,726,944,536)	1,526,644,171 (1,358,565,153–1,709,062,230)	1,453,679,684 (1,278,837,285–1,638,364,929)	1,483,125,171 (1,275,646,210–1,716,545,662)
Bermuda	Group I	157,988,263 (137,369,895–183,719,408)	197,574,083 (180,390,344–219,236,094)	161,984,254 (144,247,360–183,453,371)	133,390,040 (113,805,527–157,605,261)
Bermuda	NCD	1,238,362,616 (1,110,680,096–1,382,025,433)	1,220,469,391 (1,076,392,939–1,369,797,590)	1,176,389,219 (1,027,036,023–1,336,181,014)	1,231,038,188 (1,052,120,403–1,426,859,004)
Bermuda	Injuries	154,328,180 (138,437,318–171,056,291)	108,600,697 (92,783,269–125,704,820)	115,306,211 (101,619,456–131,506,459)	118,696,943 (99,883,384–141,714,005)
Cuba	All causes	281,272,496,867 (251,584,160,752–314,190,631,283)	280,632,501,817 (248,604,615,544–316,631,140,512)	280,114,985,096 (247,949,618,379–317,374,452,854)	306,242,334,323 (267,365,705,655–348,361,369,220)
Cuba	Group I	33,631,845,817 (29,887,163,131–38,495,098,764)	27,024,391,980 (23,379,457,007–31,629,582,304)	23,764,864,049 (20,156,511,936–28,156,812,474)	23,645,534,741 (20,174,482,457–27,972,379,202)
Cuba	NCD	205,872,801,246 (180,642,055,918–232,417,149,159)	219,542,916,370 (192,678,629,076–249,594,803,842)	228,005,426,576 (200,380,150,160–260,242,757,153)	254,848,225,294 (221,511,235,376–289,839,414,371)
Cuba	Injuries	41,767,849,804 (38,925,976,277–44,716,287,285)	34,065,193,466 (31,290,553,020–36,946,073,075)	28,344,694,471 (25,709,592,660–31,584,894,535)	27,748,574,288 (24,253,049,860–31,969,790,257)
Dominica	All causes	1,820,542,973 (1,623,428,885–2,033,279,087)	1,827,980,209 (1,628,124,571–2,032,921,982)	1,877,108,424 (1,678,820,474–2,108,482,949)	2,240,761,414 (1,959,442,923–2,552,795,527)
Dominica	Group I	316,006,070 (257,663,542–386,515,638)	327,346,076 (282,627,501–383,100,926)	299,251,480 (252,434,463–357,719,620)	332,151,116 (260,801,312–437,415,047)
Dominica	NCD	1,330,813,645 (1,178,299,742–1,500,847,858)	1,337,361,595 (1,179,331,839–1,511,077,015)	1,400,069,007 (1,239,483,658–1,581,758,315)	1,687,657,989 (1,472,277,012–1,918,479,437)
Dominica	Injuries	173,723,259 (156,673,703–190,784,185)	163,272,538 (146,443,062–182,860,094)	177,787,937 (160,993,180–197,586,666)	220,952,309 (187,649,259–259,473,283)
Dominican Republic	All causes	238,989,808,392 (214,826,065,364–263,771,920,063)	243,970,820,597 (220,717,106,664–271,354,133,267)	264,214,809,543 (236,132,750,049–295,830,929,268)	264,060,381,644 (229,040,853,192–300,121,580,301)
Dominican Republic	Group I	102,094,293,661 (88,294,709,473–116,637,416,982)	91,795,077,042 (80,516,687,265–104,871,401,542)	95,378,074,914 (82,298,491,251–109,498,916,478)	69,580,984,914 (58,522,225,218–82,845,660,016)

Appendix Table 3. GBD 2016 DALY counts for all causes and level 1 cause groups by location for 1990, 2000, 2006 & 2016, both sexes combined.

	Cause	1990	2000	2006	2016
Dominican Republic	NCD	111,947,577,118<br>(97,935,042,593–127,967,003,682)	124,761,360,899<br>(106,518,666,208–144,456,736,013)	138,486,165,995<br>(118,079,780,865–160,836,040,415)	162,814,937,623<br>(136,018,187,944–189,629,913,950)
Dominican Republic	Injuries	24,947,937,613<br>(22,710,582,096–28,281,697,671)	27,414,382,656<br>(24,866,692,482–30,031,400,415)	30,350,568,634<br>(27,219,141,488–34,058,275,649)	31,664,459,107<br>(26,828,463,908–36,998,712,231)
Grenada	All causes	2,913,029,868<br>(2,537,281,615–3,368,976,846)	2,894,157,898<br>(2,588,968,944–3,231,981,689)	3,025,983,725<br>(2,701,033,834–3,416,933,851)	3,203,928,988<br>(2,800,349,011–3,631,768,567)
Grenada	Group I	639,510,080<br>(483,778,011–886,476,025)	503,962,659<br>(418,978,601–604,319,834)	447,331,068<br>(377,225,417–549,324,763)	450,731,672<br>(364,791,962–587,260,235)
Grenada	NCD	1,993,452,517<br>(1,758,284,290–2,248,257,450)	2,073,204,373<br>(1,845,484,494–2,328,173,039)	2,224,228,097<br>(1,972,410,821–2,518,082,091)	2,385,950,670<br>(2,075,429,037–2,719,709,444)
Grenada	Injuries	280,067,271<br>(240,630,906–324,102,259)	316,990,865<br>(272,096,426–359,662,046)	354,424,559<br>(300,917,422–406,379,198)	367,246,645<br>(302,503,102–435,720,168)
Guyana	All causes	31,004,895,348<br>(28,020,373,863–33,973,559,841)	29,030,247,604<br>(26,517,830,973–31,804,436,541)	29,107,596,438<br>(26,639,843,885–31,780,329,069)	27,727,742,987<br>(24,845,716,411–30,874,500,977)
Guyana	Group I	11,885,428,463<br>(10,067,567,086–13,888,077,779)	9,661,408,874<br>(8,578,747,022–10,839,905,655)	8,270,550,583<br>(7,454,170,175–9,139,504,707)	5,688,704,560<br>(4,901,279,909–6,515,936,730)
Guyana	NCD	15,152,943,088<br>(13,554,794,377–16,861,117,329)	15,253,472,901<br>(13,690,484,119–17,029,594,767)	16,378,255,206<br>(14,631,956,016–18,240,671,892)	17,833,902,135<br>(15,723,502,284–20,097,344,832)
Guyana	Injuries	3,966,523,796<br>(3,671,282,878–4,313,399,596)	4,115,365,829<br>(3,777,608,850–4,486,230,464)	4,458,790,649<br>(4,044,269,123–4,841,274,412)	4,205,136,291<br>(3,710,190,997–4,685,093,594)
Haiti	All causes	601,928,145,077<br>(528,970,768,141–675,398,572,664)	571,216,436,021<br>(491,274,135,446–658,446,864,097)	586,558,835,223<br>(517,873,723,488–662,441,124,998)	505,238,117,862<br>(423,025,569,247–607,779,239,622)
Haiti	Group I	331,980,085,431<br>(274,556,263,529–390,793,463,003)	309,680,167,922<br>(247,627,858,952–379,508,927,840)	311,107,583,684<br>(263,325,067,739–366,852,052,129)	195,045,421,822<br>(146,398,011,891–264,625,758,696)
Haiti	NCD	205,639,722,551<br>(183,510,248,785–228,510,930,747)	207,253,308,671<br>(183,896,120,309–232,880,058,961)	221,379,514,442<br>(193,118,178,176–250,557,930,520)	248,902,907,075<br>(209,780,218,723–289,040,235,154)
Haiti	Injuries	64,308,337,095<br>(49,258,045,228–77,143,666,977)	54,282,959,428<br>(44,088,785,225–64,128,811,147)	54,071,737,097<br>(44,111,964,608–65,368,400,842)	61,289,788,965<br>(49,887,051,903–73,244,356,441)
Jamaica	All causes	67,516,135,888<br>(58,655,546,170–78,146,049,438)	73,281,606,502<br>(64,501,133,058–83,653,750,139)	73,045,030,738<br>(63,543,944,715–84,012,936,242)	80,628,607,718<br>(68,513,625,666–93,294,094,841)
Jamaica	Group I	19,493,288,057<br>(15,054,897,628–26,043,566,155)	18,060,234,311<br>(14,530,105,607–22,874,160,446)	15,024,607,876<br>(11,737,330,761–20,338,901,060)	12,720,234,050<br>(9,400,693,940–17,370,139,412)
Jamaica	NCD	43,202,013,417<br>(37,778,477,916–49,257,019,640)	49,314,639,411<br>(43,220,860,989–56,065,945,600)	51,068,538,470<br>(44,380,086,883–58,369,825,386)	59,981,178,030<br>(51,163,040,178–69,370,872,232)
Jamaica	Injuries	4,820,834,415<br>(4,321,328,058–5,441,918,408)	5,906,732,779<br>(5,241,391,087–6,694,796,416)	6,951,884,392<br>(6,023,548,799–7,846,936,142)	7,927,195,638<br>(6,450,520,891–9,490,344,939)
Puerto Rico	All causes	101,506,856,012<br>(91,651,408,804–112,365,540,254)	106,676,875,553<br>(94,964,675,404–119,680,241,073)	102,033,209,208<br>(90,431,176,123–115,441,823,206)	99,506,295,831<br>(87,565,434,836–113,215,534,166)
Puerto Rico	Group I	16,500,882,297<br>(15,411,959,804–17,773,920,290)	13,407,163,815<br>(12,266,455,076–14,705,869,454)	11,053,573,698<br>(10,036,540,886–12,288,305,212)	8,787,738,685<br>(7,805,421,245–10,033,958,526)
Puerto Rico	NCD	71,704,184,929<br>(63,323,129,066–80,997,807,912)	80,572,380,830<br>(70,549,712,612–91,620,759,276)	79,041,158,648<br>(69,076,906,668–90,353,256,844)	79,843,979,759<br>(69,511,043,233–91,256,363,002)
Puerto Rico	Injuries	13,301,788,786<br>(12,073,015,837–14,562,348,061)	12,697,330,908<br>(11,365,506,385–14,055,834,940)	11,938,476,862<br>(10,686,570,844–13,292,353,853)	10,874,577,387<br>(9,472,114,404–12,452,147,136)
Saint Lucia	All causes	4,100,417,521<br>(3,542,356,772–4,742,627,640)	4,063,181,841<br>(3,604,419,779–4,575,173,406)	4,334,074,420<br>(3,847,331,668–4,899,983,080)	4,873,702,878<br>(4,312,790,558–5,554,477,361)
Saint Lucia	Group I	990,654,990<br>(726,351,670–1,357,498,244)	764,909,141<br>(617,492,317–965,089,837)	699,796,898<br>(568,285,668–868,497,673)	626,011,849<br>(492,633,829–794,853,007)
Saint Lucia	NCD	2,665,960,633<br>(2,358,616,118–2,986,406,395)	2,818,452,884<br>(2,466,153,629–3,202,759,627)	3,089,118,209<br>(2,715,093,124–3,513,867,215)	3,663,303,961<br>(3,218,075,433–4,181,902,583)
Saint Lucia	Injuries	443,801,898<br>(397,889,715–500,645,588)	479,819,817<br>(440,295,041–528,298,447)	545,159,313<br>(494,282,028–602,029,053)	584,387,068<br>(520,046,757–656,201,051)
Saint Vincent and the Grenadines	All causes	3,198,428,391<br>(2,770,696,098–3,738,144,738)	3,346,458,693<br>(3,003,792,916–3,740,691,945)	3,267,663,861<br>(2,921,861,420–3,625,369,823)	3,475,663,650<br>(3,083,372,513–3,872,932,296)
Saint Vincent and the Grenadines	Group I	826,356,848<br>(604,524,086–1,115,196,020)	794,259,115<br>(670,378,086–954,193,362)	629,061,901<br>(539,770,743–736,384,629)	521,346,507<br>(440,594,206–635,296,539)
Saint Vincent and the Grenadines	NCD	2,028,773,240<br>(1,801,955,252–2,283,197,994)	2,210,720,168<br>(1,964,920,823–2,490,940,727)	2,261,500,712<br>(1,996,301,566–2,538,092,657)	2,553,700,341<br>(2,256,572,820–2,870,117,789)
Saint Vincent and the Grenadines	Injuries	343,298,303<br>(306,353,205–394,133,683)	341,479,410<br>(307,097,595–380,348,280)	377,101,247<br>(336,485,117–417,780,991)	400,616,803<br>(341,874,332–455,657,916)
Suriname	All causes	14,336,472,061<br>(12,810,737,771–16,054,724,846)	16,539,993,389<br>(14,635,564,358–18,619,912,444)	17,312,927,812<br>(15,227,447,904–19,631,948,493)	17,597,353,655<br>(15,414,179,162–20,246,229,475)
Suriname	Group I	4,996,592,763<br>(4,149,471,136–6,040,664,222)	5,269,463,471<br>(4,237,287,791–6,366,676,589)	4,766,066,107<br>(3,715,988,168–5,861,457,521)	3,506,816,746<br>(2,582,162,504–4,713,318,609)
Suriname	NCD	7,605,003,168<br>(6,723,717,922–8,559,095,007)	9,323,130,242<br>(8,256,798,461–10,495,798,683)	10,324,333,905<br>(9,106,997,101–11,656,933,782)	11,769,321,417<br>(10,349,555,243–13,366,934,744)
Suriname	Injuries	1,734,876,130<br>(1,609,711,956–1,871,955,348)	1,947,399,676<br>(1,805,694,586–2,117,969,950)	2,222,527,799<br>(2,055,236,659–2,411,227,683)	2,321,215,492<br>(2,062,440,387–2,597,903,969)
Trinidad and Tobago	All causes	38,335,109,824<br>(34,658,768,745–42,118,857,574)	41,888,603,349<br>(37,903,089,141–46,106,492,168)	41,746,386,546<br>(37,463,705,533–46,252,412,975)	43,396,946,274<br>(37,920,584,712–49,022,116,910)
Trinidad and Tobago	Group I	7,513,197,444<br>(6,481,926,860–8,654,183,661)	8,397,279,257<br>(7,398,280,876–9,601,128,208)	6,344,118,417<br>(5,402,633,396–7,479,935,936)	4,928,502,026<br>(3,735,256,192–7,081,682,064)
Trinidad and Tobago	NCD	26,173,833,178<br>(23,369,132,290–29,116,885,034)	29,129,464,630<br>(26,001,888,268–32,464,764,280)	29,898,747,990<br>(26,371,160,737–33,583,259,478)	33,273,984,900<br>(29,029,988,856–37,807,020,042)
Trinidad and Tobago	Injuries	4,648,079,202<br>(4,300,751,335–5,063,161,559)	4,361,859,461<br>(3,999,645,211–4,738,256,242)	5,503,520,139<br>(4,862,184,559–6,024,208,413)	5,194,459,348<br>(4,522,484,480–5,850,147,038)
Virgin Islands, U.S.	All causes	2,848,591,139<br>(2,546,177,459–3,166,000,588)	3,202,511,923<br>(2,875,852,684–3,579,829,404)	3,418,067,829<br>(3,077,812,193–3,817,230,963)	3,759,693,058<br>(3,300,446,079–4,257,558,429)
Virgin Islands, U.S.	Group I	438,969,149<br>(392,711,875–501,019,567)	355,794,276<br>(292,064,331–470,986,523)	322,192,080<br>(255,251,510–442,363,696)	313,384,521<br>(234,776,847–459,002,151)
Virgin Islands, U.S.	NCD	2,057,814,450<br>(1,811,475,822–2,326,158,494)	2,493,657,459<br>(2,201,003,497–2,814,090,714)	2,718,075,061<br>(2,417,912,041–3,061,522,725)	3,101,042,788<br>(2,695,831,405–3,519,576,669)
Virgin Islands, U.S.	Injuries	351,807,540<br>(305,327,234–400,958,479)	353,060,187<br>(303,469,602–398,205,360)	377,800,689<br>(312,991,429–428,189,187)	345,265,749<br>(267,116,989–412,924,122)
Tropical Latin America	All causes	5,313,535,675,690<br>(4,916,015,878,490–5,758,488,465,800)	5,289,808,443,960<br>(4,816,461,860,420–5,839,249,979,270)	5,424,947,075,370<br>(4,889,860,764,270–6,043,293,892,060)	5,875,362,994,510<br>(5,231,786,327,180–6,566,876,453,440)
Tropical Latin America	Group I	1,779,238,156,030<br>(1,650,961,880,840–1,918,342,239,400)	1,176,413,709,220<br>(1,096,348,587,210–1,269,886,590,720)	972,226,948,353<br>(895,970,974,680–1,064,922,593,060)	804,378,983,831<br>(730,095,457,317–895,565,987,185)
Tropical Latin America	NCD	2,743,619,541,190<br>(2,402,750,151,200–3,073,997,082,680)	3,263,746,866,790<br>(2,851,197,460,140–3,699,025,517,020)	3,581,166,060,720<br>(3,116,214,200,870–4,073,477,202,460)	4,147,366,903,230<br>(3,621,332,138,640–4,733,222,469,890)
Tropical Latin America	Injuries	790,677,978,469<br>(746,006,769,342–843,192,666,869)	849,647,867,947<br>(794,628,918,887–903,384,381,427)	871,554,066,289<br>(817,174,909,633–928,089,149,616)	923,617,107,447<br>(851,819,266,104–994,691,599,321)
Brazil	All causes	5,202,209,075,840<br>(4,811,920,049,070–5,637,674,740,490)	5,155,843,993,360<br>(4,697,168,671,840–5,692,482,057,010)	5,276,934,340,550<br>(4,758,463,894,560–5,874,985,368,670)	5,709,099,056,890<br>(5,080,557,631,620–6,383,117,164,360)
Brazil	Group I	1,738,384,979,780<br>(1,611,925,008,760–1,875,324,699,260)	1,136,758,484,190<br>(1,057,126,488,960–1,229,341,657,000)	936,482,785,873<br>(863,193,933,626–1,026,233,049,540)	776,908,176,911<br>(706,157,848,428–866,157,010,698)
Brazil	NCD	2,684,580,978,230<br>(2,351,327,106,370–3,006,840,640,980)	3,186,125,677,000<br>(2,784,202,876,970–3,609,428,014,110)	3,488,405,969,540<br>(3,032,661,587,830–3,966,035,698,820)	4,032,115,999,070<br>(3,519,071,916,030–4,604,756,402,410)
Brazil	Injuries	779,243,117,826<br>(735,175,195,938–831,684,496,753)	832,959,832,171<br>(778,125,383,023–885,606,281,460)	852,045,585,141<br>(797,971,987,568–908,405,097,336)	900,074,880,912<br>(830,351,771,098–970,887,397,326)
Paraguay	All causes	111,326,599,853<br>(100,521,161,121–123,337,354,208)	133,964,450,595<br>(119,405,683,709–149,764,331,105)	148,012,734,813<br>(131,971,635,432–167,005,325,347)	166,263,937,622<br>(144,548,203,954–189,025,426,133)
Paraguay	Group I	40,853,176,248<br>(36,775,888,723–44,858,070,938)	39,655,225,029<br>(35,278,554,676–44,492,096,949)	35,744,162,480<br>(30,991,935,793–41,073,026,470)	27,470,806,920<br>(22,750,999,471–32,883,764,147)
Paraguay	NCD	59,038,562,961<br>(50,612,055,563–67,783,932,543)	77,621,189,790<br>(66,719,473,117–89,343,616,801)	92,760,091,184<br>(80,583,585,868–106,915,803,821)	115,250,904,166<br>(99,112,533,472–132,773,529,031)
Paraguay	Injuries	11,434,860,644<br>(10,591,532,136–12,440,915,939)	16,688,035,776<br>(15,352,069,231–18,046,006,690)	19,508,481,148<br>(18,098,369,794–21,095,680,997)	23,542,226,536<br>(20,844,835,452–26,665,860,074)
Southeast Asia, East Asia, and Oceania	All causes	60,076,130,779,400<br>(56,153,640,044,100–64,497,198,882,900)	58,392,857,140,100<br>(53,948,531,433,700–63,394,107,930,900)	55,535,686,695,000<br>(50,718,662,578,100–60,993,818,323,900)	55,063,586,179,700<br>(49,706,319,772,500–61,085,048,827,900)
Southeast Asia, East Asia, and Oceania	Group I	19,340,328,842,100<br>(18,405,641,209,500–20,500,374,476,700)	12,950,804,379,300<br>(12,215,035,611,800–13,838,778,960,400)	9,524,745,877,380<br>(8,954,958,131,660–10,230,439,449,300)	6,525,566,666,180<br>(5,986,622,057,650–7,152,213,121,310)
Southeast Asia, East Asia, and Oceania	NCD	32,704,237,181,700<br>(29,448,612,863,000–36,101,226,097,500)	37,808,138,504,800<br>(34,016,538,243,700–41,949,253,914,900)	39,060,726,576,200<br>(34,949,571,027,300–43,634,451,239,500)	42,605,867,819,900<br>(38,065,808,452,700–47,816,857,779,400)
Southeast Asia, East Asia, and Oceania	Injuries	8,031,564,755,560<br>(7,632,483,468,100–8,437,303,267,140)	7,633,914,256,000<br>(7,261,773,607,210–8,067,535,417,550)	6,950,214,241,400<br>(6,515,578,257,720–7,425,169,196,350)	5,932,151,693,610<br>(5,461,256,915,280–6,511,899,526,190)
East Asia	All causes	40,847,194,362,500<br>(38,015,015,546,800–43,948,140,255,100)	39,058,263,591,900<br>(35,942,796,429,700–42,480,783,254,600)	36,427,078,865,300<br>(33,085,187,212,700–40,218,172,403,600)	36,267,000,971,000<br>(32,568,320,654,800–40,499,635,630,600)
East Asia	Group I	10,055,426,134,800<br>(9,445,803,860,420–10,706,393,367,300)	5,704,884,233,020<br>(5,200,319,969,990–6,293,938,528,080)	3,469,029,466,540<br>(3,180,062,049,240–3,819,369,403,270)	2,329,613,132,940<br>(2,049,532,442,940–2,671,211,606,240)
East Asia	NCD	24,670,925,129,100<br>(22,341,927,651,200–27,128,722,811,100)	27,803,924,821,100<br>(25,111,298,795,500–30,750,757,548,700)	28,020,968,015,300<br>(25,109,433,295,100–31,301,063,246,700)	29,896,301,533,800<br>(26,677,108,070,000–33,581,219,235,700)
East Asia	Injuries	6,120,843,098,600<br>(5,827,033,501,540–6,428,523,647,180)	5,549,454,537,770<br>(5,288,038,328,860–5,876,824,858,310)	4,937,081,383,430<br>(4,605,265,109,800–5,266,864,937,060)	4,041,086,304,230<br>(3,686,651,854,870–4,456,719,807,330)
China	All causes	39,789,919,101,500<br>(37,055,139,210,200–42,811,254,650,300)	37,245,262,675,900<br>(34,267,384,625,400–40,569,649,226,800)	35,027,866,721,800<br>(31,735,347,354,300–38,653,864,254,200)	34,765,539,404,100<br>(31,219,585,271,100–38,784,735,001,000)
China	Group I	9,855,117,167,540<br>(9,255,075,938,750–10,492,382,313,400)	4,978,542,043,180<br>(4,669,653,332,900–5,389,929,415,590)	3,255,473,654,700<br>(2,984,043,381,180–3,585,358,839,560)	2,156,911,936,050<br>(1,888,307,701,080–2,478,377,336,760)
China	NCD	23,957,738,097,600<br>(21,705,764,899,900–26,319,798,291,100)	26,882,647,652,600<br>(24,274,490,635,600–29,742,873,470,100)	27,004,683,196,300<br>(24,173,477,989,300–30,165,689,341,000)	28,734,823,413,900<br>(25,623,187,421,000–32,272,730,905,500)
China	Injuries	5,977,063,836,430<br>(5,691,708,505,000–6,278,778,486,960)	5,384,072,980,110<br>(5,129,832,884,640–5,697,445,207,620)	4,767,709,870,830<br>(4,455,376,377,330–5,090,139,816,350)	3,873,804,054,210<br>(3,527,118,267,410–4,273,008,083,360)
North Korea	All causes	586,395,024,179<br>(508,773,715,841–678,798,938,378)	1,297,344,121,810<br>(889,786,946,866–1,747,923,677,530)	866,596,434,828<br>(769,577,083,139–981,275,213,274)	910,614,209,367<br>(812,085,253,125–1,019,582,103,700)
North Korea	Group I	153,263,047,726<br>(113,672,024,793–203,905,551,355)	690,338,487,911<br>(296,427,177,390–1,121,239,392,400)	180,801,977,882<br>(138,235,819,402–236,146,470,123)	140,010,210,801<br>(107,448,153,188–188,849,601,483)
North Korea	NCD	360,259,081,602<br>(316,021,691,763–410,580,138,811)	506,804,454,590<br>(451,892,613,251–568,580,321,777)	576,438,303,811<br>(517,009,943,297–642,802,610,490)	660,581,787,677<br>(591,639,004,174–733,395,178,117)
North Korea	Injuries	72,872,894,851<br>(64,412,293,516–82,912,539,655)	100,201,179,304<br>(87,407,992,191–114,365,013,084)	109,356,153,134<br>(95,703,455,374–124,997,657,069)	110,022,210,889<br>(95,675,131,551–126,850,631,017)
Taiwan, Province of China	All causes	470,880,236,797<br>(419,491,880,903–529,245,888,343)	515,656,794,182<br>(457,421,673,389–579,798,400,715)	532,615,708,598<br>(474,864,050,281–599,296,102,596)	590,847,357,480<br>(512,137,536,292–675,643,028,783)

Appendix Table 3. GBD 2016 DALY counts for all causes and level 1 cause groups by location for 1990, 2000, 2006 & 2016, both sexes combined.

	Cause	1990	2000	2006	2016
Taiwan, Province of China	Group I	47,045,919,529 (37,438,533,892–59,596,788,590)	36,003,701,928 (30,644,599,361–42,819,042,234)	32,753,833,956 (27,522,404,755–38,526,063,777)	32,690,986,083 (25,105,400,222–39,220,079,026)
Taiwan, Province of China	NCD	352,927,949,943 (312,462,043,251–398,326,918,996)	414,472,713,900 (363,497,295,622–471,242,757,469)	439,846,515,173 (387,948,473,147–499,469,208,433)	500,896,332,260 (432,889,982,061–574,704,124,643)
Taiwan, Province of China	Injuries	70,906,367,325 (64,805,493,026–76,217,210,210)	65,180,378,355 (59,604,704,433–71,141,131,453)	60,015,359,469 (54,982,903,275–65,443,083,342)	57,260,039,137 (49,709,557,012–65,758,252,592)
Southeast Asia	All causes	18,866,546,351,200 (17,702,887,947,200–20,162,726,557,800)	18,915,589,078,000 (17,540,562,153,600–20,509,530,665,700)	18,642,795,876,600 (17,140,274,676,900–20,270,273,648,800)	18,300,049,432,200 (16,566,707,714,200–20,233,384,629,200)
Southeast Asia	Group I	9,111,898,477,030 (8,580,392,616,830–9,717,832,720,320)	7,060,640,459,200 (6,635,426,013,970–7,556,197,969,990)	5,869,228,580,030 (5,545,253,026,290–6,255,847,729,840)	4,042,933,884,460 (3,744,954,793,020–4,360,405,080,690)
Southeast Asia	NCD	7,878,132,432,890 (6,962,861,052,380–8,849,230,401,360)	9,809,456,604,840 (8,728,149,845,100–10,998,223,719,800)	10,805,980,562,100 (9,607,493,216,290–12,092,194,509,200)	12,418,764,185,300 (10,989,200,130,100–13,935,912,589,900)
Southeast Asia	Injuries	1,876,515,441,320 (1,727,779,823,040–2,009,602,866,470)	2,045,492,013,960 (1,910,981,166,080–2,182,219,328,200)	1,967,586,734,420 (1,835,522,867,660–2,116,429,875,040)	1,838,351,362,440 (1,686,586,873,650–2,007,123,569,310)
Cambodia	All causes	569,583,703,512 (527,081,023,896–612,196,021,258)	643,705,694,549 (599,668,911,967–694,260,489,438)	582,183,494,853 (539,713,281,542–631,010,987,231)	515,185,717,169 (465,444,399,110–570,653,026,423)
Cambodia	Group I	367,131,294,192 (332,006,466,343–403,258,010,285)	366,574,166,898 (335,412,367,029–398,149,132,669)	280,243,607,966 (255,214,159,994–307,210,626,583)	173,012,192,576 (150,512,407,563–198,118,573,280)
Cambodia	NCD	153,007,707,179 (132,309,886,036–173,207,277,367)	212,758,356,991 (188,489,549,805–238,780,887,266)	239,068,554,540 (211,249,038,338–267,782,328,708)	281,520,289,324 (248,076,929,439–317,625,207,118)
Cambodia	Injuries	49,444,702,141 (41,516,912,621–56,827,988,337)	64,373,170,660 (57,664,759,619–71,782,458,057)	62,871,332,347 (56,986,341,502–69,353,816,759)	60,653,235,269 (53,536,628,669–67,630,784,623)
Indonesia	All causes	8,057,000,053,510 (7,591,404,896,730–8,565,031,745,600)	7,637,147,250,910 (7,105,037,222,440–8,229,346,689,380)	7,502,743,914,170 (6,923,246,162,240–8,165,392,717,000)	7,273,299,021,800 (6,583,175,097,730–8,022,276,033,170)
Indonesia	Group I	4,383,149,451,350 (4,137,562,495,370–4,653,951,718,460)	3,207,921,336,080 (3,021,153,001,610–3,406,364,104,390)	2,657,934,440,860 (2,520,656,758,630–2,827,007,804,310)	1,814,350,625,520 (1,682,937,776,670–1,959,032,792,970)
Indonesia	NCD	3,074,492,945,910 (2,699,565,624,900–3,459,803,473,290)	3,811,022,300,260 (3,391,509,119,630–4,256,729,879,170)	4,230,721,843,380 (3,763,943,755,850–4,722,192,385,020)	4,878,251,425,700 (4,335,158,904,600–5,491,579,268,490)
Indonesia	Injuries	599,357,656,252 (527,761,406,782–664,550,722,980)	618,203,614,565 (564,749,839,691–676,414,747,551)	614,087,629,932 (565,012,004,540–670,348,869,855)	580,696,970,587 (526,923,439,086–645,545,614,328)
Laos	All causes	401,624,116,246 (342,754,788,184–464,144,512,001)	387,105,086,034 (337,346,867,476–438,860,996,617)	341,687,537,250 (292,186,182,939–398,741,993,008)	292,416,905,050 (225,445,974,198–388,261,749,603)
Laos	Group I	279,074,405,761 (223,545,895,952–340,120,735,532)	239,810,829,202 (193,208,635,583–291,893,573,683)	188,541,898,358 (145,570,383,486–238,095,400,369)	127,152,218,483 (78,194,242,284–203,723,379,384)
Laos	NCD	97,336,748,224 (78,807,459,019–119,228,360,315)	118,186,359,215 (97,443,765,883–142,472,364,667)	123,790,978,464 (103,769,849,328–146,431,444,833)	137,086,092,947 (116,247,650,196–161,022,419,541)
Laos	Injuries	25,212,962,261 (18,777,183,080–30,916,970,845)	29,107,897,617 (22,940,733,919–34,885,450,950)	29,354,660,428 (23,888,719,070–34,763,214,436)	28,178,593,620 (22,996,402,612–35,095,756,337)
Malaysia	All causes	430,888,974,944 (389,967,602,292–476,832,563,103)	503,129,748,403 (450,451,426,812–560,014,551,854)	558,578,808,137 (496,866,830,925–631,318,613,402)	673,751,631,846 (594,041,776,923–760,889,786,301)
Malaysia	Group I	109,044,386,315 (98,594,429,226–122,101,717,916)	99,430,018,144 (88,774,952,261–111,714,407,024)	96,163,114,590 (82,282,959,742–110,633,170,883)	98,318,126,851 (80,163,368,736–115,531,368,544)
Malaysia	NCD	267,515,542,899 (234,408,712,956–302,665,544,358)	340,171,785,946 (298,331,080,728–386,340,701,092)	395,345,580,557 (343,429,294,644–452,296,158,563)	499,848,725,596 (434,154,015,767–570,525,973,133)
Malaysia	Injuries	54,329,045,731 (50,470,951,128–58,476,097,570)	63,527,944,313 (59,185,118,330–68,424,262,550)	67,070,112,990 (62,361,117,668–72,793,390,830)	75,584,779,399 (67,873,674,763–84,029,507,169)
Maldives	All causes	9,201,240,950 (8,381,241,235–10,080,706,903)	6,315,816,418 (5,648,917,344–7,090,955,842)	5,810,748,717 (5,044,882,748–6,671,521,264)	6,086,234,028 (5,089,623,587–7,250,117,266)
Maldives	Group I	3,648,198,110 (2,958,303,568–4,567,932,410)	1,608,394,126 (1,398,609,540–1,864,184,124)	1,214,114,762 (1,051,481,893–1,402,207,385)	860,074,204 (702,415,283–1,061,221,833)
Maldives	NCD	4,551,447,456 (3,759,041,700–5,304,596,778)	4,107,740,284 (3,593,666,644–4,683,328,643)	4,110,064,453 (3,498,215,396–4,786,094,504)	4,761,557,422 (3,938,769,633–5,679,864,583)
Maldives	Injuries	1,001,595,384 (750,590,412–1,166,273,000)	599,682,009 (542,289,352–666,607,656)	486,569,502 (435,910,934–546,646,677)	464,602,401 (384,256,178–557,991,836)
Mauritius	All causes	31,022,408,707 (28,227,793,674–34,207,749,837)	33,809,937,730 (30,579,687,638–37,401,042,352)	35,082,574,925 (31,660,712,413–39,037,515,397)	38,123,149,434 (33,119,790,595–43,534,541,771)
Mauritius	Group I	6,301,957,692 (5,728,194,963–7,039,508,867)	4,804,140,401 (4,229,377,791–5,511,278,906)	4,161,754,774 (3,603,133,741–4,891,181,467)	3,800,712,711 (3,227,927,127–4,520,414,485)
Mauritius	NCD	21,843,875,616 (19,652,275,758–24,281,284,982)	25,979,485,079 (23,297,903,460–28,788,081,150)	27,964,375,008 (25,143,976,655–31,124,025,716)	31,483,277,743 (27,362,747,739–35,876,313,627)
Mauritius	Injuries	2,876,575,400 (2,659,825,465–3,097,060,907)	3,026,312,250 (2,805,380,616–3,290,308,494)	2,956,445,143 (2,701,997,120–3,243,328,792)	2,839,158,981 (2,428,914,695–3,321,870,309)
Myanmar	All causes	2,402,386,239,140 (2,122,629,541,370–2,745,219,120,230)	2,372,053,404,190 (2,062,035,093,140–2,713,672,200,070)	2,139,479,488,710 (1,868,463,174,280–2,471,524,013,060)	1,701,542,521,240 (1,500,921,679,540–1,930,329,760,740)
Myanmar	Group I	1,371,895,360,320 (1,136,083,278,560–1,661,625,189,640)	1,139,299,714,500 (922,699,724,232–1,406,314,820,030)	887,624,643,280 (723,641,808,872–1,099,496,931,940)	434,285,532,813 (353,551,866,194–555,495,109,408)
Myanmar	NCD	860,817,343,412 (762,219,161,098–958,109,356,152)	1,029,952,609,800 (909,633,015,660–1,170,959,440,950)	1,058,478,015,790 (928,465,630,974–1,209,313,026,610)	1,100,622,067,540 (967,976,815,774–1,247,004,624,390)
Myanmar	Injuries	169,673,535,402 (148,728,811,920–193,349,976,760)	202,801,079,894 (179,626,968,800–232,887,164,201)	193,376,829,638 (170,122,716,069–220,953,607,008)	166,634,920,894 (146,606,987,074–193,578,889,052)
Philippines	All causes	2,346,762,545,800 (2,174,509,923,640–2,532,838,147,170)	2,511,206,502,150 (2,312,864,175,590–2,725,286,071,070)	2,762,068,549,170 (2,536,483,760,060–3,008,522,717,900)	3,006,824,948,590 (2,677,511,765,730–3,356,838,337,740)
Philippines	Group I	1,177,402,916,730 (1,097,602,696,090–1,261,194,255,650)	953,740,615,171 (888,819,775,935–1,025,919,416,390)	905,880,018,273 (838,508,940,785–979,393,731,771)	776,854,320,473 (694,214,477,398–861,499,139,242)
Philippines	NCD	945,322,559,728 (833,173,452,295–1,067,067,420,660)	1,297,635,710,270 (1,150,495,428,340–1,462,329,787,560)	1,564,831,310,950 (1,393,398,348,540–1,747,234,720,920)	1,934,553,194,490 (1,683,495,671,500–2,185,930,729,290)
Philippines	Injuries	224,037,069,347 (202,832,250,939–248,129,558,717)	259,830,176,708 (233,247,493,923–282,906,927,094)	291,357,219,949 (261,597,727,667–319,170,272,587)	295,417,433,623 (255,484,663,004–341,753,145,361)
Sri Lanka	All causes	522,217,481,171 (473,725,377,615–573,506,956,187)	537,020,649,523 (481,246,600,391–594,447,902,806)	523,676,933,019 (469,911,914,731–582,844,527,962)	500,051,231,759 (423,058,061,802–585,830,733,416)
Sri Lanka	Group I	140,048,966,525 (128,460,617,847–152,244,841,130)	87,596,898,441 (79,651,665,574–96,827,028,819)	74,495,144,862 (67,204,892,180–82,882,660,496)	49,081,413,660 (40,834,293,471–58,173,375,784)
Sri Lanka	NCD	271,991,957,196 (235,235,308,805–309,872,835,252)	338,104,475,331 (295,169,989,272–382,492,313,308)	356,825,918,526 (312,534,925,853–403,573,138,473)	382,799,266,759 (321,694,738,290–448,078,506,207)
Sri Lanka	Injuries	110,176,557,450 (99,270,788,148–122,234,664,995)	111,319,275,752 (98,385,799,892–123,946,082,837)	92,355,869,632 (81,884,631,863–103,189,484,890)	68,170,551,340 (55,196,994,892–84,084,304,082)
Seychelles	All causes	2,135,504,387 (1,967,663,404–2,325,961,231)	2,350,455,775 (2,137,141,608–2,574,972,489)	2,504,676,710 (2,261,567,590–2,776,743,314)	2,725,718,621 (2,414,175,508–3,080,390,826)
Seychelles	Group I	461,719,441 (418,504,097–503,918,156)	357,179,336 (320,124,000–408,463,200)	386,534,002 (351,365,611–432,598,060)	391,201,362 (348,539,295–441,548,711)
Seychelles	NCD	1,408,947,266 (1,270,785,345–1,562,577,861)	1,688,525,805 (1,517,820,984–1,875,332,482)	1,813,123,027 (1,614,082,805–2,038,612,450)	2,049,199,145 (1,803,706,877–2,333,029,562)
Seychelles	Injuries	264,837,681 (245,105,813–283,825,393)	304,750,634 (275,813,017–332,652,953)	305,019,681 (272,934,286–339,920,861)	285,318,115 (244,412,333–330,007,906)
Thailand	All causes	1,723,908,447,810 (1,558,554,131,080–1,899,177,034,760)	1,971,919,840,690 (1,792,008,400,560–2,170,400,656,490)	1,834,088,197,250 (1,652,151,680,710–2,035,218,723,970)	1,834,144,130,690 (1,609,684,527,160–2,072,829,427,830)
Thailand	Group I	432,937,273,926 (371,658,055,402–500,838,984,855)	408,685,648,611 (359,814,272,353–454,974,698,098)	304,704,824,776 (267,873,542,044–347,974,682,086)	229,520,485,841 (183,451,016,706–276,044,049,636)
Thailand	NCD	958,491,672,186 (838,584,079,058–1,092,295,411,440)	1,188,014,495,300 (1,042,268,627,440–1,349,107,363,340)	1,232,660,649,610 (1,071,038,508,860–1,405,577,688,970)	1,358,329,934,510 (1,172,965,232,450–1,556,390,583,700)
Thailand	Injuries	332,479,501,698 (305,474,392,798–355,877,683,911)	375,219,696,783 (341,683,806,018–409,917,054,330)	296,722,722,930 (272,934,712,708–321,587,043,056)	246,293,710,335 (215,748,330,100–279,149,620,575)
Timor-Leste	All causes	53,383,559,504 (45,146,474,423–62,275,396,969)	35,868,294,951 (30,989,199,300–41,397,258,555)	35,029,210,090 (29,669,154,929–40,695,024,500)	32,238,505,573 (26,306,167,627–39,799,563,132)
Timor-Leste	Group I	36,837,064,013 (29,679,665,442–45,027,132,384)	20,955,684,188 (16,647,535,922–26,218,094,162)	18,090,534,848 (13,885,778,038–22,652,451,517)	12,713,807,819 (8,738,150,033–18,503,138,888)
Timor-Leste	NCD	11,957,808,802 (9,487,242,278–14,472,816,703)	11,564,947,145 (9,381,933,363–13,749,734,339)	13,670,081,389 (11,363,032,824–16,010,759,461)	16,623,227,171 (13,691,869,483–19,569,335,175)
Timor-Leste	Injuries	4,588,686,689 (3,553,516,749–5,639,882,799)	3,347,663,618 (2,623,520,233–4,043,799,484)	3,268,593,853 (2,629,337,088–3,959,052,564)	2,901,470,583 (2,139,594,191–3,651,068,096)
Vietnam	All causes	2,310,604,923,860 (2,069,723,035,800–2,545,105,129,010)	2,266,674,060,060 (2,021,754,205,540–2,520,305,766,640)	2,311,750,190,130 (2,031,993,014,400–2,574,903,432,830)	2,414,077,224,800 (2,129,446,091,970–2,744,358,143,320)
Vietnam	Group I	802,892,005,627 (693,105,431,368–932,256,960,627)	528,698,460,317 (470,956,697,263–595,908,353,028)	448,644,828,114 (393,332,675,123–505,766,767,881)	321,550,787,769 (274,991,807,825–371,891,784,683)
Vietnam	NCD	1,204,903,796,900 (1,044,202,167,670–1,364,256,025,790)	1,424,501,342,780 (1,250,731,544,520–1,608,537,678,680)	1,550,142,264,200 (1,349,003,786,790–1,748,024,006,030)	1,782,837,806,220 (1,562,986,753,300–2,033,636,783,890)
Vietnam	Injuries	302,809,121,334 (261,709,136,411–339,820,001,485)	313,474,256,963 (268,016,687,904–357,843,241,893)	312,963,097,821 (265,218,628,547–358,694,957,103)	309,688,630,814 (258,877,505,806–359,893,560,122)
Oceania	All causes	341,997,835,252 (308,849,385,593–379,467,297,412)	399,168,118,501 (348,206,380,614–450,505,719,381)	445,939,372,360 (383,791,961,466–519,323,789,257)	477,544,772,319 (411,350,857,244–560,263,060,510)
Oceania	Group I	162,060,172,720 (139,999,922,969–187,639,067,573)	177,109,554,918 (143,893,922,235–217,468,553,243)	179,544,355,681 (139,180,858,399–234,886,550,814)	148,666,499,209 (113,634,442,059–202,921,339,680)
Oceania	NCD	148,004,993,775 (130,498,978,133–167,198,341,569)	185,507,100,146 (162,896,758,820–211,213,618,455)	223,133,521,539 (196,950,305,833–251,836,545,436)	278,086,473,959 (243,747,335,342–316,021,392,123)
Oceania	Injuries	31,932,668,758 (27,825,361,411–36,459,174,222)	36,551,463,436 (31,619,784,659–42,257,267,645)	43,261,495,140 (37,193,599,112–50,365,624,477)	50,791,799,151 (43,671,246,022–58,830,669,234)
American Samoa	All causes	1,260,316,247 (1,129,131,136–1,409,028,803)	1,432,477,518 (1,270,156,585–1,619,900,231)	1,587,547,891 (1,407,240,742–1,788,313,485)	1,902,197,701 (1,632,096,774–2,206,487,067)
American Samoa	Group I	305,458,942 (270,175,057–342,895,208)	285,957,409 (250,574,373–326,525,952)	263,715,301 (227,199,136–306,007,019)	252,121,810 (205,497,043–318,603,344)
American Samoa	NCD	800,571,721 (703,255,699–906,358,409)	981,789,006 (853,735,347–1,127,057,651)	1,128,436,972 (984,055,276–1,286,405,419)	1,451,278,294 (1,235,655,218–1,684,400,947)
American Samoa	Injuries	154,285,584 (139,591,374–171,052,098)	164,731,103 (147,018,328–184,048,165)	195,395,618 (176,170,392–216,174,294)	198,797,597 (168,276,979–232,362,685)
Federated States of Micronesia	All causes	4,035,917,589 (3,508,479,305–4,676,365,053)	3,804,867,046 (3,333,307,118–4,343,600,839)	3,426,864,857 (2,965,310,766–3,984,092,335)	3,564,728,714 (3,030,780,808–4,205,721,553)
Federated States of Micronesia	Group I	1,584,074,264 (1,263,903,922–1,986,209,301)	1,113,621,334 (926,324,608–1,377,422,798)	668,894,578 (532,116,019–841,161,212)	612,013,453 (462,569,261–860,512,686)
Federated States of Micronesia	NCD	2,033,625,600 (1,764,921,877–2,334,388,228)	2,263,747,481 (1,946,028,388–2,606,078,209)	2,342,321,665 (2,011,966,234–2,730,772,657)	2,523,450,143 (2,064,958,335–2,999,923,153)
Federated States of Micronesia	Injuries	418,217,726 (363,578,520–487,986,720)	427,498,231 (360,199,903–498,906,232)	415,648,614 (346,787,429–492,821,782)	429,265,118 (344,417,325–527,760,838)

Appendix Table 3. GBD 2016 DALY counts for all causes and level 1 cause groups by location for 1990, 2000, 2006 & 2016, both sexes combined.

	Cause	1990	2000	2006	2016
Fiji	All causes	24,249,117,170 (20,527,666,833–28,332,271,937)	29,042,685,062 (25,923,917,755–32,513,471,383)	30,992,619,809 (27,629,805,058–34,578,198,058)	34,617,536,486 (28,852,031,691–40,651,666,350)
Fiji	Group I	6,447,143,916 (5,165,569,127–8,110,100,505)	5,802,144,517 (5,040,709,341–6,683,549,454)	5,630,430,917 (4,818,454,180–6,500,426,025)	4,850,861,911 (3,828,852,536–6,186,068,397)
Fiji	NCD	15,369,832,878 (12,889,071,920–18,088,290,745)	20,230,677,547 (17,872,753,852–23,007,550,622)	22,423,927,421 (19,800,057,963–25,207,509,346)	26,711,147,920 (21,999,255,151–31,675,386,805)
Fiji	Injuries	2,432,140,377 (1,992,857,027–2,979,976,242)	3,009,862,998 (2,662,018,030–3,374,945,527)	2,938,261,470 (2,568,219,767–3,340,627,352)	3,055,526,655 (2,410,874,055–3,758,196,103)
Guam	All causes	3,099,631,120 (2,755,285,921–3,480,195,576)	3,726,304,125 (3,266,095,506–4,208,272,588)	4,236,348,027 (3,769,630,550–4,768,503,196)	5,477,954,000 (4,780,015,653–6,203,023,296)
Guam	Group I	523,654,738 (464,521,031–592,045,815)	510,248,926 (441,720,340–590,562,918)	538,125,001 (472,358,848–632,256,510)	624,757,009 (511,869,796–770,167,432)
Guam	NCD	2,153,138,290 (1,884,401,716–2,446,511,227)	2,786,655,138 (2,418,501,546–3,169,173,969)	3,236,664,002 (2,849,939,992–3,676,224,551)	4,292,881,857 (3,740,747,357–4,889,197,000)
Guam	Injuries	422,838,093 (368,319,757–469,548,607)	429,400,061 (371,888,563–478,998,246)	461,559,024 (404,936,664–520,647,249)	560,315,135 (473,355,998–653,766,060)
Kiribati	All causes	4,703,274,851 (4,048,594,055–5,452,724,679)	4,591,375,479 (4,107,062,107–5,112,078,516)	4,899,893,479 (4,256,925,849–5,600,800,074)	5,197,939,137 (4,452,412,708–6,238,670,523)
Kiribati	Group I	2,415,709,334 (1,896,499,593–3,000,955,571)	1,852,897,964 (1,547,921,659–2,211,381,325)	1,829,917,484 (1,417,608,203–2,322,820,915)	1,510,326,706 (1,080,955,370–2,228,545,868)
Kiribati	NCD	1,973,000,692 (1,727,423,697–2,202,642,696)	2,392,410,281 (2,165,091,950–2,640,350,710)	2,679,773,365 (2,378,830,601–3,007,082,181)	3,241,063,098 (2,844,177,310–3,697,201,446)
Kiribati	Injuries	314,564,825 (263,397,474–362,036,684)	346,067,233 (303,142,209–387,622,903)	390,202,630 (333,255,744–445,279,433)	446,549,333 (371,102,420–533,188,045)
Marshall Islands	All causes	1,719,227,644 (1,519,250,109–1,972,377,445)	2,158,404,673 (1,864,077,039–2,517,984,287)	2,386,257,579 (2,104,753,364–2,714,408,754)	2,702,412,288 (2,332,139,884–3,160,238,096)
Marshall Islands	Group I	639,314,005 (502,175,781–818,139,893)	719,929,927 (527,390,490–980,193,150)	617,050,367 (453,062,252–806,425,318)	528,060,798 (377,134,072–752,203,075)
Marshall Islands	NCD	889,654,508 (788,755,728–1,003,083,575)	1,209,047,434 (1,067,663,757–1,357,301,529)	1,503,165,107 (1,340,304,646–1,679,657,156)	1,884,137,732 (1,651,001,678–2,170,319,853)
Marshall Islands	Injuries	190,259,132 (168,908,066–212,700,585)	229,427,312 (198,448,923–263,084,774)	266,042,105 (230,723,161–301,160,977)	290,213,758 (241,137,978–342,137,960)
Northern Mariana Islands	All causes	912,572,876 (760,888,135–1,085,030,421)	1,264,811,875 (1,072,623,338–1,497,939,929)	1,522,144,728 (1,295,608,014–1,797,787,331)	2,259,054,919 (1,902,121,092–2,668,278,705)
Northern Mariana Islands	Group I	166,850,063 (123,565,497–227,429,122)	193,543,737 (157,566,054–239,042,752)	197,855,398 (158,076,923–259,618,260)	278,894,034 (209,749,697–403,502,789)
Northern Mariana Islands	NCD	604,225,717 (499,521,774–718,054,458)	894,774,791 (740,826,852–1,069,068,714)	1,103,089,114 (929,317,389–1,320,705,465)	1,654,425,129 (1,376,981,279–1,970,335,249)
Northern Mariana Islands	Injuries	141,497,096 (109,464,700–168,512,315)	176,493,347 (144,833,270–209,925,568)	221,200,215 (183,214,556–261,656,747)	325,735,755 (255,703,114–393,366,996)
Papua New Guinea	All causes	267,782,060,629 (239,387,468,785–302,019,197,967)	313,039,144,463 (265,913,038,822–362,690,887,796)	351,518,020,838 (292,577,407,606–424,835,422,259)	371,470,328,331 (308,937,499,206–446,037,695,139)
Papua New Guinea	Group I	136,808,618,092 (115,947,834,418–161,714,632,850)	153,763,051,978 (120,393,753,184–194,634,723,321)	156,062,588,178 (116,116,924,310–211,669,351,816)	128,777,293,177 (94,063,855,573–182,292,995,157)
Papua New Guinea	NCD	105,863,113,445 (91,093,527,405–122,004,741,780)	131,171,780,296 (112,935,870,933–151,124,725,989)	161,209,705,821 (138,870,173,783–185,706,566,117)	202,119,756,610 (173,418,632,701–233,894,131,588)
Papua New Guinea	Injuries	25,110,329,092 (21,261,271,206–29,452,572,520)	28,104,312,190 (23,399,282,119–33,575,411,690)	34,245,726,838 (28,418,099,769–40,984,528,772)	40,573,278,544 (33,631,121,271–48,312,982,385)
Samoa	All causes	4,796,238,273 (4,032,135,634–5,881,168,781)	4,682,170,765 (4,018,532,444–5,490,456,473)	4,769,710,768 (4,125,068,806–5,537,031,242)	5,085,795,206 (4,418,531,227–5,890,117,194)
Samoa	Group I	1,408,406,328 (955,466,661–2,139,123,475)	1,059,230,656 (743,469,626–1,524,055,574)	976,964,396 (681,253,447–1,417,807,917)	838,312,717 (613,128,361–1,186,252,163)
Samoa	NCD	2,858,087,637 (2,487,543,945–3,281,182,837)	3,109,298,243 (2,695,235,111–3,552,480,995)	3,289,899,546 (2,850,782,291–3,756,367,145)	3,733,254,272 (3,203,787,509–4,249,901,383)
Samoa	Injuries	529,744,308 (449,843,707–634,982,291)	513,641,865 (429,756,294–616,648,499)	502,846,826 (417,365,962–600,191,882)	514,228,217 (418,236,528–622,233,100)
Solomon Islands	All causes	14,128,373,339 (12,168,816,872–16,460,490,561)	17,511,125,768 (15,184,231,189–20,092,782,818)	20,793,817,556 (17,822,046,482–23,836,594,055)	22,795,691,939 (19,714,368,633–26,496,833,499)
Solomon Islands	Group I	6,477,874,396 (5,107,238,198–8,165,200,639)	6,743,652,675 (5,312,259,857–8,377,787,354)	7,369,384,990 (5,840,700,581–9,276,114,294)	5,491,809,719 (4,280,954,712–7,051,438,643)
Solomon Islands	NCD	6,498,929,101 (5,523,868,767–7,450,372,709)	9,033,371,264 (7,718,089,134–10,481,956,908)	11,360,145,145 (9,749,238,080–13,206,741,442)	14,759,805,610 (12,790,888,685–17,167,710,219)
Solomon Islands	Injuries	1,151,569,841 (921,182,800–1,407,757,413)	1,734,101,829 (1,433,471,600–2,088,097,564)	2,064,287,422 (1,701,851,514–2,453,841,124)	2,544,076,610 (2,102,936,435–3,046,345,590)
Tonga	All causes	3,151,039,606 (2,677,446,957–3,691,807,930)	3,158,214,947 (2,719,234,691–3,703,064,672)	3,182,588,376 (2,726,894,097–3,693,599,293)	3,177,917,304 (2,733,593,261–3,690,382,832)
Tonga	Group I	1,023,702,214 (777,411,031–1,348,349,177)	840,487,868 (619,829,556–1,136,965,239)	801,762,172 (599,386,963–1,100,716,239)	668,461,596 (501,267,304–896,758,455)
Tonga	NCD	1,800,538,495 (1,547,678,800–2,064,847,323)	1,998,594,502 (1,759,214,158–2,270,965,219)	2,066,062,034 (1,813,960,319–2,358,583,102)	2,195,027,703 (1,890,160,042–2,532,974,273)
Tonga	Injuries	326,798,896 (274,308,335–386,907,837)	319,132,577 (270,495,176–377,025,134)	314,764,170 (264,860,345–371,612,905)	314,428,006 (261,398,111–372,117,922)
Vanuatu	All causes	6,803,954,875 (5,973,327,710–7,695,028,126)	8,094,498,620 (7,131,939,369–9,127,585,062)	9,471,059,885 (8,356,492,857–10,814,679,337)	11,176,491,743 (9,582,707,421–12,962,027,558)
Vanuatu	Group I	3,031,874,417 (2,507,284,388–3,646,655,950)	2,895,234,853 (2,415,788,058–3,494,896,596)	3,277,151,493 (2,692,200,413–4,017,570,842)	2,969,833,317 (2,342,599,813–3,833,427,867)
Vanuatu	NCD	3,251,474,185 (2,804,745,421–3,705,312,325)	4,403,324,559 (3,801,381,666–5,034,898,793)	5,280,323,751 (4,534,895,066–6,080,451,839)	7,085,100,696 (6,055,416,841–8,316,319,339)
Vanuatu	Injuries	520,606,273 (438,053,130–620,155,926)	795,939,207 (685,639,279–922,657,524)	913,584,641 (769,083,022–1,088,158,658)	1,121,557,731 (946,438,008–1,342,040,626)
North Africa and Middle East	All causes	14,958,617,000,100 (13,933,400,479,800–16,074,012,002,300)	14,582,562,328,400 (13,354,762,242,400–15,880,846,433,400)	15,145,472,133,500 (13,774,272,950,500–16,777,149,703,900)	16,525,150,035,300 (14,644,745,698,400–18,560,617,435,000)
North Africa and Middle East	Group I	6,316,655,740,300 (5,721,793,664,600–6,966,812,152,970)	4,855,382,724,780 (4,400,052,574,290–5,360,456,696,830)	4,139,145,589,350 (3,740,109,833,500–4,631,553,284,420)	3,251,124,931,310 (2,803,174,198,910–3,748,516,823,140)
North Africa and Middle East	NCD	6,707,119,810,430 (5,819,473,130,190–7,599,002,056,880)	7,955,696,241,820 (6,947,530,669,200–9,050,691,879,750)	8,901,179,284,880 (7,749,678,139,390–10,161,688,972,300)	10,569,104,010,800 (9,097,377,211,660–12,065,126,944,000)
North Africa and Middle East	Injuries	1,934,841,449,340 (1,687,275,243,650–2,188,297,601,720)	1,771,483,361,790 (1,643,512,372,380–1,933,498,748,640)	2,105,147,259,270 (1,872,532,870,980–2,366,735,765,470)	2,704,921,093,180 (2,346,879,014,540–3,104,760,782,640)
North Africa and Middle East	All causes	14,951,596,305,500 (13,926,663,939,000–16,066,487,825,500)	14,576,053,043,000 (13,348,499,201,200–15,874,346,981,800)	15,136,888,208,800 (13,766,174,428,200–16,768,384,404,200)	16,515,581,022,900 (14,635,205,471,700–18,550,673,720,500)
North Africa and Middle East	Group I	6,313,132,311,760 (5,718,609,431,130–6,962,905,994,250)	4,852,708,953,240 (4,397,624,387,340–5,357,404,800,370)	4,136,153,028,590 (3,737,443,123,070–4,628,112,220,100)	3,248,983,104,230 (2,801,190,350,700–3,746,226,838,580)
North Africa and Middle East	NCD	6,704,758,862,190 (5,817,363,325,700–7,596,669,577,950)	7,952,998,392,310 (6,944,958,122,460–9,047,961,333,120)	8,897,347,738,800 (7,746,026,166,010–10,157,779,095,800)	10,564,025,304,200 (9,092,071,120,290–12,059,956,311,400)
North Africa and Middle East	Injuries	1,933,705,131,560 (1,686,280,272,450–2,186,979,517,970)	1,770,345,697,450 (1,642,387,990,150–1,932,338,765,410)	2,103,387,441,430 (1,870,892,965,920–2,364,845,426,310)	2,702,572,614,560 (2,344,822,853,420–3,102,154,250,480)
Afghanistan	All causes	1,142,748,297,780 (966,355,209,067–1,316,568,614,950)	1,635,483,850,420 (1,421,760,610,290–1,838,036,345,230)	1,752,410,152,480 (1,551,423,665,690–1,968,148,948,730)	1,855,991,378,380 (1,579,700,536,640–2,159,683,058,860)
Afghanistan	Group I	709,770,254,664 (541,613,507,000–888,411,440,744)	929,606,476,231 (728,218,226,326–1,136,896,409,260)	843,559,953,259 (671,999,182,890–1,046,336,162,150)	658,887,918,804 (491,770,950,624–867,255,070,917)
Afghanistan	NCD	305,274,191,299 (236,286,551,805–380,484,033,998)	496,358,406,995 (402,210,402,211–594,280,411,471)	640,618,370,452 (526,133,248,648–751,899,383,526)	795,301,533,123 (685,718,498,238–904,268,731,608)
Afghanistan	Injuries	127,703,851,816 (98,970,818,292–164,600,556,542)	209,518,967,190 (172,405,430,680–252,822,203,030)	268,231,828,764 (233,839,096,400–309,295,221,070)	401,801,926,449 (286,169,714,287–525,442,140,894)
Algeria	All causes	864,813,160,619 (755,772,298,428–969,894,008,555)	910,704,176,228 (797,295,723,347–1,032,441,994,990)	913,355,075,302 (797,583,517,161–1,032,612,743,420)	939,878,164,414 (808,084,363,286–1,098,335,660,250)
Algeria	Group I	300,184,495,457 (242,322,324,269–367,216,115,679)	249,278,132,243 (198,949,859,722–307,237,348,237)	212,465,863,680 (171,897,572,769–258,279,773,869)	146,281,407,154 (114,332,036,240–188,573,770,758)
Algeria	NCD	461,320,085,065 (389,657,816,111–532,440,527,645)	546,642,907,900 (464,257,420,712–630,964,565,328)	596,061,706,389 (507,140,006,595–689,254,092,975)	691,966,387,521 (585,036,905,281–805,094,160,558)
Algeria	Injuries	103,308,580,097 (91,292,494,606–116,662,514,110)	114,783,136,085 (102,257,499,784–129,081,101,642)	104,827,505,233 (94,327,471,006–117,311,576,399)	101,630,369,738 (88,993,165,743–116,321,278,674)
Bahrain	All causes	12,779,449,135 (11,175,666,067–14,396,055,335)	15,484,922,398 (13,417,003,490–17,764,238,814)	19,022,056,746 (16,140,962,789–21,977,427,408)	26,175,434,673 (21,309,022,280–31,515,702,873)
Bahrain	Group I	2,332,448,241 (2,039,207,813–2,670,099,366)	1,774,844,354 (1,518,117,829–2,092,015,364)	2,011,763,562 (1,699,970,877–2,378,295,282)	2,370,816,548 (1,919,113,264–2,956,521,155)
Bahrain	NCD	8,965,578,393 (7,639,981,389–10,311,300,308)	11,193,533,904 (9,592,022,394–13,043,329,129)	14,394,066,129 (12,035,084,381–16,949,668,767)	21,029,588,874 (17,111,820,775–25,477,216,791)
Bahrain	Injuries	1,481,422,502 (1,301,786,478–1,675,963,198)	2,516,544,140 (1,938,580,110–3,123,316,190)	2,616,227,055 (2,237,004,245–3,066,465,294)	2,775,029,251 (2,244,729,117–3,458,687,203)
Egypt	All causes	2,682,610,205,730 (2,465,025,329,790–2,906,131,058,220)	2,266,388,164,930 (2,033,438,007,800–2,500,730,205,950)	2,414,412,782,470 (2,165,866,073,770–2,683,911,082,620)	2,545,370,659,600 (2,203,604,126,420–2,891,676,922,070)
Egypt	Group I	1,220,364,140,420 (1,099,352,761,530–1,343,381,905,020)	679,093,746,956 (587,089,289,720–781,962,908,255)	602,147,954,826 (511,578,181,069–703,944,878,775)	425,837,311,706 (325,930,919,611–559,979,278,443)
Egypt	NCD	1,219,732,476,570 (1,087,026,260,670–1,357,313,548,310)	1,375,916,834,930 (1,206,255,764,820–1,547,569,797,640)	1,586,258,427,820 (1,391,545,271,220–1,780,493,018,960)	1,874,186,511,560 (1,612,400,321,840–2,137,251,549,380)
Egypt	Injuries	242,513,588,739 (216,217,990,196–268,553,393,612)	211,377,583,043 (193,923,430,653–230,921,537,575)	226,006,399,821 (205,876,484,213–248,025,671,691)	245,346,836,326 (211,128,515,552–280,248,857,497)
Iran	All causes	2,447,106,564,510 (2,128,491,126,860–2,840,916,992,740)	1,863,129,602,100 (1,609,601,782,990–2,160,928,568,620)	1,998,300,533,660 (1,667,364,240,890–2,404,805,874,000)	1,995,442,433,500 (1,662,139,413,370–2,373,837,448,180)
Iran	Group I	782,479,345,744 (628,094,983,692–967,203,570,028)	348,988,760,019 (254,239,475,539–476,224,875,081)	312,017,481,150 (193,442,778,579–496,748,272,438)	236,553,414,114 (147,528,119,139–393,047,899,600)
Iran	NCD	1,052,380,045,880 (902,926,482,227–1,230,891,673,670)	1,160,874,010,910 (981,479,525,891–1,350,041,867,640)	1,317,816,711,050 (1,112,142,949,670–1,541,994,234,850)	1,477,388,445,220 (1,232,044,878,580–1,751,219,258,370)
Iran	Injuries	612,247,172,890 (415,641,420,369–808,624,526,345)	353,266,831,173 (307,247,815,897–403,226,222,877)	368,466,341,459 (310,146,048,384–436,081,525,456)	281,500,574,171 (235,899,191,855–338,699,233,620)
Iraq	All causes	743,216,245,016 (569,638,017,784–888,133,330,607)	916,936,686,399 (757,971,433,525–1,086,060,993,860)	1,251,146,916,220 (1,035,934,440,920–1,481,383,341,950)	1,414,186,188,300 (1,129,312,636,270–1,690,851,993,400)
Iraq	Group I	278,034,097,215 (148,200,244,304–379,081,806,858)	302,009,143,435 (192,679,914,725–415,780,633,712)	307,786,459,314 (230,014,274,480–394,614,350,386)	354,464,860,405 (204,060,688,761–496,131,202,009)
Iraq	NCD	364,125,749,771 (306,539,424,567–420,021,152,041)	487,076,650,908 (426,625,966,519–556,187,727,900)	576,262,391,581 (496,178,724,246–654,287,998,930)	727,168,183,604 (608,995,848,208–848,119,513,836)

Appendix Table 3. GBD 2016 DALY counts for all causes and level 1 cause groups by location for 1990, 2000, 2006 & 2016, both sexes combined.

	Cause	1990	2000	2006	2016
Iraq	Injuries	101,056,398,030<br>(82,175,778,782–122,214,339,320)	127,850,892,056<br>(107,878,214,092–150,116,210,909)	367,098,065,320<br>(188,742,797,228–543,669,823,023)	332,553,144,293<br>(201,074,177,284–480,174,327,223)
Jordan	All causes	94,871,228,649<br>(84,699,100,886–105,711,086,806)	119,768,877,689<br>(104,916,241,014–135,958,611,773)	129,138,475,601<br>(111,956,066,942–147,057,231,241)	169,169,208,118<br>(144,554,453,533–196,983,372,903)
Jordan	Group I	26,908,650,972<br>(23,643,668,227–30,527,139,011)	27,149,479,960<br>(23,894,634,957–30,781,109,522)	26,236,850,017<br>(23,116,466,622–29,863,979,748)	26,452,690,024<br>(21,941,352,438–31,441,245,659)
Jordan	NCD	56,467,337,145<br>(49,001,816,918–64,629,534,285)	78,477,344,214<br>(67,027,909,367–90,773,468,012)	89,001,009,502<br>(75,138,847,044–103,470,733,181)	117,676,064,406<br>(98,326,850,133–139,208,822,683)
Jordan	Injuries	11,495,240,533<br>(10,300,009,352–12,756,122,795)	14,142,053,516<br>(12,229,455,479–16,181,641,239)	13,900,616,081<br>(12,134,010,729–16,023,535,320)	25,040,453,689<br>(18,371,795,499–31,551,217,667)
Kuwait	All causes	40,252,494,576<br>(33,866,478,890–48,078,048,609)	35,373,361,955<br>(30,258,316,010–41,342,102,598)	43,657,757,340<br>(36,906,325,409–50,998,732,033)	65,262,996,927<br>(52,376,037,972–79,184,680,711)
Kuwait	Group I	5,261,965,553<br>(4,476,800,339–6,193,494,659)	4,433,688,427<br>(3,822,152,581–5,153,767,994)	5,091,698,636<br>(4,405,512,470–5,979,003,116)	6,222,654,611<br>(4,983,954,104–7,776,662,258)
Kuwait	NCD	25,518,975,947<br>(21,086,574,465–30,384,312,941)	26,399,296,997<br>(22,224,752,802–31,302,824,729)	32,674,305,026<br>(27,153,679,266–38,712,703,417)	51,790,634,144<br>(41,004,067,825–63,028,105,210)
Kuwait	Injuries	9,471,553,076<br>(5,551,818,716–13,659,179,555)	4,540,376,532<br>(3,935,665,565–5,229,819,473)	5,891,753,678<br>(5,079,280,364–6,800,589,181)	7,249,708,172<br>(5,675,761,048–9,102,772,566)
Lebanon	All causes	99,823,137,376<br>(89,453,772,614–110,984,918,167)	77,897,587,614<br>(67,659,532,457–89,237,939,525)	90,441,286,328<br>(77,751,809,886–104,470,269,553)	124,128,724,280<br>(104,536,528,260–145,453,971,702)
Lebanon	Group I	16,412,762,336<br>(13,422,346,346–19,822,612,577)	9,770,107,388<br>(7,899,057,776–11,919,389,562)	8,611,961,879<br>(6,805,435,517–10,977,829,484)	9,162,601,364<br>(7,237,944,526–11,523,905,606)
Lebanon	NCD	61,150,195,659<br>(53,331,993,003–69,413,816,490)	58,797,381,984<br>(50,679,449,332–67,797,258,900)	68,498,239,781<br>(58,156,280,014–79,582,400,485)	100,188,997,947<br>(84,034,393,746–117,639,630,065)
Lebanon	Injuries	22,260,179,381<br>(18,348,304,216–26,494,442,100)	9,330,098,242<br>(7,189,423,844–12,172,097,209)	13,331,084,668<br>(10,263,840,046–16,837,713,151)	14,777,124,969<br>(10,735,782,642–19,439,113,069)
Libya	All causes	115,443,505,008<br>(101,574,573,172–131,351,221,809)	132,128,193,760<br>(114,821,398,321–150,315,587,460)	136,094,512,152<br>(116,699,750,821–157,151,668,220)	147,543,798,358<br>(126,048,036,282–171,277,030,177)
Libya	Group I	27,619,682,129<br>(22,026,636,593–33,971,839,356)	24,900,638,678<br>(20,276,024,038–30,031,896,300)	21,283,745,392<br>(16,837,331,855–26,076,760,305)	13,523,425,303<br>(10,621,143,529–16,855,505,298)
Libya	NCD	73,471,207,663<br>(63,045,794,807–84,655,060,652)	89,549,352,362<br>(76,438,276,790–103,226,715,499)	97,410,562,203<br>(82,474,144,233–113,547,463,004)	107,105,999,697<br>(90,931,500,572–125,228,279,725)
Libya	Injuries	14,352,615,216<br>(12,776,673,781–16,109,709,351)	17,678,202,721<br>(15,600,620,767–19,804,274,494)	17,400,204,556<br>(15,265,340,704–19,717,480,751)	26,914,373,358<br>(18,194,708,862–36,134,326,207)
Morocco	All causes	1,053,289,085,350<br>(969,837,720,056–1,140,440,573,220)	921,616,688,416<br>(828,891,910,107–1,021,767,335,560)	891,054,964,769<br>(788,068,896,435–1,001,415,259,150)	893,475,714,094<br>(780,358,711,340–1,025,441,631,500)
Morocco	Group I	519,627,828,121<br>(474,458,325,759–574,332,166,699)	304,046,920,692<br>(263,551,959,619–355,595,543,875)	230,710,491,945<br>(196,178,606,668–273,562,191,901)	146,088,029,720<br>(120,673,349,122–178,132,196,618)
Morocco	NCD	434,995,666,165<br>(370,423,990,315–500,504,285,030)	523,376,550,636<br>(445,783,372,375–600,226,675,332)	568,595,914,014<br>(483,114,078,776–653,524,984,238)	656,098,996,281<br>(558,629,235,742–758,530,848,955)
Morocco	Injuries	98,665,591,062<br>(86,785,636,693–110,303,826,453)	94,193,217,088<br>(84,078,113,545–104,115,807,813)	91,748,558,810<br>(81,643,606,731–102,190,887,293)	91,288,688,094<br>(81,306,497,116–102,039,383,896)
Palestine	All causes	64,609,076,939<br>(47,208,800,764–79,614,836,942)	81,264,627,597<br>(64,225,108,824–98,244,220,594)	92,263,306,681<br>(76,355,008,949–108,639,165,879)	123,471,053,559<br>(103,015,183,976–147,182,065,851)
Palestine	Group I	21,015,579,060<br>(10,986,813,287–29,653,403,804)	21,577,372,070<br>(12,345,735,780–30,723,574,973)	23,889,577,064<br>(15,572,058,985–32,234,124,430)	29,522,759,095<br>(18,703,571,243–42,169,300,697)
Palestine	NCD	35,935,999,523<br>(28,450,172,501–43,103,442,454)	48,830,789,094<br>(40,752,918,920–56,755,279,930)	57,932,857,551<br>(49,336,654,870–66,679,062,973)	82,258,505,290<br>(70,662,233,414–95,314,695,688)
Palestine	Injuries	7,657,498,356<br>(5,709,680,228–9,564,993,851)	10,856,466,434<br>(7,584,982,250–14,050,968,899)	10,440,872,066<br>(8,432,939,940–12,556,505,586)	11,689,789,174<br>(9,762,678,088–13,850,472,717)
Oman	All causes	64,177,379,944<br>(56,153,077,183–73,144,348,497)	51,098,332,001<br>(44,308,294,510–58,257,749,997)	49,759,846,760<br>(42,845,904,060–57,592,688,805)	89,275,562,042<br>(75,774,606,924–103,898,163,004)
Oman	Group I	20,009,792,812<br>(15,506,877,381–25,296,834,258)	10,830,945,751<br>(9,023,700,776–12,927,505,615)	7,123,027,193<br>(6,024,157,333–8,437,307,014)	9,428,041,176<br>(7,881,859,158–11,468,710,926)
Oman	NCD	32,824,108,871<br>(27,767,707,636–38,609,972,371)	31,179,400,945<br>(26,337,600,677–36,308,766,395)	33,825,867,454<br>(28,225,993,876–39,974,387,670)	63,593,424,417<br>(52,402,773,520–75,821,639,375)
Oman	Injuries	11,343,478,261<br>(9,703,859,497–12,951,789,570)	9,087,985,304<br>(8,209,506,710–10,096,788,703)	8,810,952,113<br>(7,946,544,504–9,851,823,290)	16,254,096,450<br>(14,213,732,926–18,504,182,986)
Qatar	All causes	10,376,375,940<br>(8,792,579,477–12,147,863,330)	12,669,218,035<br>(10,849,313,822–14,846,882,608)	18,727,280,899<br>(15,630,604,446–21,856,141,625)	37,704,406,384<br>(30,159,003,222–45,756,279,146)
Qatar	Group I	1,582,876,707<br>(1,259,753,169–2,009,267,027)	1,337,205,077<br>(1,068,329,259–1,676,349,971)	1,623,385,059<br>(1,294,921,289–2,016,432,216)	2,764,332,352<br>(2,193,068,656–3,462,698,509)
Qatar	NCD	7,061,707,177<br>(5,876,881,734–8,398,482,724)	8,936,225,705<br>(7,484,007,304–10,570,519,473)	13,052,352,941<br>(10,657,024,633–15,651,056,072)	27,577,771,201<br>(21,771,334,788–34,097,059,092)
Qatar	Injuries	1,731,792,056<br>(1,451,107,577–2,099,565,683)	2,395,787,254<br>(2,002,328,782–2,915,670,345)	4,051,542,899<br>(3,325,845,213–4,802,986,717)	7,362,302,831<br>(5,699,095,928–9,427,439,819)
Saudi Arabia	All causes	551,366,923,617<br>(492,127,158,233–617,231,600,572)	527,414,001,282<br>(464,822,607,004–597,621,010,378)	543,943,018,697<br>(476,162,978,998–621,519,894,873)	585,360,139,236<br>(498,851,052,746–688,785,360,343)
Saudi Arabia	Group I	181,444,529,348<br>(152,027,674,760–222,021,057,389)	128,143,760,851<br>(108,615,487,008–151,690,243,927)	99,403,265,090<br>(83,509,995,125–117,745,636,088)	64,508,067,202<br>(53,122,535,055–79,746,771,078)
Saudi Arabia	NCD	279,436,643,847<br>(233,999,377,557–324,748,729,190)	317,183,941,093<br>(270,837,186,344–366,968,963,203)	352,891,588,158<br>(298,360,336,610–413,448,958,142)	430,303,288,440<br>(354,968,082,804–511,384,610,181)
Saudi Arabia	Injuries	90,485,750,422<br>(80,433,889,593–101,123,124,582)	82,086,299,338<br>(76,073,425,390–89,027,453,355)	91,648,165,449<br>(84,704,571,644–99,135,426,317)	90,548,783,594<br>(80,324,025,394–101,541,180,265)
Sudan	All causes	1,267,560,958,040<br>(1,151,931,706,410–1,389,605,618,360)	1,463,687,660,630<br>(1,314,694,442,840–1,631,997,662,700)	1,420,529,550,710<br>(1,249,269,610,100–1,602,115,184,930)	1,416,049,067,220<br>(1,171,610,637,290–1,751,990,112,580)
Sudan	Group I	679,943,797,160<br>(573,776,535,839–800,549,384,528)	723,053,027,841<br>(601,279,437,406–867,823,986,014)	603,931,844,251<br>(483,303,384,900–739,331,455,784)	469,626,616,908<br>(330,856,482,280–688,009,287,273)
Sudan	NCD	454,037,836,012<br>(361,820,040,602–537,478,331,813)	581,716,702,769<br>(487,447,619,920–674,845,286,510)	642,849,209,261<br>(544,899,584,223–744,628,195,632)	755,448,842,838<br>(633,460,465,870–897,641,499,559)
Sudan	Injuries	133,579,324,872<br>(108,882,181,725–157,433,747,882)	158,917,930,020<br>(137,015,300,328–184,394,126,712)	173,748,497,201<br>(151,108,889,840–196,529,120,284)	190,973,607,478<br>(162,084,974,766–219,573,728,700)
Syria	All causes	394,990,596,880<br>(350,018,201,603–443,132,327,590)	373,889,290,613<br>(329,781,580,700–420,085,974,283)	393,276,965,502<br>(342,531,203,898–447,292,579,123)	689,984,970,347<br>(437,565,116,238–949,445,009,900)
Syria	Group I	130,521,878,927<br>(106,879,394,344–157,042,337,098)	74,728,880,685<br>(63,978,829,548–86,249,983,849)	70,276,477,674<br>(59,443,473,448–82,359,031,394)	44,402,427,676<br>(35,795,877,002–55,462,577,478)
Syria	NCD	235,106,690,418<br>(204,886,574,583–266,114,551,907)	267,754,425,708<br>(233,078,541,706–303,790,565,998)	288,027,101,422<br>(248,250,263,160–332,014,935,539)	279,532,014,110<br>(238,329,756,425–323,412,921,649)
Syria	Injuries	29,362,027,535<br>(25,884,669,254–33,258,749,116)	31,405,984,221<br>(28,321,232,825–35,328,294,615)	34,973,386,407<br>(30,891,687,793–39,556,987,368)	366,050,528,561<br>(127,111,540,681–618,755,631,063)
Tunisia	All causes	261,623,750,357<br>(237,502,631,930–288,574,868,425)	242,812,993,434<br>(213,905,529,501–276,429,379,523)	249,263,123,896<br>(216,297,499,588–286,361,498,670)	269,620,250,309<br>(228,555,877,663–315,365,487,243)
Tunisia	Group I	67,488,244,811<br>(57,050,264,899–81,113,409,079)	36,923,826,498<br>(31,785,782,331–42,785,679,910)	31,598,958,145<br>(27,203,614,460–36,627,164,814)	23,997,408,328<br>(20,021,295,863–28,733,764,140)
Tunisia	NCD	163,652,567,002<br>(140,737,598,423–187,433,243,175)	177,630,879,675<br>(151,921,136,872–204,492,456,076)	189,564,396,390<br>(161,201,306,485–220,680,995,710)	217,712,554,163<br>(182,841,047,999–254,617,555,562)
Tunisia	Injuries	30,482,938,544<br>(26,963,221,082–34,504,793,065)	28,258,287,260<br>(24,510,863,540–32,248,329,034)	28,099,769,361<br>(23,789,940,757–32,736,429,661)	27,910,287,818<br>(23,262,051,507–33,427,547,507)
Turkey	All causes	2,069,600,177,690<br>(1,844,937,295,830–2,301,948,666,430)	1,844,193,370,410<br>(1,632,139,026,620–2,045,682,115,620)	1,668,899,583,910<br>(1,459,801,137,640–1,901,035,556,050)	1,782,453,251,790<br>(1,512,743,878,610–2,073,250,688,570)
Turkey	Group I	744,429,574,384<br>(607,536,131,848–893,018,302,539)	417,091,657,672<br>(352,772,659,095–489,343,812,669)	261,007,421,462<br>(220,235,547,298–307,240,489,481)	179,253,751,791<br>(142,571,449,788–227,009,880,296)
Turkey	NCD	1,131,472,636,570<br>(976,035,365,075–1,291,589,351,210)	1,249,432,606,600<br>(1,078,425,224,150–1,420,098,385,660)	1,261,341,898,040<br>(1,073,795,173,390–1,458,278,080,190)	1,445,368,868,110<br>(1,215,617,944,700–1,684,495,629,560)
Turkey	Injuries	193,697,966,738<br>(170,836,307,183–220,769,486,474)	177,669,106,138<br>(158,928,264,124–199,823,259,295)	146,550,264,408<br>(130,654,686,298–166,234,515,416)	157,830,631,895<br>(132,991,924,375–186,490,167,885)
United Arab Emirates	All causes	45,582,120,982<br>(37,224,333,009–54,849,159,410)	63,315,625,234<br>(53,222,963,761–74,161,969,051)	98,846,815,022<br>(83,742,096,561–115,402,291,483)	204,025,524,414<br>(169,493,260,025–244,193,252,074)
United Arab Emirates	Group I	8,458,781,656<br>(5,551,572,813–13,057,266,880)	7,581,504,093<br>(5,771,346,481–10,289,402,432)	9,252,727,078<br>(7,433,985,122–11,544,404,633)	15,593,070,489<br>(12,034,919,805–20,023,874,806)
United Arab Emirates	NCD	29,152,735,057<br>(23,337,094,747–35,232,747,404)	43,960,604,750<br>(36,314,537,872–52,128,408,881)	70,178,036,722<br>(58,594,465,689–83,542,607,409)	150,409,450,599<br>(124,156,025,978–180,978,454,367)
United Arab Emirates	Injuries	7,970,604,269<br>(6,274,807,324–9,846,121,228)	11,773,516,390<br>(9,669,244,787–14,089,840,036)	19,416,051,222<br>(16,581,023,861–22,435,213,164)	38,023,003,326<br>(28,770,180,450–47,780,146,334)
Yemen	All causes	922,507,878,476<br>(744,339,554,534–1,053,651,639,220)	1,017,620,285,640<br>(774,386,427,938–1,214,863,046,190)	957,575,723,663<br>(744,637,255,814–1,160,424,091,410)	1,134,324,987,830<br>(903,191,929,663–1,376,273,106,960)
Yemen	Group I	568,872,063,632<br>(413,500,679,785–694,058,571,808)	549,930,134,108<br>(350,906,501,040–718,626,291,813)	455,501,338,963<br>(300,007,833,686–622,061,621,238)	383,335,966,731<br>(222,553,111,828–558,559,877,011)
Yemen	NCD	270,958,447,874<br>(210,403,028,799–348,582,630,422)	369,198,024,163<br>(303,234,889,633–450,418,478,282)	396,244,091,018<br>(326,945,745,364–466,213,291,244)	486,366,151,125<br>(410,499,911,030–571,927,179,948)
Yemen	Injuries	82,677,366,971<br>(67,847,797,217–97,819,277,550)	98,492,127,373<br>(85,073,543,180–112,501,833,776)	105,830,293,683<br>(92,930,434,515–119,752,388,376)	264,622,869,971<br>(233,945,874,633–308,725,076,708)
South Asia	All causes	69,241,703,665,300<br>(65,921,273,047,400–72,901,296,534,500)	66,508,113,364,700<br>(62,800,230,865,900–70,775,034,159,300)	64,762,731,812,700<br>(60,573,138,208,800–69,421,204,734,900)	59,101,340,911,700<br>(54,185,922,640,400–64,717,900,474,000)
South Asia	Group I	42,908,885,330,100<br>(41,301,139,820,400–44,621,513,668,900)	34,990,705,222,200<br>(33,553,364,697,400–36,678,254,910,500)	30,492,468,724,800<br>(29,097,458,811,500–32,126,168,376,000)	19,636,819,112,500<br>(18,167,196,690,300–21,247,676,578,500)
South Asia	NCD	20,572,446,620,400<br>(18,297,205,227,400–23,084,388,635,300)	25,115,527,920,000<br>(22,233,729,491,900–28,169,614,306,300)	27,644,966,030,000<br>(24,497,395,194,300–30,972,218,184,700)	32,786,124,643,900<br>(29,280,426,474,300–36,817,672,718,100)
South Asia	Injuries	5,760,371,714,890<br>(5,130,556,018,280–6,286,614,908,510)	6,401,880,222,530<br>(5,747,620,717,770–6,928,581,032,720)	6,625,297,057,970<br>(5,971,648,746,950–7,216,936,124,560)	6,678,397,155,330<br>(6,039,641,648,580–7,333,858,455,250)
Bangladesh	All causes	7,171,488,281,000<br>(6,773,452,226,680–7,598,370,538,180)	5,672,650,455,420<br>(5,241,452,775,440–6,125,986,760,870)	5,225,521,850,890<br>(4,817,383,169,950–5,691,095,747,900)	4,460,497,452,290<br>(3,971,261,165,140–5,011,023,076,020)
Bangladesh	Group I	4,509,987,917,900<br>(4,255,289,217,370–4,770,976,211,270)	2,798,225,724,530<br>(2,604,009,331,460–3,002,799,082,320)	2,157,819,416,430<br>(2,000,480,442,430–2,332,660,976,960)	1,193,255,331,240<br>(1,062,033,000,770–1,340,547,759,080)
Bangladesh	NCD	1,972,350,295,580<br>(1,719,617,295,830–2,235,385,641,160)	2,242,930,372,570<br>(1,949,481,394,860–2,551,555,340,960)	2,493,564,402,920<br>(2,176,357,144,870–2,831,919,574,490)	2,796,753,675,870<br>(2,415,620,289,050–3,193,092,458,920)
Bangladesh	Injuries	689,150,067,521<br>(562,200,133,539–778,812,132,419)	631,494,358,316<br>(483,365,618,925–707,454,861,309)	574,138,031,545<br>(464,676,083,669–638,312,424,474)	470,488,445,178<br>(402,413,210,012–538,882,711,906)
Bhutan	All causes	38,153,653,247<br>(34,573,065,002–41,876,523,775)	29,098,532,763<br>(26,165,053,312–32,275,090,298)	25,553,502,739<br>(22,696,562,218–28,438,327,799)	21,794,044,890<br>(18,973,256,044–24,915,682,310)
Bhutan	Group I	24,965,337,130<br>(21,836,090,512–28,263,659,900)	15,559,688,053<br>(13,585,743,938–17,853,337,331)	12,177,071,892<br>(10,345,456,446–14,059,940,401)	6,770,049,614<br>(5,645,329,175–8,146,484,279)

Appendix Table 3. GBD 2016 DALY counts for all causes and level 1 cause groups by location for 1990, 2000, 2006 & 2016, both sexes combined.

	Cause	1990	2000	2006	2016
Bhutan	NCD	10,616,996,953<br>(9,058,717,420–12,085,973,531)	10,112,108,811<br>(8,711,888,443–11,546,755,841)	11,172,397,462<br>(9,574,407,073–12,686,523,205)	12,982,354,792<br>(11,057,456,118–15,062,248,156)
Bhutan	Injuries	2,571,319,164<br>(2,054,141,819–3,017,845,695)	3,426,735,899<br>(2,621,633,212–4,211,381,968)	2,204,033,385<br>(1,935,112,039–2,505,434,646)	2,041,640,484<br>(1,717,167,184–2,429,050,494)
India	All causes	53,745,723,377,600<br>(51,006,616,771,900–56,542,697,827,200)	52,733,454,505,700<br>(49,740,597,512,400–56,062,741,737,100)	51,337,420,074,100<br>(47,927,056,686,500–54,975,078,402,700)	46,633,653,208,600<br>(42,823,488,379,100–51,029,274,102,900)
India	Group I	32,717,234,308,500<br>(31,345,273,171,500–34,183,194,739,500)	27,666,204,270,900<br>(26,482,823,533,100–29,156,760,460,600)	24,185,512,613,800<br>(22,979,493,525,600–25,579,204,472,500)	15,258,141,873,200<br>(14,008,607,699,600–16,669,736,444,000)
India	NCD	16,422,864,767,600<br>(14,653,396,538,000–18,413,903,788,300)	19,868,236,178,600<br>(17,625,246,466,000–22,269,681,219,300)	21,726,555,873,600<br>(19,263,169,058,100–24,403,535,852,700)	25,852,552,385,000<br>(23,070,353,915,200–28,954,560,367,900)
India	Injuries	4,605,624,301,540<br>(4,104,935,856,210–5,034,604,623,870)	5,199,014,056,140<br>(4,663,423,720,110–5,623,076,209,970)	5,425,351,586,790<br>(4,865,092,729,690–5,914,697,074,430)	5,522,958,950,330<br>(4,974,389,056,470–6,051,703,181,170)
Nepal	All causes	1,422,656,897,860<br>(1,230,606,101,840–1,618,890,724,470)	1,176,018,623,020<br>(1,060,054,410,770–1,304,807,466,720)	1,033,037,510,200<br>(931,812,972,453–1,142,661,609,980)	922,853,991,255<br>(814,627,867,331–1,034,240,640,300)
Nepal	Group I	1,002,743,801,230<br>(829,225,505,982–1,186,526,429,700)	683,675,796,262<br>(581,883,640,465–791,759,293,306)	498,140,894,372<br>(424,989,362,024–575,796,169,932)	317,055,210,795<br>(263,591,508,318–379,158,337,584)
Nepal	NCD	327,648,912,166<br>(285,920,642,290–370,219,001,760)	393,670,709,965<br>(346,661,126,634–447,887,128,501)	438,251,025,217<br>(383,014,081,676–495,577,378,728)	515,043,096,284<br>(449,375,932,629–585,154,842,385)
Nepal	Injuries	92,264,184,463<br>(76,134,041,504–110,583,497,738)	98,672,116,792<br>(86,886,075,558–110,239,871,567)	96,645,590,608<br>(84,562,640,466–107,848,415,995)	90,755,684,176<br>(78,224,035,466–102,874,227,841)
Pakistan	All causes	6,863,681,455,630<br>(6,345,485,168,120–7,410,762,472,200)	6,896,891,247,830<br>(6,431,249,081,050–7,444,531,194,190)	7,141,198,874,780<br>(6,573,339,802,860–7,757,157,599,500)	7,062,542,214,670<br>(6,249,236,467,570–7,893,890,276,120)
Pakistan	Group I	4,653,953,965,300<br>(4,257,529,277,720–5,080,831,814,970)	3,827,039,742,390<br>(3,555,676,155,390–4,134,000,615,700)	3,638,818,728,280<br>(3,317,580,515,260–4,000,472,377,000)	2,861,596,647,560<br>(2,393,445,303,240–3,394,403,662,480)
Pakistan	NCD	1,838,965,648,120<br>(1,607,075,270,520–2,071,884,444,530)	2,600,578,550,050<br>(2,316,544,234,380–2,893,438,476,960)	2,975,422,330,860<br>(2,628,786,416,230–3,326,241,121,960)	3,608,793,131,950<br>(3,145,865,227,620–4,129,274,905,790)
Pakistan	Injuries	370,761,842,200<br>(315,899,671,503–430,145,042,759)	469,272,955,389<br>(409,341,670,801–534,730,035,232)	526,957,815,638<br>(464,354,741,825–598,704,174,987)	592,152,435,154<br>(507,231,958,987–686,533,311,450)
Sub-Saharan Africa	All causes	45,245,795,572,100<br>(43,338,798,468,400–47,183,428,638,300)	55,011,850,684,800<br>(52,538,074,216,000–57,584,758,288,900)	56,418,527,658,200<br>(53,939,461,925,500–59,250,153,808,700)	50,035,314,508,400<br>(46,998,834,295,900–53,498,056,084,200)
Sub-Saharan Africa	Group I	34,079,306,655,700<br>(32,663,403,022,900–35,579,135,007,000)	41,470,571,515,600<br>(39,693,564,473,300–43,258,652,242,700)	41,850,252,502,400<br>(40,078,236,672,700–43,746,116,459,900)	32,514,707,613,600<br>(30,708,771,891,200–34,599,743,284,900)
Sub-Saharan Africa	NCD	8,302,844,265,390<br>(7,238,114,299,360–9,396,801,485,980)	10,304,987,706,500<br>(8,964,747,091,990–11,734,851,843,600)	11,527,104,288,600<br>(10,048,998,069,200–13,109,742,588,300)	14,138,429,163,500<br>(12,288,424,848,200–16,187,009,156,600)
Sub-Saharan Africa	Injuries	2,863,644,650,940<br>(2,486,068,322,240–3,219,329,852,560)	3,236,291,462,700<br>(2,903,628,493,200–3,571,182,543,420)	3,041,170,867,200<br>(2,755,682,548,560–3,315,644,910,210)	3,382,177,731,240<br>(3,135,082,863,200–3,662,411,123,110)
Southern Sub-Saharan Africa	All causes	2,528,403,071,980<br>(2,367,517,539,350–2,704,666,643,940)	4,581,581,330,600<br>(4,287,978,488,720–4,899,207,627,360)	5,801,387,614,270<br>(5,482,453,215,070–6,161,989,975,540)	4,016,237,969,390<br>(3,733,878,507,740–4,334,937,383,320)
Southern Sub-Saharan Africa	Group I	1,281,256,718,260<br>(1,199,201,771,380–1,375,337,652,750)	2,895,623,357,830<br>(2,657,112,959,520–3,152,952,676,960)	3,965,070,299,400<br>(3,660,890,739,940–4,277,339,775,750)	2,200,485,206,790<br>(2,021,656,406,020–2,384,048,466,510)
Southern Sub-Saharan Africa	NCD	859,424,857,132<br>(759,787,832,617–969,590,829,650)	1,205,121,028,200<br>(1,060,226,680,920–1,361,332,611,670)	1,332,226,849,400<br>(1,177,549,797,050–1,499,144,317,640)	1,374,941,618,840<br>(1,209,225,333,060–1,558,070,027,700)
Southern Sub-Saharan Africa	Injuries	387,721,496,587<br>(354,782,561,252–415,216,973,052)	480,836,944,575<br>(430,811,700,532–533,395,160,107)	504,090,465,471<br>(452,392,450,265–566,430,572,354)	440,811,143,767<br>(401,549,257,923–476,994,245,533)
Botswana	All causes	52,630,424,364<br>(44,719,832,971–60,966,538,576)	154,924,531,942<br>(113,100,245,524–200,841,306,430)	151,842,441,453<br>(122,888,674,218–186,903,682,220)	89,007,855,765<br>(70,954,435,866–105,607,883,362)
Botswana	Group I	29,490,565,572<br>(23,582,456,960–36,164,814,917)	117,030,166,571<br>(86,341,945,976–154,030,939,320)	111,417,765,075<br>(88,942,565,610–138,572,380,747)	42,945,361,626<br>(35,796,839,399–50,933,985,288)
Botswana	NCD	18,577,049,259<br>(15,316,120,092–21,915,096,195)	29,436,879,449<br>(19,261,979,002–46,818,294,154)	31,237,462,810<br>(22,528,590,280–44,930,904,765)	37,090,102,205<br>(26,015,695,944–47,560,258,584)
Botswana	Injuries	4,562,809,532<br>(3,661,398,049–5,509,332,706)	8,457,485,922<br>(4,828,148,595–15,291,369,887)	9,187,213,568<br>(5,863,519,697–13,796,414,232)	8,972,391,934<br>(5,122,377,509–12,064,397,680)
Lesotho	All causes	84,225,646,024<br>(74,181,616,351–94,534,469,316)	152,617,231,504<br>(134,513,927,739–176,040,296,589)	200,702,936,241<br>(175,888,352,871–230,029,720,423)	167,405,444,010<br>(145,574,113,165–191,721,137,585)
Lesotho	Group I	49,393,859,984<br>(41,087,853,143–57,881,673,796)	105,795,091,110<br>(89,885,658,737–126,567,982,600)	146,847,045,554<br>(124,821,492,447–173,662,593,510)	108,756,915,952<br>(93,298,411,226–128,896,812,052)
Lesotho	NCD	26,679,200,607<br>(23,017,454,498–30,838,708,815)	34,755,069,030<br>(28,157,546,876–40,676,075,483)	39,504,025,206<br>(33,136,620,353–46,188,124,314)	42,377,180,925<br>(35,052,874,643–50,152,114,260)
Lesotho	Injuries	8,152,585,433<br>(6,835,067,398–9,271,486,958)	12,067,071,364<br>(9,411,796,186–14,425,951,596)	14,351,865,481<br>(11,639,792,073–17,038,565,765)	16,271,347,133<br>(12,737,555,192–20,108,609,622)
Namibia	All causes	72,168,840,600<br>(65,879,046,590–79,084,077,879)	127,664,498,381<br>(110,462,388,227–148,245,522,049)	143,664,856,430<br>(125,432,409,461–164,888,442,741)	102,531,728,716<br>(88,292,749,111–118,729,558,407)
Namibia	Group I	42,424,837,664<br>(38,303,994,491–47,184,388,627)	85,836,721,277<br>(71,996,480,983–104,748,695,225)	101,685,753,092<br>(84,745,081,874–121,897,174,360)	56,823,143,358<br>(47,329,778,902–70,224,818,016)
Namibia	NCD	22,339,707,367<br>(19,575,787,645–25,361,147,028)	30,823,792,239<br>(23,770,568,002–37,538,010,854)	31,434,143,688<br>(25,360,165,417–37,853,476,502)	34,864,778,470<br>(27,737,659,257–41,822,059,726)
Namibia	Injuries	7,404,295,568<br>(6,632,074,867–8,187,061,199)	11,003,984,866<br>(8,684,407,169–13,211,667,679)	10,544,959,650<br>(8,742,415,819–12,422,392,532)	10,843,806,888<br>(8,259,990,784–12,868,668,019)
South Africa	All causes	1,771,254,697,570<br>(1,651,178,329,960–1,905,153,593,270)	3,001,112,279,020<br>(2,798,772,203,680–3,211,374,257,830)	4,024,363,066,670<br>(3,760,345,679,970–4,302,293,887,230)	2,751,019,485,310<br>(2,535,892,946,470–3,003,627,044,180)
South Africa	Group I	803,626,294,674<br>(742,274,039,366–875,222,004,268)	1,704,699,646,630<br>(1,541,532,338,040–1,896,522,090,130)	2,621,150,098,660<br>(2,358,753,412,010–2,888,383,153,570)	1,429,143,062,290<br>(1,276,737,427,880–1,592,771,744,620)
South Africa	NCD	642,406,257,282<br>(565,893,748,429–724,586,540,331)	905,423,122,826<br>(801,987,735,117–1,021,222,311,710)	994,319,581,856<br>(875,631,153,916–1,120,117,056,280)	986,402,880,673<br>(865,733,940,729–1,117,241,142,340)
South Africa	Injuries	325,222,145,618<br>(297,155,919,300–348,229,964,834)	390,989,509,564<br>(346,521,914,825–438,577,049,837)	408,893,386,149<br>(359,365,354,131–468,285,990,566)	335,473,542,345<br>(300,212,616,241–368,905,954,890)
Swaziland	All causes	39,145,856,746<br>(35,003,525,304–43,542,656,211)	89,771,908,019<br>(75,739,623,892–106,439,502,753)	116,477,960,016<br>(100,279,444,971–133,908,183,148)	77,295,497,011<br>(66,409,875,366–89,671,772,921)
Swaziland	Group I	21,903,505,351<br>(18,933,711,657–24,953,473,453)	65,714,526,853<br>(53,760,210,624–81,876,684,696)	88,129,157,606<br>(73,839,954,907–105,318,861,113)	48,053,258,680<br>(41,684,306,190–56,888,013,386)
Swaziland	NCD	12,802,108,332<br>(10,985,109,516–14,729,163,254)	17,593,454,744<br>(13,782,714,939–23,426,276,042)	20,771,423,590<br>(17,237,618,396–26,269,631,975)	21,865,942,776<br>(17,061,159,881–27,310,375,250)
Swaziland	Injuries	4,440,243,062<br>(3,902,060,417–5,040,756,684)	6,463,926,422<br>(4,900,498,815–8,804,706,642)	7,577,378,821<br>(6,040,779,580–10,000,055,813)	7,376,295,554<br>(5,547,329,862–9,497,835,153)
Zimbabwe	All causes	508,977,606,675<br>(454,447,869,989–572,482,318,427)	1,055,490,881,730<br>(901,361,473,653–1,229,673,914,750)	1,164,336,353,460<br>(1,022,369,779,130–1,317,227,060,350)	828,977,958,578<br>(744,785,696,146–915,221,222,809)
Zimbabwe	Group I	334,417,655,017<br>(293,331,854,484–395,062,894,739)	816,547,205,386<br>(670,752,904,093–980,160,210,299)	895,840,479,409<br>(767,671,529,301–1,040,305,426,490)	514,763,464,876<br>(459,816,590,504–581,012,357,431)
Zimbabwe	NCD	136,620,534,285<br>(109,656,561,947–160,984,313,240)	187,088,709,906<br>(148,769,832,298–227,687,230,137)	214,960,212,248<br>(178,021,897,545–250,246,398,572)	252,340,733,789<br>(213,767,473,271–293,607,457,916)
Zimbabwe	Injuries	37,939,417,373<br>(28,416,741,300–45,862,167,371)	51,854,966,437<br>(40,326,594,482–65,203,301,155)	53,535,661,802<br>(45,176,265,190–69,056,970,838)	61,873,759,913<br>(51,136,227,459–76,556,449,288)
Western Sub-Saharan Africa	All causes	19,072,608,838,100<br>(18,149,086,500,500–20,087,721,490,400)	22,795,928,244,900<br>(21,457,458,988,100–24,128,236,127,000)	23,761,899,415,800<br>(22,289,755,000,300–25,372,248,142,500)	22,076,225,914,000<br>(20,520,217,830,600–23,775,565,838,400)
Western Sub-Saharan Africa	Group I	14,780,119,174,700<br>(13,965,894,668,700–15,635,862,486,600)	17,540,720,425,500<br>(16,323,538,821,800–18,793,864,636,300)	17,982,923,976,000<br>(16,686,534,150,200–19,436,638,914,200)	15,061,539,154,700<br>(13,838,153,658,800–16,421,577,925,800)
Western Sub-Saharan Africa	NCD	3,447,791,896,220<br>(2,885,293,052,140–4,011,215,028,580)	4,272,854,875,050<br>(3,541,638,133,890–5,004,064,355,970)	4,765,008,569,190<br>(3,957,666,452,430–5,516,287,902,420)	5,831,070,227,750<br>(4,926,554,946,580–6,717,024,261,040)
Western Sub-Saharan Africa	Injuries	844,697,767,208<br>(691,879,416,342–989,500,917,313)	982,352,944,283<br>(839,938,923,290–1,118,389,772,600)	1,013,966,870,580<br>(899,590,714,474–1,136,740,233,080)	1,183,616,531,500<br>(1,068,261,803,990–1,318,824,081,700)
Benin	All causes	464,704,625,131<br>(433,444,311,389–499,367,893,048)	527,411,185,555<br>(487,182,894,963–570,705,418,359)	553,026,995,882<br>(512,888,146,436–602,346,740,618)	550,241,296,734<br>(494,291,770,845–617,289,227,201)
Benin	Group I	342,164,282,350<br>(312,850,276,124–371,574,428,909)	371,452,713,353<br>(337,424,587,804–407,869,123,756)	382,379,137,690<br>(349,004,128,320–421,425,596,958)	336,892,730,019<br>(293,147,269,340–384,931,198,490)
Benin	NCD	98,461,945,347<br>(83,563,127,994–113,685,901,016)	126,566,234,114<br>(106,782,663,903–145,886,536,338)	140,600,066,672<br>(120,583,642,814–161,347,638,264)	178,521,255,341<br>(155,125,810,260–203,755,627,532)
Benin	Injuries	24,078,397,434<br>(19,480,614,151–29,454,064,233)	29,392,238,088<br>(25,139,155,640–34,032,545,971)	30,047,791,520<br>(26,795,388,044–33,962,707,098)	34,827,311,373<br>(30,438,270,750–39,513,181,030)
Burkina Faso	All causes	1,036,366,420,590<br>(903,880,819,316–1,153,012,139,630)	1,174,959,983,270<br>(1,020,071,793,240–1,312,732,221,160)	1,212,455,685,730<br>(1,021,538,604,750–1,389,920,332,400)	1,156,798,416,570<br>(946,027,670,098–1,366,205,674,940)
Burkina Faso	Group I	821,220,905,195<br>(705,337,348,788–924,196,421,846)	918,799,367,079<br>(777,187,900,530–1,049,717,077,170)	920,556,209,240<br>(758,024,253,605–1,085,726,779,410)	783,497,582,740<br>(605,651,574,298–956,155,252,981)
Burkina Faso	NCD	169,172,046,274<br>(141,404,035,630–199,059,097,500)	206,261,563,910<br>(171,905,441,705–240,583,091,566)	238,731,339,215<br>(195,491,331,060–276,562,530,431)	306,567,530,724<br>(256,053,215,400–356,315,477,941)
Burkina Faso	Injuries	45,973,469,120<br>(37,632,380,330–56,002,274,745)	49,899,052,280<br>(42,521,894,100–59,212,391,923)	53,168,137,280<br>(46,010,016,177–61,164,624,407)	66,733,303,110<br>(57,594,140,120–77,281,108,928)
Cameroon	All causes	896,689,543,685<br>(841,281,225,487–961,766,093,683)	1,251,001,937,240<br>(1,152,336,844,930–1,371,507,530,750)	1,378,866,848,770<br>(1,260,856,342,960–1,509,886,703,970)	1,394,704,726,990<br>(1,221,776,104,820–1,593,098,676,220)
Cameroon	Group I	643,351,187,600<br>(597,702,079,686–693,582,033,648)	907,909,588,964<br>(825,506,568,815–1,004,413,588,220)	986,122,370,830<br>(888,906,510,793–1,089,306,788,900)	891,671,707,807<br>(759,777,972,930–1,049,500,961,680)
Cameroon	NCD	207,567,305,369<br>(179,699,183,636–237,036,052,718)	282,264,563,808<br>(237,741,191,004–325,816,300,534)	323,564,617,552<br>(273,145,602,881–375,219,465,993)	412,611,227,039<br>(344,690,624,380–483,408,989,109)
Cameroon	Injuries	45,771,050,716<br>(38,875,594,565–52,968,988,499)	60,827,784,468<br>(51,839,617,511–70,174,899,696)	69,179,860,389<br>(59,835,241,534–79,186,241,797)	90,421,792,142<br>(77,060,270,211–105,928,497,592)
Cape Verde	All causes	14,363,868,530<br>(13,236,897,110–15,588,404,168)	14,961,895,295<br>(13,333,355,594–16,695,085,528)	14,872,238,690<br>(13,153,004,420–16,769,791,366)	14,048,031,937<br>(12,390,803,628–15,946,097,874)
Cape Verde	Group I	7,547,525,990<br>(6,907,573,305–8,205,207,678)	6,554,285,632<br>(5,720,972,705–7,486,746,910)	5,567,813,243<br>(4,845,983,491–6,382,762,723)	3,957,272,569<br>(3,449,892,388–4,518,301,774)
Cape Verde	NCD	5,718,378,548<br>(5,047,913,351–6,423,499,221)	6,808,939,621<br>(5,788,117,166–7,850,464,371)	7,552,204,543<br>(6,432,689,868–8,683,655,384)	8,266,908,595<br>(7,033,881,207–9,546,253,455)
Cape Verde	Injuries	1,097,963,993<br>(946,517,249–1,257,543,969)	1,598,670,043<br>(1,335,477,124–1,885,503,176)	1,752,220,904<br>(1,458,765,945–2,051,589,145)	1,823,850,773<br>(1,489,092,022–2,127,878,047)
Chad	All causes	623,843,069,664<br>(590,462,088,745–656,025,236,326)	826,546,603,474<br>(777,879,058,266–876,919,729,440)	930,788,236,307<br>(867,306,299,708–996,844,833,516)	993,516,044,831<br>(897,947,849,873–1,100,068,208,110)
Chad	Group I	492,369,345,818<br>(463,512,974,020–523,413,321,711)	649,487,547,406<br>(605,481,647,917–695,814,430,120)	712,614,888,593<br>(655,812,045,493–771,056,901,399)	718,105,600,662<br>(640,473,744,079–814,753,386,963)
Chad	NCD	100,028,892,053<br>(83,961,323,380–118,394,103,183)	134,116,098,590<br>(110,007,929,517–157,894,363,743)	161,391,388,481<br>(133,010,897,321–191,277,414,692)	219,328,293,106<br>(183,205,684,760–256,005,007,448)
Chad	Injuries	31,444,831,792<br>(24,215,335,078–38,853,778,969)	42,942,957,479<br>(36,064,428,833–49,976,524,899)	56,781,959,233<br>(45,758,647,151–69,179,568,580)	56,082,151,062<br>(48,693,402,033–65,286,740,519)
Cote d'Ivoire	All causes	1,013,062,958,020<br>(916,416,743,948–1,097,802,354,930)	1,346,229,179,250<br>(1,230,435,819,340–1,471,886,387,670)	1,398,066,103,640<br>(1,237,315,972,780–1,560,598,183,130)	1,323,359,886,460<br>(1,158,102,116,100–1,515,094,185,070)

Appendix Table 3. GBD 2016 DALY counts for all causes and level 1 cause groups by location for 1990, 2000, 2006 & 2016, both sexes combined.

	Cause	1990	2000	2006	2016
Cote d'Ivoire	Group I	733,768,144,570 (653,440,804,243–805,941,936,953)	964,815,719,709 (875,132,732,862–1,072,834,377,380)	990,273,687,537 (852,742,590,821–1,136,310,018,040)	820,886,497,191 (689,216,941,624–968,402,060,374)
Cote d'Ivoire	NCD	227,796,263,562 (199,586,286,832–257,993,162,569)	311,591,298,858 (268,572,464,240–358,936,217,049)	338,694,995,064 (287,046,005,702–387,976,917,158)	416,365,145,636 (358,526,501,007–481,873,271,133)
Cote d'Ivoire	Injuries	51,498,549,884 (44,414,192,306–59,855,221,300)	69,822,160,684 (61,206,919,305–80,047,798,633)	69,097,421,035 (61,004,790,114–78,514,596,542)	86,108,243,635 (75,420,563,309–98,202,122,498)
The Gambia	All causes	64,070,784,590 (57,445,173,810–71,009,704,414)	72,831,144,707 (65,878,201,791–79,872,172,882)	75,441,668,649 (67,611,038,753–83,717,717,391)	78,661,011,529 (69,563,774,804–88,084,607,962)
The Gambia	Group I	46,563,943,668 (40,876,451,038–52,149,776,304)	51,034,485,680 (45,216,603,933–56,676,113,327)	49,864,257,786 (43,831,085,946–56,460,406,440)	46,683,582,638 (40,451,972,667–53,717,145,581)
The Gambia	NCD	14,498,539,723 (12,433,405,598–16,523,666,925)	18,172,613,685 (15,230,907,057–20,896,890,408)	21,490,774,054 (18,088,754,353–24,703,367,206)	27,330,589,788 (23,348,691,286–31,622,207,213)
The Gambia	Injuries	3,008,301,199 (2,519,708,175–3,552,783,135)	3,624,045,342 (3,157,839,614–4,123,215,638)	4,086,636,809 (3,594,315,781–4,639,337,403)	4,646,839,103 (4,019,218,160–5,345,396,495)
Ghana	All causes	1,003,845,260,840 (924,440,260,188–1,084,084,906,150)	1,106,017,314,490 (1,004,865,411,150–1,206,336,239,130)	1,232,023,984,790 (1,133,833,783,770–1,333,177,810,240)	1,137,097,054,280 (1,021,222,194,540–1,264,919,533,630)
Ghana	Group I	731,174,238,309 (669,565,520,856–795,296,457,624)	747,997,159,670 (665,804,212,393–826,260,428,208)	802,452,708,636 (728,305,461,534–878,696,733,925)	621,736,846,837 (542,597,033,838–713,043,308,206)
Ghana	NCD	225,597,444,405 (196,835,153,914–256,553,139,796)	302,026,770,523 (264,325,471,721–342,031,403,131)	362,607,721,330 (317,139,231,494–408,771,816,069)	438,868,473,317 (378,297,270,686–500,058,790,996)
Ghana	Injuries	47,073,578,131 (39,499,649,142–56,286,309,522)	55,993,384,292 (48,678,268,286–64,412,488,245)	66,963,554,827 (59,954,732,147–75,338,848,016)	76,491,734,128 (66,922,565,226–87,290,654,710)
Guinea	All causes	643,675,101,848 (610,851,222,398–676,977,706,950)	773,360,027,263 (728,101,506,054–820,431,732,559)	771,678,528,038 (717,353,273,738–831,741,241,276)	756,998,631,420 (678,148,256,690–847,503,497,804)
Guinea	Group I	491,169,632,782 (457,367,959,867–524,283,588,067)	568,947,805,302 (529,194,663,953–615,006,714,059)	552,418,788,556 (508,501,777,658–607,205,703,644)	489,942,664,827 (430,277,211,369–567,853,846,134)
Guinea	NCD	123,572,503,840 (102,383,741,242–144,790,695,375)	167,226,085,619 (138,494,745,543–192,457,374,709)	182,491,446,212 (153,778,084,093–209,496,990,866)	225,944,734,447 (192,430,302,092–258,177,514,572)
Guinea	Injuries	28,932,965,226 (22,187,242,503–35,759,396,023)	37,186,136,342 (31,009,161,626–43,551,833,248)	36,768,293,270 (31,856,650,217–41,971,970,044)	41,111,232,146 (35,559,450,860–46,706,371,057)
Guinea-Bissau	All causes	115,176,089,480 (107,972,524,235–122,642,265,179)	117,244,670,339 (110,049,201,975–125,315,681,651)	119,532,826,276 (111,206,453,434–128,586,936,299)	109,626,139,608 (100,469,021,193–119,607,329,961)
Guinea-Bissau	Group I	84,105,766,206 (77,813,618,047–90,774,477,867)	81,041,846,013 (74,921,003,874–87,554,573,647)	80,299,472,547 (73,823,638,483–87,234,783,010)	64,900,871,706 (58,728,301,749–71,386,332,207)
Guinea-Bissau	NCD	24,913,371,486 (21,483,530,489–28,848,784,974)	29,331,925,444 (25,121,688,825–33,127,318,156)	31,635,433,847 (27,391,440,212–35,792,045,131)	37,024,187,043 (32,456,006,339–41,733,596,546)
Guinea-Bissau	Injuries	6,156,951,788 (5,013,829,179–7,283,132,989)	6,870,898,882 (5,998,677,934–7,810,762,583)	7,597,919,882 (6,566,835,525–8,644,168,327)	7,701,080,859 (6,688,612,063–8,792,430,225)
Liberia	All causes	263,694,419,009 (246,552,401,235–280,926,213,337)	269,651,560,659 (252,796,332,869–288,595,419,323)	236,831,757,914 (221,286,501,385–254,227,685,670)	211,895,895,303 (192,068,311,582–233,649,715,009)
Liberia	Group I	187,622,025,002 (171,816,770,013–204,894,660,088)	208,858,337,096 (193,721,251,601–225,725,170,205)	174,475,333,192 (161,511,113,232–188,753,532,407)	131,666,364,906 (117,211,456,025–148,100,036,281)
Liberia	NCD	42,505,659,349 (34,724,034,864–49,786,781,400)	50,272,753,654 (41,077,728,917–58,563,530,226)	52,790,856,091 (44,685,495,368–60,803,617,379)	68,690,949,952 (59,368,045,573–78,453,443,920)
Liberia	Injuries	33,566,734,658 (29,347,452,736–37,982,149,390)	10,520,469,909 (8,884,187,302–12,297,284,609)	9,565,568,631 (8,298,100,819–10,960,559,957)	11,538,580,445 (9,983,590,871–13,151,681,417)
Mali	All causes	1,082,500,771,270 (1,034,056,061,110–1,131,237,819,820)	1,174,618,605,460 (1,109,600,765,300–1,248,571,509,170)	1,177,810,577,090 (1,099,733,148,640–1,262,951,820,270)	1,162,347,153,310 (1,023,953,443,930–1,322,344,822,490)
Mali	Group I	861,113,721,374 (809,493,174,300–915,941,840,069)	920,009,220,206 (855,574,319,728–985,682,638,547)	912,979,109,719 (838,073,287,768–995,472,021,890)	845,834,162,864 (726,390,795,791–993,972,966,554)
Mali	NCD	175,405,822,564 (142,822,971,224–209,365,074,220)	195,543,242,905 (161,593,236,769–230,570,791,199)	215,889,005,271 (175,435,284,061–252,871,630,194)	262,893,095,374 (214,602,808,837–307,823,404,693)
Mali	Injuries	45,981,227,327 (34,049,583,463–56,013,075,936)	59,066,142,350 (45,303,458,039–73,813,854,384)	48,942,462,097 (39,716,426,863–57,850,183,259)	53,619,895,068 (44,986,162,309–63,555,960,958)
Mauritania	All causes	120,494,957,126 (112,320,310,235–128,861,665,919)	132,225,801,667 (123,160,960,332–142,570,402,280)	137,748,792,747 (126,006,000,615–150,984,590,113)	131,296,215,128 (116,720,883,914–147,133,892,225)
Mauritania	Group I	81,940,914,217 (76,515,597,397–87,362,752,125)	85,350,824,180 (78,784,465,969–92,927,585,972)	83,073,136,690 (74,903,073,924–92,226,103,726)	64,990,098,644 (57,159,166,245–74,757,281,813)
Mauritania	NCD	31,211,299,650 (26,793,426,421–35,678,181,569)	39,681,309,859 (33,061,131,459–46,186,482,839)	46,618,178,052 (38,206,845,851–53,863,108,115)	57,660,086,700 (47,337,944,391–67,246,907,693)
Mauritania	Injuries	7,342,743,259 (5,636,766,259–9,085,780,383)	7,193,667,628 (5,870,718,939–8,235,730,848)	8,057,478,005 (6,626,649,450–9,410,561,423)	8,646,029,783 (6,919,350,097–10,576,728,098)
Niger	All causes	1,197,587,091,090 (1,133,149,563,150–1,263,410,292,920)	1,318,461,442,320 (1,237,030,313,100–1,403,621,112,350)	1,274,294,625,160 (1,175,689,211,440–1,374,014,779,000)	1,279,367,997,590 (1,129,035,690,680–1,458,489,867,310)
Niger	Group I	1,000,087,690,940 (932,265,533,667–1,070,253,252,490)	1,088,103,194,910 (1,005,817,445,530–1,175,126,130,610)	1,002,927,185,620 (913,668,151,783–1,100,940,642,250)	925,090,872,662 (793,404,823,976–1,077,969,357,390)
Niger	NCD	149,134,732,227 (116,317,186,995–181,967,771,211)	180,431,486,254 (144,208,613,957–217,881,205,757)	216,832,892,905 (177,593,715,548–257,293,776,899)	291,968,557,888 (242,035,726,312–341,951,108,255)
Niger	Injuries	48,364,667,928 (32,655,713,147–61,635,419,984)	49,926,761,151 (37,578,584,364–61,825,803,328)	54,534,546,631 (44,481,364,964–65,742,720,036)	62,308,567,040 (53,266,125,602–73,138,970,618)
Nigeria	All causes	9,222,044,622,470 (8,443,099,316,690–10,026,009,032,900)	11,304,076,197,600 (10,162,717,414,200–12,440,359,796,600)	11,768,973,804,500 (10,535,498,280,700–13,085,744,516,000)	10,393,898,523,300 (9,164,610,240,250–11,763,479,201,900)
Nigeria	Group I	7,292,314,214,420 (6,618,686,219,050–8,048,897,677,940)	8,981,475,835,480 (7,922,287,018,570–10,012,352,227,600)	9,315,438,759,320 (8,220,471,502,010–10,532,344,292,100)	7,497,840,363,360 (6,410,642,945,910–8,626,074,938,690)
Nigeria	NCD	1,569,484,243,200 (1,271,278,930,480–1,884,780,503,350)	1,899,771,706,060 (1,483,356,177,870–2,324,282,975,770)	2,036,297,734,200 (1,604,946,314,760–2,463,781,514,530)	2,403,141,494,710 (1,910,090,687,480–2,877,953,252,790)
Nigeria	Injuries	360,246,164,846 (280,652,749,972–442,063,171,097)	422,828,656,043 (339,121,494,393–509,842,797,827)	417,237,310,964 (343,214,849,563–493,002,681,099)	492,916,665,203 (418,598,266,599–581,865,664,838)
Sao Tome and Principe	All causes	6,798,085,398 (6,358,537,049–7,285,701,208)	6,773,493,270 (6,240,232,920–7,330,257,708)	6,264,458,172 (5,738,861,248–6,865,086,673)	5,702,419,093 (5,072,825,158–6,434,065,709)
Sao Tome and Principe	Group I	4,140,367,107 (3,822,928,865–4,476,021,307)	3,853,267,494 (3,442,961,294–4,371,209,277)	3,232,085,472 (2,846,888,158–3,772,146,612)	2,298,064,510 (1,985,161,993–2,729,555,642)
Sao Tome and Principe	NCD	2,273,474,633 (2,009,559,867–2,538,162,643)	2,543,094,910 (2,167,973,387–2,870,521,593)	2,638,329,087 (2,210,827,660–3,008,183,078)	2,967,582,908 (2,559,400,255–3,435,371,014)
Sao Tome and Principe	Injuries	384,243,659 (330,511,014–440,430,377)	377,130,867 (304,932,984–443,765,757)	394,043,613 (292,046,591–473,934,155)	436,771,675 (351,852,222–528,736,234)
Senegal	All causes	567,547,814,887 (535,424,699,003–600,979,860,824)	614,354,473,042 (580,475,398,757–653,352,089,351)	620,830,525,090 (578,844,997,727–666,619,013,560)	596,210,826,750 (545,838,094,566–656,404,608,439)
Senegal	Group I	412,175,573,779 (385,275,892,120–438,762,417,338)	422,319,678,089 (395,827,645,902–451,386,124,275)	388,889,238,136 (358,058,089,440–421,022,817,787)	317,883,204,114 (289,755,204,174–354,611,496,790)
Senegal	NCD	125,979,553,558 (109,869,326,636–143,210,896,307)	158,849,556,878 (137,305,495,266–180,283,300,235)	190,879,327,261 (164,866,741,502–214,710,105,621)	234,383,505,119 (203,700,871,741–266,228,069,949)
Senegal	Injuries	29,392,687,550 (25,247,199,132–33,801,769,802)	33,185,238,076 (29,384,591,075–37,383,523,090)	41,061,959,692 (36,719,364,163–45,747,011,304)	43,944,117,517 (38,692,198,621–50,094,867,746)
Sierra Leone	All causes	443,956,566,084 (421,002,452,429–466,754,106,098)	417,569,143,799 (396,052,529,303–439,234,114,858)	472,350,783,005 (445,576,708,267–500,333,214,917)	428,195,263,556 (393,208,423,509–466,945,153,984)
Sierra Leone	Group I	338,288,306,595 (314,122,838,576–365,985,138,056)	314,948,368,849 (292,635,401,167–338,204,245,820)	352,868,790,467 (325,836,448,139–384,806,181,869)	286,178,352,428 (255,590,494,001–322,921,428,774)
Sierra Leone	NCD	86,354,812,646 (69,202,236,517–102,535,403,835)	79,199,771,598 (62,722,652,380–93,972,854,568)	100,089,835,995 (79,619,915,367–116,749,607,118)	120,109,274,360 (99,181,736,376–139,683,284,057)
Sierra Leone	Injuries	19,313,446,843 (14,292,127,666–23,608,290,654)	23,421,003,352 (18,556,525,388–28,627,321,080)	19,392,156,543 (16,130,468,519–22,857,060,782)	21,907,636,769 (18,788,790,434–25,604,088,567)
Togo	All causes	292,122,690,318 (266,828,084,723–318,628,265,413)	347,571,299,953 (310,035,389,123–386,106,863,482)	379,986,302,366 (331,007,892,340–433,084,902,699)	352,206,675,070 (313,311,024,137–396,104,723,633)
Togo	Group I	208,982,178,675 (187,357,014,746–231,657,989,052)	247,743,569,695 (216,245,142,159–280,515,317,555)	266,477,672,033 (223,327,363,306–313,377,864,590)	211,472,467,514 (182,077,190,634–242,979,864,056)
Togo	NCD	68,074,155,761 (58,980,339,863–77,498,286,883)	82,154,445,506 (69,860,436,565–94,055,244,052)	94,174,171,898 (79,830,704,998–107,796,768,493)	118,386,850,381 (102,691,288,356–134,986,184,472)
Togo	Injuries	15,066,355,882 (12,936,967,056–17,389,489,056)	17,673,284,753 (15,285,998,317–20,286,176,254)	19,334,458,435 (16,815,859,754–22,250,346,780)	22,347,357,175 (19,763,945,368–25,362,618,536)
Eastern Sub-Saharan Africa	All causes	18,580,321,818,200 (17,928,654,274,400–19,245,655,829,700)	21,203,517,608,500 (20,382,746,000,800–22,057,650,393,700)	20,216,170,289,200 (19,322,520,392,600–21,161,721,177,300)	17,574,196,428,300 (16,448,352,675,600–18,764,841,933,700)
Eastern Sub-Saharan Africa	Group I	14,168,415,445,900 (13,710,835,194,500–14,595,672,989,800)	16,143,528,348,500 (15,641,974,348,600–16,612,070,648,900)	15,008,930,364,000 (14,515,115,177,300–15,517,926,187,700)	11,078,666,349,900 (10,551,125,722,200–11,624,560,546,300)
Eastern Sub-Saharan Africa	NCD	3,103,940,097,100 (2,755,822,315,070–3,481,182,892,100)	3,685,827,609,430 (3,248,546,178,190–4,163,212,327,500)	4,101,540,571,470 (3,608,176,397,420–4,653,252,566,630)	5,220,213,544,080 (4,544,067,259,950–5,982,680,952,310)
Eastern Sub-Saharan Africa	Injuries	1,307,966,275,130 (1,156,829,890,490–1,469,340,313,420)	1,374,161,650,630 (1,255,717,781,230–1,500,465,162,370)	1,105,699,353,710 (1,006,101,735,150–1,213,615,217,270)	1,275,316,534,380 (1,156,697,955,410–1,396,956,207,640)
Burundi	All causes	617,120,848,709 (568,029,973,051–671,540,084,799)	665,220,047,934 (613,346,536,114–718,730,775,310)	619,734,692,541 (570,190,299,839–674,713,707,494)	655,288,894,871 (545,680,481,573–799,738,192,569)
Burundi	Group I	472,718,477,831 (432,668,642,671–518,172,593,278)	509,537,616,446 (465,226,613,217–556,362,254,908)	463,186,918,192 (422,769,539,959–509,608,240,878)	447,066,113,585 (350,800,610,272–586,517,376,187)
Burundi	NCD	113,984,182,682 (100,012,178,151–128,877,711,794)	112,280,472,250 (98,444,171,012–127,198,134,067)	122,416,493,896 (105,912,608,832–139,726,880,609)	163,694,841,311 (139,855,252,858–191,005,019,073)
Burundi	Injuries	30,418,188,195 (25,296,857,583–36,029,031,463)	43,401,959,238 (35,996,407,914–51,376,488,857)	34,131,280,454 (29,793,467,710–39,006,155,970)	44,527,939,975 (37,725,231,159–52,411,340,284)
Comoros	All causes	26,837,268,467 (24,785,484,036–29,023,918,849)	29,056,915,282 (26,640,093,793–31,633,916,468)	28,311,155,473 (25,586,206,379–31,345,939,383)	26,364,470,574 (23,136,072,118–29,946,934,901)
Comoros	Group I	18,152,310,653 (16,498,330,160–19,672,549,552)	19,352,896,614 (17,511,323,219–21,425,649,446)	17,450,495,227 (15,459,281,785–19,830,976,926)	13,546,516,355 (11,395,921,410–16,098,683,675)
Comoros	NCD	6,922,885,952 (5,996,410,299–7,874,074,677)	7,940,192,142 (6,912,590,166–9,080,283,543)	8,626,899,749 (7,438,055,604–9,984,727,467)	10,642,038,637 (9,073,318,779–12,428,189,973)
Comoros	Injuries	1,762,071,863 (1,471,624,081–2,078,474,136)	1,763,826,527 (1,542,748,695–1,993,927,068)	2,233,760,497 (1,954,258,323–2,529,274,134)	2,175,915,582 (1,862,761,705–2,552,374,859)
Djibouti	All causes	41,115,131,038 (36,499,399,573–45,592,093,280)	48,997,011,772 (43,237,159,259–55,069,420,334)	48,744,374,389 (42,146,580,968–55,769,511,734)	40,434,741,473 (33,666,115,153–48,604,266,694)
Djibouti	Group I	28,678,010,492 (24,851,686,703–32,497,345,959)	34,803,865,767 (30,232,267,339–39,750,450,370)	32,798,499,183 (27,474,047,354–38,555,997,552)	23,150,896,866 (18,136,818,431–29,810,291,113)
Djibouti	NCD	9,303,399,884 (8,052,085,059–10,705,891,899)	11,108,538,830 (9,448,261,477–12,847,932,304)	12,149,462,366 (10,200,623,379–14,241,587,396)	14,286,852,666 (11,841,176,152–16,742,495,132)
Djibouti	Injuries	3,133,720,662 (2,571,151,852–3,790,305,133)	3,084,607,175 (2,528,848,286–3,722,456,391)	3,796,412,839 (3,082,854,213–4,515,796,522)	2,996,991,941 (2,489,326,284–3,636,188,443)

Appendix Table 3. GBD 2016 DALY counts for all causes and level 1 cause groups by location for 1990, 2000, 2006 & 2016, both sexes combined.

	Cause	1990	2000	2006	2016
Eritrea	All causes	273,743,761,219 (251,532,744,900–296,999,351,328)	208,511,408,648 (189,592,470,078–229,503,611,591)	221,074,382,897 (196,628,359,193–247,792,960,718)	211,813,093,627 (186,019,735,076–240,923,367,229)
Eritrea	Group I	209,472,022,109 (189,811,872,891–229,987,289,166)	133,751,489,105 (118,609,555,887–149,692,643,977)	144,503,667,260 (124,949,962,206–166,520,759,324)	120,354,672,616 (102,595,873,206–141,272,490,956)
Eritrea	NCD	50,183,827,996 (43,382,532,196–56,936,012,240)	48,755,022,392 (41,806,405,403–55,961,203,704)	59,334,688,962 (50,695,333,380–68,400,647,420)	72,835,190,966 (61,820,625,988–84,108,612,577)
Eritrea	Injuries	14,087,911,114 (11,787,896,125–16,640,963,356)	26,004,897,151 (23,847,500,108–28,168,108,747)	17,236,026,675 (15,087,385,461–19,660,714,542)	18,623,230,045 (16,030,562,778–21,265,165,803)
Ethiopia	All causes	5,327,302,681,460 (5,075,682,037,920–5,585,552,426,080)	5,622,946,831,730 (5,322,532,229,960–5,946,711,863,260)	4,903,524,825,510 (4,556,197,384,280–5,272,088,058,940)	3,815,751,422,250 (3,394,012,064,870–4,230,415,110,300)
Ethiopia	Group I	3,825,189,689,520 (3,602,871,283,310–4,063,889,115,530)	3,946,331,084,370 (3,698,002,062,440–4,211,209,154,960)	3,429,857,406,960 (3,187,978,886,100–3,694,177,711,570)	2,055,360,120,550 (1,835,757,549,220–2,309,596,993,870)
Ethiopia	NCD	914,339,486,160 (807,317,111,866–1,034,475,348,480)	1,069,158,271,960 (938,536,971,952–1,210,330,286,880)	1,162,151,517,040 (1,013,603,751,520–1,328,181,233,060)	1,434,665,912,020 (1,217,521,045,720–1,686,864,224,650)
Ethiopia	Injuries	587,773,505,786 (525,252,220,367–654,974,242,122)	607,457,475,402 (556,450,258,448–659,620,240,934)	311,515,901,505 (273,582,250,247–353,818,140,590)	325,725,389,683 (274,648,521,533–380,706,006,486)
Kenya	All causes	1,350,691,607,160 (1,292,808,674,510–1,415,612,125,650)	2,065,386,441,880 (1,970,586,554,460–2,163,281,349,770)	2,007,957,541,070 (1,914,605,426,720–2,110,872,619,710)	1,664,140,434,460 (1,553,031,891,300–1,799,026,466,690)
Kenya	Group I	1,044,201,932,670 (1,005,864,242,930–1,079,238,661,870)	1,650,100,777,140 (1,592,726,886,260–1,707,940,786,770)	1,533,004,089,170 (1,479,667,754,030–1,587,359,169,840)	1,053,404,106,280 (997,312,140,720–1,113,832,780,110)
Kenya	NCD	240,803,298,989 (199,375,010,425–285,107,148,897)	330,670,690,712 (276,859,068,038–392,849,064,989)	379,870,130,806 (316,491,228,402–451,094,726,988)	498,101,396,742 (413,296,213,818–592,198,361,942)
Kenya	Injuries	65,686,375,505 (54,812,580,960–76,561,105,612)	84,614,974,034 (72,402,771,381–97,041,695,875)	95,083,321,094 (82,176,004,411–108,907,786,272)	112,634,931,438 (96,405,657,180–130,225,700,010)
Madagascar	All causes	1,010,599,745,470 (939,116,866,682–1,086,117,929,550)	1,108,730,178,200 (1,014,726,691,930–1,210,884,991,470)	1,143,514,611,390 (1,035,692,154,980–1,252,044,495,620)	1,250,104,547,560 (1,084,101,196,590–1,432,611,417,840)
Madagascar	Group I	739,672,051,520 (680,143,531,643–801,655,861,851)	778,544,465,667 (706,044,558,148–858,120,467,030)	775,184,391,588 (688,492,495,220–858,568,187,636)	764,775,941,040 (641,914,766,903–919,724,415,352)
Madagascar	NCD	224,711,625,099 (200,297,741,807–249,342,860,528)	278,064,375,158 (245,613,083,102–311,876,140,690)	314,648,372,698 (272,811,635,151–356,712,634,402)	415,476,016,722 (350,836,304,036–477,209,122,740)
Madagascar	Injuries	46,216,068,851 (37,878,445,225–54,566,173,473)	52,121,337,376 (45,044,098,804–59,763,400,326)	53,681,847,106 (46,005,142,712–61,410,901,913)	69,852,589,802 (57,991,790,753–81,644,630,255)
Malawi	All causes	1,191,340,973,030 (1,114,887,420,900–1,284,178,719,050)	1,299,863,103,810 (1,181,528,145,430–1,429,042,897,180)	1,218,169,377,850 (1,097,426,385,590–1,335,488,742,990)	998,739,772,835 (881,510,776,118–1,135,991,756,790)
Malawi	Group I	976,712,091,372 (907,001,696,301–1,060,766,007,100)	1,062,264,279,100 (963,550,199,166–1,174,208,199,940)	969,718,867,602 (874,503,629,206–1,070,027,579,490)	690,491,010,066 (598,821,821,129–802,915,186,985)
Malawi	NCD	162,517,443,745 (135,523,151,041–189,048,183,523)	186,750,384,349 (147,592,142,266–222,575,276,870)	202,326,203,881 (162,000,732,166–239,857,617,657)	255,943,474,240 (213,704,361,730–297,950,981,753)
Malawi	Injuries	52,111,437,911 (38,522,356,861–65,937,064,262)	50,848,440,359 (40,498,999,215–61,504,395,596)	46,124,306,368 (37,318,422,423–54,141,303,913)	52,305,288,530 (43,692,853,498–61,764,545,488)
Mozambique	All causes	1,461,337,679,770 (1,301,994,552,160–1,611,337,832,810)	1,770,853,639,580 (1,621,038,099,060–1,931,244,181,520)	1,869,447,885,450 (1,698,801,168,380–2,056,507,214,000)	1,684,362,951,300 (1,499,001,223,160–1,876,897,692,860)
Mozambique	Group I	1,147,412,129,260 (1,005,586,514,750–1,286,478,090,700)	1,400,633,357,680 (1,274,611,545,950–1,543,697,246,820)	1,475,828,077,740 (1,336,837,936,780–1,635,708,668,280)	1,195,225,232,290 (1,058,611,395,900–1,350,111,603,370)
Mozambique	NCD	229,570,862,935 (199,992,651,968–261,158,885,671)	280,325,312,449 (233,488,550,162–329,409,672,327)	304,483,347,253 (253,152,861,635–363,937,645,523)	385,733,499,186 (325,400,281,045–447,642,549,655)
Mozambique	Injuries	84,354,687,576 (68,155,152,874–102,054,340,764)	89,894,969,458 (74,669,705,633–106,513,488,847)	89,136,460,455 (73,735,855,595–106,567,831,244)	103,404,219,818 (87,390,780,812–122,890,555,234)
Rwanda	All causes	762,760,964,585 (714,059,362,617–820,156,477,932)	763,905,132,537 (701,312,657,277–827,724,942,490)	586,270,116,578 (541,240,966,552–639,724,862,396)	431,424,480,259 (386,633,137,354–489,126,569,525)
Rwanda	Group I	587,364,129,619 (547,007,407,953–634,473,290,677)	594,185,837,928 (539,830,023,564–651,198,352,300)	421,806,234,417 (385,268,227,720–462,041,306,967)	241,955,735,213 (208,222,755,248–284,547,492,044)
Rwanda	NCD	119,815,558,226 (103,889,985,475–136,873,952,238)	125,184,424,917 (108,258,164,560–143,003,424,709)	123,023,504,926 (105,998,183,707–142,294,211,719)	149,495,245,319 (126,744,904,115–175,348,216,828)
Rwanda	Injuries	55,581,276,739 (42,706,401,629–68,548,387,742)	44,534,869,692 (36,870,793,870–53,269,973,748)	41,440,377,235 (35,743,804,679–48,352,977,355)	39,973,499,728 (34,042,571,775–47,333,097,650)
Somalia	All causes	580,374,474,678 (494,012,752,732–669,747,118,659)	565,040,620,563 (487,195,502,127–646,198,687,474)	576,463,166,813 (506,829,402,709–647,696,648,348)	599,705,760,841 (521,578,265,423–690,182,555,964)
Somalia	Group I	411,660,876,046 (335,175,253,831–493,115,985,905)	408,228,075,474 (343,026,131,463–482,618,308,507)	397,851,277,998 (338,069,535,235–460,758,140,420)	374,437,003,266 (308,365,272,493–447,350,049,740)
Somalia	NCD	118,978,045,316 (98,303,918,066–137,652,455,495)	121,108,774,588 (101,467,999,721–139,963,336,275)	133,773,974,819 (112,091,011,554–155,760,458,790)	164,288,320,393 (141,439,739,109–191,299,744,470)
Somalia	Injuries	49,735,553,316 (39,116,186,573–62,035,330,302)	35,703,770,501 (29,121,278,909–43,460,301,989)	44,837,913,996 (36,949,078,877–53,123,299,136)	60,980,437,183 (50,132,495,564–71,725,482,244)
South Sudan	All causes	758,758,351,896 (667,427,042,853–853,181,444,345)	667,331,494,668 (600,459,584,231–733,487,661,810)	739,846,813,815 (658,521,723,108–826,256,416,716)	942,684,641,706 (800,614,600,596–1,115,269,901,190)
South Sudan	Group I	613,317,585,040 (532,606,536,464–697,955,268,406)	533,626,145,859 (479,019,846,122–588,756,703,930)	586,776,781,339 (519,208,797,518–663,813,750,087)	708,930,809,004 (585,099,625,976–861,800,010,786)
South Sudan	NCD	89,086,044,353 (71,995,356,134–105,580,327,569)	93,829,555,250 (76,238,322,570–111,063,063,685)	117,216,562,957 (95,549,978,657–140,074,560,148)	180,043,327,619 (149,700,168,785–214,157,987,279)
South Sudan	Injuries	56,354,722,503 (36,087,479,426–77,116,891,680)	39,875,793,559 (29,183,916,639–50,874,993,172)	35,853,469,519 (28,880,360,403–43,803,003,604)	53,710,505,084 (43,807,519,650–65,263,472,144)
Tanzania	All causes	2,367,847,808,650 (2,197,451,651,480–2,537,670,669,800)	2,873,640,101,300 (2,659,223,690,170–3,091,043,364,810)	2,806,735,498,710 (2,584,471,275,850–3,028,644,526,570)	2,372,216,326,600 (2,133,192,219,280–2,639,983,096,360)
Tanzania	Group I	1,822,917,537,670 (1,680,269,220,300–1,969,726,098,410)	2,236,415,112,400 (2,068,620,656,750–2,424,483,879,010)	2,082,561,458,870 (1,889,235,087,400–2,263,725,845,330)	1,460,724,990,150 (1,290,921,071,080–1,643,414,796,830)
Tanzania	NCD	417,460,904,043 (360,927,247,412–477,669,607,558)	500,966,946,442 (426,216,727,294–575,416,566,349)	568,070,599,153 (485,112,678,742–656,778,483,028)	730,247,234,267 (625,228,041,285–847,845,950,108)
Tanzania	Injuries	127,469,366,941 (108,345,422,170–149,633,981,972)	136,258,042,463 (116,731,199,722–154,969,782,556)	156,103,440,690 (134,621,862,046–177,043,221,941)	181,244,102,183 (157,414,325,546–208,727,310,928)
Uganda	All causes	1,982,755,126,900 (1,871,020,030,430–2,123,650,975,900)	2,359,506,114,220 (2,222,613,978,960–2,503,832,242,850)	2,295,233,261,790 (2,164,944,751,670–2,440,636,384,020)	1,981,756,079,110 (1,817,332,694,820–2,184,357,125,000)
Uganda	Group I	1,609,844,919,320 (1,512,755,015,660–1,733,160,723,310)	1,914,168,501,790 (1,793,278,713,140–2,035,082,256,250)	1,789,660,300,610 (1,687,935,369,400–1,907,181,000,330)	1,338,728,023,800 (1,222,538,070,400–1,476,841,681,270)
Uganda	NCD	285,029,189,987 (246,035,497,466–322,193,473,899)	340,856,263,176 (299,639,825,074–388,045,601,831)	391,820,824,877 (340,206,596,996–446,845,752,834)	503,155,563,515 (431,762,904,780–578,776,193,589)
Uganda	Injuries	87,881,017,594 (72,637,981,446–104,092,038,116)	104,481,349,261 (90,590,791,349–119,208,419,177)	113,752,136,299 (98,331,309,325–130,913,131,941)	139,872,491,788 (121,613,404,118–157,167,229,687)
Zambia	All causes	826,840,043,318 (769,020,516,110–886,771,268,642)	1,153,059,579,270 (1,050,930,378,490–1,267,202,109,430)	1,149,378,116,820 (1,039,961,748,770–1,256,400,122,550)	897,108,563,653 (790,503,747,494–1,013,285,399,430)
Zambia	Group I	660,824,856,862 (610,498,973,376–716,160,081,142)	921,123,446,211 (823,558,985,894–1,027,216,824,420)	888,217,436,754 (797,995,739,280–987,983,752,951)	589,959,201,910 (515,761,976,316–679,678,382,768)
Zambia	NCD	120,671,034,237 (103,489,805,724–137,898,971,674)	177,909,019,286 (152,299,597,944–203,712,106,471)	200,490,258,087 (170,065,757,818–228,239,167,048)	240,002,630,752 (202,210,303,639–281,447,087,590)
Zambia	Injuries	45,344,152,219 (35,868,372,671–56,072,201,580)	54,027,113,777 (45,989,084,169–62,749,311,194)	60,670,421,978 (51,741,597,395–69,858,330,905)	67,146,730,990 (56,267,908,220–79,208,246,489)
Central Sub-Saharan Africa	All causes	5,055,692,759,560 (4,039,879,534,220–5,876,340,826,720)	6,419,131,985,110 (5,206,597,187,340–7,511,857,922,070)	6,627,865,354,210 (5,681,301,903,190–7,526,318,946,570)	6,359,987,317,720 (5,312,257,042,850–7,527,871,433,950)
Central Sub-Saharan Africa	Group I	3,842,377,207,230 (2,955,167,425,660–4,587,944,904,920)	4,881,181,496,350 (3,809,376,878,420–5,848,431,795,410)	4,884,301,356,800 (4,043,771,045,510–5,694,463,104,690)	4,168,044,674,110 (3,297,108,526,580–5,217,832,081,800)
Central Sub-Saharan Africa	NCD	890,683,103,476 (731,107,122,856–1,036,360,806,500)	1,139,785,620,890 (954,525,315,352–1,324,368,443,830)	1,326,755,759,620 (1,122,957,999,150–1,528,419,721,580)	1,710,180,678,770 (1,458,185,265,680–1,969,237,746,600)
Central Sub-Saharan Africa	Injuries	322,632,448,855 (233,614,123,537–395,618,837,879)	398,164,867,866 (310,599,081,779–475,442,003,774)	416,808,237,778 (329,516,361,915–485,854,198,410)	481,761,964,833 (403,628,522,651–564,934,365,691)
Angola	All causes	1,237,129,005,850 (1,102,017,937,310–1,380,231,080,180)	1,387,886,378,040 (1,236,735,523,140–1,553,518,496,130)	1,383,595,471,160 (1,232,233,196,790–1,545,279,730,710)	1,130,034,998,360 (917,418,255,870–1,379,293,044,750)
Angola	Group I	963,466,022,769 (844,229,753,387–1,096,302,859,320)	1,054,824,733,600 (920,974,581,300–1,209,239,190,990)	980,224,756,105 (856,399,199,738–1,116,811,085,900)	676,377,170,493 (518,781,962,332–880,347,912,836)
Angola	NCD	179,741,830,052 (146,714,727,390–217,289,338,310)	232,564,184,178 (190,542,881,982–276,468,070,134)	287,878,436,718 (236,792,518,250–336,641,201,099)	347,263,093,244 (285,729,883,587–415,626,314,206)
Angola	Injuries	93,921,153,030 (67,371,024,730–117,774,813,060)	100,497,460,271 (74,319,749,249–126,009,247,330)	115,492,278,340 (87,382,723,618–140,404,626,522)	106,394,734,620 (83,483,086,241–131,271,089,628)
Central African Republic	All causes	303,769,913,528 (278,927,003,370–332,787,164,347)	425,286,158,340 (380,011,875,855–473,066,377,317)	446,618,941,943 (399,511,572,703–496,669,778,913)	448,514,212,158 (382,084,184,010–527,132,512,916)
Central African Republic	Group I	222,301,481,781 (199,225,719,346–249,609,004,972)	324,903,450,581 (286,009,183,109–366,883,581,923)	332,698,303,813 (294,616,592,622–377,925,126,282)	316,673,673,820 (262,393,080,964–385,501,859,477)
Central African Republic	NCD	61,918,329,917 (52,724,723,062–70,776,847,035)	76,130,986,511 (63,084,198,028–88,980,168,230)	84,697,223,171 (69,863,820,817–99,063,492,497)	99,647,016,213 (81,927,058,285–116,540,514,344)
Central African Republic	Injuries	19,550,101,830 (14,786,201,268–23,592,717,856)	24,251,721,248 (18,323,841,233–29,516,504,778)	29,223,414,959 (22,992,198,346–35,326,637,073)	32,193,522,125 (25,554,736,376–39,351,054,618)
Congo (Brazzaville)	All causes	176,785,644,446 (160,165,856,180–194,012,613,059)	246,609,650,561 (225,214,047,064–271,480,220,724)	230,386,838,591 (209,561,096,882–251,148,557,104)	217,523,183,201 (184,276,761,356–254,990,437,267)
Congo (Brazzaville)	Group I	115,404,769,121 (102,714,450,481–129,492,365,907)	167,978,975,042 (151,465,633,371–187,991,476,367)	152,214,685,833 (137,205,814,462–168,950,128,948)	126,699,146,721 (104,762,052,257–154,929,546,749)
Congo (Brazzaville)	NCD	47,918,312,195 (41,382,948,508–54,670,945,751)	58,724,965,889 (50,035,370,905–67,478,755,060)	61,573,760,690 (52,451,913,858–70,819,988,797)	73,398,045,030 (61,064,680,650–85,899,800,706)
Congo (Brazzaville)	Injuries	13,462,563,131 (10,580,588,716–16,107,235,822)	19,905,709,629 (15,474,613,435–24,358,781,085)	16,598,392,068 (13,375,296,372–19,598,831,496)	17,425,991,450 (14,080,976,095–21,083,790,500)
Democratic Republic of the Congo	All causes	3,235,786,548,750 (2,268,159,218,020–4,037,213,387,570)	4,247,809,597,070 (3,142,309,325,600–5,282,480,798,700)	4,452,788,749,810 (3,554,937,467,410–5,313,783,048,640)	4,457,827,617,450 (3,471,554,629,890–5,547,309,706,170)
Democratic Republic of the Congo	Group I	2,476,098,736,560 (1,620,943,422,680–3,191,442,243,970)	3,263,743,422,560 (2,239,522,446,650–4,237,762,511,890)	3,349,850,320,860 (2,522,056,870,090–4,146,531,749,560)	2,991,242,771,090 (2,159,521,486,560–3,968,908,879,450)
Democratic Republic of the Congo	NCD	571,961,860,232 (460,543,360,557–675,384,680,584)	738,752,808,444 (607,232,736,854–871,806,532,289)	856,249,215,479 (711,871,589,959–992,298,520,514)	1,149,476,042,990 (966,986,466,231–1,341,044,205,230)
Democratic Republic of the Congo	Injuries	187,725,951,960 (126,587,541,497–245,889,855,191)	245,313,366,062 (186,003,808,750–304,468,598,495)	246,689,213,472 (194,124,983,150–300,222,325,806)	317,108,803,362 (257,304,504,071–387,672,991,433)
Equatorial Guinea	All causes	44,234,825,085 (37,992,019,684–50,627,843,813)	40,604,923,619 (34,537,244,897–47,116,971,045)	37,807,584,960 (30,880,071,494–44,943,200,170)	35,796,755,968 (29,507,023,158–42,712,065,531)
Equatorial Guinea	Group I	32,757,143,356 (27,444,573,246–38,536,071,700)	28,567,732,937 (23,792,532,057–33,724,907,449)	25,803,676,197 (20,785,134,375–31,456,436,179)	22,199,918,191 (17,958,135,689–27,293,345,410)
Equatorial Guinea	NCD	8,504,343,340 (7,091,815,326–10,018,137,379)	9,175,417,353 (7,251,605,809–11,014,965,312)	9,444,669,095 (7,205,346,692–11,859,274,414)	11,231,221,397 (8,640,883,220–14,165,514,604)

Appendix Table 3. GBD 2016 DALY counts for all causes and level 1 cause groups by location for 1990, 2000, 2006 & 2016, both sexes combined.

	Cause	1990	2000	2006	2016
Equatorial Guinea	Injuries	2,973,338,388<br>(2,143,724,680–3,779,669,777)	2,861,773,329<br>(2,094,303,782–3,622,826,233)	2,559,239,667<br>(1,811,440,049–3,347,686,252)	2,365,616,380<br>(1,723,762,982–3,194,012,071)
Gabon	All causes	57,986,821,903<br>(50,596,849,397–65,162,890,073)	70,935,277,475<br>(63,720,183,678–78,202,180,691)	76,667,767,740<br>(68,426,017,839–85,600,494,524)	70,290,550,592<br>(61,303,988,423–80,772,478,035)
Gabon	Group I	32,349,053,646<br>(26,707,550,362–38,263,991,652)	41,163,181,632<br>(35,921,053,700–46,661,550,328)	43,509,613,998<br>(37,265,645,410–50,270,175,162)	34,851,993,796<br>(29,251,698,379–41,457,482,609)
Gabon	NCD	20,638,427,740<br>(18,145,402,480–23,125,965,913)	24,437,258,518<br>(21,240,709,580–27,635,776,915)	26,912,454,470<br>(23,302,791,571–30,683,417,098)	29,165,259,901<br>(24,577,236,359–34,162,406,369)
Gabon	Injuries	4,999,340,516<br>(4,184,803,626–5,851,476,700)	5,334,837,325<br>(4,457,010,405–6,249,884,310)	6,245,699,271<br>(5,223,308,025–7,438,024,878)	6,273,296,895<br>(5,064,304,033–7,649,177,767)

Appendix Table 4. GBD 2016 age-standardized rate of DALYs (per 100,000) for all causes and level 1 cause groups by location for 1990, 2000, 2006 & 2016, both sexes combined.

	Cause	1990	2000	2006	2016
Global	All causes	46,506.52<br>(43,786.28–49,543.89)	41,161.71<br>(38,377.56–44,167.39)	37,880.97<br>(35,110.32–40,910.02)	32,348.03<br>(29,547.76–35,600.63)
Global	Group I	21,163.14<br>(20,399.03–21,958.50)	16,516.00<br>(15,895.45–17,191.15)	13,974.08<br>(13,463.62–14,592.28)	9,034.07<br>(8,552.33–9,583.04)
Global	NCD	20,410.25<br>(18,189.72–22,736.48)	20,283.00<br>(18,061.05–22,629.49)	19,955.72<br>(17,689.00–22,395.13)	19,858.55<br>(17,539.04–22,436.07)
Global	Injuries	4,933.13<br>(4,608.94–5,258.00)	4,362.71<br>(4,078.58–4,678.59)	3,951.17<br>(3,684.14–4,273.37)	3,455.42<br>(3,193.73–3,797.06)
High SDI	All causes	29,033.97<br>(26,017.86–32,392.34)	27,531.45<br>(24,443.20–31,059.94)	27,020.30<br>(23,843.29–30,605.58)	27,231.34<br>(23,885.89–30,958.50)
High SDI	Group I	1,831.03<br>(1,685.51–2,003.29)	1,432.30<br>(1,305.10–1,590.08)	1,317.52<br>(1,193.81–1,471.13)	1,204.34<br>(1,083.35–1,366.13)
High SDI	NCD	23,852.65<br>(21,197.64–26,773.81)	23,155.41<br>(20,403.22–26,266.67)	22,926.58<br>(20,078.02–26,098.46)	23,425.63<br>(20,438.10–26,735.95)
High SDI	Injuries	3,350.29<br>(3,017.41–3,758.51)	2,943.75<br>(2,619.53–3,360.27)	2,776.20<br>(2,447.07–3,200.04)	2,601.37<br>(2,236.43–3,053.55)
High-middle SDI	All causes	33,249.36<br>(30,400.55–36,464.42)	31,735.01<br>(28,932.84–34,846.72)	29,735.85<br>(26,853.17–33,085.15)	26,633.72<br>(23,341.53–30,105.66)
High-middle SDI	Group I	5,906.71<br>(5,494.46–6,383.86)	3,863.30<br>(3,583.10–4,196.98)	3,106.75<br>(2,831.10–3,452.92)	2,274.94<br>(2,007.85–2,577.99)
High-middle SDI	NCD	22,254.41<br>(19,903.70–24,792.28)	23,137.51<br>(20,772.49–25,645.02)	22,473.35<br>(20,039.71–25,195.66)	21,134.53<br>(18,440.51–24,003.95)
High-middle SDI	Injuries	5,088.25<br>(4,657.24–5,553.25)	4,734.19<br>(4,361.77–5,160.71)	4,155.75<br>(3,799.23–4,563.27)	3,224.26<br>(2,804.66–3,702.85)
Middle SDI	All causes	37,872.88<br>(35,470.34–40,613.95)	32,395.15<br>(29,974.82–35,132.33)	30,809.77<br>(28,311.31–33,656.69)	28,548.51<br>(25,933.16–31,522.76)
Middle SDI	Group I	13,064.25<br>(12,510.90–13,716.57)	8,069.43<br>(7,696.55–8,547.81)	6,880.04<br>(6,535.73–7,289.96)	4,436.13<br>(4,113.04–4,808.52)
Middle SDI	NCD	19,612.25<br>(17,590.86–21,705.98)	19,914.87<br>(17,862.59–22,160.83)	19,966.57<br>(17,871.84–22,301.62)	20,739.56<br>(18,575.39–23,242.58)
Middle SDI	Injuries	5,196.38<br>(4,938.27–5,440.45)	4,410.86<br>(4,183.39–4,646.83)	3,963.17<br>(3,734.22–4,205.90)	3,372.82<br>(3,133.06–3,654.49)
Low-middle SDI	All causes	64,284.69<br>(61,403.82–67,617.51)	52,968.79<br>(50,118.05–56,086.21)	46,609.29<br>(43,745.73–49,815.10)	36,119.63<br>(33,251.17–39,270.40)
Low-middle SDI	Group I	40,718.02<br>(39,222.48–42,272.59)	30,168.52<br>(28,899.15–31,426.93)	24,313.09<br>(23,219.23–25,432.92)	14,261.26<br>(13,354.50–15,240.35)
Low-middle SDI	NCD	18,495.43<br>(16,366.24–20,707.14)	18,273.14<br>(16,190.70–20,533.19)	18,005.29<br>(15,894.38–20,231.23)	18,012.06<br>(15,922.99–20,303.05)
Low-middle SDI	Injuries	5,071.24<br>(4,558.26–5,516.54)	4,527.14<br>(4,123.99–4,881.60)	4,290.91<br>(3,934.66–4,624.70)	3,846.31<br>(3,516.93–4,218.01)
Low SDI	All causes	97,649.55<br>(93,290.32–101,970.49)	83,597.34<br>(79,659.97–87,448.08)	69,713.33<br>(66,501.95–73,237.90)	49,001.61<br>(45,848.29–52,531.07)
Low SDI	Group I	72,332.54<br>(68,861.16–76,016.30)	60,536.56<br>(57,645.76–63,407.08)	48,586.73<br>(46,451.48–50,834.55)	29,114.37<br>(27,393.69–30,984.28)
Low SDI	NCD	18,648.14<br>(16,323.97–21,055.84)	17,404.00<br>(15,338.14–19,615.61)	16,629.14<br>(14,724.86–18,738.68)	15,729.75<br>(13,842.52–17,727.98)
Low SDI	Injuries	6,668.88<br>(5,715.91–7,518.50)	5,656.77<br>(5,075.46–6,224.62)	4,497.46<br>(4,118.20–4,894.14)	4,157.50<br>(3,849.27–4,525.54)
High-income	All causes	28,658.69<br>(25,656.84–32,049.10)	27,151.40<br>(24,095.78–30,677.36)	26,514.42<br>(23,338.39–30,071.40)	26,702.71<br>(23,381.02–30,410.04)
High-income	Group I	1,911.55<br>(1,773.42–2,077.26)	1,519.96<br>(1,394.05–1,676.33)	1,387.23<br>(1,263.14–1,541.82)	1,262.57<br>(1,140.15–1,419.88)
High-income	NCD	23,538.94<br>(20,856.38–26,495.79)	22,812.77<br>(20,068.84–25,913.98)	22,478.11<br>(19,646.55–25,640.15)	22,949.88<br>(19,947.01–26,279.38)
High-income	Injuries	3,208.20<br>(2,892.42–3,594.76)	2,818.67<br>(2,507.05–3,214.99)	2,649.07<br>(2,332.08–3,047.75)	2,490.25<br>(2,146.11–2,917.20)
High-income North America	All causes	29,539.86<br>(26,406.83–32,976.99)	27,904.97<br>(24,775.75–31,502.15)	27,564.13<br>(24,340.86–31,196.74)	28,225.62<br>(24,919.31–32,003.05)
High-income North America	Group I	2,155.04<br>(2,037.20–2,298.77)	1,612.19<br>(1,502.96–1,747.00)	1,505.88<br>(1,393.68–1,644.89)	1,349.88<br>(1,232.95–1,494.93)
High-income North America	NCD	23,838.11<br>(21,023.78–26,913.34)	23,241.41<br>(20,434.03–26,398.26)	23,076.22<br>(20,145.87–26,323.39)	23,991.94<br>(20,963.56–27,390.36)
High-income North America	Injuries	3,546.72<br>(3,239.97–3,938.13)	3,051.36<br>(2,732.67–3,455.50)	2,982.02<br>(2,673.45–3,378.15)	2,883.80<br>(2,538.71–3,313.33)
Canada	All causes	25,029.78<br>(22,137.68–28,253.38)	24,152.99<br>(21,121.35–27,525.42)	23,899.18<br>(20,867.00–27,278.99)	24,292.73<br>(21,027.22–27,863.24)
Canada	Group I	1,322.49<br>(1,213.01–1,454.21)	1,060.83<br>(956.95–1,191.34)	1,032.92<br>(929.92–1,158.76)	1,022.92<br>(903.75–1,159.67)
Canada	NCD	20,754.77<br>(18,164.65–23,637.49)	20,616.80<br>(17,918.64–23,555.30)	20,464.82<br>(17,736.01–23,476.72)	20,963.35<br>(18,071.84–24,128.75)
Canada	Injuries	2,952.52<br>(2,651.84–3,356.16)	2,475.36<br>(2,174.66–2,858.22)	2,401.39<br>(2,075.38–2,820.64)	2,306.46<br>(1,965.80–2,764.64)
Greenland	All causes	47,117.48<br>(43,629.16–51,256.43)	38,595.53<br>(35,133.60–42,347.28)	37,246.47<br>(33,860.10–40,871.07)	34,762.46<br>(29,705.93–40,118.70)
Greenland	Group I	6,070.96<br>(5,416.62–6,805.99)	3,233.84<br>(2,915.18–3,577.26)	2,820.28<br>(2,488.39–3,119.91)	2,274.56<br>(1,915.71–2,618.62)
Greenland	NCD	25,554.07<br>(22,843.75–28,545.17)	24,784.65<br>(22,011.86–27,782.41)	24,921.31<br>(22,090.85–27,847.60)	24,963.87<br>(21,007.43–28,943.18)
Greenland	Injuries	15,492.45<br>(13,964.28–16,967.06)	10,577.04<br>(9,697.01–11,494.58)	9,504.88<br>(8,737.37–10,359.98)	7,524.03<br>(6,188.95–9,108.64)
United States	All causes	30,043.31<br>(26,869.62–33,504.17)	28,317.34<br>(25,137.13–31,912.43)	27,968.42<br>(24,717.89–31,632.53)	28,672.91<br>(25,312.00–32,439.40)
United States	Group I	2,247.02<br>(2,124.89–2,392.03)	1,672.26<br>(1,563.88–1,807.34)	1,557.66<br>(1,445.06–1,697.68)	1,386.89<br>(1,268.12–1,534.43)
United States	NCD	24,185.01<br>(21,341.18–27,279.01)	23,531.58<br>(20,706.50–26,696.40)	23,365.65<br>(20,412.90–26,633.91)	24,337.24<br>(21,297.26–27,736.51)
United States	Injuries	3,611.28<br>(3,303.95–4,007.07)	3,113.50<br>(2,795.42–3,526.94)	3,045.11<br>(2,736.70–3,443.34)	2,948.77<br>(2,600.72–3,380.30)
Australasia	All causes	25,968.76<br>(22,889.84–29,334.27)	23,919.81<br>(20,897.62–27,325.35)	22,986.48<br>(19,836.14–26,575.33)	22,653.58<br>(19,566.94–26,132.72)
Australasia	Group I	1,239.03<br>(1,129.95–1,375.03)	986.50<br>(880.61–1,112.18)	926.59<br>(826.11–1,050.94)	804.39<br>(695.57–935.36)
Australasia	NCD	21,820.36<br>(19,069.71–24,777.36)	20,547.68<br>(17,736.21–23,628.89)	19,895.93<br>(17,030.42–23,091.93)	19,837.72<br>(16,988.28–23,027.67)
Australasia	Injuries	2,909.37<br>(2,635.75–3,247.23)	2,385.62<br>(2,114.60–2,724.09)	2,163.96<br>(1,894.92–2,528.35)	2,011.46<br>(1,716.22–2,384.36)
Australia	All causes	25,662.13<br>(22,620.80–28,996.85)	23,800.64<br>(20,739.12–27,260.69)	22,887.97<br>(19,731.60–26,482.71)	22,504.97<br>(19,417.84–25,980.63)
Australia	Group I	1,210.93<br>(1,098.53–1,348.14)	982.20<br>(878.44–1,108.08)	931.20<br>(830.10–1,057.14)	799.96<br>(689.51–931.76)
Australia	NCD	21,644.08<br>(18,916.64–24,602.44)	20,467.67<br>(17,643.41–23,534.59)	19,832.19<br>(16,945.55–23,044.41)	19,721.52<br>(16,821.26–22,932.89)
Australia	Injuries	2,807.12<br>(2,530.44–3,147.14)	2,350.78<br>(2,077.99–2,687.35)	2,124.57<br>(1,852.18–2,489.91)	1,983.48<br>(1,685.56–2,358.72)
New Zealand	All causes	27,512.78<br>(24,597.17–30,855.15)	24,507.04<br>(21,424.28–27,861.73)	23,468.49<br>(20,419.57–26,994.70)	23,440.51<br>(20,184.33–27,051.64)
New Zealand	Group I	1,380.54<br>(1,263.17–1,522.85)	1,007.74<br>(893.10–1,148.36)	904.02<br>(792.61–1,042.76)	827.88<br>(713.47–972.33)
New Zealand	NCD	22,708.01<br>(19,978.94–25,722.72)	20,941.96<br>(18,177.74–23,941.29)	20,207.82<br>(17,440.36–23,339.38)	20,453.05<br>(17,496.83–23,698.24)
New Zealand	Injuries	3,424.24<br>(3,143.70–3,786.07)	2,557.34<br>(2,278.76–2,907.05)	2,356.65<br>(2,083.08–2,728.24)	2,159.59<br>(1,844.63–2,570.72)
High-income Asia Pacific	All causes	23,997.65<br>(21,172.43–27,043.65)	24,306.33<br>(21,325.41–27,629.05)	24,583.78<br>(21,341.83–28,106.25)	25,652.09<br>(22,178.88–29,522.50)
High-income Asia Pacific	Group I	1,658.39<br>(1,491.94–1,857.78)	1,428.07<br>(1,283.31–1,614.25)	1,283.94<br>(1,145.79–1,466.27)	1,232.17<br>(1,092.86–1,410.42)
High-income Asia Pacific	NCD	19,259.80<br>(16,827.25–21,925.59)	19,934.60<br>(17,323.72–22,802.87)	20,509.58<br>(17,692.79–23,496.07)	21,791.32<br>(18,752.60–25,049.76)
High-income Asia Pacific	Injuries	3,079.46<br>(2,748.93–3,477.72)	2,943.66<br>(2,602.26–3,380.13)	2,790.27<br>(2,438.92–3,235.97)	2,628.59<br>(2,217.06–3,153.37)
Brunei	All causes	20,459.93<br>(18,003.21–23,106.05)	18,120.98<br>(15,735.45–20,593.16)	18,276.31<br>(15,834.71–20,815.21)	20,706.07<br>(17,494.92–24,061.77)
Brunei	Group I	2,353.18<br>(2,115.40–2,628.60)	1,871.04<br>(1,671.14–2,114.07)	1,653.06<br>(1,470.81–1,882.08)	1,623.72<br>(1,404.58–1,873.95)
Brunei	NCD	14,675.48<br>(12,683.93–16,837.87)	13,497.81<br>(11,518.07–15,551.75)	14,114.99<br>(12,089.51–16,187.95)	16,547.26<br>(13,944.00–19,378.52)
Brunei	Injuries	3,431.28<br>(2,968.95–3,921.01)	2,752.12<br>(2,400.15–3,161.36)	2,508.26<br>(2,154.31–2,910.81)	2,535.09<br>(2,021.83–3,133.89)
Japan	All causes	23,453.57<br>(20,646.25–26,641.14)	24,839.39<br>(21,791.61–28,254.09)	25,593.14<br>(22,425.85–29,244.07)	26,660.22<br>(23,217.76–30,569.54)
Japan	Group I	1,384.88<br>(1,262.09–1,544.44)	1,367.55<br>(1,243.13–1,524.24)	1,287.44<br>(1,162.84–1,444.40)	1,265.69<br>(1,135.76–1,434.98)
Japan	NCD	19,480.57<br>(16,971.39–22,262.05)	20,750.73<br>(18,056.69–23,726.84)	21,640.10<br>(18,788.59–24,786.01)	22,916.10<br>(19,769.87–26,297.14)
Japan	Injuries	2,588.12<br>(2,276.26–2,973.98)	2,721.10<br>(2,382.82–3,165.16)	2,665.60<br>(2,313.35–3,128.52)	2,478.44<br>(2,097.19–2,977.10)
Singapore	All causes	21,506.80<br>(18,729.49–24,511.85)	19,162.94<br>(16,300.10–22,180.49)	18,835.34<br>(16,071.04–21,823.79)	19,086.83<br>(15,807.19–22,550.64)
Singapore	Group I	2,097.92<br>(1,878.44–2,362.80)	1,591.36<br>(1,400.85–1,826.37)	1,513.85<br>(1,323.20–1,740.72)	1,422.85<br>(1,206.40–1,674.92)
Singapore	NCD	17,021.42<br>(14,748.97–19,557.80)	15,757.10<br>(13,283.81–18,225.26)	15,620.58<br>(13,233.66–18,113.56)	16,026.74<br>(13,258.70–19,023.10)

Appendix Table 4. GBD 2016 age-standardized rate of DALYs (per 100,000) for all causes and level 1 cause groups by location for 1990, 2000, 2006 & 2016, both sexes combined.

	Cause	1990	2000	2006	2016
Singapore	Injuries	2,387.46 (2,061.11–2,781.70)	1,814.49 (1,518.04–2,193.76)	1,700.91 (1,384.09–2,110.99)	1,637.23 (1,270.18–2,089.42)
South Korea	All causes	25,732.42 (22,789.85–29,088.81)	23,265.21 (20,317.46–26,488.10)	22,388.74 (19,238.18–25,726.38)	23,689.81 (19,271.75–28,583.34)
South Korea	Group I	2,407.12 (2,066.87–2,799.27)	1,578.09 (1,366.03–1,841.58)	1,254.27 (1,047.96–1,500.03)	1,130.28 (907.90–1,390.14)
South Korea	NCD	18,800.35 (16,417.18–21,355.63)	18,055.41 (15,646.80–20,618.48)	17,928.05 (15,237.62–20,624.72)	19,477.86 (15,899.46–23,426.91)
South Korea	Injuries	4,524.95 (4,013.17–5,121.00)	3,631.71 (3,208.15–4,112.30)	3,206.41 (2,768.15–3,654.47)	3,081.67 (2,385.43–3,886.31)
Western Europe	All causes	30,029.86 (26,895.73–33,565.42)	28,020.14 (24,852.32–31,665.83)	26,805.04 (23,558.56–30,464.29)	26,350.30 (22,971.04–30,126.59)
Western Europe	Group I	1,523.98 (1,385.70–1,689.71)	1,270.19 (1,138.27–1,433.40)	1,156.53 (1,029.15–1,319.69)	1,057.15 (932.10–1,221.16)
Western Europe	NCD	25,489.07 (22,690.19–28,600.88)	24,179.83 (21,342.25–27,383.76)	23,326.09 (20,443.64–26,539.87)	23,194.22 (20,171.71–26,493.09)
Western Europe	Injuries	3,016.81 (2,690.98–3,427.72)	2,570.11 (2,243.65–2,978.75)	2,322.42 (1,986.24–2,743.40)	2,098.93 (1,740.33–2,538.24)
Andorra	All causes	25,867.17 (21,554.79–30,115.46)	24,162.75 (20,242.77–28,149.99)	24,327.11 (20,546.36–28,581.35)	25,922.03 (21,860.56–30,734.23)
Andorra	Group I	1,324.67 (979.47–1,925.39)	1,137.11 (815.64–1,551.58)	1,025.51 (752.55–1,326.58)	1,043.77 (788.28–1,317.16)
Andorra	NCD	22,323.94 (18,513.25–26,026.80)	21,080.29 (17,641.43–24,647.98)	21,370.30 (18,035.44–25,139.66)	22,889.70 (19,334.33–27,204.81)
Andorra	Injuries	2,218.56 (1,734.48–2,783.73)	1,945.35 (1,538.94–2,442.52)	1,931.30 (1,533.36–2,411.78)	1,988.56 (1,565.63–2,505.68)
Austria	All causes	31,156.39 (27,959.03–34,664.60)	27,831.10 (24,626.90–31,396.48)	26,492.41 (23,305.19–30,160.21)	26,189.01 (22,734.26–30,044.88)
Austria	Group I	1,250.70 (1,116.23–1,413.34)	918.08 (778.00–1,075.61)	836.94 (704.58–994.50)	730.89 (606.57–894.91)
Austria	NCD	26,183.68 (23,435.36–29,181.74)	23,977.17 (21,198.21–27,076.86)	23,029.75 (20,159.58–26,276.01)	23,096.20 (19,995.96–26,480.74)
Austria	Injuries	3,722.01 (3,323.00–4,205.16)	2,935.85 (2,548.67–3,411.92)	2,625.72 (2,244.02–3,101.05)	2,361.91 (1,956.27–2,847.21)
Belgium	All causes	30,880.95 (27,718.14–34,534.07)	29,518.84 (26,131.19–33,216.90)	28,159.92 (24,823.45–31,907.73)	27,030.42 (23,531.18–30,876.14)
Belgium	Group I	1,397.50 (1,265.27–1,547.69)	1,342.45 (1,211.35–1,508.30)	1,309.85 (1,178.53–1,475.77)	1,201.25 (1,068.85–1,376.39)
Belgium	NCD	25,967.05 (23,118.65–29,215.11)	24,840.99 (21,857.06–28,074.98)	23,758.94 (20,807.13–27,007.99)	23,091.41 (19,992.82–26,425.48)
Belgium	Injuries	3,516.40 (3,152.26–3,942.11)	3,335.39 (2,963.53–3,782.29)	3,091.13 (2,707.46–3,601.41)	2,737.76 (2,328.52–3,242.62)
Cyprus	All causes	26,923.63 (24,035.80–29,985.30)	26,508.05 (23,504.06–29,820.26)	25,023.01 (22,017.96–28,466.38)	23,775.65 (20,572.62–27,154.85)
Cyprus	Group I	2,004.99 (1,768.78–2,276.63)	1,342.79 (1,178.82–1,536.68)	1,117.37 (970.47–1,285.27)	922.02 (774.03–1,085.54)
Cyprus	NCD	21,863.51 (19,309.04–24,548.94)	21,990.35 (19,334.78–24,971.26)	21,127.53 (18,518.04–24,159.97)	20,557.99 (17,668.18–23,526.91)
Cyprus	Injuries	3,055.13 (2,745.41–3,438.20)	3,174.91 (2,851.44–3,595.88)	2,778.11 (2,447.30–3,201.91)	2,295.64 (1,947.60–2,729.99)
Denmark	All causes	32,857.46 (29,438.41–36,556.32)	30,339.68 (26,936.60–33,990.92)	28,923.19 (25,462.77–32,712.20)	27,015.50 (23,393.78–30,931.39)
Denmark	Group I	1,391.00 (1,262.61–1,546.99)	1,188.18 (1,064.09–1,345.63)	1,197.43 (1,074.51–1,345.00)	1,107.27 (968.02–1,271.20)
Denmark	NCD	28,036.36 (25,026.22–31,260.32)	26,524.24 (23,577.14–29,769.03)	25,448.95 (22,366.80–28,762.07)	23,945.29 (20,724.41–27,408.68)
Denmark	Injuries	3,430.11 (3,016.63–3,937.48)	2,627.26 (2,271.00–3,079.02)	2,276.80 (1,934.68–2,704.64)	1,962.94 (1,591.67–2,425.72)
Finland	All causes	31,790.34 (28,423.78–35,511.66)	29,224.24 (25,826.20–33,087.49)	28,819.55 (25,337.34–32,658.29)	26,912.72 (23,275.87–30,811.35)
Finland	Group I	1,513.52 (1,369.68–1,688.76)	1,210.26 (1,077.82–1,372.87)	907.78 (780.83–1,055.29)	711.98 (589.52–861.03)
Finland	NCD	26,008.63 (23,120.53–29,230.18)	24,397.90 (21,381.68–27,622.12)	24,454.75 (21,472.16–27,777.23)	23,543.29 (20,287.95–26,979.86)
Finland	Injuries	4,268.19 (3,808.06–4,763.67)	3,616.08 (3,173.01–4,177.95)	3,457.03 (3,002.17–4,058.82)	2,657.44 (2,205.74–3,193.79)
France	All causes	28,130.86 (25,086.17–31,578.24)	26,644.22 (23,577.36–30,115.29)	25,455.79 (22,392.48–28,986.90)	25,008.25 (21,668.67–28,712.86)
France	Group I	1,548.85 (1,427.11–1,697.72)	1,252.79 (1,133.14–1,390.76)	1,117.62 (1,002.54–1,262.13)	1,032.74 (914.97–1,182.77)
France	NCD	22,617.33 (19,945.80–25,552.65)	22,029.01 (19,305.21–25,142.77)	21,423.72 (18,641.57–24,524.54)	21,386.29 (18,412.35–24,573.56)
France	Injuries	3,964.67 (3,606.23–4,422.70)	3,362.42 (2,998.45–3,804.09)	2,914.45 (2,557.69–3,360.33)	2,589.23 (2,191.63–3,050.09)
Germany	All causes	32,771.21 (29,434.37–36,454.23)	29,886.94 (26,416.77–33,757.90)	29,148.91 (25,618.25–32,914.50)	29,458.12 (25,575.49–33,803.11)
Germany	Group I	1,360.94 (1,215.71–1,531.13)	1,076.04 (940.27–1,243.63)	1,061.05 (930.04–1,218.85)	1,040.28 (902.61–1,208.10)
Germany	NCD	28,468.98 (25,530.62–31,738.27)	26,358.47 (23,327.17–29,834.91)	25,875.36 (22,756.03–29,218.36)	26,296.72 (22,852.70–30,051.71)
Germany	Injuries	2,941.29 (2,591.84–3,397.68)	2,452.43 (2,119.53–2,863.31)	2,212.50 (1,864.58–2,623.32)	2,121.12 (1,744.59–2,578.77)
Greece	All causes	28,095.34 (25,052.65–31,616.36)	27,493.62 (24,339.50–31,070.15)	27,288.44 (24,088.42–30,963.17)	28,562.11 (25,033.29–32,282.48)
Greece	Group I	1,252.67 (1,104.36–1,450.32)	1,049.66 (921.59–1,210.17)	949.40 (823.14–1,109.62)	908.63 (772.25–1,063.28)
Greece	NCD	24,056.15 (21,370.28–27,094.78)	23,880.22 (21,061.93–27,018.30)	24,020.31 (21,137.84–27,187.20)	25,540.84 (22,444.02–28,836.81)
Greece	Injuries	2,786.52 (2,449.68–3,218.40)	2,563.74 (2,227.56–2,981.10)	2,318.74 (1,968.51–2,738.81)	2,112.65 (1,745.19–2,587.40)
Iceland	All causes	23,397.77 (20,510.95–26,646.68)	21,946.61 (19,069.53–25,231.05)	21,113.02 (18,165.64–24,514.25)	21,629.60 (18,432.14–25,116.50)
Iceland	Group I	1,436.57 (1,301.53–1,613.96)	1,071.19 (945.25–1,237.98)	936.94 (815.10–1,091.41)	852.30 (722.10–1,003.20)
Iceland	NCD	19,364.81 (16,805.79–22,172.35)	18,715.51 (16,135.65–21,624.79)	18,215.80 (15,603.27–21,277.67)	18,870.68 (16,031.18–22,018.61)
Iceland	Injuries	2,596.39 (2,327.34–2,944.28)	2,159.91 (1,875.68–2,503.92)	1,960.28 (1,671.54–2,324.95)	1,906.62 (1,584.14–2,333.54)
Ireland	All causes	27,575.72 (24,621.39–30,927.17)	26,153.24 (23,031.12–29,430.49)	23,055.52 (20,033.11–26,418.22)	22,539.94 (19,358.68–26,254.53)
Ireland	Group I	1,599.88 (1,455.59–1,787.46)	1,441.69 (1,305.23–1,607.63)	1,060.22 (929.99–1,221.86)	923.15 (786.80–1,091.78)
Ireland	NCD	23,469.18 (20,831.82–26,323.75)	22,172.77 (19,393.24–25,148.76)	19,795.40 (17,109.78–22,741.72)	19,700.92 (16,884.28–22,950.19)
Ireland	Injuries	2,506.65 (2,219.98–2,866.99)	2,538.77 (2,216.84–2,931.54)	2,199.90 (1,891.26–2,593.47)	1,915.87 (1,571.58–2,367.77)
Israel	All causes	23,680.31 (20,872.62–26,761.29)	21,931.72 (19,021.78–25,158.69)	20,950.07 (18,150.54–24,098.56)	19,257.90 (16,175.56–22,567.17)
Israel	Group I	2,006.84 (1,826.84–2,260.91)	1,481.16 (1,318.88–1,670.76)	1,293.17 (1,142.85–1,481.23)	1,101.03 (942.08–1,286.40)
Israel	NCD	19,261.26 (16,829.86–21,970.96)	18,131.92 (15,559.56–20,842.56)	17,112.85 (14,634.31–19,804.62)	16,339.82 (13,691.90–19,195.24)
Israel	Injuries	2,412.21 (2,118.53–2,778.75)	2,318.65 (2,007.30–2,719.05)	2,544.05 (2,213.76–2,938.05)	1,817.05 (1,448.48–2,254.87)
Italy	All causes	29,178.48 (25,961.46–32,761.62)	27,784.44 (24,492.27–31,468.50)	26,806.97 (23,476.45–30,579.73)	26,929.03 (23,371.81–30,765.71)
Italy	Group I	1,370.42 (1,231.20–1,530.45)	1,027.49 (898.16–1,183.10)	949.73 (818.67–1,113.39)	825.05 (686.35–990.12)
Italy	NCD	25,067.27 (22,217.46–28,208.68)	24,325.45 (21,439.68–27,565.97)	23,682.04 (20,732.60–26,941.25)	24,047.83 (20,945.30–27,404.18)
Italy	Injuries	2,740.79 (2,395.52–3,161.14)	2,431.50 (2,070.45–2,870.08)	2,175.20 (1,814.03–2,618.95)	2,056.15 (1,671.02–2,533.84)
Luxembourg	All causes	31,985.71 (28,653.38–35,751.26)	26,817.67 (23,507.10–30,551.62)	24,606.64 (21,415.08–28,285.53)	23,245.98 (19,846.44–26,884.55)
Luxembourg	Group I	1,356.23 (1,220.77–1,518.26)	1,098.84 (968.76–1,255.26)	954.26 (832.15–1,109.89)	859.46 (733.85–1,007.49)
Luxembourg	NCD	26,942.71 (23,996.18–30,301.37)	22,952.49 (20,090.01–26,225.78)	21,357.98 (18,381.57–24,644.37)	20,364.28 (17,300.25–23,646.46)
Luxembourg	Injuries	3,686.76 (3,278.42–4,180.95)	2,766.34 (2,398.26–3,229.53)	2,294.39 (1,952.18–2,728.98)	2,022.24 (1,672.71–2,469.33)
Malta	All causes	25,478.91 (22,300.20–28,831.33)	25,075.02 (21,779.77–28,531.37)	24,921.49 (21,827.23–28,349.14)	25,280.79 (21,314.70–29,498.86)
Malta	Group I	1,721.96 (1,557.41–1,936.02)	1,392.03 (1,242.68–1,567.60)	1,170.98 (1,036.83–1,333.72)	1,070.73 (915.04–1,252.94)
Malta	NCD	21,747.35 (18,935.71–24,671.28)	21,756.44 (18,878.41–24,881.14)	21,865.59 (19,098.97–24,777.74)	22,407.47 (18,890.86–26,119.29)
Malta	Injuries	2,009.60 (1,678.54–2,395.70)	1,926.55 (1,610.44–2,345.24)	1,884.93 (1,547.52–2,297.57)	1,802.59 (1,419.21–2,300.18)
Netherlands	All causes	27,255.33 (24,217.71–30,720.43)	27,090.23 (23,866.40–30,844.15)	25,597.09 (22,280.04–29,338.51)	25,422.94 (21,795.50–29,261.80)
Netherlands	Group I	1,327.92 (1,184.29–1,487.98)	1,376.37 (1,239.58–1,544.95)	1,219.49 (1,085.47–1,382.41)	1,100.99 (956.65–1,260.68)

Appendix Table 4. GBD 2016 age-standardized rate of DALYs (per 100,000) for all causes and level 1 cause groups by location for 1990, 2000, 2006 & 2016, both sexes combined.

	Cause	1990	2000	2006	2016
Netherlands	NCD	23,728.47 (20,977.25–26,838.36)	23,813.06 (20,918.06–27,204.47)	22,657.90 (19,654.63–25,965.61)	22,554.45 (19,302.19–25,997.79)
Netherlands	Injuries	2,198.93 (1,924.83–2,538.17)	1,900.81 (1,657.94–2,199.86)	1,719.70 (1,462.41–2,042.76)	1,767.50 (1,446.98–2,165.59)
Norway	All causes	29,658.15 (26,457.59–33,280.98)	26,898.00 (23,843.42–30,333.20)	24,836.21 (21,685.61–28,317.35)	23,078.76 (19,815.97–26,483.15)
Norway	Group I	1,579.61 (1,447.35–1,738.69)	1,180.75 (1,046.42–1,347.99)	1,060.19 (937.47–1,210.31)	921.52 (790.11–1,084.67)
Norway	NCD	25,031.60 (22,123.43–28,236.28)	23,230.16 (20,435.06–26,326.95)	21,555.94 (18,802.11–24,584.91)	20,129.67 (17,162.03–23,262.40)
Norway	Injuries	3,046.94 (2,704.48–3,456.80)	2,487.09 (2,168.29–2,886.92)	2,220.09 (1,898.15–2,633.17)	2,027.57 (1,677.86–2,455.40)
Portugal	All causes	31,951.15 (28,983.99–35,529.27)	30,602.52 (27,298.61–34,269.68)	28,684.61 (25,464.22–32,395.10)	28,218.97 (24,645.03–32,236.56)
Portugal	Group I	2,120.19 (1,947.13–2,341.03)	2,245.02 (2,053.00–2,495.81)	1,901.70 (1,712.50–2,145.04)	1,629.09 (1,416.12–1,900.23)
Portugal	NCD	25,682.20 (23,095.14–28,772.26)	25,226.41 (22,353.29–28,486.18)	24,207.79 (21,376.54–27,440.86)	24,407.68 (21,201.34–28,000.94)
Portugal	Injuries	4,148.75 (3,785.29–4,611.05)	3,131.10 (2,776.55–3,568.14)	2,575.11 (2,236.87–2,982.18)	2,182.20 (1,844.61–2,595.29)
Spain	All causes	27,007.58 (24,008.60–30,430.14)	26,028.07 (23,011.07–29,568.93)	24,789.17 (21,678.79–28,359.96)	23,961.35 (20,733.52–27,395.52)
Spain	Group I	1,561.42 (1,415.41–1,740.26)	1,281.29 (1,138.46–1,471.40)	1,157.15 (1,014.52–1,345.82)	968.19 (828.02–1,149.20)
Spain	NCD	22,532.57 (19,916.23–25,463.42)	22,298.95 (19,569.52–25,322.39)	21,384.66 (18,618.25–24,444.07)	21,193.11 (18,373.87–24,286.59)
Spain	Injuries	2,913.60 (2,592.43–3,303.62)	2,447.83 (2,109.51–2,866.20)	2,247.37 (1,872.07–2,694.78)	1,800.05 (1,459.01–2,232.16)
Sweden	All causes	30,402.81 (27,087.56–34,157.63)	27,807.65 (24,439.20–31,625.62)	26,758.56 (23,367.92–30,582.70)	25,629.87 (22,051.44–29,669.38)
Sweden	Group I	1,326.99 (1,207.97–1,480.26)	986.24 (867.26–1,131.08)	934.25 (816.38–1,084.94)	910.79 (779.99–1,068.50)
Sweden	NCD	26,254.51 (23,230.14–29,614.89)	24,507.98 (21,505.96–27,916.63)	23,575.43 (20,495.89–26,901.64)	22,580.38 (19,339.61–26,172.16)
Sweden	Injuries	2,821.30 (2,478.98–3,248.27)	2,313.43 (1,988.08–2,728.55)	2,248.88 (1,912.09–2,696.19)	2,138.70 (1,756.46–2,619.78)
Switzerland	All causes	28,948.64 (25,450.18–32,577.73)	26,530.36 (22,978.02–30,323.26)	25,258.09 (21,754.48–29,173.77)	23,787.73 (19,763.06–28,284.82)
Switzerland	Group I	1,561.11 (1,369.35–1,797.52)	1,317.91 (1,131.33–1,547.10)	1,179.37 (986.40–1,401.32)	1,082.92 (896.51–1,312.65)
Switzerland	NCD	23,490.79 (20,512.99–26,527.25)	22,267.79 (19,231.59–25,512.69)	21,544.85 (18,467.72–24,883.24)	20,617.88 (17,107.29–24,460.30)
Switzerland	Injuries	3,896.74 (3,442.53–4,470.95)	2,944.65 (2,523.73–3,446.01)	2,533.87 (2,134.29–3,018.97)	2,086.93 (1,641.96–2,644.97)
United Kingdom	All causes	31,994.44 (28,740.24–35,653.39)	29,006.69 (25,734.91–32,709.21)	27,321.61 (24,043.53–30,989.72)	26,362.71 (23,017.18–30,089.83)
United Kingdom	Group I	1,897.57 (1,748.36–2,086.05)	1,689.87 (1,552.64–1,869.43)	1,484.13 (1,355.00–1,652.91)	1,387.52 (1,247.49–1,566.51)
United Kingdom	NCD	27,822.37 (24,867.39–31,118.48)	25,321.56 (22,394.13–28,570.13)	23,936.72 (20,995.87–27,201.38)	23,191.27 (20,150.90–26,514.71)
United Kingdom	Injuries	2,274.50 (1,984.58–2,665.95)	1,995.26 (1,709.92–2,374.87)	1,900.76 (1,603.81–2,291.60)	1,783.92 (1,468.76–2,191.46)
England	All causes	31,711.93 (28,490.06–35,369.61)	28,678.17 (25,409.28–32,344.10)	26,943.99 (23,665.81–30,652.42)	25,927.09 (22,614.16–29,668.46)
England	Group I	1,896.88 (1,744.98–2,086.85)	1,705.09 (1,561.38–1,885.29)	1,494.30 (1,366.46–1,666.46)	1,403.12 (1,260.42–1,589.10)
England	NCD	27,600.60 (24,651.36–30,897.47)	25,038.48 (22,071.81–28,301.84)	23,610.09 (20,647.81–26,916.74)	22,798.87 (19,756.88–26,106.15)
England	Injuries	2,214.45 (1,918.74–2,605.14)	1,934.61 (1,643.83–2,309.98)	1,839.60 (1,538.03–2,224.74)	1,725.10 (1,412.69–2,129.18)
Northern Ireland	All causes	29,417.71 (26,264.88–32,940.79)	26,633.69 (23,504.30–30,252.78)	25,948.36 (22,563.69–29,457.14)	25,765.88 (22,000.19–29,610.61)
Northern Ireland	Group I	2,160.27 (1,977.04–2,369.41)	1,716.69 (1,560.05–1,898.30)	1,470.82 (1,325.95–1,638.21)	1,310.11 (1,129.64–1,507.01)
Northern Ireland	NCD	24,687.94 (21,867.82–27,783.13)	22,688.55 (19,865.79–25,841.47)	22,178.96 (19,167.08–25,256.65)	22,277.67 (18,908.78–25,650.20)
Northern Ireland	Injuries	2,569.51 (2,242.78–2,972.16)	2,228.45 (1,902.77–2,611.33)	2,298.58 (1,916.22–2,724.34)	2,178.10 (1,777.11–2,682.81)
Scotland	All causes	34,691.89 (31,363.03–38,540.09)	32,072.21 (28,781.02–35,987.46)	30,719.18 (27,312.09–34,377.10)	29,716.29 (25,836.57–33,826.76)
Scotland	Group I	1,924.88 (1,764.71–2,117.74)	1,569.80 (1,429.02–1,745.51)	1,438.11 (1,299.67–1,603.53)	1,290.63 (1,128.13–1,469.84)
Scotland	NCD	29,983.63 (26,990.08–33,349.36)	28,045.82 (25,093.47–31,412.06)	26,951.93 (23,966.91–30,244.21)	26,272.38 (22,776.62–29,861.73)
Scotland	Injuries	2,783.38 (2,383.12–3,209.49)	2,456.59 (2,041.93–2,893.60)	2,329.14 (1,909.13–2,760.22)	2,153.28 (1,757.49–2,647.23)
Wales	All causes	33,364.60 (29,923.30–37,190.48)	30,601.95 (27,141.22–34,314.65)	28,731.31 (25,365.20–32,384.55)	28,606.38 (24,810.77–32,672.40)
Wales	Group I	1,713.09 (1,553.60–1,895.55)	1,625.84 (1,471.84–1,808.30)	1,397.23 (1,245.01–1,568.85)	1,326.10 (1,165.73–1,515.30)
Wales	NCD	29,443.77 (26,363.91–32,811.05)	26,892.75 (23,758.88–30,260.73)	25,357.41 (22,287.40–28,558.52)	25,333.06 (21,963.12–29,003.51)
Wales	Injuries	2,207.75 (1,907.75–2,565.57)	2,083.36 (1,766.84–2,465.86)	1,976.68 (1,656.83–2,378.49)	1,947.22 (1,592.30–2,399.22)
Southern Latin America	All causes	30,162.40 (27,423.19–33,258.30)	27,025.85 (24,264.60–30,107.70)	25,902.22 (23,156.27–29,042.22)	25,311.71 (22,259.98–28,676.09)
Southern Latin America	Group I	4,728.02 (4,496.26–5,019.71)	3,296.66 (3,102.62–3,538.74)	2,822.26 (2,636.33–3,036.66)	2,414.18 (2,211.17–2,647.63)
Southern Latin America	NCD	22,088.19 (19,635.31–24,774.54)	20,667.01 (18,194.73–23,376.58)	20,262.19 (17,812.60–23,065.95)	20,179.09 (17,461.15–23,113.76)
Southern Latin America	Injuries	3,346.19 (3,112.65–3,627.75)	3,062.17 (2,846.53–3,333.01)	2,817.76 (2,605.15–3,087.61)	2,718.44 (2,427.30–3,074.68)
Argentina	All causes	31,325.16 (28,571.04–34,434.69)	28,480.30 (25,768.40–31,566.89)	27,031.36 (24,236.34–30,073.27)	26,031.48 (23,044.60–29,223.79)
Argentina	Group I	5,135.34 (4,881.12–5,448.43)	3,837.51 (3,606.54–4,121.51)	3,334.42 (3,120.69–3,587.54)	2,829.67 (2,581.23–3,104.25)
Argentina	NCD	23,000.21 (20,546.83–25,765.66)	21,458.51 (19,050.69–24,216.44)	20,770.17 (18,350.90–23,482.36)	20,384.14 (17,771.83–23,247.68)
Argentina	Injuries	3,189.61 (2,948.62–3,469.09)	3,184.29 (2,941.62–3,479.25)	2,926.77 (2,697.39–3,227.88)	2,817.68 (2,520.49–3,170.22)
Chile	All causes	26,798.00 (23,986.78–29,804.75)	22,964.61 (20,126.33–26,131.27)	22,602.16 (19,717.94–25,928.82)	23,064.93 (19,162.68–27,539.18)
Chile	Group I	4,005.27 (3,699.60–4,338.37)	2,123.95 (1,953.84–2,334.43)	1,683.38 (1,522.10–1,879.38)	1,500.28 (1,296.99–1,733.83)
Chile	NCD	19,051.90 (16,562.28–21,621.19)	18,126.87 (15,548.68–21,021.82)	18,432.14 (15,836.21–21,372.79)	19,182.07 (15,828.76–22,986.97)
Chile	Injuries	3,740.83 (3,383.46–4,094.87)	2,713.79 (2,494.95–2,983.77)	2,486.64 (2,264.22–2,753.40)	2,382.58 (1,899.63–2,980.78)
Uruguay	All causes	32,301.55 (29,531.17–35,431.10)	29,507.07 (26,723.67–32,556.73)	28,680.91 (25,882.36–31,836.31)	28,044.96 (24,944.70–31,555.22)
Uruguay	Group I	3,536.31 (3,254.67–3,798.31)	2,675.53 (2,473.11–2,897.50)	2,328.64 (2,135.33–2,542.19)	1,965.46 (1,728.03–2,261.47)
Uruguay	NCD	25,455.06 (23,066.61–28,316.44)	23,526.21 (21,028.64–26,251.12)	23,204.29 (20,673.75–26,000.09)	22,846.35 (20,111.91–25,840.20)
Uruguay	Injuries	3,310.19 (3,041.11–3,616.14)	3,305.33 (3,057.35–3,633.37)	3,147.98 (2,879.70–3,452.76)	3,233.15 (2,885.25–3,610.03)
Central Europe, Eastern Europe, and Central Asia	All causes	38,520.33 (35,267.15–42,084.78)	42,906.62 (39,590.53–46,486.75)	42,104.44 (38,719.74–45,858.90)	37,197.54 (32,701.47–42,157.63)
Central Europe, Eastern Europe, and Central Asia	Group I	5,000.30 (4,726.00–5,347.00)	4,240.45 (3,951.91–4,559.36)	3,890.47 (3,618.16–4,239.66)	3,042.75 (2,706.31–3,411.28)
Central Europe, Eastern Europe, and Central Asia	NCD	27,625.76 (25,118.86–30,396.16)	31,810.28 (29,152.58–34,678.58)	32,023.33 (29,242.44–35,006.24)	29,331.10 (25,687.96–33,280.21)
Central Europe, Eastern Europe, and Central Asia	Injuries	5,894.27 (5,335.63–6,505.52)	6,855.89 (6,211.36–7,521.92)	6,190.64 (5,608.64–6,859.38)	4,823.70 (4,036.60–5,745.79)
Eastern Europe	All causes	39,787.17 (36,195.83–43,432.06)	49,556.04 (45,433.30–53,961.56)	48,928.17 (44,946.21–53,185.96)	42,330.11 (35,882.11–49,722.46)
Eastern Europe	Group I	2,927.06 (2,710.76–3,181.38)	3,364.28 (3,108.11–3,640.88)	3,411.42 (3,158.08–3,675.27)	2,774.13 (2,419.52–3,147.63)
Eastern Europe	NCD	29,917.88 (27,110.74–32,807.30)	37,010.28 (33,905.50–40,468.18)	37,374.59 (34,168.79–40,781.32)	33,521.59 (28,296.50–39,384.05)
Eastern Europe	Injuries	6,942.24 (6,234.32–7,712.42)	9,181.48 (8,249.98–10,143.20)	8,142.16 (7,316.67–9,059.45)	6,034.39 (4,774.17–7,561.57)
Belarus	All causes	37,153.67 (33,797.98–40,746.25)	44,036.04 (40,264.74–47,813.43)	44,665.91 (41,062.90–48,749.37)	38,099.25 (32,678.71–43,257.16)
Belarus	Group I	2,526.84 (2,203.26–2,898.17)	2,131.96 (1,909.04–2,401.05)	1,994.87 (1,800.84–2,238.61)	1,523.24 (1,324.81–1,754.28)
Belarus	NCD	28,619.82 (25,894.53–31,546.55)	34,280.92 (31,218.38–37,256.71)	35,360.01 (32,474.30–38,499.59)	31,133.16 (26,827.35–35,349.14)
Belarus	Injuries	6,007.01 (5,407.52–6,673.12)	7,623.16 (6,930.65–8,375.73)	7,311.04 (6,635.16–8,095.42)	5,442.85 (4,475.82–6,503.91)
Estonia	All causes	39,795.07 (36,339.94–43,582.88)	41,561.65 (37,959.11–45,466.56)	38,937.40 (35,077.26–42,809.36)	33,670.04 (29,552.99–38,207.92)

Appendix Table 4. GBD 2016 age-standardized rate of DALYs (per 100,000) for all causes and level 1 cause groups by location for 1990, 2000, 2006 & 2016, both sexes combined.

	Cause	1990	2000	2006	2016
Estonia	Group I	2,281.55 (2,070.41–2,523.81)	2,038.72 (1,843.07–2,271.80)	1,728.31 (1,533.79–1,944.97)	1,242.59 (1,060.18–1,463.61)
Estonia	NCD	30,201.26 (27,440.00–33,285.76)	32,525.94 (29,567.53–35,725.49)	31,634.54 (28,437.28–34,883.32)	28,544.83 (25,109.78–32,380.48)
Estonia	Injuries	7,312.26 (6,680.37–8,051.50)	6,996.98 (6,326.94–7,765.64)	5,574.56 (4,982.98–6,272.03)	3,882.62 (3,205.96–4,699.86)
Latvia	All causes	41,660.99 (38,159.45–45,488.65)	41,983.50 (38,179.78–46,012.13)	44,547.08 (40,794.57–48,459.06)	39,028.01 (34,430.67–43,833.26)
Latvia	Group I	2,412.59 (2,213.94–2,648.89)	2,122.02 (1,924.35–2,368.76)	2,071.21 (1,871.59–2,304.69)	1,626.08 (1,411.24–1,853.91)
Latvia	NCD	31,423.88 (28,634.35–34,401.63)	32,543.99 (29,487.79–35,760.81)	35,874.04 (32,777.49–39,040.23)	32,693.82 (28,895.72–36,531.24)
Latvia	Injuries	7,824.53 (7,143.96–8,585.72)	7,317.49 (6,579.83–8,131.64)	6,601.84 (5,990.26–7,311.68)	4,708.11 (4,007.46–5,541.16)
Lithuania	All causes	36,244.55 (32,992.15–39,790.35)	36,965.22 (33,466.28–40,534.76)	42,254.05 (38,602.61–46,215.01)	38,544.72 (34,590.76–43,050.56)
Lithuania	Group I	1,992.34 (1,794.97–2,235.73)	1,860.54 (1,667.36–2,091.54)	2,004.96 (1,807.94–2,228.96)	1,539.85 (1,357.70–1,750.15)
Lithuania	NCD	27,327.32 (24,641.60–30,156.65)	28,025.86 (25,176.02–30,936.82)	32,824.52 (29,884.24–36,066.86)	31,398.38 (28,076.62–34,929.82)
Lithuania	Injuries	6,924.89 (6,299.40–7,643.81)	7,078.82 (6,434.34–7,803.20)	7,424.57 (6,715.25–8,202.66)	5,606.50 (4,926.07–6,470.36)
Moldova	All causes	40,237.78 (36,604.90–43,977.04)	38,942.65 (35,230.87–42,865.05)	38,785.80 (35,137.57–42,551.46)	35,689.27 (31,434.22–39,949.05)
Moldova	Group I	5,112.06 (4,457.38–5,905.86)	4,167.13 (3,663.09–4,732.71)	3,474.76 (3,060.83–3,902.26)	2,564.14 (2,196.97–3,003.17)
Moldova	NCD	28,569.80 (25,780.48–31,347.28)	29,450.07 (26,582.76–32,584.73)	30,526.36 (27,573.14–33,705.07)	29,176.57 (25,777.03–32,780.48)
Moldova	Injuries	6,555.91 (5,957.68–7,226.30)	5,325.45 (4,788.72–5,925.53)	4,784.68 (4,266.99–5,358.05)	3,948.56 (3,344.57–4,588.31)
Russia	All causes	39,847.44 (35,774.32–43,653.18)	51,318.51 (46,728.52–56,195.41)	49,706.08 (44,931.32–54,648.90)	43,056.00 (34,476.25–53,258.58)
Russia	Group I	3,054.60 (2,824.10–3,325.28)	3,612.11 (3,315.24–3,928.75)	3,581.99 (3,271.56–3,891.92)	2,998.94 (2,552.72–3,492.20)
Russia	NCD	29,425.76 (26,299.84–32,445.51)	37,521.66 (34,012.86–41,262.31)	37,260.95 (33,582.59–40,942.24)	33,506.89 (26,852.18–41,394.44)
Russia	Injuries	7,367.07 (6,525.21–8,247.17)	10,184.74 (9,040.07–11,400.72)	8,863.14 (7,837.14–9,967.31)	6,550.17 (4,929.28–8,601.66)
Ukraine	All causes	40,253.39 (36,789.54–44,083.76)	47,847.54 (43,931.34–52,021.01)	49,291.96 (45,376.89–53,386.33)	42,117.39 (35,402.50–50,181.92)
Ukraine	Group I	2,568.20 (2,252.64–2,939.36)	3,015.22 (2,763.15–3,344.63)	3,386.21 (3,129.77–3,677.07)	2,507.34 (2,153.64–2,902.09)
Ukraine	NCD	31,799.71 (28,906.11–35,099.39)	37,684.38 (34,480.32–41,168.14)	39,306.60 (36,117.59–42,825.04)	34,769.85 (29,195.10–41,516.27)
Ukraine	Injuries	5,885.48 (5,273.27–6,574.69)	7,147.93 (6,432.84–7,896.75)	6,599.15 (5,951.58–7,327.10)	4,840.20 (3,760.74–6,023.96)
Central Europe	All causes	35,485.08 (32,426.36–39,002.84)	34,339.82 (31,162.91–37,864.14)	33,872.20 (30,650.16–37,590.49)	32,885.80 (29,479.60–36,709.30)
Central Europe	Group I	3,131.65 (2,906.86–3,415.85)	2,025.91 (1,824.44–2,259.93)	1,697.02 (1,508.81–1,927.42)	1,370.06 (1,203.28–1,574.35)
Central Europe	NCD	27,801.49 (25,312.60–30,530.57)	28,248.01 (25,609.57–31,076.25)	28,336.77 (25,664.12–31,339.98)	27,976.44 (25,160.70–31,121.25)
Central Europe	Injuries	4,551.93 (4,052.72–5,153.32)	4,065.89 (3,581.05–4,683.80)	3,838.40 (3,332.82–4,470.19)	3,539.29 (2,965.83–4,258.33)
Albania	All causes	27,821.68 (24,896.17–30,858.09)	25,452.73 (22,771.55–28,434.73)	25,445.50 (22,600.47–28,613.97)	26,586.69 (23,044.35–30,235.79)
Albania	Group I	8,603.27 (7,340.38–10,062.59)	3,921.98 (3,342.04–4,535.56)	2,383.00 (2,007.58–2,871.21)	1,789.89 (1,376.79–2,319.58)
Albania	NCD	16,081.01 (13,952.03–18,209.31)	18,140.60 (16,001.58–20,502.49)	20,467.26 (18,160.37–23,039.50)	22,260.91 (19,381.80–25,280.55)
Albania	Injuries	3,137.40 (2,795.57–3,552.27)	3,390.15 (2,865.31–3,893.93)	2,595.24 (2,215.63–3,082.29)	2,535.89 (2,066.50–3,126.74)
Bosnia and Herzegovina	All causes	28,418.08 (25,011.85–31,857.78)	28,873.36 (25,329.19–32,690.36)	29,902.12 (26,308.51–33,799.93)	31,395.05 (27,231.37–36,049.46)
Bosnia and Herzegovina	Group I	3,290.56 (2,926.56–3,712.91)	1,754.34 (1,508.32–2,050.68)	1,477.69 (1,253.72–1,767.69)	1,178.30 (969.92–1,425.70)
Bosnia and Herzegovina	NCD	22,005.86 (19,239.61–24,868.78)	24,432.96 (21,562.17–27,514.04)	25,823.62 (22,830.59–28,983.25)	27,457.14 (23,931.35–31,296.39)
Bosnia and Herzegovina	Injuries	3,121.66 (2,619.27–3,650.00)	2,686.07 (2,191.17–3,305.13)	2,600.81 (2,082.77–3,238.38)	2,759.61 (2,157.81–3,544.06)
Bulgaria	All causes	37,844.88 (34,620.02–41,434.21)	40,360.91 (36,967.53–44,184.01)	40,666.16 (37,143.02–44,575.41)	39,097.88 (34,032.81–44,367.02)
Bulgaria	Group I	2,705.00 (2,484.97–2,970.08)	2,092.85 (1,897.75–2,328.84)	1,785.58 (1,602.12–2,003.62)	1,424.16 (1,200.94–1,699.49)
Bulgaria	NCD	30,841.28 (28,229.49–33,858.51)	34,297.70 (31,533.46–37,389.74)	35,171.95 (32,226.47–38,350.61)	34,330.56 (29,882.89–38,820.06)
Bulgaria	Injuries	4,298.61 (3,757.16–4,924.82)	3,970.36 (3,457.37–4,583.50)	3,708.62 (3,162.87–4,358.71)	3,343.16 (2,667.91–4,131.03)
Croatia	All causes	35,243.92 (31,735.48–39,047.93)	35,285.36 (31,761.58–39,060.36)	34,505.78 (30,835.24–38,391.01)	33,923.86 (29,810.35–38,278.16)
Croatia	Group I	1,816.86 (1,667.27–1,988.85)	1,471.25 (1,319.94–1,637.48)	1,207.56 (1,070.08–1,369.17)	887.58 (767.83–1,033.65)
Croatia	NCD	28,653.25 (25,721.24–31,705.15)	30,050.90 (27,000.26–33,268.84)	29,713.77 (26,556.69–33,023.90)	29,611.95 (26,079.78–33,312.66)
Croatia	Injuries	4,773.81 (4,223.72–5,440.05)	3,763.20 (3,280.92–4,312.43)	3,584.45 (3,094.69–4,196.55)	3,424.33 (2,837.99–4,173.82)
Czech Republic	All causes	36,480.72 (33,361.55–40,229.14)	32,211.70 (28,914.14–36,011.24)	31,595.14 (28,084.71–35,452.81)	30,032.88 (26,326.72–34,294.74)
Czech Republic	Group I	1,762.66 (1,587.72–1,969.73)	1,223.11 (1,067.53–1,424.17)	1,214.64 (1,062.06–1,418.12)	1,106.38 (945.66–1,302.89)
Czech Republic	NCD	30,058.24 (27,490.38–33,030.77)	26,953.91 (24,249.66–29,956.93)	26,371.45 (23,554.93–29,417.80)	25,307.52 (22,260.63–28,633.37)
Czech Republic	Injuries	4,659.82 (4,061.95–5,371.12)	4,034.67 (3,469.89–4,741.89)	4,009.05 (3,364.73–4,797.68)	3,618.98 (2,932.28–4,483.35)
Hungary	All causes	42,988.96 (39,298.63–47,023.77)	39,927.18 (36,373.97–43,955.60)	38,454.19 (34,753.86–42,510.50)	36,465.16 (31,950.32–41,083.82)
Hungary	Group I	2,269.26 (2,061.54–2,526.80)	1,490.33 (1,302.36–1,723.01)	1,228.02 (1,047.93–1,445.82)	1,085.24 (891.34–1,303.03)
Hungary	NCD	34,891.45 (31,827.01–38,007.05)	33,799.08 (30,850.28–37,140.69)	33,060.40 (30,011.01–36,466.53)	31,703.90 (28,025.31–35,455.21)
Hungary	Injuries	5,828.25 (5,119.26–6,616.88)	4,637.77 (4,051.29–5,366.77)	4,165.76 (3,600.26–4,844.53)	3,676.02 (3,028.68–4,461.69)
Macedonia	All causes	31,400.37 (28,496.77–34,549.59)	30,073.94 (27,148.22–33,378.35)	30,673.52 (27,544.64–33,940.99)	31,368.21 (28,139.59–35,011.09)
Macedonia	Group I	5,145.33 (4,714.27–5,628.96)	2,341.33 (2,069.63–2,642.93)	1,823.55 (1,601.94–2,095.91)	1,479.32 (1,170.46–1,855.62)
Macedonia	NCD	23,245.77 (20,862.65–25,760.06)	25,017.88 (22,495.51–27,711.86)	26,300.47 (23,707.21–29,088.30)	27,289.67 (24,655.80–30,356.70)
Macedonia	Injuries	3,009.27 (2,610.38–3,480.53)	2,714.73 (2,352.90–3,185.93)	2,549.51 (2,158.38–3,067.71)	2,599.22 (2,124.61–3,186.20)
Montenegro	All causes	26,457.59 (23,415.41–29,912.47)	30,661.97 (27,648.99–34,100.03)	29,912.06 (26,704.00–33,432.78)	29,332.73 (25,722.46–33,148.42)
Montenegro	Group I	2,504.24 (2,023.88–3,050.08)	2,362.56 (2,129.17–2,653.99)	1,649.91 (1,454.47–1,880.82)	1,150.08 (945.14–1,403.13)
Montenegro	NCD	20,621.32 (18,228.82–23,294.76)	24,818.26 (22,316.92–27,660.10)	24,968.61 (22,290.25–27,886.24)	24,974.85 (22,073.77–28,214.10)
Montenegro	Injuries	3,332.04 (2,882.47–3,883.44)	3,481.15 (3,041.60–4,021.73)	3,293.54 (2,834.56–3,884.28)	3,207.80 (2,672.18–3,868.31)
Poland	All causes	34,634.75 (31,553.93–38,045.59)	31,747.14 (28,607.87–35,262.89)	31,594.40 (28,351.95–35,250.80)	30,660.13 (26,818.20–34,672.26)
Poland	Group I	2,749.06 (2,520.50–3,029.18)	1,563.68 (1,392.40–1,774.98)	1,427.30 (1,261.25–1,633.20)	1,224.11 (1,046.68–1,444.76)
Poland	NCD	27,309.32 (24,868.09–30,042.60)	25,962.88 (23,400.11–28,747.91)	26,054.01 (23,412.45–29,036.32)	25,710.80 (22,550.92–28,978.67)
Poland	Injuries	4,576.37 (4,082.82–5,160.22)	4,220.58 (3,718.28–4,879.62)	4,113.09 (3,584.11–4,802.70)	3,725.22 (3,100.19–4,480.55)
Romania	All causes	37,445.32 (34,183.73–41,127.96)	37,728.01 (34,252.62–41,371.90)	36,969.11 (33,496.44–40,829.35)	36,513.95 (32,318.86–40,822.82)
Romania	Group I	4,651.22 (4,324.02–5,061.02)	3,438.35 (3,148.65–3,807.21)	2,767.15 (2,489.33–3,081.04)	2,074.23 (1,809.72–2,406.53)
Romania	NCD	27,901.43 (25,311.82–30,676.36)	30,053.25 (27,231.91–33,049.45)	30,256.03 (27,468.73–33,414.37)	30,817.88 (27,392.16–34,230.90)
Romania	Injuries	4,892.67 (4,311.81–5,560.18)	4,236.41 (3,680.76–4,870.95)	3,945.93 (3,410.38–4,611.34)	3,621.84 (3,007.65–4,351.84)
Serbia	All causes	31,793.18 (28,460.10–35,480.20)	36,173.61 (32,818.86–39,898.51)	35,468.51 (31,980.05–39,146.37)	34,233.59 (30,627.44–38,228.00)
Serbia	Group I	2,642.01 (2,211.63–3,176.62)	1,936.91 (1,677.10–2,243.16)	1,584.02 (1,367.86–1,838.65)	1,305.59 (1,120.48–1,547.23)
Serbia	NCD	25,548.80 (22,728.59–28,498.42)	30,487.81 (27,636.76–33,446.77)	30,563.97 (27,689.12–33,706.37)	29,586.71 (26,548.47–32,838.12)
Serbia	Injuries	3,602.38 (3,167.48–4,139.00)	3,748.89 (3,213.90–4,332.99)	3,320.51 (2,830.91–3,972.26)	3,341.29 (2,807.25–4,044.33)

Appendix Table 4. GBD 2016 age-standardized rate of DALYs (per 100,000) for all causes and level 1 cause groups by location for 1990, 2000, 2006 & 2016, both sexes combined.					
	Cause	1990	2000	2006	2016
Slovakia	All causes	33,976.04 (30,981.97–37,464.36)	31,474.28 (28,168.83–34,964.16)	31,520.31 (28,289.10–35,228.97)	30,535.92 (26,518.28–34,857.76)
Slovakia	Group I	2,620.59 (2,341.13–2,889.52)	1,735.17 (1,536.50–1,974.52)	1,612.75 (1,419.25–1,850.28)	1,393.06 (1,172.97–1,632.82)
Slovakia	NCD	26,769.95 (24,398.49–29,562.29)	25,850.06 (23,159.26–28,703.30)	26,263.52 (23,591.61–29,252.84)	25,661.20 (22,355.91–29,116.41)
Slovakia	Injuries	4,585.49 (4,051.93–5,225.71)	3,889.05 (3,388.86–4,494.59)	3,644.04 (3,139.64–4,252.27)	3,481.66 (2,872.31–4,224.40)
Slovenia	All causes	31,770.72 (28,231.09–35,617.93)	31,193.30 (27,497.46–35,155.25)	30,288.42 (26,395.83–34,518.60)	28,779.71 (24,414.51–33,510.28)
Slovenia	Group I	1,589.91 (1,408.64–1,817.39)	1,291.26 (1,118.56–1,499.74)	1,125.72 (963.77–1,330.07)	954.88 (801.81–1,145.75)
Slovenia	NCD	24,776.53 (21,976.20–27,701.66)	25,194.62 (22,139.61–28,298.92)	24,573.14 (21,495.72–27,749.09)	23,800.76 (20,358.39–27,377.41)
Slovenia	Injuries	5,404.27 (4,739.10–6,230.05)	4,707.41 (4,038.97–5,533.74)	4,589.55 (3,849.74–5,487.90)	4,024.08 (3,253.99–4,984.24)
Central Asia	All causes	39,869.49 (37,027.11–42,940.88)	37,322.21 (34,469.78–40,334.44)	36,160.28 (33,418.48–39,441.51)	30,554.47 (27,467.62–33,714.25)
Central Asia	Group I	15,018.33 (14,130.60–16,023.12)	10,414.92 (9,679.08–11,279.87)	8,513.64 (7,695.87–9,550.88)	5,877.69 (4,969.81–6,980.57)
Central Asia	NCD	19,930.69 (18,036.60–22,067.93)	22,339.90 (20,317.22–24,518.94)	23,159.15 (21,049.64–25,390.96)	21,069.83 (18,735.98–23,445.33)
Central Asia	Injuries	4,920.47 (4,575.53–5,317.32)	4,567.40 (4,226.58–4,947.28)	4,487.49 (4,120.43–4,874.53)	3,606.95 (3,224.84–4,041.25)
Armenia	All causes	33,183.50 (30,099.76–36,419.21)	30,552.00 (27,672.28–33,725.11)	31,494.67 (28,430.20–34,849.07)	28,928.98 (25,609.36–32,557.02)
Armenia	Group I	8,328.11 (7,352.09–9,535.26)	3,737.91 (3,248.33–4,258.93)	3,048.78 (2,569.88–3,590.57)	2,069.95 (1,709.68–2,497.27)
Armenia	NCD	20,299.97 (18,220.51–22,598.34)	23,637.64 (21,218.39–26,195.04)	25,467.56 (23,025.99–28,214.50)	24,231.49 (21,416.98–27,284.41)
Armenia	Injuries	4,555.42 (4,044.59–5,191.75)	3,176.44 (2,744.28–3,751.05)	2,978.32 (2,560.15–3,541.27)	2,627.54 (2,181.16–3,253.56)
Azerbaijan	All causes	41,713.54 (37,637.52–45,790.35)	34,693.94 (31,623.17–38,117.67)	34,257.58 (30,748.06–38,082.98)	30,742.25 (26,385.19–35,299.16)
Azerbaijan	Group I	17,465.89 (14,914.85–20,253.47)	10,330.30 (9,109.36–11,761.77)	8,921.51 (7,473.25–10,665.70)	5,589.98 (4,109.30–7,362.63)
Azerbaijan	NCD	20,105.48 (17,938.53–22,309.36)	21,276.84 (19,082.18–23,638.82)	22,528.63 (20,065.36–25,067.24)	22,422.73 (19,294.21–25,813.44)
Azerbaijan	Injuries	4,142.17 (3,689.65–4,639.32)	3,086.81 (2,762.57–3,482.36)	2,807.44 (2,450.80–3,217.70)	2,729.54 (2,259.15–3,274.08)
Georgia	All causes	36,769.32 (33,631.83–40,399.25)	33,313.62 (29,967.43–36,788.19)	34,967.35 (31,590.96–38,623.98)	34,470.18 (29,890.25–39,386.99)
Georgia	Group I	7,211.83 (6,465.73–8,047.80)	4,828.63 (4,265.74–5,470.34)	4,062.72 (3,467.39–4,774.35)	3,078.09 (2,535.40–3,754.99)
Georgia	NCD	25,423.33 (22,841.70–28,214.15)	25,778.65 (23,074.73–28,471.95)	27,908.51 (25,200.73–30,864.03)	27,980.47 (24,295.91–32,121.07)
Georgia	Injuries	4,134.17 (3,710.29–4,643.27)	2,706.34 (2,359.32–3,113.41)	2,996.11 (2,613.35–3,452.74)	3,411.61 (2,814.66–4,102.94)
Kazakhstan	All causes	36,637.41 (33,168.17–40,393.49)	42,948.27 (38,928.31–47,153.07)	43,536.51 (39,353.79–47,969.90)	31,178.05 (26,995.68–35,970.91)
Kazakhstan	Group I	8,121.68 (7,222.93–9,038.58)	7,179.20 (6,351.70–8,107.39)	6,386.20 (5,525.43–7,324.34)	3,654.77 (3,015.80–4,421.14)
Kazakhstan	NCD	22,494.16 (20,132.53–25,164.85)	28,871.88 (26,014.43–31,784.45)	29,449.41 (26,566.96–32,425.91)	22,675.24 (19,533.50–26,241.56)
Kazakhstan	Injuries	6,021.57 (5,426.32–6,690.92)	6,897.18 (6,175.26–7,703.01)	7,700.91 (6,913.99–8,550.41)	4,848.05 (4,081.99–5,758.84)
Kyrgyzstan	All causes	43,166.60 (40,142.23–46,293.28)	36,208.00 (33,478.08–39,146.03)	35,338.60 (32,652.26–38,383.51)	29,301.64 (26,045.57–32,465.31)
Kyrgyzstan	Group I	17,023.56 (15,757.40–18,404.31)	10,459.61 (9,675.53–11,298.36)	8,865.43 (8,104.95–9,718.19)	7,230.53 (6,158.83–8,474.03)
Kyrgyzstan	NCD	20,395.50 (18,443.55–22,482.97)	21,199.46 (19,154.07–23,314.50)	22,269.63 (20,217.04–24,425.19)	18,622.84 (16,325.46–21,050.59)
Kyrgyzstan	Injuries	5,747.53 (5,272.15–6,222.68)	4,548.93 (4,166.91–4,921.15)	4,203.54 (3,870.11–4,582.96)	3,448.28 (3,054.34–3,892.94)
Mongolia	All causes	51,152.56 (47,293.08–55,446.93)	38,824.75 (35,895.64–42,251.18)	37,061.33 (33,905.60–40,346.59)	33,666.53 (29,836.91–38,326.00)
Mongolia	Group I	24,442.99 (21,979.36–27,342.24)	11,286.23 (10,132.29–12,533.98)	7,842.95 (6,957.79–8,799.45)	5,506.75 (4,533.48–6,619.56)
Mongolia	NCD	20,956.95 (18,671.18–23,459.44)	21,884.58 (19,749.69–24,215.49)	23,208.90 (20,882.72–25,696.62)	22,760.04 (19,954.16–25,971.29)
Mongolia	Injuries	5,752.62 (5,110.72–6,463.76)	5,653.94 (4,989.72–6,228.43)	6,009.48 (5,233.04–6,696.73)	5,399.74 (4,567.44–6,305.51)
Tajikistan	All causes	53,677.54 (48,482.43–59,034.29)	40,156.24 (36,178.26–44,754.76)	32,225.49 (29,043.99–35,993.30)	28,652.10 (24,036.75–34,100.90)
Tajikistan	Group I	31,723.20 (27,804.18–35,962.27)	19,448.94 (16,720.51–22,720.64)	12,838.22 (11,133.56–14,940.60)	10,492.57 (7,399.44–14,454.95)
Tajikistan	NCD	17,143.13 (15,136.10–19,179.42)	16,463.15 (14,607.11–18,493.17)	16,028.96 (14,073.80–18,140.68)	15,088.42 (12,969.79–17,371.45)
Tajikistan	Injuries	4,811.21 (4,259.56–5,577.92)	4,244.14 (3,781.55–4,740.75)	3,358.32 (2,995.31–3,808.93)	3,071.11 (2,597.99–3,592.72)
Turkmenistan	All causes	50,513.73 (45,474.97–55,634.39)	42,194.35 (37,289.91–48,016.21)	37,964.67 (33,715.19–42,864.83)	30,787.39 (27,128.95–34,716.09)
Turkmenistan	Group I	25,526.57 (21,932.00–29,479.28)	16,254.66 (13,026.84–20,499.86)	11,585.21 (8,876.77–14,764.02)	6,812.41 (5,103.27–9,157.90)
Turkmenistan	NCD	19,547.53 (17,768.91–21,563.53)	21,084.78 (19,226.07–23,106.97)	21,949.56 (19,950.34–24,169.61)	20,937.00 (18,626.06–23,384.50)
Turkmenistan	Injuries	5,439.63 (4,929.08–6,013.24)	4,854.91 (4,360.13–5,406.12)	4,429.90 (3,961.33–4,955.68)	3,037.98 (2,632.05–3,504.58)
Uzbekistan	All causes	36,531.35 (33,014.14–40,273.50)	34,843.25 (31,711.12–38,316.16)	33,933.31 (30,171.06–38,150.84)	30,201.33 (26,322.21–34,698.86)
Uzbekistan	Group I	15,442.82 (13,368.58–17,639.73)	10,943.91 (9,548.62–12,576.01)	9,320.80 (7,159.11–11,784.39)	6,327.94 (4,453.26–9,119.61)
Uzbekistan	NCD	16,842.75 (15,024.38–18,899.73)	19,816.91 (17,795.13–21,848.94)	20,835.29 (18,635.25–23,175.29)	20,481.75 (18,113.92–23,040.53)
Uzbekistan	Injuries	4,245.79 (3,867.71–4,706.20)	4,082.43 (3,701.21–4,480.00)	3,777.23 (3,355.10–4,234.73)	3,391.64 (2,942.38–3,865.04)
Latin America and Caribbean	All causes	34,344.30 (31,936.33–37,068.31)	28,308.35 (25,814.56–31,117.04)	26,485.49 (23,966.18–29,346.41)	25,295.99 (22,661.21–28,288.41)
Latin America and Caribbean	Group I	12,512.58 (11,933.39–13,183.80)	7,322.21 (6,949.46–7,792.74)	5,670.43 (5,341.96–6,097.16)	3,899.97 (3,604.41–4,266.95)
Latin America and Caribbean	NCD	16,648.23 (14,620.09–18,728.78)	16,537.26 (14,444.93–18,812.40)	16,732.39 (14,622.95–19,095.82)	17,586.35 (15,352.80–20,112.88)
Latin America and Caribbean	Injuries	5,183.49 (4,912.66–5,461.54)	4,448.87 (4,182.91–4,705.49)	4,082.67 (3,845.41–4,330.04)	3,809.68 (3,558.90–4,098.00)
Central Latin America	All causes	31,205.02 (29,041.31–33,727.90)	25,678.50 (23,466.75–28,247.11)	23,954.26 (21,654.75–26,590.61)	23,191.18 (20,677.36–25,957.46)
Central Latin America	Group I	11,044.48 (10,629.52–11,527.77)	6,308.38 (6,020.60–6,700.07)	4,699.76 (4,436.86–5,016.67)	3,168.70 (2,919.89–3,442.31)
Central Latin America	NCD	14,889.21 (13,085.79–16,907.85)	14,923.28 (13,051.67–17,070.86)	15,263.75 (13,309.58–17,521.02)	16,380.39 (14,240.12–18,791.82)
Central Latin America	Injuries	5,271.33 (4,970.03–5,543.64)	4,446.84 (4,170.80–4,698.34)	3,990.76 (3,736.86–4,243.36)	3,642.09 (3,352.68–3,965.78)
Colombia	All causes	29,255.17 (26,750.86–32,109.74)	26,313.19 (23,934.34–28,927.85)	23,579.76 (21,170.24–26,245.83)	21,191.99 (18,477.98–24,208.02)
Colombia	Group I	7,652.84 (6,776.25–8,587.90)	5,148.79 (4,674.33–5,697.99)	4,104.66 (3,680.76–4,567.96)	2,572.95 (2,250.09–2,950.84)
Colombia	NCD	14,323.82 (12,469.42–16,393.28)	14,395.45 (12,441.83–16,554.79)	14,625.26 (12,663.58–16,896.84)	14,858.14 (12,585.46–17,466.45)
Colombia	Injuries	7,278.51 (6,637.31–7,738.93)	6,768.95 (6,176.04–7,193.94)	4,849.84 (4,496.67–5,175.95)	3,760.89 (3,351.56–4,198.51)
Costa Rica	All causes	20,560.46 (18,249.67–23,121.00)	19,551.69 (17,203.15–22,213.72)	19,065.01 (16,608.36–21,917.54)	19,739.21 (16,951.56–22,896.02)
Costa Rica	Group I	4,081.28 (3,565.98–4,712.42)	2,720.21 (2,328.93–3,209.75)	2,222.97 (1,898.11–2,626.27)	1,678.56 (1,361.77–2,066.41)
Costa Rica	NCD	13,923.09 (11,975.04–16,121.73)	14,186.11 (12,164.77–16,459.09)	14,339.53 (12,242.33–16,795.58)	15,623.21 (13,254.06–18,254.09)
Costa Rica	Injuries	2,556.09 (2,380.03–2,757.81)	2,645.37 (2,467.15–2,866.76)	2,502.51 (2,306.08–2,722.49)	2,437.44 (2,157.27–2,746.63)
El Salvador	All causes	38,609.25 (35,631.24–41,744.51)	29,328.49 (26,755.58–32,252.49)	27,242.38 (24,689.61–30,194.92)	25,891.68 (22,920.52–29,392.35)
El Salvador	Group I	12,902.11 (11,488.70–14,544.37)	6,700.48 (5,928.43–7,582.73)	4,367.11 (3,791.44–5,092.79)	2,727.57 (2,271.57–3,276.78)
El Salvador	NCD	17,329.55 (15,118.17–19,574.50)	16,117.52 (14,171.55–18,442.22)	16,436.09 (14,351.63–18,783.45)	17,519.15 (15,085.39–20,245.58)
El Salvador	Injuries	8,377.58 (7,826.55–8,990.83)	6,510.49 (6,038.60–6,967.01)	6,439.18 (5,798.20–6,888.11)	5,644.96 (4,926.84–6,418.84)
Guatemala	All causes	50,903.74 (47,236.88–54,748.78)	37,745.33 (34,264.67–41,574.17)	33,140.19 (29,772.56–36,802.44)	27,400.31 (23,317.83–31,820.38)
Guatemala	Group I	29,535.71 (27,217.04–31,807.96)	17,925.68 (16,408.83–19,464.84)	12,796.04 (11,708.42–14,015.45)	7,234.49 (6,220.40–8,314.35)
Guatemala	NCD	15,882.56 (13,938.16–17,880.23)	14,906.24 (12,826.53–17,319.38)	15,032.96 (12,794.60–17,295.25)	15,426.86 (12,867.42–18,121.31)

Appendix Table 4. GBD 2016 age-standardized rate of DALYs (per 100,000) for all causes and level 1 cause groups by location for 1990, 2000, 2006 & 2016, both sexes combined.

	Cause	1990	2000	2006	2016
Guatemala	Injuries	5,485.47 (4,731.61–6,540.80)	4,913.41 (4,253.05–5,754.91)	5,311.19 (4,509.68–6,168.57)	4,738.97 (3,674.47–5,966.19)
Honduras	All causes	38,894.20 (35,314.43–42,462.98)	29,680.42 (25,917.35–33,621.66)	26,956.74 (23,151.61–30,959.21)	25,133.70 (21,335.71–29,570.52)
Honduras	Group I	16,487.33 (14,750.00–18,259.26)	9,165.44 (8,020.65–10,322.65)	6,720.00 (5,923.65–7,634.11)	4,752.16 (4,027.84–5,559.36)
Honduras	NCD	17,460.74 (15,300.50–19,536.91)	16,310.04 (13,801.54–18,876.84)	16,242.39 (13,570.40–19,026.46)	16,586.25 (13,746.50–19,704.22)
Honduras	Injuries	4,946.13 (3,988.32–5,879.90)	4,204.93 (3,234.47–5,274.32)	3,994.36 (3,051.11–5,109.62)	3,795.29 (2,808.43–5,079.32)
Mexico	All causes	30,800.16 (28,682.09–33,179.34)	24,434.79 (22,213.97–26,971.46)	22,991.87 (20,751.14–25,570.10)	23,057.72 (20,647.09–25,964.68)
Mexico	Group I	11,014.47 (10,471.40–11,456.18)	5,645.51 (5,376.72–5,996.81)	4,077.58 (3,823.76–4,378.33)	2,768.19 (2,552.40–3,035.77)
Mexico	NCD	15,050.39 (13,239.86–17,031.13)	15,308.36 (13,430.87–17,465.41)	15,758.09 (13,792.80–18,024.47)	17,257.22 (15,138.67–19,808.63)
Mexico	Injuries	4,735.30 (4,473.88–4,999.45)	3,480.93 (3,278.41–3,729.06)	3,156.21 (2,978.51–3,404.34)	3,032.31 (2,826.86–3,275.73)
Nicaragua	All causes	33,260.89 (30,813.65–35,791.43)	24,812.35 (22,529.82–27,157.42)	21,437.79 (19,174.12–23,795.65)	19,794.48 (17,106.24–22,980.11)
Nicaragua	Group I	16,238.26 (15,204.25–17,243.28)	8,463.38 (7,824.68–9,106.59)	5,431.53 (4,945.14–5,985.86)	2,908.20 (2,537.68–3,339.12)
Nicaragua	NCD	12,880.93 (11,044.87–14,842.88)	13,035.96 (11,190.51–15,008.22)	13,172.64 (11,233.06–15,287.43)	14,322.79 (12,100.18–16,867.64)
Nicaragua	Injuries	4,141.70 (3,783.06–4,545.45)	3,313.01 (3,063.41–3,594.00)	2,833.62 (2,621.13–3,088.22)	2,563.49 (2,172.55–3,034.86)
Panama	All causes	23,641.13 (21,257.52–26,328.01)	22,273.87 (19,823.21–24,947.38)	21,987.44 (19,665.77–24,708.04)	21,666.57 (18,605.21–24,985.81)
Panama	Group I	5,744.76 (5,022.45–6,497.92)	5,131.14 (4,574.61–5,888.15)	4,482.10 (3,954.02–5,197.15)	3,481.27 (2,916.53–4,189.92)
Panama	NCD	14,306.54 (12,417.84–16,487.52)	14,039.53 (12,002.03–16,324.06)	14,420.96 (12,442.95–16,659.93)	15,184.58 (12,883.97–17,776.80)
Panama	Injuries	3,589.83 (3,311.09–3,904.98)	3,103.21 (2,822.78–3,399.23)	3,084.38 (2,801.97–3,413.42)	3,000.72 (2,507.38–3,504.86)
Venezuela	All causes	25,514.25 (23,263.54–28,083.03)	23,828.41 (21,479.19–26,494.08)	24,000.45 (21,655.05–26,697.08)	24,952.56 (21,727.86–28,460.11)
Venezuela	Group I	7,306.06 (6,994.12–7,676.37)	4,933.30 (4,507.31–5,376.61)	4,035.23 (3,761.61–4,350.66)	3,496.94 (3,103.59–3,945.59)
Venezuela	NCD	14,044.76 (12,234.22–16,181.90)	14,149.65 (12,253.40–16,307.31)	14,530.89 (12,595.56–16,708.77)	16,034.51 (13,602.57–18,581.98)
Venezuela	Injuries	4,163.43 (3,855.62–4,537.92)	4,745.47 (4,333.70–5,165.15)	5,434.33 (4,841.50–5,992.40)	5,421.11 (4,471.39–6,724.84)
Andean Latin America	All causes	42,081.28 (39,353.29–44,901.62)	29,241.61 (26,738.88–31,932.05)	25,552.25 (23,069.62–28,371.35)	22,633.82 (19,971.11–25,652.24)
Andean Latin America	Group I	20,252.26 (18,962.04–21,593.27)	10,310.11 (9,702.97–11,014.69)	7,510.16 (6,947.16–8,127.87)	4,933.05 (4,412.94–5,474.37)
Andean Latin America	NCD	16,316.60 (14,310.06–18,509.42)	14,935.47 (12,929.62–17,124.15)	14,607.96 (12,602.12–16,904.04)	14,637.36 (12,462.70–17,062.84)
Andean Latin America	Injuries	5,512.42 (4,945.66–6,196.20)	3,996.03 (3,690.33–4,305.53)	3,434.13 (3,193.05–3,674.70)	3,063.40 (2,752.44–3,379.34)
Bolivia	All causes	62,243.94 (58,464.82–66,257.08)	42,706.10 (39,408.70–46,411.89)	35,517.72 (31,944.70–39,179.41)	29,057.30 (25,251.42–33,240.48)
Bolivia	Group I	34,509.35 (31,608.60–37,743.37)	19,389.92 (17,326.55–21,834.69)	14,001.22 (12,338.45–16,030.07)	8,453.50 (7,006.49–10,115.41)
Bolivia	NCD	20,573.37 (17,922.43–23,466.13)	18,183.11 (15,982.03–20,652.25)	17,358.59 (15,133.91–19,733.15)	17,146.00 (14,508.39–20,019.16)
Bolivia	Injuries	7,161.23 (5,957.38–8,327.94)	5,133.07 (4,534.26–5,810.17)	4,157.91 (3,667.88–4,678.12)	3,457.80 (2,873.38–4,080.06)
Ecuador	All causes	33,336.71 (30,581.24–36,408.19)	27,367.03 (24,835.49–30,262.01)	25,135.88 (22,535.06–28,053.72)	22,884.07 (20,302.56–25,863.33)
Ecuador	Group I	13,552.44 (12,316.12–14,836.98)	8,173.40 (7,356.68–9,020.05)	6,034.62 (5,398.41–6,741.64)	3,879.88 (3,412.22–4,403.67)
Ecuador	NCD	15,170.28 (13,218.03–17,180.03)	14,793.63 (12,724.24–17,023.69)	14,803.66 (12,771.99–17,058.36)	14,932.88 (12,763.78–17,287.59)
Ecuador	Injuries	4,613.98 (4,310.88–5,035.33)	4,400.00 (4,079.67–4,695.98)	4,297.60 (3,938.60–4,607.44)	4,071.31 (3,553.04–4,611.68)
Peru	All causes	39,826.74 (36,894.56–42,959.99)	25,778.84 (23,182.47–28,627.43)	22,408.12 (19,856.36–25,295.18)	20,300.59 (17,270.55–23,675.46)
Peru	Group I	18,903.83 (17,278.06–20,584.42)	8,405.67 (7,740.12–9,155.38)	6,071.97 (5,528.97–6,707.48)	4,265.66 (3,688.37–4,973.02)
Peru	NCD	15,507.61 (13,486.25–17,754.04)	13,947.09 (11,918.66–16,119.80)	13,582.86 (11,565.36–15,952.71)	13,624.56 (11,253.36–16,273.83)
Peru	Injuries	5,415.29 (4,589.59–6,330.44)	3,426.08 (3,052.84–3,784.55)	2,753.28 (2,490.51–3,029.26)	2,410.37 (2,052.62–2,820.80)
Caribbean	All causes	39,979.64 (36,519.54–43,537.94)	35,453.75 (32,079.09–39,275.84)	34,408.89 (31,241.39–38,074.81)	30,946.60 (27,580.75–34,788.92)
Caribbean	Group I	15,093.47 (13,341.25–16,904.88)	12,333.85 (10,727.63–14,188.14)	11,484.94 (10,232.55–12,899.30)	7,289.85 (6,126.43–8,860.02)
Caribbean	NCD	20,275.11 (18,055.22–22,591.24)	19,368.76 (17,109.99–21,819.84)	19,377.51 (17,082.17–21,903.93)	20,194.91 (17,646.74–22,903.82)
Caribbean	Injuries	4,611.06 (4,128.95–5,050.41)	3,751.13 (3,431.93–4,080.16)	3,546.44 (3,234.21–3,898.01)	3,461.84 (3,098.67–3,867.47)
Antigua and Barbuda	All causes	25,417.21 (22,454.69–28,527.51)	24,768.57 (21,660.49–28,145.72)	23,710.42 (20,647.04–27,151.08)	23,641.42 (20,398.72–27,233.54)
Antigua and Barbuda	Group I	3,918.29 (3,270.63–4,788.35)	4,792.35 (4,034.00–5,824.60)	3,790.80 (3,266.18–4,475.36)	3,023.69 (2,524.41–3,694.80)
Antigua and Barbuda	NCD	19,263.69 (16,912.24–21,729.44)	17,834.08 (15,472.33–20,428.14)	17,582.28 (15,146.11–20,288.55)	18,402.38 (15,775.24–21,222.14)
Antigua and Barbuda	Injuries	2,235.22 (1,995.22–2,517.98)	2,142.15 (1,887.20–2,443.01)	2,337.34 (2,062.62–2,644.65)	2,215.35 (1,886.88–2,557.42)
The Bahamas	All causes	28,717.82 (25,277.65–32,589.42)	28,869.33 (26,141.17–32,139.73)	27,839.40 (25,013.45–31,156.87)	29,585.48 (26,007.09–33,416.89)
The Bahamas	Group I	6,217.96 (4,793.28–8,273.82)	7,231.95 (6,592.22–7,985.87)	5,453.67 (4,902.21–6,070.85)	4,116.81 (3,455.06–5,254.64)
The Bahamas	NCD	18,390.54 (16,214.45–20,709.80)	18,305.74 (16,157.85–20,836.67)	18,958.92 (16,652.14–21,580.25)	21,891.33 (19,022.89–24,798.70)
The Bahamas	Injuries	4,109.31 (3,732.17–4,546.12)	3,331.64 (3,022.78–3,687.08)	3,426.81 (3,051.98–3,770.83)	3,577.34 (3,048.99–4,127.02)
Barbados	All causes	28,106.75 (25,072.75–31,432.19)	29,016.60 (25,649.09–32,745.87)	28,489.34 (25,332.67–32,070.81)	30,138.26 (26,499.86–34,121.97)
Barbados	Group I	4,013.39 (3,217.20–5,034.41)	4,547.46 (3,831.87–5,554.60)	3,764.15 (3,242.38–4,404.49)	3,390.69 (2,753.49–4,293.31)
Barbados	NCD	21,767.03 (19,213.84–24,475.58)	22,181.46 (19,425.05–25,190.74)	22,310.03 (19,644.79–25,282.06)	24,424.26 (21,377.34–27,701.68)
Barbados	Injuries	2,326.33 (2,069.25–2,634.51)	2,287.68 (2,030.07–2,603.44)	2,415.16 (2,116.46–2,719.48)	2,323.31 (1,992.92–2,697.49)
Belize	All causes	31,874.15 (28,542.48–35,696.11)	30,694.00 (27,416.44–34,326.55)	26,862.85 (23,564.21–30,507.12)	25,734.08 (21,891.39–30,494.17)
Belize	Group I	12,244.57 (10,445.89–14,315.45)	9,275.52 (7,809.75–10,886.66)	7,261.05 (6,002.81–8,849.12)	5,537.00 (4,140.50–7,653.11)
Belize	NCD	15,349.17 (13,482.96–17,414.14)	16,345.37 (14,321.71–18,521.99)	15,198.96 (13,160.52–17,373.27)	16,144.96 (13,696.25–18,713.98)
Belize	Injuries	4,280.41 (3,789.63–4,781.85)	5,073.11 (4,436.86–5,681.07)	4,402.85 (3,889.37–4,929.26)	4,052.12 (3,219.06–4,994.21)
Bermuda	All causes	29,216.45 (26,332.66–32,537.48)	25,939.91 (23,084.00–29,039.46)	23,273.93 (20,474.64–26,230.81)	21,087.42 (18,137.43–24,406.24)
Bermuda	Group I	2,976.67 (2,588.20–3,461.47)	3,357.07 (3,065.10–3,725.14)	2,593.43 (2,309.45–2,937.15)	1,896.57 (1,618.11–2,240.87)
Bermuda	NCD	23,332.07 (20,926.40–26,038.83)	20,737.55 (18,289.49–23,274.86)	18,834.41 (16,443.21–21,392.73)	17,503.18 (14,959.29–20,287.41)
Bermuda	Injuries	2,907.71 (2,608.31–3,222.88)	1,845.28 (1,576.52–2,135.91)	1,846.09 (1,626.96–2,105.47)	1,687.66 (1,420.16–2,014.92)
Cuba	All causes	26,584.72 (23,778.70–29,696.01)	25,265.05 (22,381.61–28,505.96)	24,890.89 (22,032.69–28,201.75)	26,840.41 (23,433.10–30,531.91)
Cuba	Group I	3,178.74 (2,824.81–3,638.40)	2,432.98 (2,104.83–2,847.58)	2,111.74 (1,791.10–2,502.00)	2,072.40 (1,768.18–2,451.62)
Cuba	NCD	19,458.25 (17,073.55–21,967.12)	19,765.22 (17,346.65–22,470.75)	20,260.46 (17,805.69–23,125.05)	22,336.01 (19,414.21–25,402.79)
Cuba	Injuries	3,947.73 (3,679.12–4,226.40)	3,066.85 (2,817.06–3,326.22)	2,518.70 (2,284.54–2,806.62)	2,432.01 (2,125.64–2,801.97)
Dominica	All causes	26,711.32 (23,819.23–29,832.62)	26,845.36 (23,910.32–29,855.09)	27,193.87 (24,321.25–30,545.82)	30,166.77 (26,379.45–34,367.60)
Dominica	Group I	4,636.50 (3,780.48–5,671.02)	4,807.34 (4,150.61–5,626.15)	4,335.29 (3,657.04–5,182.32)	4,471.66 (3,511.10–5,888.80)
Dominica	NCD	19,525.93 (17,288.22–22,020.70)	19,640.23 (17,319.44–22,191.38)	20,282.95 (17,956.53–22,915.10)	22,720.49 (19,820.87–25,827.97)
Dominica	Injuries	2,548.90 (2,298.74–2,799.22)	2,397.79 (2,150.63–2,685.45)	2,575.63 (2,332.33–2,862.46)	2,974.62 (2,526.27–3,493.22)
Dominican Republic	All causes	33,685.47 (30,279.60–37,178.49)	28,441.80 (25,730.91–31,634.11)	28,327.18 (25,316.43–31,716.83)	25,076.13 (21,750.55–28,500.63)
Dominican Republic	Group I	14,390.13 (12,445.09–16,439.97)	10,701.35 (9,386.53–12,225.77)	10,225.74 (8,823.44–11,739.67)	6,607.66 (5,557.48–7,867.32)

Appendix Table 4. GBD 2016 age-standardized rate of DALYs (per 100,000) for all causes and level 1 cause groups by location for 1990, 2000, 2006 & 2016, both sexes combined.

	Cause	1990	2000	2006	2016
Dominican Republic	NCD	15,778.94 (13,803.89–18,036.87)	14,544.51 (12,417.81–16,840.58)	14,847.47 (12,659.65–17,243.66)	15,461.50 (12,916.78–18,007.94)
Dominican Republic	Injuries	3,516.40 (3,201.04–3,986.29)	3,195.93 (2,898.93–3,501.02)	3,253.97 (2,918.24–3,651.48)	3,006.97 (2,547.73–3,513.53)
Grenada	All causes	30,315.93 (26,405.51–35,060.97)	28,338.25 (25,349.98–31,646.06)	29,148.72 (26,018.54–32,914.67)	30,182.12 (26,380.26–34,212.52)
Grenada	Group I	6,655.39 (5,034.68–9,225.56)	4,934.57 (4,102.44–5,917.22)	4,309.05 (3,633.74–5,291.54)	4,246.05 (3,436.47–5,532.19)
Grenada	NCD	20,745.88 (18,298.48–23,397.63)	20,299.85 (18,070.13–22,796.39)	21,425.56 (18,999.86–24,256.20)	22,476.48 (19,551.26–25,620.60)
Grenada	Injuries	2,914.66 (2,504.25–3,372.94)	3,103.83 (2,664.24–3,521.64)	3,414.10 (2,898.68–3,914.57)	3,459.59 (2,849.68–4,104.63)
Guyana	All causes	42,066.44 (38,017.13–46,094.22)	39,016.99 (35,640.27–42,745.53)	39,039.77 (35,729.97–42,624.50)	36,290.92 (32,518.83–40,409.49)
Guyana	Group I	16,125.76 (13,659.35–18,842.89)	12,985.05 (11,529.94–14,568.96)	11,092.65 (9,997.70–12,258.11)	7,445.55 (6,414.94–8,528.26)
Guyana	NCD	20,559.02 (18,390.71–22,876.62)	20,500.84 (18,400.17–22,887.97)	21,966.89 (19,624.71–24,464.80)	23,341.55 (20,579.40–26,304.02)
Guyana	Injuries	5,381.65 (4,981.08–5,852.28)	5,531.10 (5,077.15–6,029.55)	5,980.23 (5,424.27–6,493.23)	5,503.81 (4,856.01–6,131.99)
Haiti	All causes	84,846.81 (74,562.86–95,203.08)	66,452.49 (57,152.40–76,600.45)	61,209.70 (54,042.14–69,128.31)	45,378.88 (37,994.81–54,588.80)
Haiti	Group I	46,795.37 (38,701.00–55,085.61)	36,026.66 (28,807.80–44,150.19)	32,465.29 (27,479.00–38,282.44)	17,518.36 (13,149.01–23,767.85)
Haiti	NCD	28,986.64 (25,867.31–32,210.53)	24,110.82 (21,393.56–27,092.11)	23,101.81 (20,152.63–26,146.70)	22,355.67 (18,841.79–25,960.68)
Haiti	Injuries	9,064.80 (6,943.33–10,874.05)	6,315.01 (5,129.07–7,460.43)	5,642.60 (4,603.26–6,821.45)	5,504.85 (4,480.70–6,578.58)
Jamaica	All causes	27,774.73 (24,129.67–32,147.66)	27,790.24 (24,460.47–31,723.62)	26,619.09 (23,156.70–30,616.02)	28,025.39 (23,814.39–32,427.74)
Jamaica	Group I	8,019.13 (6,193.27–10,713.78)	6,848.90 (5,510.18–8,674.46)	5,475.27 (4,277.32–7,411.91)	4,421.38 (3,267.55–6,037.62)
Jamaica	NCD	17,772.41 (15,541.28–20,263.31)	18,701.36 (16,390.45–21,261.63)	18,610.41 (16,173.00–21,271.15)	20,848.63 (17,783.57–24,112.36)
Jamaica	Injuries	1,983.19 (1,777.70–2,238.69)	2,239.98 (1,987.67–2,538.84)	2,533.41 (2,195.10–2,859.58)	2,755.38 (2,242.11–3,298.71)
Puerto Rico	All causes	28,759.25 (25,966.98–31,835.77)	28,238.94 (25,138.55–31,681.12)	27,358.28 (24,247.41–30,953.54)	27,103.32 (23,850.89–30,837.41)
Puerto Rico	Group I	4,675.08 (4,366.57–5,035.77)	3,549.07 (3,247.11–3,892.86)	2,963.81 (2,691.11–3,294.88)	2,393.59 (2,126.02–2,733.03)
Puerto Rico	NCD	20,315.46 (17,940.92–22,948.56)	21,328.69 (18,675.55–24,253.36)	21,193.39 (18,521.67–24,226.52)	21,747.74 (18,933.27–24,856.22)
Puerto Rico	Injuries	3,768.71 (3,420.57–4,125.85)	3,361.17 (3,008.62–3,720.79)	3,201.08 (2,865.40–3,564.09)	2,961.99 (2,579.99–3,391.69)
Saint Lucia	All causes	29,058.26 (25,103.47–33,609.38)	26,021.30 (23,083.31–29,300.18)	26,081.30 (23,152.21–29,486.78)	26,739.54 (23,662.10–30,474.60)
Saint Lucia	Group I	7,020.43 (5,147.41–9,620.13)	4,898.61 (3,954.52–6,180.60)	4,211.19 (3,419.79–5,226.39)	3,434.61 (2,702.83–4,360.96)
Saint Lucia	NCD	18,892.75 (16,714.71–21,163.64)	18,049.84 (15,793.66–20,511.01)	18,589.48 (16,338.70–21,145.51)	20,098.69 (17,655.95–22,943.98)
Saint Lucia	Injuries	3,145.07 (2,819.71–3,547.90)	3,072.85 (2,819.72–3,383.31)	3,280.62 (2,974.46–3,622.85)	3,206.24 (2,853.23–3,600.24)
Saint Vincent and the Grenadines	All causes	29,157.36 (25,258.09–34,077.50)	30,793.32 (27,640.19–34,420.97)	29,875.95 (26,714.31–33,146.42)	31,459.00 (27,908.28–35,054.76)
Saint Vincent and the Grenadines	Group I	7,533.20 (5,510.93–10,166.30)	7,308.59 (6,168.66–8,780.26)	5,751.45 (4,935.07–6,732.70)	4,718.82 (3,987.92–5,750.21)
Saint Vincent and the Grenadines	NCD	18,494.61 (16,426.90–20,813.98)	20,342.53 (18,080.74–22,921.05)	20,676.69 (18,252.00–23,205.54)	23,114.10 (20,424.74–25,978.07)
Saint Vincent and the Grenadines	Injuries	3,129.56 (2,792.76–3,592.98)	3,142.21 (2,825.84–3,499.88)	3,447.80 (3,076.45–3,819.73)	3,626.07 (3,094.38–4,124.26)
Suriname	All causes	34,822.81 (31,116.86–38,996.39)	34,077.34 (30,153.64–38,362.59)	34,482.95 (30,329.20–39,101.85)	32,226.39 (28,228.30–37,077.33)
Suriname	Group I	12,136.56 (10,078.93–14,672.57)	10,856.67 (8,730.08–13,117.26)	9,492.79 (7,401.30–11,674.53)	6,422.10 (4,728.77–8,631.60)
Suriname	NCD	18,472.30 (16,331.69–20,789.76)	19,208.44 (17,011.48–21,624.49)	20,563.45 (18,138.82–23,217.65)	21,553.40 (18,953.35–24,479.14)
Suriname	Injuries	4,213.96 (3,909.94–4,546.92)	4,012.23 (3,720.27–4,363.65)	4,426.71 (4,093.51–4,802.55)	4,250.89 (3,776.99–4,757.59)
Trinidad and Tobago	All causes	31,143.11 (28,156.49–34,217.00)	32,805.12 (29,683.86–36,108.37)	32,090.70 (28,798.58–35,554.51)	32,403.58 (28,314.50–36,603.78)
Trinidad and Tobago	Group I	6,103.66 (5,265.86–7,030.58)	6,576.34 (5,793.97–7,519.14)	4,876.76 (4,153.04–5,749.87)	3,680.01 (2,789.04–5,287.74)
Trinidad and Tobago	NCD	21,263.40 (18,984.88–23,654.31)	22,812.78 (20,363.42–25,424.83)	22,983.35 (20,271.67–25,815.66)	24,844.98 (21,676.08–28,229.70)
Trinidad and Tobago	Injuries	3,776.06 (3,493.89–4,113.27)	3,416.00 (3,132.33–3,710.77)	4,230.59 (3,737.59–4,630.85)	3,878.59 (3,376.84–4,368.18)
Virgin Islands, U.S.	All causes	27,147.47 (24,265.43–30,172.43)	29,505.07 (26,495.53–32,981.34)	31,846.21 (28,676.04–35,565.23)	35,842.12 (31,464.00–40,588.40)
Virgin Islands, U.S.	Group I	4,183.44 (3,742.60–4,774.79)	3,277.97 (2,690.82–4,339.25)	3,001.87 (2,378.18–4,121.51)	2,987.58 (2,238.19–4,375.79)
Virgin Islands, U.S.	NCD	19,611.26 (17,263.62–22,168.62)	22,974.32 (20,278.07–25,926.51)	25,324.37 (22,527.74–28,524.28)	29,563.04 (25,700.05–33,553.03)
Virgin Islands, U.S.	Injuries	3,352.78 (2,909.81–3,821.19)	3,252.78 (2,795.90–3,668.71)	3,519.98 (2,916.15–3,989.45)	3,291.51 (2,546.49–3,936.51)
Tropical Latin America	All causes	34,300.23 (31,734.14–37,172.51)	29,253.96 (26,636.24–32,292.51)	27,702.87 (24,970.42–30,860.51)	27,138.15 (24,165.49–30,332.24)
Tropical Latin America	Group I	11,485.44 (10,657.38–12,383.39)	6,505.86 (6,063.08–7,022.79)	4,964.75 (4,575.34–5,438.10)	3,715.41 (3,372.29–4,136.60)
Tropical Latin America	NCD	17,710.77 (15,510.37–19,843.44)	18,049.34 (15,767.83–20,456.54)	18,287.48 (15,913.17–20,801.50)	19,156.58 (16,726.84–21,862.64)
Tropical Latin America	Injuries	5,104.03 (4,815.66–5,443.02)	4,698.77 (4,394.50–4,995.94)	4,450.65 (4,172.96–4,739.35)	4,266.16 (3,934.53–4,594.46)
Brazil	All causes	34,515.88 (31,926.37–37,405.13)	29,376.80 (26,763.37–32,434.44)	27,785.52 (25,055.53–30,934.53)	27,210.31 (24,214.60–30,422.77)
Brazil	Group I	11,533.92 (10,694.88–12,442.50)	6,476.98 (6,023.26–7,004.50)	4,931.02 (4,545.12–5,403.59)	3,702.85 (3,365.64–4,128.22)
Brazil	NCD	17,811.79 (15,600.70–19,949.94)	18,153.80 (15,863.74–20,565.68)	18,368.08 (15,968.38–20,883.02)	19,217.59 (16,772.35–21,946.87)
Brazil	Injuries	5,170.16 (4,877.78–5,518.10)	4,746.01 (4,433.58–5,045.98)	4,486.42 (4,201.69–4,783.18)	4,289.87 (3,957.56–4,627.38)
Paraguay	All causes	26,549.11 (23,972.23–29,413.42)	25,198.82 (22,460.31–28,170.80)	25,046.92 (22,332.42–28,260.87)	24,873.38 (21,624.66–28,278.53)
Paraguay	Group I	9,742.64 (8,770.29–10,697.73)	7,459.18 (6,635.93–8,369.00)	6,048.68 (5,244.50–6,950.43)	4,109.68 (3,403.59–4,919.47)
Paraguay	NCD	14,079.48 (12,069.93–16,165.07)	14,600.61 (12,549.99–16,805.61)	15,696.99 (13,636.47–18,092.44)	17,241.74 (14,827.41–19,863.15)
Paraguay	Injuries	2,726.98 (2,525.86–2,966.90)	3,139.03 (2,887.74–3,394.47)	3,301.25 (3,062.63–3,569.84)	3,521.96 (3,118.42–3,989.26)
Southeast Asia, East Asia, and Oceania	All causes	36,595.64 (34,206.23–39,288.75)	31,128.53 (28,759.32–33,794.64)	28,412.49 (25,948.06–31,204.91)	26,429.17 (23,857.82–29,319.33)
Southeast Asia, East Asia, and Oceania	Group I	11,781.25 (11,211.88–12,487.89)	6,903.92 (6,511.69–7,377.29)	4,872.93 (4,581.43–5,233.97)	3,132.11 (2,873.43–3,432.89)
Southeast Asia, East Asia, and Oceania	NCD	19,921.93 (17,938.75–21,991.22)	20,155.07 (18,133.81–22,362.64)	19,983.77 (17,880.47–22,323.72)	20,449.77 (18,270.65–22,950.92)
Southeast Asia, East Asia, and Oceania	Injuries	4,892.46 (4,649.36–5,139.62)	4,069.55 (3,871.16–4,300.71)	3,555.78 (3,333.42–3,798.77)	2,847.29 (2,621.27–3,125.55)
East Asia	All causes	34,691.18 (32,285.84–37,324.79)	29,526.11 (27,170.97–32,113.37)	26,838.53 (24,376.31–29,631.71)	25,590.25 (22,980.44–28,576.83)
East Asia	Group I	8,539.99 (8,022.24–9,092.85)	4,312.61 (3,931.18–4,757.91)	2,555.89 (2,342.99–2,814.01)	1,643.79 (1,446.16–1,884.83)
East Asia	NCD	20,952.81 (18,974.81–23,040.20)	21,018.39 (18,982.90–23,246.05)	20,645.12 (18,499.98–23,061.81)	21,095.04 (18,823.56–23,695.15)
East Asia	Injuries	5,198.38 (4,948.85–5,459.69)	4,195.11 (3,997.49–4,442.59)	3,637.51 (3,393.04–3,880.49)	2,851.42 (2,601.33–3,144.69)
China	All causes	35,027.19 (32,619.75–37,686.88)	29,183.82 (26,850.48–31,788.67)	26,756.54 (24,241.50–29,526.31)	25,431.47 (22,837.55–28,371.56)
China	Group I	8,675.49 (8,147.27–9,236.48)	3,900.98 (3,658.94–4,223.32)	2,486.74 (2,279.40–2,738.73)	1,577.81 (1,381.32–1,812.97)
China	NCD	21,090.07 (19,107.65–23,169.40)	21,064.11 (19,020.47–23,305.27)	20,627.92 (18,465.26–23,042.50)	21,019.92 (18,743.71–23,607.94)
China	Injuries	5,261.63 (5,010.43–5,527.23)	4,218.73 (4,019.52–4,464.28)	3,641.88 (3,403.30–3,888.18)	2,833.74 (2,580.14–3,125.76)
North Korea	All causes	27,621.95 (23,965.62–31,974.61)	53,062.66 (36,393.17–71,491.81)	34,186.55 (30,359.21–38,710.53)	34,414.05 (30,690.43–38,532.18)
North Korea	Group I	7,219.41 (5,354.48–9,604.91)	28,235.53 (12,124.17–45,859.80)	7,132.50 (5,453.29–9,315.79)	5,291.28 (4,060.69–7,137.03)
North Korea	NCD	16,969.89 (14,886.10–19,340.25)	20,728.80 (18,482.86–23,255.50)	22,740.04 (20,395.64–25,358.06)	24,964.79 (22,359.30–27,716.57)
North Korea	Injuries	3,432.65 (3,034.12–3,905.57)	4,098.33 (3,575.07–4,677.64)	4,314.01 (3,775.43–4,931.06)	4,157.97 (3,615.77–4,793.96)
Taiwan, Province of China	All causes	23,253.43 (20,715.72–26,135.69)	23,270.68 (20,642.63–26,165.27)	23,374.45 (20,839.95–26,300.79)	24,898.56 (21,581.70–28,471.89)

Appendix Table 4. GBD 2016 age-standardized rate of DALYs (per 100,000) for all causes and level 1 cause groups by location for 1990, 2000, 2006 & 2016, both sexes combined.

	Cause	1990	2000	2006	2016
Taiwan, Province of China	Group I	2,323.26 (1,848.82–2,943.06)	1,624.78 (1,382.94–1,932.35)	1,437.44 (1,207.85–1,690.76)	1,377.61 (1,057.95–1,652.75)
Taiwan, Province of China	NCD	17,428.60 (15,430.28–19,670.54)	18,704.42 (16,403.99–21,266.35)	19,303.17 (17,025.56–21,919.78)	21,107.99 (18,242.17–24,218.28)
Taiwan, Province of China	Injuries	3,501.56 (3,200.28–3,763.83)	2,941.47 (2,689.85–3,210.47)	2,633.84 (2,412.99–2,872.04)	2,412.96 (2,094.78–2,771.08)
Southeast Asia	All causes	41,234.19 (38,690.93–44,067.08)	34,721.23 (32,197.25–37,647.05)	31,700.94 (29,145.99–34,468.37)	27,938.44 (25,292.17–30,890.04)
Southeast Asia	Group I	19,914.71 (18,753.06–21,239.02)	12,960.43 (12,179.91–13,870.07)	9,980.27 (9,429.37–10,637.69)	6,172.29 (5,717.37–6,656.97)
Southeast Asia	NCD	17,218.22 (15,217.83–19,340.63)	18,006.12 (16,021.29–20,188.21)	18,374.91 (16,336.96–20,562.04)	18,959.56 (16,777.07–21,275.77)
Southeast Asia	Injuries	4,101.26 (3,776.19–4,392.13)	3,754.68 (3,507.77–4,005.66)	3,345.76 (3,121.20–3,598.86)	2,806.59 (2,574.89–3,064.25)
Cambodia	All causes	66,949.53 (61,953.72–71,958.23)	53,350.15 (49,700.39–57,540.12)	42,789.63 (39,668.13–46,378.37)	32,317.53 (29,197.26–35,796.98)
Cambodia	Group I	43,153.04 (39,024.43–47,399.42)	30,381.56 (27,798.88–32,998.49)	20,597.49 (18,757.86–22,579.53)	10,853.03 (9,441.62–12,427.95)
Cambodia	NCD	17,984.70 (15,551.86–20,358.98)	17,633.35 (15,621.96–19,790.09)	17,571.19 (15,526.49–19,681.61)	17,659.73 (15,561.83–19,924.58)
Cambodia	Injuries	5,811.79 (4,879.95–6,679.63)	5,335.23 (4,779.24–5,949.31)	4,620.95 (4,188.41–5,097.40)	3,804.77 (3,358.35–4,242.47)
Indonesia	All causes	44,820.25 (42,230.19–47,646.37)	35,791.63 (33,297.89–38,566.98)	32,525.99 (30,013.74–35,398.71)	28,200.20 (25,524.44–31,104.15)
Indonesia	Group I	24,383.00 (23,016.83–25,889.44)	15,033.98 (14,158.69–15,963.99)	11,522.71 (10,927.58–12,255.68)	7,034.64 (6,525.12–7,595.61)
Indonesia	NCD	17,103.08 (15,017.40–19,246.52)	17,860.42 (15,894.37–19,949.24)	18,341.08 (16,317.50–20,471.71)	18,914.07 (16,808.38–21,292.08)
Indonesia	Injuries	3,334.16 (2,935.88–3,696.83)	2,897.22 (2,646.71–3,170.03)	2,662.20 (2,449.45–2,906.10)	2,251.49 (2,043.00–2,502.92)
Laos	All causes	96,718.20 (82,541.42–111,774.22)	72,304.62 (63,010.63–81,971.74)	57,529.25 (49,194.80–67,135.39)	40,550.59 (31,263.47–53,841.77)
Laos	Group I	67,206.06 (53,833.81–81,907.10)	44,792.57 (36,088.07–54,520.74)	31,744.42 (24,509.39–40,087.65)	17,632.69 (10,843.50–28,251.11)
Laos	NCD	23,440.41 (18,978.23–28,712.30)	22,075.19 (18,200.83–26,611.40)	20,842.44 (17,471.52–24,654.37)	19,010.26 (16,120.51–22,329.60)
Laos	Injuries	6,071.73 (4,521.88–7,445.35)	5,436.86 (4,284.94–6,516.01)	4,942.39 (4,022.10–5,853.01)	3,907.64 (3,189.00–4,866.86)
Malaysia	All causes	23,661.15 (21,414.06–26,184.03)	21,544.01 (19,288.32–23,979.81)	21,319.08 (18,963.74–24,095.32)	21,908.05 (19,316.17–24,741.48)
Malaysia	Group I	5,987.89 (5,414.06–6,704.90)	4,257.59 (3,801.34–4,783.61)	3,670.22 (3,140.47–4,222.50)	3,196.96 (2,606.63–3,756.68)
Malaysia	NCD	14,689.92 (12,871.95–16,620.10)	14,566.15 (12,774.53–16,543.10)	15,089.02 (13,107.55–17,262.63)	16,253.34 (14,117.18–18,551.52)
Malaysia	Injuries	2,983.34 (2,771.48–3,211.06)	2,720.27 (2,534.31–2,929.93)	2,559.84 (2,380.12–2,778.28)	2,457.75 (2,207.02–2,732.35)
Maldives	All causes	45,937.78 (41,843.88–50,328.57)	23,278.93 (20,820.87–26,135.95)	18,917.22 (16,423.90–21,719.51)	16,753.81 (14,010.40–19,957.68)
Maldives	Group I	18,213.86 (14,769.52–22,805.69)	5,928.24 (5,155.02–6,871.04)	3,952.62 (3,423.16–4,564.96)	2,367.56 (1,933.57–2,921.27)
Maldives	NCD	22,723.39 (18,767.25–26,483.53)	15,140.37 (13,245.59–17,261.88)	13,380.55 (11,388.64–15,581.40)	13,107.32 (10,842.40–15,635.18)
Maldives	Injuries	5,000.53 (3,747.37–5,822.69)	2,210.32 (1,998.78–2,456.99)	1,584.05 (1,419.13–1,779.64)	1,278.93 (1,057.76–1,536.01)
Mauritius	All causes	29,273.70 (26,636.62–32,279.49)	28,453.49 (25,735.01–31,475.67)	28,747.37 (25,943.43–31,988.13)	29,937.01 (26,008.01–34,186.42)
Mauritius	Group I	5,946.72 (5,405.30–6,642.70)	4,043.03 (3,559.33–4,638.14)	3,410.23 (2,952.48–4,007.93)	2,984.59 (2,534.80–3,549.75)
Mauritius	NCD	20,612.56 (18,544.49–22,912.57)	21,863.60 (19,606.86–24,227.24)	22,914.58 (20,603.48–25,503.66)	24,722.91 (21,487.17–28,172.63)
Mauritius	Injuries	2,714.43 (2,509.89–2,922.48)	2,546.86 (2,360.93–2,769.03)	2,422.57 (2,214.07–2,657.65)	2,229.51 (1,907.36–2,608.57)
Myanmar	All causes	57,777.28 (51,049.14–66,022.39)	49,917.51 (43,393.48–57,106.54)	42,719.78 (37,308.30–49,349.84)	31,215.00 (27,534.59–35,412.13)
Myanmar	Group I	32,994.02 (27,322.75–39,962.01)	23,975.43 (19,417.30–29,594.50)	17,723.53 (14,449.22–21,954.06)	7,967.02 (6,485.95–10,190.62)
Myanmar	NCD	20,702.62 (18,331.34–23,042.49)	21,674.33 (19,142.32–24,641.68)	21,135.02 (18,539.02–24,146.80)	20,191.04 (17,757.65–22,876.45)
Myanmar	Injuries	4,080.64 (3,576.92–4,650.06)	4,267.75 (3,780.07–4,900.88)	3,861.23 (3,396.90–4,411.86)	3,056.94 (2,689.52–3,551.23)
Philippines	All causes	38,265.19 (35,456.52–41,299.25)	32,422.97 (29,862.11–35,187.02)	31,706.31 (29,116.78–34,535.40)	29,348.11 (26,133.85–32,764.41)
Philippines	Group I	19,198.17 (17,896.99–20,564.43)	12,314.04 (11,475.83–13,245.97)	10,398.77 (9,625.40–11,242.65)	7,582.49 (6,775.88–8,408.66)
Philippines	NCD	15,413.98 (13,585.33–17,399.09)	16,754.18 (14,854.41–18,880.60)	17,962.99 (15,995.08–20,056.84)	18,882.20 (16,431.76–21,335.77)
Philippines	Injuries	3,653.04 (3,307.29–4,045.88)	3,354.75 (3,011.53–3,652.70)	3,344.54 (3,002.93–3,663.82)	2,883.42 (2,493.66–3,335.68)
Sri Lanka	All causes	30,124.12 (27,326.85–33,082.76)	28,540.63 (25,576.45–31,592.68)	26,581.73 (23,852.63–29,585.07)	24,133.98 (20,418.06–28,273.96)
Sri Lanka	Group I	8,078.73 (7,410.25–8,782.25)	4,655.45 (4,233.19–5,145.99)	3,781.36 (3,411.31–4,207.11)	2,368.82 (1,970.79–2,807.62)
Sri Lanka	NCD	15,689.86 (13,569.55–17,875.02)	17,968.99 (15,687.18–20,328.03)	18,112.41 (15,864.21–20,485.29)	18,475.05 (15,525.96–21,625.62)
Sri Lanka	Injuries	6,355.54 (5,726.44–7,051.11)	5,916.20 (5,228.84–6,587.27)	4,687.96 (4,156.45–5,237.88)	3,290.12 (2,663.97–4,058.16)
Seychelles	All causes	30,252.71 (27,874.98–32,950.82)	29,031.15 (26,396.45–31,804.23)	27,886.86 (25,180.10–30,916.03)	28,077.51 (24,868.32–31,730.97)
Seychelles	Group I	6,540.97 (5,928.75–7,138.78)	4,411.62 (3,953.94–5,045.05)	4,303.64 (3,912.08–4,816.51)	4,029.75 (3,590.29–4,548.37)
Seychelles	NCD	19,959.91 (18,002.63–22,136.32)	20,855.47 (18,747.04–23,162.77)	20,187.16 (17,971.06–22,697.74)	21,108.71 (18,579.91–24,032.44)
Seychelles	Injuries	3,751.83 (3,472.30–4,020.82)	3,764.06 (3,406.65–4,108.69)	3,396.06 (3,038.83–3,784.65)	2,939.05 (2,517.68–3,399.40)
Thailand	All causes	30,212.77 (27,314.82–33,284.49)	31,047.14 (28,214.50–34,172.14)	27,659.33 (24,915.60–30,692.52)	27,172.26 (23,846.96–30,708.31)
Thailand	Group I	7,587.55 (6,513.58–8,777.57)	6,434.60 (5,665.14–7,163.41)	4,595.16 (4,039.72–5,247.70)	3,400.27 (2,717.77–4,089.50)
Thailand	NCD	16,798.28 (14,696.81–19,143.29)	18,704.84 (16,410.13–21,241.19)	18,589.38 (16,152.01–21,197.09)	20,123.22 (17,377.11–23,057.43)
Thailand	Injuries	5,826.95 (5,353.67–6,237.02)	5,907.69 (5,379.68–6,453.99)	4,474.79 (4,116.05–4,849.76)	3,648.76 (3,196.24–4,135.51)
Timor-Leste	All causes	73,346.50 (62,029.13–85,563.47)	46,250.05 (39,958.74–53,379.32)	36,570.58 (30,974.67–42,485.70)	27,814.97 (22,696.62–34,338.55)
Timor-Leste	Group I	50,612.39 (40,778.46–61,865.16)	27,021.12 (21,466.01–33,806.68)	18,886.56 (14,496.78–23,649.21)	10,969.31 (7,539.16–15,964.27)
Timor-Leste	NCD	16,429.47 (13,035.02–19,884.97)	14,912.32 (12,097.45–17,729.47)	14,271.60 (11,863.03–16,715.27)	14,342.31 (11,813.17–16,884.17)
Timor-Leste	Injuries	6,304.64 (4,882.36–7,748.93)	4,316.61 (3,382.87–5,214.24)	3,412.42 (2,745.03–4,133.26)	2,503.35 (1,846.01–3,150.09)
Vietnam	All causes	34,517.60 (30,919.12–38,020.74)	28,234.74 (25,183.91–31,394.09)	27,169.61 (23,881.67–30,262.41)	25,614.64 (22,594.55–29,119.09)
Vietnam	Group I	11,994.22 (10,354.14–13,926.77)	6,585.71 (5,866.45–7,422.91)	5,272.85 (4,622.77–5,944.19)	3,411.82 (2,917.81–3,945.97)
Vietnam	NCD	17,999.78 (15,599.10–20,380.31)	17,744.25 (15,579.69–20,036.69)	18,218.56 (15,854.62–20,544.23)	18,916.85 (16,584.12–21,577.96)
Vietnam	Injuries	4,523.60 (3,909.61–5,076.49)	3,904.78 (3,338.54–4,457.46)	3,678.20 (3,117.07–4,215.68)	3,285.96 (2,746.83–3,818.66)
Oceania	All causes	51,644.23 (46,638.56–57,302.39)	48,441.00 (42,256.54–54,671.07)	48,100.59 (41,397.15–56,016.09)	42,610.37 (36,704.02–49,991.16)
Oceania	Group I	24,472.30 (21,141.03–28,334.90)	21,493.11 (17,462.23–26,390.87)	19,366.28 (15,012.54–25,335.69)	13,265.22 (10,139.38–18,106.27)
Oceania	NCD	22,349.86 (19,706.32–25,248.20)	22,512.19 (19,768.32–25,631.80)	24,067.96 (21,243.75–27,163.97)	24,813.10 (21,749.09–28,197.96)
Oceania	Injuries	4,822.07 (4,201.84–5,505.61)	4,435.70 (3,837.22–5,128.13)	4,666.34 (4,011.83–5,432.61)	4,532.05 (3,896.70–5,249.34)
American Samoa	All causes	26,464.43 (23,709.78–29,587.14)	24,720.44 (21,919.25–27,954.82)	24,359.34 (21,592.70–27,439.88)	23,573.57 (20,226.27–27,344.57)
American Samoa	Group I	6,414.10 (5,673.20–7,200.20)	4,934.80 (4,324.19–5,634.90)	4,046.45 (3,486.14–4,695.37)	3,124.50 (2,546.69–3,948.39)
American Samoa	NCD	16,810.60 (14,767.14–19,031.94)	16,942.85 (14,733.02–19,449.77)	17,314.74 (15,099.35–19,738.61)	17,985.42 (15,313.24–20,874.46)
American Samoa	Injuries	3,239.73 (2,931.17–3,591.79)	2,842.78 (2,537.11–3,176.14)	2,998.15 (2,703.16–3,316.98)	2,463.66 (2,085.42–2,879.63)
Federated States of Micronesia	All causes	40,985.56 (35,629.32–47,489.44)	35,464.77 (31,069.41–40,486.25)	32,935.06 (28,499.14–38,290.49)	34,647.62 (29,457.88–40,877.79)
Federated States of Micronesia	Group I	16,086.60 (12,835.20–20,170.36)	10,379.95 (8,634.17–12,838.81)	6,428.64 (5,114.08–8,084.27)	5,948.51 (4,495.97–8,363.81)
Federated States of Micronesia	NCD	20,651.88 (17,923.14–23,706.19)	21,100.15 (18,138.73–24,290.98)	22,511.69 (19,336.69–26,245.03)	24,526.84 (20,070.50–29,157.95)
Federated States of Micronesia	Injuries	4,247.09 (3,692.21–4,955.60)	3,984.67 (3,357.39–4,650.25)	3,994.73 (3,332.92–4,736.43)	4,172.27 (3,347.59–5,129.61)

Appendix Table 4. GBD 2016 age-standardized rate of DALYs (per 100,000) for all causes and level 1 cause groups by location for 1990, 2000, 2006 & 2016, both sexes combined.					
	Cause	1990	2000	2006	2016
Fiji	All causes	33,707.20 (28,534.24–39,382.94)	37,079.89 (33,098.04–41,511.17)	38,744.02 (34,540.15–43,226.36)	40,165.84 (33,476.27–47,167.08)
Fiji	Group I	8,961.78 (7,180.34–11,273.35)	7,407.82 (6,435.66–8,533.14)	7,038.63 (6,023.57–8,126.21)	5,628.33 (4,442.52–7,177.54)
Fiji	NCD	21,364.66 (17,916.30–25,143.42)	25,829.27 (22,818.82–29,374.61)	28,032.25 (24,752.14–31,512.02)	30,992.26 (25,525.17–36,752.14)
Fiji	Injuries	3,380.77 (2,770.15–4,142.28)	3,842.81 (3,398.70–4,308.92)	3,673.13 (3,210.54–4,176.13)	3,545.25 (2,797.28–4,360.54)
Guam	All causes	23,417.79 (20,816.25–26,292.96)	23,841.47 (20,896.99–26,925.18)	26,490.83 (23,572.34–29,818.52)	31,403.02 (27,402.01–35,559.57)
Guam	Group I	3,956.22 (3,509.47–4,472.92)	3,264.65 (2,826.20–3,778.51)	3,365.02 (2,953.77–3,953.64)	3,581.49 (2,934.35–4,415.08)
Guam	NCD	16,267.01 (14,236.70–18,483.45)	17,829.45 (15,473.95–20,276.87)	20,239.58 (17,821.31–22,988.25)	24,609.46 (21,444.28–28,027.90)
Guam	Injuries	3,194.55 (2,782.66–3,547.45)	2,747.37 (2,379.40–3,064.71)	2,886.23 (2,532.16–3,255.72)	3,212.07 (2,713.57–3,747.79)
Kiribati	All causes	62,842.62 (54,095.13–72,856.36)	53,195.24 (47,584.03–59,228.05)	50,166.31 (43,583.45–57,342.36)	45,824.13 (39,251.69–54,999.03)
Kiribati	Group I	32,277.40 (25,340.00–40,097.15)	21,467.50 (17,934.07–25,620.85)	18,735.14 (14,513.82–23,781.61)	13,314.78 (9,529.51–19,646.47)
Kiribati	NCD	26,362.17 (23,080.90–29,430.52)	27,718.24 (25,084.55–30,590.85)	27,436.18 (24,355.05–30,787.24)	28,572.65 (25,073.77–32,593.88)
Kiribati	Injuries	4,203.05 (3,519.37–4,837.34)	4,009.50 (3,512.18–4,490.96)	3,994.99 (3,411.95–4,558.88)	3,936.70 (3,271.57–4,700.49)
Marshall Islands	All causes	38,287.80 (33,834.23–43,925.54)	40,881.82 (35,307.03–47,692.53)	40,030.44 (35,308.10–45,535.31)	36,351.10 (31,370.44–42,509.48)
Marshall Islands	Group I	14,237.75 (11,183.63–18,220.26)	13,636.02 (9,989.18–18,565.60)	10,351.27 (7,600.30–13,528.11)	7,103.13 (5,072.96–10,118.15)
Marshall Islands	NCD	19,812.92 (17,565.87–22,339.02)	22,900.27 (20,222.36–25,708.32)	25,216.21 (22,484.16–28,176.93)	25,344.20 (22,208.21–29,193.74)
Marshall Islands	Injuries	4,237.14 (3,761.64–4,736.92)	4,345.53 (3,758.77–4,983.02)	4,462.96 (3,870.48–5,052.10)	3,903.77 (3,243.63–4,602.22)
Northern Mariana Islands	All causes	19,644.30 (16,379.09–23,356.67)	16,631.50 (14,104.34–19,696.99)	16,437.68 (13,991.30–19,414.35)	17,803.90 (14,990.86–21,029.05)
Northern Mariana Islands	Group I	3,591.66 (2,659.91–4,895.70)	2,544.98 (2,071.90–3,143.27)	2,136.65 (1,707.08–2,803.62)	2,198.00 (1,653.06–3,180.06)
Northern Mariana Islands	NCD	13,006.73 (10,752.84–15,457.04)	11,765.74 (9,741.42–14,057.60)	11,912.29 (10,035.72–14,262.33)	13,038.74 (10,852.17–15,528.46)
Northern Mariana Islands	Injuries	3,045.91 (2,356.37–3,627.44)	2,320.78 (1,904.47–2,760.39)	2,388.75 (1,978.54–2,825.64)	2,567.17 (2,015.23–3,100.18)
Papua New Guinea	All causes	63,761.03 (57,000.05–71,913.16)	57,777.75 (49,079.66–66,941.99)	56,018.60 (46,625.71–67,702.61)	47,313.51 (39,348.82–56,811.02)
Papua New Guinea	Group I	32,575.22 (27,608.10–38,505.54)	28,380.10 (22,221.12–35,923.80)	24,870.44 (18,504.62–33,732.05)	16,402.13 (11,980.74–23,218.33)
Papua New Guinea	NCD	25,206.85 (21,690.09–29,050.30)	24,210.42 (20,844.61–27,893.14)	25,690.70 (22,130.62–29,594.56)	25,743.63 (22,088.02–29,790.68)
Papua New Guinea	Injuries	5,978.97 (5,062.48–7,012.89)	5,187.22 (4,318.81–6,197.03)	5,457.47 (4,528.76–6,531.38)	5,167.75 (4,283.54–6,153.54)
Samoa	All causes	28,584.34 (24,030.48–35,050.24)	26,491.76 (22,736.89–31,065.05)	25,972.40 (22,462.14–30,150.67)	25,610.35 (22,250.24–29,660.65)
Samoa	Group I	8,393.74 (5,694.33–12,748.62)	5,993.14 (4,206.56–8,623.12)	5,319.84 (3,709.61–7,720.36)	4,221.46 (3,087.51–5,973.57)
Samoa	NCD	17,033.46 (14,825.12–19,555.00)	17,592.44 (15,249.66–20,099.97)	17,914.41 (15,523.30–20,454.46)	18,799.41 (16,133.20–21,401.07)
Samoa	Injuries	3,157.14 (2,680.95–3,784.33)	2,906.19 (2,431.56–3,489.00)	2,738.14 (2,272.67–3,268.21)	2,589.48 (2,106.10–3,133.36)
Solomon Islands	All causes	45,521.54 (39,207.86–53,035.61)	42,013.63 (36,430.82–48,207.68)	42,969.50 (36,828.46–49,257.26)	38,211.83 (33,046.69–44,415.96)
Solomon Islands	Group I	20,871.67 (16,455.49–26,308.23)	16,179.73 (12,745.46–20,100.43)	15,228.51 (12,069.55–19,168.68)	9,205.78 (7,176.05–11,820.15)
Solomon Islands	NCD	20,939.51 (17,797.87–24,005.06)	21,673.35 (18,517.65–25,148.87)	23,475.23 (20,146.37–27,291.14)	24,741.48 (21,441.04–28,777.79)
Solomon Islands	Injuries	3,710.35 (2,968.05–4,535.79)	4,160.55 (3,439.26–5,009.88)	4,265.76 (3,516.80–5,070.75)	4,264.57 (3,525.10–5,106.51)
Tonga	All causes	32,195.13 (27,356.29–37,720.32)	31,682.53 (27,278.77–37,148.34)	30,943.51 (26,512.91–35,911.95)	29,507.68 (25,382.03–34,266.04)
Tonga	Group I	10,459.48 (7,943.04–13,776.49)	8,431.59 (6,218.00–11,405.79)	7,795.33 (5,827.69–10,701.99)	6,206.82 (4,654.38–8,326.61)
Tonga	NCD	18,396.65 (15,813.10–21,097.17)	20,049.47 (17,648.05–22,781.83)	20,087.81 (17,636.68–22,931.92)	20,381.33 (17,550.56–23,519.24)
Tonga	Injuries	3,339.00 (2,802.69–3,953.15)	3,201.47 (2,713.55–3,782.23)	3,060.37 (2,575.17–3,613.10)	2,919.54 (2,427.14–3,455.20)
Vanuatu	All causes	44,809.47 (39,339.13–50,677.90)	42,604.03 (37,537.76–48,041.50)	43,034.10 (37,969.79–49,139.17)	40,406.33 (34,644.33–46,861.57)
Vanuatu	Group I	19,967.31 (16,512.47–24,016.14)	15,238.58 (12,715.09–18,394.80)	14,890.55 (12,232.68–18,254.83)	10,736.83 (8,469.19–13,858.98)
Vanuatu	NCD	21,413.55 (18,471.49–24,402.44)	23,176.16 (20,007.93–26,500.34)	23,992.46 (20,605.42–27,628.04)	25,614.74 (21,892.13–30,065.96)
Vanuatu	Injuries	3,428.61 (2,884.93–4,084.22)	4,189.29 (3,608.75–4,856.25)	4,151.10 (3,494.52–4,944.32)	4,054.76 (3,421.65–4,851.87)
North Africa and Middle East	All causes	44,856.16 (41,781.86–48,200.88)	34,662.35 (31,743.90–37,748.34)	31,836.75 (28,954.41–35,266.65)	28,745.98 (25,474.96–32,286.73)
North Africa and Middle East	Group I	18,941.65 (17,157.85–20,891.27)	11,541.11 (10,458.80–12,741.66)	8,700.75 (7,861.95–9,735.82)	5,655.43 (4,876.20–6,520.65)
North Africa and Middle East	NCD	20,112.53 (17,450.76–22,787.00)	18,910.47 (16,514.09–21,513.25)	18,710.85 (16,290.32–21,360.52)	18,385.26 (15,825.15–20,987.64)
North Africa and Middle East	Injuries	5,801.98 (5,059.60–6,562.01)	4,210.77 (3,906.58–4,595.87)	4,425.15 (3,936.18–4,975.03)	4,705.29 (4,082.46–5,400.82)
North Africa and Middle East	All causes	44,835.11 (41,761.66–48,178.32)	34,646.88 (31,729.02–37,732.89)	31,818.71 (28,937.38–35,248.22)	28,729.33 (25,458.36–32,269.44)
North Africa and Middle East	Group I	18,931.09 (17,148.30–20,879.55)	11,534.76 (10,453.03–12,734.40)	8,694.46 (7,856.35–9,728.59)	5,651.70 (4,872.75–6,516.67)
North Africa and Middle East	NCD	20,105.45 (17,444.43–22,780.01)	18,904.06 (16,507.98–21,506.76)	18,702.80 (16,282.64–21,352.30)	18,376.43 (15,815.92–20,978.64)
North Africa and Middle East	Injuries	5,798.57 (5,056.62–6,558.06)	4,208.06 (3,903.91–4,593.12)	4,421.46 (3,932.74–4,971.06)	4,701.20 (4,078.89–5,396.29)
Afghanistan	All causes	95,835.84 (81,042.75–110,413.17)	86,443.93 (75,147.53–97,149.89)	72,158.90 (63,882.89–81,042.36)	55,565.66 (47,293.92–64,657.74)
Afghanistan	Group I	59,524.42 (45,422.06–74,506.05)	49,134.59 (38,490.17–60,090.96)	34,735.22 (27,670.87–43,084.93)	19,726.14 (14,722.90–25,964.35)
Afghanistan	NCD	25,601.62 (19,816.02–31,909.05)	26,235.15 (21,258.94–31,410.85)	26,378.71 (21,664.56–30,960.92)	23,810.16 (20,529.41–27,072.48)
Afghanistan	Injuries	10,709.80 (8,300.12–13,804.12)	11,074.18 (9,112.53–13,362.98)	11,044.97 (9,628.78–12,735.83)	12,029.36 (8,567.50–15,730.97)
Algeria	All causes	33,492.40 (29,269.48–37,561.96)	27,716.03 (24,264.60–31,420.96)	25,479.14 (22,249.55–28,805.97)	23,314.97 (20,045.64–27,245.72)
Algeria	Group I	11,625.52 (9,384.64–14,221.51)	7,586.44 (6,054.77–9,350.35)	5,926.99 (4,795.29–7,205.02)	3,628.71 (2,836.16–4,677.83)
Algeria	NCD	17,865.96 (15,090.63–20,620.31)	16,636.33 (14,129.04–19,202.54)	16,627.86 (14,147.28–19,227.57)	17,165.18 (14,512.64–19,971.46)
Algeria	Injuries	4,000.93 (3,535.57–4,518.09)	3,493.27 (3,112.07–3,928.41)	2,924.29 (2,631.38–3,272.55)	2,521.08 (2,207.60–2,885.51)
Bahrain	All causes	25,642.63 (22,424.56–28,886.44)	23,408.35 (20,282.30–26,853.96)	20,664.64 (17,534.76–23,875.22)	18,846.55 (15,342.69–22,691.59)
Bahrain	Group I	4,680.18 (4,091.78–5,357.69)	2,683.01 (2,294.92–3,162.47)	2,185.48 (1,846.77–2,583.67)	1,707.01 (1,381.78–2,128.72)
Bahrain	NCD	17,989.90 (15,330.02–20,690.16)	16,921.11 (14,500.13–19,717.42)	15,637.02 (13,074.33–18,413.30)	15,141.49 (12,320.66–18,343.82)
Bahrain	Injuries	2,972.55 (2,612.10–3,362.91)	3,804.23 (2,930.53–4,721.47)	2,842.14 (2,430.17–3,331.26)	1,998.05 (1,616.23–2,490.29)
Egypt	All causes	48,366.34 (44,443.37–52,396.32)	33,149.58 (29,742.30–36,577.21)	31,650.52 (28,392.32–35,183.37)	27,762.94 (24,035.22–31,540.19)
Egypt	Group I	22,002.65 (19,820.87–24,220.61)	9,932.84 (8,587.13–11,437.47)	7,893.55 (6,706.27–9,228.01)	4,644.71 (3,555.00–6,107.82)
Egypt	NCD	21,991.27 (19,598.63–24,471.79)	20,125.00 (17,643.43–22,635.70)	20,794.25 (18,241.75–23,340.47)	20,442.18 (17,586.82–23,311.50)
Egypt	Injuries	4,372.42 (3,898.32–4,841.91)	3,091.74 (2,836.44–3,377.60)	2,962.72 (2,698.83–3,251.37)	2,676.05 (2,302.83–3,056.74)
Iran	All causes	43,494.06 (37,831.09–50,493.51)	28,354.48 (24,496.10–32,886.60)	27,649.07 (23,070.14–33,273.59)	24,535.26 (20,437.08–29,187.87)
Iran	Group I	13,907.53 (11,163.55–17,190.75)	5,311.17 (3,869.20–7,247.54)	4,317.16 (2,676.53–6,873.15)	2,908.58 (1,813.95–4,832.78)
Iran	NCD	18,704.65 (16,048.32–21,877.46)	17,667.03 (14,936.88–20,545.93)	18,233.69 (15,387.93–21,335.48)	18,165.45 (15,148.79–21,532.38)
Iran	Injuries	10,881.88 (7,387.47–14,372.22)	5,376.27 (4,675.92–6,136.59)	5,098.21 (4,291.27–6,033.75)	3,461.23 (2,900.53–4,164.53)
Iraq	All causes	42,382.95 (32,484.41–50,647.05)	38,857.64 (32,121.07–46,024.74)	44,867.60 (37,149.83–53,124.15)	35,896.61 (28,665.60–42,919.28)
Iraq	Group I	15,855.28 (8,451.33–21,617.67)	12,798.44 (8,165.33–17,619.82)	11,037.59 (8,248.58–14,151.34)	8,997.46 (5,179.72–12,593.41)
Iraq	NCD	20,764.78 (17,480.84–23,952.30)	20,641.18 (18,079.42–23,569.94)	20,665.45 (17,793.55–23,463.54)	18,457.87 (15,458.28–21,528.01)

Appendix Table 4. GBD 2016 age-standardized rate of DALYs (per 100,000) for all causes and level 1 cause groups by location for 1990, 2000, 2006 & 2016, both sexes combined.

	Cause	1990	2000	2006	2016
Iraq	Injuries	5,762.88 (4,686.19–6,969.44)	5,418.02 (4,571.63–6,361.58)	13,164.57 (6,768.54–19,496.64)	8,441.27 (5,103.91–12,188.37)
Jordan	All causes	28,601.35 (25,534.70–31,869.30)	25,085.92 (21,975.00–28,476.91)	23,407.81 (20,293.30–26,655.79)	21,922.03 (18,732.29–25,526.36)
Jordan	Group I	8,112.30 (7,127.99–9,203.18)	5,686.53 (5,004.80–6,447.19)	4,755.73 (4,190.12–5,413.18)	3,427.91 (2,843.30–4,074.36)
Jordan	NCD	17,023.52 (14,772.85–19,484.22)	16,437.30 (14,039.18–19,012.75)	16,132.44 (13,619.77–18,755.24)	15,249.22 (12,741.82–18,039.57)
Jordan	Injuries	3,465.53 (3,105.20–3,845.66)	2,962.09 (2,561.49–3,389.29)	2,519.64 (2,199.43–2,904.45)	3,244.90 (2,380.73–4,088.61)
Kuwait	All causes	19,545.94 (16,445.00–23,345.90)	18,251.16 (15,612.01–21,330.77)	18,245.65 (15,424.06–21,313.63)	16,579.19 (13,305.43–20,115.80)
Kuwait	Group I	2,555.12 (2,173.86–3,007.46)	2,287.60 (1,972.07–2,659.13)	2,127.95 (1,841.17–2,498.77)	1,580.78 (1,266.11–1,975.56)
Kuwait	NCD	12,391.59 (10,239.29–14,754.12)	13,620.92 (11,467.03–16,150.93)	13,655.40 (11,348.19–16,178.99)	13,156.71 (10,416.53–16,011.44)
Kuwait	Injuries	4,599.23 (2,695.87–6,632.67)	2,342.64 (2,030.64–2,698.37)	2,462.31 (2,122.76–2,842.13)	1,841.69 (1,441.85–2,312.44)
Lebanon	All causes	36,247.56 (32,482.26–40,300.60)	24,102.84 (20,935.01–27,611.74)	22,587.77 (19,418.56–26,091.52)	21,300.75 (17,938.69–24,960.21)
Lebanon	Group I	5,959.77 (4,873.89–7,197.94)	3,023.04 (2,444.10–3,688.06)	2,150.84 (1,699.66–2,741.72)	1,572.32 (1,242.05–1,977.53)
Lebanon	NCD	22,204.73 (19,365.80–25,205.39)	18,192.91 (15,681.09–20,977.63)	17,107.48 (14,524.57–19,875.76)	17,192.64 (14,420.48–20,187.21)
Lebanon	Injuries	8,083.07 (6,662.60–9,620.60)	2,886.89 (2,224.53–3,766.25)	3,329.45 (2,563.40–4,205.23)	2,535.79 (1,842.28–3,335.79)
Libya	All causes	27,020.44 (23,774.31–30,743.76)	24,375.24 (21,182.46–27,730.49)	22,781.16 (19,534.63–26,305.97)	23,945.53 (20,456.89–27,797.30)
Libya	Group I	6,464.60 (5,155.50–7,951.37)	4,593.71 (3,740.56–5,540.34)	3,562.73 (2,818.44–4,365.05)	2,194.78 (1,723.75–2,735.55)
Libya	NCD	17,196.50 (14,756.35–19,814.17)	16,520.22 (14,101.47–19,043.45)	16,305.77 (13,805.53–19,006.96)	17,382.70 (14,757.67–20,323.85)
Libya	Injuries	3,359.34 (2,990.48–3,770.60)	3,261.31 (2,878.03–3,653.53)	2,912.66 (2,555.30–3,300.55)	4,368.05 (2,952.90–5,864.40)
Morocco	All causes	42,168.71 (38,827.71–45,657.84)	31,714.25 (28,523.45–35,160.59)	29,128.29 (25,761.71–32,735.93)	26,544.62 (23,183.98–30,465.25)
Morocco	Group I	20,803.44 (18,995.06–22,993.54)	10,462.72 (9,069.23–12,236.59)	7,541.85 (6,413.01–8,942.66)	4,340.19 (3,585.13–5,292.20)
Morocco	NCD	17,415.17 (14,830.02–20,037.82)	18,010.19 (15,340.09–20,654.72)	18,587.21 (15,792.84–21,363.51)	19,492.30 (16,596.54–22,535.49)
Morocco	Injuries	3,950.10 (3,474.49–4,416.04)	3,241.33 (2,893.26–3,582.79)	2,999.23 (2,668.90–3,340.59)	2,712.13 (2,415.57–3,031.53)
Palestine	All causes	31,222.20 (22,813.56–38,473.71)	25,570.64 (20,209.00–30,913.42)	25,070.35 (20,747.65–29,520.10)	23,910.21 (19,948.93–28,501.86)
Palestine	Group I	10,155.74 (5,309.35–14,329.95)	6,789.51 (3,884.70–9,667.45)	6,491.42 (4,231.33–8,758.85)	5,717.09 (3,621.95–8,166.10)
Palestine	NCD	17,366.00 (13,748.49–20,829.65)	15,365.04 (12,823.27–17,858.55)	15,741.87 (13,406.06–18,118.44)	15,929.39 (13,683.77–18,457.72)
Palestine	Injuries	3,700.47 (2,759.19–4,622.26)	3,416.08 (2,386.68–4,421.26)	2,837.06 (2,291.45–3,411.93)	2,263.73 (1,890.55–2,682.15)
Oman	All causes	35,176.18 (30,777.99–40,091.05)	21,427.19 (18,579.90–24,429.36)	18,068.65 (15,558.08–20,912.89)	19,010.16 (16,135.30–22,123.87)
Oman	Group I	10,967.54 (8,499.45–13,865.41)	4,541.77 (3,783.93–5,420.92)	2,586.49 (2,187.47–3,063.73)	2,007.59 (1,678.35–2,442.12)
Oman	NCD	17,991.18 (15,219.72–21,162.46)	13,074.53 (11,044.21–15,225.44)	12,282.75 (10,249.34–14,515.39)	13,541.46 (11,158.54–16,145.31)
Oman	Injuries	6,217.46 (5,318.77–7,098.99)	3,810.89 (3,442.51–4,233.91)	3,199.41 (2,885.53–3,577.37)	3,461.12 (3,026.64–3,940.24)
Qatar	All causes	21,784.67 (18,459.57–25,503.82)	21,492.05 (18,404.77–25,186.24)	19,051.05 (15,900.84–22,234.01)	16,553.74 (13,241.01–20,088.83)
Qatar	Group I	3,323.17 (2,644.79–4,218.35)	2,268.43 (1,812.31–2,843.76)	1,651.45 (1,317.31–2,051.29)	1,213.65 (962.84–1,520.26)
Qatar	NCD	14,825.70 (12,338.22–17,632.19)	15,159.41 (12,695.87–17,931.82)	13,278.01 (10,841.27–15,921.65)	12,107.74 (9,558.48–14,969.97)
Qatar	Injuries	3,635.81 (3,046.53–4,407.93)	4,064.21 (3,396.75–4,946.14)	4,121.59 (3,383.35–4,886.02)	3,232.34 (2,502.13–4,139.02)
Saudi Arabia	All causes	33,197.04 (29,630.30–37,162.66)	24,071.12 (21,214.46–27,275.36)	20,945.37 (18,335.39–23,932.59)	18,570.44 (15,825.96–21,851.59)
Saudi Arabia	Group I	10,924.52 (9,153.38–13,367.58)	5,848.47 (4,957.20–6,923.13)	3,827.68 (3,215.68–4,533.98)	2,046.51 (1,685.30–2,529.95)
Saudi Arabia	NCD	16,824.49 (14,088.78–19,552.67)	14,476.24 (12,360.98–16,748.42)	13,588.64 (11,488.83–15,920.49)	13,651.29 (11,261.30–16,223.58)
Saudi Arabia	Injuries	5,448.02 (4,842.81–6,088.48)	3,746.41 (3,471.98–4,063.20)	3,529.05 (3,261.68–3,817.36)	2,872.64 (2,548.26–3,221.38)
Sudan	All causes	66,690.28 (60,606.67–73,111.42)	53,905.47 (48,418.27–60,104.09)	44,819.60 (39,416.12–50,548.86)	36,004.25 (29,789.20–44,545.84)
Sudan	Group I	35,773.93 (30,188.15–42,119.37)	26,628.98 (22,144.24–31,960.69)	19,054.85 (15,248.86–23,326.89)	11,940.66 (8,412.31–17,493.22)
Sudan	NCD	23,888.33 (19,036.47–28,278.39)	21,423.77 (17,951.98–24,853.56)	20,282.75 (17,192.31–23,494.01)	19,207.93 (16,106.27–22,823.30)
Sudan	Injuries	7,028.02 (5,728.63–8,283.07)	5,852.71 (5,046.07–6,790.97)	5,482.00 (4,767.69–6,200.76)	4,855.67 (4,121.15–5,582.85)
Syria	All causes	31,895.76 (28,264.21–35,783.24)	22,807.27 (20,116.69–25,625.27)	20,991.75 (18,283.12–23,874.91)	37,880.94 (24,022.81–52,125.59)
Syria	Group I	10,539.73 (8,630.58–12,681.27)	4,558.47 (3,902.71–5,261.25)	3,751.11 (3,172.88–4,396.04)	2,437.74 (1,965.23–3,044.96)
Syria	NCD	18,985.02 (16,544.73–21,488.93)	16,333.03 (14,217.80–18,531.24)	15,373.88 (13,250.73–17,721.80)	15,346.62 (13,084.57–17,755.73)
Syria	Injuries	2,371.00 (2,090.20–2,685.67)	1,915.77 (1,727.60–2,155.03)	1,866.76 (1,648.89–2,111.41)	20,096.58 (6,978.57–33,970.37)
Tunisia	All causes	31,706.46 (28,783.19–34,972.69)	24,967.18 (21,994.78–28,423.78)	24,402.40 (21,175.12–28,034.26)	24,053.75 (20,390.26–28,134.84)
Tunisia	Group I	8,178.97 (6,913.98–9,830.22)	3,796.68 (3,268.36–4,399.43)	3,093.48 (2,663.18–3,585.73)	2,140.89 (1,786.17–2,563.44)
Tunisia	NCD	19,833.23 (17,056.14–22,715.23)	18,264.85 (15,621.25–21,026.88)	18,558.00 (15,781.31–21,604.26)	19,422.89 (16,311.88–22,715.31)
Tunisia	Injuries	3,694.26 (3,267.70–4,181.67)	2,905.65 (2,520.32–3,315.93)	2,750.92 (2,328.99–3,204.84)	2,489.97 (2,075.29–2,982.19)
Turkey	All causes	38,082.68 (33,948.67–42,358.13)	28,994.52 (25,660.59–32,162.34)	24,259.71 (21,220.19–27,634.12)	22,470.89 (19,070.74–26,136.90)
Turkey	Group I	13,698.24 (11,179.26–16,432.42)	6,557.54 (5,546.31–7,693.49)	3,794.10 (3,201.42–4,466.16)	2,259.80 (1,797.36–2,861.85)
Turkey	NCD	20,820.21 (17,960.01–23,766.52)	19,643.65 (16,955.07–22,326.87)	18,335.31 (15,609.07–21,198.05)	18,221.36 (15,324.96–21,235.97)
Turkey	Injuries	3,564.23 (3,143.56–4,062.38)	2,793.32 (2,498.68–3,141.63)	2,130.31 (1,899.24–2,416.44)	1,989.73 (1,676.59–2,351.03)
United Arab Emirates	All causes	24,482.36 (19,993.36–29,459.73)	20,119.21 (16,912.16–23,565.75)	18,521.06 (15,690.86–21,623.08)	21,264.55 (17,665.43–25,451.03)
United Arab Emirates	Group I	4,543.25 (2,981.77–7,013.12)	2,409.10 (1,833.91–3,269.57)	1,733.70 (1,392.92–2,163.09)	1,625.19 (1,254.34–2,086.99)
United Arab Emirates	NCD	15,658.06 (12,534.46–18,923.67)	13,968.95 (11,539.33–16,564.35)	13,149.35 (10,978.92–15,653.49)	15,676.42 (12,940.16–18,862.47)
United Arab Emirates	Injuries	4,281.05 (3,370.23–5,288.40)	3,741.16 (3,072.50–4,477.20)	3,638.01 (3,106.81–4,203.71)	3,962.95 (2,998.57–4,979.88)
Yemen	All causes	79,887.02 (64,458.06–91,243.76)	57,258.72 (43,572.62–68,357.04)	45,297.91 (35,224.90–54,893.61)	40,307.98 (32,094.72–48,905.55)
Yemen	Group I	49,262.99 (35,808.19–60,103.84)	30,943.07 (19,744.55–40,435.14)	21,547.39 (14,191.80–29,426.49)	13,621.76 (7,908.37–19,848.30)
Yemen	NCD	23,464.37 (18,220.41–30,186.44)	20,773.77 (17,062.20–25,343.82)	18,744.24 (15,466.10–22,054.12)	17,282.91 (14,587.02–20,323.30)
Yemen	Injuries	7,159.67 (5,875.46–8,470.92)	5,541.88 (4,786.86–6,330.17)	5,006.28 (4,396.05–5,664.86)	9,403.31 (8,313.21–10,970.48)
South Asia	All causes	63,203.00 (60,172.15–66,543.43)	49,713.84 (46,942.25–52,903.30)	43,712.46 (40,884.64–46,856.76)	34,770.34 (31,878.52–38,074.66)
South Asia	Group I	39,166.72 (37,699.19–40,729.98)	26,155.04 (25,080.65–27,416.46)	20,581.29 (19,639.71–21,683.98)	11,552.68 (10,688.08–12,500.38)
South Asia	NCD	18,778.28 (16,701.47–21,071.15)	18,773.49 (16,619.39–21,056.37)	18,659.33 (16,534.84–20,905.11)	19,288.65 (17,226.18–21,660.47)
South Asia	Injuries	5,258.00 (4,683.11–5,738.35)	4,785.31 (4,296.26–5,179.01)	4,471.83 (4,030.64–4,871.17)	3,929.02 (3,553.23–4,314.64)
Bangladesh	All causes	68,837.58 (65,016.92–72,935.13)	43,805.32 (40,475.53–47,306.07)	36,397.95 (33,555.09–39,640.86)	27,552.27 (24,530.28–30,952.84)
Bangladesh	Group I	43,290.41 (40,845.61–45,795.58)	21,608.45 (20,108.67–23,188.20)	15,030.12 (13,934.18–16,247.96)	7,370.68 (6,560.12–8,280.50)
Bangladesh	NCD	18,932.17 (16,506.24–21,456.99)	17,320.35 (15,054.28–19,703.61)	17,368.72 (15,159.24–19,725.51)	17,275.41 (14,921.16–19,723.57)
Bangladesh	Injuries	6,615.00 (5,396.44–7,475.65)	4,876.52 (3,732.64–5,463.11)	3,999.11 (3,236.66–4,446.11)	2,906.18 (2,485.69–3,328.65)
Bhutan	All causes	71,810.79 (65,071.60–78,817.78)	50,373.06 (45,294.85–55,872.06)	37,041.19 (32,899.90–41,222.90)	27,313.14 (23,778.01–31,225.29)
Bhutan	Group I	46,988.44 (41,098.74–53,196.37)	26,935.69 (23,518.55–30,906.27)	17,651.33 (14,996.30–20,380.65)	8,484.49 (7,074.94–10,209.49)

Appendix Table 4. GBD 2016 age-standardized rate of DALYs (per 100,000) for all causes and level 1 cause groups by location for 1990, 2000, 2006 & 2016, both sexes combined.					
	Cause	1990	2000	2006	2016
Bhutan	NCD	19,982.75<br>(17,049.84–22,747.58)	17,505.27<br>(15,081.33–19,988.82)	16,195.00<br>(13,878.62–18,389.80)	16,269.99<br>(13,857.63–18,876.59)
Bhutan	Injuries	4,839.60<br>(3,866.20–5,680.03)	5,932.09<br>(4,538.36–7,290.41)	3,194.87<br>(2,805.05–3,631.76)	2,558.66<br>(2,152.02–3,044.18)
India	All causes	62,220.58<br>(59,049.56–65,458.59)	50,351.26<br>(47,493.61–53,530.15)	44,392.70<br>(41,443.68–47,538.27)	35,434.92<br>(32,539.74–38,774.97)
India	Group I	37,876.23<br>(36,287.93–39,573.35)	26,416.41<br>(25,286.48–27,839.63)	20,913.79<br>(19,870.92–22,118.95)	11,594.01<br>(10,644.54–12,666.62)
India	NCD	19,012.49<br>(16,964.01–21,317.49)	18,970.70<br>(16,829.04–21,263.67)	18,787.47<br>(16,657.32–21,102.32)	19,644.25<br>(17,530.18–22,001.34)
India	Injuries	5,331.86<br>(4,752.22–5,828.48)	4,964.15<br>(4,452.76–5,369.06)	4,691.43<br>(4,206.96–5,114.58)	4,196.66<br>(3,779.83–4,598.43)
Nepal	All causes	75,882.42<br>(65,638.72–86,349.25)	49,573.94<br>(44,685.58–55,002.91)	39,912.20<br>(36,001.31–44,147.61)	30,683.22<br>(27,084.89–34,386.62)
Nepal	Group I	53,484.88<br>(44,229.67–63,287.57)	28,819.70<br>(24,528.75–33,375.85)	19,246.06<br>(16,419.79–22,246.33)	10,541.51<br>(8,763.94–12,606.32)
Nepal	NCD	17,476.31<br>(15,250.59–19,746.94)	16,594.81<br>(14,613.17–18,880.25)	16,932.16<br>(14,798.04–19,147.01)	17,124.25<br>(14,940.93–19,455.33)
Nepal	Injuries	4,921.24<br>(4,060.88–5,898.36)	4,159.43<br>(3,662.60–4,647.06)	3,733.98<br>(3,267.14–4,166.81)	3,017.46<br>(2,600.81–3,420.38)
Pakistan	All causes	63,381.27<br>(58,596.09–68,433.18)	50,448.92<br>(47,042.87–54,454.76)	46,076.73<br>(42,412.77–50,051.04)	36,984.51<br>(32,725.46–41,338.04)
Pakistan	Group I	42,975.99<br>(39,315.29–46,917.91)	27,993.77<br>(26,008.82–30,239.11)	23,478.53<br>(21,405.83–25,812.01)	14,985.36<br>(12,533.79–17,775.52)
Pakistan	NCD	16,981.55<br>(14,840.21–19,132.40)	19,022.54<br>(16,944.90–21,164.73)	19,198.14<br>(16,961.56–21,461.71)	18,898.21<br>(16,473.99–21,623.83)
Pakistan	Injuries	3,423.72<br>(2,917.11–3,972.09)	3,432.61<br>(2,994.23–3,911.41)	3,400.06<br>(2,996.13–3,862.98)	3,100.93<br>(2,656.23–3,595.18)
Sub-Saharan Africa	All causes	93,266.12<br>(89,335.19–97,260.20)	86,876.27<br>(82,969.61–90,939.48)	75,853.26<br>(72,520.22–79,660.31)	51,052.40<br>(47,954.20–54,585.53)
Sub-Saharan Africa	Group I	70,248.40<br>(67,329.77–73,340.03)	65,491.50<br>(62,685.20–68,315.29)	56,266.59<br>(53,884.16–58,815.53)	33,175.65<br>(31,333.00–35,303.07)
Sub-Saharan Africa	NCD	17,114.83<br>(14,920.08–19,369.83)	16,273.93<br>(14,157.38–18,532.01)	15,497.90<br>(13,510.62–17,625.71)	14,425.83<br>(12,538.22–16,516.05)
Sub-Saharan Africa	Injuries	5,902.89<br>(5,124.59–6,636.07)	5,110.84<br>(4,585.49–5,639.71)	4,088.78<br>(3,704.94–4,457.80)	3,450.93<br>(3,198.81–3,736.86)
Southern Sub-Saharan Africa	All causes	47,767.56<br>(44,728.05–51,097.60)	71,564.26<br>(66,978.18–76,525.58)	84,233.98<br>(79,603.17–89,469.79)	52,175.75<br>(48,507.56–56,316.03)
Southern Sub-Saharan Africa	Group I	24,205.99<br>(22,655.78–25,983.41)	45,229.61<br>(41,504.08–49,249.09)	57,571.34<br>(53,154.77–62,105.38)	28,586.94<br>(26,263.74–30,971.65)
Southern Sub-Saharan Africa	NCD	16,236.58<br>(14,354.20–18,317.88)	18,823.98<br>(16,560.73–21,264.00)	19,343.44<br>(17,097.58–21,767.02)	17,862.14<br>(15,709.29–20,241.20)
Southern Sub-Saharan Africa	Injuries	7,324.98<br>(6,702.69–7,844.44)	7,510.67<br>(6,729.27–8,331.63)	7,319.21<br>(6,568.57–8,224.36)	5,726.67<br>(5,216.61–6,196.73)
Botswana	All causes	39,108.76<br>(33,230.54–45,303.19)	89,678.91<br>(65,468.70–116,258.09)	79,848.94<br>(64,623.11–98,286.49)	38,742.68<br>(30,884.52–45,968.22)
Botswana	Group I	21,913.93<br>(17,523.72–26,873.45)	67,743.55<br>(49,979.51–89,161.65)	58,590.93<br>(46,771.97–72,870.65)	18,692.94<br>(15,581.38–22,170.17)
Botswana	NCD	13,804.29<br>(11,381.15–16,284.73)	17,039.70<br>(11,149.90–27,101.03)	16,426.75<br>(11,847.04–23,627.68)	16,144.30<br>(11,323.92–20,701.68)
Botswana	Injuries	3,390.55<br>(2,720.72–4,093.89)	4,895.66<br>(2,794.80–8,851.49)	4,831.25<br>(3,083.43–7,255.08)	3,905.44<br>(2,229.63–5,251.30)
Lesotho	All causes	53,877.03<br>(47,452.11–60,471.32)	83,689.01<br>(73,761.90–96,533.26)	103,909.90<br>(91,062.65–119,093.25)	78,426.58<br>(68,198.98–89,818.07)
Lesotho	Group I	31,596.01<br>(26,282.87–37,025.45)	58,013.68<br>(49,289.60–69,404.68)	76,027.09<br>(64,623.81–89,910.30)	50,950.75<br>(43,708.71–60,385.95)
Lesotho	NCD	17,066.01<br>(14,723.69–19,726.75)	19,058.25<br>(15,440.44–22,305.09)	20,452.41<br>(17,155.82–23,912.97)	19,852.98<br>(16,421.67–23,495.41)
Lesotho	Injuries	5,215.00<br>(4,372.22–5,930.74)	6,617.09<br>(5,161.04–7,910.60)	7,430.39<br>(6,026.27–8,821.37)	7,622.85<br>(5,967.33–9,420.54)
Namibia	All causes	51,413.61<br>(46,932.71–56,340.07)	66,956.82<br>(57,934.75–77,751.05)	69,788.34<br>(60,931.53–80,098.16)	41,023.00<br>(35,325.97–47,503.76)
Namibia	Group I	30,223.76<br>(27,288.04–33,614.50)	45,019.20<br>(37,760.34–54,938.06)	49,396.07<br>(41,166.77–59,214.21)	22,734.97<br>(18,936.67–28,096.98)
Namibia	NCD	15,914.97<br>(13,945.93–18,067.47)	16,166.30<br>(12,467.07–19,687.74)	15,269.82<br>(12,319.25–18,388.15)	13,949.42<br>(11,097.85–16,733.03)
Namibia	Injuries	5,274.87<br>(4,724.74–5,832.52)	5,771.31<br>(4,554.75–6,929.19)	5,122.44<br>(4,246.82–6,034.45)	4,338.61<br>(3,304.83–5,148.76)
South Africa	All causes	47,254.74<br>(44,051.27–50,826.99)	66,601.00<br>(62,110.64–71,267.15)	82,492.75<br>(77,080.83–88,189.87)	52,151.72<br>(48,073.52–56,940.47)
South Africa	Group I	21,439.69<br>(19,802.89–23,349.77)	37,830.87<br>(34,209.85–42,087.82)	53,729.22<br>(48,350.52–59,207.05)	27,092.60<br>(24,203.41–30,194.55)
South Africa	NCD	17,138.55<br>(15,097.30–19,331.02)	20,093.24<br>(17,797.80–22,663.07)	20,381.90<br>(17,948.98–22,960.54)	18,699.47<br>(16,411.92–21,179.80)
South Africa	Injuries	8,676.50<br>(7,927.73–9,290.32)	8,676.88<br>(7,690.05–9,732.95)	8,381.63<br>(7,366.39–9,599.08)	6,359.65<br>(5,691.20–6,993.44)
Swaziland	All causes	47,297.52<br>(42,292.60–52,609.90)	84,976.72<br>(71,693.97–100,754.01)	103,544.07<br>(89,144.27–119,038.82)	57,771.02<br>(49,635.05–67,021.10)
Swaziland	Group I	26,464.65<br>(22,876.43–30,149.74)	62,204.37<br>(50,888.60–77,503.22)	78,343.16<br>(65,640.66–93,624.10)	35,915.23<br>(31,155.05–42,518.37)
Swaziland	NCD	15,468.00<br>(13,272.63–17,796.34)	16,653.70<br>(13,046.51–22,174.96)	18,464.93<br>(15,323.53–23,352.61)	16,342.71<br>(12,751.59–20,411.90)
Swaziland	Injuries	5,364.87<br>(4,714.62–6,090.43)	6,118.65<br>(4,638.74–8,334.40)	6,735.98<br>(5,370.00–8,889.63)	5,513.08<br>(4,146.10–7,098.73)
Zimbabwe	All causes	49,377.47<br>(44,087.37–55,538.25)	84,811.18<br>(72,426.52–98,807.20)	89,076.28<br>(78,215.29–100,773.02)	51,955.34<br>(46,678.68–57,360.55)
Zimbabwe	Group I	32,442.87<br>(28,457.02–38,326.25)	65,611.49<br>(53,896.58–78,758.18)	68,535.30<br>(58,729.87–79,587.43)	32,262.27<br>(28,818.53–36,414.35)
Zimbabwe	NCD	13,253.97<br>(10,638.12–15,617.58)	15,033.02<br>(11,954.01–18,295.21)	16,445.30<br>(13,619.37–19,144.83)	15,815.20<br>(13,397.66–18,401.55)
Zimbabwe	Injuries	3,680.62<br>(2,756.79–4,449.23)	4,166.67<br>(3,240.34–5,239.24)	4,095.69<br>(3,456.16–5,283.13)	3,877.87<br>(3,204.91–4,798.10)
Western Sub-Saharan Africa	All causes	97,606.76<br>(92,880.50–102,801.74)	88,980.55<br>(83,756.03–94,181.02)	78,812.73<br>(73,929.97–84,153.89)	55,434.24<br>(51,527.04–59,701.34)
Western Sub-Saharan Africa	Group I	75,639.34<br>(71,472.43–80,018.72)	68,467.62<br>(63,716.53–73,359.08)	59,645.20<br>(55,345.38–64,466.84)	37,820.09<br>(34,748.13–41,235.20)
Western Sub-Saharan Africa	NCD	17,644.56<br>(14,765.89–20,527.96)	16,678.46<br>(13,824.26–19,532.63)	15,804.43<br>(13,126.67–18,296.25)	14,642.04<br>(12,370.77–16,866.70)
Western Sub-Saharan Africa	Injuries	4,322.86<br>(3,540.79–5,063.91)	3,834.47<br>(3,278.58–4,365.47)	3,363.09<br>(2,983.73–3,770.30)	2,972.11<br>(2,682.45–3,311.62)
Benin	All causes	94,392.64<br>(88,042.92–101,433.58)	76,971.15<br>(71,100.18–83,289.58)	65,868.74<br>(61,087.97–71,743.01)	48,521.37<br>(43,587.63–54,433.79)
Benin	Group I	69,501.76<br>(63,547.38–75,475.67)	54,210.35<br>(49,244.23–59,525.01)	45,543.58<br>(41,568.43–50,194.25)	29,707.87<br>(25,850.31–33,944.00)
Benin	NCD	19,999.98<br>(16,973.67–23,092.33)	18,471.26<br>(15,584.02–21,290.89)	16,746.29<br>(14,362.21–19,217.44)	15,742.36<br>(13,679.30–17,967.57)
Benin	Injuries	4,890.90<br>(3,956.98–5,982.83)	4,289.55<br>(3,668.84–4,966.76)	3,578.87<br>(3,191.49–4,045.16)	3,071.14<br>(2,684.11–3,484.35)
Burkina Faso	All causes	118,215.85<br>(103,103.53–131,521.35)	102,014.83<br>(88,566.81–111,976.78)	87,798.29<br>(73,973.30–100,649.15)	62,094.84<br>(50,781.05–73,335.44)
Burkina Faso	Group I	93,674.71<br>(80,456.15–105,420.89)	79,773.92<br>(67,478.63–91,140.73)	66,660.80<br>(54,891.27–78,621.40)	42,056.73<br>(32,510.28–51,324.68)
Burkina Faso	NCD	19,297.05<br>(16,129.62–22,706.20)	17,908.47<br>(14,925.53–20,888.41)	17,287.40<br>(14,156.23–20,026.89)	16,455.99<br>(13,744.47–19,126.37)
Burkina Faso	Injuries	5,244.08<br>(4,292.64–6,388.05)	4,332.44<br>(3,691.92–5,141.06)	3,850.10<br>(3,331.75–4,429.15)	3,582.12<br>(3,091.55–4,148.31)
Cameroon	All causes	75,864.52<br>(71,176.69–81,370.32)	79,853.37<br>(73,555.43–87,545.43)	75,197.11<br>(68,761.36–82,342.34)	58,106.73<br>(50,902.11–66,372.30)
Cameroon	Group I	54,430.80<br>(50,568.65–58,680.58)	57,953.26<br>(52,693.35–64,113.26)	53,778.62<br>(48,476.91–59,405.83)	37,149.17<br>(31,654.16–43,724.72)
Cameroon	NCD	17,561.25<br>(15,203.47–20,054.46)	18,017.38<br>(15,175.38–20,797.35)	17,645.74<br>(14,896.12–20,462.76)	17,190.37<br>(14,360.63–20,139.97)
Cameroon	Injuries	3,872.46<br>(3,289.07–4,481.45)	3,882.73<br>(3,309.00–4,479.37)	3,772.75<br>(3,263.14–4,318.46)	3,767.19<br>(3,210.51–4,413.23)
Cape Verde	All causes	42,809.57<br>(39,450.78–46,459.13)	33,942.82<br>(30,248.28–37,874.76)	30,028.76<br>(26,557.43–33,860.14)	25,608.87<br>(22,587.83–29,068.95)
Cape Verde	Group I	22,494.38<br>(20,587.09–24,454.51)	14,869.17<br>(12,978.70–16,984.56)	11,242.06<br>(9,784.60–12,887.53)	7,213.91<br>(6,288.98–8,236.64)
Cape Verde	NCD	17,042.85<br>(15,044.62–19,144.37)	15,446.88<br>(13,131.02–17,809.70)	15,248.77<br>(12,988.34–17,533.30)	15,070.17<br>(12,822.42–17,402.35)
Cape Verde	Injuries	3,272.33<br>(2,820.97–3,747.94)	3,626.77<br>(3,029.69–4,277.49)	3,537.94<br>(2,945.42–4,142.39)	3,324.79<br>(2,714.54–3,879.02)
Chad	All causes	106,981.60<br>(101,257.16–112,500.45)	102,299.74<br>(96,276.27–108,534.31)	91,918.25<br>(85,649.21–98,441.54)	68,833.63<br>(62,212.39–76,215.87)
Chad	Group I	84,435.43<br>(79,486.91–89,759.10)	80,385.56<br>(74,939.05–86,119.33)	70,372.95<br>(64,763.49–76,144.28)	49,752.41<br>(44,373.85–56,448.44)
Chad	NCD	17,153.75<br>(14,398.36–20,303.17)	16,599.24<br>(13,615.42–19,542.22)	15,937.90<br>(13,135.24–18,889.24)	15,195.69<br>(12,693.01–17,736.76)
Chad	Injuries	5,392.41<br>(4,152.64–6,662.96)	5,314.95<br>(4,463.61–6,185.48)	5,607.40<br>(4,518.81–6,831.70)	3,885.53<br>(3,373.62–4,523.25)
Cote d'Ivoire	All causes	84,424.87<br>(76,370.73–91,486.73)	83,591.58<br>(76,401.60–91,394.03)	76,864.07<br>(68,026.21–85,799.90)	57,403.58<br>(50,235.17–65,720.46)

Appendix Table 4. GBD 2016 age-standardized rate of DALYs (per 100,000) for all causes and level 1 cause groups by location for 1990, 2000, 2006 & 2016, both sexes combined.

	Cause	1990	2000	2006	2016
Cote d'Ivoire	Group I	61,149.48 (54,455.30–67,164.18)	59,908.42 (54,339.73–66,615.64)	54,444.11 (46,882.81–62,473.02)	35,607.71 (29,896.26–42,006.52)
Cote d'Ivoire	NCD	18,983.69 (16,632.77–21,500.18)	19,347.68 (16,676.50–22,287.47)	18,621.06 (15,781.46–21,330.53)	18,060.73 (15,551.86–20,902.29)
Cote d'Ivoire	Injuries	4,291.70 (3,701.31–4,988.11)	4,335.48 (3,800.53–4,970.42)	3,798.90 (3,353.97–4,316.64)	3,735.13 (3,271.53–4,259.73)
The Gambia	All causes	69,301.28 (62,134.78–76,806.66)	58,058.19 (52,515.57–63,671.03)	50,114.87 (44,913.09–55,612.54)	38,468.91 (34,019.94–43,077.49)
The Gambia	Group I	50,365.24 (44,213.45–56,407.08)	40,682.73 (36,044.94–45,180.02)	33,124.15 (29,116.39–37,505.88)	22,830.45 (19,782.91–26,270.20)
The Gambia	NCD	15,682.14 (13,448.42–17,872.60)	14,486.51 (12,141.49–16,658.20)	14,276.03 (12,016.11–16,410.11)	13,365.94 (11,418.60–15,464.74)
The Gambia	Injuries	3,253.89 (2,725.41–3,842.82)	2,888.95 (2,517.31–3,286.87)	2,714.70 (2,387.65–3,081.85)	2,272.52 (1,965.59–2,614.15)
Ghana	All causes	68,712.91 (63,277.66–74,205.29)	59,302.73 (53,879.14–64,681.65)	56,383.61 (51,889.94–61,012.92)	40,267.80 (36,164.35–44,794.35)
Ghana	Group I	50,048.66 (45,831.56–54,437.81)	40,106.31 (35,699.26–44,302.65)	36,724.27 (33,330.92–40,213.58)	22,017.45 (19,214.88–25,250.87)
Ghana	NCD	15,442.08 (13,473.31–17,560.99)	16,194.15 (14,172.67–18,339.13)	16,594.75 (14,513.89–18,707.45)	15,541.56 (13,396.57–17,708.49)
Ghana	Injuries	3,222.17 (2,703.74–3,852.78)	3,002.27 (2,610.04–3,453.69)	3,064.59 (2,743.83–3,447.88)	2,708.79 (2,369.92–3,091.21)
Guinea	All causes	109,489.14 (103,905.80–115,153.91)	89,348.86 (84,119.99–94,787.21)	78,798.73 (73,251.39–84,931.94)	58,722.97 (52,606.28–65,743.74)
Guinea	Group I	83,547.96 (77,798.29–89,180.64)	65,732.44 (61,139.62–71,053.78)	56,409.37 (51,924.85–62,003.85)	38,006.53 (33,378.07–44,050.36)
Guinea	NCD	21,019.68 (17,415.47–24,628.90)	19,320.19 (16,000.76–22,235.24)	18,634.83 (15,702.81–21,392.45)	17,527.31 (14,927.48–20,027.71)
Guinea	Injuries	4,921.50 (3,774.05–6,082.67)	4,296.24 (3,582.59–5,031.69)	3,754.54 (3,252.99–4,285.90)	3,189.14 (2,758.47–3,623.17)
Guinea-Bissau	All causes	111,421.65 (104,452.90–118,644.44)	89,953.35 (84,432.79–96,145.65)	79,895.70 (74,330.36–85,947.47)	57,589.81 (52,779.31–62,833.22)
Guinea-Bissau	Group I	81,364.14 (75,277.10–87,815.46)	62,177.54 (57,481.46–67,174.29)	53,672.14 (49,343.70–58,307.70)	34,094.32 (30,851.69–37,501.32)
Guinea-Bissau	NCD	24,101.26 (20,783.22–27,908.39)	22,504.26 (19,274.05–25,416.19)	21,145.11 (18,308.43–23,923.39)	19,449.88 (17,050.09–21,923.87)
Guinea-Bissau	Injuries	5,956.25 (4,850.39–7,045.72)	5,271.54 (4,602.35–5,992.63)	5,078.45 (4,389.27–5,777.76)	4,045.60 (3,513.72–4,618.92)
Liberia	All causes	123,492.47 (115,464.57–131,562.40)	94,328.77 (88,432.52–100,955.66)	69,955.26 (65,363.51–75,093.66)	45,890.76 (41,596.66–50,602.04)
Liberia	Group I	87,866.50 (80,464.64–95,955.56)	73,062.25 (67,767.04–78,962.56)	51,536.45 (47,707.08–55,753.93)	28,515.28 (25,384.74–32,074.35)
Liberia	NCD	19,906.10 (16,261.84–23,315.97)	17,586.28 (14,369.70–20,486.53)	15,593.34 (13,199.18–17,960.15)	14,876.55 (12,857.47–16,990.84)
Liberia	Injuries	15,719.86 (13,743.90–17,787.67)	3,680.24 (3,107.84–4,301.80)	2,825.47 (2,451.09–3,237.53)	2,498.94 (2,162.17–2,848.29)
Mali	All causes	128,038.39 (122,308.34–133,803.02)	106,883.98 (100,967.70–113,613.29)	89,232.01 (83,316.79–95,682.39)	64,739.10 (57,031.00–73,650.47)
Mali	Group I	101,852.69 (95,747.00–108,337.77)	83,715.89 (77,852.66–89,691.82)	69,168.13 (63,493.20–75,417.87)	47,110.32 (40,457.69–55,361.18)
Mali	NCD	20,747.03 (16,893.13–24,763.74)	17,793.38 (14,704.12–20,980.70)	16,355.95 (13,291.14–19,157.79)	14,642.32 (11,952.70–17,144.80)
Mali	Injuries	5,438.67 (4,027.39–6,625.24)	5,374.70 (4,122.37–6,716.66)	3,707.93 (3,008.95–4,382.78)	2,986.46 (2,505.59–3,539.87)
Mauritania	All causes	61,226.68 (57,072.93–65,478.03)	50,157.84 (46,719.23–54,081.91)	43,573.99 (39,859.40–47,760.86)	32,253.85 (28,673.31–36,144.49)
Mauritania	Group I	41,636.35 (38,879.60–44,391.33)	32,376.53 (29,885.69–35,250.66)	26,278.47 (23,694.04–29,173.82)	15,965.28 (14,041.56–18,364.66)
Mauritania	NCD	15,859.29 (13,614.45–18,129.03)	15,052.50 (12,541.24–17,520.14)	14,746.70 (12,085.95–17,038.48)	14,164.61 (11,628.90–16,519.68)
Mauritania	Injuries	3,731.04 (2,864.19–4,616.73)	2,728.81 (2,226.97–3,124.10)	2,548.82 (2,096.20–2,976.84)	2,123.96 (1,699.79–2,598.25)
Niger	All causes	153,693.92 (145,424.24–162,141.43)	119,988.91 (112,578.13–127,739.01)	93,422.69 (86,193.61–100,733.50)	63,708.77 (56,222.66–72,628.51)
Niger	Group I	128,347.57 (119,643.53–137,352.36)	99,024.75 (91,536.19–106,944.43)	73,527.86 (66,983.99–80,713.55)	46,066.81 (39,509.23–53,679.71)
Niger	NCD	19,139.40 (14,927.72–23,353.07)	16,420.49 (13,123.96–19,828.66)	15,896.73 (13,019.97–18,863.05)	14,539.18 (12,052.67–17,028.16)
Niger	Injuries	6,206.94 (4,190.91–7,910.06)	4,543.67 (3,419.91–5,626.57)	3,998.11 (3,261.07–4,819.81)	3,102.78 (2,652.50–3,642.11)
Nigeria	All causes	98,219.70 (89,923.51–106,782.35)	93,004.38 (83,613.84–102,353.16)	83,051.26 (74,346.87–92,343.44)	56,262.05 (49,607.93–63,675.57)
Nigeria	Group I	77,667.04 (70,492.54–85,725.06)	73,895.16 (65,180.68–82,376.71)	65,737.16 (58,010.20–74,324.62)	40,585.72 (34,700.73–46,692.84)
Nigeria	NCD	16,715.84 (13,539.80–20,073.92)	15,630.39 (12,204.32–19,123.06)	14,369.74 (11,325.78–17,386.41)	13,008.18 (10,339.30–15,578.33)
Nigeria	Injuries	3,836.81 (2,989.10–4,708.21)	3,478.83 (2,790.12–4,194.74)	2,944.36 (2,422.00–3,479.02)	2,668.15 (2,265.87–3,149.63)
Sao Tome and Principe	All causes	59,866.67 (55,995.83–64,160.81)	48,728.16 (44,891.91–52,733.50)	39,758.94 (36,423.11–43,570.98)	28,840.77 (25,656.51–32,541.17)
Sao Tome and Principe	Group I	36,461.74 (33,666.25–39,417.64)	27,720.21 (24,768.49–31,446.25)	20,513.23 (18,068.48–23,940.87)	11,622.78 (10,040.23–13,805.10)
Sao Tome and Principe	NCD	20,021.13 (17,696.99–22,352.08)	18,294.89 (15,596.29–20,650.38)	16,744.81 (14,031.57–19,092.18)	15,008.96 (12,944.52–17,374.86)
Sao Tome and Principe	Injuries	3,383.80 (2,910.61–3,878.61)	2,713.06 (2,193.67–3,192.43)	2,500.90 (1,853.55–3,007.94)	2,209.03 (1,779.54–2,674.16)
Senegal	All causes	77,158.44 (72,791.28–81,703.54)	63,249.04 (59,761.12–67,263.92)	54,060.51 (50,404.51–58,047.67)	38,581.47 (35,321.80–42,476.68)
Senegal	Group I	56,035.50 (52,378.47–59,649.99)	43,478.67 (40,751.26–46,471.12)	33,863.59 (31,178.89–36,661.71)	20,570.58 (18,750.39–22,947.31)
Senegal	NCD	17,126.99 (14,936.80–19,469.60)	16,353.88 (14,135.88–18,560.53)	16,621.34 (14,356.22–18,696.47)	15,167.22 (13,181.71–17,227.92)
Senegal	Injuries	3,995.95 (3,432.37–4,595.37)	3,416.49 (3,025.20–3,848.71)	3,575.58 (3,197.44–3,983.55)	2,843.67 (2,503.82–3,241.70)
Sierra Leone	All causes	115,944.49 (109,949.75–121,898.33)	105,246.25 (99,823.09–110,706.79)	92,503.76 (87,260.40–97,983.75)	64,656.59 (59,373.65–70,507.74)
Sierra Leone	Group I	88,347.98 (82,036.88–95,581.33)	79,381.18 (73,757.31–85,242.71)	69,104.76 (63,810.83–75,359.28)	43,212.33 (38,593.63–48,760.46)
Sierra Leone	NCD	22,552.58 (18,072.98–26,778.33)	19,961.91 (15,808.94–23,685.39)	19,601.29 (15,592.52–22,863.89)	18,136.25 (14,976.24–21,091.88)
Sierra Leone	Injuries	5,043.93 (3,732.56–6,165.58)	5,903.15 (4,677.08–7,215.38)	3,797.70 (3,158.94–4,476.26)	3,308.01 (2,837.07–3,866.16)
Togo	All causes	78,219.05 (71,446.14–85,316.21)	72,049.73 (64,268.73–80,037.96)	67,197.01 (58,535.64–76,587.00)	47,507.99 (42,261.49–53,429.26)
Togo	Group I	55,957.27 (50,166.89–62,028.96)	51,355.96 (44,826.50–58,149.37)	47,124.08 (39,493.35–55,417.94)	28,524.82 (24,559.79–32,774.75)
Togo	NCD	18,227.60 (15,792.63–20,751.02)	17,030.19 (14,481.71–19,497.17)	16,653.82 (14,117.31–19,062.85)	15,968.81 (13,851.69–18,207.84)
Togo	Injuries	4,034.18 (3,464.01–4,656.23)	3,663.58 (3,168.71–4,205.22)	3,419.12 (2,973.73–3,934.77)	3,014.36 (2,665.89–3,421.08)
Eastern Sub-Saharan Africa	All causes	100,580.16 (97,052.51–104,181.79)	87,448.24 (84,063.19–90,970.88)	70,078.61 (66,980.80–73,356.33)	45,402.13 (42,493.57–48,478.11)
Eastern Sub-Saharan Africa	Group I	76,697.35 (74,220.35–79,010.21)	66,579.67 (64,511.14–68,512.04)	52,027.90 (50,316.11–53,792.32)	28,621.23 (27,258.35–30,031.52)
Eastern Sub-Saharan Africa	NCD	16,802.44 (14,917.99–18,844.56)	15,201.21 (13,397.76–17,170.06)	14,217.84 (12,507.61–16,130.33)	13,486.18 (11,739.39–15,455.98)
Eastern Sub-Saharan Africa	Injuries	7,080.36 (6,262.22–7,953.92)	5,667.36 (5,178.87–6,188.27)	3,832.87 (3,487.61–4,206.95)	3,294.72 (2,988.28–3,608.97)
Burundi	All causes	111,390.87 (102,529.92–121,213.59)	99,587.59 (91,821.80–107,598.48)	76,114.30 (70,029.38–82,866.68)	56,631.24 (47,158.68–69,114.80)
Burundi	Group I	85,326.11 (78,097.08–93,530.62)	76,280.96 (69,647.33–83,290.90)	56,887.48 (51,923.52–62,588.84)	38,636.25 (30,316.81–50,687.88)
Burundi	NCD	20,574.25 (18,052.29–23,262.54)	16,809.09 (14,737.71–19,042.35)	15,034.89 (13,007.93–17,160.91)	14,146.80 (12,086.54–16,506.99)
Burundi	Injuries	5,490.51 (4,566.11–6,503.27)	6,497.54 (5,388.89–7,691.38)	4,191.92 (3,659.16–4,790.64)	3,848.18 (3,260.28–4,529.48)
Comoros	All causes	66,571.84 (61,482.24–71,995.99)	54,289.65 (49,774.08–59,104.49)	45,372.07 (41,005.01–50,235.68)	33,856.70 (29,710.86–38,457.23)
Comoros	Group I	45,028.16 (40,925.34–48,799.22)	36,158.76 (32,717.98–40,031.47)	27,966.54 (24,775.38–31,781.55)	17,396.15 (14,634.40–20,673.59)
Comoros	NCD	17,172.73 (14,874.54–19,532.23)	14,835.38 (12,915.41–16,965.51)	13,825.66 (11,920.39–16,001.74)	13,666.28 (11,651.77–15,960.02)
Comoros	Injuries	4,370.95 (3,650.47–5,155.81)	3,295.52 (2,882.46–3,725.43)	3,579.87 (3,131.94–4,053.47)	2,794.27 (2,392.12–3,277.71)
Djibouti	All causes	69,318.64 (61,536.68–76,866.64)	63,731.50 (56,239.53–71,630.01)	57,465.32 (49,687.11–65,747.34)	41,717.56 (34,734.19–50,146.27)
Djibouti	Group I	48,350.10 (41,899.05–54,789.36)	45,270.16 (39,323.78–51,704.29)	38,666.54 (32,389.48–45,454.12)	23,885.37 (18,712.22–30,756.04)
Djibouti	NCD	15,685.20 (13,575.53–18,049.75)	14,449.12 (12,289.56–16,711.59)	14,323.15 (12,025.64–16,789.58)	14,740.11 (12,216.85–17,273.66)
Djibouti	Injuries	5,283.34 (4,334.87–6,390.32)	4,012.22 (3,289.33–4,841.88)	4,475.64 (3,634.41–5,323.73)	3,092.07 (2,568.30–3,751.55)

Appendix Table 4. GBD 2016 age-standardized rate of DALYs (per 100,000) for all causes and level 1 cause groups by location for 1990, 2000, 2006 & 2016, both sexes combined.

	Cause	1990	2000	2006	2016
Eritrea	All causes	87,506.25 (80,406.17–94,940.24)	58,933.46 (53,586.23–64,866.68)	51,482.25 (45,789.43–57,704.28)	39,718.83 (34,882.10–45,177.54)
Eritrea	Group I	66,960.83 (60,676.18–73,518.84)	37,803.39 (33,523.69–42,308.99)	33,651.00 (29,097.47–38,778.18)	22,568.70 (19,238.60–26,491.18)
Eritrea	NCD	16,042.00 (13,867.87–18,200.44)	13,780.07 (11,816.12–15,816.82)	13,817.44 (11,805.57–15,928.66)	13,657.93 (11,592.50–15,771.90)
Eritrea	Injuries	4,503.41 (3,768.18–5,319.53)	7,350.00 (6,740.23–7,961.41)	4,013.80 (3,513.44–4,578.45)	3,492.20 (3,006.02–3,987.61)
Ethiopia	All causes	114,240.37 (108,844.53–119,778.36)	87,389.66 (82,720.74–92,421.50)	63,552.11 (59,050.58–68,328.87)	37,291.71 (33,170.01–41,344.26)
Ethiopia	Group I	82,028.58 (77,261.11–87,147.33)	61,332.35 (57,472.92–65,448.98)	44,452.65 (41,317.79–47,878.37)	20,087.23 (17,941.04–22,571.92)
Ethiopia	NCD	19,607.39 (17,312.36–22,183.62)	16,616.44 (14,586.38–18,810.49)	15,062.06 (13,136.81–17,213.89)	14,021.13 (11,898.95–16,485.89)
Ethiopia	Injuries	12,604.40 (11,263.67–14,045.48)	9,440.87 (8,648.13–10,251.56)	4,037.40 (3,545.76–4,585.66)	3,183.35 (2,684.17–3,720.68)
Kenya	All causes	57,936.85 (55,454.01–60,721.57)	66,274.96 (63,232.98–69,416.25)	55,756.29 (53,164.12–58,614.01)	35,730.41 (33,344.82–38,626.52)
Kenya	Group I	44,790.22 (43,145.75–46,293.09)	52,949.10 (51,108.06–54,805.09)	42,567.95 (41,086.92–44,077.26)	22,617.42 (21,413.08–23,914.87)
Kenya	NCD	10,329.07 (8,552.03–12,205.45)	10,610.69 (8,883.97–12,605.90)	10,548.11 (8,788.22–12,525.85)	10,694.63 (8,873.80–12,714.97)
Kenya	Injuries	2,817.57 (2,351.14–3,284.03)	2,715.16 (2,323.29–3,113.91)	2,640.24 (2,281.84–3,024.12)	2,418.36 (2,069.91–2,796.05)
Madagascar	All causes	87,058.80 (80,900.86–93,564.37)	70,402.36 (64,433.31–76,889.01)	60,401.37 (54,706.10–66,134.00)	50,124.83 (43,468.68–57,442.72)
Madagascar	Group I	63,719.55 (58,591.43–69,059.19)	49,436.17 (44,832.55–54,489.10)	40,945.87 (36,366.73–45,350.27)	30,664.85 (25,738.54–36,877.74)
Madagascar	NCD	19,357.94 (17,254.79–21,479.81)	17,656.58 (15,595.99–19,803.57)	16,619.98 (14,410.13–18,841.85)	16,659.14 (14,067.31–19,134.42)
Madagascar	Injuries	3,981.31 (3,263.06–4,700.64)	3,309.61 (2,860.22–3,794.87)	2,835.52 (2,430.03–3,243.77)	2,800.85 (2,325.27–3,273.66)
Malawi	All causes	127,350.75 (119,178.10–137,274.83)	116,216.97 (105,636.99–127,766.56)	92,837.00 (83,635.14–101,777.93)	55,653.52 (49,121.08–63,301.71)
Malawi	Group I	104,407.57 (96,955.74–113,392.68)	94,973.95 (86,148.22–104,982.53)	73,902.52 (66,646.14–81,547.07)	38,476.74 (33,368.59–44,741.44)
Malawi	NCD	17,372.62 (14,487.02–20,208.68)	16,696.81 (13,195.78–19,899.81)	15,419.33 (12,346.12–18,279.61)	14,262.13 (11,908.41–16,602.94)
Malawi	Injuries	5,570.56 (4,117.92–7,048.47)	4,546.21 (3,620.90–5,498.93)	3,515.15 (2,844.05–4,126.12)	2,914.65 (2,434.73–3,441.75)
Mozambique	All causes	110,371.40 (98,336.59–121,700.56)	98,252.41 (89,940.18–107,151.37)	86,724.11 (78,807.77–95,401.83)	58,483.04 (52,047.07–65,168.08)
Mozambique	Group I	86,661.34 (75,949.59–97,164.67)	77,711.45 (70,719.37–85,649.07)	68,464.00 (62,016.21–75,880.89)	41,499.61 (36,756.22–46,877.45)
Mozambique	NCD	17,338.95 (15,104.97–19,724.72)	15,553.31 (12,954.66–18,276.66)	14,125.05 (11,743.82–16,883.15)	13,393.12 (11,298.28–15,542.67)
Mozambique	Injuries	6,371.11 (5,147.60–7,707.92)	4,987.65 (4,142.90–5,909.70)	4,135.06 (3,420.62–4,943.71)	3,590.32 (3,034.31–4,266.90)
Rwanda	All causes	105,529.91 (98,791.92–113,470.73)	95,727.84 (87,884.14–103,725.34)	62,506.24 (57,705.37–68,205.41)	35,728.46 (32,019.06–40,507.06)
Rwanda	Group I	81,263.32 (75,679.86–87,780.99)	74,459.67 (67,648.14–81,604.13)	44,971.63 (41,076.06–49,261.36)	20,037.59 (17,243.99–23,564.83)
Rwanda	NCD	16,576.79 (14,373.44–18,936.86)	15,687.33 (13,566.24–17,920.30)	13,116.37 (11,301.19–15,170.95)	12,380.46 (10,496.39–14,521.48)
Rwanda	Injuries	7,689.81 (5,908.54–9,483.84)	5,580.83 (4,620.42–6,675.46)	4,418.24 (3,810.89–5,155.24)	3,310.41 (2,819.24–3,919.89)
Somalia	All causes	95,479.50 (81,271.82–110,182.51)	82,240.25 (70,910.08–94,052.60)	71,072.38 (62,487.21–79,854.79)	57,730.02 (50,209.16–66,439.66)
Somalia	Group I	67,723.82 (55,140.89–81,124.29)	59,416.57 (49,926.59–70,243.89)	49,051.25 (41,680.73–56,807.06)	36,044.77 (29,684.44–43,063.66)
Somalia	NCD	19,573.51 (16,172.33–22,645.70)	17,627.08 (14,768.41–20,371.31)	16,493.05 (13,819.75–19,203.77)	15,815.03 (13,615.54–18,415.26)
Somalia	Injuries	8,182.18 (6,435.14–10,205.66)	5,196.59 (4,238.53–6,325.54)	5,528.09 (4,555.47–6,549.59)	5,870.21 (4,825.95–6,904.57)
South Sudan	All causes	125,848.67 (110,700.34–141,509.81)	96,614.43 (86,932.90–106,192.34)	83,703.07 (74,502.30–93,479.08)	69,381.25 (58,924.94–82,083.46)
South Sudan	Group I	101,725.67 (88,338.83–115,763.79)	77,256.94 (69,351.19–85,238.59)	66,385.39 (58,741.04–75,101.02)	52,177.05 (43,063.12–63,428.17)
South Sudan	NCD	14,775.93 (11,941.25–17,511.69)	13,584.39 (11,037.58–16,079.41)	13,261.38 (10,810.11–15,847.43)	13,251.12 (11,017.88–15,761.95)
South Sudan	Injuries	9,347.07 (5,985.52–12,790.71)	5,773.11 (4,225.17–7,365.54)	4,056.31 (3,267.40–4,955.68)	3,953.07 (3,224.22–4,803.37)
Tanzania	All causes	91,892.44 (85,279.64–98,483.00)	83,771.55 (77,520.94–90,109.23)	69,994.67 (64,451.82–75,528.66)	43,517.22 (39,132.43–48,429.28)
Tanzania	Group I	70,744.56 (65,208.60–76,441.97)	65,195.35 (60,303.85–70,677.87)	51,935.14 (47,113.95–56,453.04)	26,796.33 (23,681.35–30,147.69)
Tanzania	NCD	16,201.00 (14,007.02–18,537.60)	14,604.05 (12,424.95–16,774.38)	14,166.61 (12,097.79–16,378.81)	13,396.05 (11,469.52–15,553.34)
Tanzania	Injuries	4,946.89 (4,204.72–5,807.06)	3,972.16 (3,402.92–4,517.64)	3,892.92 (3,357.21–4,415.12)	3,324.84 (2,887.69–3,829.01)
Uganda	All causes	113,030.30 (106,660.65–121,062.30)	99,386.31 (93,620.18–105,465.56)	79,373.53 (74,867.91–84,401.85)	49,069.12 (44,997.93–54,085.61)
Uganda	Group I	91,771.92 (86,237.15–98,801.75)	80,627.95 (75,535.87–85,721.04)	61,889.86 (58,372.02–65,953.95)	33,147.47 (30,270.56–36,567.23)
Uganda	NCD	16,248.57 (14,025.67–18,367.18)	14,357.43 (12,621.33–16,345.12)	13,549.91 (11,764.99–15,452.78)	12,458.35 (10,690.63–14,330.74)
Uganda	Injuries	5,009.81 (4,140.85–5,933.94)	4,400.93 (3,815.83–5,021.26)	3,933.77 (3,400.48–4,527.23)	3,463.30 (3,011.20–3,891.53)
Zambia	All causes	101,103.04 (94,033.08–108,431.22)	109,489.56 (99,791.81–120,328.04)	93,749.92 (84,825.29–102,479.25)	53,887.33 (47,483.81–60,865.81)
Zambia	Group I	80,803.30 (74,649.63–87,569.49)	87,465.90 (78,201.60–97,540.07)	72,448.15 (65,089.14–80,585.66)	35,437.54 (30,980.68–40,826.78)
Zambia	NCD	14,755.22 (12,654.36–16,861.79)	16,893.47 (14,461.71–19,343.62)	16,353.14 (13,871.55–18,616.51)	14,416.43 (12,146.33–16,905.90)
Zambia	Injuries	5,544.52 (4,385.86–6,856.31)	5,130.18 (4,366.92–5,958.40)	4,948.63 (4,220.34–5,698.05)	4,033.36 (3,379.89–4,757.86)
Central Sub-Saharan Africa	All causes	97,112.19 (77,599.96–112,875.59)	90,998.88 (73,809.75–106,489.58)	78,033.53 (66,889.11–88,611.52)	53,997.96 (45,102.46–63,913.60)
Central Sub-Saharan Africa	Group I	73,806.24 (56,764.28–88,127.46)	69,196.59 (54,002.48–82,908.52)	57,505.58 (47,609.55–67,044.06)	35,387.79 (27,993.32–44,300.76)
Central Sub-Saharan Africa	NCD	17,108.67 (14,043.46–19,906.92)	16,157.83 (13,531.54–18,774.51)	15,620.63 (13,221.21–17,994.93)	14,519.88 (12,380.38–16,719.35)
Central Sub-Saharan Africa	Injuries	6,197.28 (4,487.37–7,599.24)	5,644.46 (4,403.11–6,739.96)	4,907.31 (3,879.58–5,720.23)	4,090.29 (3,426.91–4,796.44)
Angola	All causes	111,975.17 (99,745.98–124,927.64)	92,689.14 (82,594.63–103,750.78)	75,252.27 (67,019.84–84,046.10)	43,628.01 (35,419.37–53,251.28)
Angola	Group I	87,205.35 (76,413.02–99,228.70)	70,445.82 (61,506.72–80,758.30)	53,313.37 (46,578.63–60,742.15)	26,113.34 (20,028.96–33,988.17)
Angola	NCD	16,268.81 (13,279.46–19,667.32)	15,531.66 (12,725.29–18,463.75)	15,657.40 (12,878.89–18,309.55)	13,407.02 (11,031.36–16,046.36)
Angola	Injuries	8,501.00 (6,097.89–10,660.05)	6,711.66 (4,963.40–8,415.45)	6,281.50 (4,752.65–7,636.46)	4,107.65 (3,223.09–5,068.07)
Central African Republic	All causes	105,555.17 (96,922.66–115,638.20)	115,961.00 (103,616.25–128,989.04)	109,407.61 (97,867.79–121,668.50)	89,403.80 (76,162.09–105,075.04)
Central African Republic	Group I	77,246.20 (69,227.74–86,735.13)	88,590.07 (77,984.93–100,036.62)	81,500.64 (72,171.81–92,579.79)	63,123.60 (52,303.67–76,843.34)
Central African Republic	NCD	21,515.63 (18,321.00–24,593.82)	20,758.32 (17,200.91–24,261.85)	20,748.16 (17,114.44–24,267.44)	19,862.96 (16,330.79–23,230.40)
Central African Republic	Injuries	6,793.35 (5,137.97–8,198.09)	6,612.62 (4,996.29–8,048.14)	7,158.82 (5,632.37–8,653.92)	6,417.24 (5,093.91–7,843.97)
Congo (Brazzaville)	All causes	73,699.25 (66,770.71–80,880.91)	78,974.81 (72,123.03–86,939.41)	63,890.10 (58,114.78–69,647.67)	46,149.75 (39,096.19–54,098.81)
Congo (Brazzaville)	Group I	48,110.49 (42,820.09–53,983.40)	53,793.95 (48,505.68–60,202.80)	42,211.66 (38,049.46–46,852.68)	26,880.51 (22,226.33–32,869.88)
Congo (Brazzaville)	NCD	19,976.42 (17,251.92–22,791.49)	18,806.21 (16,023.44–21,609.54)	17,075.43 (14,545.79–19,639.56)	15,572.14 (12,955.49–18,224.52)
Congo (Brazzaville)	Injuries	5,612.34 (4,410.89–6,714.86)	6,374.65 (4,955.62–7,800.71)	4,603.01 (3,709.19–5,435.08)	3,697.10 (2,987.42–4,473.14)
Democratic Republic of the Congo	All causes	93,987.40 (65,881.47–117,265.83)	90,298.71 (66,798.30–112,293.45)	78,357.43 (62,557.60–93,508.68)	56,039.86 (43,641.31–69,735.86)
Democratic Republic of the Congo	Group I	71,921.33 (47,082.30–92,699.36)	69,379.71 (47,607.12–90,085.13)	58,948.60 (44,381.60–72,968.11)	37,603.25 (27,147.59–49,893.61)
Democratic Republic of the Congo	NCD	16,613.34 (13,377.05–19,617.38)	15,704.19 (12,908.38–18,532.61)	15,067.75 (12,527.08–17,461.86)	14,450.19 (12,156.10–16,858.42)
Democratic Republic of the Congo	Injuries	5,452.73 (3,676.89–7,142.17)	5,214.80 (3,954.02–6,472.31)	4,341.08 (3,416.09–5,283.13)	3,986.41 (3,234.60–4,873.48)
Equatorial Guinea	All causes	118,354.73 (101,651.48–135,459.90)	78,911.42 (67,119.52–91,566.90)	60,858.41 (49,707.28–72,344.53)	42,736.75 (35,227.61–50,992.75)
Equatorial Guinea	Group I	87,645.04 (73,430.72–103,107.14)	55,518.40 (46,238.29–65,540.83)	41,535.87 (33,457.58–50,635.05)	26,503.86 (21,439.72–32,584.77)
Equatorial Guinea	NCD	22,754.23 (18,974.87–26,804.54)	17,831.46 (14,092.74–21,406.43)	15,202.97 (11,598.36–19,089.73)	13,408.64 (10,316.11–16,911.81)

Appendix Table 4. GBD 2016 age-standardized rate of DALYs (per 100,000) for all causes and level 1 cause groups by location for 1990, 2000, 2006 & 2016, both sexes combined.

	Cause	1990	2000	2006	2016
Equatorial Guinea	Injuries	7,955.47<br>(5,735.75–10,112.89)	5,561.56<br>(4,070.06–7,040.58)	4,119.58<br>(2,915.85–5,388.73)	2,824.24<br>(2,057.95–3,813.24)
Gabon	All causes	62,091.85<br>(54,178.72–69,775.93)	58,107.00<br>(52,196.71–64,059.72)	54,219.79<br>(48,391.19–60,537.05)	39,820.34<br>(34,729.36–45,758.47)
Gabon	Group I	34,639.12<br>(28,598.24–40,972.79)	33,719.03<br>(29,424.92–38,223.05)	30,770.19<br>(26,354.43–35,551.29)	19,744.03<br>(16,571.40–23,486.10)
Gabon	NCD	22,099.47<br>(19,429.96–24,763.11)	20,017.91<br>(17,399.44–22,637.99)	19,032.61<br>(16,479.84–21,699.45)	16,522.43<br>(13,923.27–19,353.37)
Gabon	Injuries	5,353.26<br>(4,481.06–6,265.72)	4,370.06<br>(3,650.98–5,119.62)	4,416.99<br>(3,693.95–5,260.20)	3,553.89<br>(2,868.98–4,333.34)

Appendix Table 5. GBD 2016 DALYs exemplars for 1990, 2006, and 2016 on the basis of SDI											
1990				2006				2016			
Location	Observed Age Standardized DALYs	Expected Age Standardized DALYs	Ratio of Observed to Expected	Location	Observed Age Standardized DALYs	Expected Age Standardized DALYs	Ratio of Observed to Expected	Location	Observed Age Standardized DALYs	Expected Age Standardized DALYs	Ratio of Observed to Expected
Oman	0.381	0.719	0.531	Nicaragua	0.257	0.405	0.635	Nicaragua	0.233	0.360	0.648
Saudi Arabia	0.358	0.546	0.657	Costa Rica	0.214	0.315	0.681	Costa Rica	0.200	0.289	0.692
Costa Rica	0.250	0.373	0.669	Oman	0.271	0.380	0.714	Maldives	0.213	0.308	0.693
Paraguay	0.297	0.444	0.669	Maldives	0.258	0.360	0.715	Peru	0.219	0.310	0.708
Syria	0.373	0.540	0.690	Syria	0.288	0.387	0.745	Israel	0.184	0.259	0.712
Nicaragua	0.333	0.480	0.693	Singapore	0.183	0.241	0.762	Niger	0.567	0.791	0.717
Cape Verde	0.426	0.610	0.699	Peru	0.254	0.331	0.767	Spain	0.173	0.239	0.723
Palestine	0.368	0.504	0.731	South Sudan	0.715	0.925	0.772	Singapore	0.164	0.225	0.730
Belize	0.345	0.466	0.740	Japan	0.182	0.233	0.782	Palestine	0.345	0.466	0.741
Djibouti	0.578	0.781	0.740	Spain	0.194	0.247	0.786	Sao Tome and Principe	0.360	0.480	0.749
North Korea	0.319	0.420	0.761	Israel	0.209	0.265	0.790	Japan	0.169	0.225	0.749
South Sudan	0.881	1.136	0.775	Cape Verde	0.353	0.444	0.795	Portugal	0.197	0.261	0.756
Honduras	0.423	0.535	0.791	Algeria	0.292	0.367	0.797	Ethiopia	0.458	0.598	0.766
Panama	0.280	0.351	0.798	North Korea	0.358	0.449	0.799	Nepal	0.359	0.466	0.771
Tunisia	0.358	0.444	0.805	Palestine	0.346	0.432	0.802	Colombia	0.230	0.298	0.771
Bosnia and Herzegovina	0.321	0.394	0.816	Panama	0.247	0.308	0.802	The Gambia	0.438	0.568	0.771
Yemen	0.686	0.835	0.822	Cuba	0.232	0.287	0.809	Italy	0.180	0.233	0.774
Jordan	0.359	0.436	0.823	Italy	0.193	0.239	0.809	South Sudan	0.624	0.801	0.779
Grenada	0.351	0.424	0.828	The Gambia	0.505	0.622	0.811	North Korea	0.338	0.428	0.791
Sao Tome and Principe	0.532	0.642	0.830	Sao Tome and Principe	0.440	0.540	0.814	Ecuador	0.248	0.312	0.793
Samoa	0.387	0.466	0.830	Tunisia	0.275	0.336	0.817	Rwanda	0.416	0.524	0.794
Algeria	0.378	0.453	0.834	Portugal	0.222	0.271	0.821	Bangladesh	0.333	0.416	0.801
Sri Lanka	0.349	0.416	0.839	Ecuador	0.280	0.339	0.826	Senegal	0.452	0.562	0.804
Japan	0.211	0.251	0.841	Niger	0.720	0.871	0.826	Sri Lanka	0.241	0.298	0.806
Jamaica	0.298	0.354	0.842	Honduras	0.344	0.416	0.827	Malta	0.191	0.237	0.807
The Gambia	0.581	0.689	0.843	Colombia	0.274	0.331	0.827	France	0.188	0.231	0.812
Vietnam	0.401	0.475	0.845	El Salvador	0.309	0.373	0.829	Timor-Leste	0.326	0.401	0.812
Albania	0.307	0.363	0.845	Paraguay	0.301	0.360	0.837	Cape Verde	0.306	0.376	0.813
Morocco	0.456	0.535	0.853	Chile	0.232	0.277	0.838	Sweden	0.188	0.231	0.815
Saint Vincent and the Grenadines	0.354	0.412	0.858	Sri Lanka	0.289	0.345	0.838	Cuba	0.218	0.267	0.818
Spain	0.242	0.281	0.862	Samoa	0.340	0.405	0.840	Switzerland	0.179	0.219	0.819
Kuwait	0.282	0.325	0.867	Nepal	0.455	0.540	0.842	Honduras	0.310	0.376	0.822
Malaysia	0.326	0.376	0.867	Malta	0.209	0.247	0.848	Albania	0.238	0.289	0.824
Venezuela	0.313	0.360	0.869	France	0.203	0.239	0.850	Greece	0.196	0.237	0.825
Qatar	0.297	0.342	0.869	Sweden	0.200	0.235	0.850	Panama	0.230	0.279	0.826
Pakistan	0.554	0.635	0.872	Bosnia and Herzegovina	0.258	0.303	0.852	Chile	0.214	0.259	0.827
El Salvador	0.413	0.471	0.876	Greece	0.207	0.243	0.852	Vietnam	0.274	0.331	0.828
United Arab Emirates	0.336	0.383	0.877	Andorra	0.188	0.219	0.857	China	0.240	0.289	0.830
Ecuador	0.354	0.401	0.882	Australia	0.200	0.233	0.860	Bosnia and Herzegovina	0.237	0.285	0.832
Colombia	0.343	0.387	0.886	Vietnam	0.319	0.370	0.861	Tunisia	0.252	0.303	0.832
Saint Lucia	0.340	0.383	0.888	Timor-Leste	0.418	0.485	0.863	El Salvador	0.275	0.328	0.839
Greece	0.243	0.273	0.890	Uruguay	0.260	0.300	0.865	Algeria	0.263	0.312	0.841
Tonga	0.412	0.462	0.893	Bangladesh	0.423	0.489	0.865	Australia	0.188	0.223	0.842
Guatemala	0.493	0.551	0.895	Iceland	0.192	0.221	0.868	Mali	0.571	0.675	0.846
Dominica	0.320	0.357	0.897	Guatemala	0.376	0.432	0.870	Thailand	0.244	0.287	0.848
Dominican Republic	0.364	0.405	0.900	New Zealand	0.213	0.243	0.877	Bermuda	0.213	0.251	0.850
Mexico	0.346	0.383	0.902	Morocco	0.346	0.394	0.878	New Zealand	0.198	0.233	0.851
Israel	0.257	0.285	0.903	Mexico	0.274	0.310	0.883	South Korea	0.194	0.227	0.854
Singapore	0.256	0.283	0.906	Albania	0.275	0.310	0.887	Qatar	0.225	0.263	0.854
Cuba	0.281	0.308	0.914	Turkey	0.272	0.305	0.890	Guatemala	0.324	0.380	0.854
Iraq	0.482	0.524	0.918	Switzerland	0.201	0.225	0.893	Austria	0.191	0.223	0.854
Malta	0.257	0.279	0.921	Austria	0.208	0.231	0.900	Liberia	0.511	0.598	0.855
Thailand	0.359	0.390	0.921	South Korea	0.217	0.241	0.900	Cyprus	0.198	0.231	0.857
Sweden	0.238	0.257	0.926	China	0.295	0.328	0.901	Samoa	0.326	0.380	0.859
Chile	0.306	0.328	0.933	United Kingdom	0.219	0.243	0.902	Mauritania	0.378	0.440	0.859
Burkina Faso	0.907	0.971	0.934	Bhutan	0.398	0.440	0.905	Uruguay	0.242	0.281	0.861
Uruguay	0.310	0.331	0.937	Venezuela	0.281	0.310	0.906	Norway	0.182	0.211	0.864
Portugal	0.289	0.308	0.939	Senegal	0.554	0.610	0.908	Iceland	0.183	0.211	0.864
Italy	0.245	0.259	0.944	Yemen	0.506	0.557	0.909	United Kingdom	0.203	0.233	0.870

Appendix Table 5. GBD 2016 DALYs exemplars for 1990, 2006, and 2016 on the basis of SDI											
1990				2006				2016			
Location	Observed Age Standardized DALYs	Expected Age Standardized DALYs	Ratio of Observed to Expected	Location	Observed Age Standardized DALYs	Expected Age Standardized DALYs	Ratio of Observed to Expected	Location	Observed Age Standardized DALYs	Expected Age Standardized DALYs	Ratio of Observed to Expected
Antigua and Barbuda	0.292	0.308	0.949	Saudi Arabia	0.301	0.331	0.909	Morocco	0.300	0.345	0.870
Andorra	0.219	0.231	0.949	Thailand	0.287	0.315	0.911	Turkey	0.240	0.275	0.872
France	0.247	0.259	0.954	Kuwait	0.256	0.281	0.911	Oman	0.255	0.292	0.875
Nepal	0.680	0.711	0.957	Mali	0.703	0.771	0.911	Ireland	0.197	0.225	0.877
Kenya	0.560	0.586	0.957	Comoros	0.493	0.540	0.913	Djibouti	0.440	0.499	0.882
Senegal	0.681	0.711	0.957	Cyprus	0.221	0.241	0.916	Andorra	0.188	0.213	0.882
Libya	0.337	0.351	0.960	Norway	0.201	0.219	0.917	Lebanon	0.225	0.255	0.884
Iceland	0.232	0.241	0.965	Djibouti	0.554	0.604	0.918	Benin	0.502	0.568	0.884
Cyprus	0.272	0.281	0.967	Canada	0.205	0.223	0.918	Burundi	0.588	0.662	0.888
Vanuatu	0.567	0.586	0.968	Jamaica	0.289	0.315	0.918	Germany	0.198	0.223	0.889
Mozambique	0.883	0.911	0.969	Lebanon	0.255	0.277	0.919	Slovenia	0.202	0.227	0.891
United Kingdom	0.265	0.273	0.971	Ethiopia	0.684	0.744	0.920	Burkina Faso	0.591	0.662	0.894
Comoros	0.647	0.662	0.978	Ireland	0.216	0.235	0.920	Taiwan, CHN	0.205	0.229	0.894
Northern Mariana Islands	0.292	0.298	0.978	Mauritania	0.464	0.504	0.920	Bhutan	0.323	0.360	0.896
Togo	0.687	0.696	0.987	Germany	0.214	0.233	0.920	Finland	0.195	0.217	0.899
Taiwan, CHN	0.282	0.285	0.987	Taiwan, CHN	0.226	0.245	0.923	Canada	0.194	0.215	0.902
Australia	0.249	0.251	0.994	Argentina	0.272	0.294	0.927	Comoros	0.422	0.466	0.904
Montenegro	0.277	0.279	0.994	United Arab Emirates	0.268	0.289	0.928	Netherlands	0.191	0.211	0.905
Argentina	0.329	0.331	0.994	Jordan	0.313	0.336	0.931	Kuwait	0.224	0.247	0.906
Canada	0.240	0.241	0.998	Qatar	0.264	0.283	0.933	Mexico	0.259	0.285	0.907
Bhutan	0.629	0.629	1.000	Malaysia	0.281	0.300	0.936	Tajikistan	0.336	0.370	0.908
Benin	0.753	0.753	1.001	Antigua and Barbuda	0.265	0.283	0.938	Bolivia	0.316	0.348	0.909
China	0.417	0.416	1.002	Rwanda	0.615	0.655	0.939	Belgium	0.200	0.219	0.914
Suriname	0.377	0.376	1.002	Dominican Republic	0.317	0.336	0.942	Luxembourg	0.188	0.205	0.916
Chad	0.814	0.812	1.003	Tonga	0.376	0.397	0.947	Dominican Republic	0.278	0.303	0.919
Ireland	0.273	0.271	1.009	Saint Lucia	0.296	0.312	0.948	Bahrain	0.260	0.283	0.919
Solomon Islands	0.612	0.604	1.014	Burkina Faso	0.741	0.781	0.949	Argentina	0.253	0.275	0.920
Mali	0.940	0.925	1.016	Bolivia	0.373	0.390	0.956	Jordan	0.279	0.303	0.920
Norway	0.246	0.241	1.021	Northern Mariana Islands	0.252	0.263	0.958	Paraguay	0.291	0.315	0.925
Barbados	0.296	0.289	1.022	Netherlands	0.210	0.219	0.958	Antigua and Barbuda	0.247	0.267	0.926
Mauritania	0.618	0.604	1.024	Belgium	0.218	0.227	0.959	Saint Lucia	0.274	0.294	0.932
Netherlands	0.245	0.239	1.027	Liberia	0.664	0.682	0.973	Armenia	0.259	0.277	0.933
Afghanistan	0.897	0.871	1.030	Luxembourg	0.207	0.213	0.974	Cambodia	0.394	0.420	0.937
Brazil	0.407	0.394	1.034	Madagascar	0.595	0.610	0.976	Democratic Republic of the Congo	0.578	0.616	0.938
Eritrea	0.841	0.812	1.036	Brazil	0.318	0.325	0.977	Jamaica	0.286	0.300	0.950
Peru	0.401	0.387	1.038	Dominica	0.301	0.305	0.986	Montenegro	0.241	0.253	0.954
Uzbekistan	0.406	0.390	1.040	Eritrea	0.584	0.592	0.987	Serbia	0.259	0.271	0.957
American Samoa	0.360	0.345	1.044	Benin	0.621	0.629	0.987	Malaysia	0.257	0.269	0.957
New Zealand	0.269	0.257	1.045	Belize	0.361	0.363	0.993	Saudi Arabia	0.250	0.261	0.958
Serbia	0.316	0.300	1.050	Bermuda	0.259	0.261	0.994	Brazil	0.284	0.296	0.960
Sudan	0.672	0.635	1.058	Montenegro	0.268	0.269	0.995	Eritrea	0.505	0.524	0.964
Madagascar	0.732	0.689	1.062	Barbados	0.266	0.267	0.996	Czech Republic	0.219	0.227	0.965
Brunei	0.299	0.281	1.063	Finland	0.225	0.225	1.001	Madagascar	0.533	0.551	0.967
Austria	0.263	0.247	1.063	Slovenia	0.237	0.237	1.002	Denmark	0.202	0.209	0.968
Switzerland	0.246	0.231	1.065	Sudan	0.500	0.499	1.003	Guinea	0.592	0.610	0.971
Iran	0.443	0.416	1.065	Saint Vincent and the Grenadines	0.344	0.339	1.013	Venezuela	0.275	0.283	0.971
Seychelles	0.374	0.351	1.066	Cambodia	0.511	0.504	1.014	Belize	0.329	0.339	0.971
Kiribati	0.664	0.622	1.067	Brunei	0.246	0.243	1.014	Croatia	0.234	0.239	0.979
Philippines	0.436	0.408	1.068	Libya	0.290	0.285	1.016	Chad	0.623	0.635	0.981
Tanzania	0.753	0.704	1.070	Serbia	0.294	0.285	1.030	Mozambique	0.612	0.622	0.983
Bahrain	0.391	0.363	1.076	Tajikistan	0.407	0.394	1.034	Iraq	0.441	0.444	0.993
Belgium	0.259	0.241	1.077	Seychelles	0.313	0.303	1.034	Barbados	0.259	0.261	0.994
Egypt	0.494	0.457	1.080	Burundi	0.769	0.744	1.035	Tonga	0.362	0.363	0.996
Germany	0.268	0.247	1.085	Armenia	0.314	0.303	1.038	Myanmar	0.364	0.360	1.010
Federated States of Micronesia	0.522	0.480	1.087	Grenada	0.342	0.328	1.042	Puerto Rico	0.232	0.229	1.013
Marshall Islands	0.494	0.453	1.090	Democratic Republic of the Congo	0.742	0.711	1.044	Northern Mariana Islands	0.264	0.261	1.013

Appendix Table 5. GBD 2016 DALYs exemplars for 1990, 2006, and 2016 on the basis of SDI											
1990				2006				2016			
Location	Observed Age Standardized DALYs	Expected Age Standardized DALYs	Ratio of Observed to Expected	Location	Observed Age Standardized DALYs	Expected Age Standardized DALYs	Ratio of Observed to Expected	Location	Observed Age Standardized DALYs	Expected Age Standardized DALYs	Ratio of Observed to Expected
Mauritius	0.379	0.348	1.090	Bahrain	0.311	0.298	1.045	Poland	0.234	0.231	1.015
Guam	0.302	0.277	1.091	Georgia	0.315	0.300	1.047	United Arab Emirates	0.275	0.271	1.015
Cambodia	0.686	0.629	1.091	Somalia	0.781	0.744	1.050	Uganda	0.549	0.540	1.017
Niger	1.012	0.925	1.094	Egypt	0.379	0.360	1.051	Tanzania	0.493	0.485	1.017
Puerto Rico	0.299	0.273	1.095	Guinea	0.710	0.675	1.052	Yemen	0.475	0.466	1.018
Somalia	0.889	0.812	1.095	Denmark	0.228	0.217	1.052	Kyrgyzstan	0.342	0.336	1.018
Lesotho	0.599	0.546	1.098	Croatia	0.262	0.249	1.054	Seychelles	0.290	0.285	1.018
Turkey	0.407	0.370	1.102	Czech Republic	0.251	0.237	1.058	Angola	0.494	0.485	1.019
Bangladesh	0.672	0.610	1.102	Afghanistan	0.818	0.771	1.061	Somalia	0.684	0.669	1.023
Armenia	0.364	0.328	1.110	Macedonia	0.298	0.277	1.076	Macedonia	0.269	0.261	1.030
Timor-Leste	0.670	0.598	1.122	Mauritius	0.318	0.296	1.076	Laos	0.429	0.416	1.031
South Korea	0.320	0.283	1.130	Puerto Rico	0.254	0.235	1.080	Mauritius	0.286	0.277	1.034
Guinea	0.855	0.753	1.136	Poland	0.270	0.249	1.084	Togo	0.537	0.519	1.035
Myanmar	0.646	0.568	1.138	Pakistan	0.512	0.471	1.087	Georgia	0.289	0.279	1.037
Rwanda	0.942	0.823	1.145	Indonesia	0.388	0.354	1.097	Haiti	0.506	0.485	1.044
Uganda	0.956	0.835	1.145	United States	0.249	0.227	1.098	Iran	0.283	0.271	1.044
Finland	0.279	0.243	1.146	Solomon Islands	0.585	0.530	1.105	Egypt	0.338	0.323	1.046
Cameroon	0.664	0.580	1.146	Chad	0.788	0.711	1.108	Brunei	0.243	0.231	1.051
Maldives	0.551	0.480	1.147	American Samoa	0.337	0.303	1.113	Pakistan	0.429	0.408	1.051
Ghana	0.645	0.562	1.148	Vanuatu	0.548	0.489	1.120	Saint Vincent and the Grenadines	0.335	0.317	1.054
India	0.648	0.562	1.152	Myanmar	0.494	0.440	1.121	Malawi	0.600	0.568	1.056
Virgin Islands, U.S.	0.311	0.269	1.158	The Bahamas	0.305	0.271	1.125	Afghanistan	0.695	0.655	1.060
Zimbabwe	0.562	0.485	1.159	Philippines	0.405	0.360	1.125	Indonesia	0.331	0.312	1.061
Namibia	0.568	0.489	1.160	Mozambique	0.828	0.727	1.139	Sudan	0.435	0.408	1.066
Cote d'Ivoire	0.769	0.662	1.162	Suriname	0.384	0.336	1.141	Kenya	0.436	0.408	1.067
Papua New Guinea	0.739	0.635	1.164	Iran	0.333	0.292	1.143	Moldova	0.318	0.298	1.068
Macedonia	0.353	0.300	1.173	India	0.505	0.440	1.147	Ghana	0.455	0.424	1.073
Bolivia	0.575	0.489	1.176	Slovakia	0.280	0.241	1.162	Slovakia	0.244	0.227	1.075
United States	0.282	0.239	1.179	Moldova	0.381	0.328	1.163	Estonia	0.244	0.225	1.083
Swaziland	0.546	0.462	1.183	Kiribati	0.606	0.519	1.168	Philippines	0.361	0.331	1.092
Ethiopia	1.046	0.884	1.183	Guam	0.302	0.257	1.173	Hungary	0.261	0.239	1.094
Slovenia	0.300	0.253	1.186	Romania	0.312	0.265	1.178	United States	0.240	0.219	1.095
Botswana	0.495	0.416	1.189	Angola	0.698	0.592	1.181	Dominica	0.312	0.285	1.095
Tajikistan	0.486	0.408	1.189	Bulgaria	0.307	0.259	1.184	Guinea-Bissau	0.645	0.586	1.101
Sierra Leone	0.897	0.753	1.191	Hungary	0.299	0.251	1.191	Sierra Leone	0.632	0.568	1.113
Trinidad and Tobago	0.386	0.323	1.196	Trinidad and Tobago	0.343	0.287	1.192	Romania	0.271	0.243	1.115
Denmark	0.272	0.227	1.198	Togo	0.693	0.580	1.195	The Bahamas	0.296	0.265	1.115
Indonesia	0.508	0.424	1.198	Laos	0.579	0.480	1.206	Bulgaria	0.270	0.241	1.120
Croatia	0.318	0.265	1.199	Iraq	0.528	0.436	1.211	Grenada	0.335	0.298	1.124
Lebanon	0.409	0.339	1.205	Guinea-Bissau	0.795	0.655	1.213	Solomon Islands	0.530	0.471	1.127
Luxembourg	0.276	0.229	1.207	Uganda	0.798	0.655	1.219	India	0.414	0.363	1.139
Guyana	0.498	0.412	1.208	Haiti	0.654	0.535	1.223	Libya	0.293	0.257	1.140
Poland	0.347	0.285	1.216	Virgin Islands, U.S.	0.291	0.237	1.228	American Samoa	0.325	0.283	1.147
The Bahamas	0.355	0.292	1.219	Papua New Guinea	0.685	0.557	1.230	Nigeria	0.506	0.440	1.150
Bulgaria	0.337	0.275	1.224	Kyrgyzstan	0.437	0.351	1.244	Vanuatu	0.507	0.440	1.151
Malawi	0.954	0.771	1.238	Federated States of Micronesia	0.461	0.370	1.248	Suriname	0.351	0.303	1.159
Guinea-Bissau	0.943	0.762	1.238	Tanzania	0.716	0.574	1.248	Latvia	0.278	0.237	1.172
Burundi	0.996	0.801	1.243	Sierra Leone	0.822	0.655	1.254	Trinidad and Tobago	0.321	0.273	1.175
Democratic Republic of the Congo	0.807	0.648	1.244	Ghana	0.593	0.471	1.260	Uzbekistan	0.357	0.300	1.190
Liberia	0.926	0.744	1.245	Uzbekistan	0.424	0.336	1.262	Azerbaijan	0.326	0.273	1.194
Belarus	0.354	0.283	1.252	Estonia	0.308	0.239	1.287	Kiribati	0.562	0.471	1.194
Haiti	0.784	0.622	1.259	Marshall Islands	0.536	0.408	1.312	Kazakhstan	0.332	0.277	1.199
Georgia	0.370	0.292	1.270	Nigeria	0.693	0.524	1.322	Belarus	0.299	0.249	1.202
Bermuda	0.363	0.285	1.272	Azerbaijan	0.404	0.305	1.323	Papua New Guinea	0.594	0.494	1.203
Nigeria	0.771	0.604	1.278	Guyana	0.481	0.363	1.324	Cote d'Ivoire	0.614	0.509	1.206
Czech Republic	0.330	0.255	1.293	Kenya	0.639	0.471	1.357	Virgin Islands, U.S.	0.279	0.229	1.217

Appendix Table 5. GBD 2016 DALYs exemplars for 1990, 2006, and 2016 on the basis of SDI

1990				2006				2016			
Location	Observed Age Standardized DALYs	Expected Age Standardized DALYs	Ratio of Observed to Expected	Location	Observed Age Standardized DALYs	Expected Age Standardized DALYs	Ratio of Observed to Expected	Location	Observed Age Standardized DALYs	Expected Age Standardized DALYs	Ratio of Observed to Expected
Slovakia	0.344	0.265	1.297	Belarus	0.377	0.271	1.393	Lithuania	0.279	0.229	1.220
Romania	0.373	0.287	1.299	Latvia	0.354	0.251	1.411	Ukraine	0.325	0.261	1.244
Moldova	0.426	0.328	1.299	Cote d'Ivoire	0.787	0.557	1.413	Guam	0.319	0.251	1.271
Lithuania	0.343	0.263	1.303	Lithuania	0.347	0.243	1.426	Guyana	0.416	0.325	1.280
Kyrgyzstan	0.458	0.351	1.305	Turkmenistan	0.453	0.312	1.449	Cameroon	0.604	0.466	1.295
Angola	0.911	0.696	1.309	Cameroon	0.747	0.509	1.468	Syria	0.430	0.331	1.301
Ukraine	0.367	0.279	1.314	Ukraine	0.407	0.277	1.470	Mongolia	0.400	0.308	1.301
Zambia	0.830	0.629	1.320	Malawi	0.980	0.648	1.511	Turkmenistan	0.359	0.275	1.304
Laos	0.823	0.622	1.323	Mongolia	0.488	0.323	1.514	Marshall Islands	0.487	0.370	1.316
Kazakhstan	0.412	0.310	1.329	Congo (B)	0.692	0.453	1.528	Gabon	0.454	0.342	1.327
Fiji	0.474	0.354	1.340	Gabon	0.602	0.376	1.599	Federated States of Micronesia	0.464	0.348	1.333
Hungary	0.377	0.277	1.361	Fiji	0.487	0.303	1.607	Congo (B)	0.537	0.401	1.340
Turkmenistan	0.505	0.370	1.366	Kazakhstan	0.470	0.292	1.613	Zambia	0.645	0.457	1.410
Gabon	0.634	0.457	1.386	Russia	0.430	0.259	1.662	Russia	0.350	0.247	1.416
Mongolia	0.536	0.383	1.399	Greenland	0.452	0.265	1.706	Namibia	0.489	0.339	1.441
Estonia	0.368	0.259	1.419	Equatorial Guinea	0.645	0.367	1.760	Greenland	0.368	0.253	1.456
Azerbaijan	0.447	0.315	1.420	Central African Republic	1.131	0.635	1.780	Zimbabwe	0.625	0.428	1.460
Latvia	0.379	0.265	1.431	Zambia	1.078	0.540	1.995	Central African Republic	0.937	0.610	1.536
Central African Republic	1.010	0.704	1.436	Namibia	0.841	0.376	2.236	Equatorial Guinea	0.484	0.315	1.537
Russia	0.387	0.269	1.437	Zimbabwe	1.141	0.432	2.643	Botswana	0.480	0.300	1.597
South Africa	0.514	0.357	1.441	Lesotho	1.261	0.444	2.837	Fiji	0.469	0.285	1.644
Congo (B)	0.811	0.535	1.516	South Africa	0.887	0.310	2.863	Swaziland	0.691	0.360	1.919
Equatorial Guinea	0.997	0.642	1.554	Botswana	0.990	0.331	2.993	South Africa	0.558	0.285	1.955
Greenland	0.601	0.279	2.153	Swaziland	1.291	0.397	3.250	Lesotho	0.939	0.387	2.427

Note: Congo (B) = Congo (Brazzaville); Taiwan, CHN = Taiwan, Province of China.

Appendix Table 6: Socio-Demographic Index groupings by location, based on 2016 values

Location	SDI Level
Aichi	High SDI
Akita	High SDI
Alabama	High SDI
Alaska	High SDI
Andorra	High SDI
Aomori	High SDI
Arizona	High SDI
Arkansas	High SDI
Australia	High SDI
Austria	High SDI
Barking and Dagenham	High SDI
Barnet	High SDI
Barnsley	High SDI
Bath and North East Somerset	High SDI
Bedford	High SDI
Belgium	High SDI
Bexley	High SDI
Birmingham	High SDI
Blackburn with Darwen	High SDI
Blackpool	High SDI
Bolton	High SDI
Bournemouth	High SDI
Bracknell Forest	High SDI
Bradford	High SDI
Brent	High SDI
Brighton and Hove	High SDI
Bristol, City of	High SDI
Bromley	High SDI
Brunei	High SDI
Buckinghamshire	High SDI
Bury	High SDI
Calderdale	High SDI
California	High SDI
Cambridgeshire	High SDI
Camden	High SDI
Canada	High SDI
Central Bedfordshire	High SDI
Cheshire East	High SDI
Cheshire West and Chester	High SDI
Chiba	High SDI
Colorado	High SDI
Connecticut	High SDI
Cornwall	High SDI
County Durham	High SDI
Coventry	High SDI

Appendix Table 6: Socio-Demographic Index groupings by location, based on 2016 values	
**Location**	**SDI Level**
Croatia	High SDI
Croydon	High SDI
Cumbria	High SDI
Cyprus	High SDI
Czech Republic	High SDI
Darlington	High SDI
Delaware	High SDI
Denmark	High SDI
Derby	High SDI
Derbyshire	High SDI
Devon	High SDI
District of Columbia	High SDI
Doncaster	High SDI
Dorset	High SDI
Dudley	High SDI
Ealing	High SDI
East Riding of Yorkshire	High SDI
East Sussex	High SDI
Ehime	High SDI
Enfield	High SDI
Essex	High SDI
Estonia	High SDI
Finland	High SDI
Florida	High SDI
France	High SDI
Fukui	High SDI
Fukuoka	High SDI
Fukushima	High SDI
Gateshead	High SDI
Georgia	High SDI
Germany	High SDI
Gifu	High SDI
Gloucestershire	High SDI
Greece	High SDI
Greenwich	High SDI
Gunma	High SDI
Hackney	High SDI
Halton	High SDI
Hammersmith and Fulham	High SDI
Hampshire	High SDI
Haringey	High SDI
Harrow	High SDI
Hartlepool	High SDI
Havering	High SDI
Hawaii	High SDI

Appendix Table 6: Socio-Demographic Index groupings by location, based on 2016 values

Location	SDI Level
Herefordshire, County of	High SDI
Hertfordshire	High SDI
Hillingdon	High SDI
Hiroshima	High SDI
Hokkaidō	High SDI
Hounslow	High SDI
Hyōgo	High SDI
Ibaraki	High SDI
Iceland	High SDI
Idaho	High SDI
Illinois	High SDI
Indiana	High SDI
Iowa	High SDI
Ireland	High SDI
Ishikawa	High SDI
Isle of Wight	High SDI
Islington	High SDI
Italy	High SDI
Iwate	High SDI
Kagawa	High SDI
Kagoshima	High SDI
Kanagawa	High SDI
Kansas	High SDI
Kensington and Chelsea	High SDI
Kent	High SDI
Kentucky	High SDI
Kingston upon Hull, City of	High SDI
Kingston upon Thames	High SDI
Kirklees	High SDI
Knowsley	High SDI
Kumamoto	High SDI
Kyōto	High SDI
Kōchi	High SDI
Lambeth	High SDI
Lancashire	High SDI
Latvia	High SDI
Leeds	High SDI
Leicester	High SDI
Leicestershire	High SDI
Lewisham	High SDI
Lincolnshire	High SDI
Lithuania	High SDI
Liverpool	High SDI
Louisiana	High SDI
Luton	High SDI

Appendix Table 6: Socio-Demographic Index groupings by location, based on 2016 values	
**Location**	**SDI Level**
Luxembourg	High SDI
Maine	High SDI
Malta	High SDI
Manchester	High SDI
Maryland	High SDI
Massachusetts	High SDI
Medway	High SDI
Merton	High SDI
Michigan	High SDI
Middlesbrough	High SDI
Mie	High SDI
Milton Keynes	High SDI
Minnesota	High SDI
Mississippi	High SDI
Missouri	High SDI
Miyagi	High SDI
Miyazaki	High SDI
Montana	High SDI
Nagano	High SDI
Nagasaki	High SDI
Nara	High SDI
Nebraska	High SDI
Netherlands	High SDI
Nevada	High SDI
New Hampshire	High SDI
New Jersey	High SDI
New Mexico	High SDI
New York	High SDI
New Zealand	High SDI
Newcastle upon Tyne	High SDI
Newham	High SDI
Niigata	High SDI
Norfolk	High SDI
North Carolina	High SDI
North Dakota	High SDI
North East Lincolnshire	High SDI
North Lincolnshire	High SDI
North Somerset	High SDI
North Tyneside	High SDI
North Yorkshire	High SDI
Northamptonshire	High SDI
Northern Ireland	High SDI
Northumberland	High SDI
Norway	High SDI
Nottingham	High SDI

Appendix Table 6: Socio-Demographic Index groupings by location, based on 2016 values	
**Location**	**SDI Level**
Nottinghamshire	High SDI
Ohio	High SDI
Okayama	High SDI
Okinawa	High SDI
Oklahoma	High SDI
Oldham	High SDI
Oregon	High SDI
Oxfordshire	High SDI
Pennsylvania	High SDI
Peterborough	High SDI
Plymouth	High SDI
Poland	High SDI
Poole	High SDI
Portsmouth	High SDI
Puerto Rico	High SDI
Reading	High SDI
Redbridge	High SDI
Redcar and Cleveland	High SDI
Rhode Island	High SDI
Richmond upon Thames	High SDI
Rochdale	High SDI
Rotherham	High SDI
Rutland	High SDI
Saga	High SDI
Saitama	High SDI
Salford	High SDI
Sandwell	High SDI
Scotland	High SDI
Sefton	High SDI
Sheffield	High SDI
Shiga	High SDI
Shimane	High SDI
Shizuoka	High SDI
Shropshire	High SDI
Singapore	High SDI
Slough	High SDI
Slovakia	High SDI
Slovenia	High SDI
Solihull	High SDI
Somerset	High SDI
South Carolina	High SDI
South Dakota	High SDI
South Gloucestershire	High SDI
South Korea	High SDI
South Tyneside	High SDI

Appendix Table 6: Socio-Demographic Index groupings by location, based on 2016 values	
**Location**	**SDI Level**
Southampton	High SDI
Southend-on-Sea	High SDI
Southwark	High SDI
St Helens	High SDI
Staffordshire	High SDI
Stockholm	High SDI
Stockport	High SDI
Stockton-on-Tees	High SDI
Stoke-on-Trent	High SDI
Suffolk	High SDI
Sunderland	High SDI
Surrey	High SDI
Sutton	High SDI
Sweden except Stockholm	High SDI
Swindon	High SDI
Switzerland	High SDI
Taiwan (province of China)	High SDI
Tameside	High SDI
Telford and Wrekin	High SDI
Tennessee	High SDI
Texas	High SDI
Thurrock	High SDI
Tochigi	High SDI
Tokushima	High SDI
Torbay	High SDI
Tottori	High SDI
Tower Hamlets	High SDI
Toyama	High SDI
Trafford	High SDI
Tōkyō	High SDI
Utah	High SDI
Vermont	High SDI
Virgin Islands	High SDI
Virginia	High SDI
Wakayama	High SDI
Wakefield	High SDI
Wales	High SDI
Walsall	High SDI
Waltham Forest	High SDI
Wandsworth	High SDI
Warrington	High SDI
Warwickshire	High SDI
Washington	High SDI
West Berkshire	High SDI
West Sussex	High SDI

Appendix Table 6: Socio-Demographic Index groupings by location, based on 2016 values	
**Location**	**SDI Level**
West Virginia	High SDI
Westminster	High SDI
Wigan	High SDI
Wiltshire	High SDI
Windsor and Maidenhead	High SDI
Wirral	High SDI
Wisconsin	High SDI
Wokingham	High SDI
Wolverhampton	High SDI
Worcestershire	High SDI
Wyoming	High SDI
Yamagata	High SDI
Yamaguchi	High SDI
Yamanashi	High SDI
York	High SDI
Ôita	High SDI
Ōsaka	High SDI
'Asir	High-middle SDI
Antigua and Barbuda	High-middle SDI
Argentina	High-middle SDI
Armenia	High-middle SDI
Azerbaijan	High-middle SDI
Bahah	High-middle SDI
Barbados	High-middle SDI
Beijing	High-middle SDI
Belarus	High-middle SDI
Bermuda	High-middle SDI
Bulgaria	High-middle SDI
Chile	High-middle SDI
Cuba	High-middle SDI
Delhi, Urban	High-middle SDI
Distrito Federal	High-middle SDI
Eastern Province	High-middle SDI
Georgia	High-middle SDI
Goa, Urban	High-middle SDI
Greenland	High-middle SDI
Guam	High-middle SDI
Guangdong	High-middle SDI
Ha'il	High-middle SDI
Himachal Pradesh, Urban	High-middle SDI
Hong Kong Special Administrative Region of China	High-middle SDI
Hungary	High-middle SDI
Iran	High-middle SDI
Israel	High-middle SDI

Appendix Table 6: Socio-Demographic Index groupings by location, based on 2016 values

Location	SDI Level
Jakarta	High-middle SDI
Jawf	High-middle SDI
Jiangsu	High-middle SDI
Jizan	High-middle SDI
East Kalimantan	High-middle SDI
North Kalimantan	High-middle SDI
Kazakhstan	High-middle SDI
Riau Islands	High-middle SDI
Kuwait	High-middle SDI
Lebanon	High-middle SDI
Libya	High-middle SDI
Macao Special Administrative Region of China	High-middle SDI
Macedonia	High-middle SDI
Madinah	High-middle SDI
Makkah	High-middle SDI
Malaysia	High-middle SDI
Mauritius	High-middle SDI
Montenegro	High-middle SDI
Najran	High-middle SDI
Northern Borders	High-middle SDI
Northern Mariana Islands	High-middle SDI
Panama	High-middle SDI
Portugal	High-middle SDI
Qassim	High-middle SDI
Qatar	High-middle SDI
Rio de Janeiro	High-middle SDI
Riyadh	High-middle SDI
Romania	High-middle SDI
Russia	High-middle SDI
Serbia	High-middle SDI
Shanghai	High-middle SDI
Spain	High-middle SDI
São Paulo	High-middle SDI
Tabuk	High-middle SDI
The Bahamas	High-middle SDI
Tianjin	High-middle SDI
Trinidad and Tobago	High-middle SDI
Turkey	High-middle SDI
Turkmenistan	High-middle SDI
Ukraine	High-middle SDI
United Arab Emirates	High-middle SDI
Zhejiang	High-middle SDI
Aceh	Middle SDI
Acre	Middle SDI

Appendix Table 6: Socio-Demographic Index groupings by location, based on 2016 values

Location	SDI Level
Aguascalientes	Middle SDI
Albania	Middle SDI
Algeria	Middle SDI
Amapá	Middle SDI
Amazonas	Middle SDI
American Samoa	Middle SDI
Andhra Pradesh, Urban	Middle SDI
Anhui	Middle SDI
Assam, Urban	Middle SDI
Bahia	Middle SDI
Bahrain	Middle SDI
Baja California	Middle SDI
Baja California Sur	Middle SDI
Bali	Middle SDI
Bangka–Belitung Islands	Middle SDI
Banten	Middle SDI
Bengkulu	Middle SDI
Bosnia and Herzegovina	Middle SDI
Botswana	Middle SDI
Campeche	Middle SDI
Chhattisgarh, Urban	Middle SDI
Chiapas	Middle SDI
Chihuahua	Middle SDI
Chongqing	Middle SDI
Coahuila	Middle SDI
Colima	Middle SDI
Colombia	Middle SDI
Costa Rica	Middle SDI
Delhi, Rural	Middle SDI
Distrito Federal	Middle SDI
Dominica	Middle SDI
Dominican Republic	Middle SDI
Durango	Middle SDI
Eastern Cape	Middle SDI
Ecuador	Middle SDI
Egypt	Middle SDI
El Salvador	Middle SDI
Equatorial Guinea	Middle SDI
Espírito Santo	Middle SDI
Fiji	Middle SDI
Free State	Middle SDI
Fujian	Middle SDI
Gauteng	Middle SDI
Goa, Rural	Middle SDI
Goiás	Middle SDI

Appendix Table 6: Socio-Demographic Index groupings by location, based on 2016 values	
**Location**	**SDI Level**
Grenada	Middle SDI
Guanajuato	Middle SDI
Guangxi	Middle SDI
Guerrero	Middle SDI
Gujarat, Urban	Middle SDI
Guyana	Middle SDI
Hainan	Middle SDI
Haryana, Urban	Middle SDI
Hebei	Middle SDI
Heilongjiang	Middle SDI
Henan	Middle SDI
Hidalgo	Middle SDI
Himachal Pradesh, Rural	Middle SDI
Hubei	Middle SDI
Hunan	Middle SDI
Inner Mongolia	Middle SDI
Jalisco	Middle SDI
Jamaica	Middle SDI
Jambi	Middle SDI
Jammu and Kashmir, Urban	Middle SDI
West Java	Middle SDI
East Java	Middle SDI
Jharkhand, Urban	Middle SDI
Jiangxi	Middle SDI
Jilin	Middle SDI
Jordan	Middle SDI
South Kalimantan	Middle SDI
Central Kalimantan	Middle SDI
Karnataka, Urban	Middle SDI
Kerala, Rural	Middle SDI
Kerala, Urban	Middle SDI
KwaZulu-Natal	Middle SDI
Lampung	Middle SDI
Liaoning	Middle SDI
Limpopo	Middle SDI
Madhya Pradesh, Urban	Middle SDI
Maharashtra, Urban	Middle SDI
Maldives	Middle SDI
Manipur, Urban	Middle SDI
Mato Grosso	Middle SDI
Mato Grosso do Sul	Middle SDI
Meghalaya, Urban	Middle SDI
Michoacán de Ocampo	Middle SDI
Minas Gerais	Middle SDI
Mizoram, Urban	Middle SDI

Appendix Table 6: Socio-Demographic Index groupings by location, based on 2016 values	
**Location**	**SDI Level**
Moldova	Middle SDI
Mongolia	Middle SDI
Morelos	Middle SDI
Mpumalanga	Middle SDI
México	Middle SDI
Nagaland, Urban	Middle SDI
Nayarit	Middle SDI
Ningxia	Middle SDI
North-West	Middle SDI
Northern Cape	Middle SDI
Nuevo León	Middle SDI
Oaxaca	Middle SDI
Oman	Middle SDI
West Papua	Middle SDI
Paraguay	Middle SDI
Paraná	Middle SDI
Pernambuco	Middle SDI
Peru	Middle SDI
Philippines	Middle SDI
Puebla	Middle SDI
Punjab, Urban	Middle SDI
Qinghai	Middle SDI
Querétaro	Middle SDI
Quintana Roo	Middle SDI
Riau	Middle SDI
Rio Grande do Norte	Middle SDI
Rio Grande do Sul	Middle SDI
Rondônia	Middle SDI
Roraima	Middle SDI
Saint Lucia	Middle SDI
Saint Vincent and the Grenadines	Middle SDI
San Luis Potosí	Middle SDI
Santa Catarina	Middle SDI
Sergipe	Middle SDI
Seychelles	Middle SDI
Shaanxi	Middle SDI
Shandong	Middle SDI
Shanxi	Middle SDI
Sichuan	Middle SDI
Sikkim, Urban	Middle SDI
Sinaloa	Middle SDI
Sonora	Middle SDI
Sri Lanka	Middle SDI
South Sulawesi	Middle SDI
North Sulawesi	Middle SDI

Appendix Table 6: Socio-Demographic Index groupings by location, based on 2016 values	
**Location**	**SDI Level**
West Sumatra	Middle SDI
South Sumatra	Middle SDI
North Sumatra	Middle SDI
Suriname	Middle SDI
Tabasco	Middle SDI
Tamaulipas	Middle SDI
Tamil Nadu, Urban	Middle SDI
Telangana, Urban	Middle SDI
Thailand	Middle SDI
The Six Minor Territories, Urban	Middle SDI
Tlaxcala	Middle SDI
Tocantins	Middle SDI
Tunisia	Middle SDI
Uruguay	Middle SDI
Uttarakhand, Urban	Middle SDI
Uzbekistan	Middle SDI
Venezuela	Middle SDI
Veracruz de Ignacio de la Llave	Middle SDI
Vietnam	Middle SDI
West Bengal, Urban	Middle SDI
Western Cape	Middle SDI
Xinjiang	Middle SDI
Yogyakarta	Middle SDI
Yucatán	Middle SDI
Yunnan	Middle SDI
Zacatecas	Middle SDI
Alagoas	Low-middle SDI
Andhra Pradesh, Rural	Low-middle SDI
Arunachal Pradesh, Rural	Low-middle SDI
Arunachal Pradesh, Urban	Low-middle SDI
Assam, Rural	Low-middle SDI
Bangladesh	Low-middle SDI
Baringo	Low-middle SDI
Belize	Low-middle SDI
Bhutan	Low-middle SDI
Bihar, Urban	Low-middle SDI
Bolivia	Low-middle SDI
Bomet	Low-middle SDI
Bungoma	Low-middle SDI
Busia	Low-middle SDI
Cambodia	Low-middle SDI
Cameroon	Low-middle SDI
Cape Verde	Low-middle SDI
Ceará	Low-middle SDI
Chhattisgarh, Rural	Low-middle SDI

Appendix Table 6: Socio-Demographic Index groupings by location, based on 2016 values	
**Location**	**SDI Level**
Congo (Brazzaville)	Low-middle SDI
Elgeyo-Marakwet	Low-middle SDI
Embu	Low-middle SDI
Federated States of Micronesia	Low-middle SDI
Gabon	Low-middle SDI
Gansu	Low-middle SDI
Garissa	Low-middle SDI
Ghana	Low-middle SDI
Gorontalo	Low-middle SDI
Guatemala	Low-middle SDI
Guizhou	Low-middle SDI
Gujarat, Rural	Low-middle SDI
Haryana, Rural	Low-middle SDI
HomaBay	Low-middle SDI
Honduras	Low-middle SDI
Iraq	Low-middle SDI
Isiolo	Low-middle SDI
Jammu and Kashmir, Rural	Low-middle SDI
Central Java	Low-middle SDI
Jharkhand, Rural	Low-middle SDI
Kajiado	Low-middle SDI
Kakamega	Low-middle SDI
West Kalimantan	Low-middle SDI
Karnataka, Rural	Low-middle SDI
Kericho	Low-middle SDI
Kiambu	Low-middle SDI
Kilifi	Low-middle SDI
Kirinyaga	Low-middle SDI
Kisii	Low-middle SDI
Kisumu	Low-middle SDI
Kitui	Low-middle SDI
Kwale	Low-middle SDI
Kyrgyzstan	Low-middle SDI
Laikipia	Low-middle SDI
Lamu	Low-middle SDI
Laos	Low-middle SDI
Lesotho	Low-middle SDI
Machakos	Low-middle SDI
Madhya Pradesh, Rural	Low-middle SDI
Maharashtra, Rural	Low-middle SDI
Makueni	Low-middle SDI
Maluku	Low-middle SDI
North Maluku	Low-middle SDI
Mandera	Low-middle SDI
Manipur, Rural	Low-middle SDI

Appendix Table 6: Socio-Demographic Index groupings by location, based on 2016 values	
**Location**	**SDI Level**
Maranhão	Low-middle SDI
Marsabit	Low-middle SDI
Marshall Islands	Low-middle SDI
Mauritania	Low-middle SDI
Meghalaya, Rural	Low-middle SDI
Meru	Low-middle SDI
Migori	Low-middle SDI
Mizoram, Rural	Low-middle SDI
Mombasa	Low-middle SDI
Morocco	Low-middle SDI
Murang'a	Low-middle SDI
Myanmar	Low-middle SDI
Nagaland, Rural	Low-middle SDI
Nairobi	Low-middle SDI
Nakuru	Low-middle SDI
Namibia	Low-middle SDI
Nandi	Low-middle SDI
Narok	Low-middle SDI
Nepal	Low-middle SDI
Nicaragua	Low-middle SDI
Nigeria	Low-middle SDI
North Korea	Low-middle SDI
West Nusa Tenggara	Low-middle SDI
East Nusa Tenggara	Low-middle SDI
Nyamira	Low-middle SDI
Nyandarua	Low-middle SDI
Nyeri	Low-middle SDI
Odisha, Rural	Low-middle SDI
Odisha, Urban	Low-middle SDI
Pakistan	Low-middle SDI
Papua	Low-middle SDI
Paraíba	Low-middle SDI
Pará	Low-middle SDI
Piaui	Low-middle SDI
Punjab, Rural	Low-middle SDI
Rajasthan, Rural	Low-middle SDI
Rajasthan, Urban	Low-middle SDI
Samburu	Low-middle SDI
Samoa	Low-middle SDI
Siaya	Low-middle SDI
Sikkim, Rural	Low-middle SDI
Sudan	Low-middle SDI
West Sulawesi	Low-middle SDI
Central Sulawesi	Low-middle SDI
Southeast Sulawesi	Low-middle SDI

Appendix Table 6: Socio-Demographic Index groupings by location, based on 2016 values	
**Location**	**SDI Level**
Swaziland	Low-middle SDI
Syria	Low-middle SDI
TaitaTaveta	Low-middle SDI
Tajikistan	Low-middle SDI
Tamil Nadu, Rural	Low-middle SDI
TanaRiver	Low-middle SDI
Telangana, Rural	Low-middle SDI
TharakaNithi	Low-middle SDI
The Six Minor Territories, Rural	Low-middle SDI
Tibet	Low-middle SDI
Timor-Leste	Low-middle SDI
Tonga	Low-middle SDI
TransNzoia	Low-middle SDI
Tripura, Rural	Low-middle SDI
Tripura, Urban	Low-middle SDI
Turkana	Low-middle SDI
UasinGishu	Low-middle SDI
Uttar Pradesh, Rural	Low-middle SDI
Uttar Pradesh, Urban	Low-middle SDI
Uttarakhand, Rural	Low-middle SDI
Vanuatu	Low-middle SDI
Vihiga	Low-middle SDI
Wajir	Low-middle SDI
West Bengal, Rural	Low-middle SDI
WestPokot	Low-middle SDI
Zambia	Low-middle SDI
Zimbabwe	Low-middle SDI
Afghanistan	Low SDI
Angola	Low SDI
Benin	Low SDI
Bihar, Rural	Low SDI
Burkina Faso	Low SDI
Burundi	Low SDI
Central African Republic	Low SDI
Chad	Low SDI
Comoros	Low SDI
Cote d'Ivoire	Low SDI
Democratic Republic of the Congo	Low SDI
Djibouti	Low SDI
Eritrea	Low SDI
Ethiopia	Low SDI
Guinea	Low SDI
Guinea-Bissau	Low SDI
Haiti	Low SDI
Kiribati	Low SDI

Appendix Table 6: Socio-Demographic Index groupings by location, based on 2016 values

Location	SDI Level
Liberia	Low SDI
Madagascar	Low SDI
Malawi	Low SDI
Mali	Low SDI
Mozambique	Low SDI
Niger	Low SDI
Palestine	Low SDI
Papua New Guinea	Low SDI
Rwanda	Low SDI
Sao Tome and Principe	Low SDI
Senegal	Low SDI
Sierra Leone	Low SDI
Solomon Islands	Low SDI
Somalia	Low SDI
South Sudan	Low SDI
Tanzania	Low SDI
The Gambia	Low SDI
Togo	Low SDI
Uganda	Low SDI
Yemen	Low SDI

Appendix Table 7: Socio-demographic Index values for all estimated GBD 2016 locations, 1980–2016

Location	1980	1981	1982	1983	1984	1985	1986	1987	1988	1989	1990	1991	1992	1993	1994	1995	1996	1997	1998	1999	2000	2001	2002	2003	2004	2005	2006	2007	2008	2009	2010	2011	2012	2013	2014	2015	2016
Global	0.526	0.531	0.535	0.54	0.544	0.549	0.554	0.559	0.564	0.569	0.575	0.58	0.586	0.591	0.596	0.6	0.605	0.609	0.614	0.618	0.622	0.626	0.63	0.634	0.639	0.643	0.648	0.652	0.657	0.662	0.666	0.671	0.676	0.681	0.686	0.691	0.696
Southeast Asia, East Asia, and Oceania	0.438	0.445	0.453	0.461	0.47	0.478	0.486	0.495	0.504	0.512	0.521	0.53	0.539	0.548	0.556	0.565	0.573	0.581	0.587	0.594	0.6	0.606	0.612	0.619	0.626	0.633	0.641	0.65	0.658	0.666	0.675	0.683	0.691	0.699	0.706	0.712	0.719
East Asia	0.436	0.443	0.45	0.458	0.467	0.477	0.486	0.496	0.506	0.515	0.524	0.533	0.543	0.552	0.562	0.571	0.58	0.588	0.594	0.601	0.608	0.614	0.621	0.629	0.636	0.644	0.653	0.661	0.67	0.678	0.686	0.694	0.701	0.709	0.715	0.722	0.729
China	0.42	0.428	0.435	0.444	0.453	0.463	0.473	0.483	0.494	0.503	0.512	0.521	0.531	0.542	0.552	0.562	0.57	0.578	0.585	0.592	0.6	0.607	0.614	0.622	0.63	0.638	0.647	0.657	0.665	0.674	0.682	0.691	0.698	0.706	0.713	0.72	0.727
North Korea	0.471	0.476	0.48	0.485	0.488	0.491	0.494	0.497	0.499	0.502	0.505	0.508	0.51	0.511	0.51	0.506	0.501	0.495	0.489	0.483	0.478	0.473	0.47	0.468	0.468	0.469	0.47	0.471	0.473	0.474	0.476	0.478	0.481	0.484	0.488	0.491	0.494
Taiwan (Province of China)	0.636	0.645	0.657	0.669	0.681	0.695	0.706	0.71	0.718	0.727	0.734	0.743	0.749	0.756	0.762	0.768	0.775	0.788	0.798	0.8	0.808	0.815	0.82	0.824	0.829	0.833	0.837	0.842	0.845	0.849	0.853	0.857	0.86	0.864	0.868	0.871	0.875
Hong Kong Special Administrative Region of China	0.633	0.643	0.652	0.662	0.671	0.677	0.682	0.688	0.694	0.699	0.704	0.708	0.713	0.718	0.723	0.727	0.731	0.734	0.737	0.74	0.743	0.746	0.749	0.752	0.756	0.761	0.765	0.77	0.775	0.779	0.783	0.787	0.791	0.795	0.798	0.801	0.805
Macao Special Administrative Region of China	0.638	0.646	0.654	0.66	0.667	0.672	0.678	0.684	0.689	0.695	0.7	0.705	0.711	0.716	0.721	0.725	0.728	0.731	0.732	0.734	0.735	0.738	0.742	0.747	0.754	0.761	0.768	0.776	0.784	0.789	0.793	0.796	0.799	0.802	0.806	0.809	0.811
China (without Hong Kong and Macao)	0.413	0.42	0.428	0.436	0.446	0.456	0.467	0.477	0.487	0.497	0.506	0.515	0.525	0.536	0.546	0.556	0.565	0.574	0.581	0.588	0.596	0.603	0.611	0.619	0.627	0.635	0.645	0.654	0.663	0.672	0.68	0.689	0.697	0.704	0.712	0.718	0.725
Anhui	0.353	0.36	0.367	0.375	0.385	0.396	0.406	0.417	0.427	0.437	0.446	0.455	0.464	0.474	0.483	0.493	0.502	0.51	0.518	0.525	0.532	0.538	0.545	0.552	0.559	0.567	0.576	0.585	0.594	0.603	0.611	0.619	0.626	0.633	0.639	0.645	0.651
Beijing	0.606	0.612	0.618	0.624	0.631	0.638	0.645	0.653	0.659	0.665	0.671	0.676	0.682	0.689	0.695	0.702	0.709	0.715	0.721	0.726	0.732	0.738	0.744	0.75	0.757	0.764	0.772	0.78	0.787	0.795	0.802	0.809	0.816	0.823	0.829	0.835	0.842
Chongqing	0.367	0.375	0.384	0.394	0.407	0.419	0.431	0.443	0.454	0.464	0.474	0.485	0.496	0.507	0.519	0.53	0.539	0.549	0.557	0.565	0.574	0.582	0.591	0.599	0.607	0.616	0.625	0.635	0.643	0.652	0.66	0.668	0.675	0.682	0.689	0.694	0.7
Fujian	0.372	0.378	0.386	0.396	0.408	0.42	0.433	0.445	0.458	0.469	0.48	0.492	0.504	0.518	0.531	0.544	0.555	0.564	0.573	0.581	0.59	0.598	0.606	0.615	0.623	0.632	0.642	0.652	0.662	0.671	0.68	0.689	0.698	0.706	0.713	0.72	0.727
Gansu	0.353	0.358	0.364	0.372	0.382	0.391	0.401	0.411	0.421	0.431	0.438	0.447	0.456	0.465	0.473	0.482	0.49	0.498	0.506	0.513	0.52	0.526	0.533	0.539	0.546	0.553	0.56	0.568	0.575	0.583	0.59	0.597	0.604	0.61	0.616	0.622	0.628
Guangdong	0.444	0.451	0.458	0.467	0.476	0.487	0.497	0.507	0.518	0.527	0.536	0.546	0.557	0.567	0.578	0.588	0.598	0.607	0.615	0.623	0.632	0.64	0.648	0.658	0.667	0.678	0.688	0.7	0.71	0.72	0.729	0.737	0.745	0.753	0.761	0.768	0.775
Guangxi	0.361	0.369	0.376	0.385	0.395	0.406	0.417	0.428	0.439	0.449	0.458	0.468	0.479	0.49	0.501	0.511	0.521	0.531	0.539	0.547	0.555	0.562	0.569	0.577	0.585	0.594	0.603	0.613	0.622	0.632	0.641	0.651	0.661	0.67	0.679	0.686	0.693
Guizhou	0.279	0.287	0.295	0.303	0.314	0.325	0.336	0.347	0.358	0.368	0.376	0.385	0.394	0.403	0.413	0.422	0.431	0.439	0.446	0.454	0.462	0.469	0.477	0.485	0.494	0.503	0.512	0.522	0.531	0.54	0.55	0.559	0.567	0.575	0.581	0.587	0.594
Hainan	0.438	0.443	0.448	0.454	0.462	0.47	0.478	0.486	0.495	0.502	0.511	0.52	0.529	0.539	0.549	0.558	0.566	0.575	0.582	0.59	0.597	0.604	0.611	0.618	0.625	0.632	0.64	0.648	0.656	0.665	0.672	0.68	0.686	0.693	0.699	0.705	0.711
Hebei	0.42	0.427	0.435	0.443	0.452	0.462	0.472	0.482	0.492	0.501	0.509	0.517	0.527	0.537	0.547	0.556	0.565	0.573	0.581	0.588	0.596	0.603	0.611	0.619	0.627	0.635	0.644	0.654	0.662	0.671	0.679	0.687	0.695	0.702	0.709	0.716	0.722
Heilongjiang	0.477	0.482	0.488	0.495	0.504	0.513	0.522	0.531	0.54	0.548	0.557	0.566	0.575	0.582	0.588	0.594	0.6	0.606	0.611	0.617	0.623	0.628	0.634	0.639	0.644	0.649	0.655	0.662	0.668	0.674	0.679	0.685	0.69	0.695	0.7	0.704	0.709
Henan	0.368	0.375	0.384	0.394	0.405	0.417	0.429	0.441	0.453	0.463	0.471	0.481	0.491	0.502	0.513	0.525	0.535	0.545	0.554	0.562	0.57	0.578	0.587	0.595	0.604	0.613	0.623	0.633	0.643	0.652	0.661	0.67	0.678	0.685	0.693	0.699	0.706
Hubei	0.407	0.415	0.423	0.432	0.442	0.452	0.463	0.474	0.484	0.494	0.504	0.514	0.524	0.535	0.545	0.553	0.561	0.569	0.576	0.583	0.59	0.597	0.604	0.611	0.618	0.626	0.635	0.643	0.651	0.659	0.667	0.674	0.681	0.688	0.694	0.7	0.706
Hunan	0.383	0.391	0.399	0.409	0.42	0.431	0.442	0.454	0.465	0.476	0.485	0.495	0.505	0.516	0.526	0.536	0.546	0.555	0.562	0.569	0.576	0.582	0.59	0.597	0.605	0.613	0.621	0.63	0.639	0.647	0.655	0.663	0.67	0.678	0.684	0.691	0.697
Inner Mongolia	0.407	0.413	0.422	0.431	0.444	0.456	0.468	0.48	0.492	0.502	0.512	0.522	0.534	0.545	0.556	0.566	0.575	0.584	0.593	0.602	0.61	0.618	0.627	0.634	0.641	0.648	0.656	0.664	0.672	0.68	0.688	0.696	0.703	0.71	0.716	0.722	0.728
Jiangsu	0.443	0.451	0.459	0.468	0.479	0.489	0.5	0.51	0.521	0.53	0.539	0.548	0.557	0.565	0.573	0.582	0.591	0.599	0.607	0.614	0.621	0.629	0.637	0.646	0.655	0.664	0.674	0.684	0.693	0.702	0.711	0.72	0.728	0.736	0.743	0.75	0.757
Jiangxi	0.347	0.354	0.362	0.371	0.382	0.394	0.405	0.416	0.428	0.439	0.449	0.458	0.469	0.48	0.49	0.5	0.509	0.518	0.526	0.534	0.542	0.549	0.557	0.565	0.573	0.581	0.59	0.599	0.607	0.615	0.622	0.629	0.636	0.643	0.649	0.655	0.662
Jilin	0.451	0.457	0.464	0.471	0.48	0.49	0.5	0.509	0.519	0.528	0.537	0.547	0.557	0.564	0.571	0.579	0.586	0.594	0.6	0.607	0.613	0.62	0.626	0.633	0.639	0.646	0.653	0.661	0.668	0.676	0.683	0.689	0.696	0.702	0.708	0.713	0.719
Liaoning	0.521	0.527	0.533	0.539	0.547	0.555	0.563	0.571	0.579	0.587	0.592	0.597	0.602	0.608	0.614	0.621	0.627	0.634	0.639	0.645	0.651	0.656	0.662	0.668	0.674	0.68	0.686	0.693	0.7	0.707	0.713	0.719	0.725	0.73	0.736	0.741	0.746
Ningxia	0.37	0.375	0.382	0.39	0.398	0.408	0.418	0.429	0.44	0.45	0.46	0.469	0.479	0.489	0.498	0.507	0.515	0.523	0.531	0.538	0.545	0.552	0.559	0.566	0.574	0.583	0.592	0.601	0.61	0.617	0.624	0.632	0.639	0.645	0.652	0.658	0.664
Qinghai	0.354	0.36	0.366	0.375	0.386	0.397	0.408	0.42	0.431	0.442	0.452	0.462	0.473	0.482	0.492	0.501	0.509	0.517	0.524	0.53	0.537	0.544	0.55	0.557	0.564	0.571	0.578	0.586	0.593	0.6	0.607	0.614	0.62	0.627	0.633	0.639	0.645
Shaanxi	0.385	0.392	0.4	0.409	0.419	0.43	0.441	0.451	0.462	0.471	0.48	0.49	0.501	0.512	0.522	0.532	0.54	0.549	0.556	0.564	0.571	0.579	0.587	0.594	0.602	0.61	0.619	0.628	0.637	0.645	0.654	0.662	0.67	0.677	0.684	0.69	0.696
Shandong	0.413	0.421	0.431	0.44	0.451	0.463	0.474	0.485	0.497	0.507	0.516	0.525	0.535	0.544	0.553	0.563	0.572	0.581	0.589	0.596	0.604	0.612	0.62	0.629	0.638	0.647	0.657	0.668	0.678	0.687	0.696	0.705	0.713	0.721	0.729	0.736	0.742
Shanghai	0.638	0.643	0.648	0.652	0.656	0.661	0.666	0.67	0.675	0.679	0.684	0.689	0.694	0.699	0.704	0.709	0.714	0.719	0.723	0.727	0.732	0.736	0.741	0.747	0.753	0.759	0.765	0.772	0.778	0.785	0.791	0.797	0.803	0.809	0.815	0.82	0.826
Shanxi	0.42	0.426	0.434	0.442	0.452	0.462	0.472	0.482	0.493	0.502	0.511	0.52	0.53	0.54	0.55	0.559	0.568	0.577	0.585	0.593	0.6	0.607	0.614	0.621	0.628	0.635	0.643	0.651	0.659	0.666	0.674	0.681	0.688	0.694	0.7	0.706	0.712
Sichuan	0.349	0.359	0.369	0.38	0.392	0.405	0.418	0.43	0.443	0.454	0.463	0.472	0.482	0.492	0.502	0.511	0.52	0.528	0.535	0.542	0.549	0.556	0.563	0.571	0.58	0.589	0.598	0.608	0.617	0.625	0.634	0.642	0.65	0.657	0.664	0.67	0.677
Tianjin	0.569	0.575	0.582	0.589	0.596	0.604	0.611	0.618	0.625	0.63	0.636	0.641	0.647	0.653	0.66	0.666	0.673	0.679	0.685	0.69	0.696	0.702	0.709	0.715	0.722	0.729	0.737	0.745	0.753	0.76	0.768	0.775	0.782	0.789	0.795	0.801	0.807
Tibet	0.344	0.348	0.352	0.357	0.363	0.369	0.376	0.383	0.39	0.398	0.406	0.414	0.422	0.429	0.437	0.444	0.45	0.456	0.462	0.468	0.474	0.48	0.487	0.495	0.503	0.512	0.519	0.526	0.532	0.538	0.544	0.55	0.556	0.561	0.566	0.571	0.577
Xinjiang	0.377	0.383	0.39	0.399	0.41	0.423	0.435	0.448	0.462	0.474	0.486	0.499	0.512	0.524	0.536	0.548	0.558	0.569	0.578	0.588	0.597	0.606	0.614	0.622	0.629	0.636	0.644	0.652	0.66	0.668	0.676	0.683	0.69	0.696	0.702	0.707	0.713
Yunnan	0.311	0.319	0.327	0.336	0.346	0.358	0.369	0.38	0.391	0.402	0.412	0.422	0.432	0.443	0.453	0.464	0.473	0.482	0.491	0.499	0.507	0.515	0.523	0.532	0.541	0.55	0.56	0.57	0.579	0.587	0.596	0.604	0.611	0.618	0.625	0.632	0.639
Zhejiang	0.418	0.426	0.435	0.445	0.457	0.469	0.481	0.493	0.505	0.515	0.524	0.534	0.544	0.552	0.561	0.57	0.579	0.588	0.596	0.604	0.612	0.62	0.629	0.637	0.647	0.656	0.666	0.677	0.686	0.695	0.705	0.714	0.722	0.73	0.738	0.745	0.753
Southeast Asia	0.411	0.421	0.431	0.441	0.45	0.459	0.467	0.475	0.483	0.491	0.499	0.507	0.515	0.523	0.531	0.539	0.547	0.554	0.56	0.566	0.571	0.577	0.582	0.587	0.593	0.598	0.604	0.61	0.616	0.623	0.629	0.636	0.644	0.651	0.659	0.666	0.672
Cambodia	0.258	0.261	0.264	0.267	0.271	0.275	0.279	0.284	0.29	0.296	0.301	0.306	0.312	0.316	0.321	0.326	0.331	0.336	0.342	0.349	0.357	0.365	0.373	0.382	0.391	0.401	0.412	0.423	0.433	0.443	0.452	0.461	0.47	0.479	0.487	0.496	0.503
Indonesia	0.403	0.414	0.425	0.436	0.446	0.457	0.467	0.476	0.485	0.493	0.501	0.51	0.518	0.526	0.534	0.541	0.549	0.557	0.562	0.567	0.572	0.577	0.582	0.586	0.591	0.596	0.601	0.607	0.613	0.62	0.627	0.635	0.643	0.652	0.66	0.669	0.676
Laos	0.239	0.245	0.252	0.259	0.265	0.272	0.279	0.286	0.291	0.297	0.303	0.309	0.315	0.321	0.327	0.335	0.344	0.354	0.363	0.372	0.382	0.391	0.4	0.409	0.418	0.426	0.434	0.441	0.448	0.456	0.463	0.471	0.48	0.488	0.496	0.505	0.512
Malaysia	0.495	0.502	0.51	0.515	0.518	0.523	0.531	0.54	0.549	0.557	0.564	0.569	0.575	0.583	0.593	0.603	0.613	0.625	0.632	0.637	0.648	0.661	0.672	0.679	0.687	0.694	0.701	0.707	0.712	0.718	0.726	0.734	0.742	0.75	0.758	0.766	0.773
Maldives	0.344	0.353	0.362	0.371	0.379	0.389	0.397	0.406	0.415	0.424	0.433	0.442	0.451	0.46	0.469	0.479	0.489	0.5	0.511	0.522	0.534	0.545	0.555	0.565	0.576	0.583	0.592	0.602	0.611	0.621	0.63	0.641	0.651	0.661	0.67	0.68	0.687
Myanmar	0.295	0.301	0.308	0.315	0.321	0.328	0.334	0.339	0.342	0.346	0.349	0.352	0.356	0.36	0.366	0.372	0.379	0.386	0.393	0.401	0.411	0.42	0.43	0.441	0.453	0.465	0.478	0.49	0.502	0.513	0.524	0.536	0.547	0.558	0.569	0.58	0.59
Philippines	0.457	0.465	0.474	0.482	0.489	0.495	0.5	0.505	0.51	0.515	0.52	0.525	0.529	0.534	0.539	0.544	0.549	0.554	0.558	0.562	0.566	0.57	0.573	0.577	0.581	0.585	0.589	0.594	0.598	0.603	0.608	0.613	0.619	0.625	0.631	0.637	0.642
Sri Lanka	0.43	0.437	0.445	0.453	0.463	0.473	0.484	0.492	0.5	0.506	0.512	0.519	0.526	0.533	0.541	0.55	0.559	0.568	0.576	0.581	0.584	0.588	0.592	0.598	0.604	0.609	0.615	0.621	0.628	0.636	0.646	0.656	0.666	0.676	0.685	0.695	0.703
Thailand	0.435	0.449	0.462	0.475	0.486	0.497	0.507	0.517	0.526	0.535	0.544	0.554	0.563	0.572	0.581	0.591	0.6	0.608	0.615	0.621	0.628	0.635	0.642	0.649	0.657	0.664	0.672	0.68	0.686	0.691	0.697	0.703	0.709	0.714	0.719	0.724	0.729
Timor-Leste	0.236	0.245	0.253	0.261	0.269	0.278	0.286	0.295	0.304	0.314	0.323	0.332	0.342	0.351	0.36	0.368	0.376	0.383	0.389	0.39	0.392	0.394	0.396	0.395	0.404	0.416	0.429	0.437	0.444	0.452	0.462	0.475	0.488	0.502	0.514	0.525	0.532
Vietnam	0.354	0.362	0.37	0.378	0.387	0.395	0.403	0.411	0.421	0.43	0.439	0.448	0.458	0.467	0.476	0.486	0.495	0.505	0.514	0.522	0.53	0.538	0.546	0.552	0.559	0.566	0.573	0.579	0.586	0.592	0.599	0.606	0.613	0.619	0.626	0.633	0.639
Mauritius	0.532	0.546	0.559	0.569	0.578	0.587	0.59	0.591	0.595	0.602	0.61	0.616	0.621	0.63	0.639	0.647	0.654	0.662	0.667	0.671	0.678	0.684	0.688	0.693	0.698	0.703	0.71	0.715	0.722	0.726	0.73	0.735	0.739	0.743	0.747	0.752	0.756
Seychelles	0.492	0.51	0.524	0.531	0.538	0.548	0.56	0.574	0.586	0.595	0.603	0.61	0.618	0.625	0.634	0.644	0.654	0.663	0.671	0.677	0.682	0.687	0.691	0.693	0.694	0.696	0.697	0.698	0.7	0.703	0.706	0.708	0.711	0.716	0.723	0.73	0.736
Aceh	0.418	0.43	0.443	0.456	0.469	0.482	0.494	0.505	0.516	0.527	0.537	0.547	0.557	0.566	0.575	0.583	0.591	0.599	0.604	0.609	0.612	0.614	0.616	0.618	0.62	0.622	0.625	0.629	0.633	0.637	0.642	0.647	0.653	0.66	0.667	0.674	0.681
North Sumatera	0.407	0.418	0.429	0.439	0.45	0.46	0.47	0.478	0.485	0.492	0.499	0.506	0.513	0.52	0.527	0.533	0.54	0.547	0.552	0.558	0.562	0.567	0.572	0.578	0.583	0.588	0.593	0.6	0.607	0.616	0.625	0.635	0.645	0.654	0.663	0.672	0.68

Appendix Table 7: Socio-demographic Index values for all estimated GBD 2016 locations, 1980–2016

Location	1980	1981	1982	1983	1984	1985	1986	1987	1988	1989	1990	1991	1992	1993	1994	1995	1996	1997	1998	1999	2000	2001	2002	2003	2004	2005	2006	2007	2008	2009	2010	2011	2012	2013	2014	2015	2016
West Sumatera	0.387	0.398	0.408	0.418	0.429	0.439	0.448	0.458	0.466	0.475	0.483	0.491	0.498	0.506	0.513	0.519	0.526	0.532	0.536	0.541	0.546	0.551	0.557	0.563	0.568	0.574	0.58	0.587	0.594	0.602	0.611	0.62	0.629	0.638	0.647	0.656	0.663
Riau	0.438	0.451	0.465	0.479	0.494	0.507	0.521	0.533	0.545	0.556	0.567	0.577	0.586	0.595	0.602	0.609	0.616	0.622	0.628	0.632	0.639	0.645	0.652	0.656	0.661	0.665	0.67	0.675	0.682	0.688	0.695	0.702	0.711	0.72	0.728	0.737	0.744
Jambi	0.355	0.366	0.376	0.387	0.398	0.408	0.418	0.428	0.438	0.447	0.456	0.465	0.474	0.483	0.492	0.501	0.51	0.519	0.526	0.532	0.537	0.542	0.549	0.555	0.562	0.568	0.575	0.583	0.592	0.6	0.61	0.62	0.63	0.64	0.649	0.659	0.667
South Sumatera	0.416	0.426	0.436	0.446	0.456	0.466	0.476	0.485	0.495	0.503	0.512	0.521	0.53	0.537	0.544	0.55	0.557	0.563	0.569	0.572	0.575	0.578	0.581	0.584	0.587	0.592	0.596	0.602	0.608	0.615	0.622	0.629	0.638	0.646	0.655	0.663	0.671
Bengkulu	0.348	0.359	0.369	0.38	0.391	0.402	0.413	0.423	0.433	0.443	0.452	0.462	0.471	0.48	0.489	0.498	0.507	0.515	0.521	0.526	0.53	0.534	0.538	0.543	0.548	0.554	0.561	0.567	0.575	0.582	0.589	0.598	0.607	0.616	0.625	0.634	0.641
Lampung	0.344	0.355	0.365	0.376	0.386	0.396	0.405	0.414	0.421	0.429	0.437	0.444	0.453	0.46	0.469	0.478	0.489	0.498	0.507	0.516	0.522	0.527	0.532	0.536	0.54	0.544	0.549	0.555	0.562	0.571	0.581	0.592	0.603	0.613	0.623	0.633	0.641
Bangka Belitung	0.398	0.408	0.417	0.427	0.437	0.447	0.456	0.466	0.475	0.483	0.492	0.499	0.507	0.514	0.521	0.528	0.535	0.542	0.548	0.553	0.557	0.562	0.567	0.572	0.578	0.584	0.589	0.595	0.602	0.608	0.615	0.623	0.631	0.639	0.647	0.656	0.663
Riau Islands	0.439	0.453	0.467	0.482	0.498	0.513	0.528	0.542	0.555	0.568	0.581	0.593	0.606	0.617	0.626	0.635	0.644	0.653	0.659	0.665	0.669	0.672	0.675	0.678	0.681	0.684	0.688	0.692	0.698	0.704	0.711	0.718	0.726	0.734	0.742	0.751	0.758
North Kalimantan	0.455	0.469	0.483	0.498	0.513	0.527	0.541	0.554	0.566	0.577	0.588	0.599	0.61	0.62	0.63	0.64	0.649	0.658	0.666	0.673	0.679	0.684	0.689	0.693	0.698	0.704	0.709	0.713	0.72	0.726	0.731	0.739	0.746	0.754	0.761	0.769	0.775
Jakarta	0.554	0.566	0.576	0.587	0.599	0.609	0.619	0.629	0.638	0.646	0.654	0.663	0.67	0.68	0.689	0.697	0.706	0.714	0.72	0.725	0.73	0.735	0.74	0.745	0.75	0.754	0.759	0.764	0.769	0.775	0.782	0.789	0.795	0.803	0.81	0.818	0.824
Cental Java	0.406	0.416	0.426	0.436	0.446	0.456	0.465	0.474	0.481	0.488	0.496	0.504	0.512	0.52	0.529	0.538	0.546	0.554	0.56	0.563	0.566	0.569	0.571	0.575	0.578	0.582	0.587	0.593	0.6	0.608	0.615	0.623	0.631	0.639	0.648	0.656	0.663
West Java	0.363	0.374	0.384	0.395	0.405	0.415	0.425	0.433	0.442	0.449	0.457	0.466	0.476	0.483	0.492	0.5	0.508	0.516	0.521	0.526	0.529	0.533	0.536	0.54	0.545	0.549	0.555	0.561	0.568	0.575	0.582	0.591	0.599	0.608	0.616	0.625	0.632
Yogyakarta	0.393	0.405	0.417	0.429	0.44	0.451	0.462	0.472	0.481	0.49	0.499	0.508	0.517	0.53	0.543	0.554	0.563	0.572	0.576	0.58	0.584	0.587	0.591	0.595	0.599	0.603	0.607	0.612	0.618	0.624	0.631	0.639	0.647	0.655	0.663	0.672	0.679
East Java	0.384	0.395	0.406	0.416	0.427	0.437	0.447	0.456	0.465	0.473	0.482	0.49	0.498	0.506	0.514	0.522	0.53	0.538	0.545	0.55	0.556	0.562	0.567	0.573	0.579	0.584	0.59	0.596	0.603	0.61	0.617	0.625	0.633	0.642	0.651	0.659	0.667
Banten	0.398	0.408	0.418	0.428	0.439	0.449	0.459	0.469	0.478	0.486	0.495	0.504	0.513	0.521	0.529	0.536	0.544	0.553	0.561	0.568	0.573	0.577	0.581	0.586	0.59	0.593	0.598	0.602	0.607	0.612	0.618	0.626	0.635	0.644	0.652	0.661	0.669
Bali	0.394	0.406	0.418	0.43	0.442	0.454	0.466	0.477	0.486	0.496	0.506	0.515	0.524	0.534	0.544	0.552	0.561	0.57	0.575	0.579	0.582	0.585	0.589	0.592	0.596	0.6	0.604	0.609	0.614	0.621	0.628	0.636	0.644	0.653	0.662	0.67	0.678
Nusa Tenggara Barat	0.285	0.297	0.307	0.318	0.328	0.338	0.346	0.354	0.361	0.367	0.374	0.383	0.392	0.403	0.413	0.424	0.434	0.444	0.452	0.458	0.466	0.475	0.483	0.49	0.498	0.505	0.511	0.518	0.524	0.532	0.539	0.547	0.554	0.562	0.569	0.577	0.584
East Nusa Tenggara	0.301	0.311	0.319	0.327	0.335	0.343	0.351	0.359	0.366	0.373	0.381	0.388	0.395	0.402	0.409	0.415	0.42	0.426	0.427	0.427	0.429	0.432	0.436	0.439	0.443	0.447	0.451	0.457	0.463	0.47	0.479	0.488	0.497	0.507	0.516	0.525	0.534
West Kalimantan Central Kalimantan	0.319	0.33	0.341	0.353	0.365	0.377	0.388	0.398	0.408	0.419	0.429	0.439	0.45	0.461	0.472	0.483	0.494	0.505	0.513	0.52	0.525	0.53	0.534	0.538	0.542	0.546	0.55	0.555	0.559	0.565	0.571	0.578	0.586	0.594	0.602	0.61	0.618
South Kalimantan	0.41	0.42	0.429	0.439	0.449	0.459	0.469	0.479	0.488	0.497	0.506	0.515	0.523	0.534	0.545	0.554	0.564	0.573	0.579	0.583	0.585	0.586	0.587	0.588	0.59	0.591	0.594	0.598	0.603	0.61	0.617	0.625	0.633	0.642	0.65	0.659	0.667
East Kalimantan	0.399	0.41	0.421	0.432	0.442	0.453	0.463	0.472	0.48	0.487	0.494	0.502	0.51	0.519	0.527	0.534	0.542	0.549	0.554	0.558	0.561	0.564	0.567	0.57	0.574	0.577	0.58	0.584	0.589	0.594	0.601	0.609	0.617	0.625	0.633	0.642	0.649
North Sulawesi	0.463	0.478	0.492	0.507	0.522	0.536	0.55	0.563	0.575	0.587	0.598	0.607	0.617	0.625	0.632	0.64	0.647	0.654	0.661	0.667	0.673	0.68	0.686	0.691	0.696	0.702	0.707	0.711	0.717	0.722	0.726	0.733	0.739	0.747	0.755	0.763	0.77
Central Sulawesi	0.44	0.45	0.46	0.469	0.478	0.487	0.495	0.503	0.51	0.517	0.524	0.532	0.539	0.548	0.556	0.564	0.573	0.581	0.588	0.591	0.593	0.595	0.596	0.597	0.599	0.601	0.603	0.607	0.612	0.619	0.626	0.634	0.642	0.651	0.659	0.668	0.675
South Sumatera	0.363	0.373	0.383	0.392	0.402	0.411	0.42	0.429	0.437	0.444	0.452	0.46	0.468	0.476	0.485	0.493	0.502	0.509	0.516	0.523	0.527	0.533	0.538	0.542	0.547	0.551	0.555	0.559	0.565	0.572	0.579	0.588	0.597	0.606	0.616	0.625	0.633
Southeast Sulawesi	0.354	0.366	0.377	0.388	0.399	0.409	0.42	0.429	0.438	0.447	0.456	0.465	0.474	0.482	0.489	0.496	0.504	0.51	0.516	0.52	0.525	0.53	0.535	0.54	0.546	0.55	0.554	0.56	0.566	0.574	0.583	0.593	0.603	0.613	0.623	0.632	0.64
Gorontalo	0.327	0.339	0.35	0.361	0.373	0.384	0.395	0.405	0.414	0.423	0.432	0.442	0.45	0.458	0.465	0.473	0.48	0.487	0.494	0.499	0.503	0.508	0.513	0.519	0.525	0.531	0.537	0.544	0.552	0.561	0.57	0.58	0.589	0.599	0.608	0.617	0.625
West Sulawesi	0.328	0.338	0.346	0.354	0.362	0.37	0.378	0.385	0.392	0.399	0.407	0.415	0.424	0.43	0.437	0.444	0.45	0.457	0.462	0.466	0.467	0.47	0.473	0.478	0.482	0.488	0.493	0.5	0.507	0.517	0.526	0.536	0.546	0.555	0.564	0.573	0.581
Maluku	0.318	0.329	0.338	0.348	0.358	0.368	0.378	0.387	0.396	0.405	0.414	0.422	0.43	0.436	0.442	0.447	0.453	0.458	0.462	0.466	0.469	0.473	0.477	0.481	0.485	0.489	0.493	0.498	0.507	0.516	0.525	0.536	0.547	0.558	0.568	0.579	0.587
North Maluku	0.446	0.453	0.46	0.468	0.476	0.483	0.491	0.499	0.506	0.512	0.519	0.526	0.533	0.539	0.545	0.551	0.557	0.562	0.566	0.567	0.564	0.559	0.554	0.548	0.542	0.536	0.531	0.527	0.527	0.529	0.534	0.541	0.551	0.561	0.571	0.581	0.59
West Papua	0.364	0.374	0.383	0.393	0.404	0.414	0.423	0.432	0.441	0.449	0.458	0.467	0.476	0.483	0.491	0.498	0.506	0.514	0.518	0.523	0.524	0.524	0.523	0.521	0.518	0.515	0.511	0.508	0.507	0.51	0.515	0.522	0.531	0.541	0.552	0.562	0.571
Papua	0.413	0.425	0.436	0.448	0.461	0.472	0.484	0.495	0.505	0.515	0.525	0.534	0.543	0.55	0.558	0.565	0.572	0.578	0.582	0.585	0.587	0.589	0.59	0.592	0.594	0.596	0.598	0.601	0.607	0.615	0.626	0.643	0.657	0.671	0.683	0.695	0.706
Oceania	0.442	0.45	0.457	0.465	0.474	0.481	0.488	0.494	0.5	0.505	0.51	0.516	0.522	0.529	0.534	0.54	0.546	0.552	0.558	0.562	0.565	0.569	0.571	0.573	0.574	0.577	0.58	0.583	0.584	0.585	0.588	0.589	0.591	0.595	0.6	0.605	0.61
Fiji	0.332	0.338	0.343	0.348	0.353	0.358	0.363	0.368	0.373	0.378	0.383	0.388	0.393	0.398	0.403	0.407	0.412	0.416	0.42	0.424	0.428	0.433	0.436	0.44	0.443	0.447	0.45	0.454	0.457	0.461	0.464	0.468	0.472	0.476	0.481	0.485	0.489
Kiribati	0.525	0.534	0.542	0.55	0.558	0.565	0.572	0.579	0.585	0.592	0.6	0.607	0.614	0.62	0.627	0.634	0.64	0.646	0.652	0.658	0.663	0.669	0.674	0.679	0.684	0.689	0.694	0.699	0.704	0.708	0.712	0.716	0.721	0.725	0.729	0.733	0.736
Marshall Islands	0.261	0.267	0.273	0.279	0.284	0.288	0.292	0.295	0.299	0.303	0.307	0.312	0.316	0.32	0.324	0.328	0.333	0.338	0.345	0.35	0.357	0.363	0.37	0.376	0.383	0.389	0.395	0.401	0.406	0.412	0.416	0.421	0.426	0.431	0.437	0.442	0.446
Federated States of Micronesia	0.399	0.406	0.413	0.42	0.427	0.432	0.439	0.446	0.453	0.46	0.466	0.47	0.473	0.474	0.475	0.476	0.477	0.479	0.482	0.487	0.492	0.499	0.504	0.51	0.514	0.518	0.522	0.527	0.531	0.536	0.541	0.547	0.553	0.559	0.565	0.571	0.575
Papua New Guinea	0.274	0.292	0.31	0.328	0.346	0.363	0.379	0.394	0.409	0.422	0.434	0.446	0.456	0.466	0.475	0.485	0.495	0.503	0.512	0.521	0.53	0.539	0.547	0.555	0.561	0.568	0.573	0.578	0.582	0.586	0.59	0.595	0.599	0.603	0.607	0.61	0.612
Samoa	0.256	0.26	0.263	0.267	0.271	0.274	0.278	0.283	0.287	0.292	0.296	0.3	0.304	0.31	0.315	0.32	0.325	0.33	0.334	0.338	0.342	0.345	0.348	0.352	0.355	0.359	0.362	0.367	0.371	0.376	0.382	0.387	0.394	0.4	0.406	0.413	0.418
Solomon Islands	0.372	0.381	0.389	0.397	0.405	0.412	0.42	0.428	0.435	0.443	0.451	0.458	0.465	0.472	0.477	0.482	0.487	0.492	0.498	0.501	0.504	0.507	0.511	0.514	0.518	0.521	0.525	0.529	0.533	0.537	0.54	0.542	0.545	0.548	0.553	0.558	0.562
Tonga	0.269	0.275	0.28	0.284	0.289	0.294	0.298	0.303	0.308	0.313	0.318	0.323	0.329	0.335	0.34	0.346	0.352	0.357	0.363	0.368	0.371	0.373	0.374	0.375	0.377	0.379	0.383	0.387	0.393	0.398	0.404	0.411	0.418	0.425	0.432	0.439	0.444
Vanuatu	0.382	0.389	0.397	0.403	0.412	0.419	0.427	0.435	0.442	0.448	0.454	0.461	0.466	0.471	0.476	0.481	0.486	0.491	0.496	0.501	0.506	0.511	0.516	0.522	0.527	0.532	0.537	0.541	0.546	0.551	0.555	0.561	0.566	0.571	0.576	0.582	0.586
American Samoa	0.277	0.283	0.288	0.294	0.3	0.306	0.312	0.318	0.324	0.329	0.335	0.341	0.347	0.353	0.359	0.364	0.37	0.376	0.382	0.387	0.393	0.399	0.405	0.41	0.415	0.42	0.426	0.431	0.437	0.443	0.449	0.454	0.46	0.465	0.471	0.476	0.479
Guam	0.551	0.561	0.569	0.576	0.583	0.589	0.595	0.601	0.606	0.612	0.617	0.622	0.626	0.631	0.635	0.639	0.643	0.648	0.652	0.656	0.661	0.666	0.671	0.676	0.682	0.688	0.695	0.7	0.705	0.711	0.716	0.721	0.726	0.73	0.735	0.739	0.742
Northern Mariana Islands	0.718	0.726	0.733	0.738	0.742	0.743	0.743	0.743	0.746	0.75	0.755	0.761	0.765	0.77	0.774	0.778	0.782	0.785	0.788	0.791	0.794	0.797	0.799	0.801	0.802	0.804	0.806	0.807	0.809	0.81	0.812	0.813	0.815	0.817	0.819	0.821	0.822
	0.61	0.622	0.633	0.643	0.653	0.662	0.671	0.68	0.689	0.697	0.705	0.713	0.72	0.727	0.733	0.739	0.744	0.75	0.755	0.761	0.766	0.772	0.777	0.782	0.787	0.79	0.792	0.794	0.795	0.795	0.795	0.794	0.793	0.793	0.794	0.796	0.797
Central Europe, Eastern Europe, and Central Asia	0.706	0.708	0.71	0.711	0.714	0.716	0.717	0.721	0.727	0.733	0.739	0.746	0.751	0.754	0.755	0.757	0.758	0.758	0.758	0.758	0.759	0.762	0.765	0.769	0.773	0.778	0.782	0.786	0.79	0.793	0.797	0.801	0.804	0.806	0.809	0.812	0.816
Central Asia	0.576	0.582	0.587	0.591	0.594	0.598	0.602	0.607	0.612	0.618	0.624	0.63	0.635	0.638	0.641	0.642	0.643	0.643	0.643	0.643	0.645	0.647	0.65	0.654	0.66	0.666	0.672	0.679	0.685	0.691	0.697	0.703	0.708	0.714	0.719	0.724	0.729
Armenia	0.602	0.606	0.61	0.615	0.619	0.622	0.626	0.631	0.635	0.64	0.644	0.648	0.648	0.647	0.645	0.643	0.642	0.641	0.642	0.643	0.646	0.651	0.659	0.668	0.676	0.686	0.695	0.705	0.714	0.719	0.724	0.73	0.735	0.74	0.745	0.75	0.755
Azerbaijan	0.606	0.614	0.622	0.63	0.637	0.643	0.648	0.654	0.659	0.664	0.668	0.673	0.676	0.678	0.678	0.677	0.674	0.67	0.667	0.664	0.662	0.662	0.664	0.667	0.671	0.678	0.688	0.7	0.712	0.722	0.731	0.739	0.747	0.753	0.759	0.763	0.767
Georgia	0.679	0.684	0.688	0.691	0.695	0.698	0.702	0.706	0.71	0.715	0.72	0.723	0.722	0.718	0.713	0.705	0.698	0.689	0.682	0.677	0.674	0.676	0.679	0.684	0.69	0.695	0.701	0.708	0.714	0.719	0.723	0.727	0.731	0.736	0.741	0.746	0.751
Kazakhstan	0.629	0.636	0.641	0.645	0.649	0.653	0.658	0.663	0.669	0.675	0.681	0.687	0.692	0.696	0.699	0.701	0.703	0.704	0.704	0.704	0.705	0.706	0.708	0.71	0.712	0.715	0.718	0.721	0.725	0.728	0.731	0.736	0.74	0.744	0.748	0.753	0.757
Kyrgyzstan	0.556	0.562	0.567	0.571	0.575	0.578	0.582	0.587	0.592	0.598	0.604	0.61	0.614	0.618	0.619	0.618	0.617	0.615	0.612	0.609	0.607	0.606	0.605	0.605	0.606	0.606	0.606	0.607	0.608	0.609	0.61	0.612	0.614	0.618	0.622	0.627	0.632
Mongolia	0.403	0.417	0.431	0.445	0.458	0.473	0.489	0.506	0.523	0.539	0.557	0.573	0.586	0.597	0.604	0.611	0.615	0.619	0.623	0.627	0.631	0.635	0.639	0.643	0.647	0.65	0.653	0.656	0.659	0.66	0.662	0.664	0.667	0.67	0.674	0.678	0.683
Tajikistan	0.462	0.469	0.474	0.478	0.481	0.484	0.488	0.494	0.501	0.51	0.519	0.53	0.537	0.542	0.543	0.542	0.537	0.532	0.525	0.519	0.515	0.513	0.515	0.52	0.527	0.535	0.542	0.548	0.552	0.556	0.559	0.561	0.564	0.567	0.571	0.574	0.577
Turkmenistan	0.53	0.536	0.541	0.545	0.548	0.552	0.555	0.56	0.565	0.571	0.577	0.584	0.59	0.598	0.605	0.612	0.618	0.623	0.627	0.63	0.636	0.641	0.647	0.653	0.66	0.668	0.676	0.684	0.692	0.701	0.709	0.717	0.726	0.735	0.743	0.752	0.759
Uzbekistan	0.493	0.498	0.503	0.506	0.51	0.513	0.518	0.523	0.53	0.537	0.544	0.551	0.558	0.565	0.571	0.577	0.582	0.587	0.591	0.594	0.597	0.6	0.604	0.609	0.616	0.623	0.629	0.636	0.642	0.649	0.656	0.664	0.671	0.678	0.685	0.692	0.698
Central Europe	0.701	0.705	0.709	0.711	0.713	0.717	0.72	0.724	0.729	0.736	0.741	0.746	0.75	0.753	0.758	0.763	0.766	0.77	0.773	0.777	0.781	0.785	0.79	0.794	0.799	0.803	0.808	0.814	0.819	0.823	0.828	0.832	0.836	0.839	0.842	0.846	0.849

Appendix Table 7: Socio-demographic Index values for all estimated GBD 2016 locations, 1980–2016

Location	1980	1981	1982	1983	1984	1985	1986	1987	1988	1989	1990	1991	1992	1993	1994	1995	1996	1997	1998	1999	2000	2001	2002	2003	2004	2005	2006	2007	2008	2009	2010	2011	2012	2013	2014	2015	2016
Albania	0.54	0.545	0.55	0.555	0.56	0.564	0.569	0.573	0.577	0.581	0.584	0.585	0.584	0.585	0.588	0.593	0.601	0.607	0.614	0.623	0.633	0.642	0.651	0.659	0.667	0.674	0.68	0.686	0.692	0.697	0.702	0.706	0.711	0.714	0.718	0.721	0.725
Bosnia and Herzegovina	0.502	0.508	0.514	0.518	0.522	0.527	0.532	0.535	0.539	0.542	0.542	0.542	0.543	0.543	0.546	0.552	0.573	0.597	0.619	0.635	0.648	0.658	0.667	0.675	0.682	0.688	0.694	0.699	0.705	0.71	0.714	0.717	0.721	0.724	0.727	0.73	0.733
Bulgaria	0.705	0.71	0.715	0.72	0.724	0.728	0.733	0.738	0.744	0.752	0.76	0.767	0.771	0.771	0.771	0.772	0.771	0.77	0.771	0.771	0.773	0.776	0.779	0.784	0.789	0.794	0.8	0.807	0.813	0.818	0.824	0.828	0.832	0.836	0.839	0.843	0.846
Croatia	0.746	0.751	0.755	0.759	0.763	0.767	0.772	0.775	0.779	0.782	0.786	0.789	0.788	0.785	0.782	0.779	0.779	0.785	0.79	0.793	0.797	0.801	0.806	0.811	0.815	0.819	0.824	0.828	0.833	0.836	0.838	0.841	0.843	0.845	0.846	0.848	0.85
Czech Republic	0.786	0.79	0.793	0.796	0.798	0.8	0.803	0.805	0.807	0.81	0.812	0.816	0.82	0.826	0.829	0.83	0.832	0.834	0.835	0.837	0.839	0.842	0.844	0.847	0.85	0.853	0.856	0.86	0.863	0.866	0.869	0.871	0.873	0.875	0.875	0.878	0.881
Hungary	0.711	0.719	0.727	0.732	0.733	0.735	0.739	0.744	0.748	0.751	0.753	0.756	0.761	0.766	0.77	0.777	0.78	0.783	0.786	0.79	0.794	0.798	0.803	0.807	0.812	0.816	0.821	0.825	0.828	0.831	0.834	0.836	0.838	0.841	0.843	0.846	0.849
Macedonia	0.664	0.669	0.673	0.677	0.681	0.686	0.69	0.695	0.7	0.702	0.7	0.699	0.701	0.701	0.701	0.703	0.708	0.712	0.716	0.718	0.723	0.73	0.737	0.742	0.745	0.749	0.753	0.757	0.762	0.765	0.77	0.775	0.779	0.782	0.786	0.789	0.793
Montenegro	0.725	0.728	0.732	0.734	0.737	0.739	0.742	0.744	0.746	0.748	0.75	0.751	0.75	0.747	0.743	0.74	0.74	0.741	0.743	0.745	0.748	0.753	0.759	0.764	0.769	0.773	0.777	0.781	0.786	0.79	0.795	0.8	0.803	0.806	0.809	0.812	0.815
Poland	0.702	0.702	0.699	0.699	0.703	0.708	0.714	0.719	0.724	0.73	0.735	0.739	0.745	0.751	0.759	0.767	0.773	0.778	0.783	0.789	0.795	0.8	0.805	0.81	0.815	0.82	0.824	0.83	0.835	0.84	0.845	0.85	0.855	0.86	0.864	0.868	0.872
Romania	0.661	0.672	0.684	0.688	0.687	0.688	0.689	0.695	0.703	0.715	0.731	0.738	0.738	0.739	0.74	0.742	0.744	0.747	0.749	0.751	0.755	0.759	0.764	0.769	0.774	0.78	0.786	0.794	0.802	0.808	0.813	0.818	0.822	0.827	0.83	0.833	0.838
Serbia	0.678	0.68	0.682	0.683	0.684	0.686	0.689	0.691	0.694	0.697	0.7	0.703	0.704	0.701	0.699	0.697	0.698	0.702	0.707	0.709	0.712	0.714	0.717	0.722	0.728	0.733	0.737	0.742	0.747	0.752	0.755	0.759	0.761	0.764	0.767	0.769	0.771
Slovakia	0.749	0.753	0.756	0.759	0.762	0.766	0.77	0.774	0.778	0.781	0.785	0.788	0.792	0.797	0.805	0.81	0.812	0.814	0.817	0.819	0.822	0.825	0.828	0.831	0.835	0.839	0.843	0.848	0.854	0.858	0.86	0.865	0.868	0.871	0.874	0.877	0.88
Slovenia	0.766	0.774	0.782	0.789	0.793	0.797	0.801	0.803	0.808	0.813	0.813	0.813	0.812	0.812	0.813	0.815	0.817	0.82	0.823	0.827	0.831	0.835	0.839	0.843	0.847	0.851	0.856	0.86	0.863	0.865	0.867	0.869	0.871	0.873	0.874	0.877	0.881
Eastern Europe	0.737	0.738	0.738	0.739	0.742	0.743	0.744	0.747	0.753	0.76	0.767	0.775	0.782	0.782	0.78	0.777	0.774	0.771	0.768	0.766	0.766	0.768	0.77	0.774	0.78	0.786	0.792	0.8	0.806	0.81	0.813	0.817	0.819	0.821	0.823	0.826	0.829
Belarus	0.703	0.706	0.708	0.711	0.713	0.716	0.719	0.723	0.727	0.733	0.738	0.743	0.748	0.751	0.752	0.748	0.744	0.742	0.741	0.74	0.741	0.744	0.747	0.752	0.758	0.765	0.772	0.78	0.788	0.795	0.8	0.805	0.81	0.814	0.818	0.822	0.826
Estonia	0.775	0.776	0.776	0.776	0.779	0.78	0.779	0.778	0.781	0.788	0.799	0.807	0.813	0.814	0.813	0.812	0.812	0.813	0.816	0.818	0.822	0.827	0.832	0.837	0.843	0.847	0.85	0.854	0.859	0.861	0.866	0.872	0.877	0.88	0.883	0.885	0.887
Latvia	0.759	0.759	0.758	0.758	0.759	0.76	0.762	0.765	0.77	0.776	0.783	0.789	0.793	0.794	0.79	0.786	0.783	0.781	0.781	0.783	0.785	0.79	0.795	0.801	0.808	0.815	0.822	0.831	0.837	0.841	0.843	0.847	0.85	0.851	0.851	0.851	0.853
Lithuania	0.768	0.771	0.772	0.772	0.774	0.776	0.777	0.781	0.787	0.79	0.792	0.795	0.8	0.805	0.805	0.804	0.801	0.801	0.801	0.803	0.805	0.809	0.814	0.819	0.825	0.832	0.838	0.845	0.851	0.853	0.856	0.858	0.862	0.865	0.869	0.872	0.876
Moldova	0.614	0.617	0.619	0.621	0.623	0.625	0.628	0.632	0.636	0.641	0.647	0.651	0.652	0.654	0.652	0.65	0.647	0.643	0.637	0.631	0.626	0.624	0.625	0.629	0.634	0.64	0.646	0.653	0.66	0.665	0.671	0.678	0.683	0.689	0.695	0.699	0.703
Russia	0.745	0.746	0.745	0.745	0.749	0.749	0.748	0.751	0.758	0.766	0.774	0.782	0.788	0.787	0.786	0.784	0.781	0.778	0.775	0.774	0.774	0.775	0.778	0.782	0.787	0.793	0.799	0.806	0.811	0.814	0.818	0.821	0.823	0.824	0.826	0.829	0.832
Ukraine	0.712	0.715	0.717	0.719	0.721	0.724	0.727	0.731	0.737	0.742	0.748	0.753	0.757	0.76	0.758	0.753	0.748	0.742	0.737	0.732	0.729	0.728	0.729	0.733	0.74	0.747	0.755	0.763	0.77	0.774	0.778	0.782	0.785	0.789	0.791	0.792	0.793
High-income	0.773	0.778	0.782	0.787	0.791	0.795	0.799	0.803	0.806	0.809	0.813	0.817	0.822	0.826	0.83	0.834	0.837	0.84	0.843	0.845	0.848	0.852	0.854	0.856	0.859	0.86	0.861	0.863	0.866	0.868	0.871	0.873	0.875	0.877	0.879	0.881	0.882
High-income Asia Pacific	0.745	0.75	0.756	0.763	0.769	0.775	0.781	0.787	0.793	0.799	0.805	0.809	0.816	0.821	0.826	0.831	0.835	0.839	0.841	0.844	0.847	0.849	0.851	0.854	0.856	0.859	0.861	0.864	0.867	0.868	0.871	0.873	0.875	0.877	0.879	0.882	0.884
Brunei	0.674	0.681	0.687	0.694	0.7	0.706	0.713	0.721	0.729	0.737	0.744	0.751	0.756	0.761	0.766	0.772	0.777	0.783	0.789	0.796	0.803	0.809	0.815	0.821	0.827	0.833	0.839	0.844	0.849	0.853	0.856	0.858	0.861	0.863	0.866	0.869	0.871
Japan	0.776	0.779	0.781	0.784	0.788	0.793	0.798	0.803	0.81	0.817	0.822	0.828	0.833	0.837	0.84	0.844	0.847	0.85	0.852	0.854	0.856	0.858	0.859	0.861	0.863	0.865	0.867	0.869	0.871	0.872	0.873	0.875	0.877	0.878	0.88	0.882	0.884
South Korea	0.618	0.631	0.649	0.669	0.684	0.693	0.704	0.714	0.723	0.732	0.738	0.744	0.753	0.764	0.772	0.781	0.791	0.8	0.805	0.81	0.816	0.821	0.826	0.831	0.835	0.84	0.845	0.849	0.853	0.857	0.861	0.864	0.868	0.871	0.874	0.878	0.881
Singapore	0.678	0.686	0.695	0.704	0.711	0.717	0.718	0.711	0.719	0.734	0.741	0.748	0.754	0.76	0.768	0.776	0.784	0.795	0.802	0.805	0.812	0.818	0.823	0.828	0.834	0.84	0.846	0.852	0.858	0.863	0.867	0.869	0.873	0.876	0.879	0.883	0.886
Hokkaidō	0.771	0.775	0.778	0.78	0.784	0.79	0.794	0.799	0.804	0.809	0.813	0.818	0.822	0.826	0.83	0.833	0.836	0.839	0.841	0.843	0.845	0.847	0.848	0.85	0.852	0.853	0.855	0.857	0.859	0.86	0.861	0.862	0.864	0.866	0.867	0.869	0.87
Aomori	0.76	0.764	0.766	0.769	0.773	0.778	0.784	0.789	0.795	0.801	0.805	0.81	0.814	0.818	0.822	0.827	0.831	0.835	0.838	0.84	0.842	0.843	0.845	0.847	0.848	0.85	0.852	0.854	0.855	0.856	0.858	0.859	0.861	0.862	0.864	0.865	0.867
Iwate	0.752	0.756	0.758	0.761	0.766	0.771	0.776	0.78	0.786	0.792	0.797	0.802	0.807	0.811	0.816	0.821	0.826	0.83	0.833	0.835	0.838	0.84	0.842	0.844	0.846	0.848	0.849	0.851	0.853	0.854	0.856	0.857	0.859	0.86	0.862	0.864	0.865
Miyagi	0.763	0.767	0.77	0.773	0.778	0.783	0.789	0.794	0.8	0.807	0.813	0.818	0.824	0.828	0.832	0.836	0.839	0.842	0.844	0.846	0.848	0.85	0.851	0.853	0.855	0.857	0.859	0.861	0.862	0.863	0.865	0.866	0.868	0.869	0.871	0.873	0.874
Akita	0.759	0.763	0.766	0.769	0.773	0.778	0.782	0.787	0.792	0.798	0.802	0.806	0.811	0.815	0.819	0.823	0.828	0.832	0.834	0.836	0.838	0.84	0.842	0.843	0.845	0.847	0.849	0.851	0.852	0.853	0.855	0.856	0.858	0.86	0.861	0.863	0.865
Yamagata	0.75	0.755	0.757	0.76	0.764	0.768	0.772	0.777	0.782	0.788	0.793	0.798	0.803	0.807	0.812	0.817	0.821	0.825	0.829	0.831	0.834	0.837	0.84	0.843	0.845	0.847	0.849	0.851	0.853	0.854	0.855	0.857	0.858	0.86	0.862	0.863	0.865
Fukushima	0.755	0.759	0.761	0.763	0.768	0.773	0.778	0.783	0.79	0.796	0.801	0.806	0.811	0.815	0.82	0.825	0.829	0.833	0.836	0.838	0.841	0.844	0.847	0.85	0.853	0.855	0.857	0.858	0.86	0.861	0.863	0.864	0.865	0.866	0.868	0.87	0.873
Ibaraki	0.768	0.772	0.775	0.777	0.781	0.787	0.792	0.797	0.804	0.811	0.817	0.822	0.828	0.832	0.837	0.842	0.846	0.849	0.851	0.853	0.855	0.857	0.858	0.86	0.862	0.863	0.865	0.867	0.869	0.87	0.872	0.873	0.875	0.876	0.878	0.879	0.881
Tochigi	0.771	0.774	0.776	0.778	0.782	0.787	0.792	0.798	0.805	0.812	0.818	0.823	0.829	0.834	0.839	0.844	0.848	0.851	0.853	0.855	0.857	0.859	0.861	0.862	0.864	0.866	0.868	0.87	0.872	0.873	0.874	0.876	0.877	0.879	0.881	0.882	0.884
Gunma	0.767	0.771	0.773	0.775	0.779	0.784	0.789	0.794	0.8	0.807	0.812	0.818	0.823	0.827	0.832	0.837	0.841	0.845	0.847	0.849	0.851	0.853	0.855	0.856	0.858	0.86	0.862	0.864	0.866	0.867	0.868	0.87	0.871	0.873	0.875	0.876	0.878
Saitama	0.763	0.767	0.769	0.772	0.776	0.781	0.786	0.791	0.798	0.804	0.81	0.815	0.82	0.824	0.827	0.831	0.834	0.837	0.839	0.84	0.842	0.844	0.845	0.847	0.848	0.85	0.852	0.853	0.855	0.856	0.857	0.859	0.86	0.862	0.863	0.865	0.866
Chiba	0.766	0.77	0.771	0.774	0.778	0.784	0.79	0.796	0.803	0.81	0.815	0.82	0.824	0.828	0.831	0.835	0.838	0.841	0.843	0.845	0.847	0.848	0.85	0.851	0.853	0.854	0.856	0.858	0.86	0.861	0.862	0.863	0.865	0.866	0.868	0.869	0.871
Tōkyō	0.824	0.828	0.832	0.835	0.839	0.842	0.846	0.849	0.853	0.858	0.862	0.867	0.871	0.875	0.878	0.881	0.885	0.887	0.89	0.892	0.893	0.895	0.897	0.899	0.901	0.902	0.905	0.906	0.908	0.909	0.911	0.912	0.914	0.915	0.917	0.918	0.92
Kanagawa	0.775	0.779	0.781	0.784	0.788	0.793	0.798	0.803	0.81	0.817	0.821	0.826	0.83	0.834	0.838	0.841	0.844	0.847	0.849	0.851	0.853	0.854	0.856	0.858	0.859	0.861	0.863	0.865	0.866	0.867	0.869	0.87	0.871	0.873	0.875	0.876	0.878
Niigata	0.761	0.765	0.768	0.77	0.774	0.778	0.783	0.788	0.794	0.8	0.805	0.811	0.816	0.821	0.825	0.83	0.835	0.839	0.843	0.845	0.847	0.849	0.851	0.853	0.854	0.856	0.858	0.86	0.862	0.863	0.865	0.866	0.868	0.87	0.871	0.873	0.875
Toyama	0.771	0.774	0.776	0.778	0.782	0.787	0.792	0.798	0.804	0.811	0.817	0.823	0.828	0.832	0.836	0.84	0.844	0.847	0.849	0.851	0.853	0.855	0.857	0.858	0.86	0.862	0.864	0.866	0.868	0.869	0.871	0.872	0.874	0.876	0.877	0.879	0.881
Ishikawa	0.766	0.77	0.773	0.776	0.78	0.786	0.791	0.796	0.802	0.809	0.815	0.82	0.826	0.831	0.836	0.84	0.843	0.846	0.848	0.85	0.852	0.854	0.856	0.857	0.859	0.861	0.863	0.865	0.867	0.868	0.869	0.871	0.873	0.874	0.876	0.878	0.88
Fukui	0.765	0.768	0.77	0.772	0.776	0.781	0.786	0.791	0.798	0.804	0.81	0.815	0.821	0.825	0.829	0.834	0.838	0.842	0.846	0.849	0.851	0.854	0.857	0.859	0.861	0.863	0.864	0.865	0.866	0.866	0.867	0.868	0.869	0.871	0.873	0.876	0.878
Yamanashi	0.763	0.767	0.77	0.772	0.775	0.78	0.785	0.79	0.796	0.803	0.808	0.813	0.818	0.822	0.827	0.832	0.837	0.841	0.844	0.846	0.848	0.85	0.852	0.853	0.855	0.857	0.859	0.861	0.863	0.864	0.865	0.867	0.868	0.87	0.872	0.874	0.875
Nagano	0.761	0.765	0.768	0.77	0.774	0.779	0.783	0.788	0.794	0.8	0.805	0.81	0.816	0.82	0.825	0.83	0.834	0.838	0.841	0.844	0.847	0.849	0.852	0.854	0.856	0.857	0.859	0.861	0.863	0.864	0.866	0.867	0.868	0.869	0.871	0.874	0.876
Gifu	0.764	0.768	0.77	0.772	0.776	0.781	0.787	0.792	0.799	0.806	0.811	0.817	0.822	0.826	0.831	0.835	0.838	0.841	0.843	0.845	0.847	0.849	0.85	0.852	0.854	0.856	0.857	0.859	0.861	0.862	0.864	0.865	0.867	0.868	0.87	0.872	0.873
Shizuoka	0.777	0.78	0.781	0.783	0.787	0.793	0.798	0.803	0.81	0.817	0.823	0.829	0.834	0.839	0.843	0.847	0.851	0.854	0.856	0.858	0.86	0.861	0.863	0.865	0.866	0.868	0.87	0.872	0.874	0.874	0.875	0.876	0.877	0.879	0.881	0.883	0.886
Aichi	0.786	0.79	0.792	0.794	0.797	0.803	0.808	0.813	0.82	0.828	0.833	0.839	0.845	0.849	0.852	0.856	0.859	0.862	0.864	0.866	0.868	0.87	0.871	0.873	0.875	0.877	0.878	0.88	0.882	0.883	0.885	0.886	0.887	0.888	0.89	0.893	0.895
Mie	0.77	0.774	0.776	0.778	0.782	0.788	0.793	0.798	0.805	0.812	0.818	0.824	0.83	0.834	0.839	0.843	0.846	0.849	0.851	0.853	0.855	0.857	0.859	0.86	0.862	0.864	0.866	0.868	0.87	0.871	0.873	0.874	0.875	0.877	0.879	0.881	0.883
Shiga	0.77	0.773	0.775	0.777	0.781	0.787	0.792	0.798	0.805	0.812	0.819	0.825	0.831	0.835	0.84	0.845	0.849	0.853	0.857	0.859	0.862	0.864	0.865	0.867	0.869	0.871	0.873	0.875	0.876	0.877	0.878	0.879	0.88	0.881	0.883	0.886	0.889
Kyōto	0.776	0.779	0.781	0.783	0.787	0.792	0.797	0.803	0.81	0.816	0.821	0.825	0.83	0.834	0.838	0.841	0.845	0.848	0.85	0.852	0.854	0.855	0.857	0.859	0.861	0.862	0.864	0.866	0.868	0.869	0.871	0.872	0.874	0.876	0.877	0.879	0.881
Ōsaka	0.788	0.791	0.793	0.795	0.799	0.804	0.809	0.814	0.821	0.827	0.832	0.836	0.841	0.845	0.848	0.852	0.855	0.858	0.86	0.861	0.863	0.865	0.866	0.868	0.869	0.871	0.873	0.875	0.877	0.878	0.879	0.88	0.882	0.884	0.885	0.887	0.888
Hyōgo	0.765	0.768	0.77	0.773	0.777	0.783	0.788	0.794	0.801	0.808	0.814	0.818	0.823	0.827	0.831	0.834	0.838	0.841	0.843	0.845	0.847	0.848	0.85	0.851	0.853	0.855	0.857	0.859	0.861	0.862	0.863	0.865	0.866	0.868	0.869	0.871	0.873
Nara	0.749	0.753	0.754	0.757	0.762	0.768	0.773	0.779	0.786	0.792	0.797	0.802	0.807	0.811	0.814	0.818	0.821	0.824	0.826	0.828	0.83	0.832	0.833	0.835	0.836	0.838	0.84	0.842	0.843	0.844	0.846	0.847	0.849	0.85	0.852	0.853	0.855
Wakayama	0.757	0.761	0.763	0.766	0.77	0.775	0.781	0.787	0.793	0.8	0.806	0.812	0.817	0.821	0.826	0.829	0.833	0.836	0.838	0.84	0.842	0.844	0.845	0.847	0.849	0.85	0.852	0.854	0.856	0.857	0.859	0.86	0.862	0.864	0.865	0.867	0.869

Appendix Table 7: Socio-demographic Index values for all estimated GBD 2016 locations, 1980–2016

Location	1980	1981	1982	1983	1984	1985	1986	1987	1988	1989	1990	1991	1992	1993	1994	1995	1996	1997	1998	1999	2000	2001	2002	2003	2004	2005	2006	2007	2008	2009	2010	2011	2012	2013	2014	2015	2016
Tottori	0.747	0.751	0.753	0.755	0.759	0.764	0.769	0.774	0.78	0.786	0.791	0.797	0.803	0.807	0.812	0.817	0.822	0.826	0.83	0.832	0.835	0.837	0.84	0.843	0.845	0.847	0.849	0.851	0.853	0.854	0.856	0.857	0.858	0.859	0.861	0.864	0.866
Shimane	0.744	0.748	0.75	0.752	0.756	0.761	0.766	0.771	0.777	0.783	0.788	0.794	0.799	0.803	0.808	0.813	0.818	0.823	0.827	0.83	0.833	0.836	0.839	0.842	0.845	0.847	0.847	0.849	0.85	0.85	0.851	0.852	0.853	0.854	0.856	0.859	0.862
Okayama	0.762	0.765	0.767	0.769	0.773	0.779	0.784	0.79	0.797	0.804	0.809	0.815	0.821	0.825	0.83	0.835	0.839	0.843	0.845	0.847	0.849	0.851	0.853	0.855	0.857	0.859	0.861	0.863	0.865	0.866	0.867	0.869	0.871	0.872	0.874	0.876	0.878
Hiroshima	0.77	0.773	0.775	0.777	0.781	0.787	0.792	0.797	0.804	0.811	0.816	0.822	0.828	0.833	0.837	0.842	0.845	0.848	0.85	0.852	0.854	0.856	0.857	0.859	0.861	0.863	0.865	0.867	0.869	0.869	0.869	0.87	0.871	0.872	0.874	0.877	0.881
Yamaguchi	0.767	0.771	0.773	0.775	0.779	0.784	0.789	0.795	0.801	0.808	0.813	0.818	0.824	0.828	0.832	0.837	0.84	0.843	0.846	0.848	0.85	0.851	0.853	0.855	0.857	0.859	0.861	0.863	0.865	0.866	0.867	0.867	0.868	0.87	0.872	0.875	0.878
Tokushima	0.762	0.765	0.768	0.77	0.774	0.78	0.785	0.79	0.796	0.803	0.808	0.814	0.82	0.824	0.828	0.832	0.835	0.838	0.841	0.843	0.845	0.847	0.849	0.85	0.852	0.854	0.856	0.858	0.86	0.861	0.862	0.864	0.866	0.867	0.869	0.871	0.873
Kagawa	0.764	0.768	0.77	0.772	0.776	0.781	0.786	0.791	0.798	0.805	0.811	0.817	0.822	0.827	0.831	0.835	0.839	0.842	0.844	0.846	0.848	0.85	0.852	0.854	0.855	0.857	0.859	0.861	0.863	0.863	0.864	0.865	0.866	0.867	0.869	0.872	0.875
Ehime	0.757	0.761	0.764	0.767	0.771	0.776	0.781	0.786	0.792	0.799	0.804	0.809	0.815	0.819	0.823	0.828	0.832	0.835	0.837	0.839	0.841	0.843	0.845	0.847	0.849	0.851	0.852	0.855	0.856	0.857	0.859	0.861	0.862	0.864	0.866	0.867	0.869
Kōchi	0.746	0.749	0.751	0.753	0.757	0.763	0.768	0.774	0.78	0.787	0.792	0.796	0.801	0.805	0.81	0.815	0.819	0.823	0.825	0.827	0.829	0.831	0.833	0.835	0.837	0.839	0.84	0.843	0.844	0.845	0.847	0.848	0.85	0.852	0.853	0.855	0.857
Fukuoka	0.769	0.773	0.775	0.777	0.782	0.787	0.792	0.798	0.804	0.811	0.816	0.822	0.826	0.83	0.834	0.837	0.841	0.844	0.846	0.848	0.85	0.852	0.853	0.855	0.857	0.859	0.861	0.863	0.865	0.866	0.867	0.869	0.87	0.872	0.874	0.876	0.877
Saga	0.751	0.755	0.757	0.76	0.764	0.769	0.775	0.78	0.786	0.793	0.798	0.803	0.808	0.812	0.816	0.821	0.825	0.828	0.831	0.834	0.836	0.839	0.843	0.846	0.849	0.85	0.852	0.853	0.854	0.855	0.856	0.857	0.857	0.859	0.861	0.863	0.866
Nagasaki	0.749	0.753	0.755	0.757	0.761	0.767	0.772	0.777	0.783	0.79	0.794	0.799	0.804	0.808	0.813	0.818	0.822	0.826	0.829	0.832	0.834	0.837	0.839	0.841	0.843	0.845	0.847	0.848	0.849	0.849	0.849	0.85	0.851	0.852	0.854	0.857	0.859
Kumamoto	0.75	0.754	0.757	0.759	0.763	0.768	0.773	0.778	0.784	0.791	0.796	0.801	0.806	0.81	0.814	0.819	0.823	0.827	0.83	0.832	0.835	0.837	0.839	0.841	0.843	0.845	0.846	0.846	0.847	0.848	0.849	0.85	0.851	0.852	0.854	0.857	0.86
Ôita	0.761	0.765	0.768	0.771	0.775	0.78	0.785	0.789	0.795	0.802	0.807	0.812	0.817	0.821	0.825	0.83	0.834	0.838	0.841	0.843	0.845	0.847	0.849	0.85	0.852	0.854	0.856	0.858	0.86	0.86	0.861	0.862	0.863	0.865	0.867	0.87	0.872
Miyazaki	0.749	0.752	0.755	0.757	0.762	0.767	0.773	0.778	0.784	0.79	0.795	0.799	0.804	0.808	0.812	0.816	0.821	0.825	0.828	0.831	0.833	0.836	0.839	0.841	0.844	0.845	0.845	0.846	0.847	0.847	0.847	0.848	0.849	0.85	0.853	0.855	0.858
Kagoshima	0.744	0.748	0.751	0.754	0.759	0.765	0.77	0.775	0.782	0.788	0.793	0.799	0.804	0.808	0.813	0.818	0.822	0.826	0.83	0.832	0.834	0.837	0.84	0.842	0.844	0.846	0.846	0.847	0.848	0.849	0.85	0.851	0.852	0.853	0.855	0.858	0.861
Okinawa	0.73	0.734	0.736	0.738	0.743	0.75	0.756	0.762	0.769	0.777	0.782	0.787	0.793	0.797	0.802	0.808	0.812	0.816	0.819	0.821	0.823	0.825	0.828	0.832	0.835	0.836	0.836	0.837	0.837	0.837	0.837	0.838	0.838	0.84	0.842	0.845	0.848
Australasia	0.787	0.79	0.793	0.796	0.799	0.802	0.805	0.808	0.811	0.813	0.815	0.818	0.821	0.824	0.828	0.832	0.836	0.839	0.843	0.846	0.849	0.853	0.856	0.858	0.86	0.862	0.861	0.861	0.864	0.867	0.87	0.872	0.875	0.88	0.884	0.887	0.889
Australia	0.787	0.789	0.792	0.796	0.799	0.802	0.806	0.81	0.813	0.815	0.818	0.82	0.823	0.826	0.83	0.834	0.838	0.842	0.845	0.849	0.853	0.856	0.859	0.862	0.864	0.865	0.865	0.865	0.868	0.872	0.875	0.876	0.879	0.883	0.887	0.89	0.892
New Zealand	0.786	0.79	0.794	0.797	0.799	0.8	0.8	0.8	0.8	0.8	0.803	0.807	0.81	0.813	0.816	0.819	0.822	0.826	0.828	0.828	0.831	0.836	0.839	0.84	0.841	0.843	0.84	0.838	0.841	0.844	0.847	0.851	0.856	0.86	0.863	0.865	0.867
Western Europe	0.754	0.759	0.765	0.771	0.775	0.779	0.784	0.788	0.792	0.797	0.801	0.807	0.812	0.817	0.821	0.825	0.828	0.832	0.835	0.838	0.842	0.845	0.848	0.85	0.852	0.854	0.856	0.858	0.859	0.861	0.864	0.866	0.869	0.871	0.873	0.874	0.876
Andorra	0.845	0.848	0.85	0.852	0.855	0.857	0.859	0.862	0.864	0.866	0.868	0.87	0.872	0.873	0.875	0.876	0.878	0.881	0.884	0.887	0.889	0.891	0.892	0.894	0.896	0.897	0.899	0.901	0.902	0.904	0.906	0.907	0.909	0.91	0.912	0.913	0.915
Austria	0.801	0.803	0.807	0.812	0.816	0.818	0.82	0.822	0.824	0.827	0.83	0.832	0.836	0.838	0.841	0.844	0.846	0.849	0.851	0.854	0.857	0.86	0.862	0.864	0.867	0.869	0.871	0.874	0.876	0.878	0.88	0.882	0.885	0.887	0.889	0.89	0.892
Belgium	0.813	0.818	0.823	0.827	0.831	0.833	0.835	0.837	0.84	0.841	0.843	0.846	0.85	0.855	0.859	0.861	0.862	0.865	0.867	0.869	0.871	0.874	0.877	0.877	0.877	0.878	0.879	0.88	0.882	0.883	0.886	0.89	0.893	0.896	0.897	0.898	0.899
Cyprus	0.692	0.696	0.699	0.704	0.712	0.72	0.726	0.73	0.734	0.739	0.745	0.748	0.754	0.765	0.774	0.783	0.79	0.798	0.806	0.812	0.819	0.826	0.831	0.835	0.839	0.843	0.847	0.852	0.856	0.86	0.863	0.866	0.868	0.869	0.87	0.871	0.872
Denmark	0.866	0.867	0.869	0.871	0.873	0.876	0.879	0.88	0.879	0.879	0.88	0.88	0.88	0.881	0.882	0.885	0.889	0.893	0.895	0.897	0.9	0.903	0.905	0.905	0.906	0.906	0.907	0.908	0.909	0.911	0.914	0.919	0.922	0.925	0.925	0.925	0.925
Finland	0.815	0.816	0.818	0.821	0.826	0.832	0.836	0.837	0.838	0.84	0.841	0.841	0.842	0.843	0.845	0.849	0.852	0.856	0.86	0.863	0.867	0.871	0.874	0.876	0.878	0.88	0.883	0.886	0.888	0.89	0.892	0.896	0.9	0.904	0.906	0.907	0.907
France	0.743	0.749	0.758	0.765	0.769	0.773	0.777	0.783	0.788	0.793	0.799	0.805	0.812	0.817	0.821	0.823	0.826	0.829	0.832	0.833	0.836	0.84	0.843	0.846	0.848	0.849	0.85	0.853	0.855	0.856	0.858	0.861	0.863	0.865	0.867	0.868	0.869
Germany	0.801	0.804	0.807	0.809	0.812	0.814	0.817	0.82	0.822	0.826	0.829	0.832	0.835	0.838	0.841	0.843	0.845	0.847	0.85	0.852	0.854	0.857	0.859	0.86	0.862	0.864	0.866	0.869	0.871	0.873	0.875	0.877	0.88	0.882	0.884	0.886	0.889
Greece	0.699	0.708	0.714	0.722	0.731	0.739	0.745	0.751	0.756	0.761	0.765	0.77	0.775	0.779	0.783	0.787	0.792	0.796	0.801	0.806	0.81	0.815	0.82	0.825	0.829	0.834	0.838	0.843	0.847	0.85	0.852	0.853	0.853	0.852	0.852	0.852	0.853
Iceland	0.808	0.817	0.823	0.83	0.841	0.848	0.847	0.842	0.842	0.844	0.847	0.852	0.852	0.855	0.861	0.862	0.865	0.869	0.873	0.875	0.879	0.886	0.888	0.888	0.889	0.892	0.894	0.895	0.894	0.894	0.901	0.906	0.909	0.914	0.918	0.92	0.921
Ireland	0.684	0.694	0.705	0.717	0.727	0.733	0.74	0.75	0.759	0.764	0.768	0.775	0.784	0.792	0.798	0.802	0.806	0.811	0.818	0.826	0.832	0.836	0.842	0.848	0.856	0.861	0.861	0.861	0.863	0.864	0.867	0.871	0.874	0.877	0.879	0.883	0.885
Israel	0.691	0.698	0.7	0.7	0.702	0.708	0.716	0.723	0.727	0.731	0.736	0.743	0.748	0.753	0.757	0.761	0.764	0.766	0.767	0.769	0.772	0.775	0.777	0.777	0.779	0.781	0.784	0.786	0.786	0.788	0.791	0.794	0.796	0.797	0.798	0.799	0.801
Italy	0.748	0.755	0.761	0.767	0.772	0.776	0.781	0.785	0.789	0.794	0.798	0.803	0.807	0.81	0.814	0.818	0.821	0.824	0.828	0.831	0.834	0.837	0.84	0.843	0.846	0.848	0.85	0.853	0.855	0.857	0.858	0.86	0.862	0.863	0.864	0.865	0.867
Luxembourg	0.848	0.851	0.853	0.855	0.858	0.861	0.864	0.868	0.871	0.873	0.876	0.88	0.882	0.885	0.888	0.891	0.893	0.898	0.9	0.9	0.904	0.909	0.912	0.912	0.913	0.915	0.917	0.919	0.921	0.922	0.924	0.928	0.929	0.932	0.934	0.935	0.936
Malta	0.717	0.723	0.728	0.733	0.738	0.741	0.743	0.743	0.743	0.747	0.752	0.754	0.759	0.768	0.775	0.777	0.778	0.786	0.795	0.803	0.814	0.819	0.822	0.825	0.827	0.829	0.831	0.834	0.836	0.838	0.84	0.842	0.845	0.848	0.85	0.853	0.856
Netherlands	0.825	0.83	0.833	0.835	0.838	0.839	0.84	0.843	0.846	0.848	0.85	0.854	0.859	0.862	0.866	0.871	0.873	0.874	0.876	0.878	0.88	0.884	0.886	0.889	0.893	0.896	0.898	0.9	0.901	0.902	0.905	0.909	0.913	0.915	0.917	0.917	0.918
Norway	0.824	0.828	0.833	0.837	0.84	0.843	0.845	0.845	0.845	0.845	0.847	0.851	0.856	0.859	0.862	0.864	0.868	0.874	0.877	0.88	0.885	0.891	0.894	0.894	0.896	0.897	0.899	0.9	0.901	0.903	0.907	0.912	0.916	0.92	0.922	0.922	0.922
Portugal	0.61	0.618	0.626	0.634	0.643	0.651	0.658	0.665	0.672	0.68	0.687	0.694	0.701	0.708	0.714	0.719	0.725	0.73	0.735	0.74	0.746	0.751	0.755	0.759	0.763	0.766	0.77	0.773	0.777	0.779	0.782	0.785	0.787	0.789	0.791	0.794	0.796
Spain	0.67	0.68	0.689	0.698	0.705	0.713	0.721	0.727	0.733	0.739	0.746	0.752	0.759	0.764	0.77	0.775	0.78	0.786	0.791	0.796	0.801	0.806	0.811	0.816	0.82	0.824	0.828	0.833	0.836	0.838	0.84	0.842	0.843	0.844	0.845	0.847	0.848
Sweden	0.799	0.803	0.806	0.808	0.808	0.808	0.809	0.808	0.807	0.806	0.807	0.81	0.816	0.822	0.829	0.838	0.845	0.849	0.853	0.855	0.857	0.857	0.857	0.857	0.858	0.858	0.858	0.859	0.86	0.859	0.86	0.864	0.867	0.869	0.87	0.871	0.872
Switzerland	0.867	0.867	0.868	0.868	0.869	0.869	0.869	0.869	0.869	0.869	0.869	0.87	0.872	0.874	0.874	0.874	0.874	0.874	0.875	0.875	0.876	0.877	0.878	0.879	0.88	0.881	0.883	0.885	0.887	0.888	0.889	0.891	0.893	0.895	0.896	0.899	0.901
United Kingdom	0.711	0.717	0.722	0.727	0.732	0.737	0.742	0.746	0.753	0.758	0.763	0.769	0.775	0.781	0.787	0.793	0.797	0.802	0.808	0.815	0.822	0.827	0.83	0.831	0.834	0.837	0.838	0.84	0.842	0.843	0.846	0.849	0.853	0.857	0.86	0.862	0.863
Sweden except Stockholm	0.774	0.778	0.782	0.784	0.784	0.784	0.785	0.785	0.784	0.783	0.784	0.788	0.794	0.801	0.809	0.819	0.826	0.831	0.834	0.837	0.839	0.84	0.839	0.84	0.841	0.841	0.841	0.843	0.845	0.844	0.846	0.85	0.854	0.856	0.858	0.86	0.861
Stockholm	0.866	0.868	0.87	0.871	0.873	0.875	0.877	0.879	0.88	0.879	0.879	0.882	0.885	0.889	0.891	0.892	0.893	0.895	0.897	0.899	0.901	0.903	0.905	0.906	0.908	0.909	0.908	0.907	0.906	0.904	0.904	0.905	0.906	0.906	0.905	0.905	0.905
Northern Ireland	0.718	0.726	0.732	0.737	0.742	0.747	0.752	0.759	0.768	0.775	0.782	0.788	0.795	0.802	0.807	0.811	0.815	0.82	0.826	0.832	0.838	0.842	0.846	0.848	0.85	0.852	0.852	0.851	0.853	0.855	0.856	0.857	0.859	0.863	0.866	0.87	0.872
Scotland	0.706	0.712	0.718	0.724	0.728	0.732	0.737	0.742	0.748	0.752	0.755	0.759	0.764	0.77	0.775	0.779	0.782	0.786	0.792	0.797	0.801	0.805	0.809	0.811	0.813	0.815	0.816	0.816	0.818	0.821	0.823	0.826	0.83	0.832	0.834	0.835	0.837
Wales	0.681	0.686	0.692	0.697	0.702	0.708	0.713	0.718	0.726	0.733	0.737	0.742	0.749	0.755	0.762	0.767	0.772	0.778	0.785	0.792	0.8	0.807	0.811	0.813	0.816	0.819	0.821	0.822	0.825	0.827	0.829	0.833	0.837	0.842	0.845	0.847	0.848
England	0.712	0.718	0.723	0.729	0.733	0.738	0.743	0.747	0.753	0.759	0.765	0.771	0.777	0.783	0.789	0.794	0.799	0.804	0.81	0.817	0.824	0.829	0.832	0.834	0.837	0.839	0.841	0.842	0.844	0.846	0.848	0.851	0.855	0.86	0.863	0.865	0.866
Darlington	0.695	0.701	0.708	0.714	0.719	0.725	0.731	0.735	0.741	0.744	0.746	0.75	0.756	0.764	0.772	0.779	0.785	0.791	0.796	0.802	0.808	0.812	0.813	0.814	0.817	0.82	0.823	0.824	0.824	0.825	0.831	0.836	0.841	0.846	0.848	0.848	0.849
Northumberland	0.683	0.689	0.695	0.701	0.707	0.713	0.72	0.726	0.733	0.738	0.742	0.746	0.75	0.756	0.762	0.769	0.775	0.781	0.787	0.792	0.797	0.8	0.802	0.804	0.806	0.808	0.81	0.812	0.815	0.817	0.819	0.821	0.825	0.828	0.83	0.831	0.832
Stockton-on-Tees	0.692	0.698	0.703	0.708	0.714	0.721	0.727	0.733	0.74	0.745	0.748	0.753	0.759	0.766	0.774	0.78	0.786	0.792	0.798	0.806	0.812	0.815	0.817	0.818	0.82	0.822	0.824	0.825	0.827	0.829	0.831	0.833	0.835	0.84	0.843	0.846	0.848
Newcastle upon Tyne	0.705	0.711	0.717	0.722	0.726	0.731	0.737	0.743	0.75	0.756	0.763	0.771	0.78	0.787	0.792	0.798	0.802	0.806	0.811	0.819	0.827	0.833	0.84	0.846	0.852	0.858	0.861	0.865	0.868	0.871	0.873	0.876	0.879	0.882	0.885	0.887	0.889
North Tyneside	0.682	0.687	0.692	0.697	0.701	0.706	0.711	0.718	0.725	0.731	0.737	0.742	0.749	0.756	0.762	0.768	0.772	0.778	0.784	0.79	0.797	0.802	0.805	0.807	0.811	0.816	0.82	0.824	0.827	0.828	0.831	0.834	0.837	0.84	0.842	0.844	0.846
Redcar and Cleveland	0.659	0.665	0.67	0.676	0.68	0.686	0.691	0.697	0.704	0.71	0.714	0.718	0.723	0.727	0.733	0.739	0.744	0.75	0.757	0.766	0.774	0.779	0.782	0.783	0.786	0.789	0.791	0.793	0.795	0.795	0.796	0.797	0.802	0.808	0.813	0.816	0.819
County Durham	0.673	0.679	0.685	0.69	0.696	0.702	0.708	0.713	0.719	0.724	0.728	0.733	0.74	0.746	0.753	0.759	0.764	0.771	0.777	0.784	0.791	0.796	0.799	0.8	0.803	0.805	0.807	0.809	0.812	0.813	0.816	0.818	0.822	0.827	0.83	0.832	0.833
Gateshead	0.687	0.692	0.698	0.703	0.708	0.713	0.719	0.725	0.732	0.738	0.744	0.749	0.755	0.761	0.768	0.774	0.779	0.785	0.791	0.797	0.803	0.808	0.811	0.814	0.818	0.821	0.824	0.826	0.828	0.83	0.833	0.837	0.84	0.844	0.847	0.849	0.851

Appendix Table 7: Socio-demographic Index values for all estimated GBD 2016 locations, 1980–2016

Location	1980	1981	1982	1983	1984	1985	1986	1987	1988	1989	1990	1991	1992	1993	1994	1995	1996	1997	1998	1999	2000	2001	2002	2003	2004	2005	2006	2007	2008	2009	2010	2011	2012	2013	2014	2015	2016
Middlesbrough	0.668	0.674	0.68	0.686	0.69	0.695	0.699	0.705	0.713	0.72	0.727	0.735	0.741	0.748	0.755	0.761	0.767	0.772	0.778	0.785	0.791	0.794	0.795	0.795	0.8	0.804	0.808	0.813	0.816	0.817	0.818	0.819	0.821	0.824	0.826	0.827	0.829
South Tyneside	0.657	0.662	0.666	0.669	0.672	0.675	0.68	0.686	0.694	0.701	0.707	0.713	0.719	0.725	0.731	0.736	0.741	0.746	0.753	0.761	0.768	0.775	0.78	0.784	0.789	0.793	0.796	0.799	0.802	0.803	0.805	0.807	0.81	0.814	0.817	0.818	0.82
Sunderland	0.676	0.683	0.689	0.694	0.7	0.706	0.712	0.718	0.725	0.731	0.736	0.742	0.748	0.754	0.761	0.767	0.772	0.778	0.785	0.791	0.798	0.804	0.809	0.812	0.816	0.82	0.823	0.826	0.829	0.83	0.831	0.832	0.835	0.838	0.841	0.844	0.845
Hartlepool	0.672	0.678	0.683	0.687	0.691	0.695	0.7	0.704	0.711	0.718	0.723	0.728	0.734	0.74	0.746	0.753	0.759	0.765	0.77	0.777	0.782	0.785	0.787	0.788	0.79	0.793	0.795	0.799	0.802	0.805	0.807	0.81	0.814	0.819	0.82	0.821	0.821
Cheshire East	0.726	0.732	0.737	0.742	0.747	0.753	0.758	0.764	0.769	0.774	0.778	0.783	0.788	0.795	0.802	0.809	0.815	0.821	0.826	0.832	0.837	0.842	0.845	0.845	0.847	0.848	0.848	0.847	0.848	0.851	0.854	0.858	0.863	0.869	0.872	0.873	0.874
Stockport	0.702	0.708	0.714	0.719	0.724	0.73	0.734	0.74	0.747	0.753	0.757	0.763	0.77	0.778	0.784	0.789	0.794	0.8	0.806	0.813	0.82	0.825	0.828	0.829	0.831	0.833	0.835	0.836	0.837	0.838	0.839	0.841	0.844	0.849	0.853	0.855	0.857
Trafford	0.726	0.732	0.737	0.741	0.745	0.75	0.755	0.76	0.767	0.772	0.777	0.784	0.791	0.799	0.806	0.812	0.816	0.821	0.828	0.835	0.841	0.846	0.849	0.851	0.854	0.855	0.855	0.856	0.858	0.86	0.864	0.868	0.872	0.877	0.88	0.882	0.883
Cheshire West and Chester	0.714	0.72	0.726	0.731	0.736	0.741	0.747	0.751	0.756	0.761	0.766	0.773	0.78	0.787	0.792	0.795	0.796	0.799	0.803	0.812	0.822	0.831	0.837	0.839	0.841	0.843	0.845	0.846	0.847	0.849	0.852	0.857	0.862	0.866	0.868	0.869	0.869
Sefton	0.682	0.688	0.693	0.698	0.704	0.709	0.715	0.72	0.725	0.73	0.734	0.74	0.747	0.754	0.761	0.767	0.771	0.777	0.783	0.789	0.795	0.8	0.803	0.805	0.809	0.811	0.812	0.813	0.813	0.814	0.816	0.818	0.82	0.823	0.824	0.825	0.826
Lancashire	0.694	0.7	0.705	0.711	0.716	0.721	0.727	0.732	0.738	0.744	0.749	0.755	0.761	0.768	0.775	0.781	0.786	0.792	0.798	0.804	0.81	0.815	0.817	0.819	0.822	0.824	0.826	0.827	0.829	0.83	0.832	0.834	0.838	0.842	0.845	0.847	0.848
Cumbria	0.702	0.707	0.713	0.718	0.723	0.73	0.736	0.741	0.747	0.751	0.754	0.757	0.763	0.77	0.777	0.783	0.789	0.794	0.8	0.804	0.809	0.812	0.814	0.815	0.818	0.82	0.821	0.821	0.823	0.825	0.828	0.832	0.837	0.842	0.845	0.846	0.848
Bolton	0.678	0.684	0.69	0.695	0.701	0.707	0.713	0.719	0.725	0.73	0.735	0.741	0.747	0.754	0.761	0.766	0.77	0.774	0.78	0.786	0.791	0.795	0.797	0.798	0.8	0.8	0.8	0.8	0.802	0.804	0.807	0.81	0.814	0.819	0.821	0.823	0.824
Wirral	0.677	0.682	0.687	0.692	0.696	0.7	0.705	0.71	0.716	0.722	0.727	0.733	0.739	0.746	0.753	0.76	0.765	0.771	0.777	0.783	0.787	0.79	0.791	0.792	0.795	0.797	0.798	0.798	0.799	0.8	0.803	0.808	0.812	0.817	0.819	0.82	0.82
Bury	0.683	0.688	0.693	0.697	0.7	0.704	0.709	0.714	0.72	0.727	0.733	0.739	0.747	0.754	0.761	0.766	0.77	0.774	0.78	0.787	0.793	0.797	0.799	0.8	0.803	0.806	0.807	0.806	0.806	0.805	0.808	0.812	0.818	0.824	0.828	0.83	0.831
St Helens	0.67	0.675	0.681	0.686	0.691	0.697	0.703	0.709	0.715	0.72	0.722	0.725	0.731	0.738	0.745	0.75	0.755	0.76	0.766	0.772	0.78	0.786	0.79	0.792	0.796	0.799	0.801	0.804	0.806	0.808	0.81	0.812	0.816	0.822	0.826	0.828	0.83
Warrington	0.719	0.724	0.73	0.735	0.741	0.747	0.753	0.759	0.766	0.771	0.774	0.777	0.783	0.789	0.796	0.802	0.807	0.812	0.817	0.824	0.83	0.836	0.841	0.844	0.847	0.85	0.852	0.852	0.852	0.854	0.858	0.862	0.867	0.873	0.875	0.877	0.878
Oldham	0.669	0.674	0.679	0.684	0.689	0.694	0.699	0.704	0.71	0.715	0.72	0.725	0.731	0.738	0.743	0.747	0.751	0.754	0.758	0.763	0.769	0.773	0.775	0.776	0.778	0.78	0.782	0.784	0.787	0.789	0.792	0.796	0.8	0.804	0.806	0.807	0.808
Rochdale	0.668	0.673	0.679	0.684	0.688	0.694	0.7	0.705	0.712	0.716	0.72	0.725	0.731	0.737	0.744	0.75	0.755	0.76	0.767	0.773	0.779	0.783	0.784	0.785	0.787	0.788	0.788	0.788	0.789	0.791	0.795	0.8	0.805	0.81	0.813	0.814	0.815
Wigan	0.671	0.676	0.682	0.687	0.692	0.698	0.704	0.71	0.716	0.722	0.727	0.733	0.739	0.746	0.752	0.758	0.763	0.769	0.774	0.779	0.785	0.789	0.791	0.793	0.795	0.797	0.798	0.799	0.8	0.801	0.804	0.807	0.811	0.816	0.82	0.822	0.824
Halton	0.69	0.695	0.7	0.704	0.707	0.711	0.715	0.72	0.727	0.734	0.739	0.745	0.752	0.759	0.767	0.773	0.778	0.782	0.786	0.792	0.8	0.806	0.81	0.811	0.812	0.814	0.816	0.818	0.82	0.822	0.827	0.832	0.836	0.842	0.847	0.85	0.853
Liverpool	0.688	0.693	0.698	0.702	0.705	0.709	0.712	0.716	0.722	0.729	0.738	0.75	0.76	0.767	0.773	0.777	0.781	0.787	0.794	0.803	0.812	0.818	0.824	0.83	0.836	0.839	0.842	0.844	0.848	0.852	0.855	0.859	0.862	0.865	0.867	0.868	0.87
Tameside	0.672	0.678	0.683	0.688	0.692	0.697	0.702	0.707	0.713	0.719	0.724	0.73	0.737	0.744	0.751	0.756	0.761	0.766	0.771	0.778	0.786	0.792	0.795	0.797	0.8	0.802	0.801	0.801	0.8	0.8	0.802	0.805	0.809	0.813	0.816	0.818	0.82
Salford	0.691	0.696	0.701	0.706	0.71	0.714	0.719	0.723	0.728	0.732	0.737	0.744	0.752	0.761	0.769	0.775	0.78	0.784	0.791	0.799	0.807	0.814	0.818	0.821	0.825	0.828	0.831	0.832	0.834	0.835	0.839	0.843	0.848	0.853	0.856	0.859	0.861
Blackburn with Darwen	0.669	0.675	0.68	0.685	0.689	0.694	0.698	0.702	0.709	0.715	0.72	0.726	0.733	0.739	0.745	0.75	0.755	0.76	0.765	0.77	0.775	0.779	0.781	0.782	0.784	0.786	0.786	0.787	0.791	0.794	0.798	0.802	0.807	0.812	0.816	0.818	0.82
Knowsley	0.665	0.672	0.678	0.683	0.689	0.694	0.7	0.704	0.71	0.715	0.72	0.727	0.733	0.739	0.744	0.75	0.755	0.762	0.768	0.774	0.781	0.787	0.791	0.792	0.795	0.799	0.803	0.807	0.811	0.813	0.816	0.819	0.822	0.828	0.832	0.834	0.837
Blackpool	0.667	0.673	0.679	0.685	0.688	0.69	0.691	0.693	0.699	0.704	0.711	0.722	0.732	0.74	0.746	0.751	0.754	0.759	0.766	0.773	0.778	0.782	0.782	0.78	0.782	0.786	0.788	0.788	0.787	0.788	0.792	0.796	0.799	0.801	0.803	0.806	0.809
Manchester	0.715	0.721	0.726	0.731	0.734	0.739	0.744	0.749	0.755	0.761	0.768	0.775	0.781	0.789	0.796	0.801	0.806	0.812	0.819	0.829	0.839	0.847	0.853	0.857	0.862	0.866	0.869	0.872	0.875	0.878	0.882	0.884	0.888	0.892	0.895	0.897	0.899
North Yorkshire	0.707	0.712	0.718	0.724	0.73	0.736	0.742	0.747	0.754	0.759	0.762	0.765	0.769	0.774	0.78	0.785	0.79	0.796	0.802	0.808	0.815	0.819	0.821	0.822	0.826	0.829	0.831	0.833	0.834	0.835	0.838	0.841	0.844	0.848	0.849	0.849	0.85
East Riding of Yorkshire	0.696	0.702	0.708	0.714	0.72	0.724	0.727	0.726	0.73	0.738	0.746	0.752	0.758	0.765	0.771	0.777	0.782	0.788	0.793	0.799	0.804	0.808	0.81	0.811	0.815	0.818	0.819	0.821	0.823	0.825	0.827	0.828	0.831	0.834	0.836	0.837	0.839
York	0.729	0.735	0.74	0.745	0.749	0.753	0.756	0.76	0.766	0.773	0.781	0.79	0.799	0.804	0.81	0.815	0.819	0.824	0.831	0.836	0.842	0.848	0.854	0.86	0.865	0.87	0.873	0.877	0.88	0.881	0.883	0.885	0.886	0.888	0.89	0.892	0.894
North East Lincolnshire	0.693	0.698	0.703	0.706	0.709	0.714	0.721	0.728	0.736	0.74	0.743	0.748	0.755	0.762	0.769	0.773	0.776	0.779	0.784	0.789	0.795	0.801	0.802	0.802	0.803	0.805	0.808	0.813	0.817	0.822	0.825	0.828	0.831	0.833	0.834	0.834	0.834
Calderdale	0.688	0.694	0.699	0.704	0.709	0.716	0.722	0.729	0.736	0.741	0.746	0.75	0.755	0.761	0.767	0.773	0.777	0.783	0.788	0.795	0.801	0.805	0.806	0.807	0.81	0.814	0.816	0.818	0.82	0.821	0.824	0.828	0.833	0.839	0.842	0.845	0.847
North Lincolnshire	0.691	0.696	0.701	0.706	0.71	0.715	0.72	0.724	0.731	0.736	0.741	0.746	0.751	0.757	0.763	0.769	0.775	0.781	0.787	0.792	0.796	0.8	0.802	0.802	0.803	0.804	0.805	0.806	0.809	0.812	0.816	0.821	0.826	0.831	0.833	0.834	0.835
Bradford	0.669	0.675	0.681	0.686	0.69	0.696	0.702	0.707	0.714	0.72	0.726	0.732	0.738	0.744	0.75	0.755	0.759	0.763	0.767	0.773	0.779	0.784	0.786	0.787	0.79	0.792	0.795	0.798	0.802	0.805	0.808	0.81	0.812	0.815	0.817	0.818	0.819
Kirklees	0.676	0.682	0.688	0.694	0.699	0.705	0.711	0.716	0.723	0.729	0.735	0.741	0.747	0.753	0.759	0.763	0.768	0.772	0.778	0.785	0.79	0.795	0.796	0.797	0.8	0.803	0.805	0.807	0.81	0.812	0.814	0.817	0.821	0.825	0.828	0.83	0.832
Leeds	0.707	0.713	0.719	0.724	0.729	0.734	0.739	0.743	0.75	0.756	0.763	0.771	0.778	0.785	0.791	0.796	0.802	0.807	0.814	0.822	0.83	0.836	0.842	0.847	0.852	0.857	0.861	0.865	0.869	0.869	0.869	0.868	0.869	0.872	0.874	0.876	0.878
Sheffield	0.702	0.707	0.713	0.718	0.723	0.727	0.732	0.737	0.743	0.748	0.753	0.759	0.764	0.77	0.776	0.781	0.786	0.792	0.798	0.805	0.812	0.818	0.823	0.826	0.831	0.835	0.838	0.843	0.848	0.851	0.853	0.855	0.857	0.861	0.863	0.865	0.867
Wakefield	0.68	0.685	0.691	0.696	0.701	0.706	0.711	0.716	0.723	0.728	0.733	0.738	0.744	0.75	0.756	0.761	0.766	0.771	0.777	0.784	0.792	0.797	0.8	0.801	0.804	0.806	0.807	0.808	0.809	0.81	0.812	0.815	0.82	0.825	0.828	0.831	0.833
Rotherham	0.667	0.673	0.678	0.684	0.689	0.694	0.699	0.704	0.71	0.715	0.72	0.725	0.731	0.737	0.743	0.749	0.753	0.758	0.763	0.769	0.776	0.781	0.785	0.788	0.791	0.794	0.795	0.797	0.799	0.801	0.804	0.807	0.809	0.813	0.816	0.818	0.82
Doncaster	0.663	0.668	0.674	0.679	0.683	0.689	0.694	0.7	0.707	0.712	0.716	0.721	0.725	0.73	0.736	0.741	0.745	0.75	0.756	0.763	0.769	0.774	0.777	0.779	0.782	0.785	0.787	0.79	0.793	0.796	0.801	0.805	0.809	0.813	0.816	0.818	0.819
Kingston upon Hull, City of	0.675	0.68	0.685	0.688	0.69	0.694	0.7	0.709	0.718	0.721	0.725	0.731	0.74	0.747	0.754	0.759	0.764	0.77	0.776	0.783	0.791	0.797	0.8	0.803	0.808	0.812	0.817	0.821	0.825	0.826	0.825	0.825	0.827	0.831	0.835	0.838	0.84
Barnsley	0.66	0.665	0.671	0.676	0.681	0.687	0.692	0.697	0.703	0.708	0.712	0.718	0.724	0.73	0.737	0.742	0.747	0.753	0.76	0.766	0.772	0.776	0.778	0.78	0.782	0.784	0.786	0.789	0.791	0.792	0.794	0.797	0.801	0.806	0.81	0.812	0.814
Northamptonshire	0.698	0.704	0.71	0.716	0.72	0.725	0.73	0.734	0.74	0.746	0.751	0.757	0.764	0.771	0.777	0.783	0.788	0.793	0.799	0.805	0.811	0.816	0.818	0.819	0.821	0.824	0.825	0.827	0.828	0.83	0.833	0.835	0.839	0.843	0.845	0.845	0.846
Leicestershire	0.705	0.711	0.717	0.723	0.728	0.733	0.739	0.744	0.75	0.756	0.76	0.765	0.77	0.776	0.782	0.787	0.792	0.798	0.804	0.81	0.816	0.821	0.823	0.826	0.829	0.833	0.836	0.839	0.842	0.843	0.845	0.847	0.85	0.854	0.857	0.859	0.861
Lincolnshire	0.687	0.693	0.699	0.705	0.71	0.716	0.722	0.727	0.733	0.738	0.741	0.744	0.749	0.755	0.761	0.768	0.773	0.779	0.784	0.79	0.796	0.802	0.805	0.808	0.81	0.811	0.811	0.811	0.812	0.813	0.816	0.819	0.823	0.828	0.831	0.832	0.833
Rutland	0.703	0.709	0.715	0.721	0.728	0.736	0.744	0.751	0.76	0.765	0.771	0.772	0.776	0.784	0.79	0.796	0.801	0.806	0.811	0.815	0.82	0.814	0.802	0.782	0.776	0.788	0.797	0.804	0.809	0.817	0.831	0.839	0.839	0.839	0.838	0.839	0.84
Derby	0.689	0.696	0.703	0.71	0.716	0.723	0.728	0.733	0.738	0.743	0.748	0.756	0.764	0.773	0.782	0.789	0.794	0.798	0.803	0.81	0.816	0.822	0.826	0.829	0.834	0.837	0.838	0.841	0.844	0.845	0.846	0.846	0.849	0.855	0.858	0.861	0.864
Derbyshire	0.683	0.689	0.695	0.7	0.705	0.711	0.716	0.722	0.728	0.733	0.737	0.742	0.747	0.754	0.76	0.766	0.772	0.777	0.784	0.79	0.796	0.801	0.803	0.805	0.807	0.81	0.812	0.814	0.815	0.816	0.818	0.822	0.826	0.832	0.836	0.839	0.841
Nottinghamshire	0.68	0.686	0.692	0.698	0.703	0.709	0.714	0.719	0.725	0.731	0.735	0.74	0.745	0.751	0.757	0.762	0.766	0.771	0.778	0.786	0.794	0.8	0.805	0.807	0.811	0.814	0.816	0.817	0.819	0.819	0.82	0.821	0.824	0.828	0.831	0.834	0.835
Nottingham	0.705	0.711	0.718	0.724	0.729	0.733	0.737	0.741	0.746	0.753	0.76	0.769	0.777	0.785	0.793	0.801	0.807	0.815	0.822	0.828	0.834	0.84	0.846	0.85	0.855	0.86	0.863	0.867	0.87	0.871	0.871	0.87	0.871	0.873	0.875	0.877	0.878
Leicester	0.674	0.68	0.686	0.692	0.696	0.702	0.707	0.711	0.717	0.724	0.733	0.744	0.753	0.761	0.768	0.774	0.778	0.781	0.786	0.793	0.8	0.807	0.811	0.814	0.818	0.822	0.825	0.828	0.831	0.834	0.836	0.837	0.84	0.843	0.845	0.847	0.849
Warwickshire	0.714	0.72	0.725	0.731	0.735	0.741	0.746	0.75	0.756	0.761	0.766	0.772	0.778	0.784	0.791	0.796	0.801	0.806	0.812	0.819	0.825	0.83	0.833	0.836	0.838	0.84	0.841	0.842	0.844	0.846	0.849	0.852	0.856	0.861	0.863	0.864	0.865
Herefordshire, County of	0.692	0.698	0.704	0.709	0.715	0.72	0.725	0.729	0.733	0.738	0.741	0.746	0.752	0.759	0.765	0.77	0.775	0.78	0.786	0.793	0.801	0.807	0.809	0.811	0.813	0.815	0.815	0.814	0.815	0.816	0.821	0.828	0.836	0.842	0.846	0.848	0.85
Solihull	0.723	0.728	0.733	0.738	0.742	0.746	0.751	0.755	0.761	0.765	0.768	0.773	0.779	0.787	0.794	0.801	0.807	0.814	0.82	0.826	0.83	0.834	0.836	0.839	0.842	0.846	0.849	0.851	0.852	0.851	0.852	0.853	0.857	0.861	0.863	0.864	0.864
Shropshire	0.694	0.7	0.705	0.71	0.716	0.722	0.727	0.733	0.738	0.743	0.746	0.75	0.755	0.761	0.768	0.773	0.778	0.783	0.79	0.796	0.802	0.805	0.808	0.81	0.814	0.817	0.818	0.818	0.82	0.824	0.829	0.834	0.839	0.843	0.845	0.846	0.847
Worcestershire	0.688	0.694	0.699	0.705	0.71	0.716	0.721	0.727	0.733	0.738	0.742	0.747	0.753	0.759	0.765	0.77	0.775	0.78	0.787	0.796	0.804	0.81	0.813	0.815	0.819	0.821	0.822	0.823	0.824	0.825	0.828	0.832	0.836	0.841	0.844	0.846	0.847
Staffordshire	0.69	0.695	0.701	0.706	0.711	0.716	0.721	0.727	0.733	0.739	0.744	0.749	0.756	0.762	0.768	0.773	0.778	0.783	0.79	0.796	0.802	0.806	0.809	0.81	0.813	0.816	0.818	0.821	0.824	0.826	0.829	0.832	0.835	0.839	0.841	0.842	0.843

Appendix Table 7: Socio-demographic Index values for all estimated GBD 2016 locations, 1980–2016

Location	1980	1981	1982	1983	1984	1985	1986	1987	1988	1989	1990	1991	1992	1993	1994	1995	1996	1997	1998	1999	2000	2001	2002	2003	2004	2005	2006	2007	2008	2009	2010	2011	2012	2013	2014	2015	2016
Dudley	0.677	0.682	0.688	0.693	0.698	0.703	0.708	0.714	0.721	0.726	0.731	0.736	0.741	0.747	0.753	0.757	0.762	0.767	0.772	0.778	0.784	0.789	0.792	0.794	0.797	0.8	0.802	0.804	0.806	0.806	0.807	0.808	0.81	0.814	0.816	0.817	0.818
Coventry	0.697	0.702	0.707	0.711	0.715	0.719	0.724	0.729	0.735	0.742	0.75	0.757	0.764	0.771	0.777	0.782	0.787	0.792	0.797	0.805	0.812	0.819	0.823	0.826	0.83	0.833	0.835	0.836	0.837	0.838	0.84	0.843	0.848	0.855	0.86	0.863	0.866
Telford and Wrekin	0.698	0.704	0.71	0.716	0.721	0.726	0.731	0.736	0.742	0.747	0.752	0.757	0.763	0.769	0.775	0.78	0.785	0.792	0.798	0.804	0.81	0.815	0.817	0.817	0.818	0.818	0.818	0.818	0.821	0.822	0.824	0.828	0.833	0.838	0.842	0.845	0.846
Stoke-on-Trent	0.67	0.676	0.681	0.686	0.691	0.697	0.701	0.705	0.71	0.716	0.724	0.733	0.742	0.749	0.756	0.761	0.766	0.772	0.777	0.783	0.788	0.79	0.789	0.788	0.789	0.791	0.791	0.79	0.793	0.796	0.8	0.804	0.809	0.815	0.819	0.823	0.827
Walsall	0.667	0.672	0.677	0.682	0.686	0.691	0.697	0.702	0.709	0.714	0.718	0.722	0.728	0.735	0.741	0.744	0.746	0.747	0.752	0.759	0.766	0.771	0.773	0.773	0.776	0.779	0.781	0.784	0.786	0.788	0.792	0.796	0.8	0.804	0.807	0.808	0.809
Wolverhampton	0.678	0.684	0.689	0.693	0.698	0.703	0.707	0.712	0.717	0.723	0.728	0.734	0.741	0.747	0.753	0.757	0.761	0.766	0.772	0.78	0.788	0.795	0.799	0.8	0.803	0.806	0.807	0.808	0.809	0.81	0.813	0.816	0.821	0.825	0.827	0.829	0.83
Birmingham	0.68	0.686	0.691	0.696	0.7	0.705	0.71	0.714	0.72	0.727	0.733	0.742	0.75	0.757	0.763	0.767	0.771	0.775	0.781	0.788	0.795	0.801	0.805	0.808	0.812	0.817	0.819	0.822	0.826	0.829	0.832	0.833	0.836	0.84	0.843	0.845	0.847
Sandwell	0.666	0.671	0.677	0.682	0.686	0.691	0.696	0.701	0.708	0.714	0.719	0.725	0.732	0.739	0.745	0.749	0.753	0.758	0.763	0.768	0.773	0.778	0.781	0.782	0.785	0.787	0.788	0.789	0.79	0.791	0.794	0.797	0.803	0.808	0.812	0.813	0.815
Bedford	0.711	0.717	0.723	0.728	0.733	0.739	0.745	0.75	0.757	0.764	0.771	0.776	0.781	0.784	0.788	0.791	0.795	0.801	0.807	0.814	0.822	0.828	0.831	0.833	0.836	0.839	0.841	0.842	0.843	0.844	0.845	0.846	0.848	0.85	0.85	0.851	0.851
Central Bedfordshire	0.709	0.715	0.72	0.725	0.73	0.735	0.741	0.747	0.754	0.759	0.765	0.77	0.775	0.781	0.788	0.794	0.8	0.806	0.811	0.815	0.818	0.82	0.822	0.823	0.827	0.829	0.83	0.831	0.831	0.831	0.832	0.834	0.838	0.844	0.848	0.85	0.853
Suffolk	0.703	0.709	0.715	0.72	0.725	0.731	0.736	0.74	0.746	0.752	0.757	0.763	0.768	0.773	0.78	0.786	0.791	0.797	0.802	0.807	0.811	0.814	0.814	0.814	0.817	0.82	0.822	0.823	0.824	0.825	0.827	0.83	0.833	0.837	0.838	0.839	0.84
Hertfordshire	0.731	0.737	0.743	0.748	0.753	0.757	0.76	0.764	0.769	0.775	0.781	0.788	0.795	0.801	0.806	0.812	0.816	0.822	0.828	0.836	0.843	0.848	0.851	0.853	0.855	0.858	0.858	0.859	0.861	0.862	0.864	0.866	0.869	0.874	0.876	0.878	0.879
Essex	0.701	0.706	0.712	0.717	0.722	0.726	0.73	0.734	0.739	0.745	0.75	0.757	0.763	0.768	0.774	0.779	0.784	0.79	0.796	0.802	0.808	0.812	0.814	0.816	0.819	0.823	0.825	0.828	0.83	0.83	0.831	0.833	0.836	0.84	0.842	0.844	0.845
Cambridgeshire	0.728	0.734	0.74	0.745	0.749	0.755	0.76	0.765	0.771	0.777	0.783	0.789	0.796	0.801	0.807	0.814	0.82	0.827	0.833	0.838	0.844	0.849	0.854	0.856	0.86	0.862	0.862	0.862	0.862	0.863	0.867	0.87	0.874	0.878	0.88	0.881	0.882
Thurrock	0.702	0.708	0.711	0.715	0.717	0.72	0.723	0.726	0.733	0.741	0.75	0.759	0.765	0.77	0.775	0.78	0.783	0.787	0.793	0.799	0.806	0.811	0.812	0.812	0.813	0.813	0.813	0.814	0.816	0.819	0.821	0.822	0.823	0.825	0.826	0.825	0.824
Norfolk	0.702	0.708	0.714	0.719	0.723	0.728	0.733	0.737	0.743	0.749	0.754	0.759	0.764	0.77	0.775	0.781	0.786	0.792	0.798	0.804	0.81	0.814	0.817	0.818	0.821	0.824	0.826	0.828	0.83	0.831	0.832	0.833	0.836	0.841	0.844	0.846	0.848
Southend-on-Sea	0.683	0.688	0.693	0.696	0.699	0.703	0.709	0.716	0.724	0.731	0.736	0.742	0.749	0.757	0.765	0.772	0.778	0.785	0.79	0.794	0.796	0.797	0.798	0.799	0.802	0.804	0.804	0.806	0.809	0.813	0.817	0.819	0.822	0.825	0.827	0.827	0.828
Peterborough	0.713	0.719	0.724	0.728	0.732	0.737	0.742	0.747	0.753	0.759	0.763	0.767	0.773	0.778	0.785	0.79	0.794	0.798	0.803	0.809	0.815	0.819	0.818	0.816	0.816	0.816	0.816	0.816	0.816	0.818	0.823	0.828	0.831	0.835	0.837	0.838	0.839
Luton	0.692	0.698	0.703	0.707	0.71	0.713	0.715	0.717	0.725	0.733	0.743	0.752	0.759	0.765	0.771	0.775	0.779	0.783	0.787	0.793	0.798	0.803	0.804	0.804	0.806	0.808	0.811	0.816	0.823	0.829	0.834	0.837	0.84	0.843	0.844	0.844	0.845
Richmond upon Thames	0.76	0.766	0.771	0.775	0.779	0.783	0.787	0.791	0.798	0.805	0.813	0.82	0.827	0.833	0.839	0.845	0.85	0.855	0.861	0.868	0.873	0.878	0.882	0.885	0.887	0.888	0.887	0.887	0.888	0.888	0.889	0.891	0.895	0.9	0.905	0.908	0.91
Kensington and Chelsea	0.798	0.804	0.809	0.815	0.82	0.826	0.832	0.837	0.84	0.848	0.855	0.861	0.866	0.871	0.875	0.88	0.884	0.889	0.894	0.9	0.906	0.91	0.914	0.917	0.921	0.924	0.927	0.929	0.932	0.934	0.936	0.938	0.94	0.941	0.942	0.943	0.945
Barnet	0.729	0.735	0.742	0.748	0.753	0.759	0.764	0.769	0.775	0.782	0.787	0.792	0.797	0.803	0.81	0.816	0.823	0.829	0.835	0.841	0.846	0.851	0.853	0.854	0.855	0.855	0.854	0.853	0.855	0.857	0.86	0.865	0.87	0.875	0.878	0.879	0.88
Westminster	0.808	0.812	0.817	0.821	0.825	0.829	0.831	0.832	0.837	0.844	0.853	0.86	0.865	0.869	0.873	0.877	0.882	0.886	0.891	0.896	0.901	0.906	0.91	0.914	0.918	0.921	0.924	0.927	0.929	0.931	0.933	0.935	0.936	0.938	0.939	0.941	0.943
Bromley	0.73	0.736	0.741	0.746	0.749	0.753	0.757	0.761	0.767	0.774	0.779	0.784	0.789	0.793	0.799	0.804	0.809	0.815	0.821	0.829	0.835	0.839	0.842	0.844	0.846	0.847	0.848	0.85	0.851	0.851	0.851	0.851	0.853	0.857	0.86	0.862	0.863
Bexley	0.701	0.707	0.713	0.718	0.722	0.726	0.73	0.733	0.739	0.744	0.749	0.756	0.762	0.768	0.774	0.778	0.781	0.784	0.789	0.795	0.801	0.806	0.809	0.811	0.814	0.815	0.815	0.817	0.819	0.821	0.823	0.824	0.828	0.833	0.836	0.838	0.84
Redbridge	0.702	0.708	0.713	0.718	0.723	0.729	0.734	0.74	0.746	0.752	0.757	0.761	0.767	0.772	0.777	0.782	0.787	0.792	0.797	0.804	0.81	0.815	0.818	0.82	0.822	0.823	0.824	0.827	0.83	0.83	0.831	0.831	0.834	0.837	0.84	0.841	0.843
Merton	0.726	0.732	0.737	0.74	0.743	0.747	0.752	0.757	0.765	0.773	0.782	0.792	0.8	0.807	0.813	0.818	0.823	0.828	0.836	0.845	0.852	0.857	0.859	0.861	0.863	0.865	0.866	0.867	0.869	0.87	0.87	0.871	0.874	0.879	0.881	0.882	0.884
Brent	0.724	0.73	0.736	0.741	0.746	0.752	0.759	0.765	0.773	0.779	0.784	0.788	0.792	0.797	0.802	0.807	0.811	0.815	0.82	0.826	0.834	0.841	0.845	0.846	0.847	0.846	0.843	0.836	0.832	0.832	0.84	0.85	0.858	0.864	0.866	0.865	0.865
Hillingdon	0.756	0.762	0.767	0.772	0.776	0.78	0.784	0.788	0.795	0.801	0.807	0.814	0.821	0.827	0.834	0.84	0.844	0.848	0.853	0.859	0.864	0.866	0.868	0.869	0.871	0.871	0.87	0.87	0.872	0.874	0.876	0.878	0.882	0.888	0.892	0.895	0.897
Havering	0.703	0.709	0.715	0.72	0.724	0.728	0.732	0.735	0.74	0.745	0.75	0.756	0.762	0.768	0.774	0.778	0.783	0.787	0.792	0.798	0.803	0.808	0.81	0.812	0.815	0.818	0.82	0.822	0.825	0.827	0.828	0.829	0.83	0.833	0.835	0.836	0.837
Kingston upon Thames	0.746	0.752	0.758	0.762	0.766	0.77	0.774	0.779	0.788	0.796	0.804	0.811	0.818	0.823	0.828	0.832	0.837	0.842	0.85	0.858	0.864	0.869	0.874	0.878	0.881	0.884	0.886	0.888	0.891	0.892	0.892	0.892	0.895	0.898	0.9	0.901	0.902
Sutton	0.714	0.72	0.726	0.73	0.734	0.737	0.739	0.743	0.749	0.756	0.762	0.769	0.776	0.783	0.79	0.796	0.802	0.807	0.814	0.821	0.828	0.832	0.834	0.834	0.836	0.838	0.84	0.842	0.845	0.846	0.847	0.847	0.85	0.854	0.856	0.857	0.858
Harrow	0.719	0.726	0.732	0.737	0.741	0.745	0.749	0.751	0.757	0.764	0.772	0.781	0.789	0.796	0.802	0.806	0.81	0.814	0.821	0.829	0.836	0.84	0.841	0.842	0.844	0.847	0.847	0.848	0.849	0.849	0.851	0.852	0.854	0.856	0.856	0.856	0.856
Enfield	0.716	0.722	0.728	0.732	0.736	0.741	0.745	0.749	0.755	0.761	0.767	0.773	0.778	0.783	0.787	0.79	0.793	0.798	0.804	0.812	0.82	0.826	0.828	0.828	0.828	0.827	0.826	0.826	0.828	0.831	0.837	0.842	0.847	0.851	0.853	0.853	0.854
Croydon	0.721	0.727	0.732	0.737	0.741	0.745	0.749	0.753	0.759	0.765	0.772	0.78	0.787	0.794	0.801	0.806	0.81	0.815	0.82	0.826	0.832	0.836	0.839	0.841	0.843	0.843	0.841	0.84	0.841	0.841	0.843	0.845	0.848	0.85	0.85	0.849	0.849
Hammersmith and Fulham	0.784	0.789	0.795	0.801	0.807	0.814	0.82	0.826	0.833	0.841	0.849	0.856	0.862	0.867	0.872	0.877	0.883	0.887	0.891	0.897	0.901	0.906	0.909	0.912	0.916	0.919	0.92	0.922	0.924	0.927	0.932	0.936	0.937	0.938	0.94	0.941	0.942
Ealing	0.729	0.736	0.743	0.749	0.757	0.765	0.773	0.779	0.786	0.791	0.795	0.8	0.805	0.81	0.816	0.822	0.827	0.833	0.839	0.847	0.853	0.858	0.86	0.86	0.861	0.86	0.859	0.858	0.857	0.857	0.859	0.863	0.868	0.873	0.876	0.877	0.879
Greenwich	0.701	0.707	0.713	0.719	0.723	0.727	0.732	0.735	0.74	0.746	0.753	0.76	0.768	0.775	0.78	0.784	0.788	0.793	0.798	0.806	0.814	0.821	0.825	0.826	0.826	0.824	0.821	0.82	0.822	0.825	0.83	0.835	0.841	0.846	0.848	0.848	0.848
Wandsworth	0.746	0.752	0.758	0.764	0.77	0.777	0.784	0.791	0.799	0.808	0.816	0.825	0.833	0.839	0.844	0.849	0.854	0.86	0.865	0.872	0.879	0.885	0.89	0.895	0.899	0.902	0.906	0.907	0.909	0.911	0.912	0.915	0.919	0.922	0.924	0.926	0.928
Waltham Forest	0.681	0.687	0.694	0.699	0.705	0.71	0.715	0.72	0.726	0.733	0.739	0.746	0.753	0.76	0.766	0.771	0.774	0.777	0.783	0.79	0.797	0.803	0.806	0.806	0.807	0.805	0.802	0.798	0.798	0.8	0.807	0.814	0.822	0.828	0.832	0.834	0.835
Camden	0.81	0.815	0.819	0.823	0.828	0.833	0.838	0.843	0.848	0.852	0.857	0.862	0.867	0.872	0.876	0.881	0.883	0.884	0.888	0.897	0.904	0.909	0.912	0.915	0.919	0.922	0.924	0.927	0.929	0.931	0.933	0.935	0.937	0.939	0.94	0.942	0.943
Lambeth	0.738	0.745	0.751	0.756	0.761	0.766	0.771	0.773	0.779	0.787	0.798	0.811	0.821	0.828	0.834	0.838	0.842	0.847	0.853	0.86	0.867	0.871	0.872	0.873	0.876	0.881	0.885	0.891	0.898	0.903	0.908	0.914	0.919	0.921	0.922	0.924	0.926
Lewisham	0.702	0.708	0.714	0.719	0.724	0.728	0.732	0.736	0.741	0.747	0.755	0.766	0.775	0.782	0.787	0.79	0.793	0.796	0.802	0.81	0.819	0.826	0.828	0.828	0.829	0.83	0.832	0.833	0.835	0.836	0.84	0.844	0.85	0.856	0.86	0.863	0.865
Hounslow	0.745	0.75	0.753	0.755	0.755	0.757	0.761	0.766	0.773	0.784	0.795	0.805	0.81	0.815	0.818	0.822	0.825	0.829	0.836	0.847	0.857	0.864	0.866	0.864	0.864	0.865	0.867	0.869	0.872	0.874	0.876	0.878	0.883	0.889	0.891	0.891	0.892
Southwark	0.753	0.76	0.766	0.772	0.778	0.782	0.787	0.791	0.798	0.806	0.816	0.825	0.833	0.838	0.841	0.844	0.845	0.848	0.854	0.864	0.875	0.883	0.885	0.885	0.888	0.892	0.896	0.9	0.904	0.907	0.911	0.916	0.923	0.929	0.933	0.935	0.937
Newham	0.69	0.696	0.701	0.705	0.708	0.712	0.716	0.722	0.728	0.734	0.74	0.747	0.754	0.759	0.76	0.759	0.758	0.76	0.765	0.775	0.79	0.803	0.808	0.808	0.81	0.811	0.809	0.803	0.795	0.795	0.813	0.833	0.844	0.85	0.852	0.853	0.853
Barking and Dagenham	0.692	0.698	0.702	0.705	0.707	0.711	0.715	0.719	0.724	0.728	0.733	0.739	0.745	0.752	0.758	0.763	0.765	0.768	0.775	0.784	0.793	0.799	0.801	0.798	0.794	0.79	0.787	0.787	0.791	0.794	0.798	0.8	0.804	0.81	0.814	0.815	0.815
Haringey	0.73	0.736	0.741	0.745	0.747	0.748	0.75	0.752	0.758	0.765	0.777	0.79	0.799	0.805	0.809	0.813	0.817	0.821	0.825	0.831	0.837	0.843	0.846	0.847	0.849	0.851	0.85	0.849	0.848	0.848	0.855	0.865	0.872	0.878	0.88	0.88	0.88
Hackney	0.755	0.761	0.766	0.771	0.777	0.782	0.784	0.787	0.794	0.8	0.806	0.814	0.822	0.828	0.832	0.832	0.829	0.829	0.834	0.844	0.855	0.863	0.866	0.866	0.868	0.871	0.875	0.881	0.887	0.894	0.902	0.91	0.915	0.92	0.923	0.923	0.924
Islington	0.786	0.792	0.797	0.802	0.807	0.813	0.817	0.821	0.827	0.831	0.837	0.844	0.851	0.858	0.864	0.869	0.873	0.877	0.882	0.888	0.894	0.898	0.902	0.905	0.909	0.912	0.915	0.919	0.922	0.925	0.927	0.93	0.932	0.934	0.936	0.938	0.94
Tower Hamlets	0.727	0.732	0.736	0.74	0.747	0.757	0.768	0.777	0.785	0.79	0.794	0.797	0.801	0.805	0.809	0.813	0.819	0.826	0.834	0.847	0.859	0.869	0.876	0.88	0.885	0.891	0.896	0.902	0.907	0.912	0.916	0.919	0.924	0.927	0.929	0.931	0.933
Wokingham	0.758	0.763	0.768	0.773	0.778	0.784	0.79	0.796	0.803	0.806	0.809	0.81	0.812	0.815	0.817	0.82	0.824	0.829	0.836	0.846	0.857	0.865	0.87	0.872	0.875	0.879	0.881	0.883	0.884	0.883	0.882	0.88	0.882	0.887	0.891	0.893	0.895
Buckinghamshire	0.74	0.746	0.752	0.758	0.763	0.768	0.773	0.776	0.781	0.786	0.79	0.795	0.801	0.806	0.812	0.818	0.823	0.828	0.834	0.839	0.845	0.849	0.85	0.851	0.854	0.856	0.856	0.856	0.856	0.856	0.858	0.861	0.865	0.869	0.872	0.873	0.874
Surrey	0.749	0.755	0.76	0.764	0.767	0.77	0.773	0.775	0.781	0.787	0.793	0.8	0.806	0.811	0.816	0.821	0.825	0.831	0.837	0.846	0.854	0.859	0.861	0.863	0.865	0.866	0.867	0.868	0.869	0.87	0.871	0.872	0.875	0.88	0.882	0.884	0.886
Windsor and Maidenhead	0.76	0.766	0.77	0.774	0.776	0.779	0.782	0.783	0.786	0.79	0.799	0.809	0.817	0.824	0.83	0.837	0.841	0.846	0.85	0.856	0.861	0.865	0.868	0.872	0.876	0.879	0.88	0.881	0.883	0.884	0.885	0.888	0.891	0.896	0.9	0.903	0.905
West Berkshire	0.755	0.76	0.765	0.77	0.774	0.78	0.786	0.791	0.798	0.803	0.809	0.814	0.82	0.827	0.833	0.838	0.842	0.846	0.851	0.858	0.865	0.868	0.869	0.869	0.869	0.869	0.866	0.863	0.862	0.863	0.868	0.873	0.878	0.883	0.885	0.886	0.887
Hampshire	0.725	0.731	0.737	0.742	0.747	0.752	0.756	0.76	0.766	0.771	0.776	0.781	0.786	0.791	0.797	0.803	0.808	0.813	0.819	0.824	0.83	0.834	0.836	0.837	0.839	0.841	0.842	0.842	0.844	0.845	0.847	0.85	0.855	0.859	0.862	0.864	0.866

Appendix Table 7: Socio-demographic Index values for all estimated GBD 2016 locations, 1980–2016

Location	1980	1981	1982	1983	1984	1985	1986	1987	1988	1989	1990	1991	1992	1993	1994	1995	1996	1997	1998	1999	2000	2001	2002	2003	2004	2005	2006	2007	2008	2009	2010	2011	2012	2013	2014	2015	2016
Bracknell Forest	0.733	0.74	0.746	0.753	0.76	0.766	0.771	0.777	0.784	0.791	0.796	0.799	0.804	0.808	0.814	0.82	0.824	0.828	0.833	0.842	0.849	0.855	0.858	0.86	0.863	0.866	0.868	0.87	0.871	0.872	0.873	0.875	0.878	0.882	0.885	0.887	0.888
West Sussex	0.72	0.726	0.731	0.737	0.741	0.746	0.75	0.753	0.759	0.764	0.769	0.774	0.78	0.786	0.792	0.797	0.803	0.808	0.814	0.819	0.824	0.827	0.828	0.829	0.831	0.832	0.834	0.835	0.836	0.838	0.841	0.844	0.847	0.852	0.854	0.855	0.856
Oxfordshire	0.745	0.751	0.757	0.762	0.767	0.773	0.778	0.782	0.786	0.79	0.794	0.799	0.805	0.81	0.816	0.822	0.827	0.832	0.838	0.845	0.851	0.856	0.858	0.86	0.863	0.865	0.866	0.866	0.867	0.868	0.87	0.874	0.879	0.884	0.887	0.889	0.89
Reading	0.753	0.759	0.764	0.769	0.772	0.775	0.779	0.783	0.79	0.798	0.806	0.815	0.822	0.827	0.832	0.837	0.842	0.847	0.853	0.861	0.869	0.876	0.88	0.882	0.885	0.886	0.884	0.883	0.884	0.886	0.89	0.893	0.897	0.901	0.904	0.905	0.906
Kent	0.71	0.716	0.721	0.726	0.729	0.733	0.736	0.74	0.745	0.75	0.755	0.76	0.765	0.771	0.776	0.781	0.786	0.791	0.797	0.803	0.808	0.812	0.815	0.816	0.819	0.822	0.823	0.824	0.826	0.827	0.829	0.832	0.835	0.839	0.842	0.844	0.845
Brighton and Hove	0.73	0.735	0.74	0.743	0.745	0.748	0.751	0.755	0.763	0.771	0.781	0.79	0.797	0.803	0.808	0.813	0.819	0.824	0.829	0.834	0.839	0.845	0.851	0.857	0.862	0.867	0.872	0.874	0.877	0.88	0.884	0.887	0.889	0.891	0.894	0.896	0.899
Medway	0.686	0.691	0.697	0.701	0.706	0.71	0.715	0.72	0.727	0.733	0.738	0.744	0.749	0.753	0.758	0.763	0.769	0.774	0.78	0.785	0.791	0.796	0.799	0.801	0.804	0.807	0.81	0.811	0.813	0.813	0.815	0.816	0.82	0.824	0.827	0.829	0.831
East Sussex	0.696	0.702	0.708	0.713	0.718	0.723	0.728	0.732	0.737	0.742	0.746	0.75	0.755	0.759	0.765	0.77	0.776	0.781	0.786	0.791	0.796	0.799	0.8	0.801	0.803	0.806	0.807	0.808	0.809	0.81	0.813	0.817	0.821	0.826	0.83	0.832	0.834
Portsmouth	0.728	0.734	0.74	0.744	0.746	0.748	0.751	0.754	0.76	0.768	0.776	0.784	0.791	0.797	0.802	0.806	0.81	0.815	0.821	0.828	0.836	0.843	0.849	0.853	0.858	0.861	0.864	0.867	0.869	0.87	0.87	0.868	0.869	0.871	0.873	0.876	0.878
Isle of Wight	0.695	0.7	0.706	0.71	0.714	0.719	0.724	0.73	0.737	0.741	0.744	0.746	0.748	0.751	0.756	0.762	0.768	0.774	0.78	0.787	0.795	0.804	0.809	0.811	0.812	0.813	0.814	0.816	0.818	0.819	0.821	0.823	0.826	0.829	0.831	0.833	0.835
Milton Keynes	0.757	0.761	0.762	0.762	0.763	0.768	0.775	0.783	0.79	0.795	0.796	0.799	0.804	0.811	0.818	0.825	0.83	0.835	0.839	0.843	0.847	0.85	0.85	0.85	0.853	0.855	0.856	0.855	0.855	0.856	0.862	0.868	0.873	0.878	0.88	0.88	0.88
Southampton	0.728	0.734	0.739	0.743	0.746	0.748	0.749	0.75	0.754	0.761	0.771	0.782	0.79	0.798	0.804	0.81	0.814	0.819	0.825	0.832	0.84	0.845	0.849	0.854	0.858	0.863	0.866	0.868	0.869	0.868	0.867	0.866	0.867	0.871	0.874	0.876	0.878
Slough	0.749	0.753	0.756	0.757	0.759	0.762	0.766	0.771	0.779	0.787	0.795	0.805	0.812	0.818	0.824	0.83	0.836	0.84	0.844	0.847	0.852	0.855	0.854	0.851	0.848	0.846	0.843	0.845	0.85	0.855	0.861	0.866	0.871	0.874	0.875	0.874	0.873
South Gloucestershire	0.731	0.737	0.742	0.747	0.751	0.756	0.762	0.767	0.774	0.779	0.783	0.787	0.791	0.795	0.799	0.803	0.809	0.816	0.824	0.832	0.839	0.844	0.846	0.848	0.851	0.855	0.858	0.861	0.865	0.866	0.867	0.868	0.871	0.875	0.878	0.88	0.882
Dorset	0.704	0.71	0.715	0.721	0.726	0.731	0.737	0.742	0.747	0.752	0.754	0.757	0.762	0.768	0.775	0.781	0.788	0.794	0.801	0.807	0.811	0.813	0.814	0.813	0.815	0.816	0.815	0.814	0.814	0.816	0.821	0.827	0.833	0.837	0.839	0.841	0.842
Wiltshire	0.713	0.719	0.725	0.731	0.736	0.741	0.746	0.75	0.756	0.761	0.766	0.77	0.775	0.781	0.787	0.793	0.798	0.804	0.81	0.815	0.821	0.825	0.826	0.827	0.829	0.829	0.828	0.826	0.825	0.826	0.83	0.835	0.84	0.844	0.846	0.846	0.847
North Somerset	0.695	0.701	0.708	0.714	0.721	0.727	0.734	0.74	0.746	0.751	0.753	0.756	0.761	0.766	0.771	0.776	0.781	0.787	0.793	0.799	0.805	0.809	0.811	0.813	0.817	0.821	0.823	0.825	0.827	0.826	0.827	0.829	0.833	0.838	0.841	0.843	0.844
Devon	0.701	0.707	0.712	0.717	0.721	0.726	0.731	0.736	0.742	0.748	0.753	0.757	0.762	0.767	0.772	0.777	0.782	0.788	0.794	0.802	0.809	0.815	0.818	0.821	0.825	0.829	0.831	0.833	0.834	0.835	0.836	0.837	0.84	0.845	0.849	0.851	0.853
Poole	0.708	0.714	0.719	0.724	0.729	0.735	0.741	0.748	0.755	0.761	0.766	0.771	0.776	0.782	0.789	0.796	0.802	0.808	0.814	0.82	0.826	0.83	0.83	0.83	0.832	0.834	0.835	0.835	0.836	0.836	0.84	0.845	0.851	0.856	0.859	0.861	0.862
Bath and North East Somerset	0.722	0.727	0.732	0.737	0.74	0.745	0.75	0.756	0.762	0.768	0.772	0.777	0.782	0.787	0.793	0.798	0.803	0.808	0.816	0.824	0.83	0.837	0.843	0.849	0.855	0.86	0.863	0.867	0.87	0.871	0.871	0.872	0.874	0.878	0.882	0.884	0.886
Gloucestershire	0.716	0.722	0.728	0.733	0.738	0.744	0.749	0.754	0.76	0.765	0.77	0.775	0.779	0.785	0.79	0.796	0.801	0.806	0.812	0.819	0.826	0.832	0.835	0.838	0.842	0.844	0.844	0.844	0.845	0.846	0.849	0.851	0.855	0.859	0.861	0.862	0.863
Somerset	0.701	0.708	0.714	0.719	0.725	0.73	0.734	0.738	0.743	0.748	0.752	0.756	0.762	0.767	0.774	0.78	0.786	0.791	0.795	0.801	0.807	0.811	0.812	0.813	0.816	0.818	0.819	0.82	0.82	0.821	0.823	0.827	0.83	0.834	0.835	0.835	0.836
Swindon	0.736	0.742	0.748	0.753	0.757	0.761	0.766	0.77	0.777	0.784	0.79	0.796	0.801	0.806	0.81	0.815	0.819	0.824	0.831	0.838	0.844	0.849	0.851	0.851	0.851	0.852	0.851	0.853	0.855	0.856	0.858	0.859	0.861	0.865	0.866	0.866	0.867
Torbay	0.684	0.69	0.696	0.701	0.706	0.711	0.715	0.721	0.728	0.734	0.738	0.743	0.749	0.755	0.761	0.767	0.772	0.777	0.782	0.787	0.793	0.795	0.794	0.792	0.794	0.797	0.798	0.8	0.802	0.802	0.802	0.801	0.803	0.806	0.809	0.811	0.813
Bristol, City of	0.738	0.744	0.749	0.753	0.756	0.759	0.763	0.766	0.772	0.779	0.787	0.796	0.804	0.811	0.817	0.822	0.827	0.832	0.839	0.847	0.854	0.859	0.863	0.865	0.869	0.872	0.875	0.878	0.881	0.882	0.881	0.88	0.882	0.887	0.891	0.895	0.898
Bournemouth	0.705	0.71	0.715	0.72	0.726	0.732	0.737	0.742	0.747	0.752	0.759	0.767	0.775	0.78	0.786	0.791	0.797	0.802	0.808	0.814	0.82	0.826	0.831	0.837	0.841	0.847	0.846	0.846	0.847	0.849	0.853	0.858	0.862	0.866	0.869	0.871	0.872
Cornwall	0.685	0.691	0.697	0.702	0.708	0.713	0.718	0.723	0.729	0.735	0.739	0.743	0.748	0.754	0.76	0.766	0.771	0.776	0.782	0.789	0.796	0.801	0.805	0.808	0.812	0.815	0.818	0.819	0.821	0.821	0.822	0.823	0.826	0.83	0.832	0.833	0.835
Plymouth	0.699	0.705	0.711	0.718	0.723	0.729	0.736	0.741	0.747	0.752	0.757	0.762	0.769	0.776	0.783	0.789	0.793	0.798	0.804	0.81	0.815	0.819	0.822	0.823	0.827	0.83	0.833	0.836	0.839	0.84	0.84	0.841	0.843	0.847	0.85	0.851	0.853
North East England	0.681	0.687	0.692	0.698	0.702	0.708	0.714	0.72	0.727	0.732	0.737	0.743	0.749	0.755	0.762	0.768	0.773	0.779	0.785	0.792	0.798	0.804	0.807	0.809	0.813	0.816	0.819	0.822	0.824	0.826	0.828	0.83	0.834	0.838	0.841	0.842	0.844
North West England	0.693	0.699	0.704	0.709	0.714	0.719	0.724	0.729	0.735	0.741	0.746	0.752	0.759	0.766	0.773	0.778	0.783	0.788	0.794	0.801	0.807	0.813	0.816	0.818	0.821	0.823	0.825	0.826	0.828	0.83	0.833	0.836	0.84	0.845	0.848	0.85	0.851
Yorkshire and the Humber	0.69	0.695	0.701	0.706	0.711	0.716	0.721	0.726	0.733	0.738	0.743	0.749	0.755	0.761	0.767	0.773	0.777	0.783	0.789	0.795	0.802	0.808	0.811	0.813	0.816	0.82	0.822	0.825	0.828	0.83	0.832	0.834	0.837	0.841	0.843	0.845	0.846
East Midlands	0.691	0.697	0.703	0.709	0.714	0.719	0.725	0.73	0.736	0.741	0.746	0.751	0.758	0.764	0.771	0.777	0.782	0.787	0.793	0.8	0.806	0.811	0.815	0.817	0.82	0.823	0.824	0.826	0.828	0.83	0.832	0.834	0.837	0.841	0.844	0.846	0.848
West Midlands	0.688	0.694	0.699	0.704	0.709	0.714	0.719	0.724	0.73	0.736	0.741	0.747	0.754	0.76	0.766	0.771	0.776	0.781	0.787	0.793	0.8	0.805	0.808	0.81	0.813	0.816	0.817	0.819	0.821	0.823	0.826	0.829	0.833	0.837	0.84	0.842	0.844
East of England	0.711	0.716	0.722	0.727	0.732	0.736	0.741	0.745	0.751	0.757	0.762	0.769	0.775	0.78	0.786	0.791	0.797	0.802	0.808	0.815	0.82	0.824	0.826	0.828	0.831	0.833	0.835	0.836	0.838	0.839	0.841	0.843	0.846	0.85	0.853	0.854	0.855
Greater London	0.751	0.757	0.763	0.768	0.772	0.777	0.782	0.786	0.792	0.799	0.807	0.815	0.821	0.827	0.833	0.837	0.841	0.846	0.852	0.86	0.868	0.875	0.878	0.88	0.883	0.885	0.886	0.886	0.888	0.89	0.894	0.899	0.904	0.909	0.912	0.914	0.915
South East England	0.729	0.735	0.74	0.745	0.749	0.753	0.757	0.761	0.767	0.772	0.777	0.783	0.789	0.794	0.8	0.805	0.81	0.816	0.821	0.828	0.834	0.839	0.841	0.842	0.845	0.847	0.848	0.849	0.85	0.851	0.854	0.856	0.86	0.864	0.867	0.869	0.87
South West England	0.711	0.716	0.722	0.727	0.732	0.737	0.742	0.747	0.753	0.758	0.763	0.768	0.773	0.779	0.785	0.79	0.795	0.801	0.807	0.814	0.82	0.825	0.828	0.829	0.832	0.835	0.836	0.837	0.839	0.84	0.841	0.844	0.847	0.851	0.854	0.856	0.857
Southern Latin America	0.599	0.604	0.607	0.613	0.618	0.621	0.627	0.631	0.634	0.637	0.641	0.647	0.654	0.662	0.671	0.678	0.684	0.688	0.693	0.698	0.702	0.706	0.709	0.712	0.716	0.72	0.725	0.729	0.734	0.738	0.743	0.749	0.754	0.76	0.764	0.768	0.771
Argentina	0.595	0.6	0.602	0.608	0.613	0.617	0.624	0.629	0.634	0.636	0.639	0.645	0.652	0.66	0.67	0.677	0.681	0.684	0.687	0.691	0.695	0.699	0.701	0.703	0.706	0.71	0.714	0.719	0.723	0.727	0.732	0.739	0.745	0.75	0.754	0.758	0.761
Chile	0.603	0.606	0.613	0.619	0.623	0.627	0.63	0.631	0.633	0.638	0.645	0.652	0.66	0.668	0.677	0.685	0.694	0.702	0.71	0.716	0.722	0.727	0.732	0.737	0.744	0.749	0.754	0.759	0.763	0.767	0.772	0.777	0.782	0.787	0.792	0.796	0.8
Uruguay	0.61	0.615	0.619	0.622	0.624	0.626	0.629	0.632	0.635	0.638	0.641	0.642	0.645	0.648	0.652	0.656	0.661	0.667	0.673	0.678	0.682	0.685	0.688	0.69	0.693	0.696	0.7	0.704	0.709	0.714	0.719	0.724	0.729	0.734	0.739	0.743	0.747
High-income North America	0.836	0.839	0.842	0.845	0.847	0.849	0.851	0.852	0.852	0.851	0.852	0.855	0.858	0.86	0.863	0.865	0.867	0.868	0.87	0.871	0.873	0.876	0.878	0.878	0.88	0.88	0.88	0.882	0.885	0.89	0.893	0.895	0.896	0.897	0.898	0.899	0.9
Canada	0.815	0.82	0.823	0.826	0.83	0.834	0.839	0.843	0.845	0.845	0.847	0.849	0.853	0.855	0.858	0.862	0.867	0.87	0.873	0.878	0.881	0.884	0.887	0.889	0.891	0.892	0.892	0.892	0.894	0.897	0.899	0.901	0.903	0.904	0.905	0.907	0.908
USA	0.838	0.841	0.844	0.847	0.849	0.85	0.853	0.853	0.852	0.851	0.852	0.855	0.858	0.86	0.863	0.865	0.867	0.868	0.869	0.87	0.872	0.875	0.876	0.877	0.878	0.879	0.878	0.88	0.884	0.889	0.892	0.894	0.895	0.896	0.897	0.898	0.899
Greenland	0.733	0.739	0.745	0.75	0.752	0.754	0.755	0.755	0.754	0.752	0.75	0.75	0.749	0.749	0.75	0.75	0.75	0.753	0.758	0.762	0.763	0.764	0.766	0.771	0.775	0.779	0.783	0.786	0.788	0.792	0.798	0.804	0.808	0.811	0.813	0.814	0.816
Alabama	0.808	0.812	0.816	0.82	0.823	0.826	0.828	0.829	0.829	0.83	0.832	0.835	0.839	0.842	0.845	0.847	0.849	0.85	0.851	0.853	0.856	0.86	0.862	0.864	0.865	0.865	0.864	0.865	0.869	0.874	0.878	0.88	0.882	0.882	0.883	0.884	0.884
Alaska	0.849	0.844	0.843	0.844	0.843	0.846	0.85	0.851	0.85	0.847	0.845	0.846	0.848	0.852	0.856	0.859	0.861	0.862	0.863	0.863	0.863	0.863	0.863	0.864	0.868	0.871	0.872	0.874	0.878	0.881	0.885	0.887	0.89	0.891	0.894	0.896	0.897
Arizona	0.829	0.832	0.834	0.836	0.836	0.837	0.839	0.84	0.839	0.838	0.837	0.84	0.842	0.844	0.846	0.847	0.848	0.848	0.848	0.848	0.849	0.852	0.853	0.853	0.855	0.855	0.856	0.861	0.868	0.875	0.879	0.88	0.881	0.881	0.881	0.881	0.881
Arkansas	0.799	0.803	0.807	0.811	0.814	0.816	0.819	0.82	0.82	0.82	0.822	0.826	0.83	0.833	0.836	0.837	0.839	0.84	0.842	0.844	0.846	0.849	0.85	0.851	0.853	0.853	0.852	0.854	0.859	0.863	0.867	0.869	0.871	0.872	0.874	0.875	0.876
California	0.851	0.853	0.854	0.856	0.856	0.856	0.856	0.854	0.849	0.844	0.841	0.843	0.846	0.848	0.851	0.854	0.857	0.861	0.863	0.866	0.869	0.872	0.873	0.872	0.873	0.873	0.872	0.875	0.88	0.885	0.889	0.891	0.893	0.894	0.896	0.897	0.898
Colorado	0.859	0.861	0.863	0.865	0.865	0.866	0.869	0.87	0.87	0.869	0.87	0.873	0.875	0.878	0.881	0.883	0.884	0.883	0.883	0.882	0.883	0.885	0.887	0.888	0.891	0.892	0.893	0.895	0.899	0.903	0.906	0.908	0.91	0.912	0.913	0.914	0.915
Connecticut	0.87	0.873	0.875	0.878	0.879	0.881	0.883	0.885	0.886	0.887	0.889	0.892	0.895	0.897	0.899	0.9	0.902	0.903	0.904	0.905	0.907	0.91	0.911	0.911	0.912	0.912	0.912	0.913	0.917	0.921	0.924	0.926	0.928	0.929	0.93	0.93	0.93
Delaware	0.854	0.857	0.859	0.862	0.863	0.865	0.867	0.867	0.866	0.866	0.867	0.871	0.875	0.878	0.88	0.882	0.883	0.882	0.882	0.882	0.884	0.887	0.888	0.889	0.89	0.889	0.887	0.888	0.892	0.897	0.901	0.903	0.905	0.907	0.908	0.909	0.909
District of Columbia	0.881	0.883	0.884	0.886	0.887	0.888	0.889	0.887	0.882	0.876	0.874	0.877	0.883	0.89	0.898	0.905	0.911	0.917	0.921	0.924	0.927	0.929	0.93	0.927	0.926	0.925	0.925	0.925	0.928	0.932	0.935	0.938	0.941	0.944	0.946	0.947	0.947
Florida	0.843	0.846	0.847	0.849	0.85	0.851	0.853	0.853	0.852	0.851	0.854	0.857	0.86	0.863	0.865	0.867	0.869	0.87	0.87	0.871	0.873	0.875	0.876	0.877	0.878	0.878	0.878	0.881	0.886	0.892	0.895	0.897	0.898	0.898	0.898	0.898	0.898
Georgia	0.82	0.825	0.829	0.832	0.835	0.837	0.84	0.841	0.841	0.841	0.843	0.848	0.851	0.855	0.857	0.859	0.86	0.86	0.86	0.86	0.862	0.865	0.867	0.868	0.869	0.868	0.867	0.869	0.874	0.88	0.884	0.886	0.887	0.887	0.888	0.888	0.888
Hawaii	0.843	0.844	0.845	0.848	0.85	0.852	0.855	0.855	0.854	0.853	0.854	0.857	0.86	0.863	0.866	0.869	0.872	0.874	0.875	0.875	0.875	0.876	0.874	0.873	0.874	0.875	0.875	0.877	0.881	0.885	0.889	0.891	0.894	0.896	0.898	0.9	0.901

Appendix Table 7: Socio-demographic Index values for all estimated GBD 2016 locations, 1980–2016

Location	1980	1981	1982	1983	1984	1985	1986	1987	1988	1989	1990	1991	1992	1993	1994	1995	1996	1997	1998	1999	2000	2001	2002	2003	2004	2005	2006	2007	2008	2009	2010	2011	2012	2013	2014	2015	2016
Idaho	0.809	0.813	0.816	0.822	0.825	0.829	0.833	0.834	0.834	0.832	0.833	0.835	0.837	0.84	0.842	0.845	0.846	0.847	0.848	0.849	0.851	0.854	0.854	0.854	0.855	0.854	0.853	0.854	0.859	0.864	0.867	0.869	0.87	0.871	0.872	0.872	0.872
Illinois	0.843	0.846	0.849	0.852	0.854	0.856	0.857	0.858	0.856	0.855	0.856	0.858	0.86	0.863	0.866	0.868	0.871	0.872	0.874	0.875	0.878	0.881	0.883	0.884	0.886	0.888	0.888	0.891	0.895	0.899	0.902	0.903	0.905	0.906	0.907	0.908	0.908
Indiana	0.833	0.837	0.84	0.843	0.844	0.846	0.848	0.849	0.848	0.847	0.848	0.85	0.853	0.855	0.858	0.859	0.86	0.86	0.86	0.861	0.863	0.866	0.867	0.867	0.869	0.869	0.868	0.869	0.872	0.875	0.878	0.879	0.88	0.881	0.882	0.883	0.883
Iowa	0.834	0.838	0.842	0.845	0.848	0.85	0.853	0.854	0.854	0.853	0.854	0.856	0.858	0.86	0.862	0.864	0.865	0.866	0.867	0.868	0.87	0.873	0.874	0.875	0.876	0.876	0.874	0.876	0.88	0.884	0.887	0.888	0.89	0.891	0.891	0.892	0.893
Kansas	0.835	0.839	0.842	0.846	0.848	0.851	0.854	0.855	0.856	0.855	0.857	0.859	0.861	0.862	0.864	0.865	0.866	0.866	0.866	0.866	0.868	0.871	0.873	0.874	0.875	0.875	0.874	0.875	0.879	0.883	0.886	0.888	0.89	0.892	0.893	0.895	0.895
Kentucky	0.805	0.81	0.814	0.818	0.821	0.824	0.827	0.828	0.828	0.829	0.83	0.833	0.836	0.839	0.841	0.843	0.845	0.846	0.847	0.849	0.851	0.855	0.856	0.857	0.858	0.858	0.857	0.859	0.862	0.865	0.868	0.869	0.87	0.87	0.871	0.873	0.874
Louisiana	0.796	0.801	0.806	0.811	0.814	0.817	0.82	0.822	0.822	0.822	0.824	0.827	0.83	0.833	0.837	0.84	0.842	0.844	0.846	0.847	0.85	0.854	0.856	0.857	0.86	0.86	0.861	0.864	0.869	0.873	0.876	0.878	0.88	0.882	0.883	0.885	0.885
Maine	0.832	0.834	0.836	0.839	0.841	0.844	0.848	0.85	0.853	0.855	0.858	0.862	0.866	0.869	0.872	0.874	0.876	0.877	0.878	0.88	0.882	0.885	0.886	0.887	0.889	0.89	0.891	0.893	0.896	0.899	0.901	0.902	0.902	0.903	0.904	0.905	0.906
Maryland	0.865	0.867	0.868	0.87	0.871	0.872	0.875	0.875	0.875	0.875	0.877	0.88	0.883	0.886	0.889	0.891	0.892	0.892	0.892	0.893	0.895	0.897	0.899	0.899	0.901	0.902	0.901	0.903	0.907	0.912	0.915	0.917	0.919	0.92	0.92	0.921	0.92
Massachusetts	0.865	0.868	0.871	0.874	0.877	0.88	0.882	0.884	0.886	0.886	0.889	0.892	0.895	0.897	0.9	0.902	0.904	0.905	0.906	0.907	0.91	0.913	0.915	0.916	0.918	0.919	0.919	0.921	0.923	0.926	0.928	0.929	0.931	0.932	0.933	0.935	0.935
Michigan	0.845	0.847	0.849	0.852	0.853	0.855	0.857	0.857	0.856	0.854	0.855	0.858	0.861	0.864	0.867	0.87	0.871	0.872	0.873	0.875	0.877	0.88	0.882	0.883	0.886	0.887	0.887	0.888	0.89	0.891	0.892	0.893	0.893	0.894	0.895	0.896	0.897
Minnesota	0.839	0.844	0.848	0.852	0.856	0.859	0.863	0.866	0.867	0.868	0.87	0.873	0.875	0.877	0.879	0.88	0.882	0.882	0.883	0.884	0.886	0.889	0.89	0.89	0.891	0.891	0.89	0.892	0.896	0.9	0.903	0.904	0.905	0.906	0.907	0.909	0.909
Mississippi	0.784	0.789	0.794	0.799	0.803	0.806	0.81	0.811	0.811	0.811	0.813	0.816	0.82	0.824	0.828	0.831	0.833	0.835	0.836	0.838	0.841	0.845	0.847	0.848	0.848	0.847	0.846	0.848	0.853	0.859	0.864	0.867	0.869	0.871	0.872	0.873	0.874
Missouri	0.826	0.83	0.833	0.837	0.839	0.842	0.845	0.846	0.846	0.846	0.847	0.85	0.853	0.856	0.859	0.86	0.862	0.863	0.864	0.865	0.868	0.871	0.872	0.873	0.875	0.875	0.874	0.876	0.879	0.883	0.886	0.887	0.888	0.89	0.891	0.892	0.893
Montana	0.829	0.831	0.834	0.837	0.838	0.84	0.843	0.845	0.844	0.844	0.845	0.847	0.849	0.852	0.855	0.858	0.86	0.862	0.863	0.865	0.866	0.868	0.869	0.869	0.872	0.872	0.873	0.875	0.879	0.882	0.885	0.886	0.888	0.889	0.891	0.892	0.893
Nebraska	0.832	0.835	0.838	0.842	0.845	0.848	0.852	0.853	0.854	0.853	0.855	0.858	0.86	0.862	0.863	0.865	0.866	0.867	0.867	0.867	0.868	0.87	0.87	0.871	0.873	0.873	0.871	0.873	0.877	0.881	0.884	0.886	0.888	0.888	0.889	0.89	0.89
Nevada	0.855	0.857	0.858	0.86	0.86	0.86	0.86	0.857	0.854	0.85	0.85	0.853	0.855	0.857	0.858	0.859	0.861	0.862	0.862	0.863	0.865	0.868	0.868	0.868	0.869	0.867	0.865	0.868	0.875	0.882	0.887	0.889	0.889	0.889	0.888	0.887	0.886
New Hampshire	0.852	0.856	0.859	0.862	0.864	0.866	0.869	0.871	0.873	0.876	0.879	0.882	0.885	0.887	0.889	0.891	0.893	0.894	0.896	0.897	0.9	0.903	0.904	0.905	0.908	0.91	0.911	0.914	0.916	0.919	0.921	0.922	0.924	0.926	0.928	0.93	0.931
New Jersey	0.858	0.861	0.864	0.867	0.868	0.87	0.872	0.873	0.873	0.873	0.875	0.878	0.881	0.883	0.885	0.887	0.889	0.891	0.891	0.892	0.894	0.896	0.896	0.897	0.899	0.9	0.899	0.9	0.904	0.908	0.912	0.914	0.915	0.916	0.917	0.918	0.918
New Mexico	0.809	0.812	0.814	0.818	0.819	0.822	0.825	0.826	0.826	0.824	0.825	0.826	0.829	0.832	0.835	0.838	0.841	0.843	0.844	0.845	0.847	0.85	0.852	0.852	0.854	0.855	0.855	0.858	0.862	0.867	0.871	0.873	0.875	0.877	0.879	0.88	0.881
New York	0.854	0.856	0.858	0.861	0.862	0.864	0.866	0.866	0.866	0.866	0.868	0.871	0.874	0.877	0.879	0.882	0.885	0.887	0.888	0.89	0.892	0.895	0.896	0.897	0.899	0.899	0.899	0.901	0.904	0.907	0.91	0.912	0.915	0.916	0.918	0.919	0.92
North Carolina	0.825	0.829	0.832	0.835	0.837	0.84	0.842	0.842	0.842	0.841	0.844	0.848	0.851	0.855	0.858	0.86	0.86	0.86	0.86	0.86	0.861	0.865	0.867	0.868	0.869	0.868	0.866	0.868	0.873	0.878	0.883	0.885	0.886	0.886	0.887	0.887	0.887
North Dakota	0.816	0.818	0.822	0.827	0.83	0.835	0.839	0.842	0.842	0.843	0.846	0.848	0.85	0.852	0.854	0.857	0.86	0.863	0.866	0.869	0.871	0.874	0.874	0.875	0.875	0.874	0.872	0.874	0.879	0.883	0.887	0.888	0.891	0.892	0.893	0.895	0.896
Ohio	0.838	0.841	0.844	0.847	0.848	0.85	0.853	0.853	0.853	0.852	0.854	0.856	0.858	0.86	0.863	0.865	0.866	0.866	0.867	0.868	0.87	0.874	0.875	0.876	0.878	0.879	0.879	0.88	0.883	0.885	0.887	0.888	0.889	0.89	0.891	0.892	0.893
Oklahoma	0.82	0.824	0.828	0.833	0.836	0.84	0.843	0.844	0.843	0.842	0.843	0.844	0.846	0.847	0.848	0.849	0.849	0.849	0.85	0.85	0.853	0.856	0.857	0.858	0.86	0.861	0.861	0.862	0.866	0.869	0.872	0.874	0.877	0.879	0.88	0.882	0.883
Oregon	0.85	0.852	0.854	0.857	0.857	0.858	0.859	0.858	0.857	0.856	0.857	0.86	0.863	0.866	0.868	0.869	0.87	0.871	0.873	0.875	0.878	0.882	0.884	0.885	0.887	0.887	0.886	0.887	0.89	0.894	0.897	0.898	0.899	0.9	0.901	0.903	0.903
Pennsylvania	0.842	0.845	0.847	0.85	0.851	0.853	0.855	0.856	0.856	0.856	0.858	0.861	0.864	0.867	0.87	0.873	0.875	0.877	0.878	0.879	0.881	0.884	0.885	0.885	0.887	0.887	0.887	0.888	0.892	0.896	0.899	0.9	0.902	0.904	0.905	0.907	0.908
Rhode Island	0.847	0.851	0.853	0.857	0.859	0.861	0.863	0.864	0.865	0.865	0.867	0.87	0.873	0.877	0.88	0.883	0.885	0.886	0.887	0.889	0.89	0.893	0.894	0.895	0.898	0.9	0.9	0.902	0.906	0.909	0.912	0.913	0.915	0.916	0.918	0.92	0.921
South Carolina	0.814	0.818	0.821	0.825	0.828	0.83	0.833	0.833	0.832	0.831	0.834	0.839	0.844	0.849	0.853	0.855	0.856	0.857	0.857	0.858	0.86	0.864	0.866	0.866	0.866	0.864	0.862	0.863	0.868	0.874	0.879	0.881	0.883	0.884	0.884	0.885	0.886
South Dakota	0.806	0.81	0.814	0.818	0.822	0.825	0.83	0.832	0.832	0.832	0.834	0.836	0.839	0.842	0.846	0.849	0.852	0.853	0.855	0.857	0.859	0.861	0.861	0.862	0.863	0.862	0.861	0.863	0.868	0.872	0.876	0.877	0.878	0.879	0.88	0.881	0.882
Tennessee	0.818	0.822	0.825	0.829	0.831	0.833	0.836	0.836	0.836	0.835	0.837	0.841	0.844	0.848	0.851	0.853	0.855	0.856	0.857	0.858	0.861	0.864	0.866	0.866	0.867	0.867	0.865	0.867	0.871	0.875	0.879	0.88	0.882	0.882	0.883	0.884	0.885
Texas	0.815	0.819	0.823	0.827	0.829	0.831	0.834	0.834	0.834	0.832	0.832	0.834	0.835	0.837	0.839	0.84	0.842	0.843	0.843	0.844	0.846	0.849	0.85	0.851	0.853	0.853	0.853	0.855	0.86	0.866	0.87	0.873	0.875	0.876	0.877	0.878	0.878
Utah	0.779	0.786	0.794	0.803	0.809	0.814	0.819	0.82	0.82	0.819	0.822	0.827	0.831	0.833	0.834	0.835	0.834	0.834	0.834	0.836	0.839	0.843	0.846	0.847	0.849	0.849	0.848	0.85	0.855	0.859	0.863	0.864	0.866	0.867	0.869	0.87	0.87
Vermont	0.843	0.846	0.848	0.851	0.853	0.855	0.859	0.861	0.863	0.866	0.869	0.872	0.876	0.879	0.882	0.885	0.887	0.889	0.89	0.893	0.895	0.898	0.899	0.9	0.902	0.904	0.905	0.907	0.909	0.912	0.913	0.915	0.916	0.917	0.918	0.919	0.92
Virginia	0.849	0.852	0.855	0.858	0.86	0.862	0.864	0.866	0.866	0.867	0.868	0.871	0.874	0.877	0.88	0.882	0.883	0.883	0.884	0.884	0.885	0.888	0.888	0.888	0.89	0.891	0.891	0.894	0.899	0.903	0.907	0.909	0.91	0.911	0.912	0.913	0.913
Washington	0.857	0.859	0.862	0.865	0.866	0.867	0.869	0.869	0.868	0.867	0.868	0.87	0.873	0.876	0.878	0.88	0.882	0.883	0.885	0.887	0.891	0.894	0.896	0.896	0.898	0.897	0.896	0.897	0.899	0.902	0.904	0.906	0.907	0.908	0.91	0.911	0.912
West Virginia	0.809	0.813	0.818	0.822	0.824	0.827	0.83	0.831	0.831	0.83	0.832	0.834	0.837	0.84	0.843	0.845	0.847	0.849	0.85	0.851	0.852	0.854	0.856	0.857	0.859	0.859	0.859	0.859	0.862	0.865	0.867	0.869	0.87	0.872	0.873	0.875	0.876
Wisconsin	0.836	0.839	0.842	0.845	0.847	0.85	0.853	0.855	0.856	0.857	0.859	0.861	0.864	0.867	0.869	0.871	0.873	0.874	0.875	0.876	0.878	0.88	0.881	0.882	0.884	0.884	0.884	0.885	0.888	0.892	0.894	0.895	0.897	0.898	0.899	0.901	0.902
Wyoming	0.824	0.827	0.83	0.835	0.837	0.842	0.847	0.85	0.851	0.851	0.852	0.854	0.857	0.86	0.863	0.865	0.867	0.868	0.871	0.874	0.876	0.878	0.878	0.878	0.878	0.877	0.877	0.879	0.886	0.893	0.898	0.901	0.902	0.903	0.904	0.905	0.905
Latin America and Caribbean	0.467	0.477	0.486	0.494	0.503	0.511	0.519	0.526	0.533	0.54	0.546	0.554	0.561	0.568	0.575	0.581	0.588	0.595	0.602	0.608	0.615	0.621	0.627	0.632	0.638	0.643	0.649	0.655	0.661	0.666	0.672	0.678	0.684	0.689	0.695	0.7	0.705
Caribbean	0.532	0.538	0.543	0.551	0.557	0.563	0.569	0.573	0.578	0.583	0.588	0.594	0.599	0.602	0.605	0.608	0.61	0.613	0.617	0.621	0.626	0.631	0.635	0.64	0.645	0.651	0.656	0.66	0.663	0.667	0.671	0.675	0.679	0.683	0.688	0.692	0.696
Antigua and Barbuda	0.611	0.619	0.627	0.634	0.64	0.647	0.654	0.662	0.67	0.678	0.684	0.691	0.696	0.7	0.704	0.707	0.71	0.713	0.716	0.719	0.722	0.723	0.725	0.728	0.731	0.735	0.74	0.746	0.752	0.756	0.759	0.761	0.765	0.767	0.771	0.774	0.778
The Bahamas	0.659	0.666	0.674	0.681	0.689	0.697	0.704	0.709	0.713	0.716	0.719	0.72	0.721	0.722	0.724	0.728	0.733	0.739	0.745	0.751	0.757	0.761	0.765	0.767	0.769	0.77	0.771	0.773	0.774	0.775	0.775	0.776	0.777	0.779	0.78	0.781	0.783
Barbados	0.662	0.668	0.673	0.679	0.685	0.691	0.699	0.706	0.713	0.72	0.726	0.732	0.737	0.741	0.744	0.748	0.751	0.755	0.759	0.762	0.766	0.769	0.771	0.772	0.773	0.776	0.779	0.782	0.784	0.786	0.788	0.789	0.79	0.791	0.793	0.795	0.797
Belize	0.344	0.348	0.353	0.36	0.369	0.379	0.391	0.404	0.419	0.435	0.452	0.469	0.487	0.502	0.515	0.525	0.532	0.537	0.542	0.546	0.552	0.557	0.563	0.569	0.574	0.58	0.585	0.589	0.593	0.596	0.6	0.604	0.608	0.612	0.617	0.621	0.625
Cuba	0.631	0.634	0.637	0.645	0.65	0.658	0.666	0.668	0.673	0.679	0.685	0.693	0.698	0.697	0.696	0.695	0.695	0.692	0.693	0.694	0.697	0.701	0.705	0.712	0.719	0.725	0.732	0.739	0.739	0.743	0.747	0.751	0.757	0.761	0.766	0.772	0.778
Dominica	0.529	0.534	0.541	0.548	0.556	0.563	0.57	0.577	0.584	0.59	0.596	0.6	0.605	0.612	0.62	0.629	0.637	0.645	0.653	0.659	0.665	0.67	0.674	0.678	0.682	0.686	0.69	0.695	0.701	0.705	0.71	0.714	0.718	0.721	0.725	0.729	0.734
Dominican Republic	0.435	0.449	0.463	0.477	0.49	0.499	0.506	0.513	0.519	0.524	0.527	0.53	0.535	0.54	0.545	0.551	0.558	0.566	0.574	0.582	0.589	0.596	0.603	0.609	0.615	0.622	0.629	0.637	0.643	0.649	0.656	0.662	0.668	0.675	0.682	0.69	0.696
Grenada	0.442	0.448	0.453	0.458	0.463	0.468	0.473	0.479	0.485	0.492	0.5	0.51	0.519	0.527	0.536	0.544	0.552	0.561	0.571	0.581	0.591	0.6	0.609	0.618	0.627	0.637	0.646	0.654	0.662	0.668	0.674	0.679	0.684	0.688	0.694	0.699	0.704
Guyana	0.496	0.503	0.509	0.514	0.517	0.518	0.519	0.519	0.519	0.518	0.517	0.518	0.52	0.525	0.53	0.537	0.545	0.552	0.559	0.565	0.569	0.573	0.576	0.579	0.582	0.584	0.587	0.592	0.596	0.601	0.607	0.614	0.621	0.628	0.636	0.643	0.649
Haiti	0.257	0.261	0.264	0.267	0.271	0.275	0.279	0.284	0.29	0.297	0.303	0.31	0.315	0.319	0.321	0.325	0.329	0.334	0.339	0.346	0.352	0.358	0.363	0.368	0.372	0.376	0.381	0.386	0.391	0.397	0.402	0.407	0.413	0.418	0.424	0.428	0.432
Jamaica	0.557	0.563	0.568	0.573	0.578	0.58	0.583	0.587	0.591	0.595	0.599	0.604	0.609	0.615	0.62	0.625	0.629	0.633	0.636	0.64	0.643	0.647	0.651	0.655	0.659	0.664	0.669	0.674	0.679	0.682	0.685	0.688	0.691	0.694	0.696	0.699	0.701
Saint Lucia	0.453	0.464	0.473	0.482	0.492	0.502	0.513	0.523	0.534	0.544	0.555	0.565	0.574	0.583	0.591	0.598	0.606	0.614	0.621	0.629	0.635	0.641	0.647	0.654	0.662	0.668	0.674	0.679	0.684	0.689	0.693	0.697	0.7	0.703	0.707	0.71	0.713
Saint Vincent and the Grenadines	0.418	0.428	0.438	0.449	0.46	0.471	0.48	0.489	0.499	0.508	0.516	0.524	0.533	0.541	0.547	0.555	0.562	0.569	0.576	0.583	0.589	0.595	0.602	0.608	0.615	0.621	0.627	0.633	0.638	0.642	0.645	0.648	0.651	0.655	0.659	0.663	0.667
Suriname	0.51	0.515	0.519	0.524	0.528	0.535	0.541	0.546	0.553	0.56	0.566	0.569	0.572	0.574	0.575	0.577	0.58	0.583	0.587	0.589	0.592	0.597	0.601	0.607	0.614	0.621	0.628	0.636	0.643	0.651	0.659	0.666	0.673	0.68	0.686	0.691	0.696
Trinidad and Tobago	0.592	0.595	0.599	0.603	0.608	0.615	0.624	0.633	0.642	0.649	0.655	0.659	0.663	0.666	0.668	0.669	0.671	0.675	0.68	0.687	0.694	0.7	0.706	0.713	0.719	0.725	0.731	0.738	0.745	0.75	0.755	0.758	0.76	0.762	0.764	0.765	0.766
Bermuda	0.68	0.687	0.692	0.698	0.703	0.708	0.713	0.719	0.724	0.729	0.733	0.737	0.741	0.745	0.749	0.752	0.756	0.76	0.763	0.767	0.77	0.774	0.777	0.781	0.785	0.789	0.793	0.796	0.799	0.801	0.803	0.806	0.808	0.811	0.813	0.816	0.819

Appendix Table 7: Socio-demographic Index values for all estimated GBD 2016 locations, 1980–2016

Location	1980	1981	1982	1983	1984	1985	1986	1987	1988	1989	1990	1991	1992	1993	1994	1995	1996	1997	1998
Puerto Rico	0.699	0.71	0.719	0.727	0.733	0.739	0.743	0.748	0.753	0.76	0.767	0.774	0.781	0.788	0.793	0.799	0.805	0.81	0.817
Virgin Islands, U.S.	0.723	0.732	0.738	0.743	0.749	0.755	0.76	0.764	0.767	0.771	0.777	0.782	0.789	0.796	0.802	0.808	0.814	0.82	0.825
Andean Latin America	0.45	0.457	0.465	0.473	0.481	0.488	0.496	0.504	0.511	0.518	0.524	0.53	0.536	0.541	0.548	0.555	0.561	0.568	0.575
Bolivia	0.354	0.361	0.368	0.376	0.383	0.39	0.397	0.405	0.412	0.418	0.425	0.432	0.439	0.446	0.453	0.461	0.469	0.477	0.485
Ecuador	0.45	0.458	0.467	0.475	0.484	0.493	0.501	0.509	0.516	0.523	0.53	0.537	0.543	0.55	0.556	0.563	0.569	0.576	0.583
Peru	0.478	0.485	0.493	0.5	0.507	0.514	0.521	0.529	0.537	0.543	0.548	0.553	0.558	0.563	0.569	0.576	0.582	0.59	0.597
Central Latin America	0.466	0.476	0.485	0.493	0.502	0.51	0.518	0.525	0.531	0.537	0.544	0.551	0.56	0.568	0.575	0.581	0.588	0.595	0.602
Colombia	0.485	0.493	0.5	0.506	0.513	0.519	0.526	0.532	0.538	0.544	0.55	0.556	0.562	0.568	0.574	0.581	0.588	0.595	0.601
Costa Rica	0.527	0.532	0.535	0.537	0.539	0.541	0.546	0.552	0.557	0.563	0.57	0.577	0.584	0.591	0.598	0.603	0.608	0.614	0.62
El Salvador	0.383	0.392	0.401	0.408	0.414	0.419	0.424	0.429	0.434	0.439	0.444	0.451	0.458	0.465	0.472	0.48	0.488	0.496	0.504
Guatemala	0.325	0.33	0.335	0.338	0.342	0.345	0.348	0.351	0.355	0.36	0.365	0.37	0.376	0.383	0.389	0.396	0.404	0.412	0.42
Honduras	0.295	0.304	0.313	0.321	0.329	0.337	0.345	0.353	0.362	0.372	0.381	0.39	0.4	0.409	0.418	0.427	0.435	0.443	0.451
Mexico	0.458	0.47	0.481	0.491	0.501	0.511	0.521	0.53	0.538	0.547	0.555	0.564	0.573	0.582	0.591	0.598	0.606	0.614	0.622
Nicaragua	0.409	0.412	0.415	0.418	0.421	0.424	0.427	0.43	0.431	0.433	0.434	0.436	0.438	0.44	0.443	0.447	0.453	0.459	0.466
Panama	0.542	0.552	0.56	0.568	0.574	0.581	0.588	0.594	0.597	0.6	0.603	0.607	0.611	0.615	0.62	0.624	0.628	0.633	0.64
Venezuela	0.533	0.54	0.547	0.557	0.568	0.577	0.583	0.588	0.594	0.592	0.59	0.602	0.619	0.628	0.629	0.632	0.637	0.642	0.647
Aguascalientes	0.49	0.502	0.512	0.521	0.53	0.538	0.548	0.558	0.567	0.576	0.586	0.597	0.608	0.617	0.624	0.631	0.639	0.648	0.656
Baja California	0.523	0.536	0.548	0.56	0.572	0.585	0.597	0.608	0.617	0.624	0.629	0.635	0.642	0.65	0.658	0.666	0.672	0.676	0.68
Baja California Sur	0.535	0.547	0.559	0.571	0.584	0.596	0.605	0.615	0.627	0.635	0.644	0.654	0.663	0.669	0.674	0.679	0.686	0.691	0.692
Campeche	0.437	0.449	0.459	0.468	0.477	0.485	0.494	0.503	0.511	0.52	0.53	0.541	0.551	0.56	0.567	0.574	0.582	0.593	0.603
Coahuila	0.499	0.512	0.523	0.533	0.543	0.552	0.562	0.572	0.582	0.593	0.605	0.617	0.63	0.64	0.649	0.656	0.663	0.671	0.679
Colima	0.472	0.485	0.496	0.506	0.517	0.528	0.54	0.553	0.565	0.579	0.591	0.604	0.616	0.625	0.632	0.638	0.645	0.652	0.659
Chiapas	0.3	0.313	0.323	0.332	0.338	0.342	0.345	0.345	0.342	0.339	0.342	0.355	0.375	0.393	0.41	0.427	0.444	0.459	0.472
Chihuahua	0.49	0.503	0.513	0.523	0.531	0.54	0.549	0.559	0.568	0.578	0.587	0.597	0.607	0.614	0.62	0.623	0.628	0.634	0.639
Distrito Federal	0.592	0.605	0.615	0.624	0.631	0.635	0.638	0.642	0.649	0.66	0.671	0.68	0.686	0.692	0.702	0.713	0.723	0.729	0.733
Durango	0.459	0.47	0.48	0.488	0.495	0.502	0.509	0.516	0.522	0.529	0.536	0.544	0.553	0.559	0.563	0.568	0.576	0.586	0.596
Guanajuato	0.384	0.396	0.407	0.417	0.427	0.437	0.448	0.461	0.472	0.485	0.497	0.511	0.524	0.534	0.543	0.551	0.562	0.573	0.583
Guerrero	0.355	0.367	0.378	0.387	0.395	0.404	0.413	0.423	0.431	0.437	0.441	0.444	0.445	0.442	0.438	0.439	0.445	0.454	0.464
Hidalgo	0.383	0.395	0.406	0.416	0.426	0.437	0.449	0.46	0.47	0.48	0.49	0.501	0.513	0.521	0.527	0.533	0.541	0.55	0.56
Jalisco	0.463	0.475	0.486	0.496	0.505	0.514	0.524	0.535	0.544	0.554	0.565	0.577	0.589	0.599	0.607	0.615	0.624	0.633	0.642
México	0.447	0.463	0.481	0.502	0.528	0.554	0.571	0.582	0.592	0.595	0.597	0.598	0.6	0.616	0.637	0.646	0.651	0.655	0.66
Michoacán de Ocampo	0.368	0.38	0.391	0.401	0.41	0.42	0.431	0.443	0.453	0.464	0.476	0.488	0.5	0.51	0.517	0.525	0.535	0.547	0.558
Morelos	0.479	0.492	0.503	0.513	0.523	0.533	0.543	0.554	0.564	0.576	0.587	0.599	0.611	0.62	0.627	0.634	0.64	0.647	0.653
Nayarit	0.428	0.442	0.454	0.464	0.473	0.482	0.491	0.501	0.511	0.522	0.533	0.545	0.557	0.567	0.575	0.582	0.592	0.602	0.612
Nuevo León	0.582	0.594	0.603	0.612	0.619	0.626	0.632	0.638	0.644	0.652	0.66	0.668	0.678	0.686	0.692	0.697	0.703	0.709	0.715
Oaxaca	0.351	0.362	0.371	0.38	0.388	0.396	0.406	0.417	0.427	0.437	0.446	0.456	0.466	0.471	0.474	0.476	0.481	0.487	0.494
Puebla	0.404	0.416	0.426	0.436	0.445	0.454	0.465	0.475	0.485	0.495	0.505	0.515	0.526	0.532	0.537	0.54	0.545	0.552	0.558
Querétaro	0.445	0.456	0.463	0.465	0.461	0.453	0.452	0.47	0.495	0.515	0.53	0.54	0.549	0.558	0.572	0.589	0.606	0.619	0.627
Quintana Roo	0.49	0.503	0.514	0.524	0.532	0.54	0.547	0.553	0.559	0.567	0.576	0.586	0.598	0.609	0.617	0.625	0.634	0.642	0.65
San Luis Potosí	0.375	0.39	0.403	0.414	0.425	0.436	0.449	0.462	0.474	0.487	0.5	0.513	0.526	0.536	0.544	0.552	0.561	0.572	0.583
Sinaloa	0.491	0.503	0.514	0.523	0.531	0.539	0.547	0.556	0.564	0.573	0.581	0.589	0.597	0.603	0.607	0.613	0.62	0.629	0.639
Sonora	0.528	0.541	0.552	0.561	0.569	0.577	0.584	0.592	0.598	0.606	0.614	0.623	0.632	0.64	0.645	0.65	0.656	0.663	0.67
Tabasco	0.42	0.432	0.443	0.452	0.461	0.469	0.478	0.487	0.496	0.506	0.515	0.525	0.536	0.545	0.552	0.561	0.572	0.584	0.597
Tamaulipas	0.504	0.516	0.526	0.535	0.544	0.553	0.562	0.572	0.582	0.593	0.605	0.617	0.629	0.638	0.646	0.653	0.66	0.668	0.674
Tlaxcala	0.359	0.371	0.384	0.397	0.413	0.432	0.455	0.479	0.499	0.514	0.524	0.533	0.538	0.545	0.559	0.573	0.585	0.593	0.599
Veracruz de Ignacio de la Llave	0.408	0.42	0.431	0.44	0.449	0.458	0.467	0.477	0.484	0.493	0.501	0.51	0.519	0.526	0.531	0.537	0.545	0.554	0.564
Yucatán	0.466	0.477	0.486	0.493	0.501	0.508	0.515	0.522	0.528	0.536	0.545	0.555	0.566	0.575	0.582	0.59	0.599	0.608	0.617
Zacatecas	0.381	0.393	0.405	0.415	0.425	0.435	0.446	0.459	0.471	0.484	0.498	0.512	0.526	0.537	0.546	0.554	0.564	0.574	0.583
Tropical Latin America	0.449	0.461	0.471	0.48	0.489	0.498	0.507	0.515	0.524	0.532	0.539	0.546	0.553	0.56	0.568	0.576	0.583	0.591	0.598
Brazil	0.449	0.461	0.471	0.48	0.49	0.499	0.508	0.516	0.525	0.533	0.54	0.548	0.554	0.562	0.569	0.577	0.585	0.592	0.6
Paraguay	0.437	0.444	0.448	0.451	0.454	0.456	0.459	0.461	0.465	0.469	0.473	0.478	0.484	0.49	0.496	0.504	0.511	0.519	0.527
Acre	0.332	0.346	0.358	0.369	0.38	0.392	0.404	0.416	0.428	0.439	0.449	0.459	0.467	0.474	0.483	0.492	0.501	0.509	0.517
Alagoas	0.314	0.325	0.334	0.342	0.35	0.358	0.368	0.378	0.388	0.397	0.406	0.414	0.422	0.431	0.441	0.452	0.461	0.469	0.477
Amazonas	0.367	0.382	0.394	0.405	0.416	0.427	0.439	0.452	0.464	0.475	0.486	0.497	0.506	0.513	0.523	0.534	0.544	0.552	0.56
Amapá	0.352	0.366	0.38	0.393	0.406	0.419	0.432	0.445	0.457	0.468	0.477	0.486	0.494	0.501	0.509	0.517	0.528	0.54	0.554
Bahia	0.356	0.365	0.373	0.381	0.389	0.397	0.406	0.417	0.428	0.438	0.447	0.456	0.464	0.472	0.481	0.49	0.498	0.507	0.517
Ceará	0.346	0.355	0.362	0.369	0.376	0.382	0.392	0.406	0.417	0.429	0.439	0.448	0.455	0.463	0.472	0.481	0.492	0.501	0.509

Location	1999	2000	2001	2002	2003	2004	2005	2006	2007	2008	2009	2010	2011	2012	2013	2014	2015	2016
Puerto Rico	0.822	0.828	0.834	0.84	0.845	0.85	0.854	0.858	0.86	0.863	0.864	0.866	0.867	0.868	0.87	0.871	0.873	0.875
Virgin Islands, U.S.	0.829	0.833	0.836	0.839	0.842	0.845	0.85	0.855	0.861	0.864	0.868	0.871	0.874	0.875	0.876	0.876	0.877	0.877
Andean Latin America	0.582	0.588	0.594	0.6	0.605	0.61	0.615	0.62	0.625	0.63	0.635	0.64	0.644	0.649	0.653	0.657	0.662	0.666
Bolivia	0.494	0.502	0.51	0.518	0.525	0.531	0.539	0.546	0.553	0.56	0.567	0.573	0.58	0.586	0.593	0.599	0.605	0.61
Ecuador	0.588	0.593	0.598	0.603	0.608	0.613	0.619	0.624	0.629	0.635	0.64	0.645	0.65	0.655	0.66	0.665	0.67	0.674
Peru	0.603	0.61	0.615	0.621	0.626	0.63	0.634	0.639	0.644	0.648	0.652	0.656	0.66	0.663	0.666	0.67	0.674	0.678
Central Latin America	0.609	0.615	0.622	0.627	0.633	0.639	0.645	0.652	0.659	0.665	0.671	0.676	0.682	0.689	0.695	0.701	0.707	0.711
Colombia	0.606	0.611	0.616	0.62	0.624	0.629	0.634	0.639	0.645	0.652	0.658	0.664	0.672	0.679	0.686	0.694	0.701	0.707
Costa Rica	0.627	0.634	0.64	0.646	0.651	0.657	0.662	0.668	0.674	0.679	0.685	0.691	0.697	0.704	0.71	0.716	0.722	0.726
El Salvador	0.513	0.521	0.529	0.537	0.545	0.552	0.56	0.568	0.576	0.584	0.592	0.6	0.609	0.617	0.625	0.632	0.639	0.645
Guatemala	0.429	0.438	0.446	0.455	0.463	0.472	0.48	0.489	0.498	0.506	0.514	0.522	0.53	0.537	0.543	0.55	0.556	0.561
Honduras	0.459	0.466	0.474	0.481	0.488	0.495	0.502	0.508	0.515	0.522	0.527	0.533	0.539	0.545	0.551	0.556	0.562	0.567
Mexico	0.629	0.637	0.644	0.651	0.657	0.664	0.671	0.678	0.684	0.691	0.695	0.701	0.706	0.712	0.718	0.723	0.729	0.734
Nicaragua	0.473	0.481	0.488	0.496	0.502	0.509	0.516	0.523	0.53	0.537	0.543	0.549	0.555	0.562	0.569	0.576	0.583	0.588
Panama	0.647	0.653	0.66	0.666	0.67	0.674	0.679	0.683	0.688	0.693	0.698	0.704	0.71	0.718	0.726	0.735	0.744	0.752
Venezuela	0.65	0.654	0.657	0.66	0.662	0.667	0.673	0.681	0.688	0.696	0.702	0.708	0.714	0.721	0.728	0.734	0.738	0.742
Aguascalientes	0.663	0.67	0.675	0.681	0.687	0.693	0.699	0.705	0.711	0.715	0.719	0.723	0.728	0.733	0.739	0.744	0.749	0.754
Baja California	0.687	0.695	0.703	0.709	0.713	0.715	0.718	0.722	0.728	0.735	0.74	0.745	0.751	0.758	0.764	0.769	0.773	0.776
Baja California Sur	0.689	0.689	0.694	0.698	0.701	0.707	0.712	0.711	0.707	0.706	0.71	0.718	0.729	0.738	0.744	0.748	0.753	0.757
Campeche	0.614	0.625	0.635	0.643	0.651	0.658	0.665	0.671	0.677	0.682	0.685	0.688	0.693	0.7	0.707	0.713	0.72	0.726
Coahuila	0.685	0.691	0.697	0.702	0.708	0.714	0.72	0.726	0.733	0.739	0.743	0.747	0.751	0.754	0.758	0.761	0.766	0.77
Colima	0.666	0.672	0.678	0.683	0.689	0.695	0.7	0.706	0.712	0.716	0.719	0.723	0.727	0.732	0.737	0.742	0.748	0.753
Chiapas	0.483	0.491	0.499	0.508	0.517	0.527	0.536	0.546	0.555	0.563	0.57	0.577	0.585	0.593	0.6	0.608	0.616	0.622
Chihuahua	0.645	0.651	0.658	0.666	0.673	0.682	0.689	0.697	0.705	0.713	0.719	0.726	0.734	0.743	0.75	0.758	0.764	0.769
Distrito Federal	0.738	0.743	0.749	0.755	0.761	0.766	0.771	0.776	0.779	0.782	0.783	0.785	0.79	0.796	0.802	0.808	0.814	0.819
Durango	0.605	0.615	0.624	0.632	0.641	0.65	0.657	0.665	0.672	0.678	0.681	0.684	0.688	0.691	0.696	0.7	0.705	0.709
Guanajuato	0.593	0.603	0.611	0.619	0.627	0.634	0.642	0.65	0.658	0.666	0.671	0.677	0.683	0.688	0.694	0.7	0.706	0.711
Guerrero	0.473	0.483	0.494	0.505	0.517	0.529	0.54	0.551	0.562	0.57	0.577	0.585	0.595	0.606	0.617	0.626	0.634	0.639
Hidalgo	0.569	0.577	0.585	0.593	0.6	0.608	0.616	0.624	0.633	0.64	0.646	0.651	0.656	0.662	0.668	0.674	0.68	0.685
Jalisco	0.65	0.658	0.664	0.671	0.677	0.684	0.69	0.697	0.703	0.709	0.712	0.716	0.719	0.723	0.727	0.732	0.736	0.741
México	0.666	0.673	0.679	0.684	0.687	0.692	0.697	0.701	0.703	0.706	0.711	0.717	0.723	0.728	0.733	0.739	0.745	0.75
Michoacán de Ocampo	0.568	0.579	0.588	0.597	0.607	0.616	0.624	0.633	0.641	0.648	0.653	0.657	0.661	0.665	0.67	0.675	0.681	0.685
Morelos	0.659	0.664	0.67	0.675	0.68	0.686	0.692	0.698	0.705	0.71	0.714	0.718	0.722	0.726	0.73	0.734	0.737	0.741
Nayarit	0.621	0.63	0.638	0.645	0.652	0.658	0.664	0.67	0.675	0.679	0.683	0.687	0.692	0.698	0.705	0.711	0.718	0.723
Nuevo León	0.72	0.725	0.73	0.734	0.739	0.744	0.749	0.755	0.76	0.765	0.767	0.77	0.772	0.775	0.777	0.78	0.784	0.787
Oaxaca	0.501	0.508	0.515	0.523	0.533	0.544	0.555	0.568	0.581	0.592	0.601	0.609	0.617	0.623	0.629	0.634	0.639	0.643
Puebla	0.565	0.572	0.578	0.586	0.595	0.604	0.614	0.625	0.637	0.647	0.655	0.662	0.669	0.674	0.679	0.683	0.688	0.692
Querétaro	0.636	0.649	0.661	0.669	0.673	0.677	0.682	0.687	0.693	0.699	0.705	0.71	0.715	0.722	0.728	0.734	0.74	0.744
Quintana Roo	0.658	0.665	0.671	0.677	0.683	0.688	0.693	0.698	0.704	0.709	0.713	0.718	0.724	0.731	0.738	0.744	0.75	0.755
San Luis Potosí	0.593	0.604	0.613	0.622	0.631	0.64	0.648	0.656	0.665	0.672	0.678	0.685	0.694	0.703	0.711	0.72	0.728	0.734
Sinaloa	0.649	0.658	0.666	0.674	0.682	0.689	0.697	0.704	0.712	0.718	0.723	0.727	0.732	0.736	0.74	0.744	0.748	0.751
Sonora	0.677	0.683	0.69	0.696	0.702	0.709	0.716	0.723	0.731	0.737	0.742	0.746	0.751	0.756	0.761	0.767	0.772	0.777
Tabasco	0.607	0.616	0.624	0.63	0.636	0.642	0.648	0.654	0.66	0.665	0.669	0.674	0.68	0.685	0.691	0.696	0.701	0.706
Tamaulipas	0.68	0.685	0.689	0.693	0.697	0.701	0.706	0.712	0.719	0.726	0.732	0.738	0.744	0.751	0.756	0.761	0.766	0.77
Tlaxcala	0.605	0.612	0.62	0.628	0.635	0.644	0.651	0.657	0.664	0.672	0.677	0.681	0.685	0.693	0.701	0.708	0.715	0.72
Veracruz de Ignacio de la Llave	0.573	0.582	0.59	0.597	0.605	0.613	0.621	0.63	0.638	0.646	0.653	0.659	0.665	0.67	0.675	0.68	0.686	0.69
Yucatán	0.625	0.633	0.641	0.647	0.654	0.661	0.667	0.674	0.68	0.685	0.689	0.693	0.697	0.701	0.706	0.71	0.715	0.719
Zacatecas	0.592	0.601	0.608	0.615	0.622	0.629	0.635	0.641	0.647	0.652	0.656	0.66	0.666	0.672	0.679	0.686	0.693	0.699
Tropical Latin America	0.605	0.613	0.62	0.626	0.631	0.637	0.642	0.648	0.654	0.66	0.666	0.672	0.679	0.684	0.69	0.696	0.702	0.707
Brazil	0.607	0.614	0.621	0.627	0.633	0.638	0.644	0.65	0.656	0.662	0.667	0.673	0.68	0.686	0.691	0.697	0.703	0.708
Paraguay	0.535	0.543	0.55	0.558	0.565	0.573	0.58	0.588	0.596	0.604	0.611	0.62	0.628	0.636	0.645	0.653	0.661	0.668
Acre	0.522	0.528	0.534	0.539	0.543	0.548	0.555	0.563	0.57	0.578	0.586	0.595	0.604	0.612	0.621	0.629	0.636	0.642
Alagoas	0.485	0.493	0.501	0.508	0.515	0.522	0.529	0.536	0.544	0.553	0.56	0.569	0.577	0.584	0.592	0.599	0.605	0.61
Amazonas	0.569	0.578	0.586	0.592	0.597	0.603	0.609	0.615	0.622	0.63	0.637	0.645	0.652	0.659	0.666	0.674	0.68	0.686
Amapá	0.567	0.577	0.586	0.594	0.603	0.611	0.617	0.623	0.63	0.638	0.645	0.652	0.66	0.666	0.67	0.674	0.679	0.684
Bahia	0.524	0.532	0.539	0.545	0.552	0.558	0.564	0.571	0.578	0.584	0.591	0.598	0.606	0.613	0.62	0.627	0.633	0.638
Ceará	0.516	0.523	0.53	0.538	0.545	0.552	0.558	0.565	0.573	0.581	0.588	0.596	0.604	0.612	0.619	0.626	0.632	0.637

Appendix Table 7: Socio-demographic Index values for all estimated GBD 2016 locations, 1980–2016

Location	1980	1981	1982	1983	1984	1985	1986	1987	1988	1989	1990	1991	1992	1993	1994	1995	1996	1997	1998	1999	2000	2001	2002	2003	2004	2005	2006	2007	2008	2009	2010	2011	2012	2013	2014	2015	2016
Distrito Federal	0.597	0.607	0.617	0.625	0.634	0.643	0.652	0.659	0.666	0.673	0.68	0.686	0.692	0.698	0.704	0.71	0.717	0.723	0.729	0.735	0.741	0.747	0.753	0.758	0.764	0.769	0.775	0.781	0.787	0.793	0.8	0.806	0.812	0.818	0.824	0.829	0.832
Espírito Santo	0.459	0.47	0.481	0.49	0.499	0.509	0.518	0.527	0.536	0.545	0.553	0.561	0.568	0.577	0.584	0.592	0.6	0.607	0.614	0.621	0.628	0.635	0.642	0.648	0.654	0.66	0.665	0.671	0.677	0.682	0.688	0.694	0.699	0.705	0.711	0.717	0.721
Goiás	0.396	0.411	0.425	0.437	0.45	0.462	0.473	0.483	0.493	0.502	0.511	0.52	0.528	0.537	0.546	0.555	0.565	0.574	0.582	0.59	0.597	0.604	0.611	0.617	0.623	0.628	0.635	0.641	0.647	0.653	0.659	0.665	0.67	0.676	0.682	0.689	0.693
Maranhão	0.309	0.314	0.319	0.324	0.329	0.333	0.341	0.352	0.361	0.371	0.38	0.387	0.393	0.403	0.413	0.424	0.435	0.446	0.457	0.468	0.479	0.488	0.496	0.503	0.511	0.517	0.525	0.533	0.541	0.549	0.557	0.566	0.574	0.581	0.588	0.595	0.6
Minas Gerais	0.45	0.462	0.473	0.483	0.493	0.503	0.512	0.518	0.525	0.532	0.539	0.546	0.552	0.559	0.566	0.574	0.582	0.589	0.596	0.603	0.609	0.615	0.621	0.627	0.632	0.638	0.643	0.65	0.656	0.661	0.668	0.674	0.68	0.686	0.692	0.697	0.702
Mato Grosso do Sul	0.401	0.418	0.432	0.445	0.457	0.471	0.481	0.489	0.498	0.506	0.514	0.522	0.53	0.539	0.548	0.558	0.567	0.576	0.584	0.591	0.598	0.604	0.61	0.615	0.62	0.626	0.632	0.638	0.644	0.649	0.654	0.659	0.664	0.669	0.675	0.681	0.686
Mato Grosso	0.419	0.433	0.445	0.456	0.467	0.48	0.49	0.498	0.507	0.516	0.524	0.532	0.54	0.549	0.559	0.569	0.578	0.587	0.595	0.602	0.609	0.615	0.621	0.626	0.631	0.636	0.642	0.647	0.653	0.658	0.664	0.67	0.675	0.68	0.687	0.693	0.699
Pará	0.364	0.375	0.385	0.393	0.402	0.411	0.42	0.428	0.436	0.444	0.451	0.458	0.465	0.473	0.483	0.493	0.503	0.511	0.519	0.526	0.532	0.537	0.541	0.545	0.549	0.554	0.56	0.566	0.574	0.581	0.589	0.597	0.605	0.612	0.619	0.625	0.63
Paraíba	0.325	0.337	0.347	0.356	0.365	0.372	0.384	0.399	0.412	0.424	0.436	0.444	0.451	0.459	0.467	0.476	0.486	0.495	0.504	0.512	0.519	0.527	0.533	0.54	0.546	0.553	0.559	0.567	0.574	0.581	0.589	0.596	0.603	0.61	0.616	0.622	0.627
Paraná	0.443	0.458	0.471	0.483	0.494	0.507	0.517	0.524	0.533	0.541	0.548	0.556	0.564	0.572	0.58	0.589	0.597	0.606	0.614	0.622	0.629	0.636	0.643	0.649	0.654	0.66	0.665	0.671	0.677	0.682	0.688	0.694	0.7	0.706	0.712	0.718	0.722
Pernambuco	0.364	0.377	0.387	0.396	0.405	0.414	0.425	0.437	0.448	0.458	0.468	0.477	0.485	0.492	0.5	0.508	0.516	0.523	0.53	0.536	0.543	0.549	0.555	0.561	0.567	0.573	0.579	0.585	0.592	0.598	0.605	0.611	0.617	0.623	0.63	0.636	0.64
Piauí	0.321	0.329	0.337	0.344	0.351	0.358	0.367	0.378	0.388	0.398	0.408	0.415	0.421	0.428	0.436	0.445	0.455	0.464	0.473	0.482	0.49	0.498	0.505	0.512	0.519	0.526	0.533	0.54	0.548	0.555	0.563	0.571	0.578	0.585	0.591	0.598	0.602
Rio de Janeiro	0.531	0.545	0.557	0.567	0.577	0.587	0.595	0.602	0.608	0.615	0.62	0.626	0.631	0.637	0.642	0.648	0.653	0.659	0.665	0.67	0.676	0.682	0.688	0.692	0.696	0.701	0.705	0.71	0.714	0.718	0.723	0.727	0.732	0.736	0.74	0.745	0.749
Rio Grande do Norte	0.357	0.369	0.38	0.39	0.4	0.409	0.42	0.431	0.442	0.452	0.461	0.469	0.477	0.485	0.494	0.504	0.515	0.524	0.531	0.539	0.546	0.554	0.562	0.568	0.575	0.582	0.589	0.596	0.603	0.61	0.618	0.625	0.632	0.639	0.645	0.651	0.656
Rondônia	0.358	0.373	0.386	0.398	0.41	0.422	0.434	0.446	0.457	0.467	0.477	0.486	0.494	0.502	0.511	0.519	0.528	0.536	0.545	0.552	0.56	0.568	0.575	0.582	0.589	0.597	0.604	0.612	0.619	0.626	0.633	0.64	0.646	0.653	0.66	0.666	0.671
Roraima	0.401	0.411	0.421	0.43	0.44	0.45	0.461	0.472	0.483	0.493	0.503	0.512	0.52	0.527	0.535	0.542	0.548	0.552	0.554	0.558	0.569	0.581	0.589	0.595	0.604	0.612	0.62	0.628	0.636	0.644	0.651	0.657	0.661	0.666	0.672	0.678	0.683
Rio Grande do Sul	0.506	0.516	0.525	0.533	0.541	0.549	0.557	0.565	0.572	0.579	0.585	0.592	0.598	0.604	0.61	0.617	0.622	0.628	0.635	0.641	0.647	0.654	0.66	0.665	0.671	0.676	0.682	0.688	0.693	0.698	0.703	0.708	0.712	0.717	0.722	0.727	0.732
Santa Catarina	0.477	0.489	0.499	0.508	0.517	0.526	0.536	0.544	0.553	0.561	0.569	0.577	0.584	0.592	0.6	0.609	0.617	0.624	0.632	0.639	0.647	0.654	0.66	0.665	0.671	0.676	0.681	0.687	0.693	0.698	0.704	0.711	0.716	0.722	0.729	0.735	0.74
Sergipe	0.336	0.35	0.361	0.372	0.382	0.391	0.403	0.417	0.43	0.442	0.453	0.463	0.472	0.481	0.49	0.501	0.511	0.52	0.529	0.537	0.546	0.554	0.562	0.569	0.576	0.582	0.589	0.596	0.604	0.611	0.619	0.627	0.634	0.641	0.648	0.654	0.659
São Paulo	0.53	0.542	0.552	0.561	0.571	0.581	0.589	0.596	0.602	0.609	0.615	0.621	0.628	0.634	0.64	0.647	0.653	0.659	0.666	0.672	0.679	0.686	0.692	0.697	0.702	0.706	0.711	0.716	0.721	0.726	0.73	0.736	0.74	0.745	0.75	0.756	0.76
Tocantins	0.38	0.389	0.398	0.406	0.415	0.424	0.434	0.443	0.452	0.461	0.469	0.477	0.486	0.494	0.502	0.511	0.521	0.53	0.539	0.547	0.556	0.563	0.571	0.577	0.584	0.591	0.597	0.604	0.612	0.619	0.626	0.634	0.642	0.649	0.656	0.663	0.668
North Africa and Middle East	0.382	0.392	0.401	0.41	0.418	0.427	0.436	0.446	0.455	0.465	0.475	0.485	0.495	0.505	0.513	0.522	0.531	0.539	0.548	0.556	0.564	0.571	0.578	0.586	0.593	0.601	0.608	0.615	0.622	0.628	0.634	0.64	0.647	0.654	0.661	0.669	0.674
North Africa and Middle East	0.382	0.392	0.401	0.41	0.418	0.427	0.436	0.446	0.455	0.465	0.475	0.485	0.495	0.505	0.513	0.522	0.531	0.539	0.548	0.556	0.564	0.571	0.578	0.586	0.593	0.601	0.608	0.615	0.622	0.628	0.634	0.64	0.647	0.654	0.661	0.669	0.674
Algeria	0.356	0.367	0.379	0.39	0.401	0.412	0.423	0.433	0.443	0.453	0.463	0.472	0.479	0.485	0.49	0.495	0.5	0.505	0.51	0.516	0.523	0.53	0.538	0.549	0.559	0.57	0.581	0.591	0.602	0.612	0.622	0.632	0.642	0.651	0.66	0.669	0.676
Bahrain	0.498	0.513	0.526	0.536	0.54	0.542	0.55	0.559	0.565	0.571	0.586	0.599	0.605	0.613	0.623	0.633	0.641	0.644	0.646	0.65	0.663	0.681	0.694	0.699	0.701	0.703	0.707	0.711	0.716	0.719	0.72	0.719	0.718	0.721	0.726	0.732	0.738
Egypt	0.347	0.358	0.369	0.38	0.391	0.402	0.413	0.425	0.435	0.446	0.458	0.469	0.48	0.49	0.499	0.509	0.518	0.527	0.535	0.543	0.551	0.558	0.566	0.573	0.579	0.585	0.591	0.596	0.601	0.607	0.612	0.617	0.623	0.629	0.637	0.645	0.653
Iran	0.379	0.387	0.395	0.404	0.412	0.422	0.434	0.449	0.466	0.487	0.51	0.536	0.562	0.585	0.607	0.625	0.642	0.656	0.667	0.676	0.683	0.689	0.695	0.701	0.707	0.713	0.719	0.725	0.731	0.736	0.742	0.748	0.752	0.757	0.762	0.766	0.769
Iraq	0.289	0.299	0.31	0.32	0.331	0.341	0.351	0.361	0.372	0.381	0.391	0.398	0.405	0.412	0.418	0.423	0.429	0.437	0.447	0.459	0.472	0.485	0.495	0.498	0.498	0.492	0.485	0.475	0.461	0.447	0.434	0.429	0.432	0.44	0.45	0.463	0.474
Jordan	0.264	0.294	0.322	0.346	0.369	0.389	0.409	0.428	0.448	0.466	0.483	0.497	0.511	0.524	0.536	0.548	0.558	0.568	0.577	0.586	0.595	0.603	0.61	0.615	0.621	0.626	0.632	0.637	0.643	0.649	0.656	0.663	0.669	0.676	0.683	0.689	0.694
Kuwait	0.46	0.476	0.494	0.515	0.54	0.566	0.59	0.611	0.628	0.642	0.652	0.657	0.66	0.659	0.653	0.649	0.655	0.671	0.689	0.702	0.711	0.717	0.721	0.726	0.732	0.738	0.747	0.758	0.77	0.781	0.791	0.8	0.808	0.816	0.823	0.829	0.832
Lebanon	0.51	0.525	0.536	0.547	0.561	0.575	0.588	0.602	0.613	0.621	0.627	0.636	0.645	0.653	0.661	0.669	0.678	0.687	0.697	0.706	0.715	0.723	0.73	0.738	0.744	0.751	0.757	0.763	0.77	0.777	0.785	0.792	0.797	0.801	0.804	0.807	0.81
Libya	0.565	0.57	0.575	0.579	0.583	0.588	0.591	0.595	0.598	0.601	0.605	0.611	0.617	0.623	0.63	0.636	0.643	0.651	0.659	0.667	0.675	0.684	0.692	0.702	0.712	0.723	0.734	0.746	0.757	0.768	0.781	0.785	0.793	0.8	0.804	0.807	0.806
Morocco	0.287	0.295	0.303	0.312	0.321	0.329	0.339	0.349	0.359	0.37	0.381	0.393	0.404	0.415	0.427	0.438	0.449	0.46	0.471	0.481	0.49	0.499	0.507	0.516	0.524	0.531	0.54	0.548	0.557	0.565	0.573	0.581	0.589	0.597	0.604	0.611	0.617
Palestine	0.328	0.341	0.351	0.36	0.369	0.376	0.384	0.391	0.397	0.404	0.411	0.417	0.424	0.43	0.437	0.446	0.457	0.467	0.478	0.488	0.497	0.504	0.51	0.513	0.51	0.502	0.49	0.472	0.452	0.432	0.418	0.413	0.412	0.416	0.425	0.438	0.45
Oman	0.107	0.109	0.111	0.113	0.115	0.135	0.156	0.176	0.194	0.214	0.234	0.256	0.277	0.298	0.318	0.337	0.356	0.376	0.396	0.417	0.438	0.458	0.479	0.499	0.52	0.54	0.561	0.581	0.602	0.621	0.639	0.658	0.676	0.691	0.704	0.714	0.72
Qatar	0.52	0.54	0.558	0.575	0.587	0.596	0.601	0.605	0.609	0.615	0.622	0.631	0.64	0.65	0.659	0.669	0.676	0.684	0.69	0.696	0.702	0.708	0.715	0.722	0.728	0.735	0.74	0.745	0.75	0.756	0.761	0.765	0.769	0.774	0.779	0.784	0.788
Saudi Arabia	0.21	0.225	0.242	0.26	0.277	0.293	0.308	0.323	0.338	0.353	0.368	0.383	0.398	0.414	0.429	0.445	0.462	0.479	0.497	0.515	0.533	0.551	0.569	0.587	0.605	0.623	0.642	0.66	0.679	0.696	0.713	0.73	0.746	0.761	0.775	0.788	0.797
Syria	0.28	0.29	0.301	0.311	0.32	0.33	0.339	0.348	0.357	0.366	0.376	0.386	0.397	0.408	0.419	0.43	0.441	0.451	0.462	0.472	0.483	0.493	0.503	0.514	0.525	0.537	0.549	0.56	0.571	0.582	0.593	0.603	0.611	0.618	0.625	0.632	0.638
Tunisia	0.353	0.364	0.376	0.388	0.4	0.412	0.425	0.437	0.45	0.462	0.476	0.489	0.502	0.515	0.527	0.539	0.551	0.563	0.573	0.583	0.591	0.599	0.606	0.612	0.618	0.623	0.629	0.635	0.64	0.646	0.653	0.659	0.667	0.674	0.682	0.69	0.696
Turkey	0.479	0.489	0.498	0.508	0.517	0.527	0.537	0.547	0.557	0.566	0.576	0.584	0.592	0.599	0.605	0.612	0.619	0.627	0.634	0.641	0.649	0.656	0.663	0.67	0.677	0.684	0.691	0.698	0.705	0.711	0.717	0.724	0.731	0.738	0.746	0.754	0.762
United Arab Emirates	0.34	0.365	0.389	0.413	0.436	0.458	0.48	0.501	0.521	0.539	0.557	0.574	0.59	0.606	0.62	0.633	0.645	0.656	0.667	0.677	0.686	0.695	0.703	0.71	0.716	0.721	0.725	0.729	0.732	0.736	0.74	0.745	0.751	0.756	0.762	0.768	0.772
Yemen	0.155	0.159	0.163	0.166	0.168	0.168	0.168	0.167	0.168	0.17	0.173	0.177	0.185	0.195	0.206	0.217	0.229	0.24	0.252	0.264	0.276	0.288	0.302	0.317	0.335	0.351	0.362	0.367	0.371	0.374	0.379	0.386	0.398	0.417	0.434	0.445	0.45
Afghanistan	0.134	0.136	0.139	0.142	0.145	0.147	0.15	0.153	0.154	0.156	0.158	0.16	0.163	0.165	0.165	0.166	0.166	0.167	0.168	0.169	0.17	0.171	0.175	0.181	0.188	0.195	0.203	0.211	0.22	0.229	0.238	0.247	0.255	0.262	0.269	0.276	0.281
Sudan	0.24	0.245	0.252	0.258	0.263	0.268	0.274	0.279	0.285	0.29	0.295	0.3	0.304	0.309	0.313	0.317	0.32	0.326	0.332	0.34	0.351	0.361	0.372	0.383	0.393	0.404	0.415	0.426	0.437	0.448	0.459	0.47	0.48	0.49	0.501	0.51	0.518
Ha'il	0.205	0.22	0.236	0.252	0.267	0.283	0.297	0.312	0.326	0.341	0.355	0.369	0.384	0.399	0.414	0.429	0.446	0.463	0.479	0.496	0.514	0.532	0.549	0.567	0.585	0.603	0.621	0.639	0.657	0.674	0.691	0.708	0.724	0.739	0.752	0.765	0.774
Qassim	0.15	0.169	0.19	0.211	0.231	0.251	0.269	0.288	0.306	0.324	0.341	0.357	0.373	0.389	0.405	0.422	0.439	0.458	0.476	0.495	0.515	0.534	0.554	0.573	0.592	0.61	0.629	0.647	0.666	0.684	0.701	0.717	0.733	0.748	0.762	0.776	0.785
Riyadh	0.185	0.203	0.223	0.244	0.264	0.283	0.301	0.319	0.337	0.353	0.369	0.385	0.401	0.417	0.434	0.451	0.468	0.486	0.505	0.523	0.543	0.562	0.58	0.599	0.618	0.637	0.656	0.675	0.693	0.711	0.727	0.742	0.756	0.769	0.783	0.795	0.804
Tabuk	0.194	0.21	0.229	0.248	0.267	0.285	0.301	0.316	0.332	0.347	0.361	0.376	0.391	0.406	0.422	0.438	0.454	0.471	0.489	0.507	0.525	0.543	0.562	0.58	0.598	0.62	0.64	0.659	0.678	0.695	0.712	0.729	0.745	0.76	0.775	0.79	0.8
Madinah	0.199	0.215	0.232	0.251	0.269	0.285	0.3	0.316	0.331	0.347	0.362	0.377	0.392	0.408	0.424	0.44	0.457	0.474	0.492	0.51	0.528	0.547	0.565	0.583	0.601	0.62	0.638	0.656	0.675	0.693	0.71	0.727	0.744	0.76	0.773	0.786	0.795
Makkah	0.231	0.246	0.261	0.277	0.293	0.308	0.323	0.337	0.352	0.367	0.382	0.396	0.411	0.427	0.442	0.458	0.474	0.491	0.508	0.525	0.543	0.56	0.577	0.594	0.611	0.628	0.645	0.662	0.679	0.695	0.71	0.726	0.741	0.755	0.768	0.779	0.788
Bahah	0.219	0.232	0.246	0.26	0.273	0.287	0.299	0.312	0.325	0.338	0.351	0.364	0.378	0.392	0.406	0.42	0.436	0.452	0.468	0.484	0.5	0.516	0.532	0.549	0.565	0.582	0.599	0.616	0.634	0.65	0.666	0.683	0.699	0.714	0.726	0.737	0.746
Northern Borders	0.264	0.278	0.293	0.308	0.323	0.337	0.351	0.364	0.378	0.392	0.406	0.42	0.434	0.45	0.465	0.48	0.497	0.513	0.53	0.546	0.563	0.58	0.596	0.612	0.629	0.647	0.665	0.681	0.698	0.713	0.728	0.742	0.755	0.766	0.779	0.79	0.797
Jawf	0.236	0.253	0.272	0.291	0.311	0.33	0.347	0.364	0.381	0.398	0.414	0.431	0.448	0.465	0.482	0.499	0.517	0.536	0.555	0.573	0.592	0.61	0.628	0.645	0.663	0.681	0.699	0.717	0.735	0.752	0.768	0.784	0.799	0.814	0.826	0.838	0.846
Jizan	0.202	0.212	0.223	0.234	0.245	0.255	0.265	0.275	0.285	0.296	0.306	0.317	0.329	0.341	0.353	0.365	0.379	0.393	0.407	0.421	0.436	0.45	0.465	0.48	0.496	0.513	0.53	0.546	0.564	0.581	0.597	0.614	0.63	0.645	0.658	0.67	0.679
'Asir	0.167	0.181	0.197	0.213	0.229	0.243	0.258	0.272	0.286	0.301	0.314	0.327	0.341	0.355	0.369	0.384	0.401	0.417	0.434	0.451	0.469	0.487	0.504	0.522	0.541	0.559	0.578	0.598	0.617	0.636	0.654	0.672	0.69	0.706	0.721	0.735	0.744
Najran	0.187	0.199	0.212	0.224	0.237	0.249	0.261	0.273	0.285	0.297	0.309	0.32	0.332	0.345	0.357	0.371	0.385	0.4	0.415	0.431	0.447	0.463	0.479	0.496	0.513	0.53	0.548	0.566	0.585	0.602	0.62	0.637	0.654	0.671	0.685	0.699	0.709
Eastern Province	0.209	0.225	0.243	0.261	0.28	0.297	0.313	0.329	0.346	0.362	0.377	0.393	0.409	0.425	0.441	0.458	0.475	0.493	0.511	0.529	0.548	0.566	0.584	0.603	0.621	0.639	0.658	0.676	0.694	0.711	0.728	0.745	0.761	0.776	0.79	0.803	0.813

Appendix Table 7: Socio-demographic Index values for all estimated GBD 2016 locations, 1980–2016

Location	1980	1981	1982	1983	1984	1985	1986	1987	1988	1989	1990	1991	1992	1993	1994	1995	1996	1997	1998	1999	2000	2001	2002	2003	2004	2005	2006	2007	2008	2009	2010	2011	2012	2013	2014	2015	2016
South Asia	0.288	0.293	0.298	0.303	0.309	0.314	0.32	0.327	0.334	0.341	0.349	0.356	0.364	0.371	0.378	0.386	0.394	0.402	0.409	0.417	0.425	0.432	0.44	0.447	0.455	0.464	0.473	0.482	0.491	0.5	0.51	0.519	0.529	0.539	0.549	0.56	0.569
South Asia	0.288	0.293	0.298	0.303	0.309	0.314	0.32	0.327	0.334	0.341	0.349	0.356	0.364	0.371	0.378	0.386	0.394	0.402	0.409	0.417	0.425	0.432	0.44	0.447	0.455	0.464	0.473	0.482	0.491	0.5	0.51	0.519	0.529	0.539	0.549	0.56	0.569
Bangladesh	0.254	0.258	0.263	0.269	0.275	0.281	0.287	0.294	0.3	0.307	0.315	0.323	0.331	0.339	0.347	0.355	0.363	0.37	0.377	0.384	0.39	0.396	0.402	0.407	0.414	0.42	0.427	0.434	0.442	0.45	0.459	0.467	0.476	0.485	0.494	0.503	0.511
Bhutan	0.237	0.242	0.247	0.253	0.259	0.265	0.272	0.279	0.286	0.293	0.301	0.31	0.318	0.327	0.337	0.348	0.359	0.37	0.382	0.394	0.406	0.418	0.431	0.444	0.457	0.47	0.482	0.494	0.506	0.518	0.53	0.541	0.552	0.563	0.572	0.581	0.588
India	0.298	0.303	0.308	0.313	0.318	0.324	0.329	0.336	0.342	0.35	0.357	0.364	0.371	0.378	0.385	0.393	0.401	0.408	0.416	0.424	0.432	0.44	0.447	0.455	0.464	0.473	0.482	0.492	0.502	0.512	0.522	0.533	0.543	0.553	0.564	0.575	0.584
Nepal	0.194	0.198	0.202	0.206	0.21	0.214	0.219	0.224	0.23	0.236	0.242	0.25	0.258	0.266	0.276	0.285	0.293	0.302	0.31	0.319	0.328	0.337	0.345	0.354	0.362	0.37	0.377	0.384	0.391	0.398	0.404	0.411	0.417	0.424	0.433	0.443	0.452
Pakistan	0.216	0.218	0.22	0.223	0.227	0.234	0.243	0.255	0.268	0.282	0.297	0.309	0.32	0.33	0.339	0.349	0.36	0.371	0.383	0.392	0.4	0.408	0.416	0.424	0.431	0.438	0.443	0.448	0.453	0.458	0.464	0.471	0.478	0.488	0.499	0.51	0.519
Andhra Pradesh	0.276	0.281	0.285	0.29	0.294	0.299	0.303	0.309	0.315	0.322	0.329	0.337	0.345	0.354	0.363	0.373	0.383	0.393	0.403	0.414	0.424	0.434	0.444	0.453	0.463	0.472	0.481	0.493	0.503	0.514	0.525	0.536	0.546	0.556	0.566	0.577	0.585
Arunachal Pradesh	0.263	0.269	0.274	0.28	0.286	0.292	0.3	0.307	0.315	0.322	0.33	0.338	0.346	0.354	0.362	0.371	0.38	0.389	0.397	0.405	0.413	0.422	0.43	0.438	0.447	0.457	0.466	0.477	0.487	0.5	0.513	0.526	0.539	0.552	0.565	0.578	0.589
Assam	0.292	0.298	0.304	0.311	0.318	0.325	0.332	0.339	0.346	0.353	0.361	0.367	0.373	0.378	0.383	0.389	0.394	0.4	0.405	0.413	0.421	0.429	0.437	0.446	0.454	0.462	0.471	0.479	0.487	0.496	0.504	0.514	0.523	0.532	0.542	0.552	0.562
Bihar	0.25	0.255	0.26	0.264	0.27	0.275	0.281	0.287	0.294	0.3	0.306	0.312	0.317	0.318	0.319	0.318	0.318	0.319	0.317	0.317	0.318	0.321	0.325	0.329	0.334	0.338	0.346	0.354	0.363	0.371	0.38	0.39	0.4	0.411	0.421	0.431	0.44
Chhattisgarh	0.256	0.26	0.264	0.269	0.273	0.277	0.282	0.287	0.292	0.298	0.304	0.31	0.316	0.323	0.329	0.336	0.344	0.352	0.36	0.369	0.376	0.386	0.395	0.405	0.414	0.425	0.436	0.449	0.461	0.473	0.485	0.497	0.509	0.521	0.534	0.547	0.558
Delhi	0.471	0.478	0.484	0.49	0.495	0.5	0.505	0.51	0.515	0.52	0.524	0.529	0.533	0.538	0.544	0.55	0.557	0.565	0.573	0.582	0.592	0.602	0.612	0.623	0.633	0.643	0.653	0.664	0.674	0.684	0.694	0.705	0.715	0.724	0.734	0.744	0.752
Goa	0.394	0.401	0.409	0.416	0.424	0.432	0.44	0.448	0.456	0.465	0.473	0.481	0.49	0.5	0.511	0.523	0.535	0.547	0.561	0.573	0.586	0.597	0.608	0.616	0.623	0.631	0.638	0.645	0.654	0.664	0.673	0.686	0.696	0.707	0.718	0.729	0.738
Gujarat	0.319	0.326	0.331	0.338	0.345	0.352	0.359	0.365	0.373	0.38	0.389	0.395	0.403	0.411	0.42	0.429	0.439	0.448	0.457	0.465	0.473	0.48	0.487	0.495	0.503	0.512	0.522	0.532	0.542	0.551	0.561	0.571	0.58	0.589	0.598	0.607	0.615
Haryana	0.317	0.324	0.331	0.338	0.345	0.353	0.361	0.368	0.377	0.386	0.395	0.405	0.413	0.422	0.431	0.439	0.449	0.458	0.467	0.475	0.484	0.494	0.503	0.513	0.524	0.534	0.545	0.557	0.569	0.58	0.591	0.602	0.613	0.624	0.635	0.646	0.655
Himachal Pradesh	0.289	0.296	0.303	0.311	0.318	0.326	0.334	0.343	0.353	0.363	0.374	0.385	0.396	0.407	0.419	0.43	0.443	0.456	0.469	0.483	0.496	0.508	0.52	0.531	0.541	0.553	0.564	0.575	0.587	0.598	0.61	0.622	0.633	0.644	0.654	0.662	0.669
Jammu and Kashmir	0.276	0.282	0.288	0.295	0.301	0.308	0.316	0.322	0.329	0.336	0.343	0.349	0.353	0.361	0.369	0.377	0.384	0.395	0.405	0.417	0.429	0.441	0.452	0.464	0.475	0.486	0.497	0.507	0.518	0.528	0.539	0.549	0.559	0.569	0.579	0.589	0.598
Jharkhand	0.259	0.262	0.266	0.269	0.273	0.277	0.281	0.285	0.29	0.295	0.3	0.306	0.311	0.316	0.322	0.329	0.334	0.342	0.35	0.358	0.365	0.373	0.381	0.389	0.397	0.406	0.415	0.424	0.433	0.442	0.453	0.464	0.475	0.487	0.499	0.512	0.523
Karnataka	0.296	0.301	0.306	0.312	0.318	0.324	0.33	0.337	0.344	0.352	0.36	0.368	0.376	0.385	0.395	0.405	0.415	0.425	0.436	0.447	0.457	0.465	0.474	0.482	0.49	0.499	0.509	0.521	0.532	0.543	0.554	0.565	0.576	0.586	0.596	0.606	0.614
Kerala	0.369	0.374	0.379	0.385	0.391	0.396	0.403	0.409	0.415	0.422	0.429	0.436	0.444	0.454	0.464	0.476	0.488	0.5	0.512	0.524	0.535	0.545	0.553	0.562	0.57	0.58	0.589	0.599	0.608	0.619	0.628	0.638	0.648	0.657	0.667	0.676	0.683
Madhya Pradesh	0.271	0.275	0.28	0.286	0.291	0.297	0.303	0.309	0.317	0.324	0.332	0.339	0.345	0.35	0.355	0.36	0.365	0.37	0.375	0.38	0.385	0.391	0.397	0.404	0.411	0.418	0.425	0.433	0.441	0.45	0.459	0.47	0.481	0.494	0.507	0.52	0.532
Maharashtra	0.339	0.346	0.353	0.36	0.368	0.375	0.383	0.391	0.399	0.408	0.418	0.426	0.435	0.444	0.454	0.464	0.475	0.485	0.494	0.503	0.512	0.519	0.526	0.534	0.543	0.553	0.564	0.576	0.588	0.599	0.61	0.62	0.63	0.639	0.648	0.658	0.666
Manipur	0.28	0.288	0.295	0.302	0.31	0.318	0.326	0.336	0.346	0.355	0.364	0.372	0.38	0.386	0.391	0.395	0.4	0.406	0.413	0.423	0.433	0.444	0.455	0.467	0.48	0.494	0.507	0.518	0.529	0.54	0.549	0.56	0.571	0.581	0.591	0.601	0.61
Meghalaya	0.287	0.291	0.296	0.3	0.305	0.31	0.316	0.323	0.328	0.335	0.342	0.348	0.353	0.358	0.36	0.363	0.367	0.372	0.379	0.389	0.402	0.417	0.431	0.443	0.455	0.467	0.477	0.488	0.498	0.508	0.519	0.53	0.541	0.552	0.563	0.575	0.585
Mizoram	0.334	0.34	0.346	0.352	0.359	0.369	0.378	0.39	0.399	0.407	0.414	0.422	0.429	0.436	0.44	0.446	0.453	0.459	0.465	0.473	0.482	0.492	0.502	0.511	0.52	0.528	0.537	0.545	0.553	0.562	0.572	0.581	0.591	0.601	0.611	0.621	0.63
Nagaland	0.343	0.348	0.354	0.36	0.366	0.371	0.377	0.383	0.389	0.395	0.401	0.406	0.409	0.413	0.416	0.419	0.422	0.428	0.434	0.442	0.456	0.473	0.49	0.505	0.519	0.533	0.545	0.559	0.572	0.585	0.597	0.61	0.622	0.634	0.645	0.654	0.661
Odisha	0.251	0.255	0.259	0.264	0.269	0.274	0.28	0.285	0.292	0.299	0.305	0.312	0.318	0.324	0.33	0.338	0.344	0.351	0.359	0.368	0.376	0.385	0.393	0.402	0.413	0.422	0.433	0.445	0.457	0.468	0.48	0.491	0.503	0.514	0.526	0.537	0.547
Punjab	0.329	0.337	0.344	0.352	0.359	0.367	0.375	0.383	0.392	0.401	0.409	0.418	0.427	0.436	0.445	0.455	0.465	0.474	0.484	0.493	0.503	0.511	0.519	0.527	0.535	0.545	0.555	0.566	0.576	0.587	0.598	0.608	0.618	0.627	0.636	0.643	0.65
Rajasthan	0.254	0.259	0.263	0.27	0.275	0.28	0.286	0.292	0.299	0.306	0.315	0.322	0.329	0.335	0.341	0.348	0.355	0.362	0.368	0.375	0.382	0.39	0.397	0.405	0.414	0.422	0.43	0.44	0.449	0.457	0.468	0.479	0.49	0.5	0.511	0.521	0.53
Sikkim	0.268	0.273	0.279	0.284	0.29	0.297	0.304	0.313	0.321	0.329	0.337	0.346	0.352	0.361	0.369	0.378	0.388	0.398	0.408	0.418	0.43	0.441	0.453	0.466	0.478	0.49	0.501	0.511	0.52	0.536	0.551	0.565	0.579	0.593	0.607	0.62	0.632
Tamil Nadu	0.309	0.315	0.319	0.325	0.33	0.337	0.343	0.351	0.359	0.367	0.376	0.385	0.395	0.405	0.417	0.428	0.44	0.452	0.464	0.476	0.487	0.496	0.505	0.513	0.521	0.531	0.541	0.552	0.563	0.575	0.587	0.599	0.61	0.621	0.632	0.64	0.648
Telangana	0.265	0.269	0.273	0.278	0.282	0.286	0.291	0.296	0.302	0.309	0.317	0.325	0.333	0.342	0.352	0.362	0.373	0.383	0.395	0.406	0.417	0.428	0.438	0.449	0.459	0.47	0.482	0.495	0.509	0.521	0.535	0.549	0.562	0.574	0.587	0.599	0.609
Tripura	0.315	0.319	0.324	0.328	0.331	0.335	0.339	0.343	0.348	0.353	0.358	0.362	0.366	0.372	0.378	0.386	0.395	0.404	0.412	0.425	0.438	0.451	0.462	0.47	0.478	0.485	0.491	0.497	0.504	0.511	0.518	0.527	0.536	0.545	0.554	0.564	0.573
Uttar Pradesh	0.265	0.269	0.273	0.278	0.282	0.287	0.293	0.299	0.306	0.312	0.32	0.326	0.332	0.336	0.34	0.344	0.348	0.351	0.355	0.358	0.362	0.368	0.374	0.382	0.39	0.398	0.407	0.415	0.423	0.432	0.441	0.451	0.462	0.473	0.485	0.498	0.508
Uttarakhand	0.257	0.262	0.267	0.272	0.277	0.283	0.289	0.295	0.302	0.309	0.317	0.324	0.332	0.34	0.349	0.358	0.368	0.379	0.389	0.4	0.412	0.424	0.436	0.449	0.463	0.477	0.491	0.505	0.519	0.535	0.551	0.568	0.583	0.598	0.613	0.628	0.639
West Bengal	0.324	0.328	0.331	0.335	0.339	0.343	0.347	0.352	0.356	0.361	0.366	0.371	0.376	0.382	0.389	0.396	0.404	0.412	0.42	0.429	0.438	0.446	0.454	0.462	0.47	0.478	0.486	0.496	0.506	0.516	0.526	0.536	0.546	0.555	0.565	0.573	0.58
The Six Minor Territories	0.38	0.388	0.397	0.405	0.413	0.42	0.429	0.437	0.445	0.452	0.461	0.468	0.475	0.482	0.49	0.498	0.506	0.516	0.525	0.534	0.543	0.552	0.562	0.572	0.581	0.592	0.602	0.613	0.623	0.634	0.644	0.654	0.663	0.672	0.682	0.691	0.698
Andhra Pradesh, Urban	0.377	0.382	0.387	0.392	0.397	0.403	0.408	0.413	0.42	0.427	0.434	0.441	0.447	0.454	0.462	0.47	0.479	0.488	0.498	0.51	0.521	0.532	0.541	0.55	0.559	0.567	0.576	0.586	0.595	0.604	0.613	0.623	0.632	0.641	0.65	0.66	0.668
Andhra Pradesh, Rural	0.239	0.242	0.246	0.249	0.253	0.257	0.261	0.265	0.271	0.277	0.284	0.291	0.298	0.307	0.317	0.327	0.337	0.347	0.358	0.368	0.379	0.389	0.398	0.407	0.417	0.425	0.435	0.445	0.455	0.465	0.475	0.485	0.495	0.506	0.516	0.528	0.537
Arunachal Pradesh, Urban	0.405	0.411	0.416	0.421	0.427	0.433	0.439	0.445	0.452	0.457	0.463	0.469	0.474	0.481	0.486	0.493	0.499	0.506	0.511	0.516	0.52	0.525	0.528	0.532	0.536	0.541	0.546	0.552	0.557	0.565	0.573	0.582	0.59	0.599	0.61	0.621	0.631
Arunachal Pradesh, Rural	0.251	0.256	0.261	0.265	0.271	0.276	0.283	0.289	0.296	0.302	0.309	0.316	0.323	0.331	0.338	0.346	0.353	0.361	0.369	0.376	0.384	0.392	0.401	0.409	0.419	0.428	0.438	0.45	0.46	0.474	0.488	0.501	0.515	0.528	0.541	0.554	0.565
Assam, Urban	0.443	0.449	0.454	0.46	0.467	0.473	0.478	0.484	0.489	0.494	0.499	0.504	0.508	0.513	0.519	0.525	0.531	0.536	0.541	0.549	0.556	0.564	0.571	0.579	0.586	0.592	0.599	0.606	0.612	0.618	0.624	0.63	0.636	0.643	0.65	0.657	0.663
Assam, Rural	0.274	0.28	0.286	0.292	0.299	0.306	0.313	0.32	0.327	0.334	0.342	0.348	0.353	0.358	0.363	0.368	0.374	0.379	0.384	0.391	0.399	0.407	0.415	0.423	0.432	0.44	0.449	0.457	0.466	0.474	0.483	0.492	0.501	0.511	0.521	0.532	0.542
Bihar, Urban	0.37	0.377	0.384	0.391	0.399	0.406	0.413	0.42	0.428	0.434	0.441	0.447	0.453	0.456	0.459	0.46	0.463	0.466	0.467	0.468	0.471	0.474	0.479	0.482	0.487	0.491	0.498	0.505	0.513	0.52	0.529	0.537	0.546	0.554	0.561	0.568	0.575
Bihar, Rural	0.231	0.235	0.239	0.243	0.248	0.253	0.258	0.264	0.27	0.275	0.281	0.287	0.292	0.294	0.294	0.294	0.294	0.296	0.295	0.295	0.297	0.299	0.304	0.308	0.313	0.317	0.325	0.333	0.342	0.35	0.359	0.368	0.379	0.389	0.4	0.41	0.419
Chhattisgarh, Urban	0.456	0.462	0.468	0.472	0.477	0.481	0.485	0.489	0.492	0.496	0.5	0.503	0.507	0.51	0.513	0.517	0.521	0.527	0.533	0.539	0.543	0.55	0.557	0.565	0.572	0.581	0.59	0.601	0.611	0.62	0.628	0.637	0.645	0.653	0.661	0.669	0.677
Chhattisgarh, Rural	0.203	0.206	0.21	0.214	0.218	0.223	0.228	0.233	0.238	0.244	0.251	0.257	0.264	0.271	0.278	0.286	0.294	0.302	0.31	0.319	0.328	0.337	0.346	0.356	0.365	0.376	0.387	0.399	0.412	0.424	0.435	0.448	0.461	0.474	0.488	0.502	0.514
Delhi, Urban	0.478	0.485	0.491	0.497	0.502	0.506	0.512	0.517	0.521	0.526	0.53	0.535	0.539	0.543	0.549	0.555	0.561	0.569	0.577	0.586	0.596	0.606	0.616	0.626	0.636	0.646	0.656	0.666	0.676	0.686	0.696	0.706	0.716	0.726	0.736	0.746	0.753
Delhi, Rural	0.388	0.395	0.402	0.409	0.415	0.422	0.429	0.436	0.443	0.45	0.457	0.464	0.471	0.477	0.483	0.49	0.497	0.505	0.515	0.525	0.536	0.547	0.558	0.569	0.58	0.591	0.603	0.614	0.624	0.636	0.647	0.658	0.669	0.681	0.692	0.703	0.713
Goa, Urban	0.459	0.464	0.471	0.476	0.483	0.489	0.495	0.501	0.507	0.514	0.52	0.526	0.533	0.541	0.551	0.561	0.572	0.582	0.595	0.606	0.616	0.626	0.635	0.642	0.648	0.654	0.66	0.665	0.672	0.681	0.69	0.701	0.711	0.721	0.732	0.744	0.753
Goa, Rural	0.365	0.372	0.38	0.386	0.394	0.401	0.409	0.417	0.424	0.433	0.441	0.449	0.458	0.468	0.479	0.491	0.503	0.515	0.529	0.542	0.554	0.566	0.577	0.585	0.592	0.6	0.608	0.616	0.625	0.636	0.646	0.658	0.669	0.679	0.69	0.701	0.71
Gujarat, Urban	0.408	0.415	0.422	0.431	0.439	0.446	0.454	0.459	0.467	0.474	0.482	0.487	0.494	0.501	0.51	0.519	0.529	0.538	0.547	0.555	0.562	0.569	0.575	0.581	0.587	0.594	0.602	0.611	0.618	0.627	0.635	0.643	0.65	0.657	0.664	0.671	0.677
Gujarat, Rural	0.273	0.278	0.283	0.289	0.294	0.3	0.306	0.312	0.319	0.326	0.334	0.341	0.349	0.356	0.364	0.373	0.382	0.39	0.399	0.407	0.414	0.421	0.428	0.435	0.443	0.452	0.461	0.471	0.48	0.49	0.5	0.51	0.52	0.53	0.541	0.552	0.561
Haryana, Urban	0.428	0.436	0.444	0.452	0.46	0.468	0.476	0.483	0.491	0.498	0.506	0.514	0.521	0.528	0.536	0.543	0.552	0.56	0.567	0.575	0.582	0.59	0.596	0.603	0.61	0.617	0.626	0.635	0.645	0.655	0.665	0.674	0.683	0.691	0.7	0.709	0.717
Haryana, Rural	0.284	0.29	0.296	0.302	0.308	0.315	0.322	0.329	0.337	0.346	0.354	0.364	0.372	0.38	0.389	0.397	0.407	0.416	0.424	0.433	0.441	0.45	0.459	0.469	0.478	0.488	0.499	0.511	0.523	0.535	0.547	0.559	0.57	0.581	0.592	0.604	0.614
Himachal Pradesh, Urban	0.433	0.441	0.449	0.457	0.464	0.472	0.48	0.488	0.497	0.505	0.514	0.522	0.531	0.54	0.549	0.558	0.569	0.579	0.59	0.602	0.614	0.624	0.635	0.645	0.655	0.665	0.675	0.685	0.692	0.699	0.708	0.715	0.723	0.729	0.736	0.743	0.75

Appendix Table 7: Socio-demographic Index values for all estimated GBD 2016 locations, 1980–2016

Location	1980	1981	1982	1983	1984	1985	1986	1987	1988	1989	1990	1991	1992	1993	1994	1995	1996	1997	1998	1999	2000	2001	2002	2003	2004	2005	2006	2007	2008	2009	2010	2011	2012	2013	2014	2015	2016
Himachal Pradesh, Rural	0.275	0.282	0.289	0.297	0.304	0.312	0.32	0.329	0.339	0.349	0.36	0.37	0.381	0.393	0.404	0.416	0.429	0.442	0.455	0.469	0.482	0.495	0.506	0.517	0.528	0.539	0.551	0.563	0.574	0.585	0.598	0.61	0.621	0.632	0.643	0.652	0.659
Jammu and Kashmir, Urban	0.366	0.374	0.383	0.392	0.401	0.411	0.42	0.428	0.437	0.444	0.451	0.457	0.462	0.47	0.478	0.486	0.494	0.506	0.516	0.528	0.54	0.551	0.562	0.572	0.581	0.59	0.599	0.607	0.615	0.623	0.629	0.636	0.643	0.65	0.657	0.664	0.671
Jammu and Kashmir, Rural	0.247	0.252	0.258	0.263	0.269	0.275	0.282	0.287	0.294	0.301	0.307	0.312	0.317	0.324	0.332	0.339	0.346	0.357	0.366	0.378	0.389	0.4	0.412	0.423	0.435	0.446	0.458	0.468	0.479	0.49	0.5	0.511	0.522	0.532	0.543	0.553	0.562
Jharkhand, Urban	0.453	0.457	0.462	0.466	0.469	0.472	0.476	0.479	0.483	0.487	0.492	0.497	0.502	0.508	0.514	0.521	0.527	0.536	0.545	0.554	0.561	0.569	0.577	0.584	0.591	0.599	0.607	0.615	0.622	0.63	0.638	0.646	0.653	0.659	0.666	0.673	0.679
Jharkhand, Rural	0.233	0.236	0.239	0.242	0.245	0.248	0.251	0.255	0.259	0.264	0.269	0.274	0.279	0.285	0.292	0.299	0.305	0.313	0.321	0.33	0.338	0.346	0.354	0.362	0.37	0.379	0.387	0.397	0.405	0.415	0.425	0.436	0.448	0.459	0.472	0.485	0.496
Karnataka, Urban	0.391	0.398	0.404	0.412	0.42	0.427	0.434	0.441	0.449	0.456	0.463	0.471	0.478	0.486	0.495	0.505	0.515	0.525	0.536	0.546	0.557	0.565	0.574	0.581	0.589	0.598	0.607	0.616	0.624	0.631	0.639	0.647	0.655	0.664	0.672	0.681	0.688
Karnataka, Rural	0.251	0.256	0.26	0.264	0.269	0.274	0.28	0.286	0.292	0.299	0.306	0.314	0.322	0.33	0.339	0.348	0.357	0.367	0.377	0.387	0.397	0.406	0.415	0.423	0.432	0.442	0.453	0.464	0.475	0.485	0.496	0.507	0.518	0.529	0.54	0.552	0.561
Kerala, Urban	0.408	0.412	0.417	0.422	0.428	0.432	0.438	0.443	0.448	0.454	0.46	0.466	0.474	0.483	0.494	0.506	0.518	0.53	0.542	0.554	0.565	0.574	0.582	0.589	0.597	0.605	0.613	0.621	0.629	0.638	0.646	0.655	0.664	0.673	0.683	0.692	0.699
Kerala, Rural	0.358	0.363	0.368	0.374	0.38	0.385	0.391	0.397	0.404	0.411	0.418	0.425	0.433	0.442	0.452	0.464	0.476	0.488	0.5	0.513	0.524	0.534	0.543	0.551	0.559	0.568	0.577	0.586	0.595	0.605	0.613	0.623	0.632	0.642	0.653	0.662	0.669
Madhya Pradesh, Urban	0.403	0.409	0.415	0.422	0.428	0.435	0.441	0.448	0.455	0.461	0.469	0.476	0.483	0.49	0.497	0.504	0.511	0.517	0.522	0.527	0.531	0.535	0.538	0.541	0.545	0.55	0.557	0.564	0.572	0.581	0.591	0.6	0.609	0.618	0.626	0.635	0.643
Madhya Pradesh, Rural	0.229	0.232	0.236	0.24	0.245	0.249	0.254	0.26	0.266	0.272	0.28	0.286	0.292	0.296	0.3	0.305	0.309	0.314	0.318	0.322	0.325	0.331	0.336	0.342	0.349	0.355	0.362	0.37	0.378	0.388	0.399	0.411	0.423	0.436	0.451	0.465	0.478
Maharashtra, Urban	0.421	0.428	0.436	0.444	0.451	0.458	0.465	0.472	0.478	0.486	0.493	0.499	0.506	0.514	0.522	0.531	0.54	0.55	0.558	0.568	0.576	0.584	0.591	0.598	0.606	0.616	0.627	0.638	0.648	0.658	0.669	0.678	0.687	0.695	0.703	0.712	0.719
Maharashtra, Rural	0.288	0.295	0.301	0.308	0.314	0.322	0.329	0.337	0.345	0.354	0.364	0.373	0.382	0.391	0.4	0.41	0.421	0.43	0.439	0.449	0.457	0.464	0.472	0.48	0.488	0.498	0.51	0.522	0.533	0.544	0.556	0.567	0.577	0.586	0.596	0.607	0.615
Manipur, Urban	0.346	0.353	0.361	0.369	0.378	0.385	0.393	0.402	0.411	0.419	0.427	0.434	0.441	0.447	0.453	0.458	0.465	0.472	0.479	0.489	0.498	0.509	0.519	0.53	0.541	0.554	0.564	0.574	0.582	0.592	0.6	0.609	0.618	0.627	0.636	0.643	0.65
Manipur, Rural	0.265	0.272	0.278	0.285	0.292	0.299	0.306	0.315	0.324	0.332	0.341	0.349	0.356	0.362	0.367	0.371	0.375	0.381	0.387	0.397	0.408	0.419	0.43	0.442	0.455	0.469	0.482	0.494	0.504	0.515	0.525	0.535	0.545	0.555	0.565	0.575	0.585
Meghalaya, Urban	0.456	0.459	0.46	0.462	0.463	0.465	0.466	0.468	0.469	0.471	0.473	0.474	0.474	0.476	0.479	0.484	0.49	0.498	0.506	0.516	0.529	0.545	0.559	0.572	0.584	0.595	0.605	0.613	0.621	0.63	0.638	0.648	0.657	0.667	0.677	0.687	0.696
Meghalaya, Rural	0.25	0.254	0.258	0.263	0.268	0.273	0.279	0.286	0.292	0.299	0.307	0.313	0.319	0.323	0.325	0.328	0.331	0.336	0.342	0.352	0.365	0.379	0.393	0.406	0.417	0.429	0.44	0.451	0.461	0.472	0.483	0.495	0.505	0.516	0.528	0.54	0.55
Mizoram, Urban	0.383	0.389	0.395	0.401	0.408	0.417	0.426	0.437	0.446	0.453	0.459	0.466	0.472	0.479	0.485	0.492	0.5	0.508	0.514	0.522	0.531	0.54	0.548	0.555	0.562	0.568	0.575	0.581	0.587	0.593	0.601	0.608	0.616	0.625	0.633	0.643	0.651
Mizoram, Rural	0.292	0.298	0.303	0.309	0.315	0.325	0.334	0.346	0.355	0.363	0.37	0.378	0.385	0.392	0.395	0.398	0.404	0.408	0.413	0.42	0.43	0.44	0.451	0.461	0.472	0.482	0.491	0.502	0.512	0.523	0.535	0.546	0.557	0.568	0.58	0.591	0.6
Nagaland, Urban	0.446	0.451	0.458	0.466	0.473	0.48	0.486	0.494	0.501	0.507	0.513	0.517	0.521	0.525	0.527	0.53	0.532	0.536	0.54	0.544	0.553	0.564	0.576	0.586	0.595	0.604	0.612	0.622	0.632	0.641	0.65	0.66	0.67	0.68	0.691	0.702	0.711
Nagaland, Rural	0.328	0.333	0.338	0.343	0.348	0.353	0.357	0.363	0.368	0.374	0.379	0.383	0.387	0.39	0.393	0.395	0.399	0.405	0.412	0.42	0.435	0.452	0.469	0.484	0.498	0.512	0.524	0.537	0.55	0.563	0.575	0.588	0.6	0.611	0.622	0.629	0.637
Odisha, Urban	0.37	0.376	0.382	0.389	0.396	0.403	0.411	0.417	0.424	0.432	0.438	0.444	0.45	0.455	0.461	0.469	0.474	0.481	0.488	0.496	0.504	0.512	0.519	0.527	0.536	0.544	0.552	0.561	0.569	0.575	0.583	0.591	0.599	0.608	0.617	0.627	0.635
Odisha, Rural	0.233	0.237	0.24	0.245	0.248	0.253	0.259	0.263	0.269	0.276	0.281	0.287	0.293	0.299	0.305	0.312	0.318	0.325	0.333	0.341	0.35	0.358	0.366	0.376	0.387	0.397	0.408	0.42	0.432	0.443	0.455	0.467	0.479	0.49	0.502	0.514	0.524
Punjab, Urban	0.441	0.449	0.456	0.463	0.47	0.477	0.484	0.491	0.498	0.506	0.513	0.519	0.526	0.533	0.541	0.548	0.556	0.564	0.571	0.578	0.585	0.592	0.598	0.605	0.611	0.619	0.627	0.636	0.645	0.654	0.662	0.671	0.679	0.688	0.696	0.703	0.709
Punjab, Rural	0.286	0.293	0.3	0.307	0.314	0.322	0.33	0.338	0.346	0.355	0.364	0.373	0.381	0.39	0.399	0.408	0.418	0.427	0.437	0.447	0.457	0.466	0.474	0.483	0.492	0.502	0.513	0.524	0.535	0.546	0.556	0.566	0.577	0.587	0.596	0.604	0.611
Rajasthan, Urban	0.374	0.379	0.384	0.39	0.396	0.401	0.407	0.412	0.419	0.425	0.433	0.44	0.446	0.452	0.459	0.465	0.473	0.481	0.489	0.499	0.507	0.516	0.523	0.531	0.539	0.546	0.554	0.561	0.569	0.576	0.585	0.594	0.602	0.61	0.618	0.626	0.632
Rajasthan, Rural	0.218	0.222	0.226	0.232	0.236	0.241	0.246	0.251	0.258	0.265	0.273	0.28	0.286	0.292	0.299	0.305	0.312	0.318	0.324	0.331	0.337	0.345	0.352	0.36	0.368	0.376	0.385	0.395	0.404	0.412	0.423	0.434	0.445	0.456	0.467	0.478	0.487
Sikkim, Urban	0.371	0.377	0.386	0.394	0.404	0.414	0.426	0.437	0.448	0.459	0.469	0.477	0.483	0.491	0.498	0.504	0.511	0.518	0.525	0.533	0.542	0.551	0.56	0.57	0.579	0.588	0.595	0.601	0.605	0.618	0.628	0.639	0.649	0.66	0.672	0.685	0.695
Sikkim, Rural	0.257	0.261	0.266	0.271	0.276	0.283	0.29	0.297	0.305	0.313	0.321	0.329	0.335	0.343	0.352	0.36	0.37	0.38	0.39	0.4	0.411	0.422	0.434	0.445	0.457	0.469	0.479	0.489	0.496	0.513	0.527	0.542	0.555	0.569	0.584	0.598	0.61
Tamil Nadu, Urban	0.391	0.398	0.403	0.408	0.413	0.419	0.425	0.432	0.439	0.446	0.453	0.459	0.467	0.476	0.486	0.495	0.505	0.515	0.526	0.537	0.547	0.557	0.565	0.573	0.581	0.59	0.599	0.609	0.617	0.627	0.636	0.646	0.656	0.667	0.678	0.686	0.693
Tamil Nadu, Rural	0.264	0.27	0.274	0.279	0.285	0.292	0.299	0.307	0.315	0.324	0.333	0.341	0.35	0.359	0.369	0.379	0.389	0.399	0.411	0.422	0.433	0.443	0.453	0.462	0.472	0.483	0.494	0.506	0.516	0.528	0.539	0.551	0.562	0.574	0.586	0.595	0.603
Telangana, Urban	0.37	0.376	0.382	0.389	0.396	0.402	0.409	0.416	0.424	0.432	0.44	0.449	0.457	0.465	0.474	0.483	0.494	0.503	0.514	0.526	0.539	0.55	0.56	0.569	0.578	0.587	0.596	0.607	0.618	0.626	0.637	0.647	0.658	0.668	0.679	0.689	0.698
Telangana, Rural	0.225	0.228	0.231	0.234	0.237	0.24	0.244	0.248	0.253	0.259	0.265	0.272	0.28	0.289	0.298	0.308	0.32	0.33	0.342	0.353	0.364	0.375	0.385	0.396	0.406	0.417	0.429	0.441	0.454	0.466	0.48	0.493	0.506	0.519	0.533	0.547	0.558
Tripura, Urban	0.447	0.45	0.453	0.455	0.456	0.458	0.459	0.46	0.463	0.465	0.467	0.469	0.469	0.474	0.479	0.485	0.493	0.501	0.507	0.519	0.53	0.542	0.549	0.555	0.559	0.563	0.567	0.57	0.574	0.579	0.584	0.592	0.599	0.608	0.617	0.627	0.635
Tripura, Rural	0.294	0.298	0.302	0.306	0.31	0.313	0.317	0.321	0.326	0.331	0.336	0.34	0.344	0.351	0.357	0.365	0.373	0.382	0.39	0.403	0.416	0.429	0.439	0.448	0.455	0.461	0.467	0.472	0.479	0.486	0.493	0.501	0.509	0.518	0.528	0.538	0.546
Uttar Pradesh, Urban	0.367	0.371	0.377	0.382	0.387	0.393	0.399	0.404	0.41	0.417	0.423	0.43	0.435	0.44	0.446	0.451	0.457	0.463	0.468	0.473	0.478	0.484	0.49	0.496	0.502	0.509	0.516	0.524	0.531	0.538	0.546	0.554	0.562	0.57	0.578	0.587	0.594
Uttar Pradesh, Rural	0.24	0.243	0.247	0.251	0.255	0.259	0.264	0.27	0.276	0.282	0.289	0.295	0.3	0.304	0.308	0.311	0.314	0.317	0.32	0.323	0.327	0.332	0.339	0.347	0.355	0.364	0.372	0.381	0.389	0.398	0.407	0.417	0.429	0.441	0.454	0.468	0.479
Uttarakhand, Urban	0.401	0.406	0.412	0.417	0.422	0.427	0.432	0.437	0.442	0.448	0.454	0.459	0.464	0.469	0.476	0.482	0.489	0.496	0.503	0.511	0.519	0.528	0.537	0.547	0.556	0.567	0.578	0.589	0.6	0.615	0.628	0.642	0.656	0.669	0.682	0.695	0.706
Uttarakhand, Rural	0.223	0.227	0.231	0.235	0.24	0.245	0.25	0.256	0.263	0.269	0.277	0.284	0.292	0.3	0.309	0.318	0.328	0.338	0.349	0.36	0.372	0.385	0.397	0.411	0.424	0.439	0.453	0.468	0.482	0.499	0.515	0.531	0.547	0.562	0.577	0.592	0.604
West Bengal, Urban	0.44	0.444	0.45	0.455	0.461	0.466	0.471	0.476	0.481	0.486	0.491	0.496	0.501	0.507	0.513	0.52	0.527	0.535	0.543	0.552	0.56	0.568	0.574	0.58	0.586	0.591	0.597	0.604	0.612	0.619	0.625	0.632	0.638	0.644	0.651	0.658	0.665
West Bengal, Rural	0.275	0.279	0.281	0.284	0.288	0.291	0.295	0.299	0.303	0.307	0.312	0.317	0.323	0.329	0.335	0.343	0.351	0.358	0.367	0.376	0.384	0.392	0.4	0.407	0.414	0.422	0.43	0.441	0.451	0.462	0.473	0.483	0.493	0.504	0.514	0.525	0.533
The Six Minor Territories, Rural	0.293	0.299	0.306	0.312	0.319	0.326	0.333	0.34	0.348	0.355	0.363	0.371	0.379	0.387	0.397	0.406	0.416	0.428	0.439	0.451	0.462	0.474	0.487	0.499	0.512	0.525	0.538	0.552	0.565	0.579	0.591	0.603	0.61	0.617	0.623	0.63	0.637
The Six Minor Territories, Urban	0.477	0.483	0.488	0.493	0.497	0.501	0.506	0.51	0.514	0.519	0.523	0.528	0.533	0.538	0.544	0.55	0.557	0.565	0.572	0.579	0.586	0.593	0.6	0.607	0.614	0.622	0.63	0.638	0.645	0.654	0.661	0.669	0.677	0.685	0.693	0.702	0.71
Sub-Saharan Africa	0.263	0.268	0.272	0.277	0.282	0.287	0.291	0.295	0.298	0.302	0.307	0.311	0.316	0.321	0.325	0.329	0.334	0.339	0.343	0.346	0.35	0.354	0.358	0.362	0.366	0.371	0.377	0.383	0.389	0.395	0.401	0.408	0.416	0.423	0.43	0.437	0.442
Central Sub-Saharan Africa	0.259	0.262	0.266	0.271	0.275	0.28	0.283	0.287	0.291	0.295	0.298	0.302	0.305	0.306	0.307	0.308	0.31	0.312	0.314	0.315	0.316	0.317	0.32	0.322	0.327	0.332	0.338	0.345	0.352	0.359	0.366	0.374	0.382	0.388	0.394	0.401	0.406
Angola	0.218	0.22	0.223	0.225	0.229	0.232	0.235	0.239	0.243	0.248	0.252	0.256	0.26	0.262	0.265	0.268	0.272	0.276	0.281	0.286	0.289	0.294	0.299	0.305	0.312	0.32	0.329	0.339	0.349	0.357	0.366	0.377	0.388	0.4	0.411	0.422	0.431
the Central African Republic	0.212	0.216	0.22	0.222	0.226	0.23	0.233	0.236	0.24	0.243	0.247	0.25	0.252	0.254	0.258	0.261	0.263	0.266	0.269	0.273	0.276	0.279	0.283	0.285	0.288	0.291	0.295	0.299	0.303	0.307	0.311	0.316	0.322	0.32	0.319	0.318	0.316
Congo (Brazzaville)	0.3	0.308	0.317	0.326	0.335	0.344	0.351	0.358	0.365	0.372	0.379	0.386	0.393	0.399	0.404	0.41	0.415	0.421	0.426	0.43	0.434	0.439	0.444	0.45	0.456	0.462	0.467	0.471	0.475	0.48	0.487	0.495	0.503	0.511	0.519	0.526	0.531
Democratic Republic of the Congo	0.246	0.249	0.253	0.257	0.262	0.267	0.271	0.274	0.277	0.281	0.283	0.284	0.285	0.284	0.282	0.28	0.277	0.273	0.268	0.264	0.257	0.251	0.246	0.243	0.242	0.243	0.242	0.241	0.242	0.244	0.251	0.26	0.27	0.282	0.292	0.304	0.312
Equatorial Guinea	0.242	0.252	0.26	0.268	0.275	0.279	0.282	0.284	0.286	0.289	0.29	0.292	0.297	0.303	0.311	0.321	0.343	0.382	0.409	0.433	0.459	0.486	0.509	0.528	0.548	0.565	0.579	0.592	0.605	0.614	0.624	0.633	0.643	0.651	0.658	0.665	0.669
Gabon	0.38	0.389	0.397	0.406	0.414	0.422	0.429	0.435	0.442	0.45	0.458	0.466	0.474	0.482	0.49	0.498	0.506	0.514	0.521	0.527	0.531	0.534	0.539	0.545	0.551	0.558	0.564	0.57	0.576	0.581	0.586	0.592	0.599	0.605	0.611	0.617	0.622
Eastern Sub-Saharan Africa	0.176	0.181	0.185	0.19	0.196	0.201	0.207	0.212	0.217	0.222	0.228	0.234	0.239	0.244	0.249	0.254	0.259	0.264	0.268	0.273	0.277	0.282	0.286	0.291	0.296	0.302	0.308	0.314	0.321	0.329	0.337	0.345	0.354	0.363	0.371	0.38	0.387
Burundi	0.131	0.137	0.142	0.148	0.154	0.16	0.166	0.172	0.178	0.183	0.188	0.193	0.197	0.2	0.202	0.202	0.202	0.201	0.202	0.202	0.204	0.205	0.208	0.21	0.214	0.217	0.221	0.224	0.229	0.234	0.24	0.246	0.252	0.259	0.266	0.272	0.277
Comoros	0.208	0.213	0.219	0.225	0.232	0.239	0.246	0.253	0.26	0.268	0.275	0.282	0.289	0.297	0.303	0.309	0.315	0.321	0.325	0.33	0.335	0.34	0.346	0.352	0.359	0.366	0.373	0.381	0.388	0.395	0.403	0.41	0.419	0.427	0.436	0.443	0.449
Djibouti	0.089	0.1	0.11	0.121	0.135	0.148	0.16	0.171	0.181	0.19	0.199	0.207	0.215	0.223	0.23	0.237	0.244	0.252	0.258	0.265	0.272	0.28	0.288	0.296	0.304	0.312	0.321	0.331	0.34	0.35	0.359	0.369	0.379	0.389	0.399	0.409	0.416
Eritrea	0.143	0.146	0.15	0.154	0.158	0.163	0.167	0.172	0.177	0.182	0.187	0.193	0.2	0.208	0.218	0.229	0.241	0.254	0.267	0.279	0.29	0.3	0.309	0.315	0.321	0.326	0.33	0.335	0.338	0.342	0.346	0.352	0.359	0.367	0.375	0.383	0.389
Ethiopia	0.122	0.125	0.128	0.132	0.136	0.139	0.142	0.145	0.148	0.15	0.153	0.156	0.158	0.161	0.163	0.166	0.171	0.175	0.179	0.182	0.187	0.192	0.196	0.2	0.205	0.212	0.22	0.229	0.239	0.25	0.26	0.272	0.284	0.295	0.306	0.317	0.326

Appendix Table 7: Socio-demographic Index values for all estimated GBD 2016 locations, 1980–2016

Location	1980	1981	1982	1983	1984	1985	1986	1987	1988	1989	1990	1991	1992	1993	1994	1995	1996	1997	1998	1999	2000	2001	2002	2003	2004	2005	2006	2007	2008	2009	2010	2011	2012	2013	2014	2015	2016
Kenya	0.22	0.232	0.245	0.257	0.269	0.28	0.291	0.302	0.313	0.323	0.335	0.346	0.357	0.367	0.376	0.384	0.393	0.4	0.405	0.41	0.415	0.42	0.425	0.429	0.434	0.44	0.446	0.452	0.459	0.465	0.473	0.481	0.489	0.498	0.506	0.514	0.52
Madagascar	0.195	0.201	0.207	0.213	0.219	0.226	0.232	0.238	0.244	0.249	0.255	0.26	0.264	0.267	0.269	0.272	0.275	0.278	0.282	0.286	0.289	0.294	0.297	0.302	0.306	0.311	0.315	0.319	0.323	0.327	0.331	0.338	0.344	0.35	0.355	0.361	0.365
Malawi	0.143	0.149	0.155	0.161	0.167	0.175	0.182	0.188	0.194	0.199	0.205	0.21	0.215	0.219	0.223	0.227	0.232	0.238	0.243	0.249	0.254	0.258	0.263	0.268	0.273	0.279	0.284	0.29	0.296	0.302	0.308	0.314	0.32	0.326	0.334	0.342	0.348
Mozambique	0.131	0.133	0.135	0.136	0.137	0.138	0.138	0.139	0.14	0.142	0.145	0.149	0.151	0.153	0.157	0.158	0.165	0.172	0.18	0.186	0.193	0.2	0.207	0.214	0.22	0.226	0.231	0.236	0.242	0.247	0.253	0.26	0.268	0.277	0.287	0.297	0.306
Rwanda	0.065	0.067	0.068	0.069	0.07	0.071	0.089	0.12	0.144	0.164	0.181	0.197	0.208	0.217	0.22	0.223	0.226	0.23	0.234	0.237	0.24	0.244	0.25	0.256	0.263	0.272	0.282	0.293	0.305	0.318	0.33	0.342	0.354	0.365	0.374	0.383	0.39
Somalia	0.161	0.162	0.164	0.166	0.168	0.171	0.173	0.176	0.18	0.183	0.187	0.19	0.192	0.194	0.195	0.196	0.197	0.198	0.199	0.2	0.201	0.203	0.205	0.208	0.212	0.216	0.22	0.225	0.23	0.235	0.24	0.245	0.251	0.256	0.26	0.265	0.268
Tanzania	0.176	0.183	0.19	0.197	0.205	0.213	0.22	0.226	0.233	0.239	0.245	0.251	0.256	0.259	0.263	0.271	0.279	0.288	0.295	0.302	0.308	0.314	0.32	0.326	0.332	0.339	0.345	0.352	0.359	0.367	0.375	0.384	0.392	0.402	0.411	0.421	0.428
Uganda	0.101	0.11	0.117	0.125	0.134	0.142	0.149	0.156	0.163	0.17	0.177	0.184	0.19	0.196	0.203	0.209	0.215	0.22	0.226	0.232	0.238	0.244	0.251	0.257	0.265	0.272	0.28	0.289	0.298	0.308	0.317	0.328	0.338	0.348	0.359	0.37	0.377
Zambia	0.193	0.205	0.216	0.228	0.239	0.251	0.261	0.271	0.281	0.291	0.299	0.307	0.315	0.321	0.326	0.331	0.336	0.34	0.343	0.346	0.35	0.354	0.357	0.362	0.366	0.371	0.377	0.385	0.393	0.401	0.41	0.419	0.428	0.437	0.446	0.454	0.46
South Sudan	0.053	0.054	0.054	0.055	0.056	0.056	0.057	0.062	0.07	0.077	0.082	0.088	0.092	0.095	0.098	0.102	0.105	0.108	0.111	0.114	0.117	0.12	0.123	0.127	0.13	0.134	0.138	0.143	0.148	0.152	0.158	0.164	0.17	0.176	0.182	0.188	0.192
Central	0.292	0.305	0.318	0.33	0.343	0.356	0.368	0.379	0.39	0.401	0.413	0.425	0.435	0.444	0.453	0.462	0.47	0.476	0.482	0.487	0.492	0.497	0.501	0.505	0.51	0.515	0.521	0.527	0.533	0.54	0.546	0.553	0.56	0.566	0.573	0.58	0.585
Coast	0.146	0.16	0.172	0.185	0.196	0.205	0.214	0.222	0.231	0.241	0.252	0.264	0.275	0.286	0.296	0.305	0.313	0.321	0.327	0.332	0.337	0.343	0.348	0.354	0.361	0.368	0.376	0.385	0.394	0.403	0.413	0.423	0.434	0.444	0.455	0.465	0.473
Eastern	0.196	0.209	0.221	0.233	0.245	0.256	0.266	0.277	0.287	0.298	0.31	0.322	0.333	0.344	0.353	0.362	0.371	0.379	0.385	0.391	0.396	0.401	0.407	0.412	0.418	0.424	0.431	0.438	0.445	0.452	0.46	0.467	0.475	0.483	0.49	0.498	0.503
Nairobi	0.42	0.431	0.442	0.453	0.464	0.475	0.486	0.496	0.506	0.516	0.526	0.536	0.545	0.553	0.56	0.567	0.574	0.58	0.585	0.589	0.593	0.598	0.602	0.607	0.611	0.617	0.623	0.63	0.637	0.644	0.652	0.66	0.669	0.677	0.685	0.694	0.701
North Eastern	0.049	0.05	0.051	0.052	0.053	0.053	0.054	0.055	0.056	0.057	0.058	0.059	0.061	0.062	0.067	0.075	0.08	0.081	0.076	0.068	0.069	0.07	0.071	0.072	0.073	0.074	0.075	0.077	0.078	0.079	0.08	0.081	0.083	0.084	0.092	0.112	0.124
Nyanza	0.204	0.216	0.229	0.241	0.253	0.264	0.274	0.285	0.295	0.306	0.318	0.331	0.343	0.353	0.364	0.373	0.383	0.391	0.398	0.404	0.411	0.417	0.423	0.429	0.436	0.443	0.45	0.458	0.466	0.475	0.483	0.491	0.5	0.508	0.516	0.524	0.53
Rift Valley	0.173	0.188	0.202	0.216	0.228	0.239	0.249	0.259	0.269	0.28	0.292	0.305	0.316	0.327	0.337	0.347	0.356	0.363	0.37	0.375	0.381	0.386	0.392	0.397	0.403	0.41	0.417	0.424	0.432	0.44	0.448	0.457	0.465	0.474	0.482	0.491	0.497
Western	0.228	0.24	0.251	0.263	0.274	0.284	0.294	0.303	0.312	0.322	0.332	0.343	0.353	0.362	0.37	0.378	0.385	0.391	0.396	0.4	0.404	0.408	0.412	0.416	0.421	0.426	0.431	0.437	0.443	0.45	0.456	0.463	0.47	0.477	0.484	0.491	0.496
Kiambu	0.313	0.327	0.341	0.355	0.368	0.382	0.394	0.406	0.418	0.429	0.441	0.452	0.462	0.47	0.478	0.486	0.493	0.498	0.503	0.507	0.511	0.515	0.519	0.523	0.527	0.531	0.536	0.541	0.547	0.553	0.559	0.566	0.574	0.581	0.588	0.596	0.601
Kirinyaga	0.28	0.292	0.303	0.314	0.326	0.337	0.348	0.358	0.369	0.379	0.389	0.399	0.408	0.417	0.427	0.437	0.446	0.454	0.461	0.467	0.473	0.479	0.484	0.49	0.495	0.501	0.508	0.514	0.521	0.527	0.533	0.54	0.546	0.552	0.558	0.564	0.569
Murang'a	0.278	0.29	0.303	0.315	0.328	0.34	0.352	0.363	0.374	0.384	0.395	0.406	0.415	0.424	0.432	0.44	0.447	0.454	0.461	0.466	0.472	0.477	0.482	0.487	0.492	0.497	0.503	0.509	0.515	0.52	0.526	0.531	0.537	0.542	0.547	0.552	0.556
Nyandarua	0.263	0.275	0.287	0.3	0.312	0.325	0.337	0.348	0.36	0.371	0.383	0.395	0.405	0.415	0.423	0.431	0.439	0.446	0.452	0.457	0.462	0.466	0.47	0.474	0.479	0.484	0.489	0.496	0.503	0.51	0.518	0.526	0.534	0.542	0.55	0.558	0.564
Nyeri	0.292	0.305	0.318	0.332	0.345	0.358	0.37	0.382	0.394	0.407	0.419	0.432	0.443	0.453	0.462	0.47	0.477	0.483	0.488	0.492	0.496	0.5	0.503	0.507	0.512	0.517	0.524	0.531	0.538	0.546	0.553	0.56	0.566	0.572	0.577	0.583	0.587
Kilifi	0.085	0.086	0.087	0.088	0.089	0.09	0.098	0.111	0.122	0.134	0.15	0.166	0.182	0.197	0.212	0.226	0.238	0.249	0.258	0.266	0.273	0.28	0.286	0.291	0.295	0.301	0.306	0.313	0.321	0.33	0.341	0.352	0.364	0.375	0.387	0.398	0.406
Kwale	0.13	0.141	0.151	0.161	0.17	0.177	0.185	0.192	0.2	0.209	0.221	0.234	0.246	0.255	0.261	0.266	0.271	0.275	0.278	0.28	0.281	0.283	0.285	0.289	0.294	0.3	0.306	0.314	0.324	0.334	0.346	0.357	0.369	0.381	0.393	0.404	0.412
Lamu	0.168	0.181	0.193	0.205	0.215	0.224	0.233	0.241	0.249	0.255	0.262	0.269	0.276	0.283	0.292	0.3	0.309	0.317	0.325	0.332	0.338	0.344	0.349	0.355	0.36	0.365	0.371	0.376	0.381	0.388	0.397	0.406	0.415	0.425	0.434	0.444	0.45
Mombasa	0.246	0.259	0.271	0.283	0.294	0.305	0.316	0.326	0.337	0.347	0.36	0.372	0.384	0.394	0.404	0.413	0.421	0.429	0.435	0.44	0.444	0.448	0.452	0.457	0.463	0.47	0.477	0.484	0.491	0.499	0.508	0.518	0.529	0.539	0.549	0.56	0.568
TaitaTaveta	0.156	0.172	0.186	0.2	0.212	0.223	0.233	0.242	0.252	0.263	0.276	0.29	0.303	0.315	0.327	0.339	0.349	0.359	0.367	0.374	0.38	0.386	0.392	0.399	0.408	0.417	0.426	0.435	0.444	0.453	0.464	0.474	0.484	0.494	0.504	0.513	0.52
TanaRiver	0.064	0.065	0.066	0.077	0.084	0.088	0.09	0.092	0.093	0.097	0.107	0.12	0.132	0.145	0.157	0.167	0.176	0.183	0.188	0.189	0.187	0.185	0.182	0.179	0.175	0.172	0.169	0.168	0.171	0.178	0.191	0.204	0.219	0.234	0.248	0.261	0.271
Embu	0.237	0.25	0.262	0.274	0.287	0.298	0.31	0.32	0.331	0.342	0.353	0.365	0.375	0.386	0.396	0.407	0.417	0.426	0.434	0.441	0.449	0.456	0.463	0.469	0.474	0.48	0.486	0.493	0.499	0.506	0.514	0.521	0.529	0.536	0.543	0.55	0.555
Isiolo	0.205	0.212	0.219	0.226	0.233	0.24	0.246	0.252	0.258	0.264	0.27	0.277	0.284	0.289	0.295	0.3	0.306	0.311	0.315	0.32	0.325	0.33	0.334	0.336	0.336	0.335	0.334	0.334	0.336	0.339	0.343	0.348	0.354	0.36	0.366	0.372	0.377
Kitui	0.145	0.158	0.171	0.183	0.195	0.207	0.217	0.227	0.237	0.246	0.255	0.265	0.274	0.283	0.293	0.302	0.311	0.319	0.327	0.333	0.339	0.345	0.351	0.357	0.364	0.371	0.378	0.386	0.394	0.402	0.411	0.42	0.429	0.438	0.447	0.455	0.461
Machakos	0.194	0.21	0.225	0.24	0.253	0.266	0.278	0.29	0.302	0.315	0.33	0.345	0.358	0.371	0.382	0.393	0.403	0.412	0.42	0.426	0.432	0.437	0.442	0.447	0.453	0.459	0.466	0.474	0.482	0.49	0.497	0.505	0.512	0.519	0.526	0.533	0.538
Makueni	0.154	0.175	0.193	0.21	0.225	0.239	0.252	0.264	0.276	0.288	0.301	0.314	0.325	0.336	0.344	0.351	0.358	0.363	0.367	0.369	0.369	0.369	0.37	0.373	0.378	0.385	0.393	0.402	0.411	0.422	0.432	0.442	0.451	0.46	0.468	0.475	0.48
Marsabit	0.184	0.189	0.193	0.198	0.203	0.207	0.212	0.216	0.22	0.224	0.229	0.234	0.238	0.242	0.246	0.25	0.254	0.257	0.261	0.264	0.268	0.272	0.277	0.282	0.288	0.293	0.299	0.304	0.308	0.312	0.315	0.318	0.322	0.326	0.33	0.335	0.339
Meru	0.209	0.221	0.232	0.243	0.254	0.264	0.274	0.284	0.294	0.305	0.318	0.332	0.345	0.356	0.367	0.377	0.386	0.395	0.402	0.408	0.413	0.419	0.425	0.43	0.435	0.44	0.445	0.451	0.455	0.461	0.467	0.475	0.482	0.49	0.498	0.505	0.512
TharakaNithi	0.204	0.216	0.227	0.238	0.25	0.26	0.271	0.282	0.293	0.303	0.315	0.327	0.338	0.349	0.359	0.368	0.377	0.386	0.393	0.4	0.407	0.413	0.42	0.428	0.435	0.444	0.452	0.462	0.471	0.48	0.489	0.497	0.506	0.514	0.522	0.53	0.536
Nairobi	0.42	0.431	0.442	0.453	0.464	0.475	0.486	0.496	0.506	0.516	0.526	0.536	0.545	0.553	0.56	0.567	0.574	0.58	0.585	0.589	0.593	0.598	0.602	0.607	0.611	0.617	0.623	0.63	0.637	0.644	0.652	0.66	0.669	0.677	0.685	0.694	0.701
Garissa	0.052	0.053	0.054	0.055	0.056	0.057	0.058	0.059	0.06	0.061	0.062	0.064	0.065	0.069	0.075	0.077	0.077	0.074	0.073	0.074	0.075	0.077	0.078	0.079	0.08	0.081	0.083	0.084	0.106	0.127	0.141	0.154	0.166	0.178	0.19	0.202	0.21
Mandera	0.045	0.046	0.047	0.048	0.049	0.05	0.051	0.052	0.053	0.054	0.055	0.057	0.065	0.079	0.088	0.095	0.1	0.103	0.102	0.095	0.086	0.074	0.07	0.072	0.073	0.074	0.075	0.076	0.077	0.077	0.079	0.08	0.081	0.083	0.085	0.087	0.089
Wajir	0.048	0.049	0.05	0.05	0.051	0.052	0.052	0.053	0.054	0.055	0.056	0.057	0.058	0.059	0.06	0.061	0.062	0.062	0.063	0.064	0.065	0.065	0.066	0.067	0.067	0.068	0.069	0.07	0.071	0.072	0.073	0.074	0.075	0.083	0.101	0.115	0.124
HomaBay	0.173	0.188	0.202	0.216	0.229	0.24	0.251	0.261	0.271	0.281	0.291	0.301	0.311	0.32	0.329	0.338	0.347	0.354	0.361	0.368	0.374	0.38	0.385	0.39	0.395	0.401	0.408	0.416	0.425	0.435	0.445	0.455	0.464	0.473	0.481	0.488	0.493
Kisii	0.206	0.218	0.23	0.242	0.254	0.264	0.275	0.285	0.296	0.308	0.322	0.336	0.349	0.362	0.375	0.387	0.399	0.409	0.417	0.425	0.432	0.439	0.447	0.454	0.462	0.47	0.478	0.487	0.495	0.502	0.51	0.518	0.526	0.534	0.542	0.55	0.556
Kisumu	0.214	0.228	0.241	0.254	0.266	0.278	0.29	0.301	0.313	0.326	0.341	0.355	0.369	0.38	0.388	0.396	0.403	0.409	0.413	0.417	0.421	0.425	0.43	0.435	0.441	0.448	0.456	0.465	0.475	0.485	0.495	0.504	0.512	0.521	0.529	0.536	0.542
Migori	0.18	0.194	0.208	0.22	0.233	0.244	0.255	0.264	0.274	0.283	0.294	0.304	0.314	0.324	0.334	0.343	0.353	0.362	0.37	0.378	0.386	0.395	0.404	0.411	0.417	0.422	0.428	0.435	0.44	0.445	0.451	0.458	0.465	0.473	0.48	0.488	0.494
Nyamira	0.268	0.28	0.292	0.304	0.316	0.328	0.34	0.351	0.363	0.374	0.385	0.397	0.409	0.419	0.43	0.44	0.449	0.458	0.464	0.47	0.476	0.483	0.49	0.496	0.501	0.507	0.514	0.521	0.528	0.537	0.545	0.555	0.563	0.572	0.58	0.588	0.594
Siaya	0.166	0.176	0.186	0.196	0.206	0.215	0.225	0.234	0.244	0.253	0.264	0.275	0.285	0.295	0.305	0.314	0.324	0.333	0.34	0.347	0.355	0.363	0.371	0.38	0.39	0.401	0.411	0.422	0.432	0.442	0.451	0.46	0.47	0.479	0.489	0.498	0.505
Baringo	0.151	0.169	0.185	0.2	0.213	0.225	0.236	0.247	0.258	0.268	0.28	0.292	0.304	0.313	0.322	0.329	0.337	0.344	0.351	0.358	0.366	0.373	0.379	0.383	0.385	0.387	0.39	0.393	0.398	0.406	0.414	0.422	0.432	0.441	0.45	0.458	0.465
Bomet	0.099	0.108	0.143	0.169	0.189	0.204	0.218	0.231	0.243	0.254	0.268	0.281	0.293	0.305	0.316	0.327	0.338	0.348	0.357	0.366	0.377	0.388	0.398	0.409	0.418	0.427	0.436	0.446	0.454	0.462	0.47	0.479	0.488	0.497	0.506	0.514	0.521
Elgeyo-Marakwet	0.149	0.167	0.183	0.198	0.211	0.223	0.234	0.245	0.255	0.266	0.278	0.291	0.303	0.313	0.322	0.331	0.34	0.348	0.355	0.362	0.371	0.379	0.388	0.395	0.401	0.408	0.415	0.424	0.433	0.444	0.453	0.463	0.473	0.483	0.492	0.501	0.508
Kajiado	0.197	0.213	0.227	0.241	0.254	0.266	0.278	0.29	0.301	0.313	0.325	0.338	0.35	0.36	0.368	0.376	0.383	0.389	0.393	0.395	0.399	0.403	0.409	0.416	0.426	0.437	0.448	0.46	0.471	0.48	0.489	0.498	0.506	0.515	0.524	0.533	0.539
Kericho	0.094	0.096	0.108	0.135	0.154	0.169	0.183	0.196	0.208	0.224	0.242	0.261	0.278	0.295	0.313	0.328	0.343	0.356	0.368	0.378	0.388	0.397	0.406	0.413	0.42	0.428	0.436	0.445	0.455	0.466	0.476	0.487	0.498	0.509	0.519	0.529	0.536
Laikipia	0.229	0.241	0.253	0.265	0.276	0.287	0.297	0.307	0.317	0.327	0.338	0.349	0.36	0.372	0.386	0.398	0.41	0.42	0.428	0.435	0.442	0.449	0.456	0.463	0.47	0.478	0.487	0.496	0.507	0.518	0.527	0.537	0.547	0.556	0.566	0.575	0.582
Nakuru	0.208	0.221	0.232	0.244	0.255	0.266	0.277	0.287	0.298	0.309	0.321	0.333	0.345	0.355	0.365	0.373	0.382	0.389	0.394	0.398	0.403	0.408	0.413	0.42	0.43	0.441	0.453	0.466	0.479	0.492	0.503	0.515	0.526	0.537	0.546	0.556	0.563
Nandi	0.099	0.124	0.152	0.173	0.191	0.206	0.219	0.232	0.244	0.256	0.27	0.285	0.299	0.313	0.327	0.339	0.35	0.36	0.368	0.374	0.382	0.39	0.399	0.408	0.417	0.427	0.436	0.446	0.455	0.465	0.474	0.483	0.491	0.5	0.508	0.516	0.522
Narok	0.106	0.125	0.141	0.155	0.168	0.178	0.188	0.198	0.208	0.219	0.231	0.244	0.257	0.269	0.282	0.293	0.305	0.315	0.323	0.328	0.332	0.336	0.339	0.345	0.353	0.361	0.368	0.375	0.381	0.387	0.394	0.402	0.41	0.418	0.427	0.435	0.441
Samburu	0.169	0.175	0.181	0.187	0.192	0.196	0.201	0.205	0.209	0.212	0.216	0.22	0.224	0.227	0.229	0.231	0.233	0.235	0.235	0.235	0.236	0.238	0.241	0.245	0.251	0.257	0.264	0.27	0.275	0.281	0.286	0.292	0.297	0.302	0.308	0.313	0.316

Appendix Table 7: Socio-demographic Index values for all estimated GBD 2016 locations, 1980–2016

Location	1980	1981	1982	1983	1984	1985	1986	1987	1988	1989	1990	1991	1992	1993	1994	1995	1996	1997	1998	1999	2000	2001	2002	2003	2004	2005	2006	2007	2008	2009	2010	2011	2012	2013	2014	2015	2016
TransNzoia	0.103	0.137	0.161	0.18	0.197	0.21	0.222	0.234	0.246	0.26	0.278	0.296	0.313	0.328	0.341	0.353	0.364	0.374	0.382	0.39	0.398	0.405	0.412	0.418	0.422	0.427	0.432	0.438	0.445	0.453	0.462	0.471	0.48	0.489	0.498	0.506	0.513
Turkana	0.185	0.19	0.195	0.2	0.205	0.209	0.213	0.217	0.22	0.223	0.227	0.231	0.235	0.238	0.241	0.244	0.247	0.249	0.251	0.25	0.249	0.248	0.248	0.249	0.251	0.253	0.256	0.26	0.265	0.268	0.271	0.273	0.276	0.279	0.283	0.287	0.289
UasinGishu	0.183	0.202	0.22	0.237	0.252	0.266	0.279	0.291	0.303	0.316	0.329	0.342	0.355	0.366	0.376	0.386	0.395	0.404	0.413	0.422	0.432	0.443	0.453	0.462	0.47	0.478	0.486	0.495	0.504	0.513	0.522	0.53	0.539	0.548	0.556	0.564	0.57
WestPokot	0.153	0.16	0.168	0.176	0.183	0.19	0.196	0.203	0.211	0.219	0.23	0.24	0.25	0.258	0.264	0.27	0.275	0.279	0.28	0.281	0.283	0.284	0.286	0.289	0.292	0.296	0.301	0.308	0.314	0.321	0.328	0.335	0.343	0.351	0.359	0.367	0.373
Bungoma	0.199	0.214	0.228	0.242	0.254	0.266	0.277	0.287	0.296	0.305	0.316	0.327	0.336	0.345	0.354	0.362	0.37	0.378	0.385	0.392	0.399	0.406	0.413	0.42	0.426	0.432	0.438	0.444	0.45	0.456	0.462	0.469	0.477	0.484	0.492	0.499	0.505
Busia	0.235	0.246	0.256	0.266	0.276	0.285	0.294	0.303	0.312	0.321	0.331	0.341	0.35	0.359	0.365	0.371	0.377	0.382	0.385	0.387	0.39	0.393	0.396	0.399	0.402	0.406	0.41	0.415	0.421	0.427	0.433	0.44	0.447	0.454	0.461	0.468	0.473
Kakamega	0.23	0.241	0.252	0.263	0.273	0.283	0.292	0.302	0.311	0.321	0.332	0.344	0.355	0.365	0.374	0.383	0.39	0.397	0.402	0.405	0.408	0.411	0.414	0.417	0.421	0.426	0.431	0.438	0.444	0.45	0.457	0.463	0.47	0.477	0.484	0.491	0.496
Vihiga	0.258	0.27	0.281	0.293	0.304	0.315	0.325	0.334	0.344	0.352	0.361	0.37	0.378	0.385	0.391	0.397	0.401	0.405	0.408	0.409	0.41	0.412	0.415	0.418	0.422	0.428	0.436	0.446	0.456	0.467	0.476	0.485	0.493	0.5	0.507	0.513	0.517
Southern Sub-Saharan Africa	0.505	0.511	0.516	0.521	0.527	0.531	0.536	0.541	0.546	0.552	0.558	0.564	0.57	0.577	0.583	0.589	0.595	0.601	0.606	0.611	0.615	0.62	0.625	0.63	0.635	0.64	0.646	0.651	0.656	0.661	0.666	0.672	0.677	0.681	0.685	0.689	0.691
Botswana	0.404	0.414	0.423	0.432	0.441	0.451	0.461	0.471	0.484	0.497	0.508	0.518	0.527	0.536	0.544	0.552	0.56	0.569	0.578	0.587	0.595	0.602	0.609	0.616	0.623	0.631	0.638	0.645	0.65	0.656	0.662	0.669	0.675	0.682	0.689	0.695	0.701
Lesotho	0.323	0.328	0.333	0.337	0.341	0.345	0.349	0.353	0.358	0.364	0.37	0.376	0.383	0.39	0.397	0.403	0.41	0.417	0.423	0.429	0.435	0.442	0.448	0.455	0.462	0.469	0.475	0.482	0.489	0.496	0.504	0.513	0.521	0.529	0.538	0.545	0.552
Namibia	0.374	0.38	0.385	0.39	0.395	0.399	0.404	0.41	0.416	0.422	0.427	0.433	0.441	0.45	0.459	0.469	0.478	0.488	0.497	0.505	0.514	0.522	0.531	0.539	0.547	0.555	0.563	0.569	0.575	0.58	0.586	0.591	0.597	0.604	0.611	0.618	0.624
South Africa	0.542	0.548	0.554	0.559	0.564	0.569	0.574	0.579	0.584	0.59	0.596	0.601	0.606	0.613	0.619	0.624	0.63	0.636	0.64	0.645	0.649	0.653	0.658	0.663	0.669	0.675	0.682	0.688	0.695	0.701	0.707	0.714	0.719	0.724	0.727	0.73	0.734
Swaziland	0.384	0.391	0.398	0.404	0.41	0.415	0.421	0.429	0.437	0.446	0.454	0.462	0.47	0.477	0.484	0.491	0.498	0.504	0.509	0.514	0.518	0.522	0.525	0.528	0.531	0.534	0.537	0.538	0.538	0.539	0.544	0.553	0.562	0.571	0.578	0.584	0.589
Zimbabwe	0.357	0.364	0.37	0.377	0.384	0.39	0.397	0.403	0.412	0.422	0.432	0.443	0.452	0.461	0.47	0.477	0.486	0.493	0.498	0.502	0.505	0.507	0.508	0.506	0.503	0.498	0.492	0.485	0.477	0.472	0.469	0.47	0.474	0.479	0.485	0.491	0.497
Eastern Cape	0.495	0.501	0.506	0.511	0.516	0.521	0.524	0.528	0.533	0.538	0.543	0.547	0.551	0.557	0.562	0.567	0.573	0.578	0.583	0.587	0.591	0.596	0.601	0.606	0.612	0.618	0.626	0.633	0.64	0.647	0.653	0.66	0.665	0.67	0.673	0.676	0.679
Free State	0.546	0.551	0.556	0.56	0.564	0.568	0.572	0.576	0.581	0.586	0.591	0.597	0.602	0.609	0.617	0.624	0.631	0.637	0.641	0.645	0.649	0.652	0.656	0.661	0.666	0.672	0.678	0.684	0.69	0.696	0.702	0.708	0.714	0.718	0.721	0.724	0.727
Gauteng	0.656	0.66	0.664	0.667	0.671	0.674	0.677	0.681	0.684	0.688	0.692	0.695	0.698	0.702	0.705	0.707	0.709	0.712	0.715	0.718	0.721	0.724	0.728	0.731	0.735	0.74	0.744	0.749	0.754	0.759	0.763	0.768	0.773	0.775	0.778	0.78	0.782
KwaZulu-Natal	0.521	0.527	0.532	0.537	0.542	0.546	0.551	0.555	0.56	0.566	0.571	0.576	0.581	0.588	0.594	0.599	0.605	0.61	0.615	0.619	0.624	0.629	0.634	0.639	0.645	0.651	0.658	0.665	0.672	0.679	0.685	0.692	0.698	0.702	0.706	0.71	0.713
Limpopo	0.447	0.454	0.461	0.467	0.473	0.479	0.484	0.49	0.497	0.504	0.511	0.518	0.524	0.532	0.54	0.547	0.554	0.562	0.568	0.572	0.577	0.582	0.588	0.594	0.602	0.61	0.618	0.627	0.636	0.643	0.651	0.659	0.665	0.671	0.675	0.679	0.682
Mpumalanga	0.495	0.502	0.508	0.514	0.52	0.525	0.531	0.537	0.543	0.55	0.557	0.563	0.57	0.577	0.584	0.589	0.595	0.601	0.606	0.61	0.615	0.62	0.625	0.631	0.637	0.644	0.652	0.659	0.667	0.674	0.681	0.688	0.695	0.7	0.703	0.707	0.71
North-West	0.523	0.529	0.534	0.539	0.545	0.55	0.555	0.56	0.565	0.571	0.576	0.58	0.585	0.59	0.594	0.597	0.6	0.604	0.608	0.613	0.618	0.623	0.628	0.633	0.639	0.645	0.651	0.658	0.664	0.671	0.677	0.683	0.689	0.693	0.696	0.699	0.702
Northern Cape	0.545	0.55	0.555	0.559	0.564	0.568	0.572	0.576	0.58	0.585	0.59	0.594	0.598	0.603	0.607	0.61	0.614	0.619	0.623	0.626	0.63	0.633	0.637	0.641	0.646	0.652	0.658	0.664	0.67	0.675	0.681	0.688	0.693	0.697	0.7	0.704	0.707
Western Cape	0.653	0.657	0.66	0.662	0.665	0.668	0.67	0.672	0.675	0.678	0.681	0.684	0.687	0.691	0.695	0.698	0.703	0.708	0.711	0.713	0.715	0.717	0.72	0.722	0.725	0.728	0.732	0.736	0.74	0.744	0.748	0.752	0.756	0.758	0.76	0.762	0.764
Western Sub-Saharan Africa	0.254	0.259	0.263	0.268	0.272	0.276	0.279	0.281	0.282	0.285	0.287	0.29	0.293	0.297	0.301	0.305	0.31	0.314	0.318	0.322	0.326	0.33	0.335	0.339	0.345	0.351	0.357	0.364	0.37	0.376	0.383	0.391	0.399	0.407	0.415	0.423	0.428
Benin	0.171	0.175	0.178	0.182	0.187	0.192	0.197	0.202	0.207	0.212	0.217	0.222	0.227	0.233	0.237	0.242	0.247	0.252	0.257	0.262	0.267	0.273	0.278	0.283	0.288	0.293	0.298	0.304	0.31	0.315	0.32	0.326	0.33	0.335	0.34	0.346	0.351
Burkina Faso	0.103	0.106	0.108	0.109	0.111	0.113	0.115	0.117	0.12	0.123	0.125	0.13	0.136	0.141	0.146	0.15	0.155	0.16	0.165	0.169	0.173	0.178	0.182	0.186	0.191	0.195	0.2	0.206	0.212	0.217	0.225	0.234	0.244	0.253	0.262	0.269	0.274
Cameroon	0.283	0.289	0.296	0.302	0.309	0.316	0.322	0.327	0.332	0.337	0.341	0.345	0.349	0.353	0.356	0.36	0.364	0.369	0.374	0.379	0.383	0.388	0.392	0.396	0.4	0.403	0.406	0.409	0.412	0.415	0.419	0.423	0.428	0.433	0.439	0.445	0.451
Cape Verde	0.241	0.248	0.256	0.263	0.271	0.279	0.287	0.294	0.302	0.31	0.317	0.325	0.332	0.34	0.349	0.358	0.367	0.377	0.387	0.399	0.41	0.421	0.431	0.442	0.452	0.462	0.473	0.484	0.495	0.505	0.514	0.524	0.534	0.542	0.551	0.559	0.566
Chad	0.158	0.159	0.159	0.161	0.163	0.166	0.169	0.172	0.175	0.179	0.183	0.187	0.191	0.194	0.197	0.2	0.203	0.206	0.209	0.212	0.214	0.217	0.219	0.222	0.228	0.235	0.241	0.247	0.252	0.257	0.262	0.268	0.274	0.28	0.285	0.29	0.295
Cote d'Ivoire	0.225	0.226	0.227	0.228	0.23	0.234	0.241	0.249	0.258	0.267	0.277	0.287	0.297	0.306	0.313	0.32	0.327	0.332	0.337	0.341	0.345	0.348	0.351	0.353	0.355	0.358	0.36	0.363	0.366	0.369	0.373	0.377	0.382	0.388	0.394	0.401	0.407
The Gambia	0.205	0.21	0.215	0.221	0.226	0.232	0.237	0.242	0.247	0.251	0.254	0.255	0.255	0.255	0.254	0.254	0.255	0.259	0.264	0.269	0.274	0.28	0.285	0.29	0.295	0.3	0.305	0.31	0.315	0.32	0.325	0.329	0.334	0.339	0.344	0.349	0.352
Ghana	0.309	0.313	0.315	0.317	0.32	0.323	0.326	0.33	0.338	0.347	0.356	0.366	0.375	0.384	0.392	0.401	0.409	0.416	0.421	0.426	0.429	0.431	0.433	0.436	0.438	0.44	0.443	0.446	0.448	0.449	0.451	0.457	0.466	0.477	0.486	0.494	0.499
Guinea	0.181	0.184	0.188	0.191	0.194	0.197	0.2	0.203	0.207	0.21	0.213	0.217	0.22	0.223	0.226	0.23	0.233	0.237	0.24	0.243	0.246	0.249	0.251	0.254	0.257	0.26	0.264	0.268	0.273	0.277	0.282	0.288	0.293	0.299	0.305	0.311	0.316
Guinea-Bissau	0.176	0.179	0.182	0.184	0.187	0.19	0.193	0.197	0.2	0.204	0.208	0.213	0.217	0.222	0.226	0.231	0.236	0.242	0.246	0.25	0.254	0.258	0.262	0.266	0.27	0.274	0.279	0.283	0.288	0.293	0.298	0.305	0.31	0.316	0.322	0.328	0.333
Liberia	0.198	0.201	0.203	0.205	0.207	0.209	0.211	0.213	0.216	0.219	0.218	0.218	0.215	0.211	0.204	0.196	0.186	0.183	0.184	0.19	0.204	0.218	0.234	0.241	0.247	0.253	0.258	0.264	0.27	0.276	0.282	0.289	0.298	0.307	0.314	0.321	0.327
Mali	0.114	0.117	0.118	0.12	0.122	0.124	0.127	0.129	0.131	0.135	0.138	0.142	0.146	0.15	0.153	0.157	0.161	0.164	0.168	0.172	0.176	0.18	0.185	0.19	0.195	0.2	0.205	0.211	0.217	0.223	0.23	0.237	0.243	0.249	0.256	0.262	0.267
Mauritania	0.273	0.279	0.283	0.288	0.293	0.297	0.302	0.307	0.312	0.317	0.322	0.327	0.332	0.338	0.344	0.349	0.356	0.361	0.366	0.372	0.376	0.381	0.386	0.391	0.396	0.402	0.41	0.417	0.424	0.431	0.438	0.445	0.452	0.46	0.468	0.475	0.482
Niger	0.119	0.121	0.123	0.126	0.127	0.129	0.131	0.133	0.135	0.136	0.138	0.139	0.14	0.141	0.141	0.141	0.142	0.143	0.145	0.146	0.148	0.149	0.152	0.154	0.156	0.158	0.161	0.163	0.166	0.169	0.172	0.175	0.179	0.183	0.188	0.192	0.195
Nigeria	0.289	0.296	0.303	0.309	0.314	0.32	0.323	0.323	0.323	0.322	0.322	0.322	0.324	0.327	0.33	0.333	0.337	0.341	0.345	0.349	0.353	0.358	0.364	0.369	0.377	0.385	0.392	0.4	0.407	0.414	0.423	0.433	0.443	0.454	0.464	0.473	0.479
Sao Tome and Principe	0.237	0.243	0.248	0.254	0.259	0.265	0.27	0.275	0.279	0.284	0.289	0.294	0.299	0.304	0.31	0.315	0.32	0.326	0.331	0.336	0.34	0.346	0.351	0.357	0.363	0.369	0.376	0.382	0.388	0.393	0.398	0.403	0.408	0.413	0.42	0.427	0.434
Senegal	0.197	0.2	0.204	0.208	0.211	0.216	0.22	0.225	0.23	0.235	0.24	0.245	0.251	0.257	0.263	0.27	0.276	0.283	0.29	0.295	0.299	0.302	0.304	0.307	0.309	0.312	0.314	0.317	0.32	0.324	0.327	0.331	0.336	0.34	0.345	0.35	0.354
Sierra Leone	0.186	0.189	0.192	0.195	0.198	0.201	0.204	0.207	0.21	0.214	0.217	0.222	0.225	0.229	0.233	0.237	0.241	0.243	0.246	0.247	0.249	0.252	0.257	0.262	0.268	0.273	0.279	0.286	0.292	0.299	0.306	0.313	0.322	0.332	0.341	0.348	0.352
Togo	0.208	0.212	0.215	0.218	0.222	0.225	0.228	0.232	0.236	0.242	0.248	0.255	0.261	0.267	0.273	0.28	0.288	0.296	0.301	0.307	0.312	0.317	0.321	0.326	0.331	0.336	0.341	0.346	0.351	0.356	0.361	0.367	0.373	0.379	0.385	0.392	0.397

Appendix Table 8: GBD 2016 location hierarchy with levels	
**Location**	**Level**
Global	0
Southeast Asia, East Asia, and Oceania	1
East Asia	2
China	3
Anhui	4
Beijing	4
Chongqing	4
Fujian	4
Gansu	4
Guangdong	4
Guangxi	4
Guizhou	4
Hainan	4
Hebei	4
Heilongjiang	4
Henan	4
Hong Kong Special Administrative Region of China	4
Hubei	4
Hunan	4
Inner Mongolia	4
Jiangsu	4
Jiangxi	4
Jilin	4
Liaoning	4
Macao Special Administrative Region of China	4
Ningxia	4
Qinghai	4
Shaanxi	4
Shandong	4
Shanghai	4
Shanxi	4
Sichuan	4
Tianjin	4
Tibet	4
Xinjiang	4
Yunnan	4
Zhejiang	4
North Korea	3
Taiwan (Province of China)	3
Southeast Asia	2
Cambodia	3
Indonesia	3
Aceh	4
Bali	4
Bangka Belitung	4
Banten	4

Appendix Table 8: GBD 2016 location hierarchy with levels

Location	Level
Bengkulu	4
Gorontalo	4
Jakarta	4
Jambi	4
Cental Java	4
West Java	4
East Java	4
West Kalimantan	4
South Kalimantan	4
Central Kalimantan	4
East Kalimantan	4
North Kalimantan	4
Riau Islands	4
Lampung	4
Maluku	4
North Maluku	4
Nusa Tenggara Barat	4
East Nusa Tenggara	4
Papua	4
West Papua	4
Riau	4
West Sulawesi	4
South Sumatera	4
Central Sulawesi	4
Southeast Sulawesi	4
North Sulawesi	4
West Sumatera	4
South Sumatera	4
North Sumatera	4
Yogyakarta	4
Laos	3
Malaysia	3
Maldives	3
Mauritius	3
Myanmar	3
Philippines	3
Sri Lanka	3
Seychelles	3
Thailand	3
Timor-Leste	3
Vietnam	3
Oceania	2
American Samoa	3
Federated States of Micronesia	3
Fiji	3
Guam	3

Appendix Table 8: GBD 2016 location hierarchy with levels

Location	Level
Kiribati	3
Marshall Islands	3
Northern Mariana Islands	3
Papua New Guinea	3
Samoa	3
Solomon Islands	3
Tonga	3
Vanuatu	3
Central Europe, Eastern Europe, and Central Asia	1
Central Asia	2
Armenia	3
Azerbaijan	3
Georgia	4
Georgia	3
Kazakhstan	3
Kyrgyzstan	3
Mongolia	3
Tajikistan	3
Turkmenistan	3
Uzbekistan	3
Central Europe	2
Albania	3
Bosnia and Herzegovina	3
Bulgaria	3
Croatia	3
Czech Republic	3
Hungary	3
Macedonia	3
Montenegro	3
Poland	3
Romania	3
Serbia	3
Slovakia	3
Slovenia	3
Eastern Europe	2
Belarus	3
Estonia	3
Latvia	3
Lithuania	3
Moldova	3
Russia	3
Ukraine	3
High-income	1
High-income Asia Pacific	2
Brunei	3
Japan	3

Appendix Table 8: GBD 2016 location hierarchy with levels

Location	Level
Aichi	4
Akita	4
Aomori	4
Chiba	4
Ehime	4
Fukui	4
Fukuoka	4
Fukushima	4
Gifu	4
Gunma	4
Hiroshima	4
Hokkaidō	4
Hyōgo	4
Ibaraki	4
Ishikawa	4
Iwate	4
Kagawa	4
Kagoshima	4
Kanagawa	4
Kōchi	4
Kumamoto	4
Kyōto	4
Mie	4
Miyagi	4
Miyazaki	4
Nagano	4
Nagasaki	4
Nara	4
Niigata	4
Ôita	4
Okayama	4
Okinawa	4
Ōsaka	4
Saga	4
Saitama	4
Shiga	4
Shimane	4
Shizuoka	4
Tochigi	4
Tokushima	4
Tōkyō	4
Tottori	4
Toyama	4
Wakayama	4
Yamagata	4
Yamaguchi	4

Appendix Table 8: GBD 2016 location hierarchy with levels	
**Location**	**Level**
Yamanashi	4
South Korea	3
Singapore	3
Australasia	2
Australia	3
New Zealand	3
Western Europe	2
Andorra	3
Austria	3
Belgium	3
Cyprus	3
Denmark	3
Finland	3
France	3
Germany	3
Greece	3
Iceland	3
Ireland	3
Israel	3
Italy	3
Luxembourg	3
Malta	3
Netherlands	3
Norway	3
Portugal	3
Spain	3
Sweden	3
Stockholm	4
Sweden except Stockholm	4
Switzerland	3
United Kingdom	3
England	4
East Midlands	5
Derby	6
Derbyshire	6
Leicester	6
Leicestershire	6
Lincolnshire	6
Northamptonshire	6
Nottingham	6
Nottinghamshire	6
Rutland	6
East of England	5
Bedford	6
Cambridgeshire	6
Central Bedfordshire	6

Appendix Table 8: GBD 2016 location hierarchy with levels

Location	Level
Essex	6
Hertfordshire	6
Luton	6
Norfolk	6
Peterborough	6
Southend-on-Sea	6
Suffolk	6
Thurrock	6
Greater London	5
Barking and Dagenham	6
Barnet	6
Bexley	6
Brent	6
Bromley	6
Camden	6
Croydon	6
Ealing	6
Enfield	6
Greenwich	6
Hackney	6
Hammersmith and Fulham	6
Haringey	6
Harrow	6
Havering	6
Hillingdon	6
Hounslow	6
Islington	6
Kensington and Chelsea	6
Kingston upon Thames	6
Lambeth	6
Lewisham	6
Merton	6
Newham	6
Redbridge	6
Richmond upon Thames	6
Southwark	6
Sutton	6
Tower Hamlets	6
Waltham Forest	6
Wandsworth	6
Westminster	6
North East England	5
County Durham	6
Darlington	6
Gateshead	6
Hartlepool	6

Appendix Table 8: GBD 2016 location hierarchy with levels

Location	Level
Middlesbrough	6
Newcastle upon Tyne	6
North Tyneside	6
Northumberland	6
Redcar and Cleveland	6
South Tyneside	6
Stockton-on-Tees	6
Sunderland	6
North West England	5
Blackburn with Darwen	6
Blackpool	6
Bolton	6
Bury	6
Cheshire East	6
Cheshire West and Chester	6
Cumbria	6
Halton	6
Knowsley	6
Lancashire	6
Liverpool	6
Manchester	6
Oldham	6
Rochdale	6
Salford	6
Sefton	6
St Helens	6
Stockport	6
Tameside	6
Trafford	6
Warrington	6
Wigan	6
Wirral	6
South East England	5
Bracknell Forest	6
Brighton and Hove	6
Buckinghamshire	6
East Sussex	6
Hampshire	6
Isle of Wight	6
Kent	6
Medway	6
Milton Keynes	6
Oxfordshire	6
Portsmouth	6
Reading	6
Slough	6

Appendix Table 8: GBD 2016 location hierarchy with levels

Location	Level
Southampton	6
Surrey	6
West Berkshire	6
West Sussex	6
Windsor and Maidenhead	6
Wokingham	6
South West England	5
Bath and North East Somerset	6
Bournemouth	6
Bristol, City of	6
Cornwall	6
Devon	6
Dorset	6
Gloucestershire	6
North Somerset	6
Plymouth	6
Poole	6
Somerset	6
South Gloucestershire	6
Swindon	6
Torbay	6
Wiltshire	6
West Midlands	5
Birmingham	6
Coventry	6
Dudley	6
Herefordshire, County of	6
Sandwell	6
Shropshire	6
Solihull	6
Staffordshire	6
Stoke-on-Trent	6
Telford and Wrekin	6
Walsall	6
Warwickshire	6
Wolverhampton	6
Worcestershire	6
Yorkshire and the Humber	5
Barnsley	6
Bradford	6
Calderdale	6
Doncaster	6
East Riding of Yorkshire	6
Kingston upon Hull, City of	6
Kirklees	6
Leeds	6

Appendix Table 8: GBD 2016 location hierarchy with levels	
**Location**	**Level**
North East Lincolnshire	6
North Lincolnshire	6
North Yorkshire	6
Rotherham	6
Sheffield	6
Wakefield	6
York	6
Northern Ireland	4
Scotland	4
Wales	4
Southern Latin America	2
Argentina	3
Chile	3
Uruguay	3
High-income North America	2
Canada	3
Greenland	3
USA	3
Alabama	4
Alaska	4
Arizona	4
Arkansas	4
California	4
Colorado	4
Connecticut	4
Delaware	4
District of Columbia	4
Florida	4
Georgia	3
Georgia	4
Hawaii	4
Idaho	4
Illinois	4
Indiana	4
Iowa	4
Kansas	4
Kentucky	4
Louisiana	4
Maine	4
Maryland	4
Massachusetts	4
Michigan	4
Minnesota	4
Mississippi	4
Missouri	4
Montana	4

Appendix Table 8: GBD 2016 location hierarchy with levels

Location	Level
Nebraska	4
Nevada	4
New Hampshire	4
New Jersey	4
New Mexico	4
New York	4
North Carolina	4
North Dakota	4
Ohio	4
Oklahoma	4
Oregon	4
Pennsylvania	4
Rhode Island	4
South Carolina	4
South Dakota	4
Tennessee	4
Texas	4
Utah	4
Vermont	4
Virginia	4
Washington	4
West Virginia	4
Wisconsin	4
Wyoming	4
Latin America and Caribbean	1
Caribbean	2
Antigua and Barbuda	3
The Bahamas	3
Barbados	3
Belize	3
Bermuda	3
Cuba	3
Dominica	3
Dominican Republic	3
Grenada	3
Guyana	3
Haiti	3
Jamaica	3
Puerto Rico	3
Saint Lucia	3
Saint Vincent and the Grenadines	3
Suriname	3
Trinidad and Tobago	3
Virgin Islands, U.S.	3
Andean Latin America	2
Bolivia	3

Appendix Table 8: GBD 2016 location hierarchy with levels	
**Location**	**Level**
Ecuador	3
Peru	3
Central Latin America	2
Colombia	3
Costa Rica	3
El Salvador	3
Guatemala	3
Honduras	3
Mexico	3
Aguascalientes	4
Baja California	4
Baja California Sur	4
Campeche	4
Chiapas	4
Chihuahua	4
Coahuila	4
Colima	4
Distrito Federal	4
Distrito Federal	4
Durango	4
Guanajuato	4
Guerrero	4
Hidalgo	4
Jalisco	4
México	4
Michoacán de Ocampo	4
Morelos	4
Nayarit	4
Nuevo León	4
Oaxaca	4
Puebla	4
Querétaro	4
Quintana Roo	4
San Luis Potosí	4
Sinaloa	4
Sonora	4
Tabasco	4
Tamaulipas	4
Tlaxcala	4
Veracruz de Ignacio de la Llave	4
Yucatán	4
Zacatecas	4
Nicaragua	3
Panama	3
Venezuela	3
Tropical Latin America	2

Appendix Table 8: GBD 2016 location hierarchy with levels	
**Location**	**Level**
Brazil	3
Acre	4
Alagoas	4
Amapá	4
Amazonas	4
Bahia	4
Ceará	4
Distrito Federal	4
Distrito Federal	4
Espírito Santo	4
Goiás	4
Maranhão	4
Mato Grosso	4
Mato Grosso do Sul	4
Minas Gerais	4
Pará	4
Paraíba	4
Paraná	4
Pernambuco	4
Piaui	4
Rio de Janeiro	4
Rio Grande do Norte	4
Rio Grande do Sul	4
Rondônia	4
Roraima	4
Santa Catarina	4
São Paulo	4
Sergipe	4
Tocantins	4
Paraguay	3
North Africa and Middle East	1
North Africa and Middle East	2
Afghanistan	3
Algeria	3
Bahrain	3
Egypt	3
Iran	3
Iraq	3
Jordan	3
Kuwait	3
Lebanon	3
Libya	3
Morocco	3
Palestine	3
Oman	3
Qatar	3

Appendix Table 8: GBD 2016 location hierarchy with levels

Location	Level
Saudi Arabia	3
'Asir	4
Bahah	4
Eastern Province	4
Ha'il	4
Jawf	4
Jizan	4
Madinah	4
Makkah	4
Najran	4
Northern Borders	4
Qassim	4
Riyadh	4
Tabuk	4
Sudan	3
Syria	3
Tunisia	3
Turkey	3
United Arab Emirates	3
Yemen	3
South Asia	1
South Asia	2
Bangladesh	3
Bhutan	3
India	3
Andhra Pradesh	4
Andhra Pradesh, Rural	5
Andhra Pradesh, Urban	5
Arunāchal Pradesh	4
Arunāchal Pradesh, Rural	5
Arunāchal Pradesh, Urban	5
Assam	4
Assam, Rural	5
Assam, Urban	5
Bihār	4
Bihār, Rural	5
Bihār, Urban	5
Chhattīsgarh	4
Chhattīsgarh, Rural	5
Chhattīsgarh, Urban	5
Delhi	4
Delhi, Rural	5
Delhi, Urban	5
Goa	4
Goa, Rural	5
Goa, Urban	5

Appendix Table 8: GBD 2016 location hierarchy with levels	
**Location**	**Level**
Gujarāt	4
Gujarāt, Rural	5
Gujarāt, Urban	5
Haryāna	4
Haryāna, Rural	5
Haryāna, Urban	5
Himachal Pradesh	4
Himachal Pradesh, Rural	5
Himachal Pradesh, Urban	5
Jammu and Kashmīr	4
Jammu and Kashmīr, Rural	5
Jammu and Kashmīr, Urban	5
Jharkhand	4
Jharkhand, Rural	5
Jharkhand, Urban	5
Karnātaka	4
Karnātaka, Rural	5
Karnātaka, Urban	5
Kerala	4
Kerala, Rural	5
Kerala, Urban	5
Madhya Pradesh	4
Madhya Pradesh, Rural	5
Madhya Pradesh, Urban	5
Mahārāshtra	4
Mahārāshtra, Rural	5
Mahārāshtra, Urban	5
Manipur	4
Manipur, Rural	5
Manipur, Urban	5
Meghālaya	4
Meghālaya, Rural	5
Meghālaya, Urban	5
Mizoram	4
Mizoram, Rural	5
Mizoram, Urban	5
Nāgāland	4
Nāgāland, Rural	5
Nāgāland, Urban	5
Orissa	4
Orissa, Rural	5
Orissa, Urban	5
Punjab	4
Punjab, Rural	5
Punjab, Urban	5
Rājasthān	4

Appendix Table 8: GBD 2016 location hierarchy with levels

Location	Level
Rājasthān, Rural	5
Rājasthān, Urban	5
Sikkim	4
Sikkim, Rural	5
Sikkim, Urban	5
Tamil Nādu	4
Tamil Nādu, Rural	5
Tamil Nādu, Urban	5
Telangana	4
Telangana, Rural	5
Telangana, Urban	5
Tripura	4
Tripura, Rural	5
Tripura, Urban	5
Uttar Pradesh	4
Uttar Pradesh, Rural	5
Uttar Pradesh, Urban	5
Uttarakhand	4
Uttarakhand, Rural	5
Uttarakhand, Urban	5
West Bengal	4
West Bengal, Rural	5
West Bengal, Urban	5
The Six Minor Territories	4
The Six Minor Territories, Rural	5
The Six Minor Territories, Urban	5
Nepal	3
Pakistan	3
Sub-Saharan Africa	1
Central Sub-Saharan Africa	2
Angola	3
the Central African Republic	3
Congo (Brazzaville)	3
Democratic Republic of the Congo	3
Equatorial Guinea	3
Gabon	3
Eastern Sub-Saharan Africa	2
Burundi	3
Comoros	3
Djibouti	3
Eritrea	3
Ethiopia	3
Kenya	3
Baringo	4
Bomet	4
Bungoma	4

Appendix Table 8: GBD 2016 location hierarchy with levels

Location	Level
Busia	4
Elgeyo-Marakwet	4
Embu	4
Garissa	4
HomaBay	4
Isiolo	4
Kajiado	4
Kakamega	4
Kericho	4
Kiambu	4
Kilifi	4
Kirinyaga	4
Kisii	4
Kisumu	4
Kitui	4
Kwale	4
Laikipia	4
Lamu	4
Machakos	4
Makueni	4
Mandera	4
Marsabit	4
Meru	4
Migori	4
Mombasa	4
Murang’a	4
Nairobi	4
Nakuru	4
Nandi	4
Narok	4
Nyamira	4
Nyandarua	4
Nyeri	4
Samburu	4
Siaya	4
TaitaTaveta	4
TanaRiver	4
TharakaNithi	4
TransNzoia	4
Turkana	4
UasinGishu	4
Vihiga	4
Wajir	4
WestPokot	4
Madagascar	3
Malawi	3

Appendix Table 8: GBD 2016 location hierarchy with levels	
**Location**	**Level**
Mozambique	3
Rwanda	3
Somalia	3
South Sudan	3
Tanzania	3
Uganda	3
Zambia	3
Southern Sub-Saharan Africa	2
Botswana	3
Lesotho	3
Namibia	3
South Africa	3
Eastern Cape	4
Free State	4
Gauteng	4
KwaZulu-Natal	4
Limpopo	4
Mpumalanga	4
North-West	4
Northern Cape	4
Western Cape	4
Swaziland	3
Zimbabwe	3
Western Sub-Saharan Africa	2
Benin	3
Burkina Faso	3
Cameroon	3
Cape Verde	3
Chad	3
Cote d'Ivoire	3
The Gambia	3
Ghana	3
Guinea	3
Guinea-Bissau	3
Liberia	3
Mali	3
Mauritania	3
Niger	3
Nigeria	3
Sao Tome and Principe	3
Senegal	3
Sierra Leone	3
Togo	3

Appendix Table 9. Causes included in the 2016 Global Burden of Disease Study

Cause level	Cause	Cause of death	Cause of burden
0	All causes	X	X
1	Communicable, maternal, neonatal, and nutritional disorders	X	X
2	HIV/AIDS and tuberculosis	X	X
3	Tuberculosis	X	X
4	Drug-sensitive tuberculosis	X	X
4	Multidrug-resistant tuberculosis without extensive drug resistance	X	X
4	Extensively drug-resistant tuberculosis	X	X
4	Latent tuberculosis infection		X
3	HIV/AIDS	X	X
4	Drug-sensitive HIV/AIDS - Tuberculosis	X	X
4	Multidrug-resistant HIV/AIDS - Tuberculosis without extensive drug resistance	X	X
4	Extensively drug-resistant HIV/AIDS - Tuberculosis	X	X
4	HIV/AIDS resulting in other diseases	X	X
2	Diarrhoea, lower respiratory infections, and other common infectious diseases	X	X
3	Diarrhoeal diseases	X	X
3	Intestinal infectious diseases	X	X
4	Typhoid fever	X	X
4	Paratyphoid fever	X	X
4	Other intestinal infectious diseases	X	X
3	Lower respiratory infections	X	X
3	Upper respiratory infections	X	X
3	Otitis media	X	X
3	Meningitis	X	X
4	Pneumococcal meningitis	X	X
4	H influenzae type B meningitis	X	X
4	Meningococcal infection	X	X

Appendix Table 9. Causes included in the 2016 Global Burden of Disease Study

Cause level	Cause	Cause of death	Cause of burden
4	Other meningitis	X	X
3	Encephalitis	X	X
3	Diphtheria	X	X
3	Whooping cough	X	X
3	Tetanus	X	X
3	Measles	X	X
3	Varicella and herpes zoster	X	X
2	Neglected tropical diseases and malaria	X	X
3	Malaria	X	X
3	Chagas disease	X	X
3	Leishmaniasis	X	X
4	Visceral leishmaniasis	X	X
4	Cutaneous and mucocutaneous leishmaniasis		X
3	African trypanosomiasis	X	X
3	Schistosomiasis	X	X
3	Cysticercosis	X	X
3	Cystic echinococcosis	X	X
3	Lymphatic filariasis		X
3	Onchocerciasis		X
3	Trachoma		X
3	Dengue	X	X
3	Yellow fever	X	X
3	Rabies	X	X
3	Intestinal nematode infections	X	X
4	Ascariasis	X	X
4	Trichuriasis		X

Appendix Table 9. Causes included in the 2016 Global Burden of Disease Study

Cause level	Cause	Cause of death	Cause of burden
4	Hookworm disease		X
3	Food-borne trematodiases		X
3	Leprosy		X
3	Ebola	X	X
3	Zika virus	X	X
3	Guinea worm disease		X
3	Other neglected tropical diseases	X	X
2	Maternal disorders	X	X
3	Maternal haemorrhage	X	X
3	Maternal sepsis and other pregnancy related infections	X	X
3	Maternal hypertensive disorders	X	X
3	Maternal obstructed labour and uterine rupture	X	X
3	Maternal abortion, miscarriage, and ectopic pregnancy	X	X
3	Indirect maternal deaths	X	
3	Late maternal deaths	X	
3	Maternal deaths aggravated by HIV/AIDS	X	
3	Other maternal disorders	X	X
2	Neonatal disorders	X	X
3	Neonatal preterm birth complications	X	X
3	Neonatal encephalopathy due to birth asphyxia and trauma	X	X
3	Neonatal sepsis and other neonatal infections	X	X
3	Hemolytic disease and other neonatal jaundice	X	X
3	Other neonatal disorders	X	X
2	Nutritional deficiencies	X	X
3	Protein-energy malnutrition	X	X
3	Iodine deficiency	X	X

Appendix Table 9. Causes included in the 2016 Global Burden of Disease Study			
**Cause level**	**Cause**	**Cause of death**	**Cause of burden**
3	Vitamin A deficiency		X
3	Iron-deficiency anaemia	X	X
3	Other nutritional deficiencies	X	X
2	Other communicable, maternal, neonatal, and nutritional diseases	X	X
3	Sexually transmitted diseases excluding HIV	X	X
4	Syphilis	X	X
4	Chlamydial infection	X	X
4	Gonococcal infection	X	X
4	Trichomoniasis		X
4	Genital herpes		X
4	Other sexually transmitted diseases	X	X
3	Hepatitis	X	X
4	Acute hepatitis A	X	X
4	Hepatitis B	X	X
4	Hepatitis C	X	X
4	Acute hepatitis E	X	X
3	Other infectious diseases	X	X
1	Non-communicable diseases	X	X
2	Neoplasms	X	X
3	Lip and oral cavity cancer	X	X
3	Nasopharynx cancer	X	X
3	Other pharynx cancer	X	X
3	Oesophageal cancer	X	X
3	Stomach cancer	X	X
3	Colon and rectum cancer	X	X
3	Liver cancer	X	X

Appendix Table 9. Causes included in the 2016 Global Burden of Disease Study

Cause level	Cause	Cause of death	Cause of burden
4	Liver cancer due to hepatitis B	X	X
4	Liver cancer due to hepatitis C	X	X
4	Liver cancer due to alcohol use	X	X
4	Liver cancer due to other causes	X	X
3	Gallbladder and biliary tract cancer	X	X
3	Pancreatic cancer	X	X
3	Larynx cancer	X	X
3	Tracheal, bronchus, and lung cancer	X	X
3	Malignant skin melanoma	X	X
3	Non-melanoma skin cancer	X	X
4	Non-melanoma skin cancer (squamous-cell carcinoma)	X	X
4	Non-melanoma skin cancer (basal-cell carcinoma)		X
3	Breast cancer	X	X
3	Cervical cancer	X	X
3	Uterine cancer	X	X
3	Ovarian cancer	X	X
3	Prostate cancer	X	X
3	Testicular cancer	X	X
3	Kidney cancer	X	X
3	Bladder cancer	X	X
3	Brain and nervous system cancer	X	X
3	Thyroid cancer	X	X
3	Mesothelioma	X	X
3	Hodgkin lymphoma	X	X
3	Non-Hodgkin's lymphoma	X	X
3	Multiple myeloma	X	X

Appendix Table 9. Causes included in the 2016 Global Burden of Disease Study

Cause level	Cause	Cause of death	Cause of burden
3	Leukaemia	X	X
4	Acute lymphoid leukaemia	X	X
4	Chronic lymphoid leukaemia	X	X
4	Acute myeloid leukaemia	X	X
4	Chronic myeloid leukaemia	X	X
4	Other leukaemia	X	X
3	Other neoplasms	X	X
2	Cardiovascular diseases	X	X
3	Rheumatic heart disease	X	X
3	Ischaemic heart disease	X	X
3	Cerebrovascular disease	X	X
4	Ischaemic stroke	X	X
4	Hemorrhagic stroke	X	X
3	Hypertensive heart disease	X	X
3	Cardiomyopathy and myocarditis	X	X
4	Myocarditis	X	X
4	Alcoholic cardiomyopathy	X	X
4	Other cardiomyopathy	X	X
3	Atrial fibrillation and flutter	X	X
3	Aortic aneurysm	X	
3	Peripheral vascular disease	X	X
3	Endocarditis	X	X
3	Other cardiovascular and circulatory diseases	X	X
2	Chronic respiratory diseases	X	X
3	Chronic obstructive pulmonary disease	X	X
3	Pneumoconiosis	X	X

Appendix Table 9. Causes included in the 2016 Global Burden of Disease Study			
**Cause level**	**Cause**	**Cause of death**	**Cause of burden**
4	Silicosis	X	X
4	Asbestosis	X	X
4	Coal workers pneumoconiosis	X	X
4	Other pneumoconiosis	X	X
3	Asthma	X	X
3	Interstitial lung disease and pulmonary sarcoidosis	X	X
3	Other chronic respiratory diseases	X	X
2	Cirrhosis and other chronic liver diseases	X	X
3	Cirrhosis and other chronic liver diseases due to hepatitis B	X	X
3	Cirrhosis and other chronic liver diseases due to hepatitis C	X	X
3	Cirrhosis and other chronic liver diseases due to alcohol use	X	X
3	Cirrhosis and other chronic liver diseases due to other causes	X	X
2	Digestive diseases	X	X
3	Peptic ulcer disease	X	X
3	Gastritis and duodenitis	X	X
3	Appendicitis	X	X
3	Paralytic ileus and intestinal obstruction	X	X
3	Inguinal, femoral, and abdominal hernia	X	X
3	Inflammatory bowel disease	X	X
3	Vascular intestinal disorders	X	X
3	Gallbladder and biliary diseases	X	X
3	Pancreatitis	X	X
3	Other digestive diseases	X	X
2	Neurological disorders	X	X
3	Alzheimer's disease and other dementias	X	X
3	Parkinson's disease	X	X

Appendix Table 9. Causes included in the 2016 Global Burden of Disease Study

Cause level	Cause	Cause of death	Cause of burden
3	Epilepsy	X	X
3	Multiple sclerosis	X	X
3	Motor neuron disease	X	X
3	Migraine		X
3	Tension-type headache		X
3	Other neurological disorders	X	X
2	Mental and substance use disorders	X	X
3	Schizophrenia		X
3	Alcohol use disorders	X	X
3	Drug use disorders	X	X
4	Opioid use disorders	X	X
4	Cocaine use disorders	X	X
4	Amphetamine use disorders	X	X
4	Cannabis use disorders		X
4	Other drug use disorders	X	X
3	Depressive disorders		X
4	Major depressive disorder		X
4	Dysthymia		X
3	Bipolar disorder		X
3	Anxiety disorders		X
3	Eating disorders	X	X
4	Anorexia nervosa	X	X
4	Bulimia nervosa	X	X
3	Autistic spectrum disorders		X
4	Autism		X
4	Asperger syndrome and other autistic spectrum disorders		X

Appendix Table 9. Causes included in the 2016 Global Burden of Disease Study

Cause level	Cause	Cause of death	Cause of burden
3	Attention-deficit/hyperactivity disorder		X
3	Conduct disorder		X
3	Idiopathic developmental intellectual disability		X
3	Other mental and substance use disorders		X
2	Diabetes, urogenital, blood, and endocrine diseases	X	X
3	Diabetes mellitus	X	X
3	Acute glomerulonephritis	X	X
3	Chronic kidney disease	X	X
4	Chronic kidney disease due to diabetes mellitus	X	X
4	Chronic kidney disease due to hypertension	X	X
4	Chronic kidney disease due to glomerulonephritis	X	X
4	Chronic kidney disease due to other causes	X	X
3	Urinary diseases and male infertility	X	X
4	Interstitial nephritis and urinary tract infections	X	X
4	Urolithiasis	X	X
4	Benign prostatic hyperplasia		X
4	Male infertility		X
4	Other urinary diseases	X	X
3	Gynecological diseases	X	X
4	Uterine fibroids	X	X
4	Polycystic ovarian syndrome	X	X
4	Female infertility		X
4	Endometriosis	X	X
4	Genital prolapse	X	X
4	Premenstrual syndrome		X
4	Other gynecological diseases	X	X

Appendix Table 9. Causes included in the 2016 Global Burden of Disease Study

Cause level	Cause	Cause of death	Cause of burden
3	Hemoglobinopathies and hemolytic anaemias	X	X
4	Thalassemias	X	X
4	Thalassaemias trait		X
4	Sickle cell disorders	X	X
4	Sickle cell trait		X
4	G6PD deficiency	X	X
4	G6PD trait		X
4	Other hemoglobinopathies and hemolytic anaemias	X	X
3	Endocrine, metabolic, blood, and immune disorders	X	X
2	Musculoskeletal disorders	X	X
3	Rheumatoid arthritis	X	X
3	Osteoarthritis		X
3	Low back and neck pain		X
4	Low back pain		X
4	Neck pain		X
3	Gout		X
3	Other musculoskeletal disorders	X	X
2	Other non-communicable diseases	X	X
3	Congenital anomalies	X	X
4	Neural tube defects	X	X
4	Congenital heart anomalies	X	X
4	Orofacial clefts	X	X
4	Down's syndrome	X	X
4	Turner syndrome		X
4	Klinefelter syndrome		X
4	Other chromosomal abnormalities	X	X

Appendix Table 9. Causes included in the 2016 Global Burden of Disease Study

Cause level	Cause	Cause of death	Cause of burden
4	Congenital musculoskeletal and limb anomalies	X	X
4	Urogenital congenital anomalies	X	X
4	Digestive congenital anomalies	X	X
4	Other congenital anomalies	X	X
3	Skin and subcutaneous diseases	X	X
4	Eczema		X
4	Psoriasis		X
4	Cellulitis	X	X
4	Pyoderma	X	X
4	Scabies		X
4	Fungal skin diseases		X
4	Viral skin diseases		X
4	Acne vulgaris		X
4	Alopecia areata		X
4	Pruritus		X
4	Urticaria		X
4	Decubitus ulcer	X	X
4	Other skin and subcutaneous diseases	X	X
3	Sense organ diseases		X
4	Glaucoma		X
4	Cataract		X
4	Macular degeneration		X
4	Refraction and accommodation disorders		X
4	Age-related and other hearing loss		X
4	Other vision loss		X
4	Other sense organ diseases		X

Appendix Table 9. Causes included in the 2016 Global Burden of Disease Study

Cause level	Cause	Cause of death	Cause of burden
3	Oral disorders		X
4	Deciduous caries		X
4	Permanent caries		X
4	Periodontal disease		X
4	Edentulism and severe tooth loss		X
4	Other oral disorders		X
3	Sudden infant death syndrome	X	
1	Injuries	X	X
2	Transport injuries	X	X
3	Road injuries	X	X
4	Pedestrian road injuries	X	X
4	Cyclist road injuries	X	X
4	Motorcyclist road injuries	X	X
4	Motor vehicle road injuries	X	X
4	Other road injuries	X	X
3	Other transport injuries	X	X
2	Unintentional injuries	X	X
3	Falls	X	X
3	Drowning	X	X
3	Fire, heat, and hot substances	X	X
3	Poisonings	X	X
3	Exposure to mechanical forces	X	X
4	Unintentional firearm injuries	X	X
4	Unintentional suffocation	X	X
4	Other exposure to mechanical forces	X	X
3	Adverse effects of medical treatment	X	X

Appendix Table 9. Causes included in the 2016 Global Burden of Disease Study

Cause level	Cause	Cause of death	Cause of burden
3	Animal contact	X	X
4	Venomous animal contact	X	X
4	Non-venomous animal contact	X	X
3	Foreign body	X	X
4	Pulmonary aspiration and foreign body in airway	X	X
4	Foreign body in eyes		X
4	Foreign body in other body part	X	X
3	Environmental heat and cold exposure	X	X
3	Other unintentional injuries	X	X
2	Self-harm and interpersonal violence	X	X
3	Self-harm	X	X
4	Self-harm by firearm	X	X
4	Self-harm by other specified means	X	X
3	Interpersonal violence	X	X
4	Assault by firearm	X	X
4	Assault by sharp object	X	X
4	Sexual violence		X
4	Assault by other means	X	X
2	Forces of nature, conflict and terrorism, and executions and police conflict	X	X
3	Exposure to forces of nature	X	X
3	Conflict and terrorism	X	X
3	Executions and police conflict	X	X
